# Retand LTR-retrotransposons in plants: a long way from *pol* to 3’LTR

**DOI:** 10.1186/s13100-025-00354-z

**Published:** 2025-04-02

**Authors:** Carlos M. Vicient

**Affiliations:** Centre for Research in Agricultural Genomics (CRAG), CSIC-IRTA-UAB-UB, Campus UAB, Bellaterra, Barcelona 08193 Spain

**Keywords:** LTR retrotransposons, Domain annotation, Additional ORF, Antisense, Tandem repeat, Retrovirus

## Abstract

**Background:**

Plant Gypsy LTR-retrotransposons are classified into lineages according to the phylogenetic relationships of the reverse transcriptase. Retand is a lineage of non-chromovirus elements characterized by the presence of a long internal region compared to other lineages.

**Results:**

This work focuses on the identification and characterization of Potentially Recently Active Retand Elements (PRAREs) in 617 genomic sequence assemblies of *Viridiplantae* species. The Retand elements were considered PRAREs if their LTRs and insertion sequences were identical, and the sizes of their internal regions and LTRs did not differ by more than 2% from the consensus. A total of 2,735 PRAREs were identified, distributed in 122 clusters corresponding to 34 species, with copy numbers per cluster varying between 1 and 180. They are present in *Eudicotyledons* and *Liliopsida* but not in other groups of plants. Some PRAREs are non-autonomous elements, lacking some of the typical LTR retrotransposon coding domains. The size of the POL-3’LTR regions varies between 2,933 and 6,566 bp, and in all cases, includes potential coding regions oriented antisense to the *gag* and *pol* genes. 97% of the clusters contain antisense ORFs encoding the TRP28 protein domain of unknown function. The analysis of the consensus TRP28 domain indicates that it probably can bind DNA. About half of the PRAREs contain arrays of tandem repeats in the POL-3’LTR region.

**Conclusions:**

The large internal region of the Retand elements is due to the presence of a long POL-3’LTR region. This region frequently contains arrays of tandem repeats that contribute to the expansion of this area. The presence of antisense ORFs in the POL-3’LTR region is also a common feature in these elements, many of which encode proteins with conserved domains, especially the TRP28 domain. The possible function of these TRP28-containing proteins is unknown, but their potential DNA binding capacity and the comparison with similar genes in some retroviruses suggest that they may play a regulatory role in the Retand transposition process.

**Supplementary Information:**

The online version contains supplementary material available at 10.1186/s13100-025-00354-z.

## Background

In plant genomes, long terminal repeat (LTR) retrotransposons are the most abundant and widely distributed class of transposable elements (TEs), creating new copies via reverse transcription of the full-length RNA intermediate by the element-encoded reverse transcriptase (RT) [1]. LTR retrotransposons share many structural features with retroviruses [2], typically consisting of two identical regions at their ends, known as long terminal repeats (LTRs), which range from a few hundred base pairs to several kilobases in length, surrounding an internal region that encodes the essential proteins for their replication and contains a primer binding site (PBS) and a polypurine tract (PPT) at the 5′ and 3′ ends, respectively, both of which are used during the retrotransposition process. The LTR retrotransposons in plants display a target site duplication (TSD) that is usually 5 bp long [3].

LTR retrotransposons are classified into two superfamilies based on the organization of their coding domains: Ty1/copia (*Pseudoviridae* in the ICTV classification of viruses) and Ty3/gypsy (*Metaviridae*). Phylogenetic analyses of the RT, RH, and INT domains divide each of these superfamilies into different lineages [4]. In plants, the Copia superfamily is divided into Ale, Alesia, Angela, Bianca, Bryco, Lyco, Gymco, Ikeros, Ivana, Osser, SIRE, Tar, and Tork lineages, while the Gypsy superfamily comprises Athila, Clamyvir, CRM, Galadriel, Ogre, Phygy, Reina, Retand, Selgy, Tat, Tcn1, and Tekay. Although the classification is based on phylogenetic analysis, in some cases, the identified lineages are also differentiated by other characteristics shared by most members of a lineage. These features include the presence of an especially long internal region in the cases of the Ogre and Retand lineages [4], the presence of chromodomains, or the ability to encode additional proteins [5].

Typical LTR retrotransposons encode two genes: *gag* and *pol* [6]. The *gag* gene encodes the structural proteins, including capsid (CA) and nucleocapsid (NC), which assemble into virus-like particles (VLPs). The *pol* gene encodes the proteins that provide enzymes involved in reverse transcription and integration into the host genome: aspartic proteinase (AP), RT, RNAseH (RH), and integrase (INT). Both genes can be encoded by the same or two ORFs. Based on the presence or absence of functional gag-pol protein-coding domains, LTR retrotransposons are classified as complete (autonomous) or incomplete (non-autonomous). Non-autonomous LTR retrotransposons must parasitize active ones to propagate [7].

Retroviruses also contain the *gag* and *pol* genes, but they additionally encode the envelope (ENV) protein and, in some cases, other proteins like TAT, REV, NEF, VPR, or VIF. Some of these are encoded in an antisense orientation relative to the *gag*-*pol*-*env* genes [8]. The roles of some of these additional proteins are known [9]: HBZ of the Human T-cell Lymphotropic Virus 1 (HTLV-1) exhibits nuclear localization and plays a critical role in the virus lifecycle and the pathogenic process, while the APH-2 from HTLV-2 also shows nuclear localization and interacts with TAX and CREB proteins [10]. Some LTR retrotransposon families also contain ORFs in the internal region encoding additional proteins (aORFs) [5]. The aORF can be found in either sense or antisense orientation with respect to the *gag*-*pol* genes and upstream or downstream of them [11]. In some cases, the presence of an aORF can be explained by a phenomenon similar to retrovirus gene transduction [12], but in these cases, the aORFs are present in only one or a few copies. In contrast, some families appear to contain aORFs or derivatives in all or most of their copies and in distant species [13]. Sense aORFs include those encoding ENV-like proteins, so named because they exhibit some structural and functional similarities with retroviral envelope (ENV) proteins [14–16]. Antisense aORFs between the *pol* gene and 3′ LTR have also been found in, for example, LTR-retrotransposon families such as maize Grande [13], rice RIRE2 [17], or Silene Retand [18]. The roles of the encoded proteins are not known, but in the case of Grande, the encoded protein shows nuclear localization [13].

The Retand lineage of plant LTR-retrotransposons belongs to the non-chromovirus gypsy elements and is abundant in some plant genomes, such as *Silene latifolia* (4,800 copies) [18] and maize (15,904 copies) [19]. No systematic studies have been conducted to determine the taxonomic distribution of the Retand elements. Here, I have taken advantage of the abundance of plant genome assembly sequences to determine the presence of PRAREs and to analyze their structures, paying special attention to the POL-3’LTR region.

## Methods

### Genomes used

A total of 617 *Viridiplantae* genome assemblies available in the NCBI database (https://www.ncbi.nlm.nih.gov) were selected, considering only one assembly per species and only genomes assembled at least at the chromosome level (Additional file 1).

### Retand LTR retrotransposon mining

tBLASTn was used to identify the Retand elements with the default parameters (except –e option set to 1e − 10), using the amino acid sequences of the RT central conserved domain of four characterized Retand elements as queries: Tat4-1 from Arabidopsis (AAD20101.1; 159 aa), Gret1 from vineyard (AB242301.1; 159 aa), Cinful-1 from maize (AF049110.1; 159 aa), and Grande1-4 from teosinte (X97604.1, 160 aa)(Additional file 2). Only hits with complete and uninterrupted RT domains (ranging from 157 to 162 aa) without stop codons or frameshifts were selected. Only RT domains encoded in chromosome assemblies, not in scaffolds, were chosen. For each genome assembly, the selected set of RT sequences was aligned using MAFFT (https://mafft.cbrc.jp/alignment/server/) with 62 model RT sequences (Additional file 2), including the four Retand sequences used as queries, 56 RT sequences from other lineages, and two RT sequences from *Caulimoviridae*. A phylogenetic tree was constructed using the NJ method and 100 bootstrap repetitions (https://mafft.cbrc.jp/alignment/server/). Only the RT sequences that clustered with the Retand RT sequences were used for further analyses.

To identify the complete LTR-retrotransposon sequences, the 10 kb up- and downstream sequences of each RT domain were selected and compared using BLAST2seq (http://kinase.com/blast/wblast2.cgi?0). Only elements with identical LTRs and identical insertion repeats were retained. The LTR sequences identified in each genome were grouped using CD-HIT with a sequence identity cut-off of 90% (https://mafft.cbrc.jp/alignment/server/spool/_ho.250120195231950bzjTZQOq87tUISl5scuRklsfnormal.html). Finally, the elements with significant insertions or deletions were removed (their LTR or internal sequences differed by more than 2% from the consensus size of the cluster).

### Sequence analyses

The ORFs were predicted using ORF Finder (https://www.ncbi.nlm.nih.gov/orffinder/). Protein domains were identified using MOTIF (https://www.genome.jp/tools/motif/). The presence of tandem repeats was determined using Tandem Repeats Finder (https://tandem.bu.edu/trf/trf.submit.options.html), and only tandem arrays containing at least three copies were considered. Sequence alignments were performed using the G-INS-I method of MAFFT (https://mafft.cbrc.jp/alignment/server/). Amino acid conservation was determined using Weblogo (https://weblogo.berkeley.edu/logo.cgi). Protein DNA-binding predictions were performed using hybridDBRpred (hybrid model for DNA-binding residue prediction)(http://biomine.cs.vcu.edu/servers/hybridDBRpred/).

## Results

### Species distribution

**Fig. 1:**
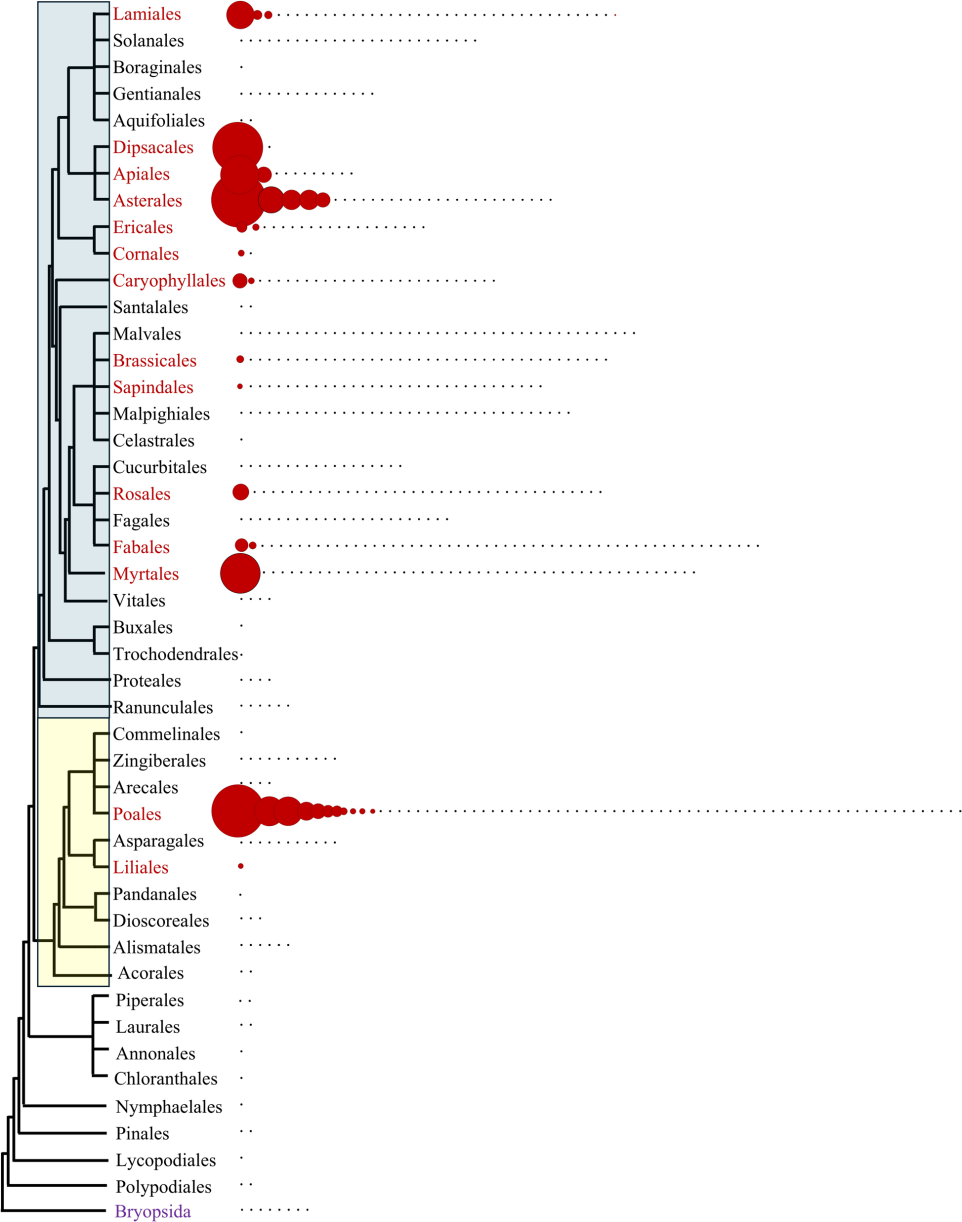
Taxonomic distribution of PRAREs. A black dot indicates an analyzed genome in which no element was detected, while a red circle represents a genome where PRAREs were identified. The diameter of the red circle correlates with the copy number. Orders in red are those in which at least one PRARE is present. The blue box indicates *Eudicotyledons*, and the yellow box represents *Liliaceae*. The phylogenetic tree is based on The Plant Tree of Life [20]

Once inserted into the genome, retrotransposons begin to suffer mutations, causing their sequences and structures to diverge from their original active form. Therefore, it is easier to determine LTR-retrotransposon structures in recent elements, meaning that they have identical LTRs and insertion repeats and do not contain significant insertions or deletions. I named them PRAREs (Potentially Recently Active Retand Elements). The presence of PRAREs was examined in 617 genome assemblies of *Viridiplantae* species (Additional file 1), and a total of 2,735 PRARE copies were identified, distributed across 122 clusters corresponding to 34 species, with copy numbers per cluster varying between 1 and 180 (Additional file 3). PRAREs were found in *Eudicotyledons* and *Liliopsida*, but not in other groups of plants (Fig. 1). Among the *Liliopsida*, PRAREs are especially abundant in *Poales*, although they are also found in *Liliales*. Among *Eudicotyledons*, the distribution is much more dispersed. They are abundant in *Gentianidae* (*Apiales*, *Asterales,* and *Dipsacales*), *Ericales*, and *Cornales*, and are also present in *Myrtales*, *Lamiales*, *Caryophyllales*, *Brassicales*, *Sapindales*, *Rosales*, and *Fabales*.

### Phylogenetic analysis

**Fig. 2:**
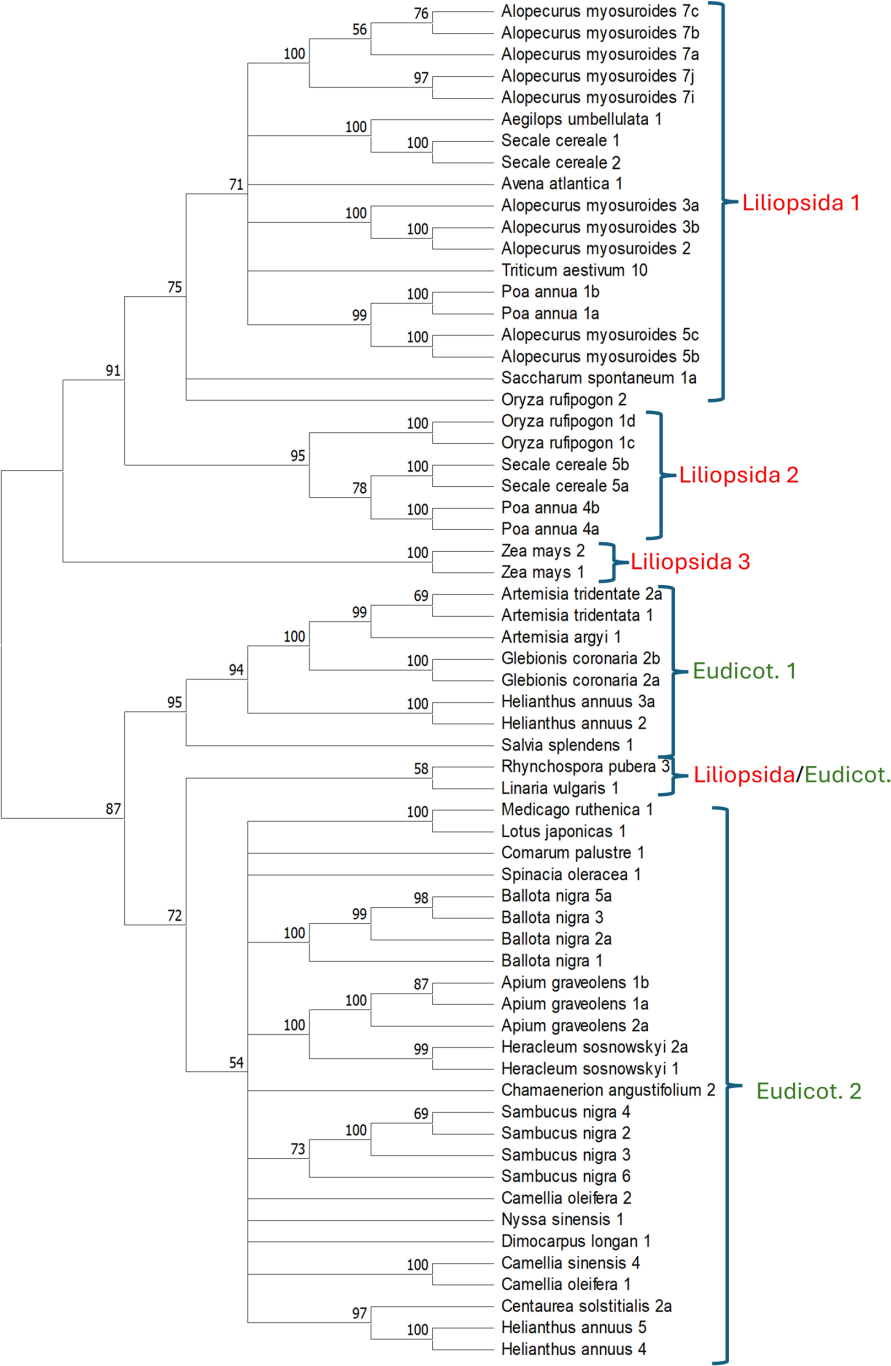
Phylogeny of PRAREs based on the RT domain. Midpoint-rooted ML phylogenetic tree of the consensus sequences of the RT domain of clusters containing at least 10 copies. Bootstrap values based on 1,000 replicates are shown

A consensus amino acid sequence of the RT domain was determined for each cluster containing at least ten copies. The RT sequences were aligned, and a ML phylogenetic tree was constructed (Fig. 2). Sequences are distributed among six clades, three containing sequences only from *Liliopsida*, two only from *Eudicotyledons*, and one from both. Clusters of the same species tend to group together in one or more groups corresponding to the same or different clades. For example, the four sunflower sequences are distributed in two groups, with two sequences each in different clades (Eudicot 1 and 2), whereas the ten *Alopecurus myosuroides* clusters are distributed in three groups, all within the same clade (Liliopsida 1).

The Liliopsida/Eudicot clade includes two sequences from *Rhynchospora pubera* (*Liliopsida*) and *Linaria vulgaris* (*Eudicotyledon*). The Liliopsida/Eudicot clade is closely related to the Eudicot-2 clade. Phylogenetic trees using other domains (GAG-AP, RH-INT, and TRP28) also show that Rhynchospora pubera 3 groups with *Eudicotyledons* and not with *Liliopsida* (Additional File 4). The location of Rhynchospora pubera 3 suggests that it arose from a horizontal transfer from an Eudicotyledonous plant.

### Structural variability and encoded conserved protein domains

The structural characteristics of the elements in the clusters were analyzed, including the sizes of the LTRs and internal regions, the presence of ORFs, and the existence of arrays of tandem repeats (Table 1). The sizes of the LTRs vary between 315 and 1,194 bp, with an average of 635 bp, while the sizes of the internal regions range from 7,380 to 12,869 bp, with an average of 10,364 bp.

**Table 1:** Structural characteristics of the selected Retand clusters

Cluster	Copy num.	LTR	Internal region	POL-3’LTR	GAG	AP	RH	INT	TRP28	Other domains	Tandem arrays
**Liliopsida 1**											
Alopecurus myosuroides 7c	26	477	9,671	5,427	NO	YES	YES	YES	YES	NO	0
Alopecurus myosuroides 7b	23	481	9,685	5,433	NO	YES	YES	YES	YES	NO	0
Alopecurus myosuroides 7a	13	479	9,680	5,428	NO	YES	YES	YES	YES	NO	0
Alopecurus myosuroides 7j	180	454	9,480	5,305	NO	YES	YES	YES	YES	NO	0
Alopecurus myosuroides 7i	52	454	9,581	5,279	NO	YES	YES	YES	YES	NO	0
Aegilops umbellulata 1	33	504	12,140	5,663	YES	YES	YES	YES	YES	NO	1
Secale cereale 1	10	506	12,869	6,091	YES	YES	YES	YES	YES	NO	1
Secale cereale 2	26	507	12,726	6,153	YES	YES	YES	YES	YES	NO	1
Avena atlantica 1	37	491	11,237	4,743	YES	YES	YES	YES	YES	DNA topo2	1
Alopecurus myosuroides 3a	46	576	10,798	3,906	YES	NO	YES	YES	YES	NO	0
Alopecurus myosuroides 3b	43	577	8,734	4,079	YES	YES	NO	NO	YES	NO	0
Alopecurus myosuroides 2	55	564	8,734	4,102	YES	YES	NO	NO	YES	NO	0
Triticum aestivum 10	10	528	11,996	5,828	YES	YES	YES	YES	YES	NO	1
Poa annua 1b	10	424	12,250	5.501	YES	YES	YES	YES	YES	NO	2
Poa annua 1a	15	439	12,029	5,301	YES	YES	YES	YES	YES	NO	2
Alopecurus myosuroides 5c	133	491	11,821	5,491	YES	YES	YES	YES	YES	NO	0
Alopecurus myosuroides 5b	15	493	11,834	5,474	YES	NO	YES	YES	YES	NO	0
Saccharum spontaneum 1a	10	1,065	12,304	5,854	YES	YES	YES	YES	YES	polIII	0
Oryza rufipogon 2	18	440	10,428	4,282	YES	YES	YES	YES	YES	NO	0
**Liliopsida 2**											
Oryza rufipogon 1d	35	1,077	10,727	4,484	YES	YES	YES2	YES	YES	Smc	0
Oryza rufipogon 1c	61	1,174	10,703	4,483	YES	YES	YES2	YES	YES	Smc	0
Secale cereale 5b	21	947	11,752	5,367	YES	YES	YES	YES	YES	Smc/polIII	0
Secale cereale 5a	65	954	11,775	5,359	YES	YES	YES	YES	YES	Smc/polIII	2
Poa annua 4b	21	1,031	10,962	4,981	YES	YES	YES	YES	YES	Smc/polIII	0
Poa annua 4a	24	1,079	10,861	4,914	YES	YES	YES	YES	YES	NO	0
**Liliopsida 3**											
Zea mays 2	12	585	8,245	4,200	NO	YES	YES	YES	YES	NO	0
Zea mays 1	21	587	8,365	4,313	NO	NO	YES	YES	YES	NO	1
**Eudicot 1**											
Artemisia tridentata 2a	63	488	9,588	3,868	YES	YES	YES	YES	YES	NO	0
Artemisia tridentata 1	42	488	9,581	3,899	YES	YES	YES	YES	YES	NO	0
Artemisia argyi 1	81	496	9,362	3,665	YES	YES	YES	YES	YES	NO	0
Glebionis coronaria 2b	13	571	9,588	4,035	YES	NO	YES	YES	YES	NO	0
Glebionis coronaria 2a	41	581	9,843	4,108	YES	NO	YES	YES	YES	NO	0
Helianthus annuus 3a	23	880	10,274	4,549	YES	YES	YES	YES	YES	NO	1
Helianthus annuus 2	15	806	11,456	5,899	YES	YES	YES	YES	YES	TolA	0
Salvia splendens 1	23	597	9,719	4,234	YES	YES	YES	YES	YES	NO	1
**Eudicot 2**											
Medicago ruthenica 1	19	315	7,380	3,888	NO	NO	YES	YES	YES	NO	0
Lotus japonicus 1	47	378	8,780	3,281	YES	YES	YES	YES	YES	NO	0
Comarum palustre 1	63	483	9,994	4,340	YES	YES	YES	YES	YES	NO	3
Spinacia oleracea 1	54	425	10719	4,850	YES	YES	YES	YES	YES	NO	1
Ballota nigra 5a	28	775	10,294	4,118	YES	YES	YES	YES	YES	NO	0
Ballota nigra 3	28	685	10,371	4,252	YES	YES	YES	YES	YES	NO	0
Ballota nigra 2a	19	737	10,519	4,282	YES	YES	YES	YES	YES	Smc	0
Ballota nigra 1	12	696	9,557	3,609	YES	YES	YES	YES	YES	Recom.Inh.	1
Apium graveolens 1b	24	569	9,357	3,801	YES	YES	YES	YES	YES	NO	2
Apium graveolens 1a	41	548	9,753	4,121	YES	YES	YES	YES	YES	NO	2
Apium graveolens 2a	10	570	9,626	3,997	YES	YES	YES	YES	YES	NO	3
Heracleum sosnowskyi 2a	16	504	10,937	3,492	YES	YES	YES	YES	YES	NO	1
Heracleum sosnowskyi 1	38	504	9198	3,459	YES	YES	YES	YES	YES	NO	2
Chamaenerion angustifolium 2	171	664	10,051	4,522	YES	YES	YES	YES	YES	NO	1
Sambucus nigra 4	76	586	10,964	4,895	YES	YES	YES	YES	YES^a^	NO	1
Sambucus nigra 2	29	586	10,938	4,954	YES	YES	YES	YES	YES^a^	NO	1
Sambucus nigra 3	68	586	10,943	4,953	YES	YES	YES	YES	YES^a^	NO	1
Sambucus nigra 6	39	857	10,861	4,771	YES	YES	YES	YES	YES	NO	1
Camellia oleifera 2	13	551	10,377	4,812	YES	YES	YES	YES	YES	Smc	2
Nyssa sinensis 1	15	627	10,468	4,582	YES	YES	YES	YES	YES	NO	2
Dimocarpus longan 1	10	713	9,540	3,682	YES	YES	YES	YES	YES	Smc	0
Camellia sinensis 4	18	824	10,483	4,472	YES	YES	YES	YES	YES^a^	NO	1
Camellia oleifera 1	23	584	9,983	4,290	YES	NO	YES	YES	YES^a^	NO	1
Centaurea solstitialis 2a	58	900	8,485	2,933	YES	YES	YES	YES	YES	NO	1
Helianthus annuus 5	121	719	9,313	3,343	YES	YES	YES	YES	NO	NO	2
Helianthus annuus 4	71	720	9,310	3,327	YES	YES	YES	YES	NO	NO	2
**Liliopsida/Eudicot**											
Rhynchospora pubera 3	12	432	11,818	5,552	YES	YES	YES	YES	YES	NO	3
Linaria vulgaris 1	22	1,194	12,114	6,556	YES	YES	YES	YES	YES	NO	0

The grouping of the clusters is based on Fig 2. The sizes in base pairs (bp) correspond to the average values of the elements in each cluster. TRP28 YES^a^ indicates that the domain is present, but in an ORF shorter than 600 nt; DNA topo 2 = DNA topoisomerase 2 (PLN03237, PLN03237); Recom.Inh. = recombination and DNA strand exchange inhibitor protein (PRK00409, PRK00409); Smc = COG1106, smc, chromosome segregation ATPase (COG1196); TolA = cell envelope integrity inner membrane protein TolA (PRK09510); PolIII = DNA polymerase III subunits gamma and tau (PRK07764, PRK07764). Tandem arrays refer to the number of tandem arrays detected containing three or more repeats

**Fig. 3:**
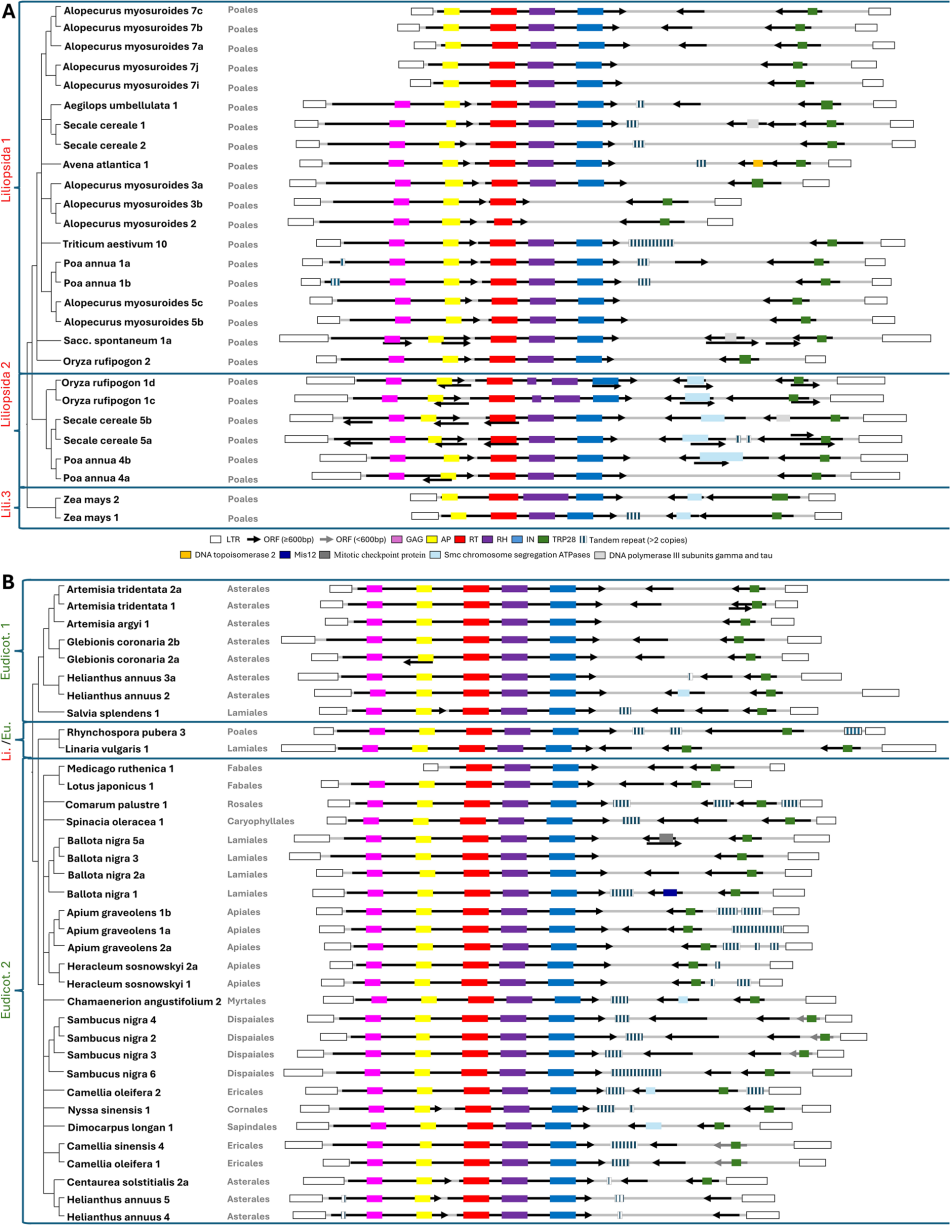
Structural organization of PRAREs. A schematic representation shows the structure and major features of the representative sequences of PRARE clusters that contain at least 10 elements. The representative sequence was chosen as one of the sequences in the cluster that exhibited the same structural characteristics as most of the elements within it (see Additional file 5 for more details). Clusters are grouped according to the phylogenetic analysis in Fig. 2. The tree on the left is based on Fig. 2. The shown structures include: LTRs (empty boxes), ORFs longer than 600 bp (black arrows), ORFs shorter than 600 bp but encoding a TRP28 domain (grey arrows), regions encoding known protein domains (colored boxes), and arrays of tandem repeats (boxes with vertical lines). Protein domains: Pink, GAG; Yellow, Aspartic proteinase; Red, Reverse transcriptase; Violet, RNAseH; Blue, Integrase; Green, TRP28, transposase 28 (PF04195); Orange, DNA topoisomerase 2 (PLN03237); Dark blue, Mis12 protein (PF05859); Dark grey, Mitotic checkpoint protein (PF05557); Light blue, Smc chromosome segregation ATPases (OG1196); DNA topoisomerase 2 (PLN03237, PLN03237); Light grey, DNA polymerase III subunits gamma and tau (PRK07764, PRK07764). **A** Liliopsida. **B** Eudicotyledons and Eudicotyledons/Liliopsida

For each element, the presence in the internal region of conserved ORFs (≥ 600 bp) and the presence of arrays of tandem repeats (≥ 3 repeats) were examined (Additional file 5). A representative element of each cluster was selected, meaning that it possesses the same structural characteristics as most of the elements in the cluster (Additional File 5). DNA sequences of all the elements are available in Additional file 6. All the representative elements contain at least two ORFs: at least one sense ORF (sense with respect to the RT coding region) and at least one antisense ORF (Fig. 3). The *gag* and *pol* genes are encoded by a unique ORF in 37 of the clusters (59%) and by two in the remainder, with 29% considering only *Liliaceae* compared to 83% in *Eudicotyledons*.

Ten of the 63 clusters are defective in at least one of the typical retrotransposon protein domains. Seven clusters do not encode the GAG domain, two do not encode both the GAG and the AP domains, and one cluster does not encode the RH and INT domains. Most of them (9) correspond to *Poales* (Table 1).

The size of the region between the *pol* gene and the 3’LTR varies between 2,933 and 6,566 bp, with an average of 4,616 bp. All clusters contain at least one ORF longer than 600 bp in this region, most of which are antisense with respect to the *gag* and *pol* genes (Fig. 3). None of the sense ORFs in this region encode known protein domains or putative transmembrane regions, as would be expected in the case of ENV proteins.

Of the 104 antisense ORFs detected, 72 encode known protein domains. 57 of the antisense ORFs encode a protein with the Transposase 28 domain (TRP28, PF04195). When also considering ORFs shorter than 600 bp, all clusters except two encode a protein with a TRP28 domain, all in the antisense direction relative to the *gag* and *pol* genes. The only two exceptions are Helianthus_annus_4 and 5. The role of the TRP28 domain is unknown, and it appears to be present only in proteins of flowering plants (InterPro database).

**Fig. 4:**
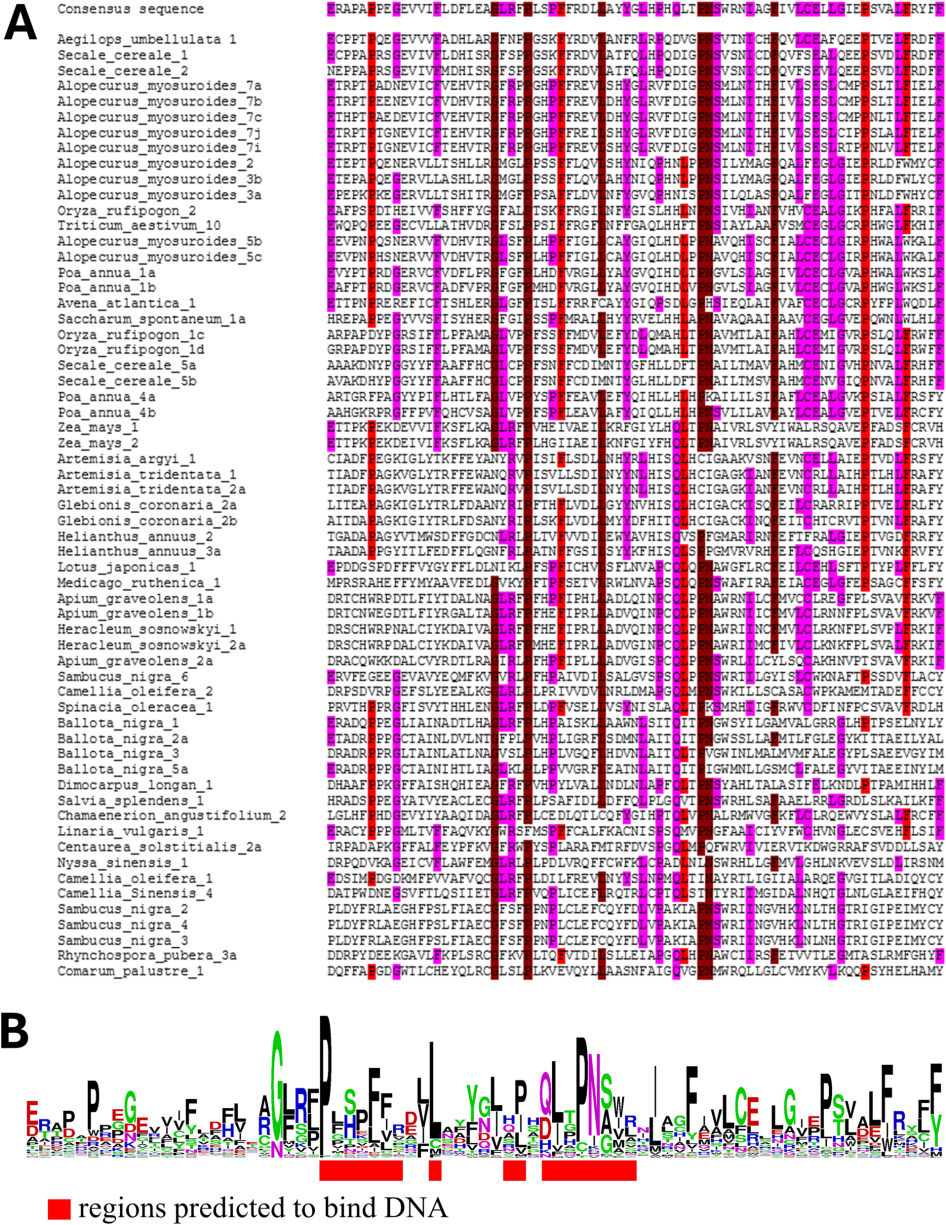
Sequence conservation of the TRP28 domain encoded by PRAREs. **A** Multiple sequence alignment of the consensus sequences of the TRP28 domains. The alignment is colored according to: dark red > 45%; red > 35%; pink > 25%. **B** Sequence logo of the TRP28 domain encoded by PRAREs. The logo is based on the aligned amino acid sequences. Amino acids are represented in a single-letter code, and the height of each letter depicts the frequency of the corresponding residues at that position, with the most frequent at the top. The red boxes represent the predicted DNA-binding residues according to hybridDBRpred

The cluster consensus sequences of the TRP28 domain were aligned (Fig. 4A) and showed several well-conserved positions (G21, P25, L34, P46, N47, and F55). A prediction program indicates that this region has DNA-binding capacity. The regions with a higher probability of binding DNA coincide with some of the most conserved amino acids (Fig. 4B). This information suggests that the function of TRP28 is related to DNA binding.

Five additional known protein domains are encoded by antisense ORFs located in the POL-3'LTR region (Fig. 3): 11 Smc domains, 2 mitotic checkpoint proteins, 2 DNA polymerase III subunits gamma and tau, one DNA topoisomerase 2, and one Mis12.

The analysis of the internal regions of the PRAREs also revealed the presence of arrays of tandemly repeated sequences in 32 of them (Fig. 3). The localization of the tandem arrays varies, but the most frequent is in the POL-3’LTR region, adjacent to the *pol* gene or next to the 3’LTR. They are highly variable in sequence and copy number. The size of the repeated elements ranges from 8 to 100 bp, and the maximum number of repetitions is 85. The number of repetitions varies among copies of the same cluster.

## Discussion

A systematic survey of recently inserted Retand elements in plant genomes revealed their presence in *Eudicotyledons* and *Liliaceae* but absence in other plant groups. This data must be interpreted with caution since the number of genomes available for some taxa is very low. In some cases, the results differ among the genomes of closely related species. For example, of the ten species of the genus *Oryza* analyzed, only one (*Oryza rufipogon*) contains PRIMEs. Burst-like amplification appears to be a common process in plants and has been described in different species, including *Arabidopsis thaliana* [21] and *Oryza sativa* [22]. The phylogenetic analysis shows a potential case of horizontal transfer of a Retand element. HTs of transposable elements between plants are known [23]. Although the phylogenetic distribution can be explained by alternative mechanisms to HTs [24], I believe that, in this case, they can be excluded because it involves taxonomically very divergent plant species (*Eudicotyledons* and *Liliaceae*).

The structural analysis of the PRIMEs showed that elements lacking some of the typical LTR-retrotransposon protein domains have been recently active. The amplification of defective LTR-retrotransposons has been described, and they are presumed to constitute non-autonomous elements whose transposition is dependent on complementation by other elements of the same or another related family [7, 25]. Transposition of non-autonomous endogenous retroviruses, dependent on a full-length copy, has also been described in endogenous retroviruses [26].

One of the characteristics of the Retand elements that has been confirmed here is the presence of a large internal region compared to other Gypsy lineages. As observed here, this large size is due to the region between the *pol* gene and the 3'LTR. This region contains ORFs, mainly antisense, some of which encode known protein domains, and frequently arrays of tandem repeats. Many active Retand elements carry tandem repeats in their 3' internal region between the gag-pol and the 3' LTR. The length of the repeat arrays varies, and the repeated sequences are family-specific. Similar structures have been observed in retrotransposons [27–30] and in retroviruses [31]. Their origin and eventual function remain unknown [32].

Plant retrotransposons capable of encoding additional proteins in antisense have been described [5, 11]. Here, I show that all PRIMEs contain antisense ORFs in the POL-3’LTR region, and that 97% of them include antisense ORFs encoding proteins with a TRP28 domain. Therefore, this structural element must be considered a characteristic of the Retand elements. Two fundamental questions arise: origin and function.

The origin of the POL-3'LTR region must be quite ancient since it is found in the major taxa of flowering plants. One possible origin is the transduction of cellular sequences. Transduction has been well documented for retroviruses [33] and has also been described for LTR-retrotransposons like maize Bs1 [34], various LTR retrotransposons in rice [35], sorghum [36], and in animals [12]. However, no sequence similarities have been found with TRP28 outside TEs, and the retrovirus-transduced genes are usually in the same sense as the retrotransposon *gag*-*pol* genes. Another possible origin is the insertion of another TE. Nested insertions of TEs are frequent in plant genomes [37]; however, the lack of sequence similarities with other known TEs does not support this hypothesis. Another possible explanation is that they originate from recombination with the genetic material of an unknown virus, which would explain the lack of similarities with available sequences.

Regardless of its origin, the fact that it is present in almost all recently active Retand elements suggests that it must serve some function. Some retroviral genomes encode antisense genes (HTLV-1,2,3 and STLV-2, 3, 4) [9, 38]. Some of these proteins are capable of binding nucleic acids and are involved in, for example, the regulation of transcription (TAT) or the transport of viral RNAs (REV). The TRP28 domain contains regions predicted to bind DNA, and the other protein domains encoded by antisense ORFs have functional categories involved in interactions with DNA. All this data suggest that TRP28 and other additional proteins encoded in the Retand elements may be involved in transcriptional regulatory roles. Further analyses must confirm this hypothesis.

## Conclusions

The Retand lineage of plant LTR-retrotransposons is present in *Eudicotyledons* and *Liliopsida*, but not in other groups of plants. They are especially abundant in *Poales* and *Gentianidae*. The horizontal transmission of Retand elements between species appears to be possible. Some of these elements are non-autonomous. Retand elements contain a large region between the *pol* gene and the 3’LTR. This region usually includes arrays of short tandem repeats and antisense ORFs that, in many cases, encode proteins with DNA-binding capabilities. Comparison with retroviruses suggests that they may play regulatory roles.

## Supplementary Information

Additional file 1. Species and genome assembly accession numbers used. Taxonomic information is retrieved from The Plant Tree of Life.

Additional file 2. Model RT sequences.

Additional file 3. Recently inserted Retand elements found.

Additional file 4. Three phylogenies of PRAREs based on the GAG-AP, RH-INT, and TRP28 domains. Midpoint-rooted ML phylogenetic tree of the consensus sequences of the indicated domains of clusters containing at least 10 copies. Bootstrap values are based on 1,000 replicates.

Additional file 5. Schematic representation of the ORFs and tandem repeats found in the PRARE elements. At the top, the consensus structure of the cluster. The meanings of the boxes and colors are as in Fig. 3. The number in red corresponds to the element chosen as representative of the cluster. Only clusters with ten or more elements are included.

Additional file 6. DNA sequences of the PRARE elements identified in this study. LTRs are in red. Flanking sequences are also included.

## Data Availability

Data is provided within the manuscript or supplementary information files.
