# Supplementary material for: Retand LTR-retrotransposons in plants: a long way from pol to 3’LTR
Source: Mob DNA. 2025 Apr 2;16:15. doi: 10.1186/s13100-025-00354-z (PMC11963269; doi:10.1186/s13100-025-00354-z)
Supplement: Supplementary file 2 — Additional file 2. Model RT sequences. [file 13100_2025_354_MOESM2_ESM.docx]

>Gypsy_Non-chromo_Retand-1; Tat4-1;

VKKKNDKWRVCIDFTDLNKACPKDSFPLPHIDRMVEATTGNELLSFMDAFSGYNQIPMHKDDQEKTSFIIDRGTYCYKVMPFGLKNVGARYQRLVNQMFAPQLGKTMEVYIDDMLVKSTRSADHIDHLKACFETLNKYNMKLNPAKCLFGVTSGEFLGY

>Gypsy_Non-chromo_Retand-2; Gret1;

VPKKEGKWRVCVDYTNLNNACPKDSFPLPRIDQIVDSTSGQGMLSFLDAFSGYHQIPMSPDDEEKIAFITPHDLYCYKVMPFGLKNAGATYQRLMTKIFKPLIGHSVEVYIDDIVVKSKTREQHILHLQEVFYLLRRYGMKLNPSKCAFGVSARKFLGF

>Gypsy_Non-chromo_Retand-3; Cinful-1;

VKKANGKWRMCIDFTDLNKACPKDEFPLPRIDSLVDATASSELMSLLDCYSGYHQIWMKREDEPKTSFITPSGTYCYLRMPEGLKNAGGSFSRMTAKVLQSQIGRNVLTYVDDIIVKSTKQENHIADLQETFASFRQAGLKLNPEKCVFGVKKGKFLGC

>Gypsy_Non-chromo_Retand-4; Grande1-4;

VRKKTGQWRMCVDYTDLNKSCPKKDPFGLPRIDQVVDSTAGCELLSFLDCYSGYHQIRLKESDCLKTSFITPFGAYCYVTMPFGLKNAGATYQRMIQRCFSTQIGRNVEAYVDDVVVKTKQKDDLISDLEETFASIRAFRMKLNPEKCTFGVPSGKLLGV

>Gypst_Chromov-Tekay-1; Peabody;

VKKKEGTMRLCVDYRQLNKVTIKNRYPLPRIDDLMDQLVGASVFSKIDLRSGYHQIRVKTEDIQKTAFRTRYGHYEYSVMPSGVTNAPGVFMEYMNRIFHPYLDKFVVVFIDDILVYSKSEEEHVEHLRVVLGVLREKKLFAKLSKCEFWLEEVSFLGH

>Gypst_Chromov-Tekay-2; Tekay;

VSKKDGSRRMCVDYRSLNEVTIKNKYPLPRIEDLFDRMKGAKIVSKIDLRLGYHQLKIRAEDVPKIAFTTRYGLYEFLVMSFGLTNAPAYFMNLMNKAFMEYLDQFVVVFIDDILIYSLNEEAHEDHLRLVLQKLRDNQLYAKFSKCDFWLKEVAFLRT

>Gypst_Chromov-Tekay-3; Xilon-Diguus;

VEKKDGTKRMCIDYRALNEVTIKNKYPLPRIEDLFDQLRGASVFSKIDLRSGYHQLRIRPSDIPKTAFITKYGLYEFTVMSFGLTNAPAFFMNLMNSVFMDYLDKFVVVFIDDILVYSQSEEEHADHLRMVLQRLREHQLYAKLSKCEFWISEVLFLGH

>Gypst_Chromov-Tekay-4; Gyma;

VKKKDQTLRMCVDYRPLNEVTIKNKYPLPRIDILFDQLTGARVFSKIDLRSGYHQIRIRPEDIPKTAFTTRYGLFEYLVMSFGLTNAPAHFTYLMNSVFMPELDKFVVVFIDDILIYSKNEEEHAQHLRIVLTRLREHQLYAKFSKCAFWLEEIQFLGH

>Gypsy­_Chromov-Chlamvir-1; Vcarteri199-102;

VGKKDGSLRMCIDYRGLNSMTTKNRYPLPRVDDLLDKLRGSAYFSSIDLQQGYNQIRIAESDIPKTAFRTPFGHFEYTVLSFGLVNAPATFQAVIDRMFRPYIDKFVVCYLDDILVYSKTKEEHLQHLRLVLDVLRREQLYAKQSKCHWAKSEVEYLGH

>Gypsy­_Chromov-Chlamvir-2; Vcarteri199-4__Chromov-Chlamvir;

EKANGQGLRMVLDYRALNKLTIKRRYPMPNITELFDQLQGANLFSSLDLQQGYNQIRISKEDIEKTAFIALGLGQYQFKVLCFGLTNAPATFQAVMNRIFAAYIGKFVLVYLDDILVFSKTPAEHEQHIRTVLSLLREHQFKAKMSKCQFNQTELHFLGV

>Gypsy­_Chromov-Chlamvir-3; REM1__Chromov-Chlamvir;

VEKADGSLRMVLDYRALNKITRKRRYPMPNITELFDQLAGAKVFSSLDLQQGYNQIRIHPDDVPKTGFIAPGMGQFEYKVLCFGLTNAPATFQSVMNNMFGPHIGKFVLVYLDDILVFSKNAEEHKEHLRTVLEILRKNQFKAKRSKCDFNRPELHFLGH

>Gypsy­_Chromov-Chlamvir-4; Ty3-RT__REXdb_ID9791;

VGKKDGSLRMCIDYRGLNSMTTKNQYPLPRVDDLLDKLRGSAYFSSIDLQQGYNQIRIAESDIPKTAFRTPFGHFEYTVLSFGLVNAPATFQAVIDRMFRPYIDKFVVCYLDDILVYSKTKEEHLQHLRLVLDVLRREQLYAKQSKCHWAKSEVEYLGH

>Gypsy_Chromov-Reina-1; Magellan;

VKKKDQTYRFCVDYRHLNALTIKTKFPVPIIDEFLDELCGAAWFSTLDLRAGFHQIRMSPKDQHKTAFQTHHGHFEFRVMPFGLSGAPATFQGAMNTTLAPLLRKCVLVFFDDILVYSHTWEDHLSHLQQVLTLLAKDQWYIKMSKCAFAKKQIAYLGH

>Gypsy_Chromov-Reina-2; Boshoe;

VKKPDGSWRFCVDYRALNALTIKDAFPIPVVEELLDELHGAKFFTKLDLRSGYHQVRMRPEDVHKTAFRTHDGLYEFLVMAFGLCNAPATFQALMNDVLRPFLRRFVLVFFDDILIYSSTWADHLRHLRAVLDELQRHQLFIKRSKCSFGAPSVAYLGH

>Gypsy_Chromov-Reina-3; Apil;

VKKSDGTWRFCVDYRELNGKTVKDRYPIPVVDELLDELHGSIFFTKLDLRSGYHQVRMHPEDVDKTAFQTHRGHFEFLVMPFGLTNAPSTFQSLMNDILNPFIRKFVLVFFDDILIYSKTWAAHLQQVKQVFQTLRAHKLALKRSKCFFGVTNVSYLGH

>Gypsy_Chromov-Reina-4; Gloin;

VKKKDGSWRFCVDYRALNRVTVLDKFPIPMIDELLDELHGTTIFSKLDLCLGYHQIRMREDDIEKTAFRTHDGHFEFLVMPFGLTNAPASFQSLMNELFGPFLGKFVLVFFDDILIYSNNLTNHVKHLTLVMEVLAKHQLFANRKKCLLRQSQIDYLGH

>Gypsy_Chromov-TCN1-1; Ppatens251-111;

VKKKDGSLRMCVDYRGLNKITIKNRYPLPLISGLLDQLNQAKIYTKIDLRGAYNLVRIKEGDEWKTAFRTRYGHFEYNVMPFGLTNAPAAFQHLMNDVFREFLDNFVVCYLDDILIYSKNVEEHEIHVRQVLQKLRDAGLYAKMEKCVFHTTQVDFLGY

>Gypsy_Chromov-TCN1-2; Ty3-RT__REXdb_ID9841;

VKKKDGSLRMCVDYRGLNKITIKNRYPLPLISGLLDQLNQAKIYTKIDLRGAYNLVRIKEGDEWKTAFRTRYGHFEYNVMPFGLTNAPTAFQHLMNDVFREFLDNFVVCYLDDILIYSKNVKEHEIHVRQVLQKLRDAGLYAKMEKCVFHTTQVDFLGY

>Gypsy_Chromov-TCN1-3; Ty3-RT__REXdb_ID10309;

VKKKDGSLRMCVDYRGLNKITIKNRYPLPLISGLLDQLNQAKIYTKIDLRGAYNLVRIKEGDEWKTAFRTRYGHFEYNVMPFGLTNAPAAFQHLMNDVFREFLDNFVVCYLDDILIYSKNVEEHEIHVRQVLQKLRDAGLYAKMEKCVFHTTQVEFLGY

>Gypsy_Chromov-TCN1-4; Ty3-RT__REXdb_ID10885

VKKKDGSXQMCVDYRGLNKITIKNRYPLSLISGLLDHLNQAKIYTKIDLRRAYNLVCIKEGDEWKTAFRTKYGHFEYNVMPFGLTNAPVAFQHLMNDVFREFLDDFVVCYLDDILIYSKKPKEHELHVRHVLQKLRDARLYAKIEKFVFHTTQVNFLGY

>Gypsy_Chromov-Galadriel-1; Galadriel__Chromov-Galadriel;

QKKQDGTMRMCVDYRALNKATIKNKYSVPLVQDLMDRLSKACWFTKLDLRAGYWQVRIAEGDEPKTTCVTRYGSYEFLVMPFGLTNAPATFCNLMNNVLFDYLDDFVVVYLDDIVIYSRTLEEHVNHLSLVLSQLRKYTLYVKMEKCEFAQQEIKFLGH

>Gypsy_Chromov-Galadriel-2; Monkey__Chromov-Galadriel;

QKKQDGSLRLCVDYRALNKVTVKNKYPIPLIADLFDQLGKAKYFSKLDLRSGYWQVCIAEGDEAKITCVTRYGAFEFLVMPFGLTNAPATFCTLMNQLFKEYLDKFVVVYLDDIVVYSQTLEEHVKHLWMIFKVLRENTLFVKREKCYFAQTEILFLGH

>Gypsy_Chromov-Galadriel-3; PtGaladriel;

QKKADGSLRMCVDYRALNKVTIKNKYPVPLVQDLMDRLSGASVFTKLDLRSGYWQVRIAEGDEHKTTCVTRYGSYEFLVMPFGLTNAPATFCNLMNDVLYEFLDDFVVVYLDDIVVFSRSMNEHVVHLSRVLSRLREHKLFVKREKCDFASAEIMFLGH

>Gypsy_Chromov-Galadriel-4; Ty3-RT__REXdb_ID12313

QKKADGSLRMCVDYRALNKVTIKNKYPVPLIQDLMDRLCGASIFTKLDLRSGYWQVRVADGDEHKTTCVTRYGSYEFLVMSFGLTNAPATFCNLMNDVLYDFLDNFVVVYLDDIVIYSRGIEEHVTHLSKVLGRLREHELYVKKEKCEFAKSEIMFLGH

>Gypsy_Chromov-CRM-1; CRM;

VPKKDGTWRMCVDCRAINNITIRYRHPIPRLDDMLDELSGAIVFSKVDLRSGYHQIRMKLGDEWKTAFKTKFGLYEWLVMPFGLTNAPSTFMRLMNEVLRAFIGKFVVVYFDDILIYSKSMDEHVDHMRAVFNALRDARLFGNLEKCTFCTDRVSFLGY

>Gypsy_Chromov-CRM-2; PetI1__Chromov-CRM;

VPKKDGSWRMCVDCRAVNKITVKYRHPIPRLDDMLDELSGSCVFSKIDLRSGYHRIRMKPGDEWKTAFKTKFGLYEWLVMPFGLSNAPSTFMRLMNHVLKPFINKFVVVYFYDILIYSKTIDEHIGHLSQVLEALRAEKLYANLKKCAFCVEKVVLLGF

>Gypsy_Chromov-CRM-3; CRA1__Chromov-CRM;

VPKKDGSWRMCVDCRAINNITVKYRHPIPRLDDMLDELHGSCVFSKIDLKSGYHQIRMKEGDEWKTAFKTKHGLYEWLVMPFGLTNAPSTFMRLMNHVLRAFIGVFVVVYFDDILIYSRSLDDHVEHLKTVLDVLRREQLFANFKKCTFCTDNLVFLGF

>Gypsy_Chromov-CRM-4; VitV6__Chromov-CRM;

TPKKYGSxCMCVDSRAINKITVKYHFPISRLNDMLDRLEGAVVFTKLDLRSGYRQIRIRQGDEWKTDFKTKDGLYEWLVMPFVLSNAPSTFMRLMNYILHSFIGKFVVVCFDDILIYSKDEEEHLSHLREVLLALQANKLYINLKKCSFMTSHLLFLGF

>Gypsy_Non-chromo_TatI-1; Smoellend91-784;

ARKANGKLRLCVDFRDLNKATAKDHFPTPFIDAIVDEMAGHWYYSFMDGFSGYNQIFIHPDHRDLMAFRTPWGVFAFNVLPFGLCNAGATFQRFMTHAFHDLIGGALRVYMDDLCTTSDSFEDHLMHLDQIFTRCRHHRFSLNPAKCKFGVTEGRLLGF

>Gypsy_Non-chromo_TatI-2; Ty3-RT__REXdb_ID6782;

ARKANGKLRLCIDFRDLNKATVKDHFPTPFIDAIVDEMAGHRYYSFMDGFSGYNQIFIHPDHRDLTAFRTPXGVFAFNVLPFGLCNAGATFQRFMTHAFHDLIGGALRVYMDDLCTTSDSFEDHLMHLEQIFTRCRHHRFSLNPAKCKFGVTEGRLLGF

>Gypsy_Non-chromo_TatI-3; Ty3-RT__REXdb_ID6783;

ARKANGKLHLCIDFRDLNKATAKDHFPTPFIDAIVDEMAGHRYYSFMDGFSGYNQIFIHPDLTAFQTPWGIFAFNVLPFGLCNAGATFQRFMTHTFHDLIGGALRVYMDDLCTTSDSFEDHLMHLEQIFTRCRHHRFSLNPAKCKFGVTEGRLLGF

>Gypsy_Non-chromo_TatI-4; Ty3-RT__REXdb_ID6784;

ARKANGKLRLCVDFRDLNKATAKDHFPTPFIDAIVDEMAGHWYYSFMDGFSGYNQIFIHPDHRDLMAFRTPWGVFAFNVLPFGLCNAGATFQRFMTHAFHDLIGGALRVYMDDLCTTSDSFEDHLMHLDQIFTRCRHHRFSLNPAKCKFGVTEGRLLGF

>Gypsy_Non-chromo_TatII-1; Ty3-RT__REXdb_ID6786;

VRKKSGEIRLCVDFRNLNRSSKKDNYPLPKMEHILQRVIGASRISMIDGFSGYNQISIMPEDREKTTFTTPWGTFMYAKMPFGLMNAGATFQRAMDITFIGEKDQFVVIYLDDITVFSRTNKEHRCHMKRVFLKCRRFGLSLNPKKSLFAMREGKLLGH

>Gypsy_Non-chromo_TatII-2; Ty3-RT__REXdb_ID6794;

VRKKNRKIQLCVDFRNLNKASDKDNYPVPSMEQILQQVSGSERLSLLDGFSGYNQVLMSPPDQLKTTFRTPWGTYAYCKMPFGLINADATFQRAMEISFRGLINHSVAIYLDDVTIYSKNKDDHLVHLRAVLLRCRKYGISLNPKKSIFAVEXGKLLGF

>Gypsy_Non-chromo_TatII-3; Ty3-RT__REXdb_ID6800;

VWKKSGEIRFCIDFXNLNRVSNKDNYPVPPMEQILQQVSGSKRLSLLDGFSGYNQVLMSPPNQLKTTFRTPWGTYAYRKMPFGLINAGATFQRVMDITFXGLINQSIIVYLYDVTVFSKNKNDHLAHLRVVLQXCRKYDISLNPKKSIFAVEXGKILGF

>Gypsy_Non-chromo_TatII-4; Ty3-RT__REXdb_ID6806;

ARKKNGEIRLCVDFRNLNKCSLKDNYPLPKMDHVLEKVVGANRMSMIDGFSGYNQITMNExDREKTAFTTPWGTFMYDKMPFGLMNAGATFQRAMDIDFVGERDKFVVIYLDDLTVFSKNDDEHLIHLKHTFEKCRRYGLSLNPKKSHFAMQEGKLLGH

>Gypsy_Non-chromo_TatIII-1; Pabies2;

IDKKGGSIRVCVDYRDINKACPKDNFPTPFVDQIVHDCTRSEIFSLMDGFSGYNQINIAPEDQHKMAFICPWGTFAYRKLPFGLKNAGATFQRAMSYAFHDIKHIVQPYLDDLPAHSLHRVDHPNHLRAIFVRCXFYRIHLNPHKCVFCVESARLLGF

>Gypsy_Non-chromo_TatIII-2; Ty3-RT__REXdb_ID6814;

IDKKQGMIRVCVDYRDINKACPKDNFLTPFVDQIVDDCARSEIFSLMDGFFGYNQINIVPEDQHKTAFICPWGTFPYRKLPFGLKNVGTTFQRAMSYAFHDIKHIVQPYLDDLPAHSMHRVDHPTHLRAIFICYRFYRIRLNPHKCVFYVESDRLLGF

>Gypsy_Non-chromo_TatIII-3; Ty3-RT__REXdb_ID6824;

IDKKGDTIHLCIDYRDINKACPKDNFPTPFIDQIVDDCVGSEIFSLMNGFSDYNQINIAPKDQHKTAFICPWGTFAYRKLPFGLKNACAMFQRAMSYAFHDIKHIIQPYLDDLPTHSMHRVDHPTHLXAIFVCCRFYRIRLNPHKCIFCVESGRLLGF

>Gypsy_Non-chromo_TatIII-4; Ty3-RT__REXdb_ID6835;

IDKKGGSIRVCVDYRDINKSCPKDNFPTPFVNQVVDDCADSEIFSLMDGFSGYNQINIAPEDQHKMDFICPWGTFAYKKLPFSLKNAGATFQRAMSYAFHNIKHIVQPYLDNLSAHSLRRVDHPNHLQAIFIRCWFYRIHLNPHKCVFCVEFGRLLGF

>Gypsy_Non-chromo_Ogre-1; Fvesca226-108;

VRKKNGKMRICVDYRDLNNATPKDIYPMPVADLLIDAAAGHEVLSFMDGTAGYHQILVAEEDRHKTAFRCPGFAGAFEYVVMPFGLKNAGATYQRAMNLIFHDILGKLIEVYIDDVVVKTKTRATHVADLRQVFTRMRRHNLKMNPAKCVFFAEAGDFLGF

>Gypsy_Non-chromo_Ogre-2; Mdomestica1;

VLKKNGALRICTDFRNLNLATPKDEYTMPISDLLIDAAANHAILSFMDGHAGYNQIFIAEADVHKTAFRCPGALGTYEWVVMPFGLKNAGATYQRAMNTIFHDLIGTIVEVYIDDVVVKSKRRQTHLDDLRQAFLRMRQHNLKMNPAKCAFGVSAGNFLGF

>Gypsy_Non-chromo_Ogre-3; Atlantys2;

VIKKNGKLRVCINFRDLNKAIPKDEYPMPVADQLVDAASGHKIISFMDGNAGYNQIFMAEEDIHKTAFRCPGAIGLYEWVVMTFGLRSAGATYQRAMNYIFHDLIGMLVEIYIddvvvkskevdeHIADLRQVLDRARKYGLKMNPTKCAFGVSAGQFLGF

>Gypsy_Non-chromo_Ogre-4; Ty3-RT__REXdb_ID6996;

VYKKNGKLRVCVDFRNLNQATPMDGYPMPTADVLIDAAAGHKIISFMDGNAGYNQILMAEEDIPKTAFRCPGHLGLFEWVVMTFGLKNAGATYQRAMNYIFHKLIGLLVEIYIDDVVVKSKSHEEHLADLRRVLECTKKHGLKMNPNKCAFGVSAGQFLGF

>Gypsy_Non-chromo_Phygy-1; >Ty3-RT__REXdb_ID5434;

VPKKNGSLRFIQDMQPVNAVTVRNAGIGPIVDEFAEAFAGRAIYSMGDLYSGYDQFQLAEGSRDMTTMRTPLGLVRMCTLPQGATNSVAHMMNGMNKVLRDFIPEKTMPFLDDVPIKGCREGDKDETLDTRGCRRYVVDHIQDCEKILTRLGEVHLTLSGMKSVFGVREVVIVGH

>Gypsy_Non-chromo_Phygy-2; >Ty3-RT__REXdb_ID5470;

VPKKNGSLRFIQDMQPVNALTVRNAGIGPIVDEFAEAFAGRAIYSMGDLYSGYDQFQLAEGSRDMTTMRTPLGLVRMCTLPQGATNSVAHMMNGMNKVLRDFISEKTMPFLDDVPIKGCREGDKDETLDTRGCRRYVVDHIQDCEKILTRLGEVHLTLSGMKSVFGVREVVIVGH

>Gypsy_Non-chromo_Phygy-3; >Ty3-RT__REXdb_ID5542;

IPKKNGSLRFIQDMQPVNALTVRNAGIGPIVDEFAEAFAGRVIYSMDDLYSGYDQFQLAEGSRDMTTMTTPLRLVRMCTLPQGATNSVAHMMNGMNKVLRDFIPEKTMPFLDDVPIKGCKEGDKDETLDTRGCRRYVVDHIQDCEKILTRLGEVHLTLSGMKSVFGVREVVIVGH

>Gypsy_Non-chromo_Phygy-4; >Ty3-RT__REXdb_ID5579;

VPKKNGSLRFIQDMQPVNAVTVKNVGIGPIVDEFAEVFARRAIYSMGDLYSGYDQFQLVEGSRDMTTMRTPLGLVRMCTLPQGATNSVAYMMNGMNKVLRDFIPKKTMPFLDDVPIKGCREGDKDETLDTRGCRRYVVDHIQDCEKILTRLGEVHLTLSGMKSVFGVREVIIVGN

>Gypsy_Non-chromo_Selgy-1; Smoellend91-110;

VTKPDGSIRFCIDYRPLNEVTIPDVYPLPHTTDLLDRLGRSRWFTKLDLASGYWQVPMAEEDIPKTAFRTPFGLYEFLVMPFGLNNAPATFQRMMDEVLCDMPEVTADFIDDVLIHSEGTWEGHVRDVCKVLDRLRDVGLKLKRSKCVFGSKDTTYLGY

>Gypsy_Non-chromo_Selgy-2; Smoellend91-571;

VTKPDGSIRFCIDYRPLNEVTIPDVYPLPHTADLLDRLGRSRWFTKLDLASGYWQVPMAEDDIPKTAFRTPFGLYEFLVMPFGLNNAPATFQRMMDEVLCDMPEVTADFIDDVLIHSEGTWEEHVRDVCKVLDRLRDVGLKLKRSKCVFGSKDTTYLGY

>Gypsy_Non-chromo_Selgy-3; Gmr1;

VPKPDGTHRFCNDFRRLNEVSDFDSYPMPRVDELIERLGPARYLSTLDLTKGYWQVPLAPSSREKTAFATPGGLFQYTVLPFGVHGAPATFQRMMDQVLRPHSSYAAAYIDDIIIHSASWDEHVKHVRAVLNGLRAAGLTANPAKCRLGREETAYLGY

>Gypsy_Non-chromo_Selgy-4; rGmr1;

VPKPDGTLRFCNDFRKLNEASSFDGYPMPRVDELLDRLGSARFISTLDLTKGYWQVPLAPGAKEKTAFTTPSGHWHYRVLPFGLHGAPATFQRMMDILLRPHQSYAAAYLDDVVVHSMCWEEHLTRLRRVLLELRRAGLTANPKKCHLGLAEAKYLGY

>Gypsy_Non-chromo_Athila-1; Athila4-1;

PTRTITGHRMCIDYRKLNAASRKDHFPLPFIDQMLERLANHPYYCFLDGYSGFFQIPIHPNDQEKTTFTCPYGTFAYKRMPFGLCNAPATFQRCMTSIFSDLIEEMVEVFMDDFSVYGPSFSSCLLNLGRVLTRCEETNLVLNWEKCHFMVKEGIVLDH

>Gypsy_Non-chromo_Athila-2; Diaspora;

PTRVQNNWRVCIHYRRLNQVTKKDHFPLPFIDQILECLAGKSHYCFLDGFSGYMQITIALEDQEKTTFTCLFGTFAYRRMSFGLCNAPGTFQRCMISIFSDFLENCIEEFMDDFTVYGSSFDGCLDSLEKVLNRRIETNLVLNFEKCHFMVEQGIVLGH

>Gypsy_Non-chromo_Athila-3;

PTRTITGHRMCIDYRKLNSASGKDHFPLPFIDQMLERLANHPYYCFLDGYSGFFQIPIHPNDQEKTTFTCPYGTFAYKRMPFGLCNAPATFQRCMTSIFSDLIEEMVEVFMDDFSVYGSSFSSCLSNLCRVLFRCEETNLVLNWEKCHFMVKEGIVLGH

>Gypsy_Non-chromo_Athila-4;

PTRTVTGHRMCIDYRKLNAATRKDHFPLPFIDQMLERLASHPYYCFLDGYSGFFQIPIHPDDQEKTTFTCPYGTFAYRRMSFGLCNAPATFQRCMMSIFTDMIEDFMEIFMDDFSVYGSSFKDCLDNLCKVLARCEEKHLVLNWEKCHFMVRDGIVLGH

>Caulimoviridae1; Petunia vein clearing virus;

SEQVRGKLRLVINYQPLNHFLQDDKFPIPNKLTLFSHLSKAKLFSKFDLKSGFWQLGIHPNERPKTGFCIPDRHFQWKVMPFGLKTAPSLFQKAMIKIFQPILFSALVYIDDILLFSETLEDHIKLLNQFISLVKKFGVMLSAKKMILAQNKIQFLGM

>Caulimoviridae2; SolLyc-03;

SEQKRGKSRIVIDYRNLNAKTKTFNYPIPNKILKIRQIQGYNYFSKFYCKSGFYLLKLEEESKQLTAFTVPQGFYEWNVLPFGYKNTPGRYQHFMDNYFNQLENCIVYIDDILLYSKTQDEHIRLLEKFIHIIKHLGISLSKKKAEIMKPQIEFLGI
