# Supplementary figures and images for: Retand LTR-retrotransposons in plants: a long way from pol to 3’LTR
Source: Mob DNA. 2025 Apr 2;16:15. doi: 10.1186/s13100-025-00354-z (PMC11963269; doi:10.1186/s13100-025-00354-z)

# GAG-AP

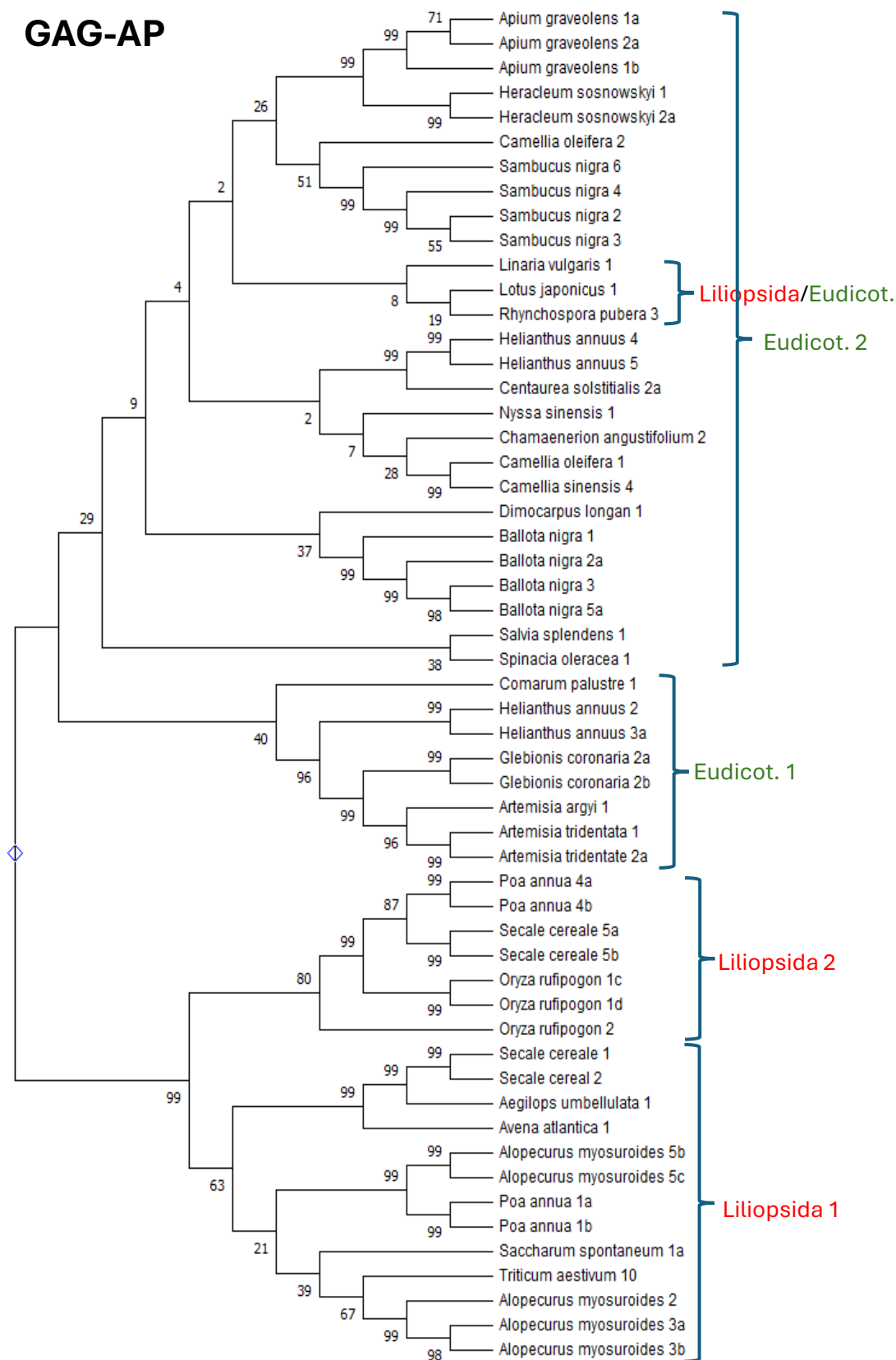

# RH-INT

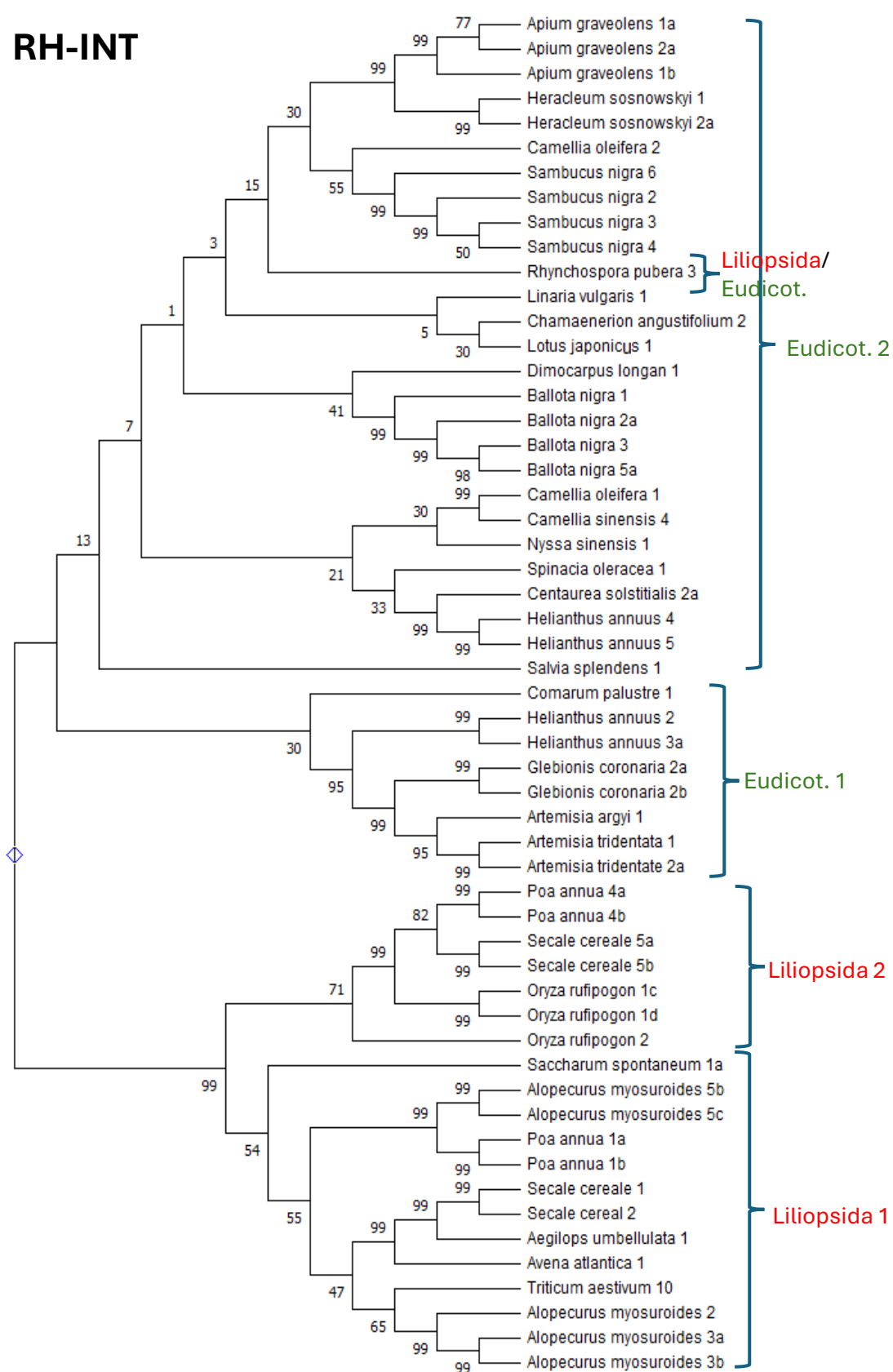

# TRP28

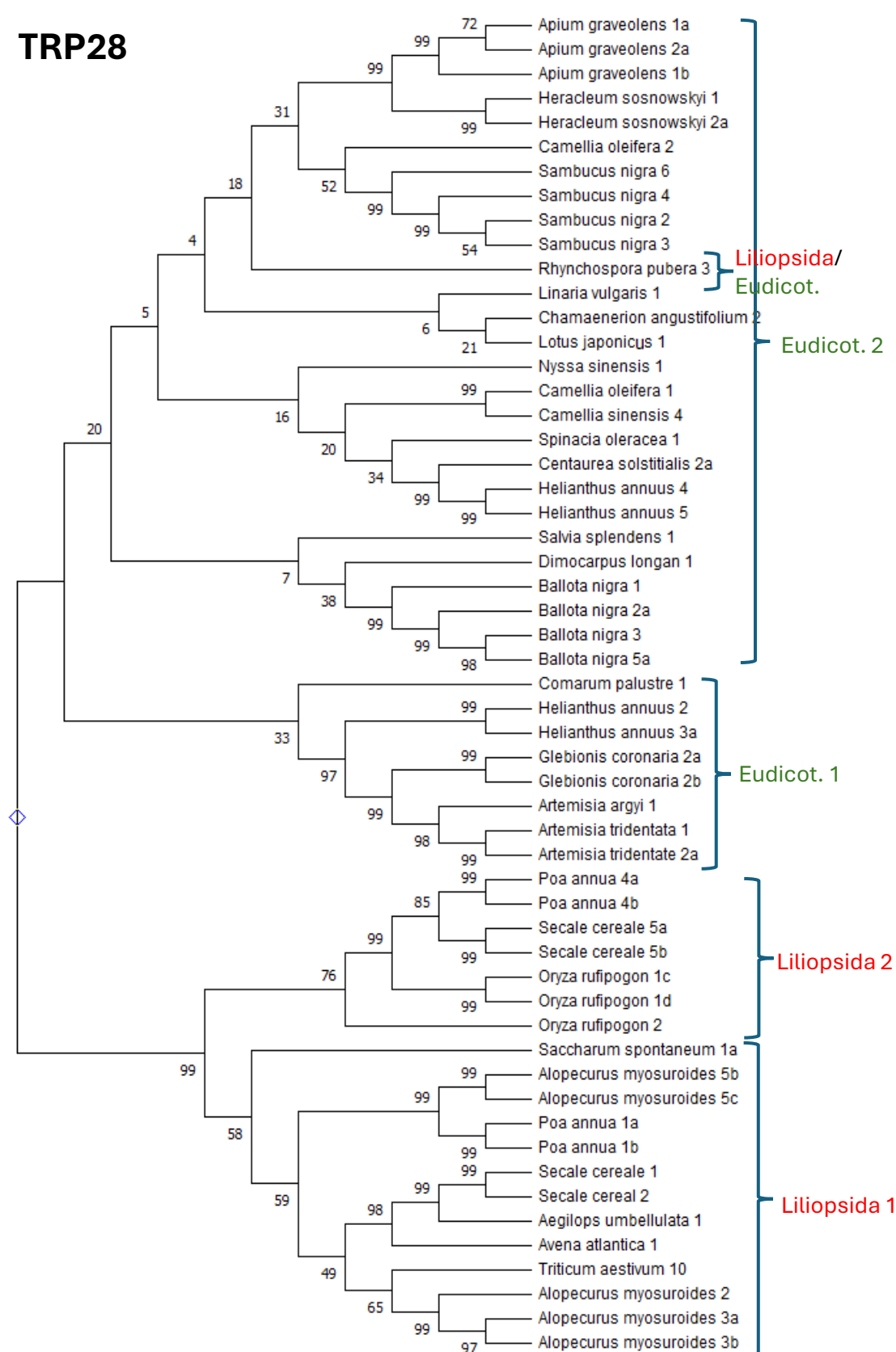

Supplement: Supplementary file 4 — Additional file 4. Three phylogenies of PRAREs based on the GAG-AP, RH-INT, and TRP28 domains. Midpoint-rooted ML phylogenetic tree of the consensus sequences of the indicated domains of clusters containing at least 10 copies. Bootstrap values are based on 1,000 replicates. [file 13100_2025_354_MOESM4_ESM.pdf]
