# Supplementary material for: Retand LTR-retrotransposons in plants: a long way from pol to 3’LTR
Source: Mob DNA. 2025 Apr 2;16:15. doi: 10.1186/s13100-025-00354-z (PMC11963269; doi:10.1186/s13100-025-00354-z)
Supplement: Supplementary file 5 — Additional file 5. Schematic representation of the ORFs and tandem repeats found in the PRARE elements. At the top, the consensus structure of the cluster. The meanings of the boxes and colors are as in Fig. 3. The number in red corresponds to the element chosen as representative of the cluster. Only clusters with ten or more elements are included. [file 13100_2025_354_MOESM5_ESM.pdf]

Aegilops umbellulata 1

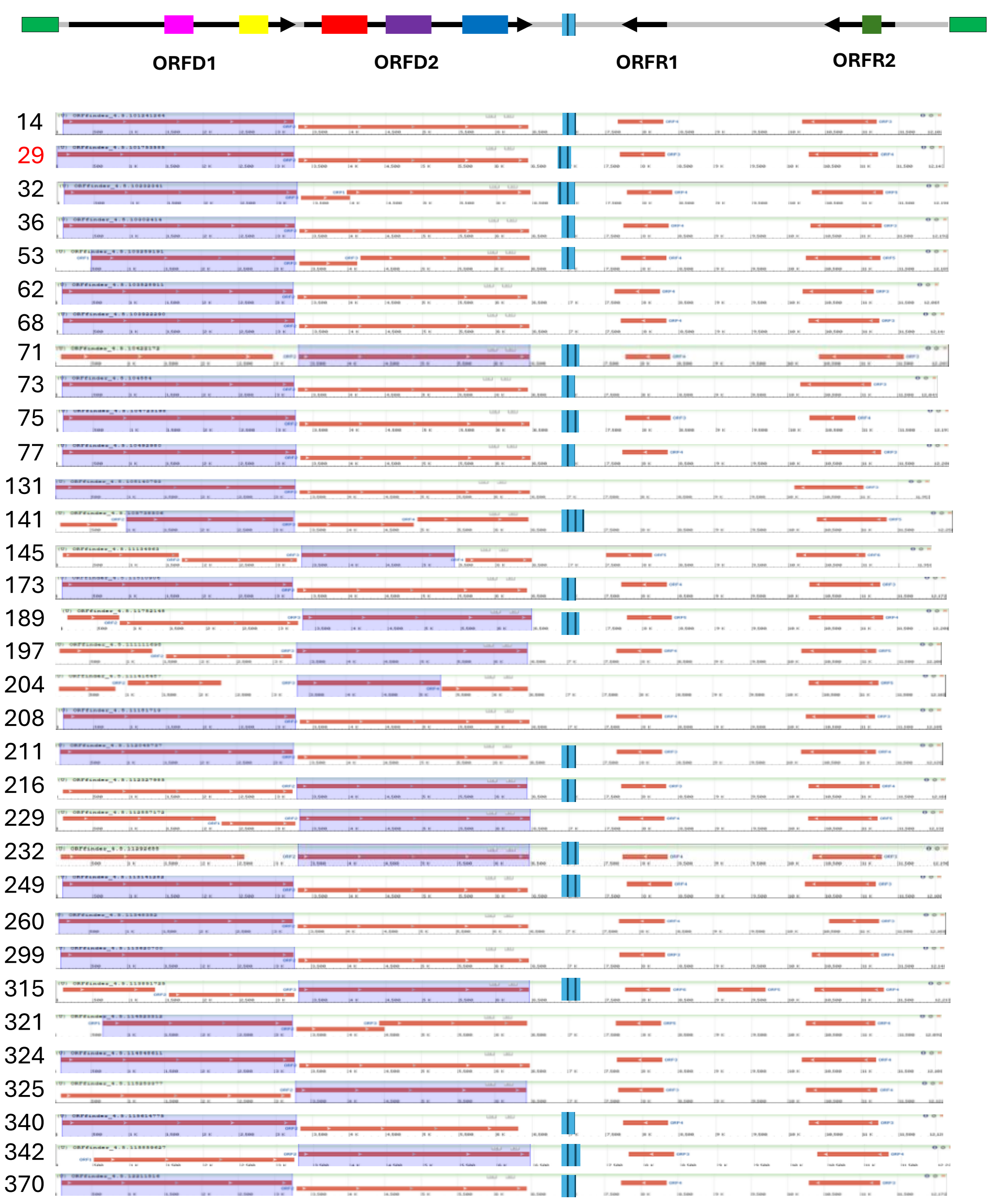

Alopecurus myosuroides 2

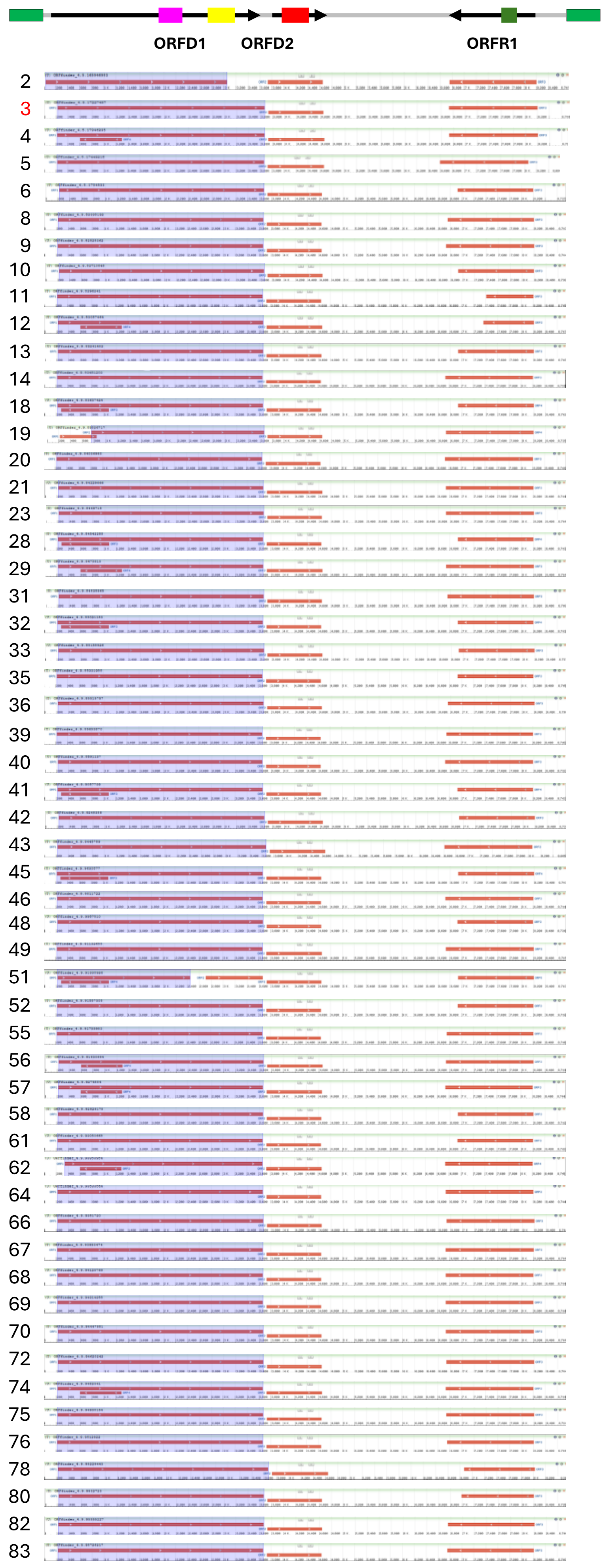

Alopecurus myosuroides 3a

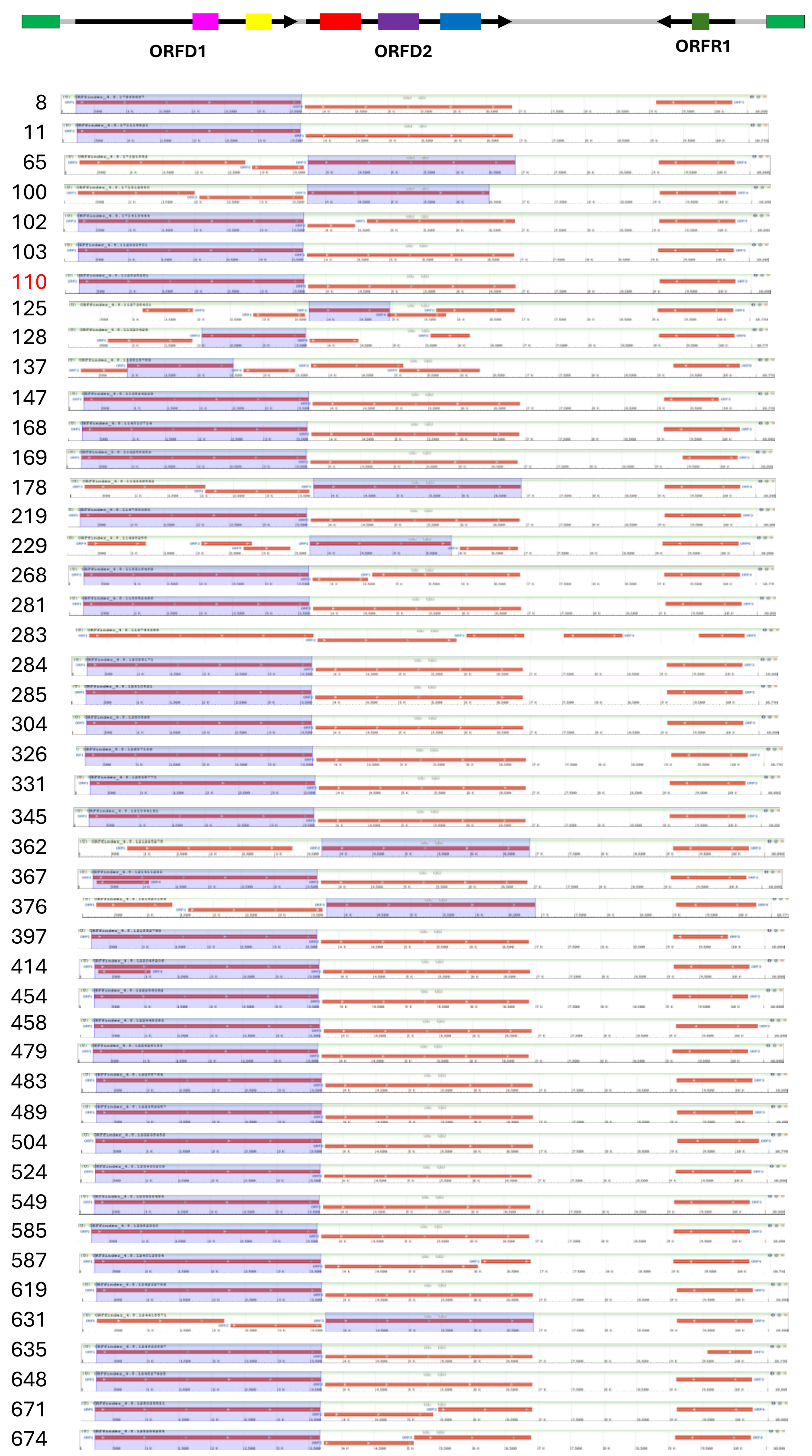

Alopecurus myosuroides 3b

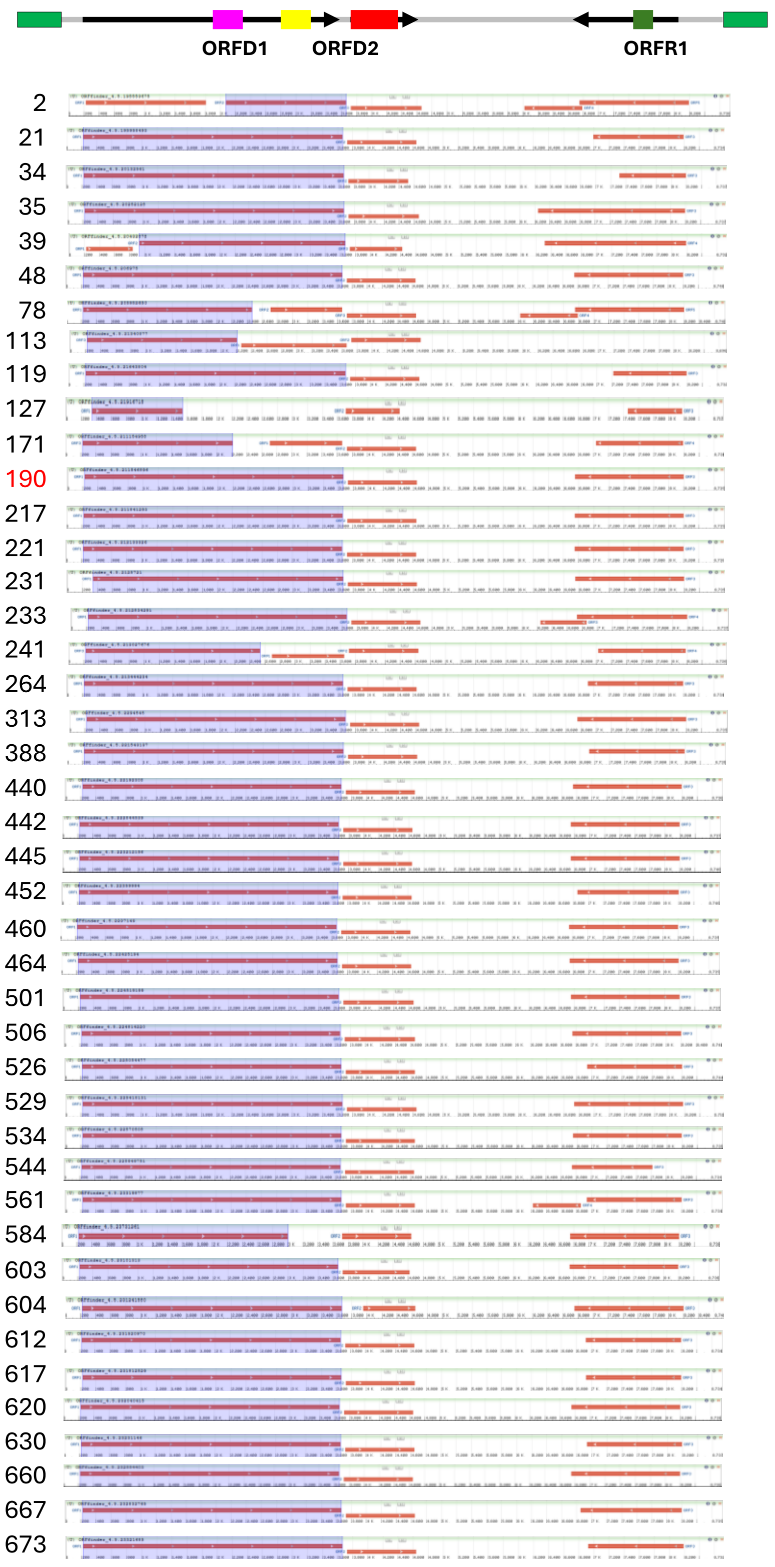

Alopecurus myosuroides 5b

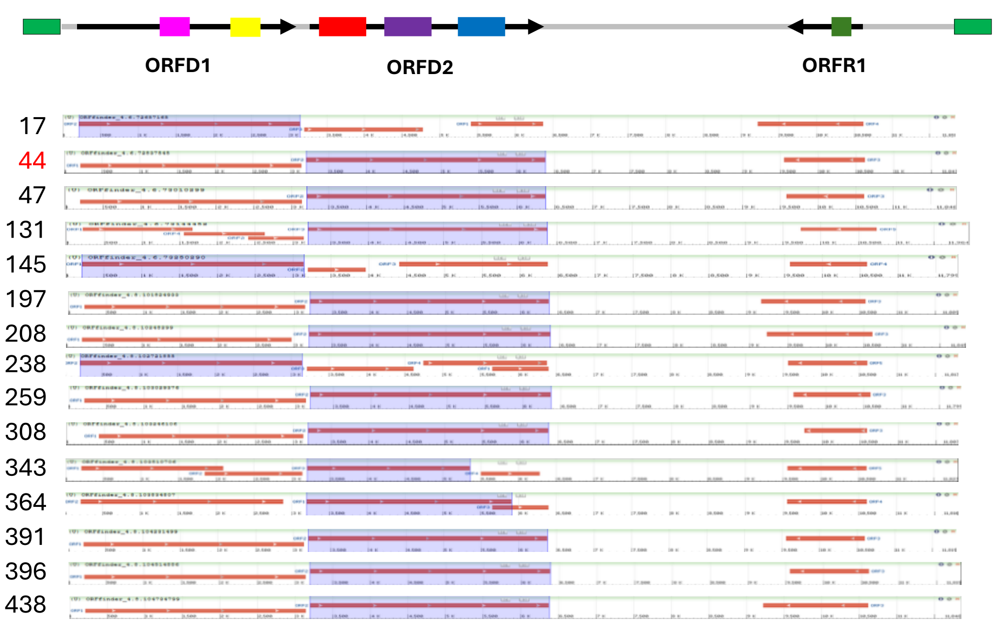

Alopecurus myosuroides 5c

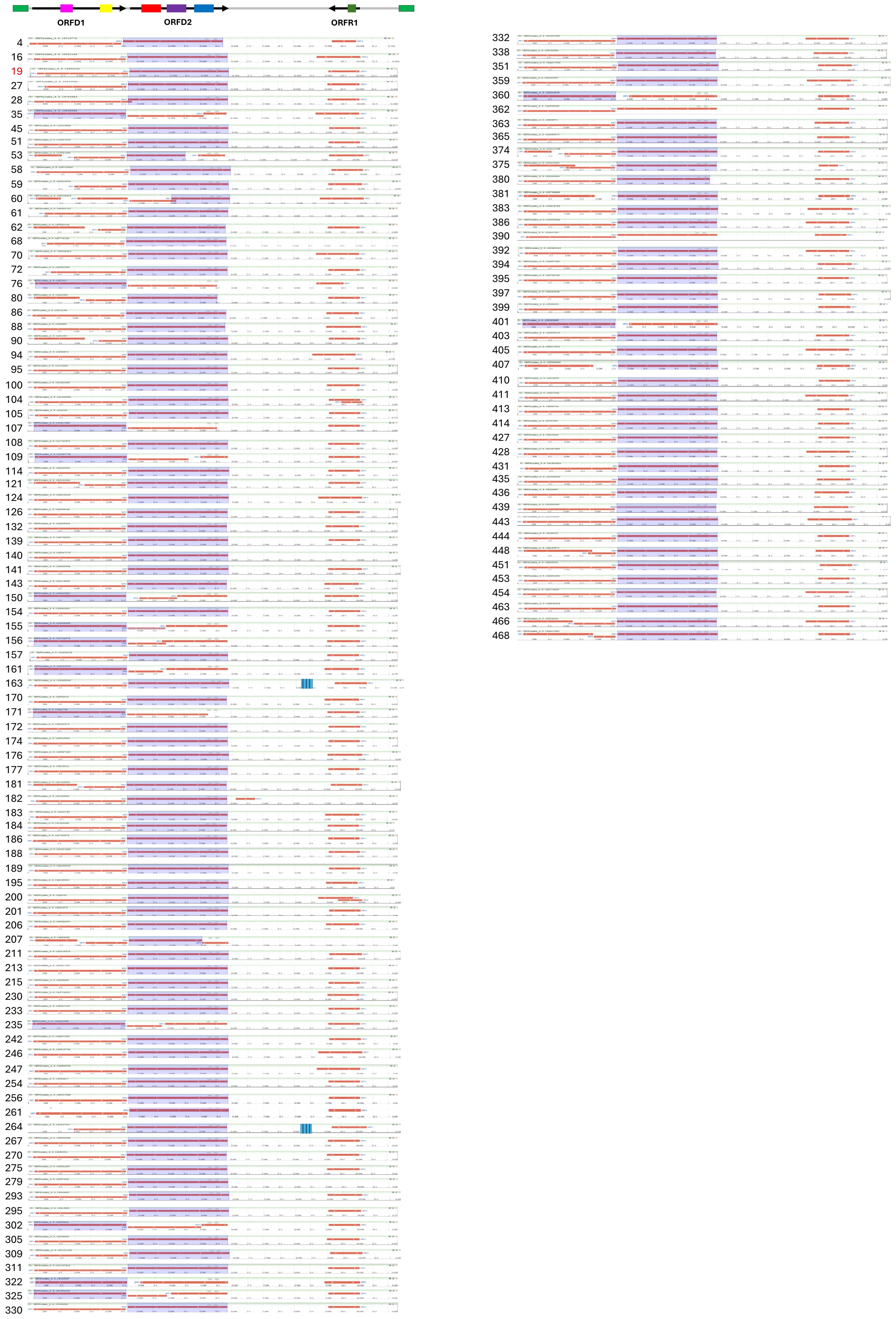

Alopecurus myosuroides 7a

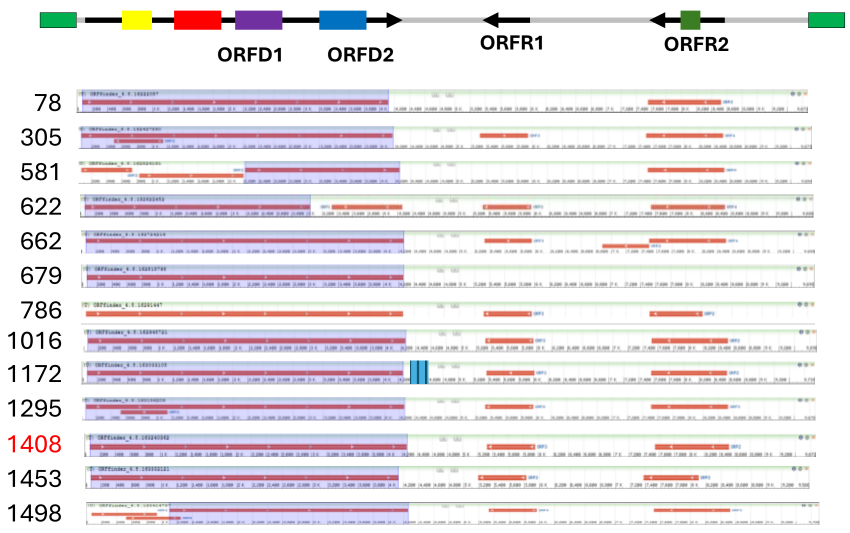

Alopecurus myosuroides 7b

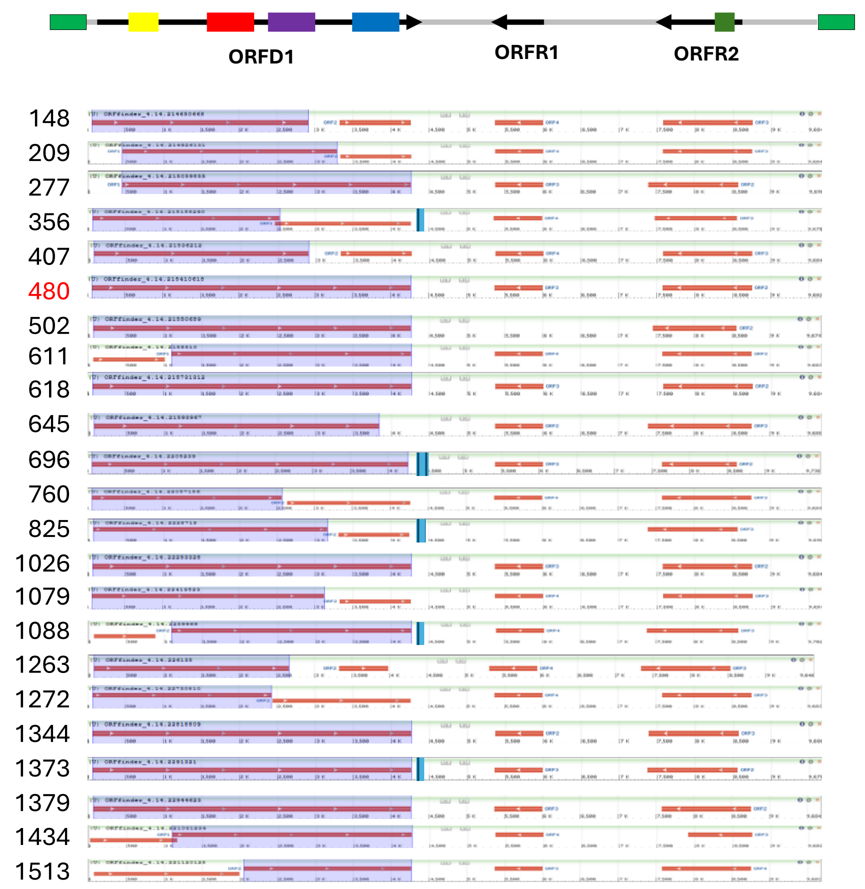

Alopecurus myosuroides 7c

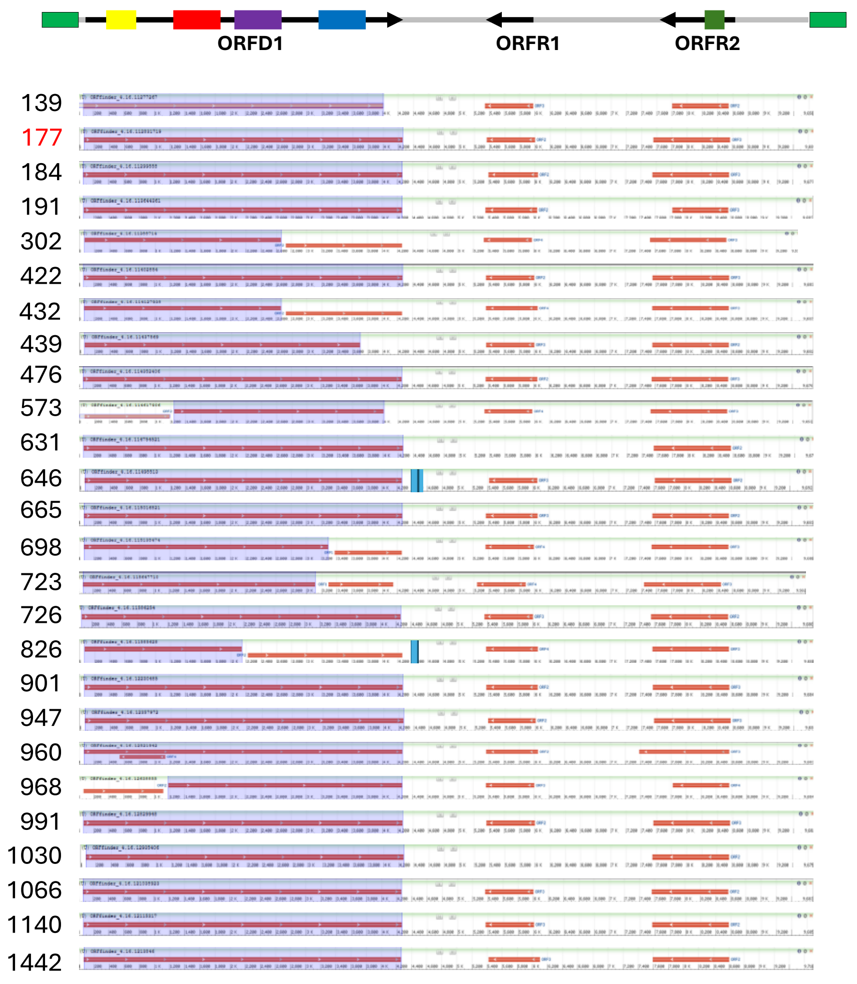

Alopecurus myosuroides 7i

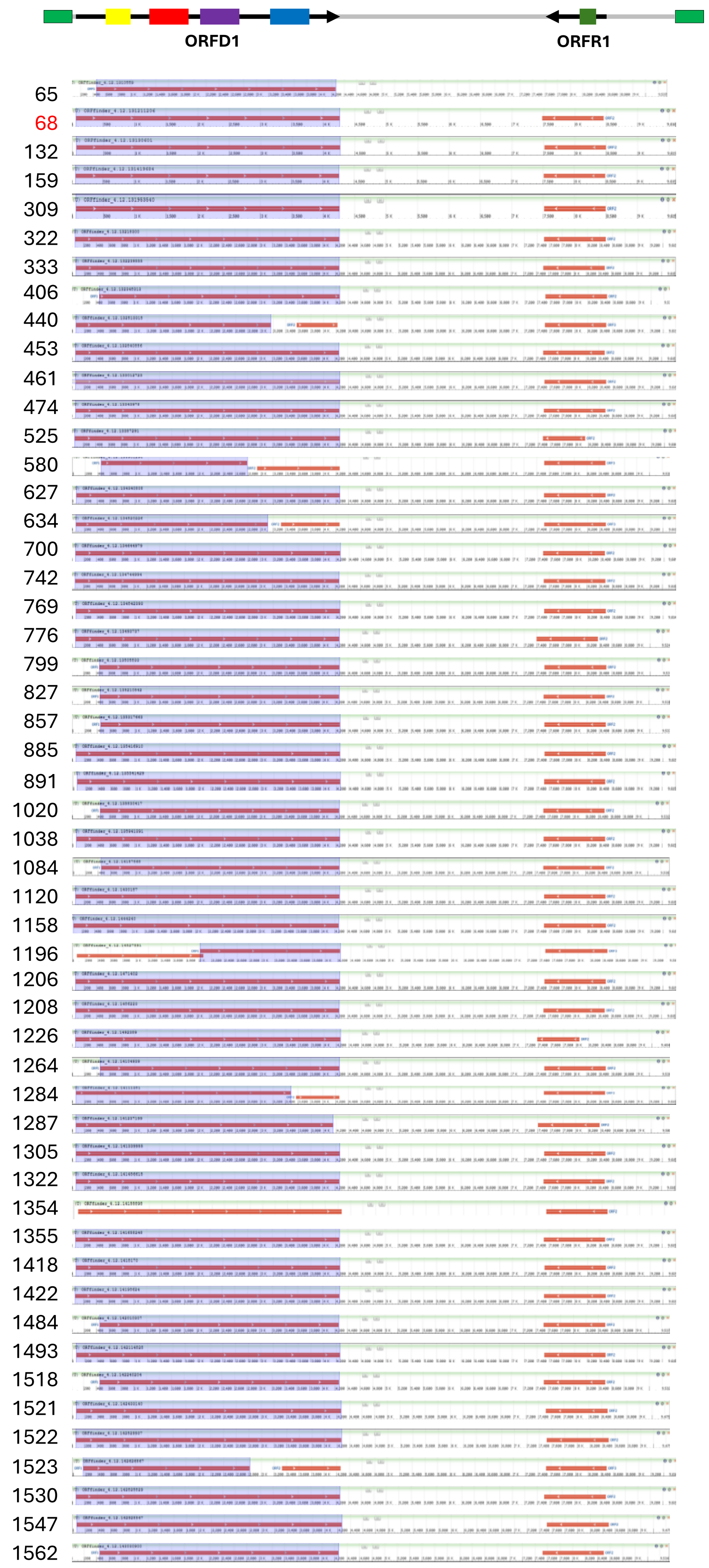

Alopecurus myosuroides 7j

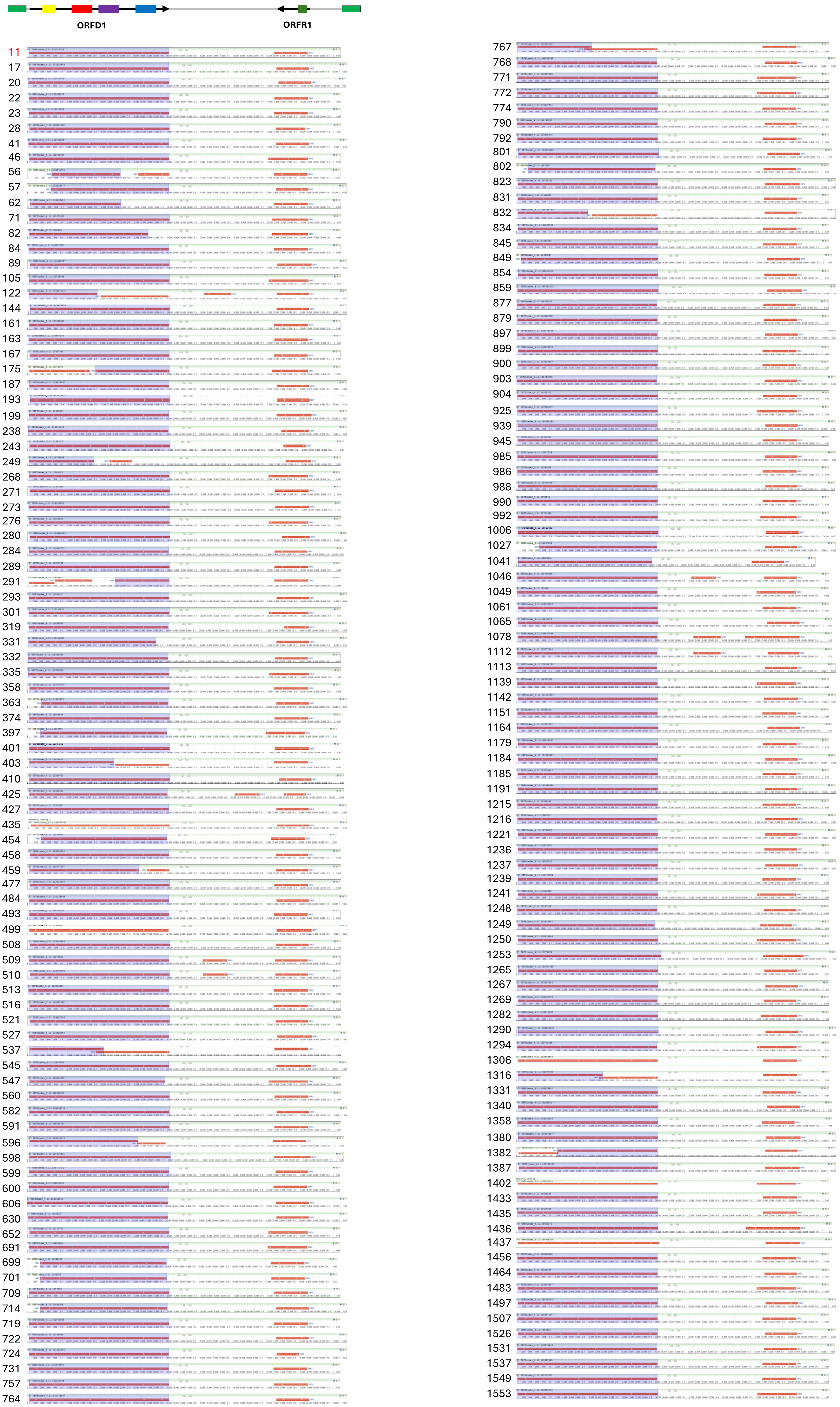

Apium graveolens 1a

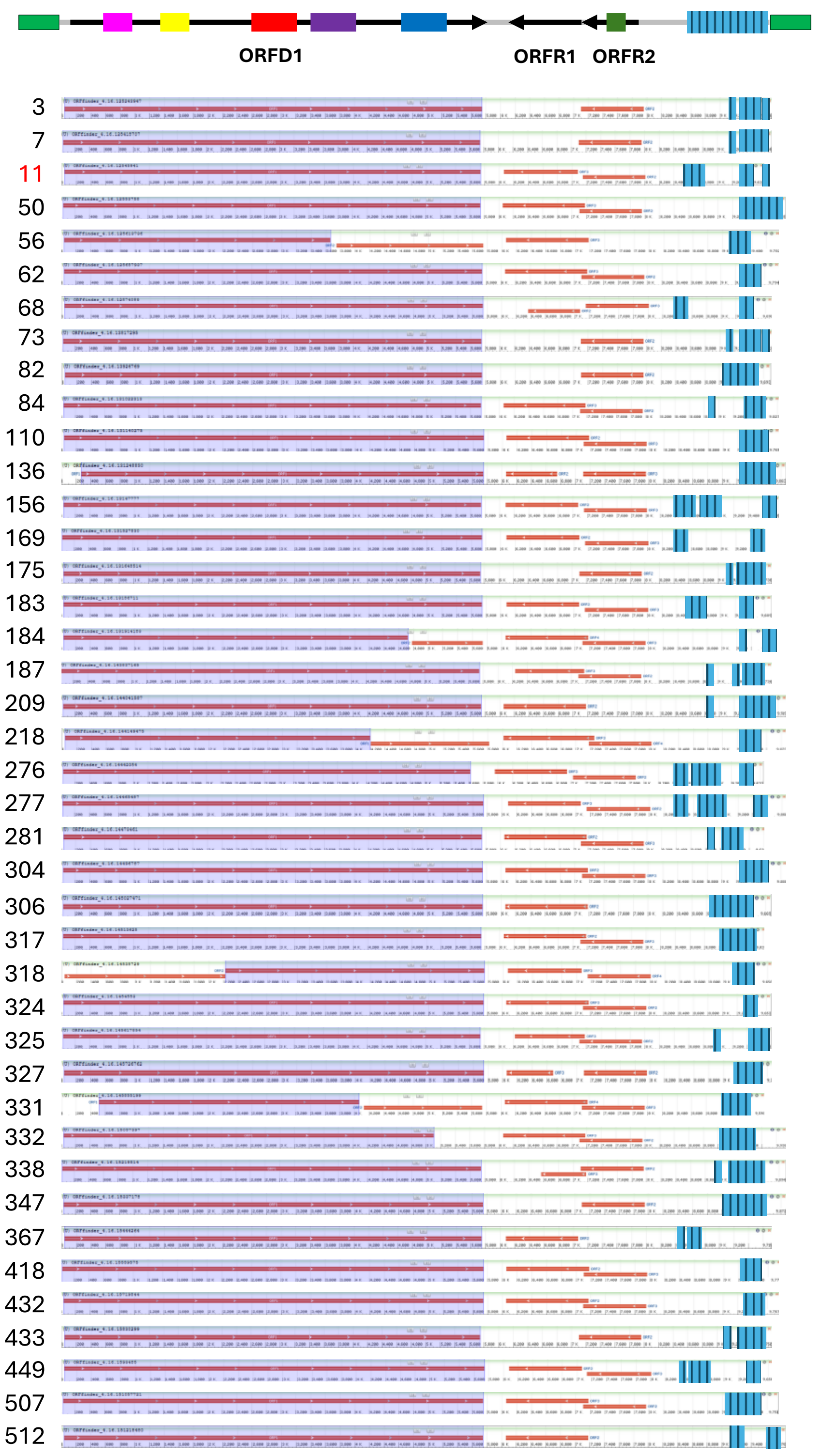

Apium graveolens 1b

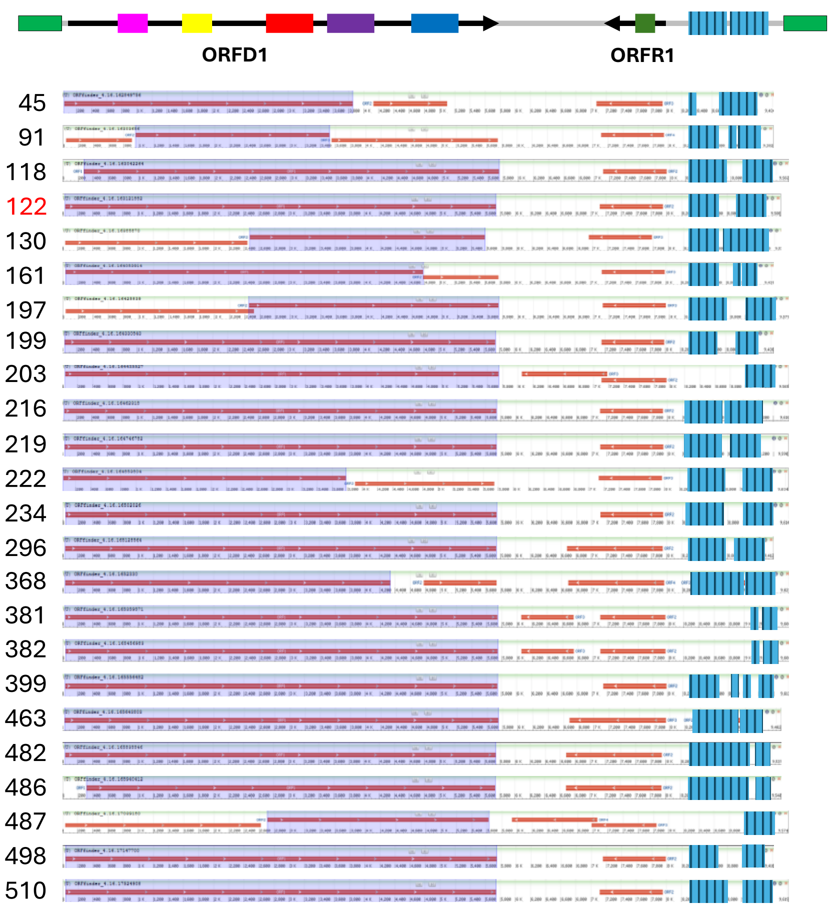

Apium graveolens 2a

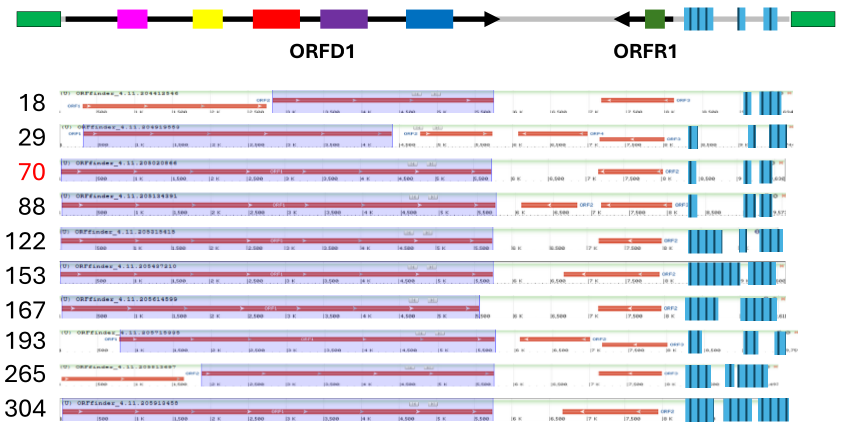

Artemisia argyi 1

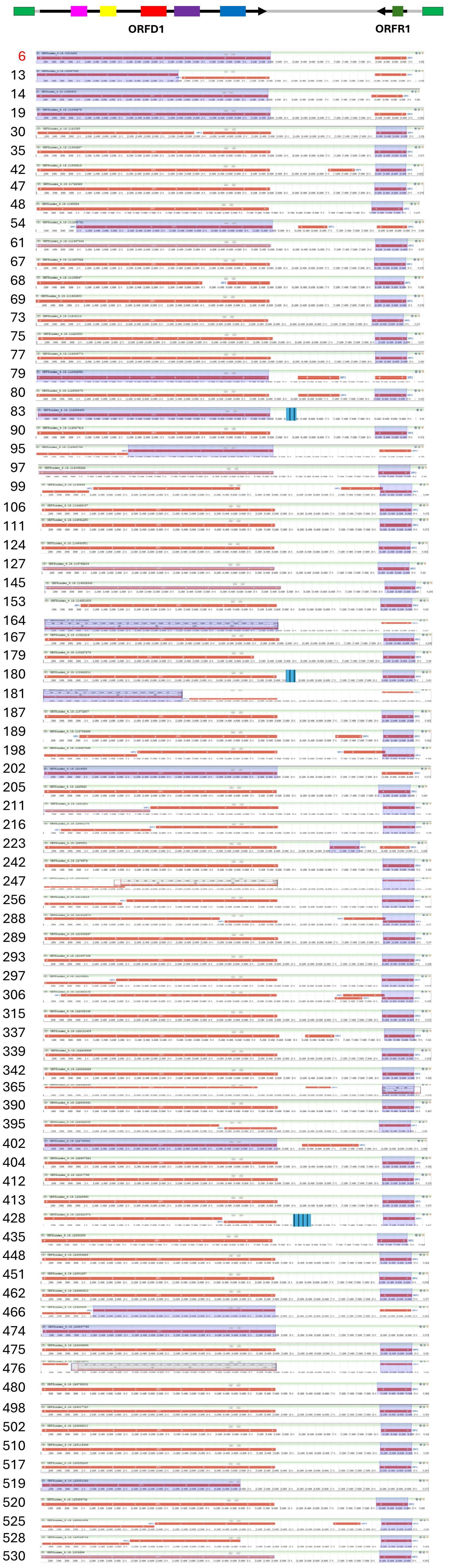

Artemisia tridentata 1

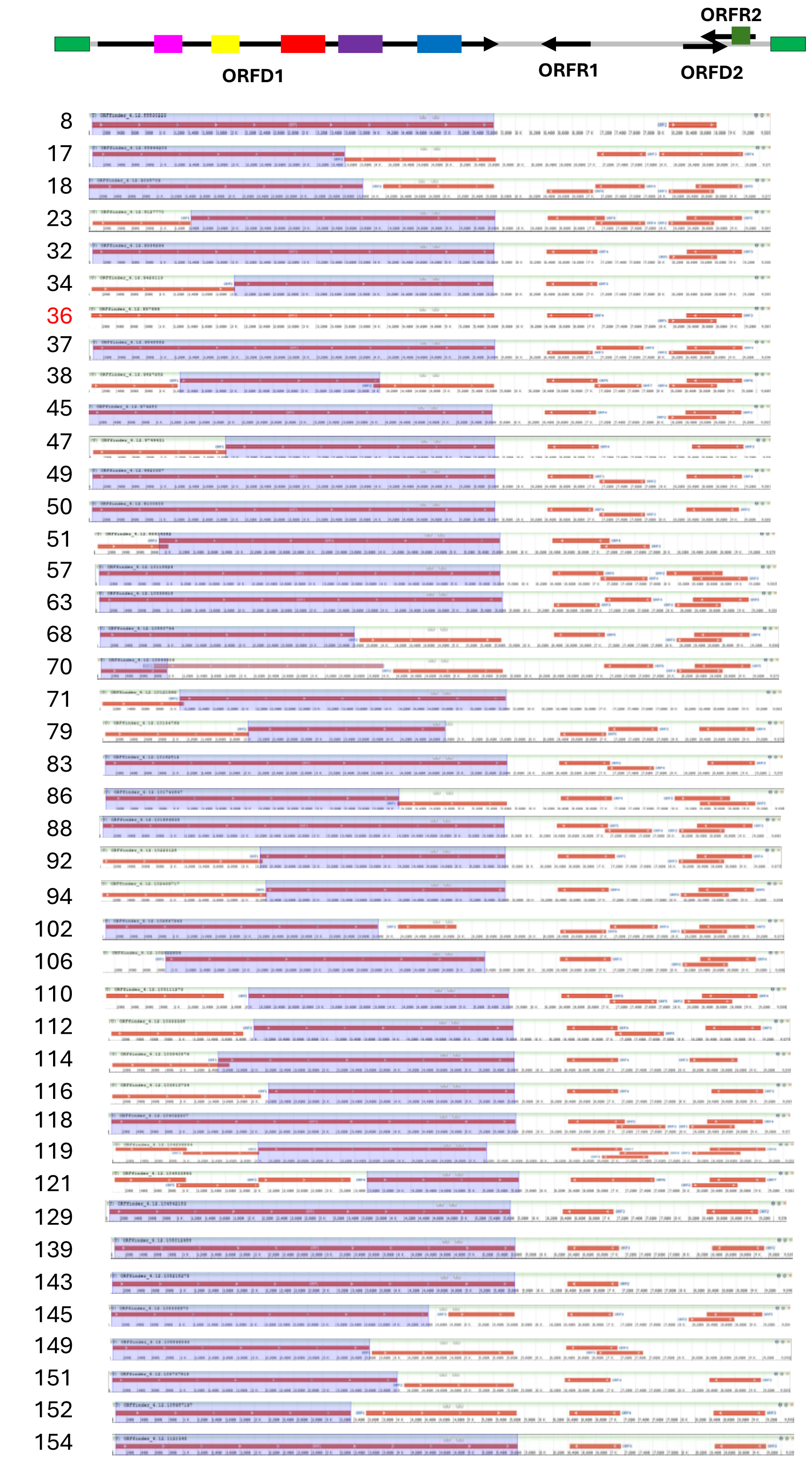

Artemisia tridentata 2a

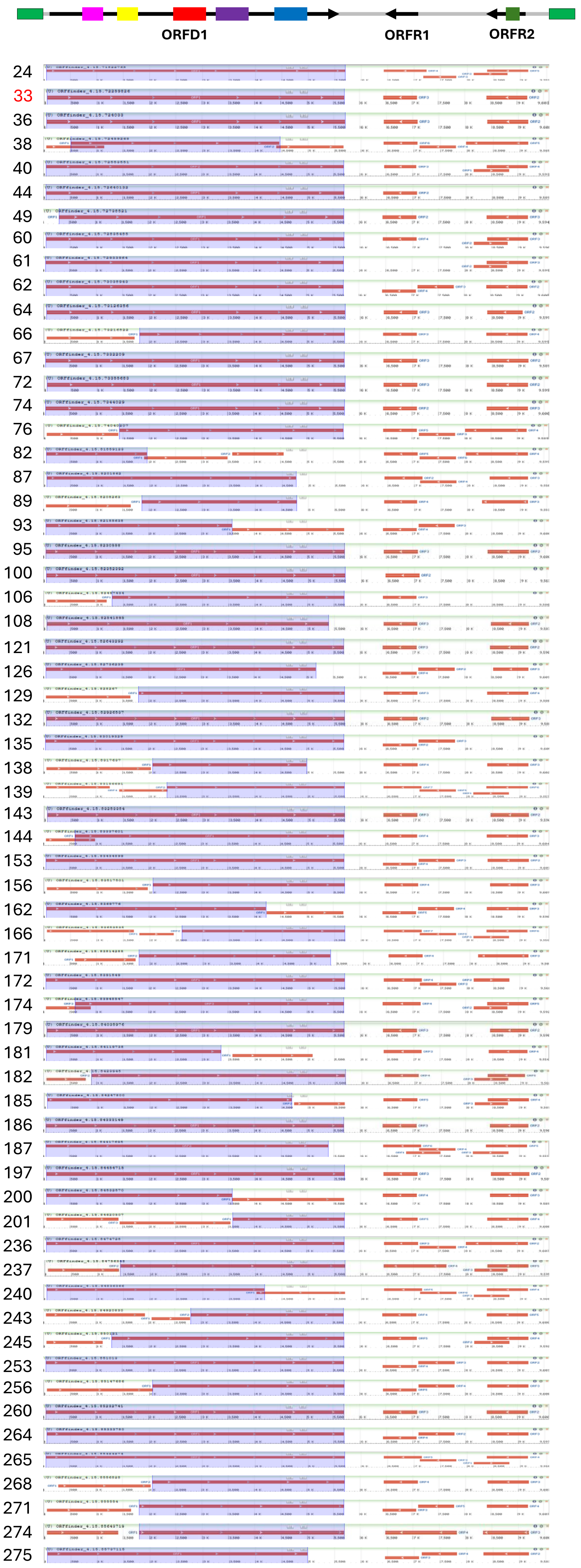

Avena atlantica 1

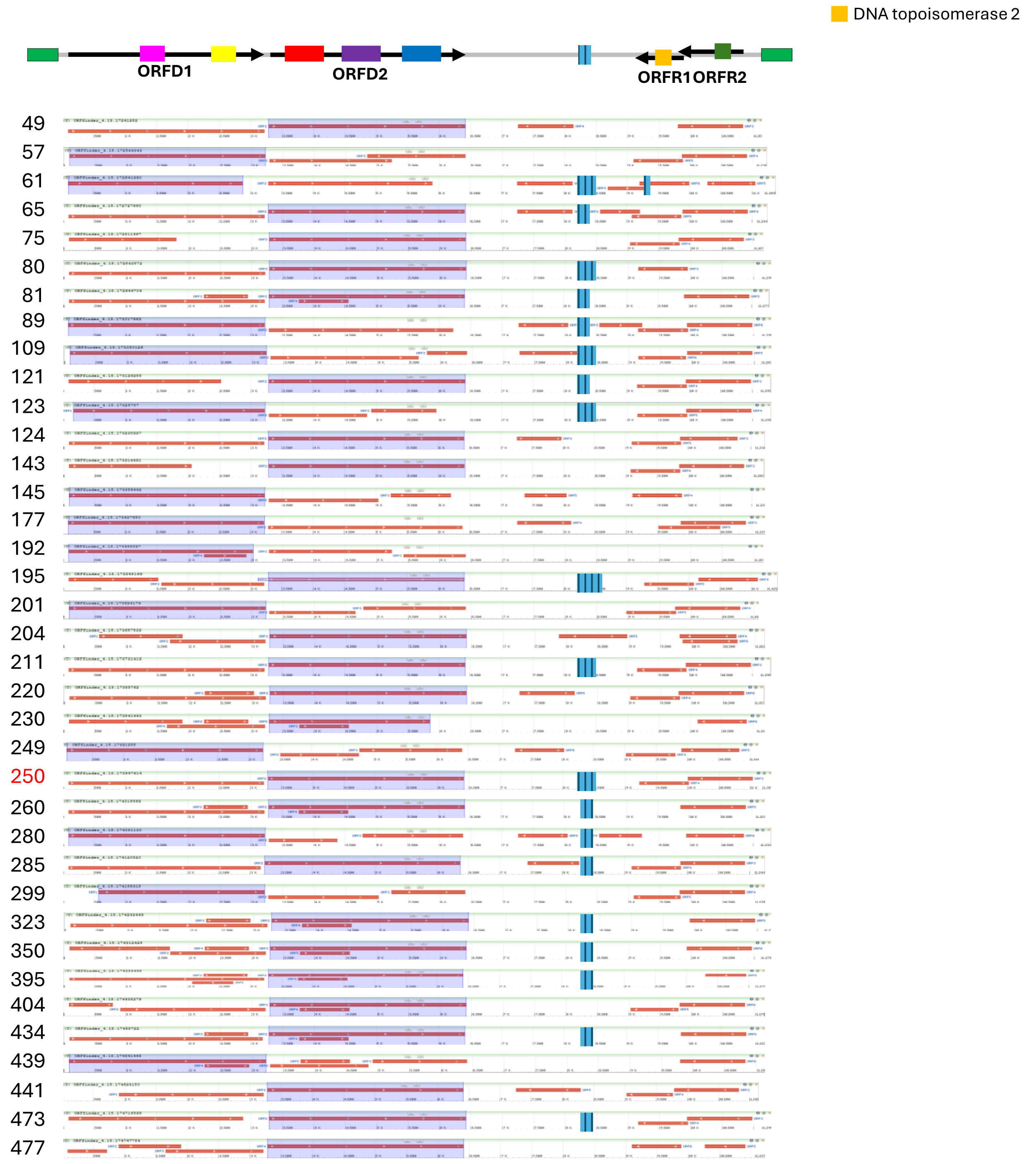

Ballota nigra 1

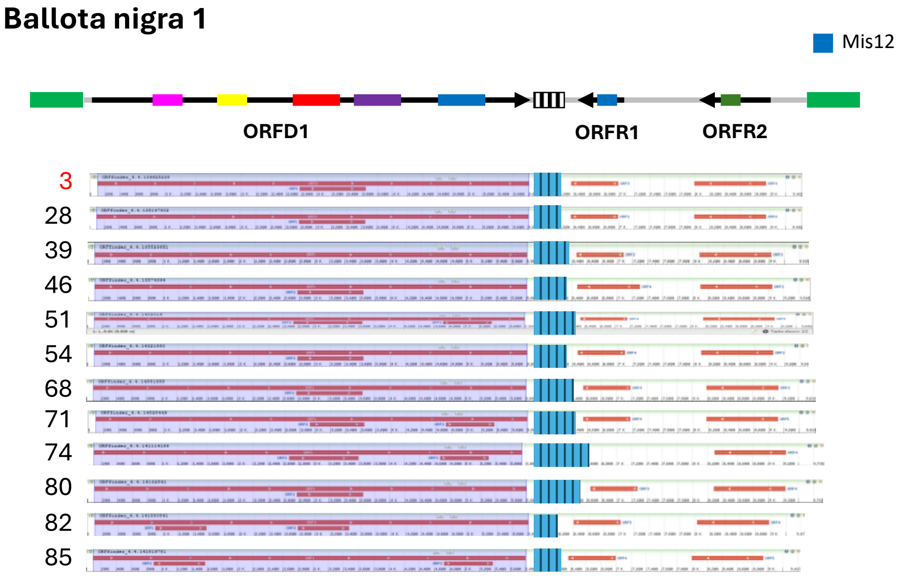

Ballota nigra 2a

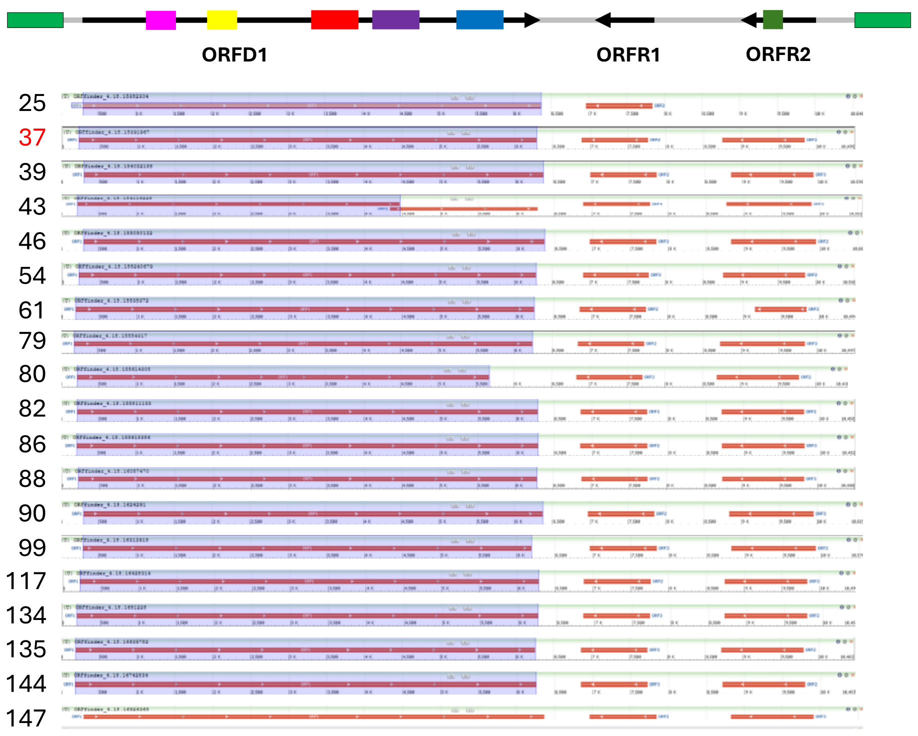

Ballota nigra 3

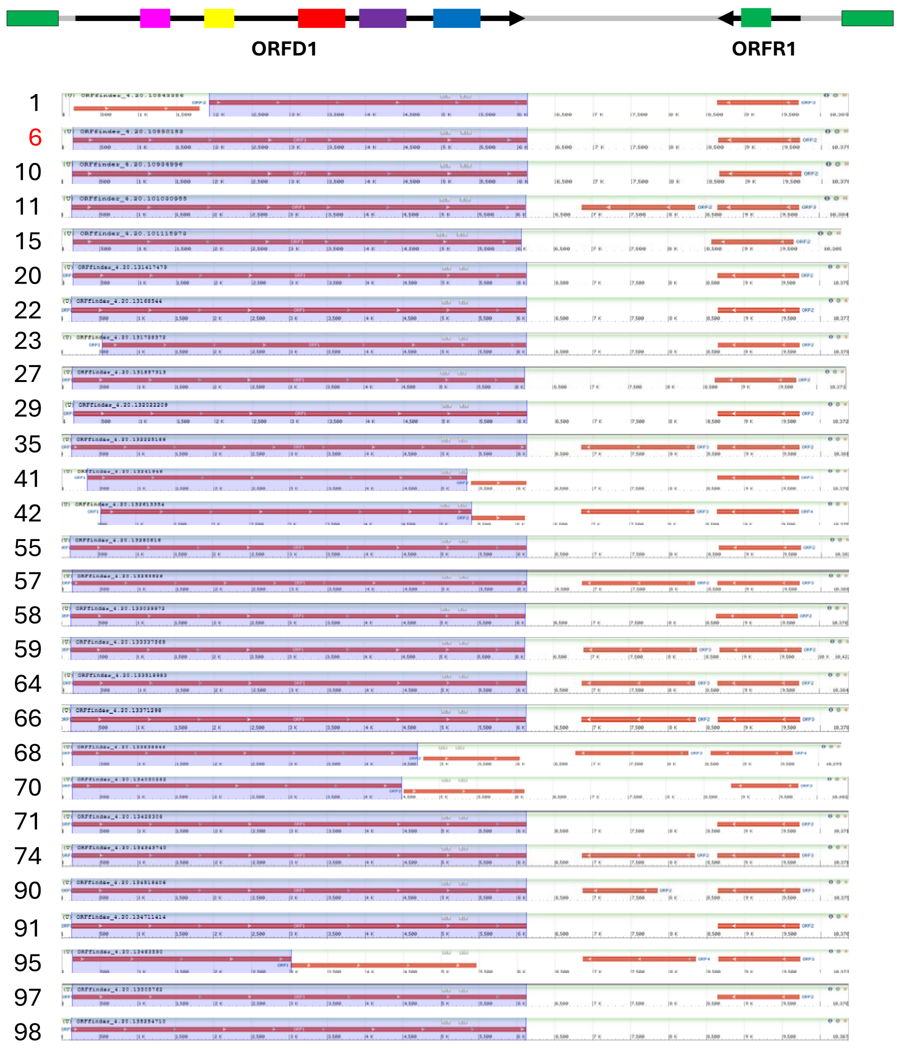

Ballota nigra 5a

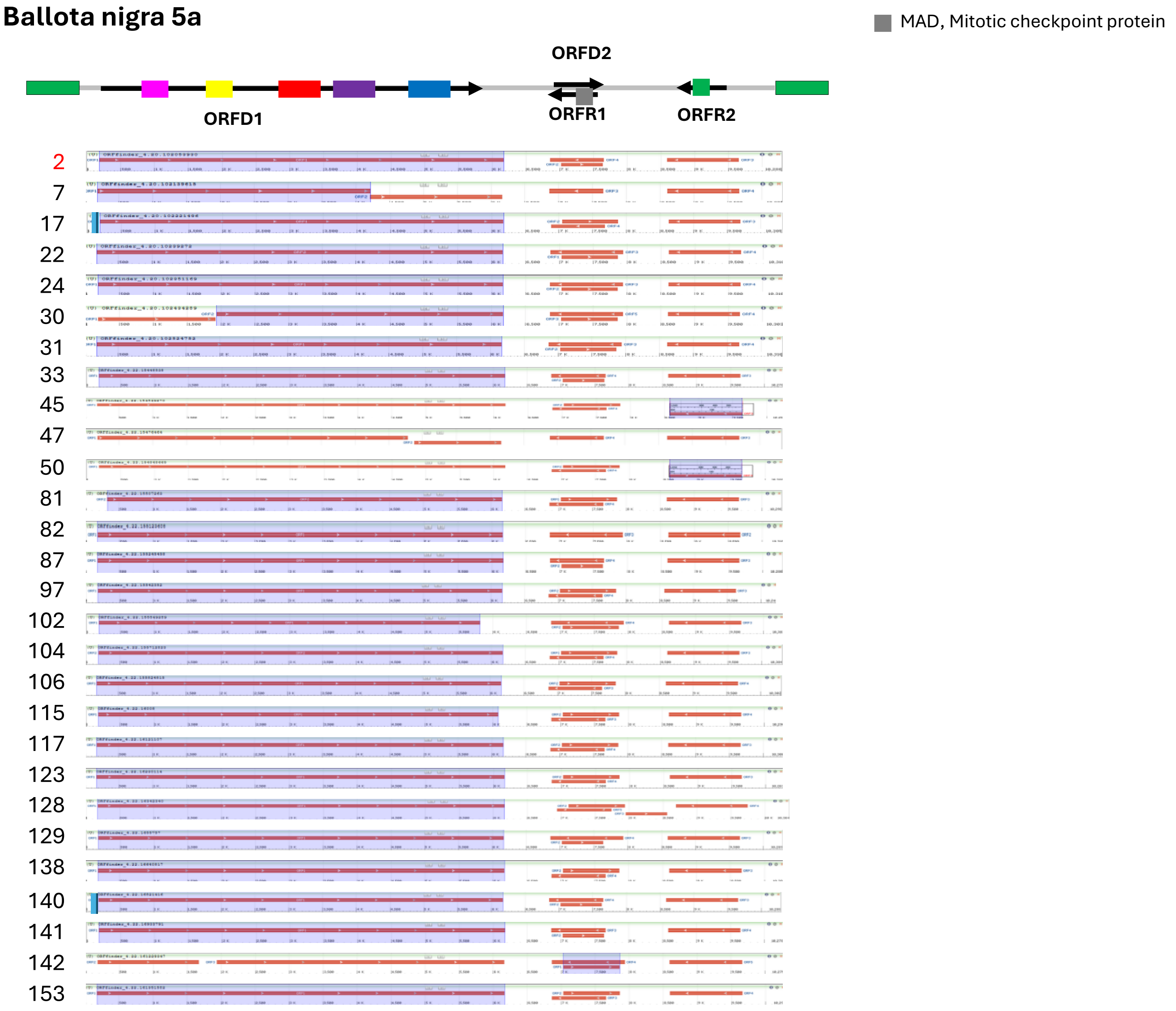

Camellia oleifera 1

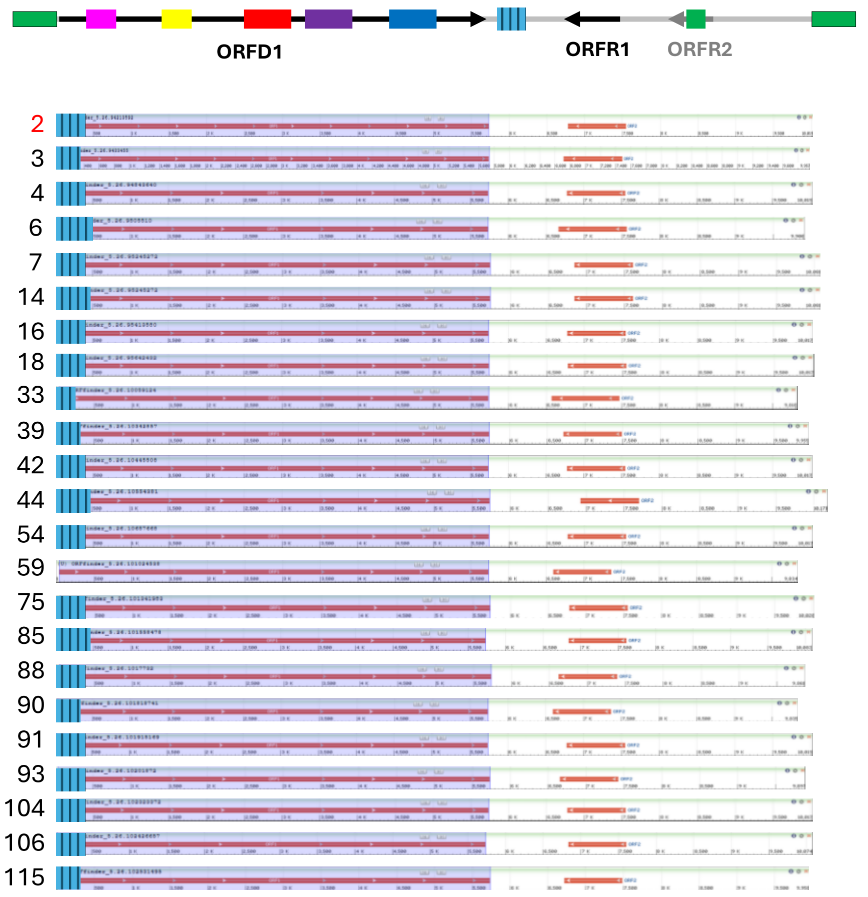

Camellia oleifera 2

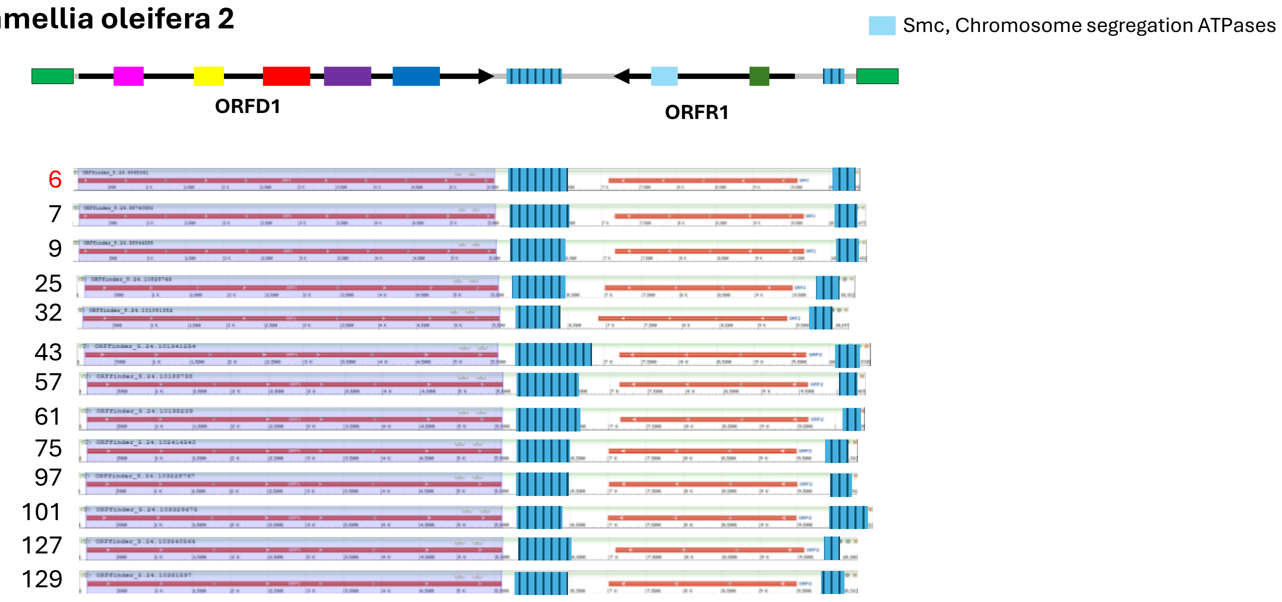

Camellia sinensis 4

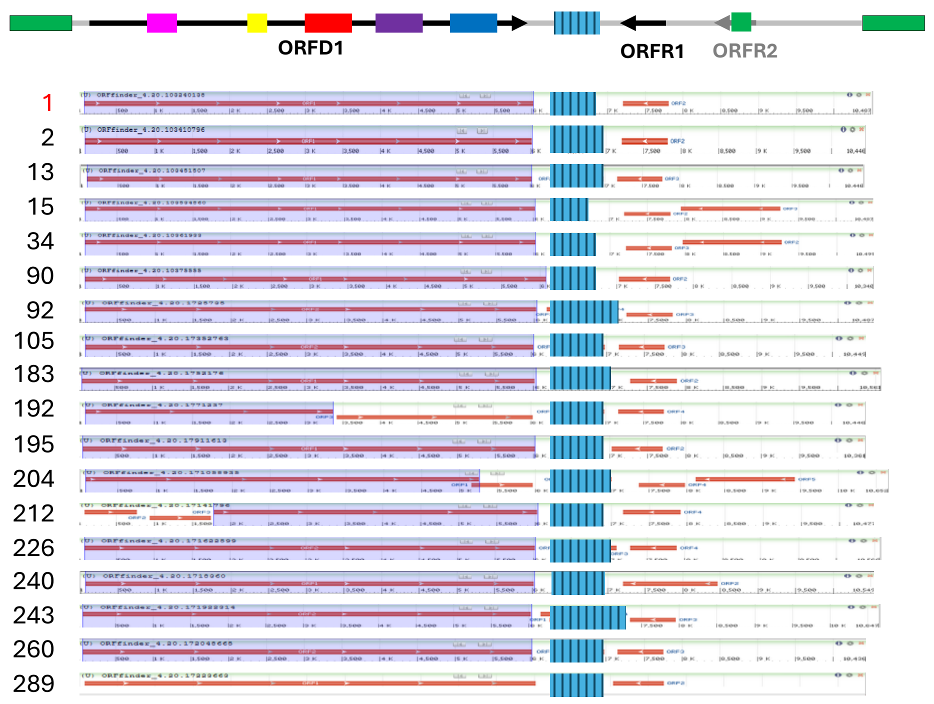

Centaurea solstitialis 2a

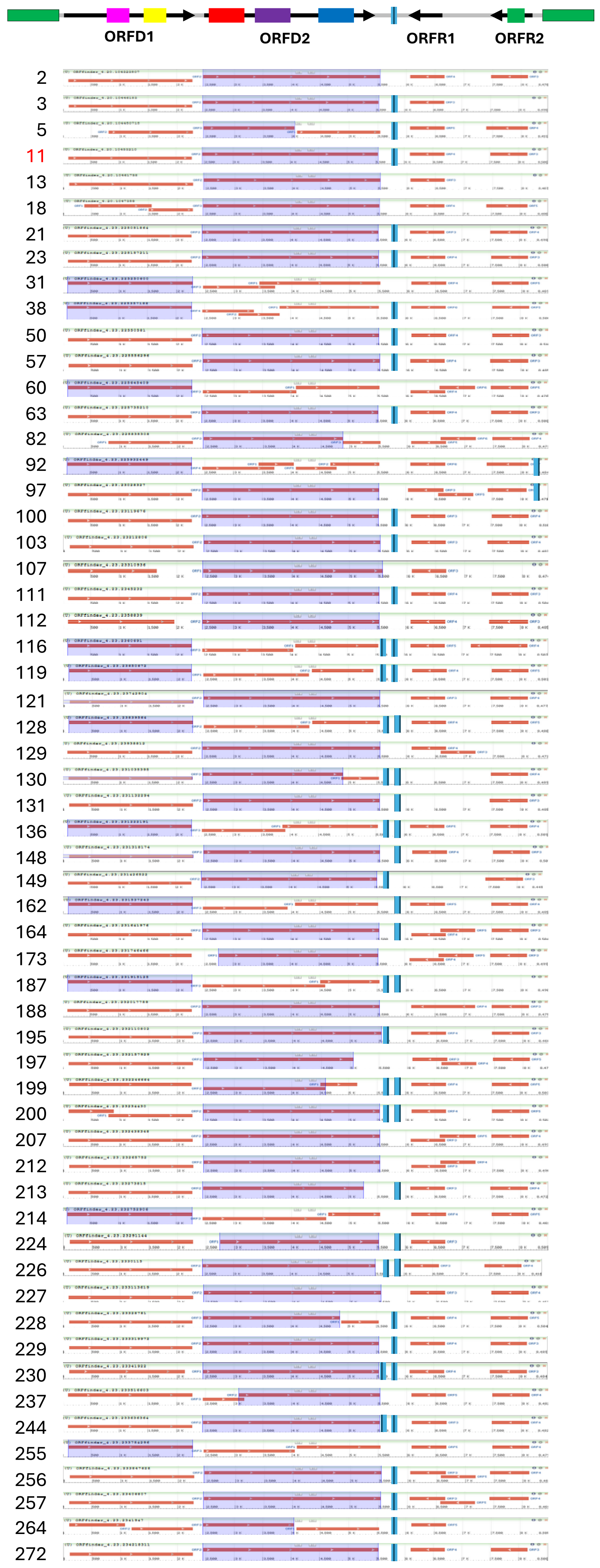

Chamaenerion angustifolium 2

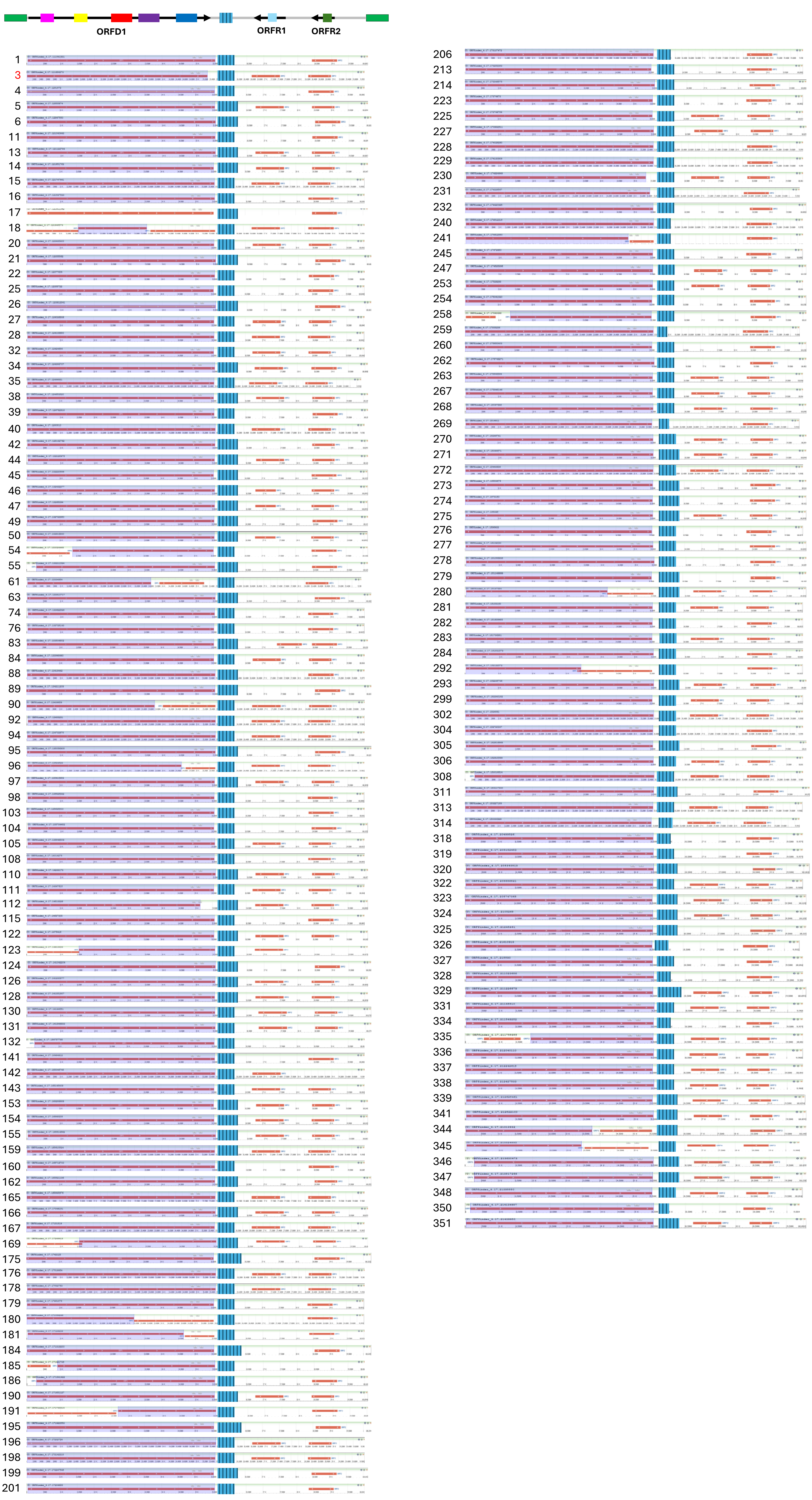

Comarum palustre 1

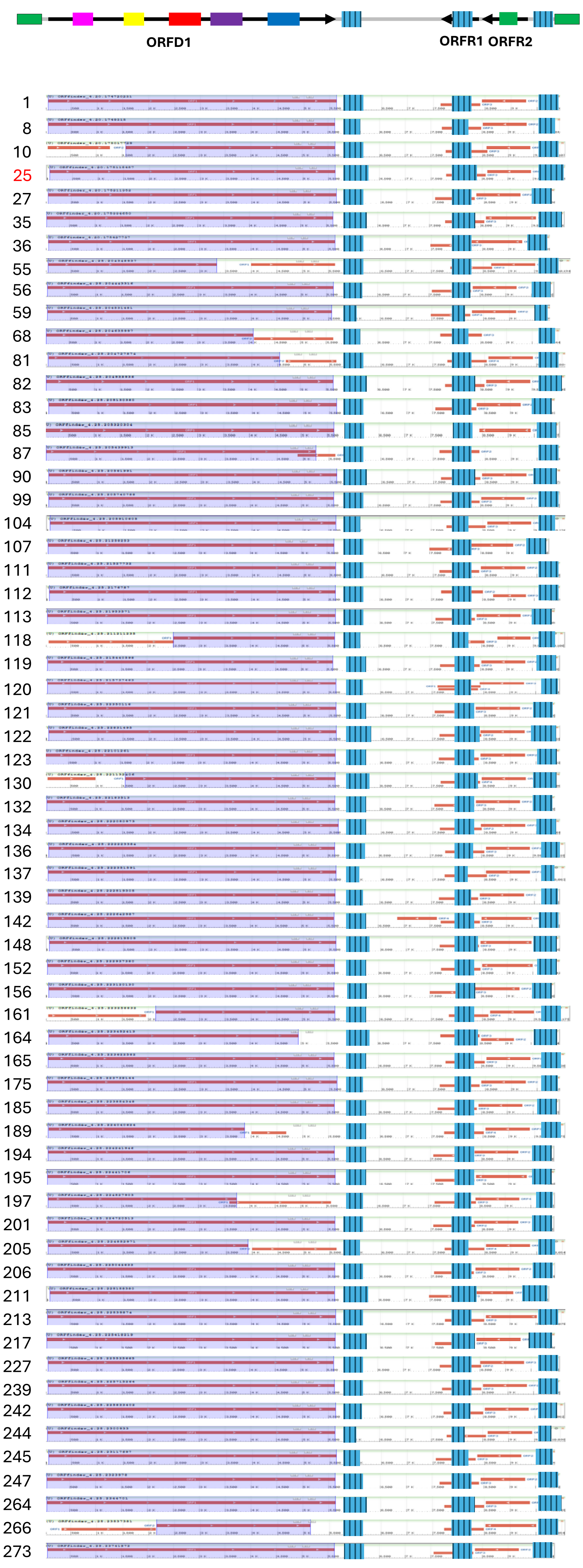

Dimocarpus longan 1

Smc, Chromosome segregation ATPases

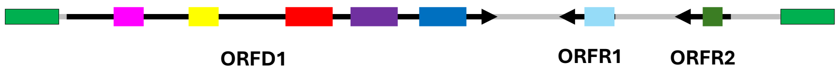

2
5
10
31
42
49
54
68
77
80

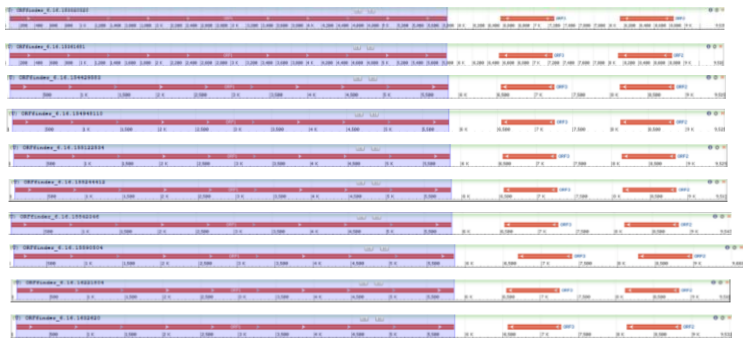

Glebionis coronaria 2a

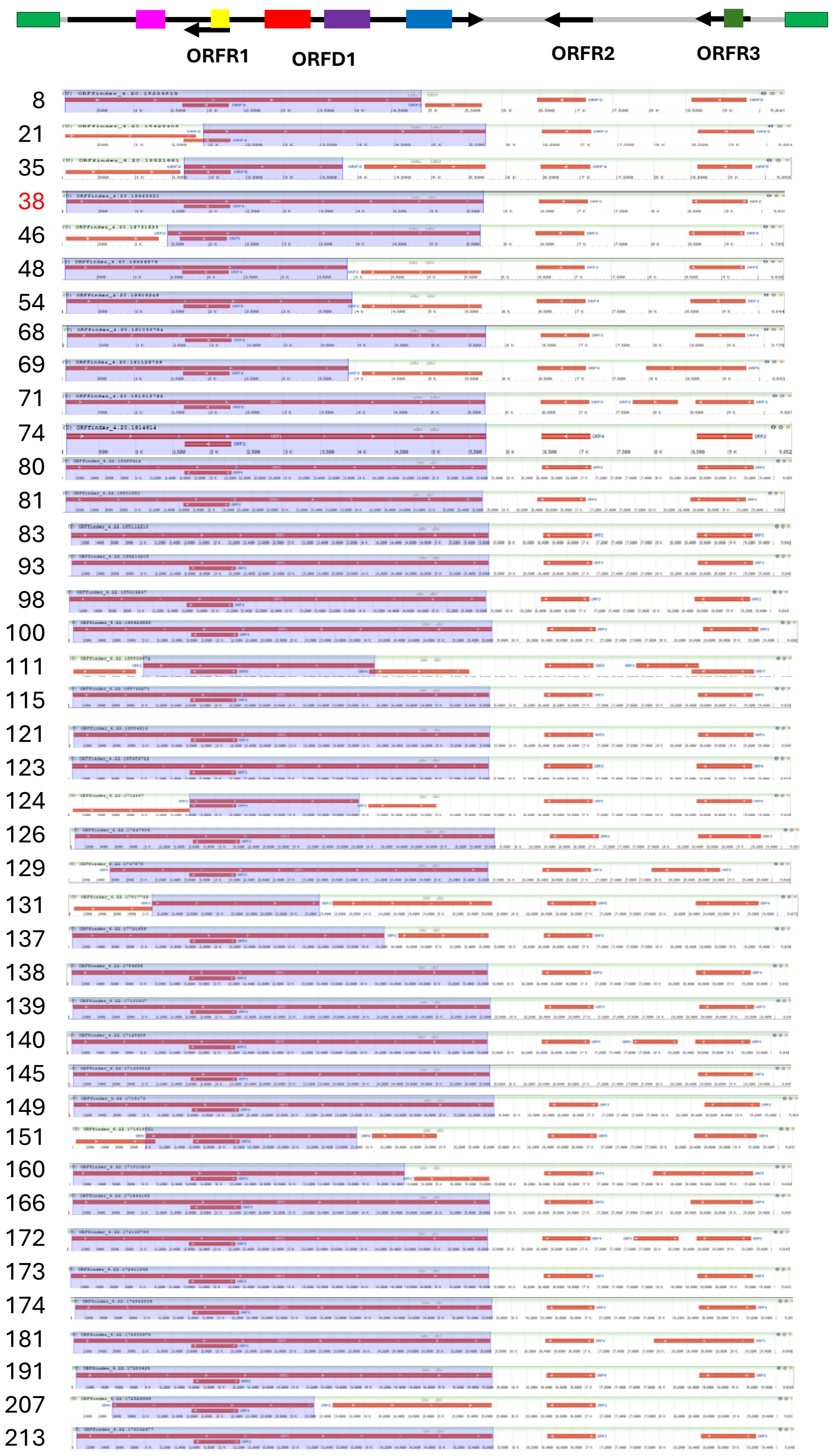

Glebionis coronaria 2b

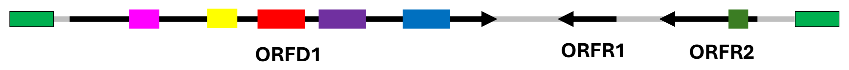

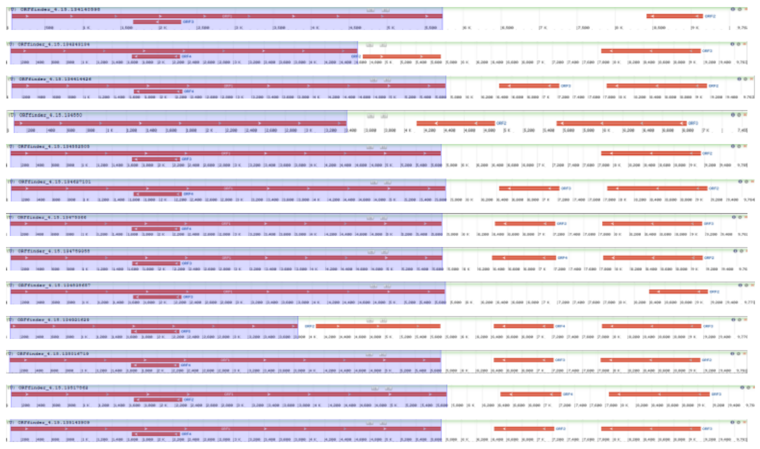

47
63
73
92
108
110
141
142
154
161
168
198
222

Helianthus annuus 2

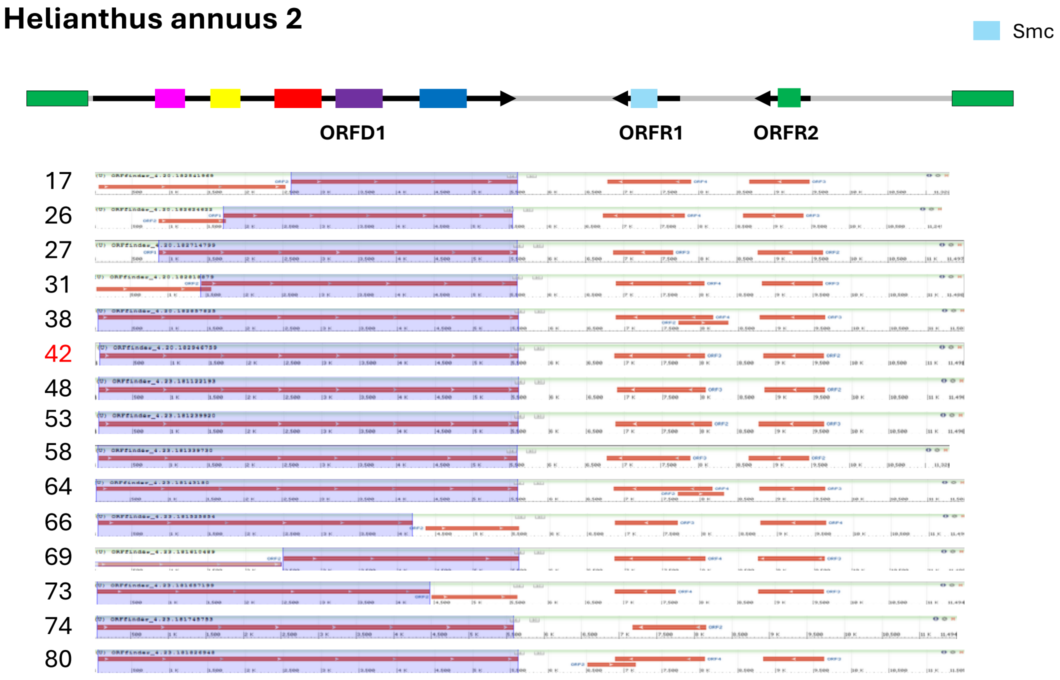

Helianthus annuus 3a

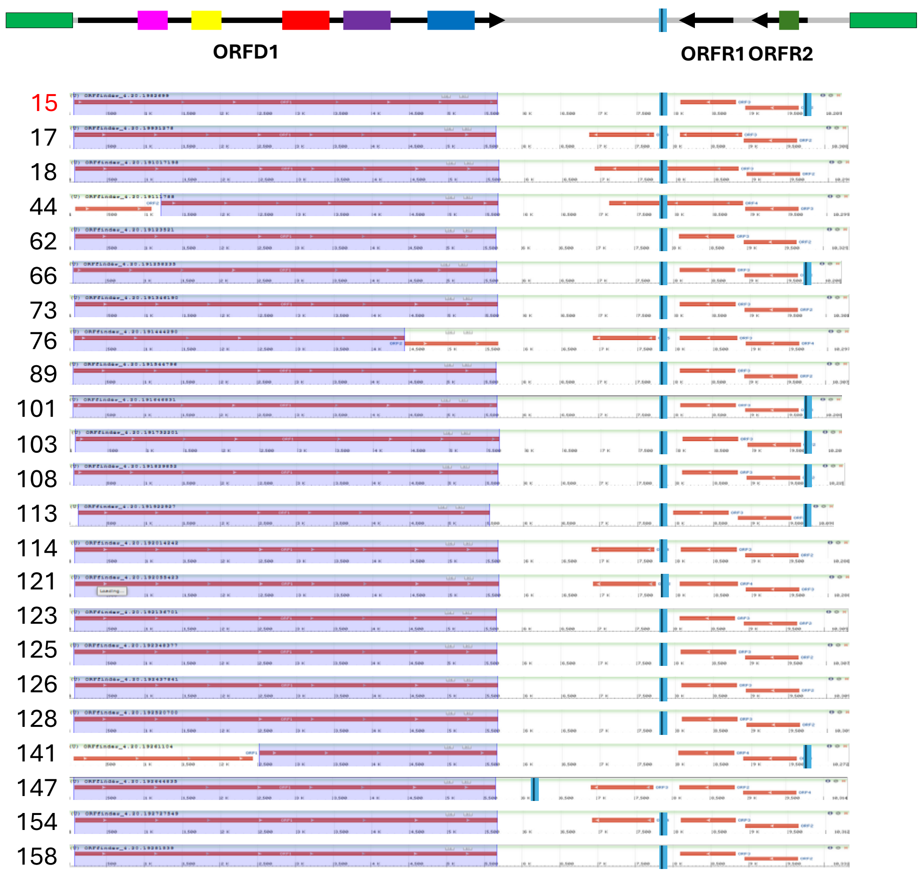

Helianthus annuus 4

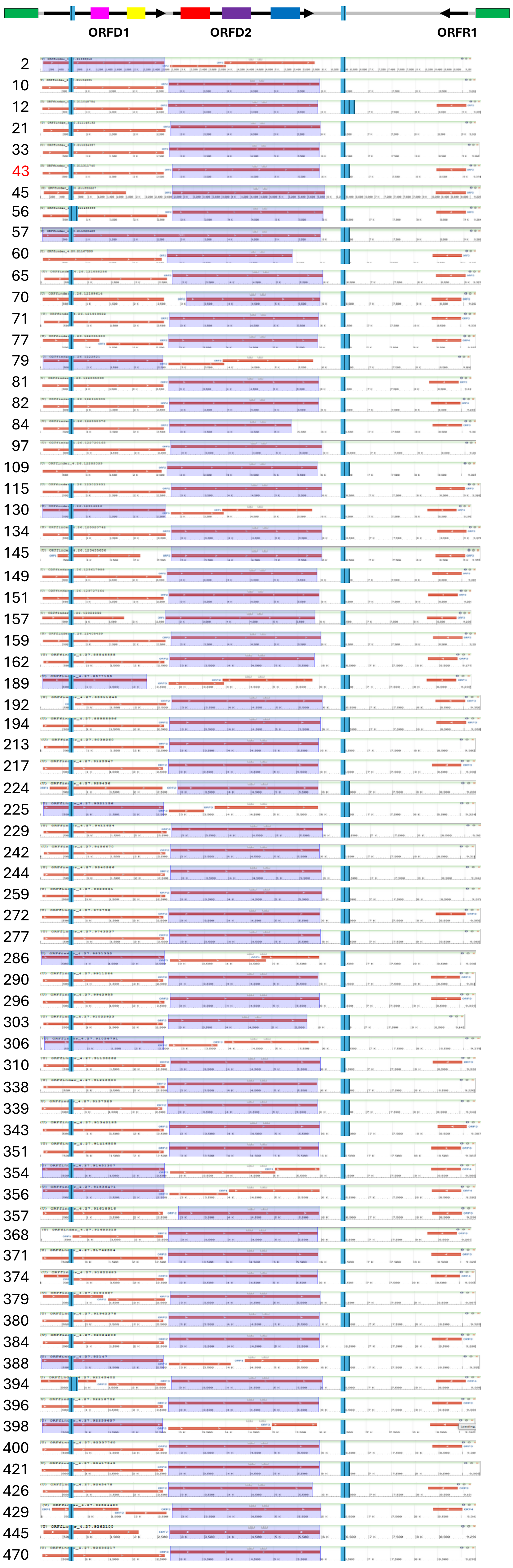

Helianthus annuus 5

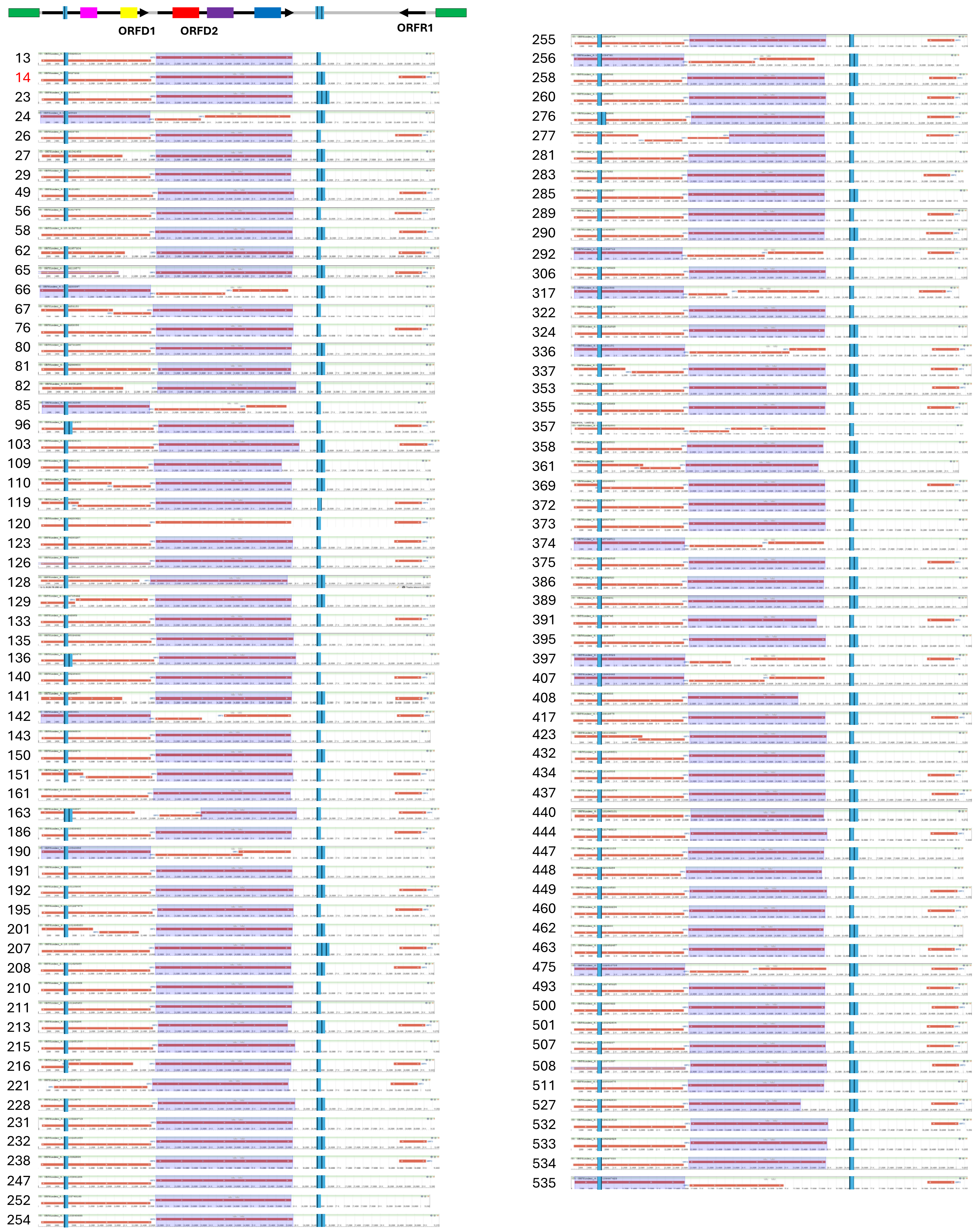

Heracleum sosnowskyi 1

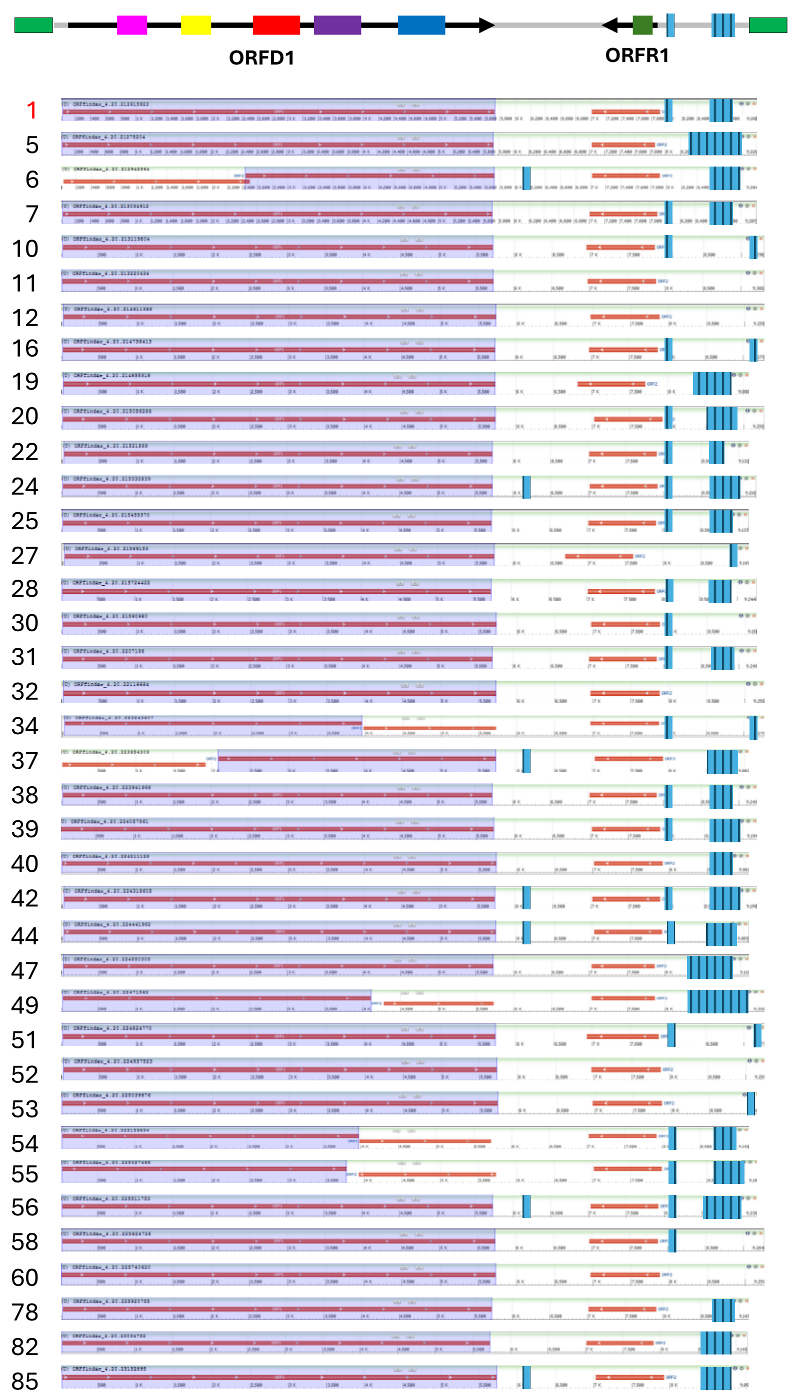

Heracleum sosnowskyi 2a

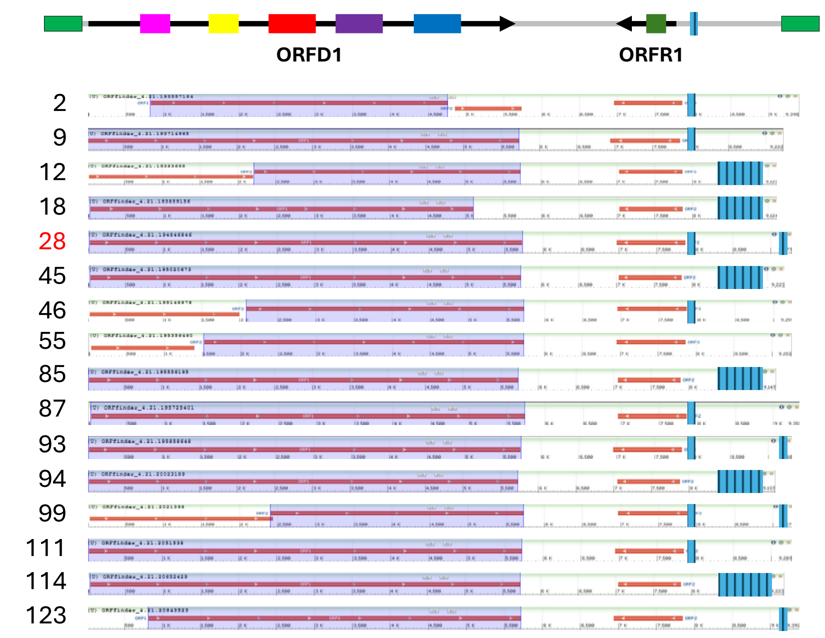

Linaria vulgaris 1

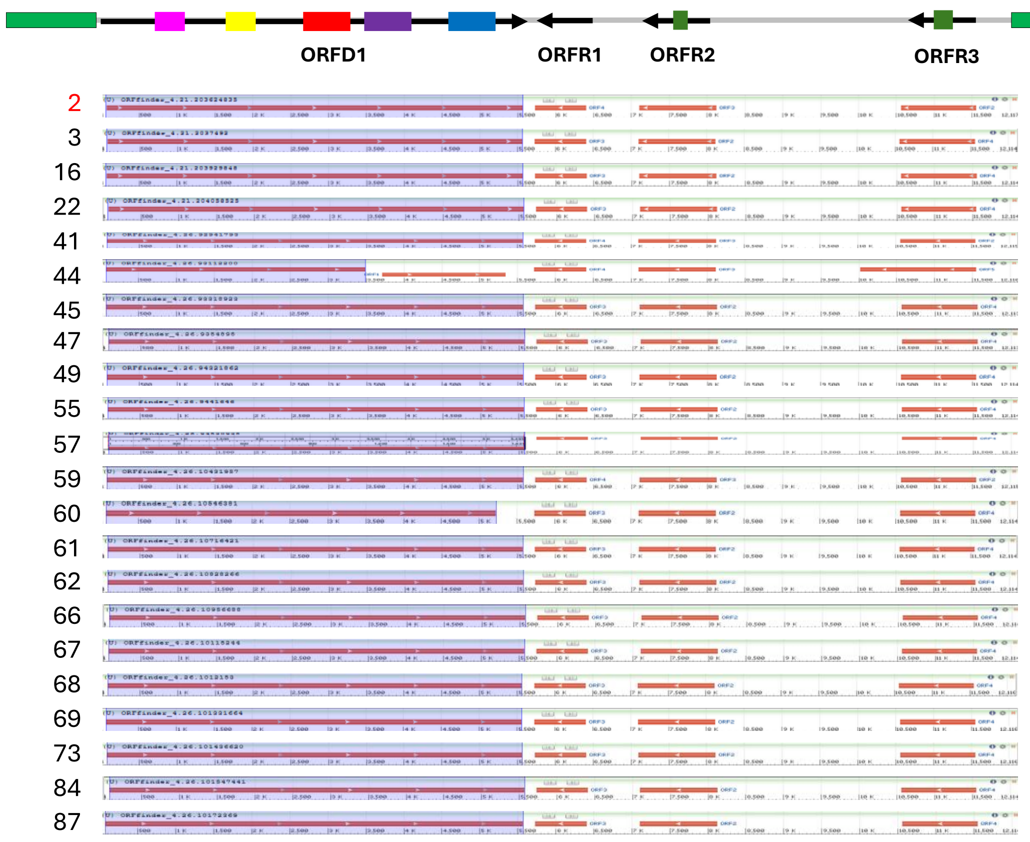

Lotus japonicus 1

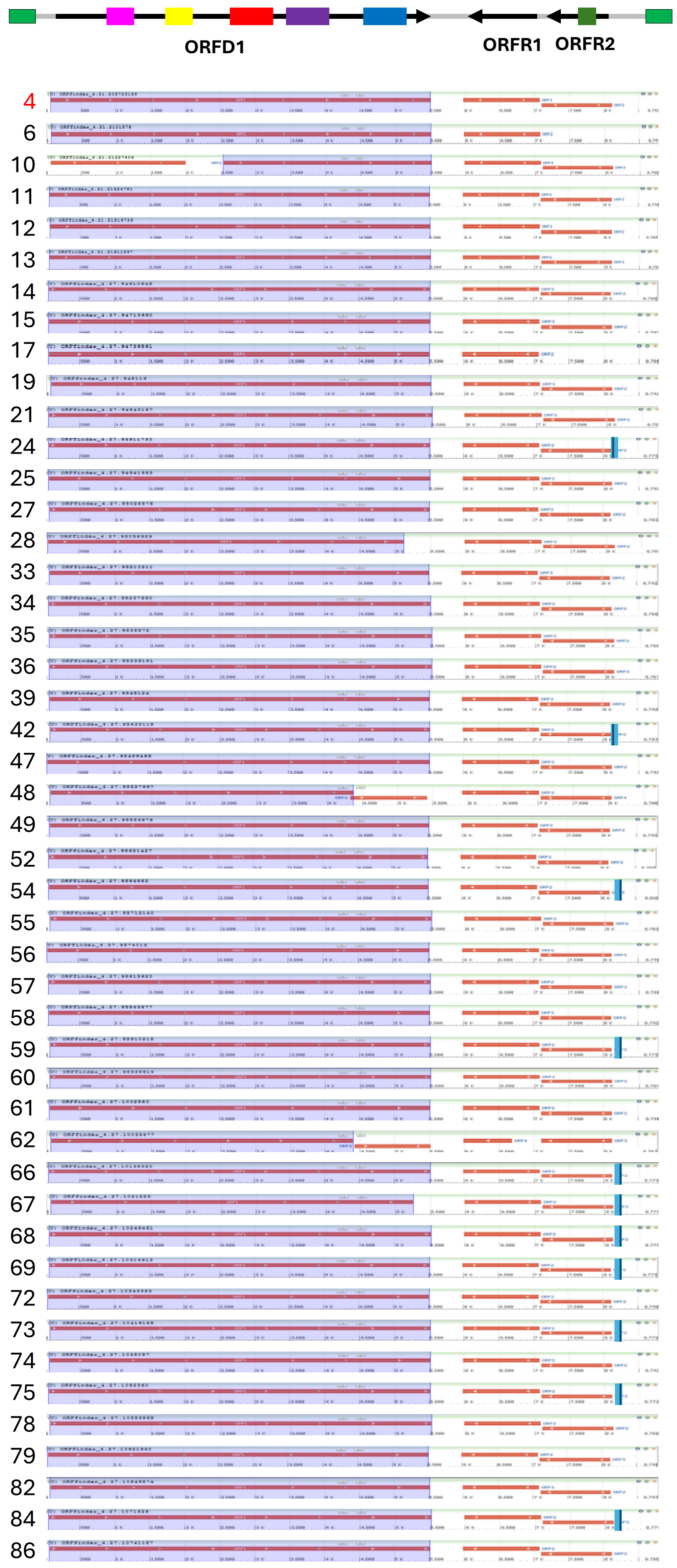

Medicago ruthenica 1

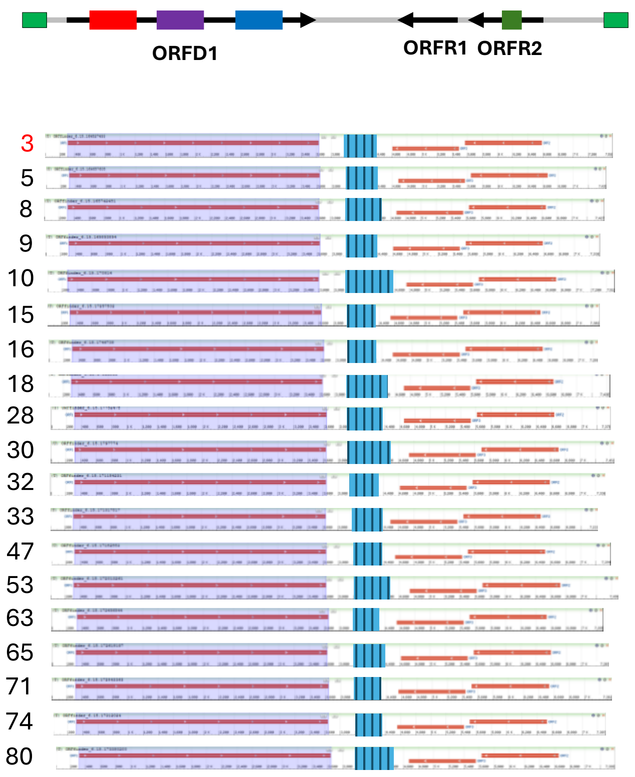

Nyssa sinensis 1

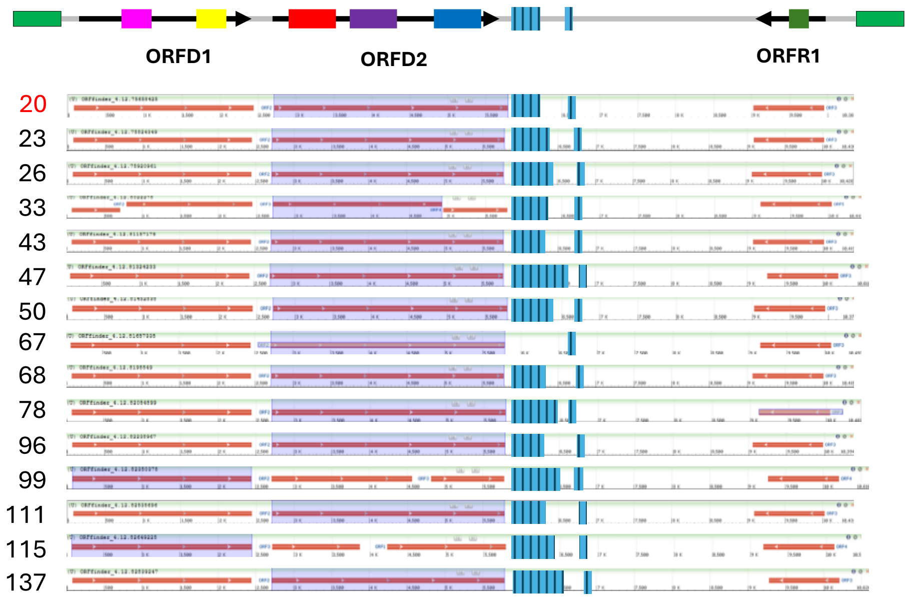

Oryza rufipogon 1c

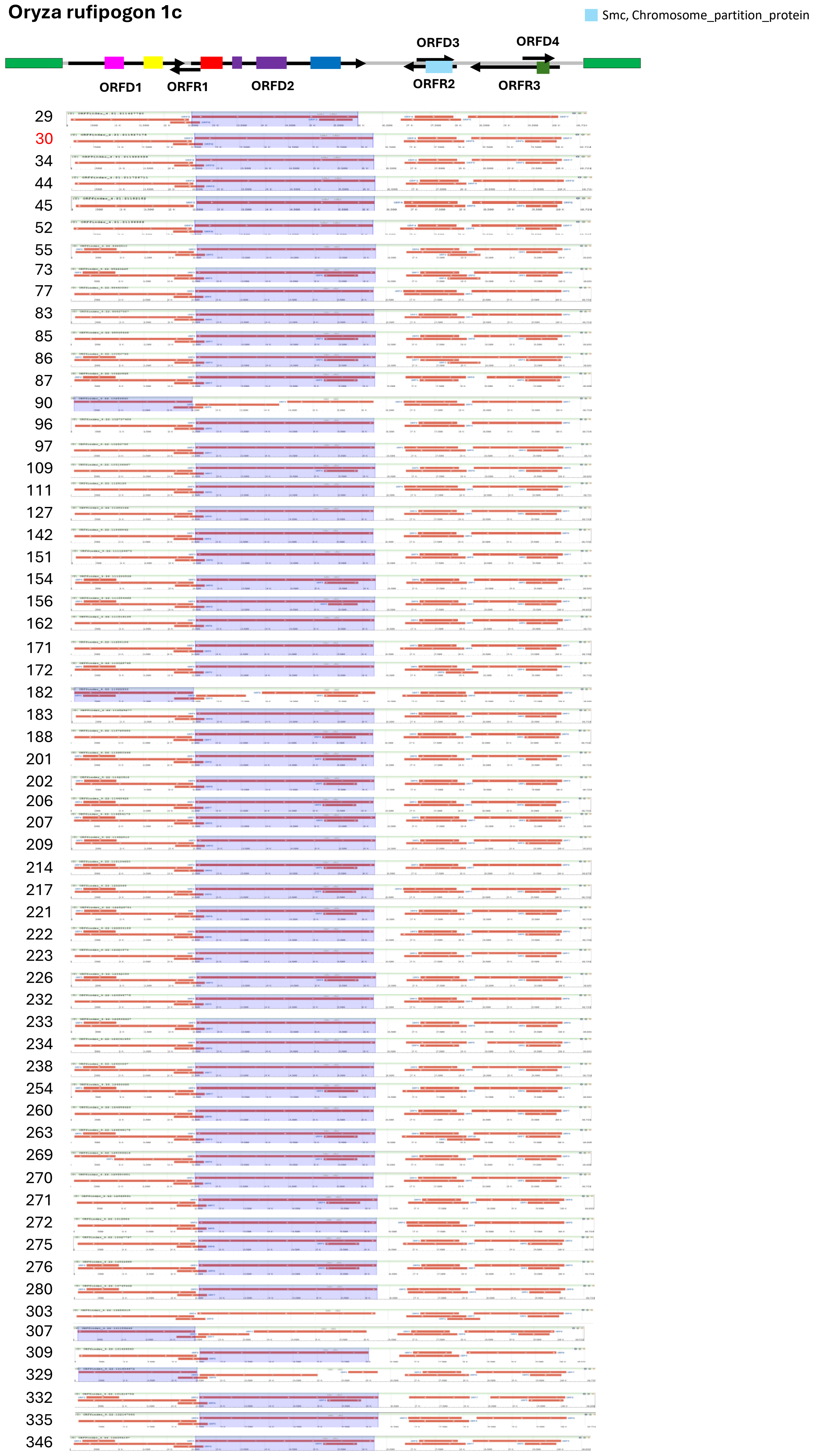

Oryza rufipogon 1d

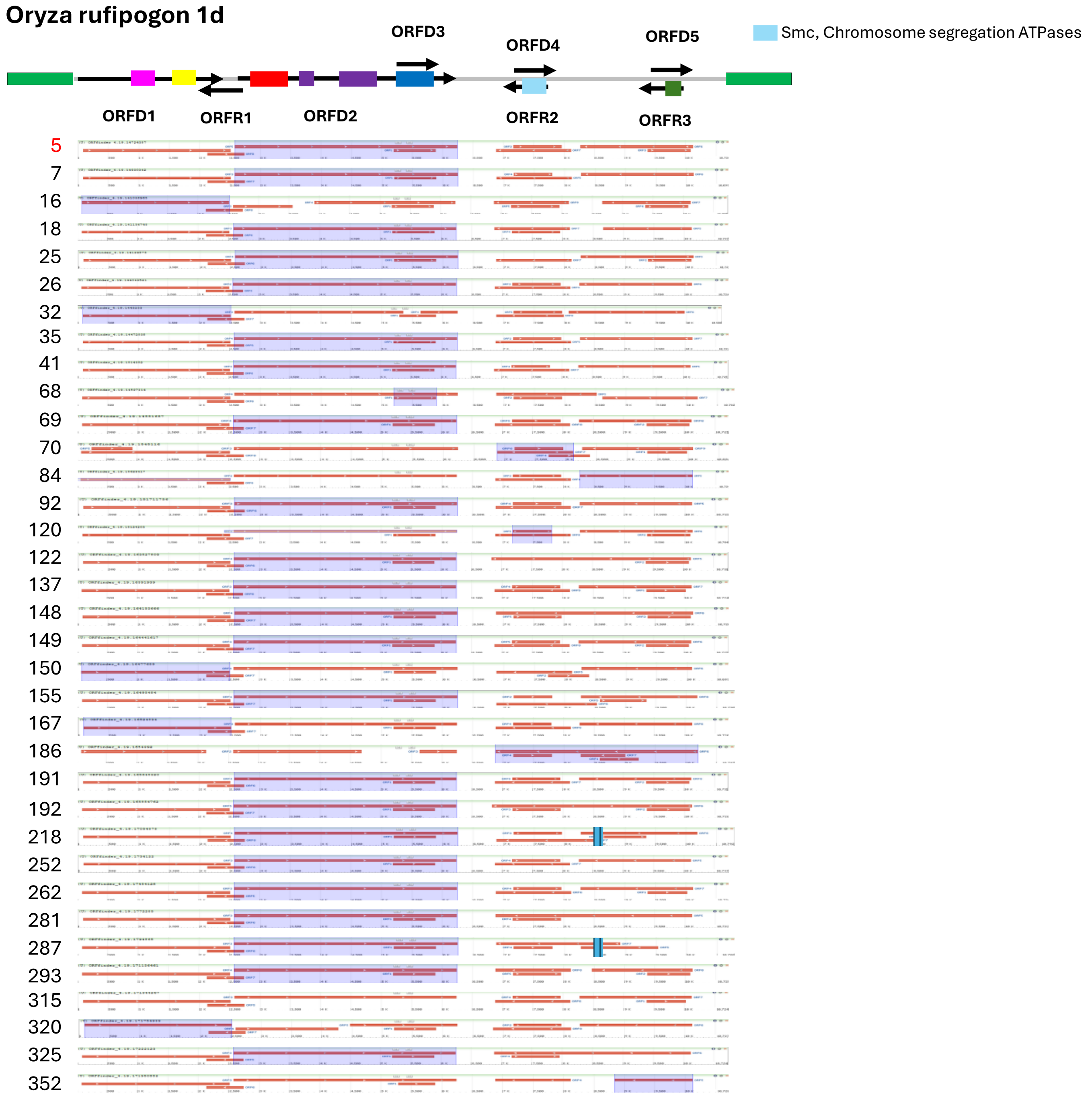

Oryza rufipogon 2

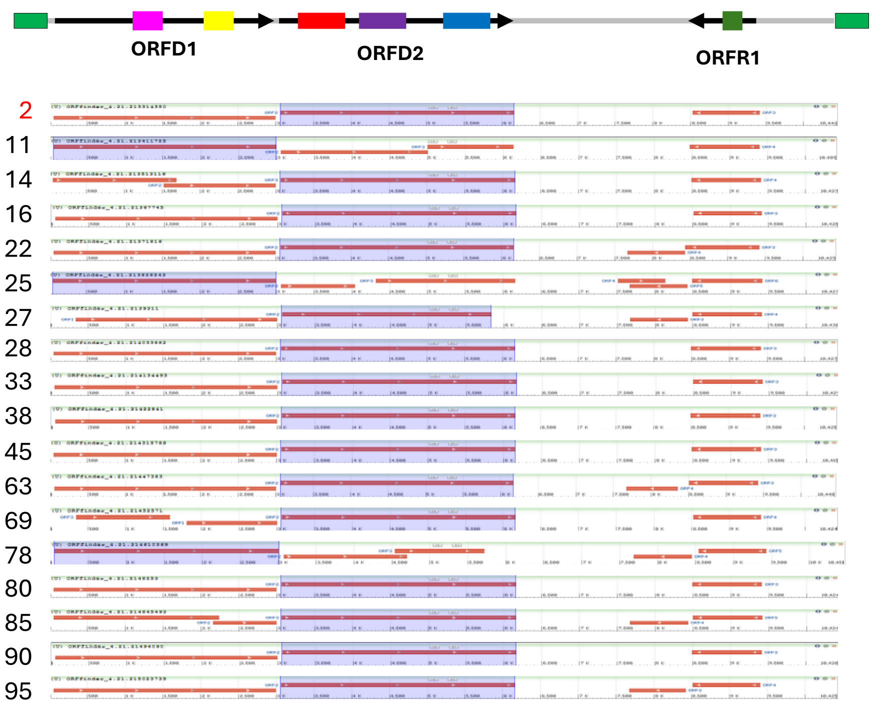

Poa annua 1a

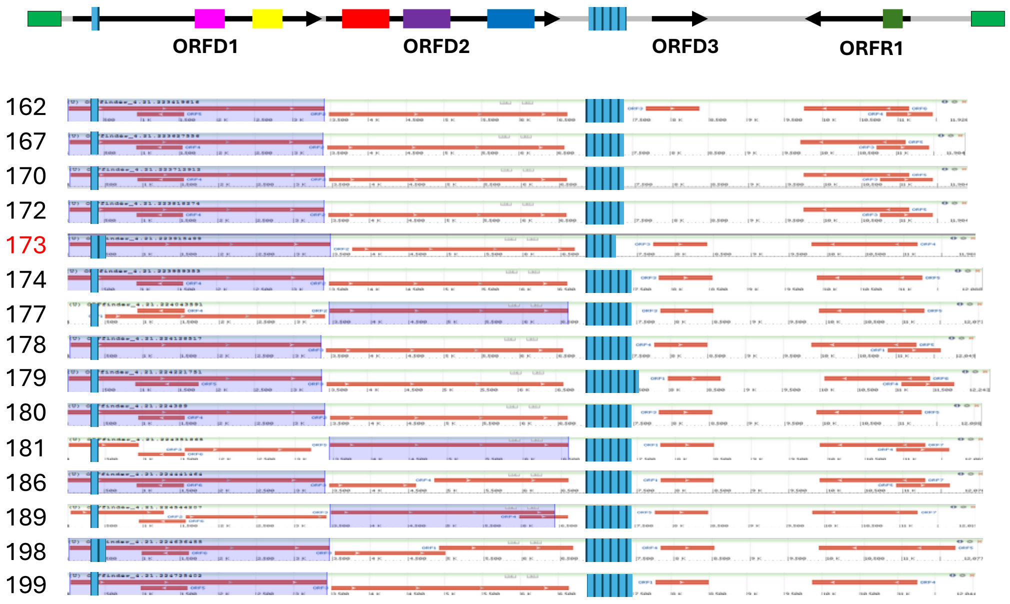

ORFD1
ORFD2
ORFD3
ORFR1
162
167
170
172
173
174
177
178
179
180
181
186
189
198
199

Poa annua 1b

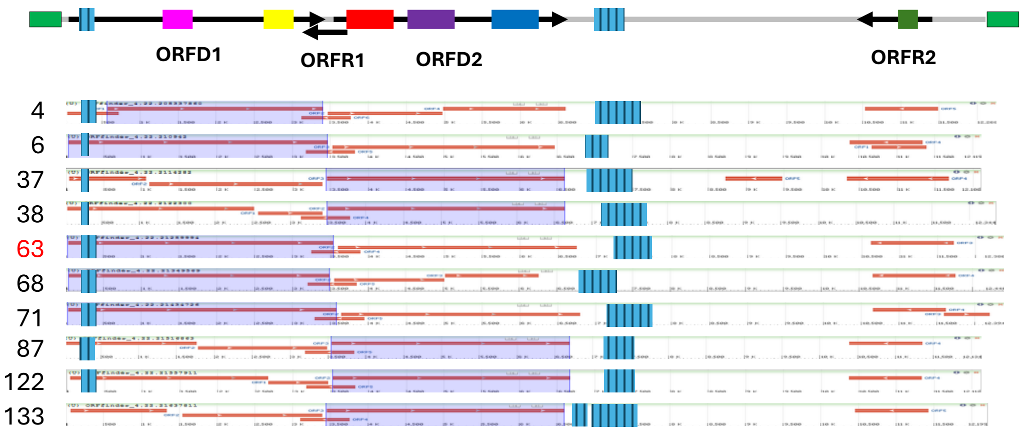

Poa annua 4a

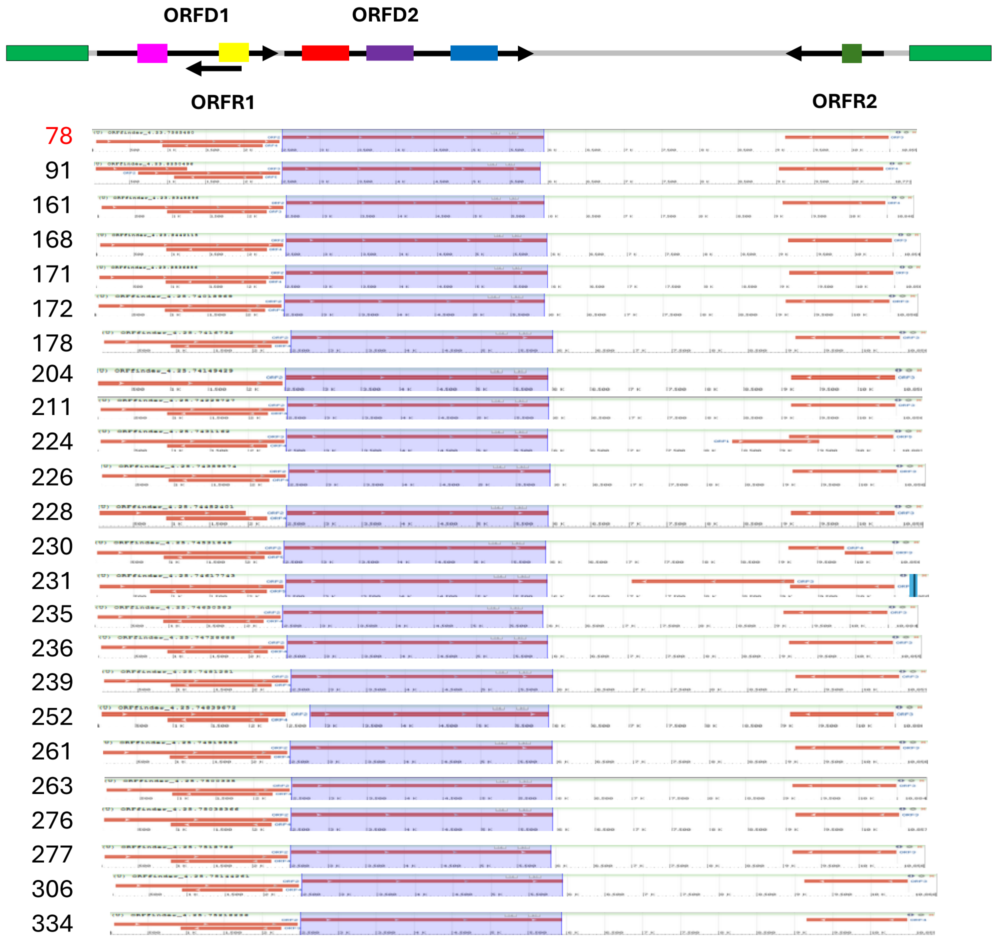
ORFD1
ORFD2
ORFR1
ORFR2
78
91
161
168
171
172
178
204
211
224
226
228
230
231
235
236
239
252
261
263
276
277
306
334

Poa annua 4b

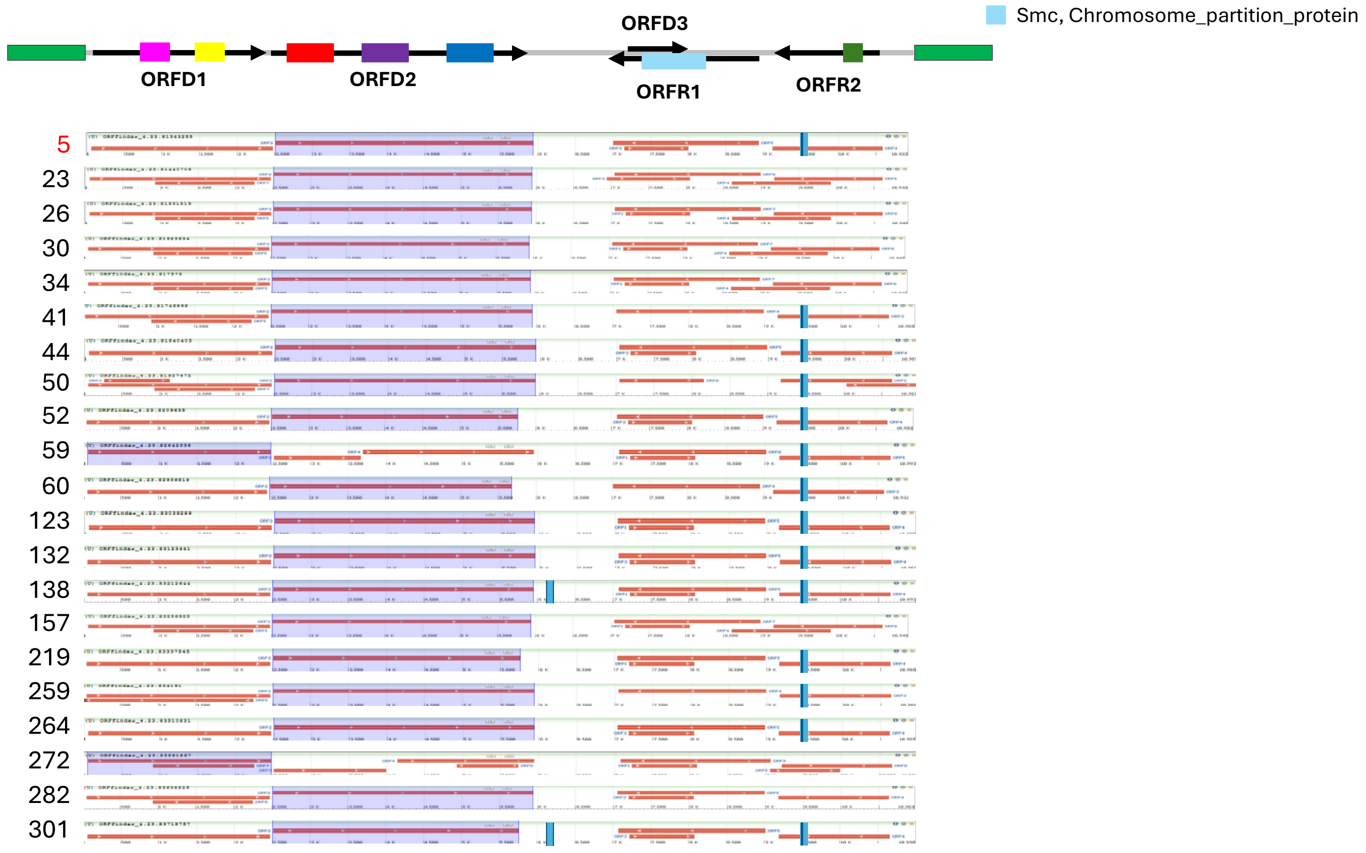
Smc, Chromosome_partition_protein
ORFD1
ORFD2
ORFD3
ORFR1
ORFR2
5
23
26
30
34
41
44
50
52
59
60
123
132
138
157
219
259
264
272
282
301

Rhynchospora pubera 3

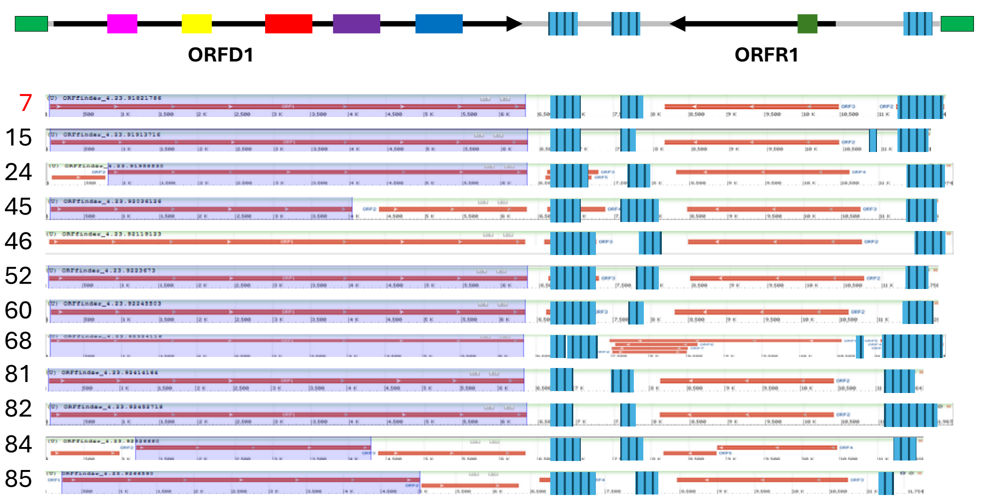

Saccharum spontaneum 1a

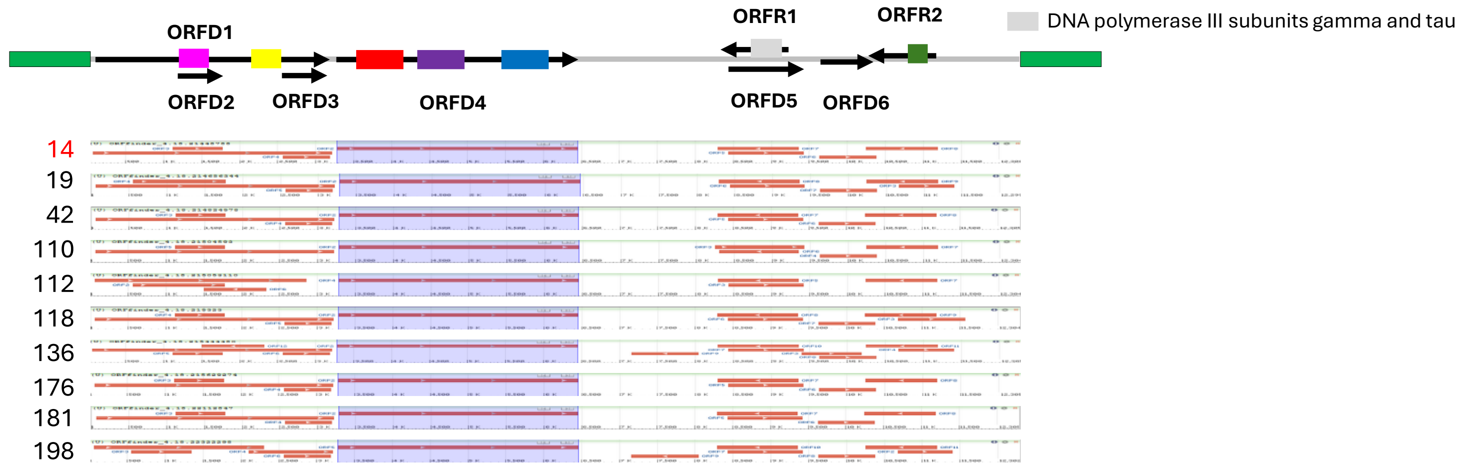

Salvia splendens 1

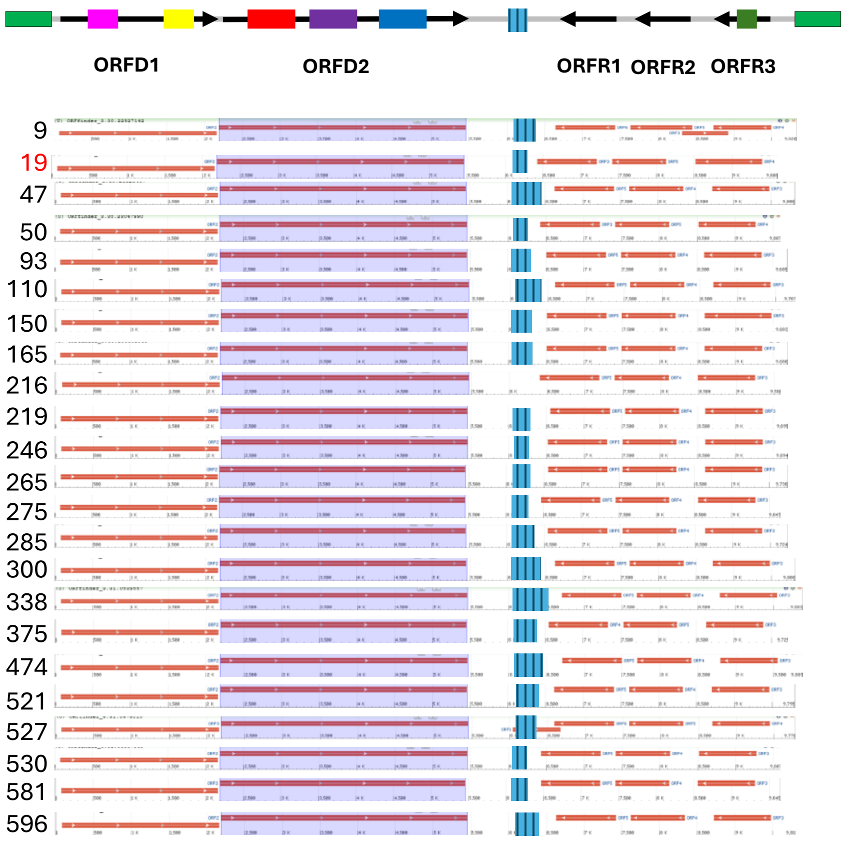

Sambucus nigra 2

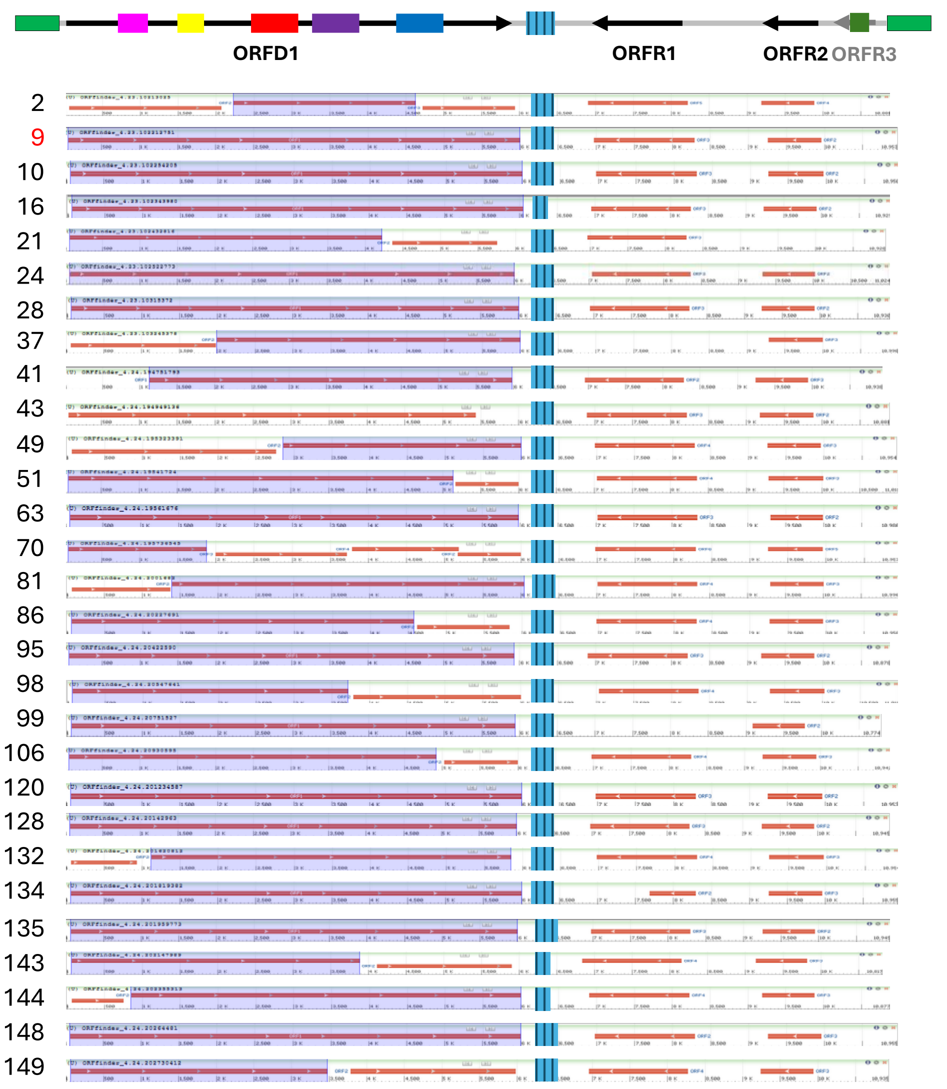

Sambucus nigra 3

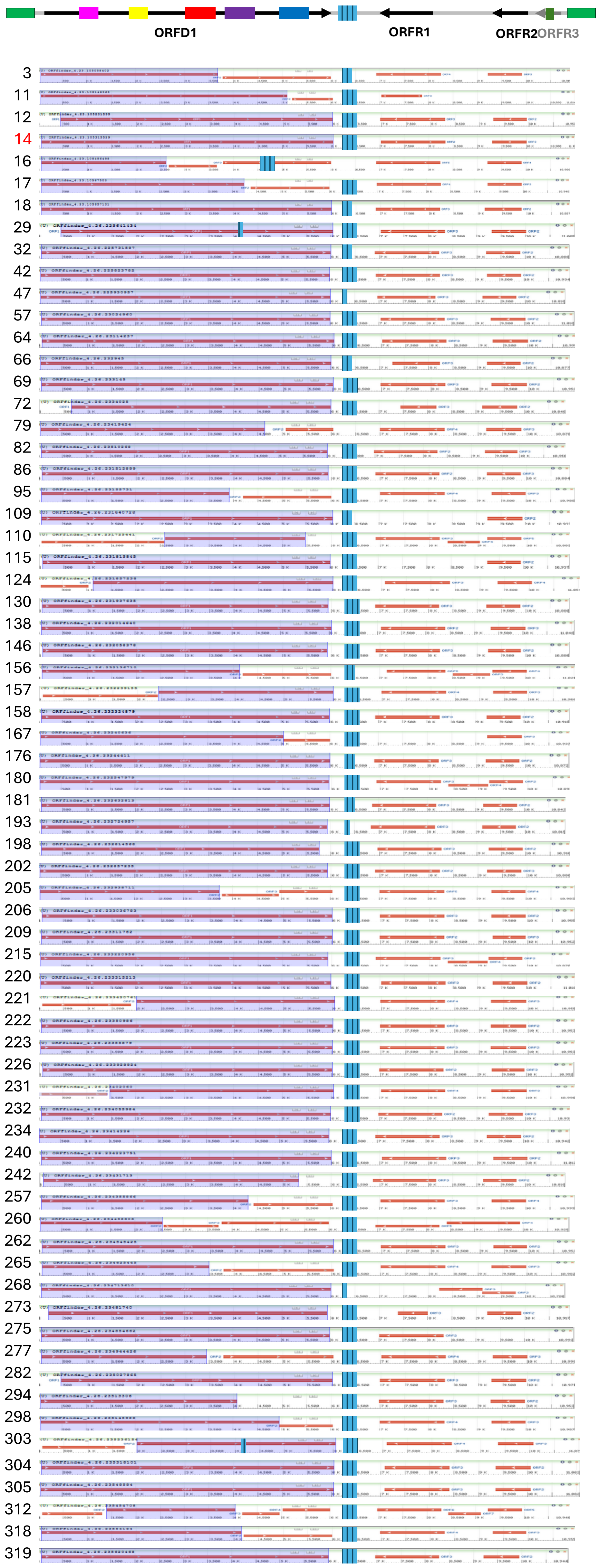

Sambucus nigra 4

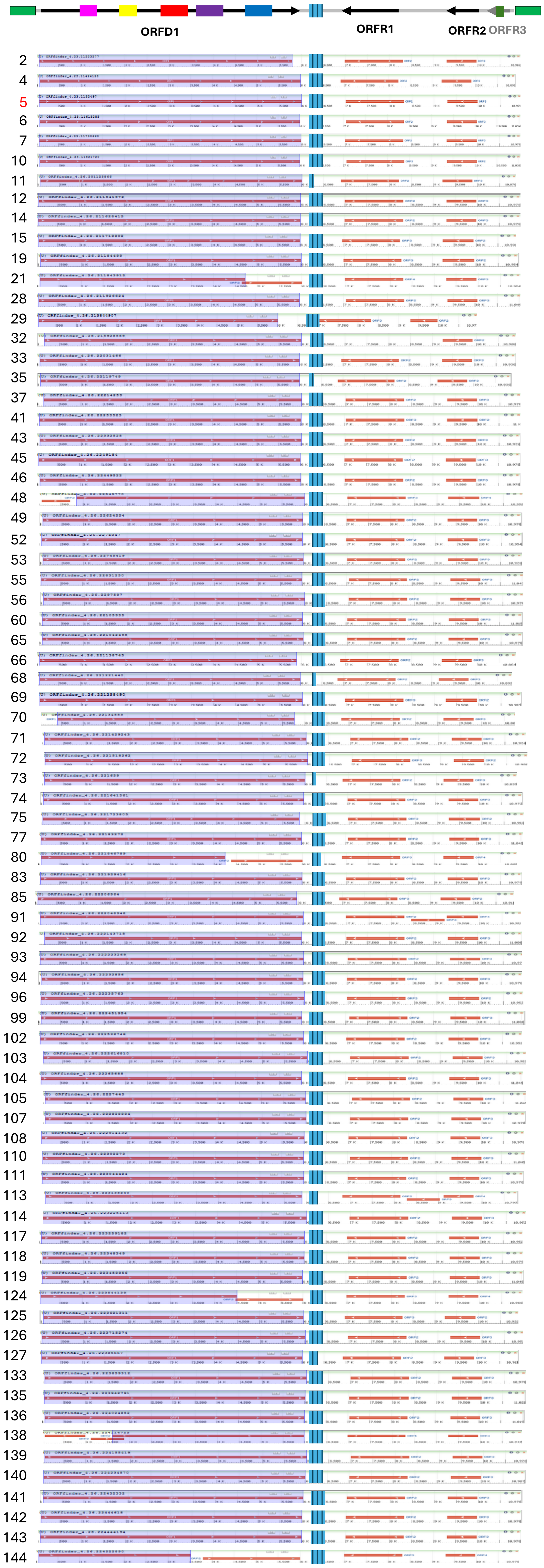

Sambucus nigra 6

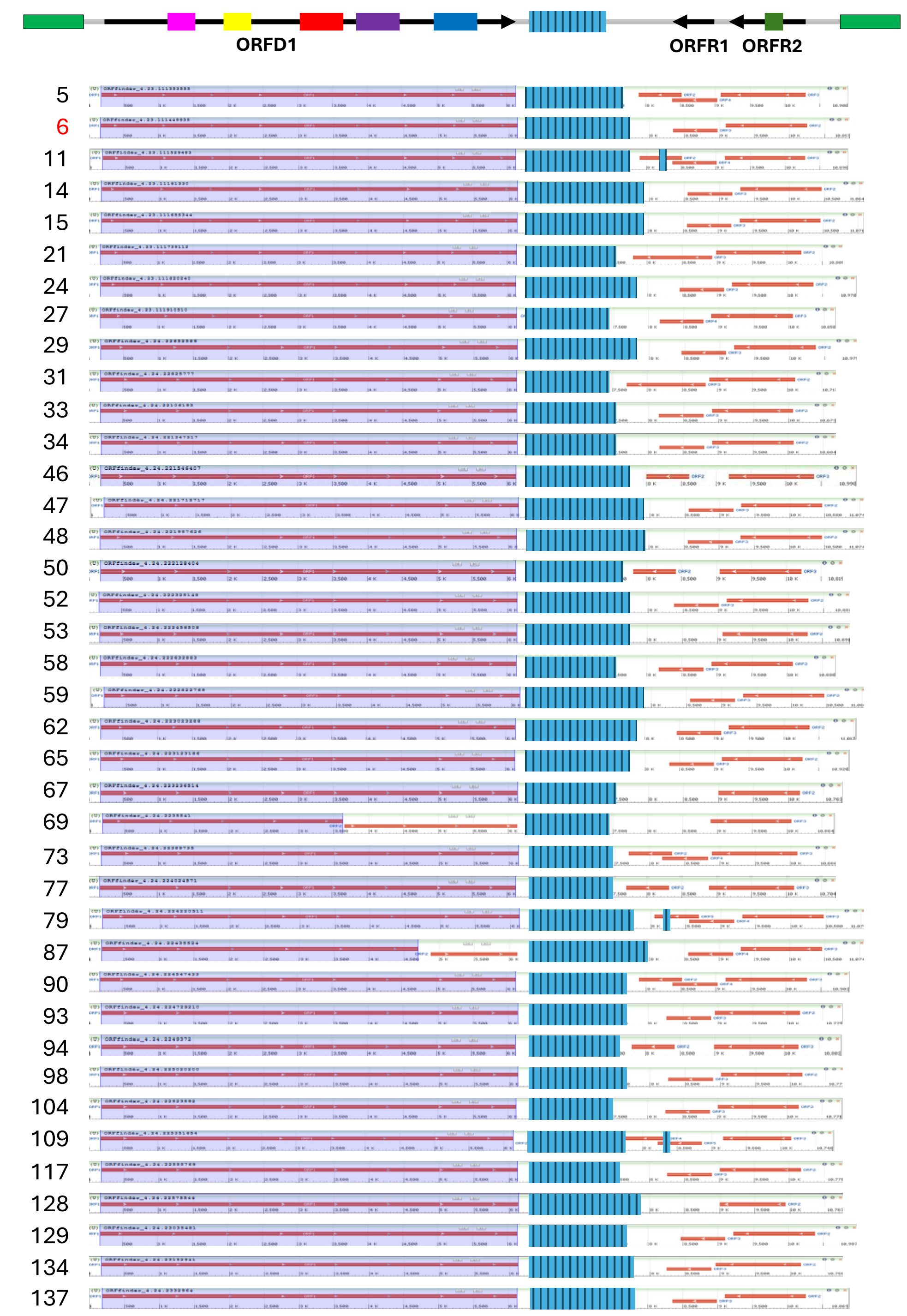

Secale cereale 1

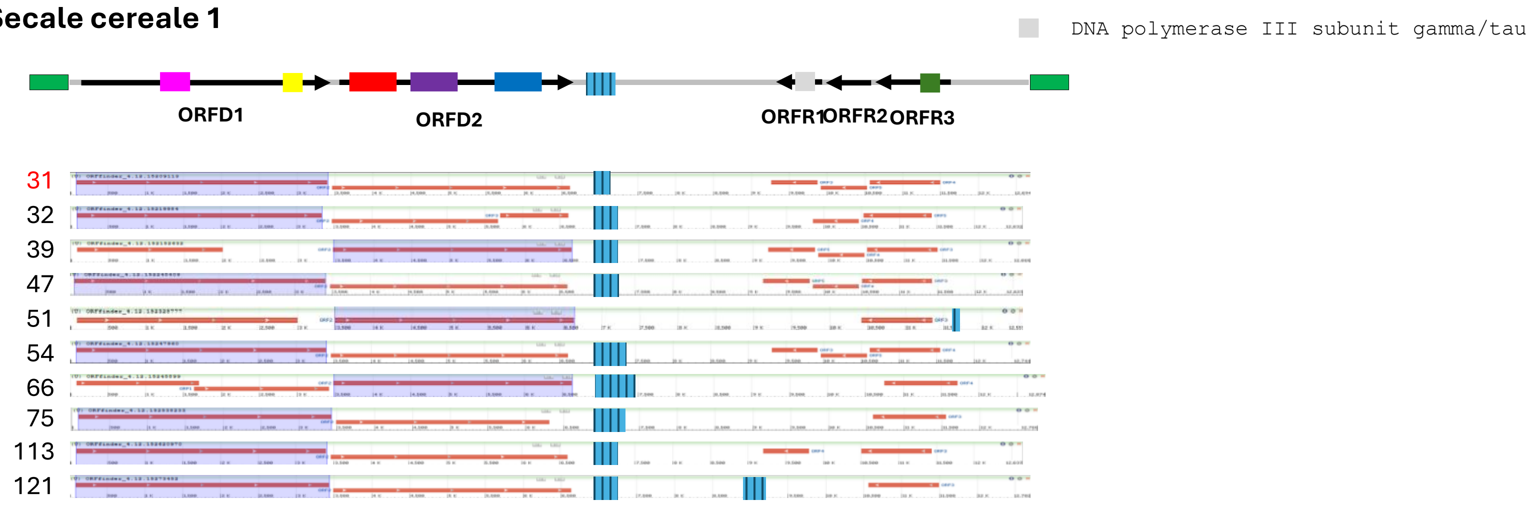

Secale cereale 2

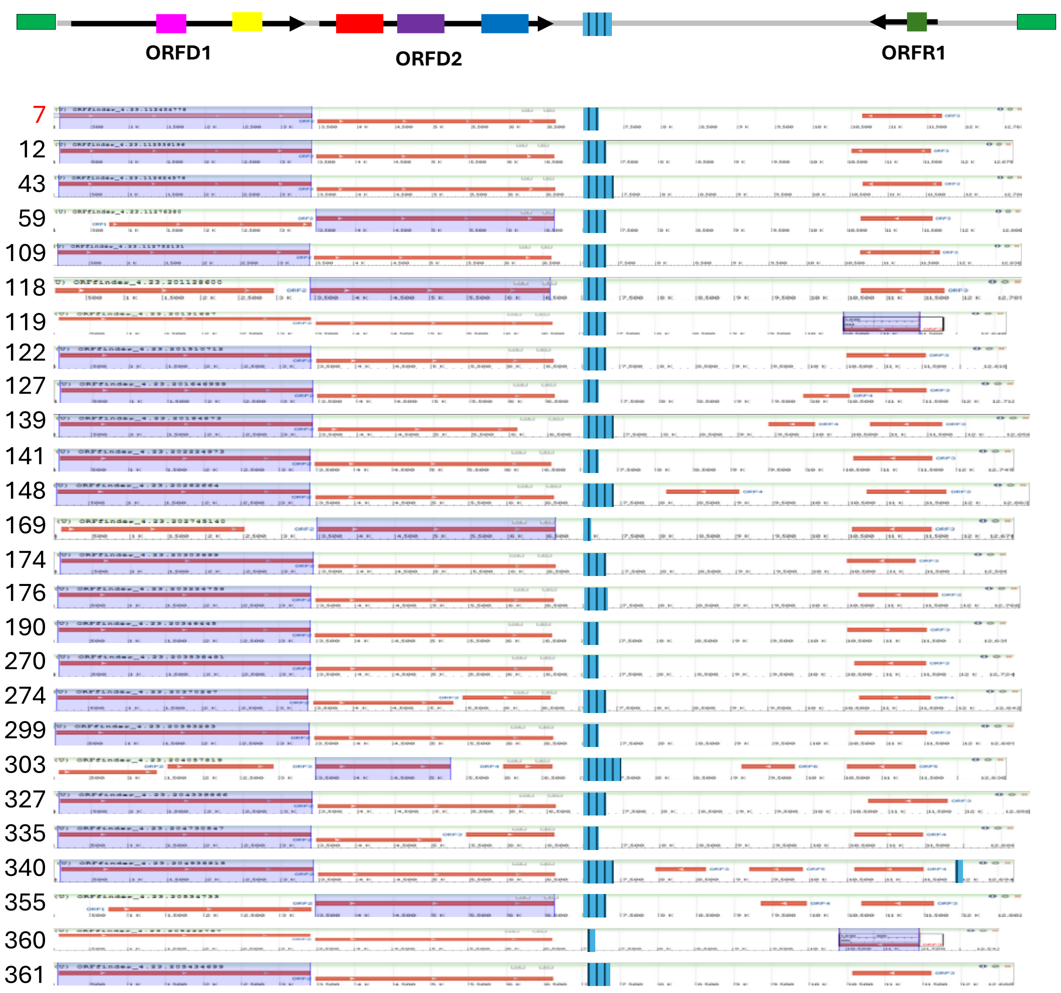

Secale cereale 5a

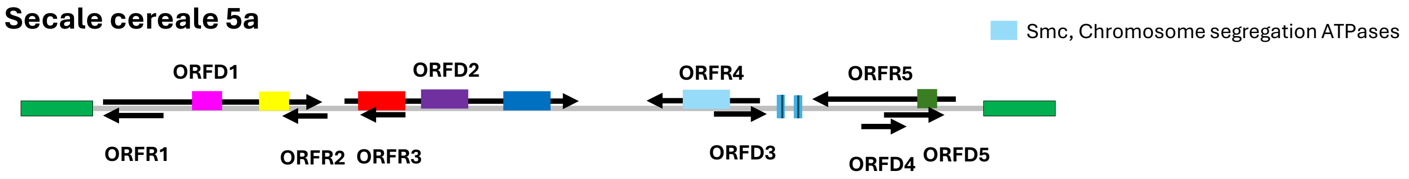

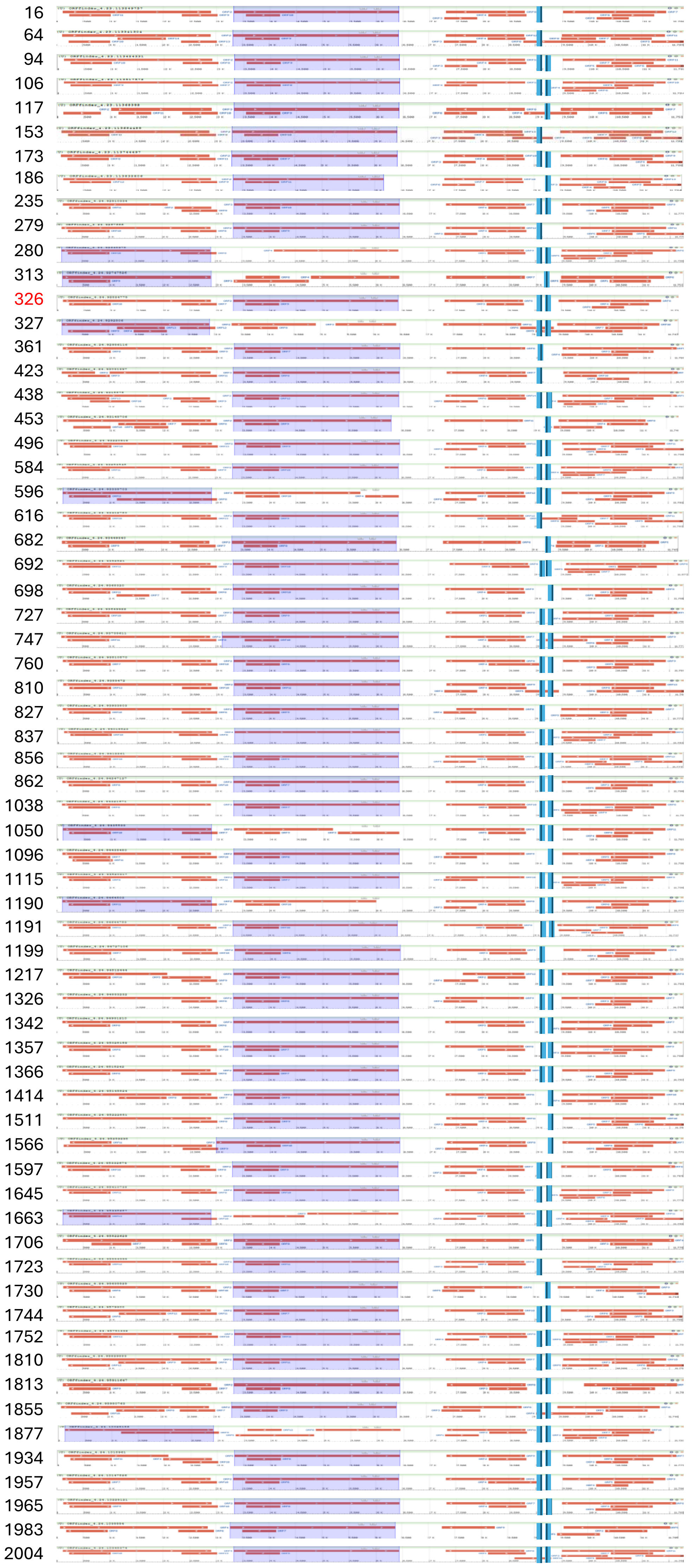

Secale cereale 5b

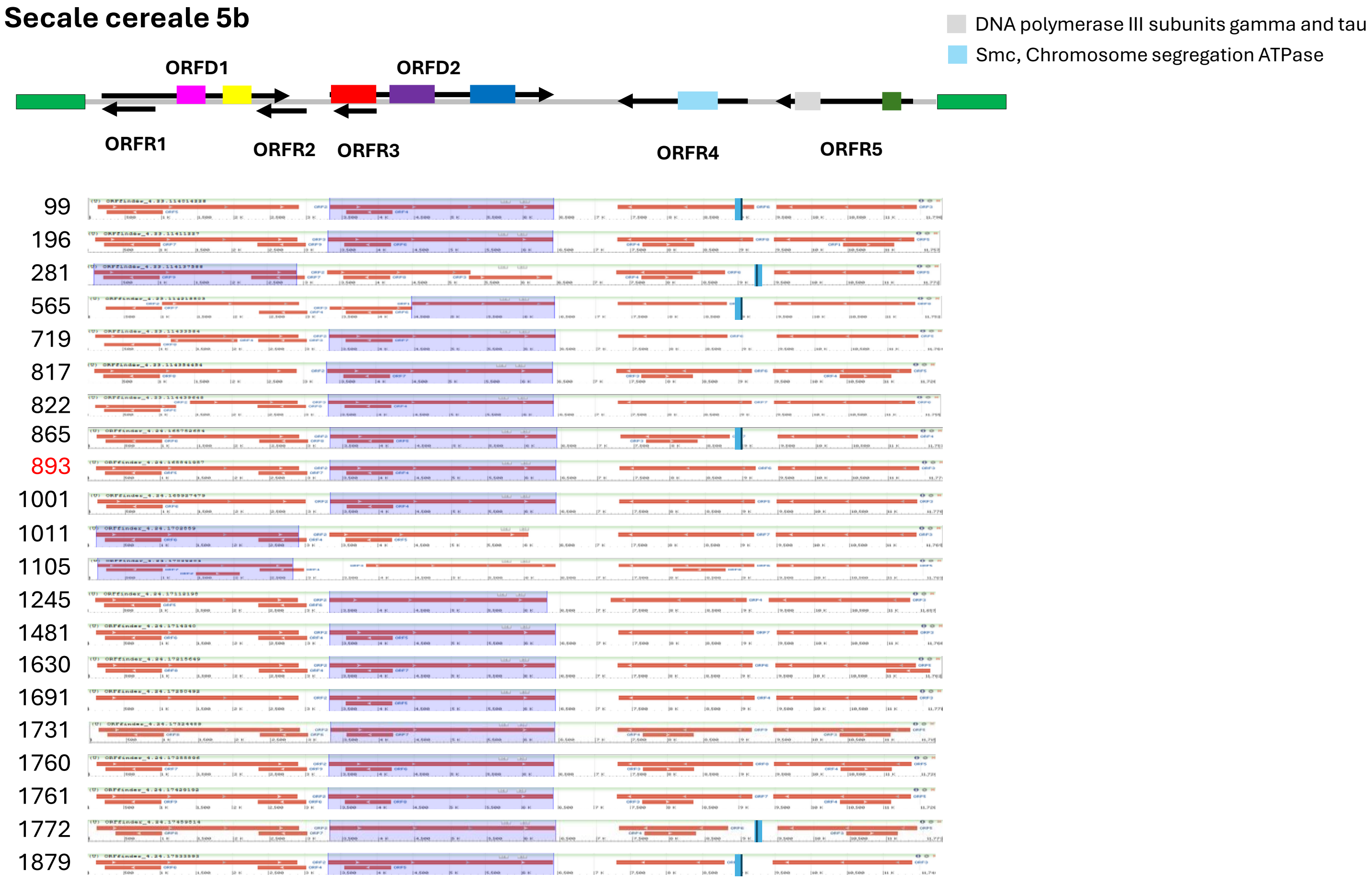

Spinacia oleracea 1

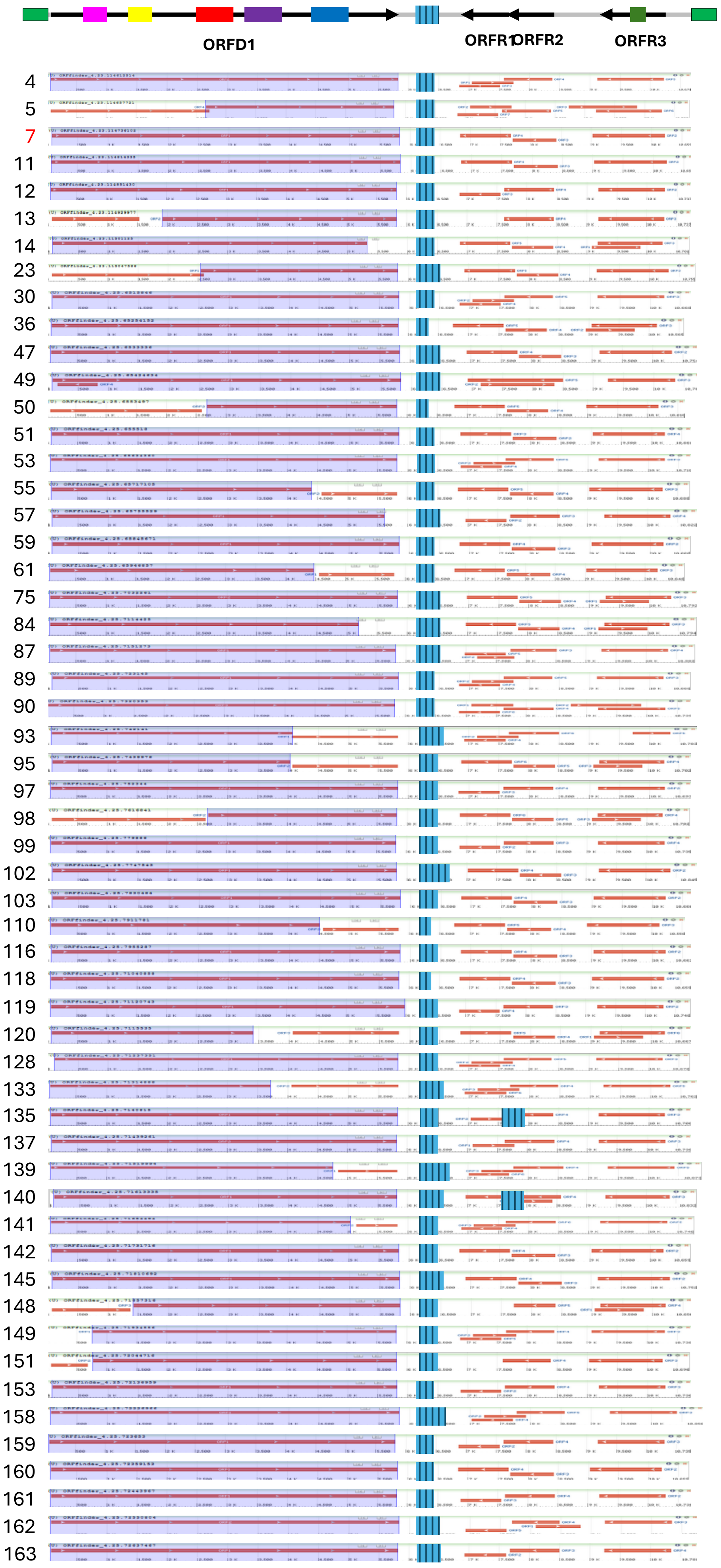

Triticum aestivum 10

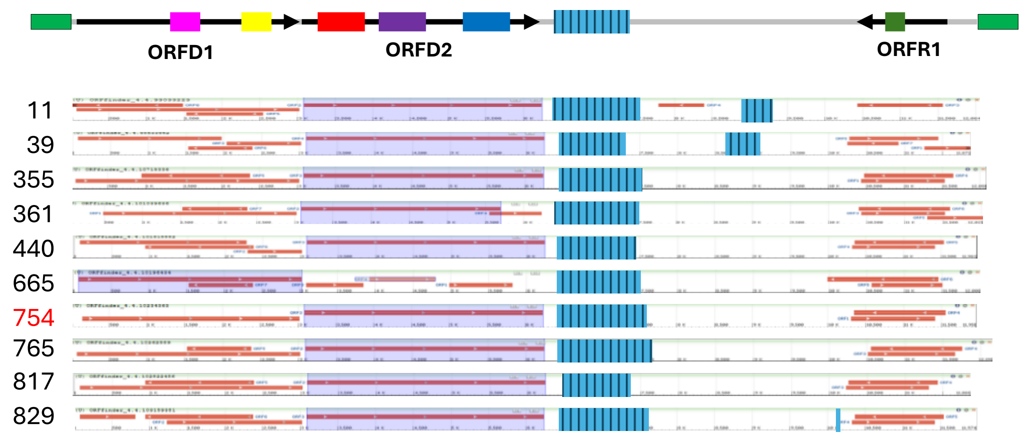

Zea mays 1

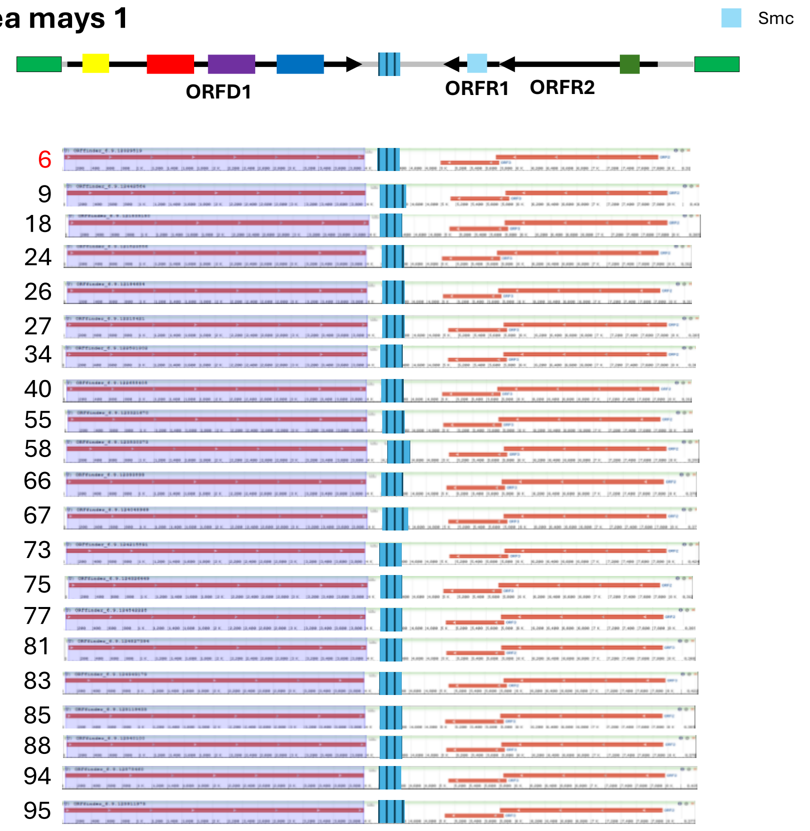
Smc
ORFD1
ORFR1
ORFR2
6
9
18
24
26
27
34
40
55
58
66
67
73
75
77
81
83
85
88
94
95

Zea mays 2

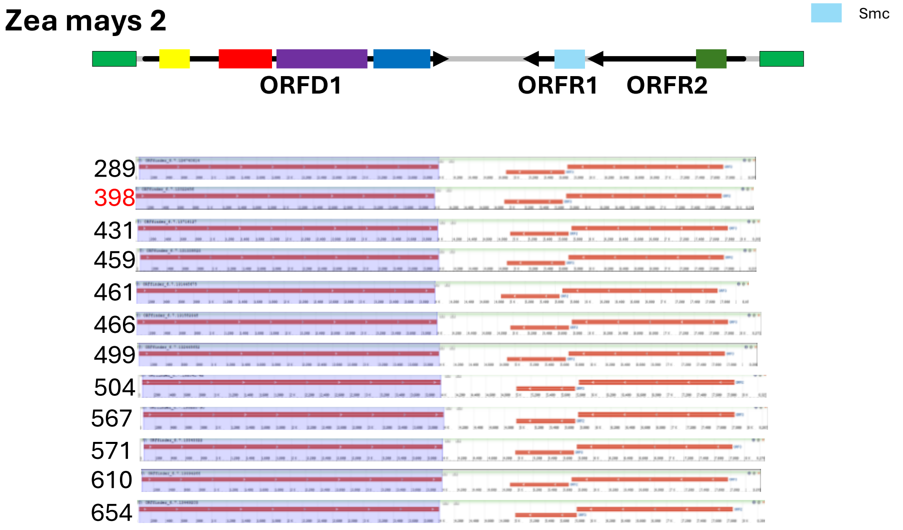
Smc
ORFD1
ORFR1
ORFR2
289
398
431
459
461
466
499
504
567
571
610
654
