# Supplementary material for: Retand LTR-retrotransposons in plants: a long way from pol to 3’LTR
Source: Mob DNA. 2025 Apr 2;16:15. doi: 10.1186/s13100-025-00354-z (PMC11963269; doi:10.1186/s13100-025-00354-z)
Supplement: Supplementary file 6 — Additional file 6. DNA sequences of the PRARE elements identified in this study. LTRs are in red. Flanking sequences are also included. [file 13100_2025_354_MOESM6_ESM.docx]

>Aegilops umbellulata 1-014;

GGACCCAATCATCGCCAAGAGCAACATGAGGAAGTGAACCAACTTCGCCAAGAGCGCCATGACAATGTCAATCCTCAACGACATGGACGTCATCATCCCCAACACAATGAAGAGCAACGATATG

TGTTGGGGATGTTGCTATCCGGGGTAAACCGGCCTGCAGGGCCCGGGTTACCTCCGTCAGCAGACCCGAGATGTTAAAAACCCAAGGAGAGATAGAAGGGCCTAAGGCCCATAATCGGTTTAAAGGTTTATGGTGCAAACCGGCCTGATGTAAACTTGTATTGTAAGATAGGAATAGAGAGACCGACCCGGGCACGTTTATAAGCCGGAGATCGAGACTTTGCAGACCGACGGGCATCACCCGTGTATATAAGGGGACGACCCGACGGCGGTTTGGAGAGAGAAAAAAGAAAACTCGAGGTTTAGGCGAAGCTTATCGCTCCCTAAGCCCTCGAAACCCATCAATCCCATCACAACTAGACGTAGGCTTTTACCTTCATCGAAGGGGCCGAACTAGTATAAAAACCGTGTCCTTGTGTCCACTTTAACCCCTTCAAGCTAACCCGTTGCGATGGCTCCACGACTAAGTCCTCTTACTAGGACATCTGCCGTGACAAATCCACGACA

GTTGGCGCCCACCGTGGGGCTATCGCGAGATGGTTTCAGGTTCTTGGAGGGCCGCTTTGAAGGACTCGAGGGTTACGCTGTGGGACGGATGACCAAGAGCCGCCGCGGAAAACTCTACATCGACAGCACAGGCTGGGGCCCCGACGCCGGTTCGATCGAGTACGGGTACCGGGTCCCCTTTGGCGGCATCCATGTTTTCATCGGCAAGATCGGAGAACCGGGCCCCGAGTTAGGCGACTACGCCGACCTCATTGAGACGGCTCAGTGCACGAGATCGACCCTGGCTAAACCGATCAGAAAGCACGCCTTCGTGGGGGTCATCCGCGGAGGAAGCTACGAGGAAGGATCCGAATCTGGTGACGAAACCGCCATCTACTCCGGCGATGAAACGTCGACCGGGGAAACCGAATCTCTTTACCAGCTACAAGATGATCGGATTGAGGGCGGCTCCGATGGCAACAGTATTCCGGACCCCTCGGATCTGCCCTGTCGGGTTGGGATCTTCATGGCTGGAACACAGGCGGCGCCACGATCATCAACTGCAGCAGCAGCGATCTCCGGATCAGCAGCAGCAACGGCTGCGCGGGCAGGAGGCCCTACAGGTTCACCAGCCCAAGTTTTGTCAGACCTGTTTGACGCACTAGCAGCGCTCATGGAGGAAGCCAACCCGGTGGATCAGGAGGTTCACAAGGCTGAGATTGCCAAAGTGAGAGAGCAGATCACTCGGGCTAAAGCGGAGCTGGCAGCAGAAGAGATCAGGATGACGGCGGAGCGCGCCGCTTTAGATGCACAGGCTTACAGGCTCATGATGGACCAGAAGGCGTCTCAAGAGGTCCTGAAGAGAAAATCCCGGTCTCGGTTGCCCGCCGCCCTCGACGCTCGAAACCTCTTCAACACCCCGGGGGTAAACCGTGCGGAGGTGACGGCCGGCGCCCCGGTTCAGCACCGGATTATAAACCTGCCTCGTCATAACACTGATACGCCACCGGCCATGCCAACGCCGTCAGGTCATTATTCCAACCCGATGGATAACCTCGTCGCGGCGGCGGCTCGGTTGGAAGCCATTCCGATCGAAGGAGATTCACCGCAGGATGAAGAAACACGCCGGGTCAAGGAGCTCCTTAGGACCGCGTTGGCCCAGCAAGAAGCATATTCGCAAAGCCGAGATCGGATTCACTCCACCCCTCGCCCCAGCGGGAGCTATAGCAGGCGCGTGGAGGAGCCGGCCGTTTCAAGTAATGCCAGGCGTGAGGCGCTCCGCGGCAACAATCCGGTGGGCGTTGACAATGCTCATGAGAGTGTGGATCGCACCCGTGCACGGGAGGCCGGATTGGCAGCGCAGCATCAGGCTCATCAGATTACACCTATCTTCCCAACAGCTGCGGCTGAACCGGGGGTGGTTTCCAGTTCTTTGGGAGTACCGTGCCTTGTTCCCGCCTTGCGCAACGTACGCCTGCCCAAAGACTTCAAAGGCCCCCGCAAGGTACCAAACTACACGGCAGATCAGCCTTCGGAGACATGGGTGGAGAGCTATGAGATGGCTATGGAGATGCTGGATGTGGATGACACGGCGTGTGCCAAGTATTTTACTATGATGTTAGAAGGAACGGCCCGGACTTGGCTAAAGAGCTTACCGCCTAATTCAATTAGTTCATGGGCCCAATTGCGCGCCCGGTTTATCAAGAATTTCAAGGACACGTGCAAACAGCCAATGTCGATAGTTGACCTAGCAGCCTGCGTTCAGCAAGAAGGGGAATCAACTACCCATTGGGTACGGCGGGTATCAGAGGTTTTGCACTCGTCGGATTGCATCAACGCAGACACCGCTGTAGTACTGTTGGAAAGTAACTGTCGGTTTGGCCCTTTGAAGCTAAAGCTGGGCCGGATGAAGCGTGACTGTACAGATATAGGAACGCTCATGACCGCTTTAGTGAAGTACGCGGATTCTGACAGTACCAAGGATCCTGACTCTGGTGATGACAAGGCCAGGAAGGGAAGAAAGAACAGCAACACCAAAGGTCAGCAGCATCGGCCGACAGGTAATGGAGGCGGAGGTAAGCGTAAACCGGACGGGAGCATGGACTTTGTAGCCAACACCAGTGCGCAGAATAAGGGCCAGCGGCGCAAGGGGAAACAGCCAAGTTTCCGCACTGATCCTGGTCCTAATCCGGAGCGTTTGAACTTTTTCTTAAACCAGCCCTGTCCGAAGCACGGAACAAAGGAGGAACCAGCAACCCATCTTTGGAAGGATTGTTATATCATGAAGGAGTTCAAAAGCTCAAACACTTTCCAAAATGATCGCAGCTCTGGCGGCGGTTCAGGTTCCGGCCCAGGATTCCAGGGTAATCCGGGCGGACAGGTCAGCCAAAATAATCAGAATAATCAAAGTAATCAAAGTGGTTATCAGAGCAACCCGAAACAGTTAAGTGGTGGACAGTATCATGTCTTTACTACGAGCTTGGATAAACGAGATAGGAAGCTTCAGAGGCGGGCAGTCAGTGCCGTCGAACCGGCCACGCCTCACTATCTGCGTTGGTCGGAACAGCCTATCATATGGAGCCGAGAAGATCATCCTCCCAGGGTTTATAATCCGGGTCAGTTAGCATTGGTGGTGGCGCCTCAGGTGGGAGGCTATAAGCTCACCAAGGTGCTCATGGACGGAGGGAGCAGCATTAACATCTTGTATTACAAGACCTTCCGTCGTATGGGACTGGTAGATAAGGATCTCAAACCGACCCATACAGTGTTCCACGGGGTGGTACCTGGCAAGTCTGCATATCCGGTTGGTAAGATAGCCCTTGAAGTGGTATTTGGCGATAGTCACGATTCCAGATCAGAGACGCTGACGTTCGAAGTGGTTAAAATCCAAAGTCCGTACCACGCTTTGTTCGGACGGCCAGCTTATGCAAAATTTATGGCACGGCCCTGCTACGTGTATCTGCAACTTAAGATGCCAGGTTACAAGGGCACTATAACGGTTCATGGGAGCCGTCGAATCGCTTTGGAATGCGAGGAAGGAGATGCGACTTATGCAGAATCGGTTTGTGCTACGGAGGAGCTAAAGCAGTACAAAGACAGTGTTGATCCGGAGGATATGACTTCATTAAAAAAGCCAACTACGGACCACGATCCGGCCTTGAAGTTCAAGTCAGCAGCTGAAACTAAGCTTGTTGACTTCGTACCTGGCGATTCATCCAAGCAGTTCAGCATCAGTGCAAACTTGGATCCAAAATAGGAAAGCGCGCTCATCGAGTTCATCCGTGAGAATCGGGACATCTTTGCATGGAAACCATCTGACATGCCTGGTGTACCGAGGCAACTCGCTGAGCACACCCTTAATGTGGATCCTAAGTACAAGCCGGTAAAGCAGTTCTTACGGCGGTTTAACGAAGAAAGACGTAAAGCGATTGGTGAAGAAGTGGCCAGGCTCCTGGCAGCTAGGTTTATCATTGAGGTTTTTCATCCCGAATGGCTCGCTAATCCGGTGCTAGTTCTTAAGAAAAACGGCACCTGGCGCATGTGTGTGGACTACACAGACTTGAATAAAGCTTGCCCAGCAGATCCTTTTGCTCTCCCCCGGATTGATCAGATCATTGATGCAACAGCAGGTTGCGAGCGTTTAAGTTTTTTGGATGCCTATTCGGGTTATCATCAGATCAAGATGGCAGTTAAGGACCAGGAGAAGACAGCGTTCATTACTCCCTTTGGAGCCTTCTGCTATGTGTCTATGCCCTTTGGGCTTAAGAGTGCGCAGGCTACTTATCAACGATGTGTGCAAAATTGCCTCCACGAGCAGATTGGTCGTAATGTGCACGCTTATGTGGATGATATTGTGGTCAAATCACGAAAGAAGGAGACCCTGGTTGACGATTTGAAGGAGACTTTCGATAACTTGAGAGTGTACCAAATGATGCTCAACCCGGCTAAATGTGTTTTTGGTGTACCTGCAGGCAAGCTGTTGGGATTTTTAGTGTCCCATAGAGGAATTGAGGCCAATCCAGAAAAGATCACAGCCATCACCTCCCTGGCCAAACCGAAATGCATCAATGATGTTCAGCGCATGGCCGGGCGGATTGCAGCGTTAAGCCGGTTTATCAGTCGCCTTGGTGAAAAGGCGATCCCTTTGTATCAAATGCTCAAGAAGACGGATCAGTTTGTCTGGAGTCCGGAGGCTGATAAAGCGTTTGAGGACTTGAAGCGACAACTAGTCAATCCGCCAGTGTTGGCAGCCCCTGTAGATAAAGAGCCACTCCTGTTATATGTTGCAGCGAATGCCAGAGCAGTCAGTGTGGCGATGGTGGTAGAACGAAAGGAGGCTGGAAAGGAGCATCCGGTTCAGCGACCGGTTTACTATATCAGTGAAGTACTTATCGAGTCCAAACAAAGGTATCCACATTGGCAGAAGCTGGTATATGGTGTTTTTATGGCAAGCCGGAAGCTGAGGCAATATTTTCAAGGACACCCGGTCACGGTGGTCAGTTCTGCTCCTTTGGGTGACATTATACAGAACCGAGAGGCAACCGGTCGGATTGCCAAGTGGGCTATCGAGCTCGGGCCGCACGACATAAGGTACGTACCCCGGACGGCCATAAAGTCACAGGCACTTGTCGATTTCATAAACGATTGGACAGAGTTACAAGCGCCTGAGGAGAAGCCAGATAACACCTATTGGACCGTTCATTTCGACGGGTCAAGAAAATTGGAAGGCTCGGGGGCTGGAGTCGTTTTAACTTCCCCACGAGGAGATAAGTTTTGTTACGTCCTCCGTTTAATGTTCCCCTGTACAAATAATGCGGCTGAGTATGAGGCTTTACTTCATGGTCTTCGGGTGGCTAAAGAGATGAATCTGAGCAGAGTTAGGTGTTTTGGTGATTCGGATCTGGTCGCTCAGCAGGTATCTGGCACCTGGGATTCCAAGGACCCGCTTATGGCTGCATACCGACGGGAAGTAGATATGGTGGCTGGGCATTTCAAAGGTTATCAAGTGGATCATGTGGACCGCAGAAAGAATGAGGCAGCGGACGCTTTAAGTCGCTTAGGCTCTCAGCGTAAACCGGTCCCGCCCAATGTGTTCCTGGATGTATTGTACCACCCATCGGTACAGCTGCCCGGTGAGCTGGAGTTGGCTGTTCCTGATCCGGAGGCTCAGTTAGTGGCGGCTCTTCACGCCACCCCGGATTGGACGCTCCCTTACCTGGCATATATGAACCGGGGTGAGTTGCCAGAAGATGAAAGCTTGGCTCGACAGATAGTACGACGGTCCAAGTCTATGACCATTTTCCAAGGAGAGTTACATCATCGCAGCGTGTCAGGAGCGCTGCAAAGGTGCATATCCCCGGAGGAGGGGTGCGAGATACTACGAGAAATACATGAAGGGGATTGCGGCCACCACGCCGGGTCAAAATCATTGGTGGCTAAAGCGTTCCGCCACGGTTTCTACTGGTTAACTGCTCATGCTGATGCGGAAGACCTGGTCCGGTTATGTGATGGTTGCCAGAAATTTTCCAGACGAGCGCACATACCGGCTCAGGAGTTGAGGATGATTCCAATCACTTGGCCGTTCGCGACTTGGGGGCTTGATATGGTTGGGCCTTTTAAGCGTTCCAAAGATAAGAAGACCCACCTACTGGTGGCGGTTGACAAATTTACAAAGTGGGTGGAGGCCGAACCTGTCAGTAAATGTGATGCTGCCACGGCGGTTCAGTTTATAAAGAAGGTGATCTTCCGGTTTGGTTTCCCACACAGCATCATCACAGATAATGGTACCAATCTATCCAAGGGGGCCATGAAAGAGTTTTGTGCACGGGAGCATATACGGCTTGATGTTTCTTCGGTAGCGCACCCACAGTCTAATGGTCAGGCAGAACGAGCGAACCAAGAGATCTTGAGAGGTATCAAACCCCGGCTTCTGGTCCCTTTGCAAAGGACGCCGGGTTGTTGGGTGGAGGAGCTACCGTCTGTATTATGGAGCATCAACACCACGCCTAACAGATCCACGGGGTTTACGCCGTTTTTTATGGTTTATGGAGCAGAGGCGGTTCTCCCAAGTGATATACGACATGACTCACCTCGCGTGGCAGCATATGTTGAAGCGGATAATGAGCAGGCACGGCAGAACGCTCTTGACTTGTTGGATGAGGAGCGTGACTTGGCAGCTGCCCGTTCAGCGATTTACCAGCAAGATCTTCGCCGTTACCACAGCCGTCGGGTTAGGACCAGAACCTTTCAGGAGGGGGATTTGGTGCTTCGGTTCATCCAGGATCGATCTGATCAGCATAAATTATCCCCGCCTTGGGAGGGACCTTTTGTGGTCAGCAAGAATCTGAATAATGGGTCATATTACCTGATTGATATTCGAGAGCGCAAGGATTCACGCACATCAGAGGAGGAGACCAGCAGGCCGTGGAACATAGCTCATCTTCGGCCGTATTATACATGAGCCCTGGGCTCTGCTTATGTACATATCATAACCATGTATATATTATGATTAATACAATAAACCGGAGCCTCGAGTAAAGCGGGGTCTCTGCTGTTCTTCACATCATGCGTGTTTACCCCTGAAGGTCGCTTCACAGAAGTTCAAATTATAGACTCCGGTTTAAGAGCCGGTCCAAGGGAAGATGTCTCCTACAAGGCTGCGAAGCTCTTAATATCCGGTTCAATATCCCGGTTCAAGGATAAGGCTTACTGTTCAAACATAGGCCGTATTCGAGCCAAAGAGAACATAGCTATTTAACATGGGGCTTCCTGTTCAAACATAGGTCGTATTCGAACCAAAGAGAACATAGCTATTCAAACATGGGGATTTCTGTTCAAACATAGGTCGTATTCGAACCAAAGAGAACACAGCTGTTCAAACATAGGTCGTGTTCGAACCAAAGAGAACATAGCTATCCCTATAAAGTCAGTTGGGGACCTAGTCGTCCTGAACTATAGCTACACCTCCGGGGGGCTTGGTCAGGTCTTGACGACGGTAATGCCTTCTGGCTGGCATATTTGCCACTGAATCAGTTGGGGACCTAATCGACTTGAACCGTAGATTACGCCTTATGGGAACTCGGTCATGTCTTGATGATGGTAATGCCTTCTGGTCGGCTTTTTAGCCATTGAGTCACTTGGGGGCTTGGTTGACGAACCAATGCAATGCCATTTGGCCGGCATATTTGCCAAGATTCAGTTAGGAACCTAGTCGACTTAAACCGTAGCTACACCTCTTGGGGGCACGGTTTTGTCTTGACGATGGTCACGCCTTCTGAACGGTTCATAGTAACCACTATTCGCATGCGTTTTACTATCGCGTTGCCTTTTCATATTTGGTTTTGTTTTCTGCCTTTATTGTGGTGTTTTTTGGTTCTTCGGAACGTCAAGTATCCTTAAACCGATTCAGCTGGCATAGCCTGGTCCGTTTTTTAACCCAGAGGCGAGTTGCCCGGTTTGGCAAGGGCGCAGCACGGTTCATAGGGCCGGAAGATCTTGTGTACTCAATAAAGAAAGGAAACAAAGTTGGAAAACGTCATTGGAAAGGAGCATCAGTACTCATGCGCGAGGGCACATTCAAATGCAGAGGTATCAAACTATCTTATTACAAGGCAAACGTAGTGCCTGAATATATTGTTCGTTGATGGATTAAGGCGTCACCAAGTAGGGTGAACCGGAGCGTTGATTACGCCTGTTCGCCACCTTGGTTCGAAGGCTGGGGATCGTCTCGCGCCGCTTCAGCTCCATCTTGGTTACCCATTGGCTGGAAATCAGCAGTGGTCCAGTCGATTCCCATTAGCGCTTGAAAGACGGCTTCTTCGTGAATCAGCGAAGACGGTTCAATGTCTGGGGCGTAAGTATGCTTACGGATTGGAGGAACAAGGTTTCCCGCTTTAGGGACCGGGGCAGATACTCGTTTGTTCTGGTTGTCATATTGGGCTTGGTAACGTGACAGATCTGCCTCCTCAGCCAATTGACAAGCTAGGGGGCGCACCGCCCGGTTTATCGCCCTCAAATCATCTTCACCAAAGTCTGAACCGTCTTCCTTCAAGCTGGGGTATCCTTGGGCCGCTTCGACAGGATCGAAATCTGGTACCCACGCTTTGGCCCGGATCAAGGCAGTGATAGCTCCAGTTCGAGCAGCTGATCTTTTCAACTCTTCAATCCGGGCCGGCAGCATGGACAGCCTCTTTAATGTGTCTTGAATCAGAGCCGGCGCAGGGTTGTTGTGCGATGCAGTGGTGATAGCTCTTTGTGCTCCGGTGTATAACTGTTCAATCAGAGTATAAGCAGCTTTGAGTTTCATCCGCACATCAGAACCCAAGTGTCCAATACGTGAGCCTGCAGTGTTTCAAGGTGGATTACAAACCGGACATAGGGTGTAGCAAACAAGGTACAAATTGGAAGAAGGGCATTGAAAGGTTTACCAAAGACGGCAGATGTCATGGAGTGTATTTGTCGCTTCACGGTGGATAACTCATCAACCACCGGTTTAAGAGCAGCTTCGGCATTTTCAGCCCTCTTCGTCAAAAGAGCTCTTTCAGTAGCCCAATCGGCTTGCTCCTTTTTGCGGCTTGCTTTCAGCTGCTCCATAACAGTTAAGGCTTTGATCAATTCCTCCTTTGCTTTTTCAGTTTCAATCTGCTGGGCTTTTAAAGCTTCCTGAAGGTCCGAGACTTGGTTCTCTTTGGTCTTCAAATCCCCCTGTAGCAGGTCATCAACAGTGTTCAGAAAATATTGCACGGTTTGGAAGTATCAACCACACAAGCAAGTTATGTGCTCGATACTTGGGGGCTAATGCATACTTGATTTTAACGCAAGTTTTCCATTACAAAGAGTCCCAAGATTAATACAAGTATTTAAACTTGGCACTTGGGGGCTAATGGTTATCTGATGATGTTTTCTGATTATGATCGATGTTGAAACCTGGGGTTTTCTAAACCAGACCTTAATTGTCAAAAGCAGCCTGGTCCGGTTTACTATCTCGGTTTGGAAATATTGATAAGCTAAGTTGCAATAGAATGAAGCACAAGGGAGTTACCTCAAGTTTCTCCTTCATCATCTTGATCATGGCAGCCTCATAATCCCGACTGGTGTACAGCCGGTTCAGATACCCAGAATAAAGATCCGGGGCGCTTAAAGAGGCGAACGTAGACAAGTCAAGGTCCCATTTGCCCTTTATGGCCGAGATTTCGTCTTTGGCAGTATGCTTGGTCAAAGTAGCAGTGTTCCCCGGTTCAGAGCGGCCAATACCGGTAATAACGACATCATCATCCTTGGAGTCGCTGCTCTGCAGTGGCCCTGTCGGTTTATCAGCAGTTGCAGAAGGGGGGATGGATTCCTCAACCCGAACAGGACTGGCAGGCGGATCAGTACGGTTAGCAGGTTCGGTAGTCCTTTCTTCAGCGCAGATATCATCTTGCGGAGATGGATAGTTCTGAGTATCTTCACAACCCAGAGCGTCAGCACCAGGAGTTTTCTCCGGTTCAGGAGCGGCAGCTCCTTCAGCCGGGTTATCCAGACGAGCCTTCTTGCTCGGTCTAGGCTTAGCCCTGAAAAAAGCCCAAAGATTGAAAAAACGGTATAAGATGGATTAAGAGACCCATACAGAGATATGTTGTGATCGGTCCAACTTACCCAATAACTGTCTTCAACGGTGGCAGTTGAGTGGCTGAAGATTCACCGGAAGAGGAATGGGAAGTGACCTGATAATCGGAGTCAGATGGATTTAGAGGCTGACGAGTTAAACCGGCTTTAGGAGGAAAGCTAGCGCATAAATCGGAGACCTCTGAGCGGCGTTTCCGAACCGGGCTGTTCGGTAAACCGGCGGAGGTGACTACCTGGCCGCTATGTCGGGTGGTGCGTCGAGCTTCATGTTGCTGGGTCTTCACAAGAAATTTGGGATCCAAGTAAGCAAGAGGGTGAGAAAATTTAATTCTCCGGTTTGCTTGGCGAGGTTTTTTCAAAGGCAAAGGAACGGCATCAGAAGAAAGAATAGTTACCTCTGGGCCTTCGGCATGGCTCACTTCAGCGTCGTCCTGACAAATATCATCATCAATAAGACGTATAAAAAGCAAACCAACTGAATCGAGGTCTACCCCGAGGTCCGGATTACCCACATCATCCTCCTCTAAATCCGGAGTAGAGGAAGCAGTGTTCCTTTTCCTCTGAGCAGGCTTCTTCACCCTCGTCTTGGGTCGGATTACCTTTTCGGTCGGCTCTTGAAGCTTCTTGCTCCAAAACGGATCATCTCCCTGTTTCGAAAAGGACTGGAAGTTAACAACATATATATAAGGAGATCAAAGATTCTATAAACAGAGGAGTTACTTACCGCAGGCGGTTTGTTTGTGGCACAAAATGGAAGCAGGCCGGTTCGAGCGCAGACATGTTCCGGTTCGTTCAGCATCTTCTTCACAGCTTCAGCGACTTCGTCCTCTGTCAGCTGAATGTTGCAGTGCCTCAAAGGATCATCAACCCGACCCGTGTATTGGCACATTAAACCGGAGCGGATGCTAAGGGGCAGGATGCTCCATGAAATCCAGCAACGGGCGAGATCTACCCCCGTTAAACCGTTAGCCATGAAGGCTCTGAGCTTGGACAGCTGAGGGGCATACTTACTCCTCTCTTTGGCAGTTAGCCGTGGGGGGAAGGGGTGAGTATTGCTAAGCCTCTCTGGACGGAAACCGGGCAGTGGATTTTCTTCAGCAGGGGAAGTATCCTTGCAATAGAACCAAGTGCAATTCCATTCTTGCGGATGACTATGCAACTTGGCGTGAGGATAAGTCACCTCTTTCCTCTTCTGAATCGCCATACCACCCAGTTCCATATTAGGACCATCGGTGAATTCAGTACGTCGGTTCAGATGGAAAAAGTCCCGGAACAGCTCTACAGTGGGTTCCTCTTGAAAGTACGCCTCGCAAAGTACTTGAAAGTGGCAGATGTTCGTGACTGAATTTGGACCAACATCTTGTGGGCGCAACTTAAAATTAGCCAAGACGTCCCGATAAAATTTTGAACCGGGTGGGTTAAACCCCCGGGCCAGGTGATCCGCAAACACCACCACTTCTCCCTCTTGTGGGGTAGGAGGACATTCGTCTCCTGGAACCCTCCAATGGATGGTATTTTTGCTACTCAAAGCACCAGTCAGAACTAAGTTGTTCAGTTGGGTCTCAGTGATGCAAGAGGGAACCCAGTTGCATTCGTACACCTGTTTTGCCATGAGGAAATCTACAAGATAAATGTATTCCGGTTTGAGAACAAGGGTGATTATAAACCGAAGATGGTCTTATATAGTCAAGTTATCCACAATGATATCAACCAGCGGTTCATCAAGGGGACTAATGGATGTGTGGGTTGTTTTGTTCACTTTGCCAAGAGTTAAACCGACCAGATCTAACAGCTGCGAGTATGAAAGCAGAAACAGATCCCGTACGGATTCTTTCAAAGCAATGGATCTACAGCTAGGGCGAAAAAGAAATAAAAACAGGTAATAAGAAGTCGGGCTCACAGCAGGACAACATAAGTTCGACAAGCGGGGACAAGAGTAAGATCTAAGGAAATGAGTGCAGATTTATAGCGACTGCTAAGAGGAACTACAAATCCAGATACAGGTAGCATCATCTGTTCGCAAAGGCTGCTATGGGGATGAGCAAGAAACAGTAGCAGAACGATGAACCCTAGAACAGATCTAAAGCAACAAGAACAGGGGCCTTACCGGGGTCCAAACAAATGCGGAAAGTCGCCGCGGTGCTCTGAACCGATCAGGTTGATGCAGCGGCCGGAGTTGGTGCAGTGGCTGACGGCGGCGGCGGAGCTTTGAGGTCTGGGTCGCGAGGAAGACGAAGAAGGGGAGGAAAGGAAGATGGCCTTCAAGTCCTATTTATAAGGCGCGGCGCGCGTGTCAGGCGCGCGAATCCAGGGGCCGGAGATTTCGGAAGCCCAGCCGTCGCCCTGATTCTTGCGGGTTAGCGAAAGGAGACGGTAGCTTGCGCTGACGTCATATCGGTTTACCACAGCAAGAGGAGACGAAGCAACAACGGTTTAACAAGTCACTGGAAGACATTTGAAGACAGAAATTCTTATTAAGGATTGACATGAACCTGTTCAAATCAATCTGGGGCCTAA

TGTTGGGGATGTTGCTATCCGGGGTAAACCGGCCTGCAGGGCCCGGGTTACCTCCGTCAGCAGACCCGAGATGTTAAAAACCCAAGGAGAGATAGAAGGGCCTAAGGCCCATAATCGGTTTAAAGGTTTATGGTGCAAACCGGCCTGATGTAAACTTGTATTGTAAGATAGGAATAGAGAGACCGACCCGGGCACGTTTATAAGCCGGAGATCGAGACTTTGCAGACCGACGGGCGTCACCCGTGTATATAAGGGGACGACCCGACGGCGGTTTGGAGAGAGAAAAAAGAAAACTCGAGGTTTAGGCGAAGCTTATCGCTCCCTAAGCCCTCGAAACCCATCAATCCCATCACAACTAGACGTAGGCTTTTACCTTCATCGAAGGGGCCGAACTAGTATAAAAACCGTGTCCTTGTGTCCACTTTAACCCCTTCAAGCTAACCCGTTGCGATGGCTCCACGACTAAGTCCTCTTACTAGGACATCTGCCGTGACAAATCCACGACA

ATATGGCAAGCTAAAGTTCACCATGCCCAAGTTCAATGGAAGCAATGACCCCGAAGAGTACATCTCATGGGCATTGAAGGTTGACAAAATCTTCCGCTTGCACAACTACGAAGAAGAGAAGAAG

>Aegilops umbellulata 1-029;

AAGAAGCAACCTTGTCTATTCCATCACGGCCGTCGCCCACGAAGGACTTGCCTCACTAGCGGTAGATCTTCACGAAGTAGGCGATCTCCTTGCCCTTACAAACTCCTTG

TGTTGGGGATGTTGCTATCTGGGGTAAACCGGCCTGCAGGGCCCGGGTTACCGCCGTCAACAGACCCGAGATGTTAAAGCCCAAGGAGAAATAGGAAGGCCCAAGGCCTATAACCGGTTTAAAGGTTTATGGTGTAAACCGGCCTGGGGCCAACTTGTATTGTAAGATAGGAATAGAGAGACCGACCCGGGCACGTTTATAAGCCGGAGGTTGGGACTTTGCAGACCGACGGGTGTCACCCGTGTATATAAGGGGACGACCCGACGGCGGTTTGGGACAGAGAAGAAGAACTCGAGGTTTAGGCGAAGCTTATCGCTCCTTAAGCCCTCGAAACCCATCAATCCCATCACAACTAGACGTAGGCTTTTACCTTCATCGAAGGGGCCGAACTAGTATAAAAACCGTGTCCTTGTGTCCACTTTAACCCCTTCAAGCTAACCCGTTGCGATGGCTCCACGACTAAGTCCTCTCACTAGGACATCTGCCGTGACAAATCCACGACA

GTTGGCGCCCACCATGGGGCCATCGCGAGATGGTTTCAAGTTTTTGGAGGGCCGCTTTGAAGGACTCGAGGGTTACGCTGTGGGACGGATGACCAAGAGCCGCCGCGGAAAACTCTACATCGACAGCACAGGCTGGGGCCCCGACGCCGGCTCGATCGAGTACGGGTACCGGGTCCCCTTTGGCGGCATCCATGTTTTCATCGGCAAGATTGGAGAACCGGGCCCCGAGCTAGGCGTCTGCGCCGACCTCGTCGAGACAGCTCAGTGCACGAGATCGACCCTAGCTAAACCGATCAGAAAGCACGCCTTCGTGGGAGTCATCCACGGAGGAAGCTACGAGGAAGGATCCGAATCTGGTGACGAAACCGCCATCTACTCCGGCGATGAAACGTCGACCGGGGAAACCGAATCTCTTTACCAGCTAGAAGATGATCGGATTGAGGGCGGCTCCGATGGCAACAGTATTCCGGACCCCTCGGATCTGCCCTATCGGGTTGGGATCTTCATGGCTGGAACGCAGGCGGCGCCACGATCATCAACTGCAGCAGCAGCGATCTCCGGATCGGCAGCGGCAACGGCTGCACGGGCAGGAGGCCCTACGGGTTCGCCAGCCCAAGTTTTGTCAGACCTATTTGACGCACTAGCAGCGCTCATGGCGGAAGCCAACCCGGTGGATCAGGAGGTTTACAATGCTGAGATTGCCAAAGTGAGAGAGCAGATCACTCGGGCTAAAGCGGAGCTGGCAGCAGAAGAGATCAGGATGACGGCGGAGCGCGCCGCTTTAGAGGCACAGGCTTACAGGCTCATGATGGATCAGAAGGCGTCTCAAGAGGTCCTGAAGAGAAAATCCCGATCTCGGTTGCCCGCCGCCCTTGACGCTCGAAATCTCTTTGACACCCCAGGGGTAAACCGTGCGGAGGCGACAGCCGGCGCCCCGGTTCAGCACCGGATAATAAACCTGCCTTGTCACAACGCTGGTACACCACCGGCCATGCCAACACCGTCAGGTCATTATTCCAACCCGATGGATAACCTCGTCGCGGCCGCGGCTCGGTTGGAAGCCATTCCGATCGAAGGAGATTCACCGCAGAATGAAGAGACGCGCCGGGTCAAGGAGCTCCTTAGGACCGCGTTGGCCCAGCAAGAAGCATATTCGCAAAGCCGTGATCGGATTCACTCCACCCCTCGCCCCAGCAGGAGCTATAGTAGGCGCGTGGAGGAGCCGGCCGTTTCAAGTAACGCCAGGCGTGAAGCGCTCCGCGGCAACAATCCGGCGGGCGACGACAATGCTCACGAGAATGTGGATCGCACCCGTGCACGGGAGGCCGGATTGGCAGCGCAGCATCAGGCTCATCAGCTTACACCTATCTTCCCAGCAGCTGCGGCTGAACCGGGGGTGGTTTCCAGTTCTTTGGGAGTACCGTGCCTTGTTCCCGCCTTGCGCAACGTACGCCTGCCCAAAGACTTCAAAGGCCCCCGCAAAGTACCAAACTACACGGCAGATCCGCCTCCGGAGACGTGGGTGGAGAGCTATGAAATGGCTATGGAGATGCTGGATGTGGATGACGCGGCGTGTGCCAAGTATTTTACTATGATGTTAGAAGGAACGGCCCGGACTTGGCTAAAGAGTTTGCCGCCTAATTCAATTAGCTCGTGGGCCCAATTGCGCGCCCGGTTTATCAAGAACTTCAAGGACACGTGCAAACAGCCAATGTCAATAGTTGACCTAGCAGCCTGCGTTCAACAAGAAGGGGAATCAACTACCCATTGGGTACGACGAGTATCAGAAGTTTTGCACTCATCAGATCGCATCAACGCAGACACCGTTGTAGTGCTGTTGGAAAGCAACTGTCGGTTTGGCCCTTTGAAGCTAAAGCTGGGCCGGATGAAGCGTGATTGTACAGATATAGGAACGCTCATGACCGCTTTAGTGAAGTATGCGGATTCTGACAGTACCAAGGATCCTGACTCTGGTGATGACAAAGCAGGGAAGGGAAGGAAGAATAGCAACACCAAAGGCCAGCAGCATCGACCGACAGGTAATGGAGGCGGAGGTAAGCGTAAACCGGACGGGAGCATGGACTTTGTAGCCAACACAAGTGCACAGAATAAGGGCCACCGGCGCAAGGGGAAACAGCCAAGTTTCCGCACAGATCCTGGTCCTAATCCGGAGCGTTTGAACTTTTTCTTAAACCAGCCCTGTCCGAAGCACGGAACAAAGGAGGAACCAGCAACCCATCTTTGGAAGGATTGTTATATCATGAAGGAGTTCAAAAGCTCAAACACTTTCCAAAATGATCGCAGCTCCGGCGGCGGTTCAGGTTCCGGTCCAGGGTTCCAGGGCAATCCGGGCGGACAGGTCAGCCAAAATAATCAAAGTAATCAGAGTGGTTTCGGGCAGCAGCAAACAGGTTATCAGAGCAACCCGAAACAGTTGAGTGGTGGACAGTATCATGTCTTTACTACAAGCCTGGATAAACGGGATAGAAAGCTTCAGAGGCGAGCAGTTAGTGCTATCGAACCAGCCACGCCTCACTATCTGCGCTGGTCAGAACAGCCTATCATATGGAGCCGAGAGGATCACCCTCCCAGGGTTTATAATCCGGGTCAGTTAGCATTGGTGGTGGCGCCTCAGGTGGGAGGTTATAAGCTCACCAAGGTGCTCATGGACGGAGGGAGCAGCATTAACATCTTGTATTACGAGACCTTCCGTCGTATGGGACTGGTAGATAAGGATCTCAAACCGACCAATACAGTGTTCCACGGGGTGGTGCCTGGCAAGTCTGCATATCCGGTTGGTAAGATAGCCCTTGAAGTGGTATTTGGCGATAGTCACGATTCCAGATCAGAGACGCTGACGTTCGAAGTGGTTAAAATCCAAAGTCCGTACCACGCTTTGTTCGGACGGCCAGCTTATGCAAAATTTATGGCACGGCCCTGCTACGTGTATCTGCAACTTAAGATGCCAGGTTACAAGGGCACTATAACGGTTCATGGGAGCCGTCGAATCGCTTTGGAATGCGAGGAAGGAGATGCGACTTATGCAGAATCGGTTTGTGCTACGGAGGAGCTAAAGCAGTACAAAGACAGTGTTGATCCGGAGGATATGACTTCATTAAAAAAGCCAACTACGGACCACGATCCGGCCTTGAAGTTCAAGTCAGCGGCCGAAACTAAGCTTGTTGACTTCGTACCTGGCGATTCATCCAAGCAGTTCAGCATCAGTGCAAACTTGGATCCAAAATAGGAAAGCGCGCTCATCGAGTTCATCCGTGAGAATCGGGACATCTTTGCATGGAAGCCATCTGACATGCCTGGTGTACCGAGGCAACTCGCTGAGCACACCCTTAATGTGGATCCTAAGTACAAGCCGGTAAAGCAGTTCTTACGGCGGTTTAATGAAGAAAGACGTAAAGCGATTGGTGAGGAAGTGGTCAGGCTCCTGGCAGCTGGGTTTATCATTGAGGTTTTTCATCCCGAATGGCTCGCTAATCCGGTGCTAGTTCTTAAGAAAAACGGCACCTGGCGCATGTGTGTGGACTACACAGACTTGAATAAAGCTTGCCCAGCAGATCCTTTTGCTCTCCCCCGGATTGATCAGATCATTGATGCTACGGCAGGTTGCGAGCGTTTAAGTTTTTTGGATGCCTATTCGGGTTATCATCAGATCAAGATGGCAGTTAAGGACCAGGAGAAGACAGCGTTCATTACTCCCTTTGGAGCCTTCTGCTATGTGTCTATGCCCTTTGGGCTTAAGAGTGCGCAGGCTACTTATCAACGATGTGTGCAAAATTGCCTCCACGAGCAGATTGGTCGTAATGTGCATGCTTATGTGGATGATATTGTGGTCAAGTCACGAAAGAAGGAGACCCTGGTTGACGATTTGAAGGAGACTTTCGATAACTTGAGAGTGTACCAAATGATGCTCAACCCGGCTAAATGTGTTTTTGGTGTACCTGCAGGCAAGCTGTTGGGATTTTTAGTGTCCCATAGAGGAATTGAGGCCAATCCAGAAAAGATCACAGCCATCACCTCCCTGGCCAAACCAAAATGCATCAATGATGTTCAGCGCATGGCCGGGAGGATTGCAGCGTTAAGCCGGTTTATCAGCCGCCTTGGTGAAAAGGCGATCCCCTTGTATCAAATGCTCAAGAAGACAGATCAATTTGTCTGGAGTCCGGAGGCTGATAAAGCGTTTGAGGACTTGAAGCGACAGCTAGTCAATCCGCCAGTTTTGGCAGCCCCTGTAGATAAAGAGCCACTCCTGTTATATGTCGCTGCGAATGCCAGAGCGGTTAGTGTGGCGATGGTGGTAGAACGAAAGGAGGCTGGAAAGGAACACCCGGTTCAACGACCGGTTTACTATATCAGTGAAGTACTTATCGAGTCCAAACAAAGGTATCCGCATTGGCAGAAGCTGGTATATGGTGTTTTTATGGCAAGTCGGAAGCTGAGGCAATATTTTCAAGGACACCCCATCACGGTGGTCAGTTCTGCTCCTTTGGGTGACATTATACAGAACCGGGAGGCGACCGGTCGGATTGCCAAGTGGGCTATCGAGCTCAGGCCGCACGATTTAAGGTACGTACCCCGGACGGCCATAAAATCACAAGCACTTGTCGATTTCATAAACGATTGGACAGAGTTACAAGCGCCTGAGGAGAAGCCAGATAACACCTATTGGACCGTTCATTTTGACGGGTCAAGACAATTGGAAGGCTCGGGGGCTGGAGTCGTTTTAACTTCCCCACGAGGAGATAAGTTTTGTTATGTCCTCCGTTTAATGTTCCCCTGTACAAATAATGCGGCTGAGTATGAAGCTTTGCTTCATGGTCTTCGGATGGCTAAAGAGATGAATCTGAGCAGAGTTAGGTGCTTTGGTGATTCGGATCTGGTGGCTCAGCAGGTATCTGGTACCTGGGATTCTAAGGACCCGCTCATGGCTGCATACCGACGTGAAGTAGATATGGTGGCCGGGCATTTCAAAGGCTATCAAGTGGACCATGTGGACCGCAGAAAGAATGAGGCAGCGGACGCTTTAAGCCGCTTGGGCTCTCAGCGTAAACCGGTCCCGCCCAATGTTTTCTTGGACGTGTTGTATCACCCATCGGTACAGCTGCCCGGTGAGCTGGAGTTGGCTGTTCCTGATCCGGAGGCTCAATTGGTGGCGGCTCTTCACATCACCCCGGATTGGACGCTCCCTTATCTGGCATACATGAACCGGGGTGAGTTGCCAGAAGATGAAAGCTTGGCTCGACAGATAGTACGACGGTCCAAGTCTATGACCATTTTCAAAGGAGAGTTACATCATCGCAGCGTGTCAGGAGCGCTGCAACGGTGCGTATCCCCTAAGGAGGGGTGCGAGATATTACGAGAAATACATGAAGGGGATTGCGGCCACCATGCCGGTTCAAAATCATTGGTGGCTAAAGCGTTCCGCCACGGTTTCTACTGGTTAACTGCTCATGCTGATGCAGAAGACCTAGTCAGATTATGTGATGGGTGCCAGAAATTTTCCAGACGAGCACACATACCGGCTCAAGAATTGAGGATGATTCCAATCACTTGGCCGTTTGCGACTTGGGGGCTTGATATGGTTGGGCCTTTTAAGCGTTCCAAAGATAAGAAGACCCACCTACTGGTGGCGGTTGACAAATTCACAAAGTGGGTGGAGGCCGAACCTGTCAGTAAGTGTGATGCTGCCACGGCGGTTCAGTTTATAAAGAAGGTGATCTTCCGGTTTGGCTTTCCACACAGCATCATCACAGATAATGGTACCAATCTATCCAAGGGGGCCATGAAAGAGTTCTGTGCACGGGAGCATATACGGCTTGATGTTTCTTCGGTAGCGCACCCACAGTCTAATGGTCAGGCAGAACGAGCGAACCAAGAGATCTTGAGGGGTACCAAACCCCGGCTTCTGGTCCCTTTGCAAAGGACGCCGGGTTGTTGGGTGGAGGAGCTACCATCTGTGTTATGGAGTATCAACACCACGCCTAACAGATCCACGGGGTTTACGCCGTTTTTTATGGTCTATAGAGCAGAGGCGGTTCTCCCAAGTGATATACGACATGACTCACCTCGCGTGGCAGCATATGTTGAAGCGGACAATGAGCAGGCACGGCAGAACGCTCTTGACTTGTTAGATGAGGAGCGTGACTTGGCAGCTGCCCGTTCAGCGATTTACCAGCAAGATCTTCGCCGTTACCACAGCCGTCGGGTTAAGACCAGAACTTTTCAGGAGGGGGATTTGGTGCTTCGGCTCATCCAGGATCAGTCTGATCAGCATAAATTATCCCCGCCTTGGGAAGGACCTTTTGTGGTCAGCAAGAATCTGAATAATGGGTCATATTACCTGATTGATATTCGAGAGCGCAAGGATTCACGCACATCAGAGGAGGAGACCAGCAGGCCGTGGAACATAGCTCATCTTCGACCTTATTATACATGAGCCTTGGGCTCTGTTTATGTACATATCATAACCATGTATATATTATGATTAATACAATAAACCGGAGCCTCGAGTAAAGCGGGGTCTCTGCTGTTCTTCACATCATGCGTGTTTACCCCTGGAGGTCGCTTCACAGAAGTTCAAATTATAAATTCCGGTTTAAAAGCCGGTTCAAGGGAAGATGTCTCCTGCAAGGCTGTGAAGCTCTTAATATCTGGTTCAATATCCCGGTTCAAGCATGAGGCTTCCTGTTCAAACATAGGTCGCATTCGAACCAAAGAGAACACAGCTATTCAAACATGGGGCTTCCTGTTCAAACATAGGTCGTATTCGAACCAAAGAGAACACAACTATTCAAACATGGGGCTTCCTGTTCAAACATAGGTCGTATTCGAACCAAAGAGAACATAGCTATCCCTATAAAAGTCAGTTGGGGACCTAGTCGGCCTGAACCGTAGCTACACCTCCGGGGGGCTTGGTCATGTCTTGACGACGGTAATGCCTTCTGGCTGGCACATTTGCCACTGAATCAGTTGGGGACCTAATCGACTTGAACCGTAGGTTACGCCTTATGGGAACTCGGTCATGTCTTGATAATGGTAATGCCTTCTGGTCGGCTTTTTAGCCAATGAATCATTTGGGGGCTTGGTCGTGTCCGAACCAACGCAATGCCACTTGATCGGCCCTTGGCCATTGAGTCACTTGGGGGCTTGGTTGACGAACCAATGCAATGCCATTTGGCCGGCATATTTGCCACGATTCAGTTAGGAACCTAGTCGACCTAAACCGTAGCTACACCTCTTGGGGGCTCGGTTTTGTCTTGACGATGGTCACGCCTTCTGAACGGTTCATAGTAACCACTATTCGCATGCGTTTTACTATCGCGTTGCCTTTTCATATTTGGTTTTGTTTTTCTGCCTTTATTGTGGTGTTTTTTTTTGGTTCTTCGGAACGTCAAGCATGCTTAAACCGATTCAGCTGGCATAGCCTGGTCCGTTTTTTAACCCGGAGGCAAATTGCCCGGTTTAGCAAAGGCGCAGCACGGTTCAGGTGGTCGAAAAATCACATTGTGTACTCAGTAAAGGCAGGAAACATAAGTTGGAAACGTCATTGGAAAGAGCATCAGTACCCGTGCGCGAAGGCACATTCAAATGTAGAGGTATTAAACTATCTTATTACAAGGCAACGTAGTGCCTGAATATATTGTTCGTTGACGGATTAAGACGTCACCAAGTAAGGTGAACCGGAGCGTTGATTACGCCTGTTCGCCGCCTTGGTTCGAAGGCTGGGGATCGTCTCGCGCCGCTTCAGCTCCATCTTGGTTACCCATTGGCTGGAAATCAGCAGTGGTCCAGTCGATTCCCATTAGCGCTTGAAAGACGGCTTCTTCGTGAATCAGCGAAGACGGTTCAATGTCTGGGGCGTAAGTATGCTTACGGATTGGAGGAACAAGGTTTCCCGCTTCAGGGACCGGGGCAGATACTCGTTTGTTCTGGTTGTCGTATTGGGCTTGGTAACGTGACAGATCTGCTTCCTCAGCCAATTGACAAGCTAGGGGGCGCACCGCCCGGTTTATCGCCCTCAAATCGTCTTCACCAAAGTCTGAACCGTCTTCCTTCAAGCTGGGGTATCCTTGGGCCGCTTCGACAGGATCGAAATCTGGTACCCACGCTTTGGCCCGGATCAAGGCAGTGATGGCTCCAGTTCGAGCAGCTGATCTTTTCAACTCTTCAATCCGGGCCGGAAGCATGGACAGCCTCTTTAATGTGTCTTGAATCAGAGACGGCGCAGGGTTGTTATGCGATGCAGTGGTGATAGCTCTTTGTGCTCCGGTGTATAACTGTTCAATCAGAGTATAAGCAGCTTTGAGTTTCATCCGCACATCAGAACCCAAGTGTCCAATACGTGAGCCTGCAGTGTTTCAAGGTGGATTACAAACCGGACATAGGGTGTAGCAAACAAGGTACAAATTGGAAGAAGGGCATCGAAAAGTTTACCAAAGATGGCAGAGGTCATGGAGTGTATTTGTCGCTTCACGGTGGATAACTCATCAACCACCGGTTTAAGAGCAGCTTCGGCATTTTCAGCCCTCTTCGTCAAAAGAGCTCTTTCAGTAGCCCAATCGGCTTGCTCCTTTTTGCGGCTTGCTTTCAGCTGCTCCATAACAGTTAAGGCTTTGGTCAATTCCTCCTTTGCTTTTTCAGTTTCAATCTGCTGGGCTTTTAAAGCTTCCTGAAGGTCCGAGACTTGGTTCTCTTTGGTCTTCAAATCCCCCTGCAGCAGGTCATCAACAGTGTTCAGAAAATATTGCACGGTTTGGAAGTATCAACCACACAAGCAAGTTATGTGCTCGATACTTGGGGGCTAATGCATATTTGATTTTAACACAAGTTTTCCATTACAAAAAGTCCCAAGATTAATACAAGTATTTAAGCTTGGCACTTGGGGGCTAATAGTTATCTAATGATGTTTTCTGGTTATGACCTATGTTGAAGCCTTGGGGTTTTCTAAACCGGACTTTAATTGTCAAGGCAGCCTGATCCGGTTTACCATCTCGGTTTGGAAATATTGATAAGCTAAGTTGCAATGGAATGAAGCACAAGGGAGTTACCTCAAGTTTCTCCTTCATCATCTTGATCATGCCAGCTTCATAATCCCGACTGGTGTACAGTCGGTTCAGGTACCCAGAATAAAGATCCGGGGCGCTTAAAGCGGCGTACGTAGAAGAGTCAAGGTCCCATTTGCCCTTCACGGCCGAGATTTCGTCCTTGGCAGTATGCTTGGCTAAGGTAGCAGTGTTCCCCGGTTCAGAGTGACCGATGCCGGTAATCACAACATCATCATCCTTGGAATCGCTGCTCTGCAGTGGCCCTGTCGGTTTATCAGCAGTTCCAGAAGGGGGGATGGATTCCTCAACCCGAACAGGACTGACAGGCGGATCAGTATGGCTGGCAGGTTCGATAATCCTTTCTTCAGCGCAGGTGTCATCTTGCGGAGATAGATAATTCTGAGTATCTTCACGACCCAGAGCGTCAGCACCAGGAGTTTTCTCCGGTTCAGGAGCGGCAGTTCCTCCAGCCGGGTTATCCAGACGAGCCTTCTTGCTCGGTCTAGGTTTGGCCCTGGAAAAAGCCCAAAGATTGAAAAAACGGTATAAGATGGATTAAGAGACCCATACAGAGATATGTTGTGATCGGTCCAACTTACCCAATAACTGTCTTCAACGGTGGCAGTTGAGTGGCTGAAGATTCACCGGAAGAGGAATGGGAAGTGACCTGATAATCGGAGTCAGATGGATTTAGAGGCTGACGAGTTAAACCTGCTTTAGGAGGAAAGCTAGCGCGTAAATCGGAGACCTCTGAGCGGCGTTTCCGAACCGGGCTGTTCGGTAAACCGGCGGAGGTGACTACCTGGCCGCTATGTCGGGTGGTGCGTCGAGCTTCATGTTGCTGGGTCTTCACAAGAAATTTGGGATCCAAGTAAGCAAGAGGGTGAGAAAATTTAATTCTCCGGTTTGCTTGGCGAGGTTTTTTCAAAGGCAAAGGAACGGCATCAGAAGAAAGAATAGTTACCTCTGGGCCTTCGGCATGGCTCACTTCAGCGTCGTCCTGACAAATATCATCATCAATAAGACATATAAAAAGCAAACCAACTGAATCGAGGTCTACCCCGAGGTCCGGATTACCCACATCATCCTCCTCTAAATCCGGAGTAGAGGAAGCGGTGTTCCTTTTCCTCTGAGCAGGCTTCTTCACCCTCGTCTTGGGTCGGGTTACCTTTTCGGTCGGCTCTTGTAACTTCTTGCTCCAAAATGGATCATCTCCCTGTCTCAGGAAGGACTGGAGTTAGCAATATGTGTATAAGGAGATCATAAGTTCTATAACAAACAGAGGGGAGATACTTACAACAGGTGGCTTGTTTGTGGCACAAAATGGAAGCAGGCCGGTTCGGGCACAAACATGTTCCGGTTCATTCAGCATCTTTTTCACAGCTTCAGCGACTTCGTCCTCCGTCAGCTGAATGTTGCAGTGCCTCAAGGGATCATCAACCCGACCCGTGTATTGGCACATTAAACCGGAGCGGATGCTAAGGGGCAGGATGCTCCATGAAATCCAGCAACGAGCAAGATCTACCCCCGTTAAACCGTTAGCCATGAAGGCTCTGAGCTTGGACAGCTGAGGGGCATATTTACTCCTCTCTTTGGCAGTTAGCCGTGGGGGGGAGGGGTGAGTATTGCTAAGTCTCTCTGGACGGAAACCGGGCAGTGGATTTTCTTCAGCAGGGGAAGTATCCTTGCAATAGAACCAAGTGCAATTCCATTCTTGCGGATGACTATGCAACTTGGCGTGAGGATAAGTCACCTCTTTCCTCTTCTGAATCGCCATACCACCCAGTTCCATATTAGGGCCATCGGTAAATTCAGTACGTCGGTTCAGATGGAAAAAGTCCCGGAACAGCTCTACAGTGGGTTCCTCTTGAAAGTACGCCTCGCAAAGTACTTGAAAATGACAGATGTTCGTGACTGAATTTGGACCAACATCTTGTGGGCGCAACCTAAAATTAGCCAAGACGTCCCGATAAAATTTTGAACCGGGTGGGTTAAACCCCCGGGCCAGGTGATCCGCAAACACCACCACTTCTCCCTCCTGCGGGGTAGGAGGACATTCGTCTCCTGGAACCCTCCAATGGATGGTATTTTTGCTACTCAAAGCACCAGTCAGAACTAAGTTGTTTAGTTGGGTCTCAGTGATGCAAGAGGGAACCCAGTTGCATTCGTACACCTGTTTTGCCATGCGGAAATCTACAAGATAAATGTATTCCGGTTTGAGAACAAGTGTGATTATAAACCGAAGATGATCTTATATAGAGTCAGGTTATCCACAATGATATCAACCAGCGGTTCATCAAGGGGACTAATGGATGTGTGGATCGTTTTGTTCACTTTGCTAAGAGTTAAACCGACCAGATCTAACAGCTGTGAGTATGAAAGCAGAAACAGATCCCATACGGATTCTTTCAAAGCAATGGATCTACAGCTAGGGCGAAAAAGAAATAAAAATATGAAGTTTGGCTCATAGCAGGACAACATAAGTTCGACAAGCAGGAAATGAGCGAGATCTAAGAAGACGAATACAGATTTATGGCGAATACCGAGAGGAACTACAAATCCAGATACAGGTATCATCATCTGTTCGCAAAGGCTGCTATGGGGATGAGCAAGAAACAGTAGCAGGACGACGAACCCTAGAACAGATCCAAAGCAACAAGAACAGGGGCCTTACCGGGGTCCAAACAGATGTGGAAAGTCGTCGCGGTGCTCTGATCCGATCAGGTTGATGCAGCGGCCGGAGTTGATGCAGCAGCTGACGGCGGCGGCGGAGCTCTGAGGTCTGGGTCGCGAGGAAGACGAAGAAGGGGAGGAAAGGAAGATGGCCTTCAAGTCCTATTTATAAGGCACGGCGCGCGTGTCAGGCGCGCGAATCCAGGGGCCGGAGATTTCGGAAGCCCAGCCGTCGCCCTGATTCTTGCGGGTTAGCGAAAGGAGACGGTAACTTGCGCTGACGTCATATCGGTTTACCATAGCAAGAGGGGACGGAGAAACAGCGGTTTAACAAGTTACTGGAAGACATTTGAAGACAGAAATTCTTATTAAGGATTGACATGAACCTGTTCAAATCAATCTGGGGCCTAA

TGTTGGGGATGTTGCTATCTGGGGTAAACCGGCCTGCAGGGCCCGGGTTACCGCCGTCAACAGACCCGAGATGTTAAAGCCCAAGGAGAAATAGGAAGGCCCAAGGCCTATAACCGTTTTAAAGGTTTATGGTGTAAACCGGCCTGGGGCCAACTTGTATTGTAAGATAGGAATAGAGAGACCGACCCGGGCACGTTTATAAGCCGGAGGTTGGGACTTTGCAGACCGACGGGTGTCACCCGTGTATATAAGGGGACGACCCGACGGCGGTTTGGGACAGAGAAGAAGAACTCGAGGTTTAGGCGAAGCTTATCGCTCCTTAAGCCCTCGAAACCCATCAATCCCATCACAACTAGACGTAGGCTTTTACCTTCATCGAAGGGGCCGAACTAGTATAAAAACCGTGTCCTTGTGTCCACTTTAACCCCTTCAAGCTAACCCGTTGCGATGGCTCCACGACTAAGTCCTCTCACTAGGACATCTGCCGTGACAAATCCACGACA

CCTTGGTTCAACTCCACAATCTTGTCGGAGGTTCCCAAGTGACACCTAGCCAATCTAGGAGACACCACTCTCCAAGAAGTAACAAATGGTGTGTTGATGATGAACTCCT

>Aegilops umbellulata 1-032;

AACAGAAGGCCTGTTTCCTCCGTTTTATGGTACCCCAGACCTCGTTTCGGCTGTTCCGTCCAAGACGGTTGGCTCCCAATGAACACGACGTATTCCGTTGCCTCCCGATGAACACAACGCATTC

TGTTGGGGATGTTGCTATCCGGGGTAAACCGGCCTGCAGGGCCCGGGTTACCTCCGTCAGCAGACCCGAGATGTTAAAACCCAAGGAGAGATAGAAGGGCCTAAGGCCCATAATCGGTTTAAAGGTTTATGGTGTAAACCGGCCTGATGTAAACTTGTATTGTAAGTTAGGAATAGAGAGACCGACCCGGGCACGTTTATTAGCCGGAGGTTGGGACCTTGCAGACCGACGGGCGTCACCCGTGTATATAAGGGGATGACCCGGCGGCGGTTTGGGGACAGAAAAAGAGAACTCGAGGTTTAGGCGAAGCTTATCGCTCCCTAAGCCCTCGAAACCCATCAATCCCATCACAACTAGACGTAGGCTTTTACCTTCATCGAAGGGGCCGAACTAGTATAAAAACCGTGTCCTTGTGTCCACTTTAACCCCTTCAAGCTAACCCGTTGCGATGGCTCCACGACTAAGTCCTCTTACTAGGACATCTGCCGTGACAAATCCACGACA

GTTGGCGCCCACCGTGGGGCTATCGCGAGATGGTTTCAGGTTCTTGGAGGGCCGCTTTGAAGGACTCGAGGGTTACGCTGTGGGACGGATGACCAAGAGCCGCCGCGGAAAACTCTACATCGACAGCACAGGCTGGGGCCCCGACGCCGGCTCGATCGAGTACGGGTACCGGGTCCCCTTTGGCGGCATCCATGTTTTCATCGGCAAGATCGGAGAACCGGGCCCCGAGCTAGGCGTCTGCGCCGACCTCGTCGAGACGGCTCAGTGCACGAGATCGACCCTGGCTAAACCGATCAGAAAGCACGCCTTCGTGGGGGTCATCCGCGGAGGAAGCTACGAGGAAGGATCCGAATCTGGTGACGAAACCGCCATCTACTCCGGCGATGAAACGTCGACCGGGGAAACCGAATCTCTTTACCAGCTACAAGATGATCGGACTGAGGGCGGCTCCGATGGCAACAGTATTCCGGACCCCTCGGATCTGCCCTATCGGGTTGGGATCTTCATGGCTGGAACGCAGGCAGCGCCACGATCATCAACTGCAGCAGCAGGGATCTCCGGATTGGCAGCGGCAACGGTCGCACGGGCAGGAGGCCCTACGGGTTCGCCAGCCCAAGTTTTGTCAGACCTATTTGACGCACTAGCAGCGCTCATGGCGGAAGCCAACCCGGTGGATCAGGAGGTTCACAATGCTGAGATTGCCAAAGTGAGAGAGCAGATCACTCGGGCTAAAGTGGAGCTGGCAGCAGAAGAGATCAGGATGACGGCGGAGCGCGCCGCTTTAGAGGCACAGGCTTACAGGCTCCTGATGGATCAGAAGGCGTCTCAAGAGGTCCTGAAGAGAAAATCCCGATCTCGGTTGCCCGCCGCCCTTGACGCTCGAAATCTCTTCGACACCCCAGGGGTAAACCGTGCGGAGGCGACAGCCGGCGCCCCGGTTCAGCACCGGGTAATAGACCTGCCTCGTCACAACACTGGTACACCACCGGCCATACCCACGCCGTCAGGTCATTATTCCAACCCGATGGATAACCTCGTCGCGGCGGCGGCTCGGTTGGAAGCCATTCCGATCGAAGGAGATTCACCGCAGGATGAAGAGACACGCCGGGTCAAGGAGCTCCTTAGGACCGCGCTGGCCCAGCAAGAAGCATATTCGCAAAGCCGTGATCGGATTCACTCCACCCCTCGCCCCAGCGGGAGCTATAGTAGGCGCGTGGAGGAGCCGGCCGTTTCAAGCAATGCCAGGCGTGAAGCGCTCCGCGGCAACAATCCGGCGGGCGTTGACAATGCTCACGAGAATGTGGATCGCACCCGTGCACGGGAGGACGGATTGGCAGCACAGCATCAGGCTCATCAGATTACACCTATCTTCCCAGCAGCTGCGGCTGAACCGGGGGTGGTTTCCAGTTCTTTGGGAGTACCGTGCCTTGTTCCCGCCTTGCGCAACGTACGCCTGCCCAAAGACTTCAAAGGCCCCCGCAAAGTATCAAACTACACGGCAGATCAGCCTCCGGAGACGTGGGTAGAGAGCTATGAAATGGCTATGGAGATGCTGGATGTGGATGACGCGGCGTGTGCCAAGTATTTTACTATGATGTTAGAAGGAACGGCCCGGACTTGGCTAAAGAGTTTGCCGCCTAATTCAATTAGCTCGTGGGCCCAATTGCGCGCCCGGTTTATCAAGAACTTCAAGGACACGTGCAAGCAGCCAATGTCAATAGTTGACCTAGCAGCCTGCGTTCAGCAAGAAGGGGAATCAACTAACCATTGGGTACGACGAGTATCAGAAGTCTTGCACTCATCAGATCGCATCAACGCAGACACCGCTGTAGTGCTGTTGGAAAGCAACTGTCGGTTTGGCCCTTTGAAGCTAAAGCTGGGCCAGATGAAGCGTGATTGTACAGATATAGGAACGCTCATGACCGCTTTAGTGAAGTATGCGGATTCTGACAGTACCAAGGATCCTGATTCTGGTGATGACAAAGCAGGGAAGGGAAGGAAGAATAGCAACACCAAAGGCCAGCAGCATCGACCGACAGGTAATGGAAGCGGAGGTAAACGTAAACCGGACGGGAGCATGGACTTGGTGGCCAGCACAAGTACACAGGATAAAGGCCAGCGGCGTAAGGGAAAACAGCCAAGTTTTCGCGCACATCCCGGTCCTAACCCGGAGCGTTTGAACTTTTTCTTAAACCAGCCCTGTCCGAAGCACGGAACAAAGGAGGAACCAGCAGCCCATCTTTGGAAGGATTGTTATATCATGAAGGAATTCAAAAGCTCAAACACTTTCCAATATGATCACAGCTCCGGCGGCGGTTCCGGTTCCGGATCAGGGTACGGTGGCGGGAATTCCGGTCCAGGGTTCCAGGGTAACCCGGGCGGACAGGTCAGCCAAAATAATCAGAATAATCAAAGTAATCAAAGTGGGTATCAGAGCAACCCGAAACAGTTGAGTGGTGGACAGTATCATGTCTTTACTACGAGCTTGGATAAACGAGACAGGAAGCTTCAGAGGCGGGCAGTCAGCGCTGTCGAACCGGCCACGCCTCACCATCTGCGTTGGTCGGAACAGCCTATCATATGGAGCCGAGAGGATCACCCTCCCAGGGTTTATAACCCGGGTCAGTTAGCATTGGTGGTGGCGCCTCAGGTGGGAGGTTATAAGCTCACCAAGGTGCTCATGGACGGAGGAAGCAGCATTAACATCTTGTATTACGAGACCTTCCGTCGTATGGGACTGGTAGATAAGGATCTCAAACCGACCAATACAGTGTTCCACGGGGTGGTGCCTGGCAAGTCTGCATATCCGGTTGGTAAGATAGCCCTTGAAGTGGTATTTGGCGATAGTCATGATTCCAGATCAGAGACGCTGACGTTCGAAGTGGTTAAAATCCAAAGTCCGTACCACGCTTTGTTCGGACGGCCAGCTTATGCAAAATTTATGGCACGGCCCTGCTACGTATATCTGCAACTTAAGATGCCAGGTTACAAGGGCACTATAACGGTTCATGGGAGCCGCCGAATCGCTTTGGAATGCGAGGAAGGAGATGCGACTTATGCAGAATCGGTTTGTGCTACGGAGGAGCTAAAGCAGTACAAAGACAGTGTTGATCCGGAGGATATGACCTCATTAAAAAAGCCAACTACGGACCACGATCCGGCCTTGAAGTTCAAGTCAGCGGCCGAAACTAAGCTTGTTGACTTCGTACCTGGCGATTCATCCAAGCAGTTCAGCATCAGTGCAAACTTGGATCCAAAATAGGAAAGCGCGCTCATCGAGTTCATCCCTGAGAATCGGGACATCTTTGCATGGAAGCCATCTGACATGCCTGGTGTACCGAGGCAACTCGCTGAGCACACCCTTAATGTGGATCCTAAGTACAAGCCGGTAAAGCAGTTCTTACGACGGTTTAACGAAGAAAGACGTAAAGCGATTGGTGAGGAAGTGGCCAGGCTCCTGGCAGCTGGGTTTATCATTGAGGTTTTTCATCCCGAATGGCTCGCTAATCCGGTGCTAGTTCTTAAGAAAAACGGCACCTGGCGCATGTGTGTGGACTACACAGACTTGAATAAAGCTTGCCCAGCAGATCCTTTTGCTCTCCCCCGGATTGATCAGATCATTGATGCTACGGCAGGTTGCGAGCGTTTAAGTTTTTTGGATGCTTATTCGGGTTATCATCAGATCAAGATGGCAGTTAAGGACCAGGAGAAGACAGCGTTCATTACTCCCTTTGGAGCCTTCTGCTATGTGTCTATGCCCTTTGGGCTTAAGAGTGCGCAGGCTACTTATCAACGATGTGTGCAAAATTGCCTCCACGAGCAGATTGGTCGTAATGTACATGCTTATGTGGATGATATTGTGGTCAAGTCACGAAAGAAGGAGACCCTGGTTGACGATTTGAAGGAGACTTTCGATAACTTGAGAGTGTACCAAATGATGCTCAACCCGGCTAAATGTGTTTTTGGTGTACCTGCAGGCAAGCTGTTGGGATTTTAGTGTCCCATAGAGGAATTGAGGCCAATCCAGAAAAGATCACAGCCATCACCTCCCTGGCCAAACCGAAATGCATCAATGATGTTCAGCGCATGGCCGGGCGGATTGCGGCGTTAAGCCGGTTTATCAGTCGCCTTGGTGAAAAGGCGATCCCCTTGTATCAAATGCTCAAGAAGACGGATCAGTTTGTCTGGAGTCCGGAGGCTGATAAAGCGTTTGAGGACTTGAAGCGACAACTAGTCAATCCGCCAGTGTTGGCAGCCCCTGTAGATAAAGAGCCACTCCTGTTATATGTTGCAGCGAATGCCAGAGCGGTCAGTGTGGCGATGGTGGTAGAACGAAAGGAGGCTGGAAAGGAACATCCGGTTCAGCGACCGGTTTACTATATCAGTGAAGTACTTATCGAATCCAAACAAAGGTATCCGCATTGGCAGAAGCTGGTATATGGCGTTTTTATGGCAAGCCGGAAGCTGAGGCAATATTTCCAAGGACACCCAATCACGGTGGTCAGTTCTGCTCCTTTGGGTGACATCATACAGAACCGGGAGGCGACCGGTCGGATTGCCAAGTGGGCTATCGAACTTGGGCCGCACGGTTTAAGATACGTACCCCGGACGGCCATAAAGTCACAAGCACTTGTCGACTTCATAAACGATTGGACAGAGTTACAAGCACCTGAGGAGAAGCCAGATAACACATATTGGACTATTCATTTTGACGGATCCAGACAGTTGGAAGGCTCGGGGGCTGGAGTCGTTTTAACTTCCCCACGAGGAGACAAGTTTTGTTATGTCCTCCGTTTAATGTTCCCCTGTACAAACAATGCGGCTGAGTATGAAGCTTTGCTTCATGGTCTTCGGATGGCTAAAGAGATGAATCTGAGCCGCGTTAGGTGCTTTGGTGATTCGGATCTAGTGGCTCAGCAAGTATCTGGCACCTGGGATTCTAAGGACCCGCTCATGGCTGCATACCGTCGTGAAGTAGATATGGTGGCAGGGCATTTCAAAGGCTATCAAGTGGACCATGTGGACCGCAGAAAGAATGAGGCATCGTACGCTTTAAGCCGCTTGGACTCTCAGCGTAAACCGGTCCCACCCAATGTTTTCTTGGATGTATTGTATCGCCCATCGGTACAGCTACCCGGTGAGCTGGAGTTGGCTATTCCTGATCCGGAGGCTCAATTAGTGGCGGCTCTTCACGTCATCCCGGATTGGACGCTTCCTTATCTGGCATATATGAACCGGGGTGAGTTGCCAGAAGACGAAATCTTGGCTCGGCAGATAGTACGGCGGTCCAAGTCTATGACCATTTTCAAAGGGGAGCTACATCATCGCAGCGTGTCAGGAGCGCTGCAACGGTGCGTATCCCCTGAGGAGGGGTGCGAAATATTACGTGAAATACATGAAGGGGATTGCGGCCACCACGCCGGTTCAAAATCATTGGTGGCTAAAGCGTTCCGCCACGGTTTCTACTGGTTAACTGCTCATGCTGATGCAGAAGACCTGGTCAGGCTATGTGATGGTTGCCAGAAATTTTCTAGACGAGCGCACATACCGGCTCAAGAATTGAGGATGATTCCAATTACTTGGCCGTTTGCGACTTGGGGGCTCGATATGGTTGGGCCTTTTAAACGTTCCAAAGATAAGAAGACCCACCTATTGGTGGCGGTTGACAAATTTACAAAGTGGGTGGAGGCAGAACCTGTCAGTAAATGTGATGCGGCCACGGCGGTTCGGTTTATAAAGAAGGTGATCTTCCGGTTTGGTTTTCCACACAGCATCATCACAGATAATGGTACCAATCTATCCAAAGGTGCCATGAAAGAGTTCGGCGCACGTGAGCATATACGGCTTGATGTTTCTTCGGTAGCTCACCCACAATCTAATGGTCAAGCAGAACGAGCGAATCAAGAGATCTTGAGAGGTATCAAACCCCGGCTTCTGATCCCTTTGCAGAGGACACCGGGTTGCTGGGTTGAGGAGCTACCGTCTGTATTATGGAGCATCAACACCACGCCTAACAGATCCACGGGGTTTACGCCGTTTTTTATGGTCTATGGAGCAGAGGCGGTTCTCCCAAGTGATATACGACATGATTCACCTCGTGTGGCAGCTTATGTTGAAGCGGACAACGAGCAGGCACGGCAGAACGCTCTTGACTTGTTGGATGAGGAACGTGACTTAGCAGCTGCCCGTTCAGCGATTTACCAGCAAGATCTTCGCTGTTACCACAGCCGTCGGGTTAGAACCAGAACCTTTCAGGAGGGGGATTTGGTGCTCCGGCTCATCCAGGATCAGTCTGATATGCATAAATTATCCCCGCCTTGGGAAGGACCTTTTTTGGTCAGCAAGAATCTGAATAATGGGTCATACTACCTGATTGATATTCGAGAGCGCAAAGATTCACGCACATCAGAGGAGGAGACCAGCAGGCCGTGGAACATAGCTCATCTTCGACCTTATTATACATGAGCCCTGGGCTCTGTTTATGTACATATCATGACCATGTATATATTATGATTAATACAATAAACCGGAGCCTCGAGTAAAACGGGGTCTCTACTGTTCTTCACATCATGTGTGCTTACCCCTGGAGGTCGTTTCACAGAAATTCAAATTATAGACTCTGGTTTAAAAGTCGGTTCAAGGGAAGATGTCTCCTACAAGGCTGTGAAGCTCTTAATATCCGGTTCAATATCCCGGTTCAAGTATAAGGCTTACTGTTCAAACATAGGCCGCATTCGAGCCAAAGAGAACATAGCCATTCAGCATGAAGCTTCCTGTTCAAACATAGGTCGTATTCGAACCAAAGAGAACATAGCTATTCAAACATGGGGCTTCTTGTTCAAACATAGGTCGTATTCGAACCAAAGAGAACATAGCTATTCAAACATGGGGCTTCCTGTTCAAACATAGGTCGGGTTCGAACCAAAGAGAACATAGCTAAACCCTATAAAGTCAGTTGGGGACCTAGTCAGCCTGAACCGTAGCTACACCTCCGGGGGGCTTGGTCATGTCTTGACGATGGTAATGCTTTCTGGCTGGCATATTTGCCACTGAATCAGTTGGGGACCTAATCGACTTGAACCGTAGATTACGCCTTATGGGAACTCGGTCATGTCTTGATGATGGTAATGCCTTCTGGTCGGCTTTTTAGCCAATGAATCATTTGGGGGCTTGGTCGTGTCCGAACCAACGCAATGCCACTTGATCGGCCCTTGGCCATTGAGTCACTTGGGGGCTTGGTTGACGAACCAATGCAATGCCATTTGGCCGGCATAATTGCCACGATTCAGTTAGGAACCTAGTCGACCTAAACCGTAGCTACACCTCTTGGGGGCTCGGTTTTGTCAAGACGATGGTCACGCCTTCTGAACGGTTCATAGTAACCACTATTTGCATGCGTTTTTCTATCGCGTTGCCTTTTCATATTTGGTTTTGTTTTTTCTGCCGTTATTGTGGTGTCTTTTTTGGTTCTTCGGAACGTCAAGCATCCTTAAACCGATTCAGCTGGCATAGCCTGGTTCGTTTTTTTAAACCCGGAGGCAAATTGCCCGATTTGGCAAAGGCGTAGCACGGTTCAGGTGGTCGAAAAATTACATTGTGTACTCAGTAAAGGCAGGAAACATAAGTTGGAAACGTCATTGAAAAGAGCATCAGCACCCGTGCGCGAAGGCACATTCAAATGTCGAGGTGTTAAGCTATCTTATTACAAGGCAACGTAGTGCCTGAATATATTGTTCGTTGACGGATTAAGACGTCACCAAGTAAGGTGAACCGGAGCGTTGATTACGCCTGTTCGCCGCCTTGGTTCGAAGGCTGGGGATCGTCTTGCGCCGCTTCAGCTCCATCTTGGTTACCCATTGGCTGGAAATCAGCAGTGGTCCAGTCGATTCCCATTAGCGCTTGAAAGACGGCTTCTTCATGAATCAGCGAAGACGGTTCAATGTCTGGGGCGTAAGTATGCTTACGGATTGGAGGAACAAGGTTTCCCGCTTCAGGGACCGGGGCAGATACTCGTTTGTTCTGATTGTCGTATTGGGCTTGGTAACGTGACAGATCTGCTTCCTCAGCCAACTGACAAGCTAGGGGGCGCACCGCCCGGTTTATCGCCCTCAAATCATCCTCACCAAAGTCTGAACCGTCTTCCTTCAAGCTGGGGTATCCTTGGGCCGCTTCGACAGGATCGAAATCTGGTACCCACGCTTTGGCCCGGATCAAGGCAGTGATGGCTCCAGTTCGAGCGGCTGATCTTTTCAGCTCTTCAATCCGGGCCGGAAGCATGGACAGCCTCTTTAATGTGTCTTGAATCAGAGACGGCGCAGGGTTGTTATGCGATGCAGTGGTGATATCTCTTTGTGCTCCGGTGTATAACTGCTCAATCAGAGTATAAGCAGCTTTGAGTTTCATCCGCACGTCAGAACCCAAGTGTCCAATACGTGAGCCTGCAGTGTTTCAAGGTGGATTACAAACCGGACATAGGGTGTAGCAAACAAGGTACAAATTGGAAGAAGGGCATCGAAAAGTTTACCAAAGACGGCAGAGGTCATGGAGTGTATTTGTCGCTTCACGGTGGATAACTCATCAATCACCGGTTTAAGAGCAGCTTCGGCATTTTCAGCCCTCTTCGTCAAAAGAGCTCTTTCAGTAGCCCAATCGGCTTGCTCCTTTTTGCGGCTTGCTTTCAGCTGCTCCATAATAGTTAAGGCTTTGGTCAATTCCTCCTTTGCTTTTTCAGTTTCAATCTGCTGGGCTTTTAAAGCTTCCTGAAGGTCCGAGACTTGGTTCTCTTTGGTCTTCAAATCCCCCTGCAGCAGGTCATCAACAGTGTTCAGAAAATATTGCACGGTTTGGAAGTATCAACCACACAAGCAAGTTATGTGCTCGATACTTGGGGGCTAATGCATATTTCATTTTAACACAAGTTATCCATTACAAAGAGTCCCAAGATTAATACAAGTATTTAAGCTTGGCACTTGGGGGCTAATAGTTATCTAATGATGTTTTTTAAACCGGACTTTAATTGACAAGGCAACCTGATCCGGTTTACCATCTTGATTTGGAAATATTGATAAGCTAAAGTTGCAATGGAATGAAGCACAAGGGAGTCACCTCAAGTTTCTCTTTCATCATCTTGATCATGCCAGCTTCATAATCCCGACTGGTGTACAGTCGGTTCAGATACCCAGAATAAAGATCCGGGGCGCTTAAAGCGGCGTACGTAGAGGAGTCAAGGTCCCATTTGCCCTTCACGGCCGAGATTTCATCCTTGGCAGTATGCTTGGCTAAGGTAGCAGTGTTCCCCGGTTCAGAGTGACCGATGCCGGTAATCACAACATCATCATCCTTGGAATCGCTGCTCTGCAGTGGCCCTGTCGGTTTATCAGCAGTTCCAGAAGGGGGGATGGATTCCTCAACCCGAACAGGACTGACAGGCGGATCAGTATGGCTGGCAGGTTCGATAATCCTTTCTTCAGCGCAGGTGTCATCTTGCGGAGATAGATAATTCTGAGTATCTTCACGACCCAGAGCGTCAGCACCAGGAGTTTTCTCCGGTTCAGGAGCGGCAGTTCCTCCAGCCGGGTTATCCAGACGAGCCTTCTTGCTCGGTCTAGGTTTGGCCCTGGAAAAAGTCCAAAGATTGAAAAAACGGTATAAGATGGATTAAGAGACCCATACAGAGATATGTTGTGATCGGTCCAACTCACCCAATAACTGTCTTCAACGGTGGCAGTTGAGTGGCTGAAGATTCACCGGAAGAGGAATGGGAAGTGACCTGATAATCGGAGTCAGATGGATTTAGAGGCTGACGAGTTAAACCTGCTTTAGGAGGAAGGCTAGCGCGTAAATCGGAGACCTCTGAGCGGCGTTTCCGAACCGGGCTGTTCGGTAAACCGGCGGAGGTGACTACCTGGCCGCTATGTCGGGTGGTGCGTCGAGCTTCATGTTGCTGGGTCTTCATAAGAAATTTGGGATCCAAGTAAGCAAGAGGGTGAGAAAATTTAATTCTCCGGTTTGCTTGGCGAGGTTTTTTCAAAGGCCAAGGAACGGCGTCAGAAGAAAGAATAGTTACCTCTGGGCCTTCGGCATGGCTCACTTCAGCGTCGTCCTGACAAATATCATCATCAATAAGACGTATAAAAACCAAACCAACTGAATCGAGGTCTACCCCGAGGTCCGGATTACCCACATCATCCTCCTCTAAATCCGGAGTATTGGAAGCGGTGTTCCTTTTCCTCTGAGCAGGCTTCTTCACCCTTGTCTTGGGTCGGGTTACCTTTTCGGTCGGCTCTTGTAACTTCTTGCTCCAAAAGGGATCATCTCCCTGTCTCAGGAAGGACTGGAGTTAACAACATATATATAAGGAGATCAAAGATTCTATAAACAGAGGAGTTACTTACAGCAGGTGGTTTGTTTGTGGCACAAAATGGAAGCAGGCCGGTTCGAGCGCAAACATGTTCCGGTTCGTTCAGCATCTTCTTCACAGCTTCAGCGACTTCGTCCTCCGTCAGCTGAATGTTGCAGTGCCTCAAAGGATCATCAACCCGACCCGTGTATTGGCACATTAAACCGGAGCGGATGCTAAGGGGCAGGATGCTCCATGAGATCCAGCAACGAGCAAGATCTACCCCCGTTAAACCGTTAGCCATGAAGGCTCTGAGCTTGGACAGCTGAGGGGCATACTTACTCCTCTCTTTGGCAGTTAGCCGTGGGGGGAAGGGGTGAGTATTGCTGAGTCTCTCTGGACGGAAACCGGGTAGTGGATTTTCTTCAGCAGGGGAAGTATCCTTGCAATAGAACCAAGTGCAATTCCATTCTTGCGGATGACTATGCAACTTGGCGTGAGGATAAGTCACCTCTTTCCTCTTCTGAATCGCCATACCACCCAGTTCCATATTAGGGCCATTGGTAAATTCAGTACGTCGGTTCAGATGGAAAAAGTCCCGGAACAGCTCTACAGTGGGTTCCTCTTGAAAGTATGCCTCGCAAAGTACTTGAAAATGGCAGATGTTCGTGACTGAATTTGGACCAACGTCTTGTGGGCGCAACCTAAAATTAGCCAAGACGTCCCGGTAAAATTTTGAACCAGGTGGGTTAAACCCCCGGGCCAGGTGATCCGCAAACACCACCACTTCTCCCTCCTGCGGGGTAGGAGGACATTCGTCTCCTGGAACCCTCCAATGGATGGTACTTTTGCTACTCAAAGCACCAGTCAGAACTAAGTTGTTTAGTTGGGTCTCAGTGATGCAAGAGGGAACCCAGTTGCATTCATACACCTGTTTTGCCATGAGGAAATCTACAAGGCAAAGGTATTCCGGTTTGAGAGCAAGTATGATTGTAAACCGAAGATGCTCTTATATAGAGTCAGGTTATCTACAATAATATCAACCGGCGGTTCATCAAGGGGACTAATGGATGTGTAGATCGGTTTGTTCGCTTTGCTAAGAGTTAAACCGACCAGATCTAAAACAGCTGTGAGTATGAAAACAGAAACAGATCCCGTACGGATTCTTTCAAAGCAATGGATCTACAGCTAGGGCGAAAAGAAATAAAAACAGGTAATATGAAGTCGGGCTCACAGCAGAACAACCTAAGTTCGACAAGCAGGAACAAGAGTAAGATCTAAGGAAATGAGTGCAGATTTATAGCGACTGCTAAGAGGAACCACAAATCCAGATACAGGTAGCATCATCTGTTCGCAAAGGCTGCTATGGGGATGAGCAAGAAACAGTAGCAGAACGATGAACCCTAGAACAGATCTAAAGCAACAAGAACAGGGGCCTTACCGGGGTCCAATCAGATGCGGAAAGTCGCCGCGGTGCTCTGAACCGATCAGGTTGATGCAGCGGCCGGAGTTGGTGCAGCAGCTGACGGCGGCGGCGGAGCTTTGAGGTCTGGGTCGCGAGGAAGACGAAGAAGGGGAGGAAAGGAAGATGGCCTTCAAGTCCTATTTATAAGGCGCGGCGCGCGTGTCAGGCGCGCGAATCCAGGGGCCGGAGATTTCGGAAGCCCAGCCGTCGCCCTGATTCTTGCGGGTTAGCGAAAGGAGATGGTAGCTTGCGCTGACGTCATATCGGTTTACCACAGCAAGAGGAGACGGAGAAACGACGGTTTAACAAATCACTGGAAGACATTTGAAGACGGAAATTCTTATTAAGGATTGACATGAACCTGTTCAAATCAATCTGGGGCCTAA

TGTTGGGGATGTTGCTATCCGGGGTAAACCGGCCTGCAGGGCCCGGGTTACCTCCGTCAGCAGACCCGAGATGTTAAAACCCAAGGAGAGATAGAAGGGCCTAAGGCCCATAATCGGTTTAAAGGTTTATGGTGTAAACCGGCCTGATGTAAACTTGTATTGTAAGTTAGGAATAGAGAGACCGACCCGGGCACGTTTATTAGCCGGAGGTTGGGACCTTGCAGACCGACGGGCGTCACCCGTGTATATAAGGGGATGACCCGGCGGCGGTTTGGGGACAGAAAAAGAGAACTCGAGGTTTAGGCGAAGCTTATCGCTCCCTAAGCCCTCGAAACCCATCAATCCCATCACAACTAGACGTAGGCTTTTACCTTCATCGAAGGGGCCGAACTAGTATAAAAACCGTGTCCTTGTGTCCACTTTAACCCCTTCAAGCTAACCCGTTGCGATGGCTCCACGACTAAGTCCTCTTACTAGGACATCTGCCGTGACAAATCCACGACA

CATTCCGTTGCCTCCCCATGAACACGACGCAATTTCTTCATTCCGGCCCAGTCGGTTGGCATCTGATGAATAGGATTAGTCGTTGTACGTGTTAGAGACCCTGCCCGTATGTACATACTGTTGG

>Aegilops umbellulata 1-036;

CTCTTGGCGGACTGAACCCTTTGGATCACCGTACTCATAACAGTACGGTGCTCCTCATCGATGGCGGCGCCCTTAAGCGCCTCCAGCAGATTGTCCGGCGCCTCCGGTTGTACGGAGGCCGCCG

TGTCGTGGATTTGTCACGGCAGATGTCCTAGTAAGAGGACTTAGTCGTGGAGCCATCGCAACGGGTTAGCTTGAAGGGGTTAAAGTGGACACAAGGACACGGTTTTTATACTAGTTCGGCCCCTTCGATGAAGGTAAAAGCCTACGTCTAGTTGTGATGGGATTGATGGGTTTCGAGGGCTTAAGGAGCGATATGCTTCGCCTAAACCTCGAATTCTTCTTCTCTGTCCCCAAACCGCCGTCGGGTCGTCCCCTTATATACACGGGTGACGCCCGTCGGTCTGCAAAGTCTCGACCTCCGGCTTATAAACGTGCCCGGGTCGGTCTCTCTATTCCTATCTTACAATACAAGTTGGCCCCAGGCCGGTTTACACCATAAACCTTTAAACCGGTTACAGACCTTGGGCCTTTCTACTTCTCCTCGGGCTTTAACATCTCGGGTCTGTTGACGGCGGTAACCCGGGCCCTGCAGGCCGGTTTACCCCAGATAATAACATCCCCAACA

TTAGGCCCCAGATTGATTTGAACAGGTTCATGTCAATCCTTAATAAGACTCTGTCTTCAAATGTCTTCCAGTGACTTGTTTAAACCGTTGTTTCTCCGTCTCCTCTTGCTGTGGTAAACCGATATGACGTCAGCGCAAGCTACCGTCTCCTTTCGCTAACCCGCAAGAATTAGGGCGACGGCTGGGCTTCCGAAATCTCCGGCCCCTGGATTCGCGCGCCTGACACGCGCGCCGCACCTTATAAATAAGACTTGAAGGCCATCTTCCTTTCCTCCCCTTCTTCGTCTTCCTCGCGACCCAGGCCTCAGAGCTCCGCCGCCGCCGTCAGCTGCTGCACCAACTCCGGCCGCTGCATCAACCTGATCGGATCAGAGCACCGCGGCGACTTTCCGCATCTGTTTGGACCCCGGTAAGGCCCCTGTTCTTGTTGCTTTAGATCTGTTTCTAGGGTTCATCGTCCTGCTACTGTTTCTTGCTCATCCCCATAGCAGCCCTTGCGAACAGATAATGCTACCTGTATCTGGATTTGCAGTTCCTCTTAGCAGTCGCCATAAATCTGCACTCATTTCCTTAGATCTCACTCTTGTTCCTGCTTGTCGAACTTATGTTGTTCTGCTGTGAGCCCGACTTCATATTACCTGTTTTTATTTCTTTTCCGCCCTAGCTGTAGATCCATTGTTTTGAAAGAATCCATACGGGCTCTGTTTCTGCTTTCATACTCACAACTGTTTAGATCTGGTCGGTTTAACTCTTAGCAAAGCGAACAAACCGATCTACACATCCATTAGTCCCCTTGATGAACCGCCGGTTGATATTATTGTAGATAACCTGACTCTATATAAGAGCATCTTCGGTTTACTATCATACTTGCTCTCAAACCGGAATACCTTTGCCTTGTAGACTTCCTCATGGCAAAACAGGTGTATGAATGCAACTGGGTTCCCTCTTGCATCACTGAGACCCAACTAAACAACTTAGTTCTGACTGGTGCTTTGAGTAGCAAAAGTACCATCCATTGGAGGGTTCCAGGAGACGAATGTCCTCCTACCCCGCAGGAGGGAGAAGTGGTGGTGTTTGCGGATCACCTGGCCCGGGGGTTTAACCCACCCGGTTCCAAATTTTATCGGGACGTCTTGGCTAATTTTAGGTTGCGCCCACAAGATGTTGGTCCAAATTCAGTCACGAACATCTGCCATTTTCAAGTACTTTGCGAGGCGTACTTTCAAGAGGAACCCACTGCAGAGCTGTTCCGGGATTTTTTCCATCTGAACCGACGTACTGAATTCACCGATGGCCCTAATATGGAACTCGGCGGTATGTCGATTCAGAAGAGAAAAGAGGTAACTTATCCTCACGCCAAGTTGCATAGTCATCCGCAAGAATGGAATTGTACTTGGTTCTATTGCAAGGATACTTCCCCTGCTGAAGAAAATCCACTGCCCGGTTTCCGTCCAGAGAGACTTAGCAATACTCACCCCTTCCCCCCACGGCTAACTGCCAAAGAAAGGAGTAAATATGCCCCTCAGCTGTCCAAGCTCAGAGCCTTCATGGCTAACGGTTTAACGGGGGTAGACCTTGCTCGTTGCTGGATTTCATGGAGCATCCTGCCCCTTAGCATCCGCTCCGGTTTAATGTGCCAATACACGGGTCGGGTTGATGATCCTTTGAGGCACTGCAACATTCAGATGACGGAGGACGAAGTCGCTGAAGCTGTGAAAAAGATGCTGAATGAACCGGAAAATGTTTGTGCCCAAACCGGCCTGCTTCCATTTTGTGCCACAAACAAACCACCTGCTGTAAGTAACTCCTCTGTTTATAGAATCTTTGATCTCCTTATATATATGTTGCTGACTCCAGTCCTTCCTGAGACAGGGAGATGATCCATTTTGGAGCAAGAAGTTACAAGAGCCGACCGAAAAGGTAATCCGACCCAAGACGAGGGTGAAGAAGCCTGCTCAGAGGAAAAGGAACACCGCTTCCAATACTCTGGATTTAGAGGAGGATGATGTGGGTAATCCGGACCTCGGGGTAGACCTCGATTCAGTTGGTTTGCTTTTTATACGTCTTATTGATGATGATATTTGTCAGGACGACGCTGAAGTGAGCCATGCCGAAGGCCCAGAGGTAACTATTCTTTCTTCTGATGCCGTTCCTTTGCCTTTGAAAAAACCTCGCCAAGCAAACCGGAGAATTAAATTTTCTCACCCTCTTGCTTACTTGGACCCCAAATTTCTTGTGAAGACCCAGCAACATGAAGCTCGACGCACCACCCGACATAGCGGCCAGGTAGTCACCTCCGCCGGTTTACCGAACAGCCTGGTTCGGAAACGCCGCTCAGAGGTCTCCGATTTACGCGCTAGCTTTCCTCCTAAAGCAGGTTTAACTCGTCAGCCTCTAAATCCATCTGACTCCGATTATCAGGTCACTTCCCATTCCTCTTCCGGTGAATCTTCAGCCACTCAACTGCCACCGTTGAAGACAGTTATTGGGTGAGTTGGACCGATCACAACATATCTCTGTATGGGCTTCTTAATCCATCTTATACCGTTTTTTCAATCTTTGGGCTTTTTCCAGGGCCAAACCTAGACCGAGCAAGAAGGCTCGTCTGGATAACCCGGCTGGAGGAACTGCCGCTCCTGAACCGGAGAAAACTCCTGGTGCTGACGCTCTGGGTCGTGAAGATACTCAGAACTATCTATCTCCGCAAGATGACATCTGCGCTGAAGAAAGGACTACCGAACCTGCTAGCCGTACTGATCCGCCTGCCAGTCCTGTTCGGGTTGAGGAATCCATCCCCCCTTCTGTAACTGCTGATAAACCGACAGGGCCACTGCAGAGCAGCGACTCCAAGGATGATGATGTCGTGATTACCGGTATTGGCCGCTCTGAACCGGGGAACACTGCTACTTTGGCCAAGCATACTGCCAAGGACGAAGTCTCGGCCATAAAGGGCAAATGGGACCTTGACTTATCTACGTACGCCTCTTTAAGCGCCCCGGATCTTTATTCTGGGTATCTGAACCGGCTGTACACCAGTCGGGATTATGAGGCTGCCATGATCAAGATGATGAAGGAGAAACTTGAGGTAACTCCCTTGTGCTTCATTCTATTGCAACTTTAGCTTATCAATACTTCCAAACCGAGATGGTAGTCCGGACCAGGCTGCTTTTGACAATTAAGGTCTGGTTTAGAAAACCCAAGGTTTCAACATCGGTCATAACCAGAAAACATCAACAGATAACCATTAGCCCCCAAGTGCCAAGCTTAAATACTTGTATTAATCTTGGGACTTTTTGTAATGGAAAACTTATGTTAAAATCAAATATGCATTAGCCCCCAAGTATCGAGCACATAACTTGCTTGTGTGGTTGATACTTCCAAACCGTGCAATATTTTTTGAACACTGTTGATGACCTGCTGCAGGGGGATTTGAAGACCAAAGAGAACCAAGTCTCGGACCTTCAGGAAGCTTTAAAAGCCCAGCAGATTGAAACTGAAAAAGCAAAGGAGGAATTGACCAAAGCCTTAACTGTTATGGAGCAGCTGAAAGCAAGCCGCAAAAAGGAGCAAACCGATTGGGCTACTGAAAGAGCTCTTTTGACGAAGAGGGCTGAAAATGCCGAAGCTGCTCTTAAACCGGTGGTTGATGAGTTATCCACCGTGAAGCGACAAATACACTCCTTGACCTCTGCCGTCTTTGGTAAACTTTTCGATGCCCTTCTTCCAATTTGTACCTTGTTTGCTACACCCTATGTCCGGTTTGTAATCCACCTTGAAACACTGCAGGCTCACGTATTGGACACTTGGGTTCTGACGTGCGGATGAAACTCAAAGCTGCTTATACTCTGATTGAACAGTTATACACCGGAGCACAAAGAGCTATCACCACTGCATCGCATAACAACCCTGCGCCGTCTCTGATTCAAGACACATTAAAGAGGCTGTCCATGCTTCCGGCCCGGATTGAAGAATTGAAGAGATCAGCTGCTCGAACTGGAGCCATTACTGCCTTGATCCGGGCCAAAGCGTGGGTACCAGATTTCGATCCTGTCGAAGCGGCCCAAGGATACCCCAGCTTGAAGGAAGACAGTTCAGACTTTGGTGAAGACGACTTGAGGGCGATAAACCGGGCGGTGCGCCCCCTAGCTTGTCAATTGGCTGAGGAAGCAGATCTGTCCCGTTACCAAGCCCAATACGACAATCAGAACAAACGAGTATCTGCCCTGATCCCTGAAGCGGGAAACCTTGTTCCTCCAATCCGTAAGCATACTTATGCCCCAGACATTGAACCGTCTTCGCTGATTCATGAAGAAGCCGTCTTTCAAGCGCTAATGGGAATCGACTGGACCACTGCTGATTTCCAGCCAATGGGTAACCAAGATGGAGCTGAAGCGGCGCGAGACGATCCCCAGCCTTCGAACCAAGGCGGCGAACAGGCGTAATCAACGCTCTGGTTCACCTTACATGGTGACGTCTTAATCCGTCAACGAACAATATATTCAGGCACTGCGTTGCCTTGTAATAAGATAGTTAATACCTCTACATTTGAATGTGCCTTCGCGCACGGGTACTGATGCTCTTTCCAATGGCGCTTCCAACTTATGTTTCCTGCCTTTACTGAATATACAATGTGATTTTTCGACCACCTGAACCGTGCTGCGCCTTTGCCAAACCGGGCAATTTGCCTCCGGGTTAAAAAACGGACCAGGCTATGCCAGCTGAATCGGTTTAAGAATGCTTGACGTTCCGAAGAACCAAAAAAGACACCACAATAAAGGCAGAAAAACCAAACCAAATATGAAAAGGTGACGCGATAGTAAACGCATGCGAATAGTGGTTACTATGAACCGTTCAGTAGGCGTGACCATCGTCAAGACAAAACCGAGCCCCCAAGAGGTGTAGCTACGGTTTAGGTCGACTAGGTTCCTAACTGAATCGTGGCAAATATGCCGGCCAAATGGCATTGCATTGGTTCGTCAACCAAGCCCCCAAGTGACTCAATGGCCAAGGGCCGATCAAGTGGCATTGCGTTGGTTCGGACACGACCAAGCCCCCAAATGATTCATTGGCTAAAAAGCCGACCAGAAGGCATTACCATCATCAAGACATGACCGAGTTCCCCTAAGGCGTAATCTACGGTTCAAGTCGATTAGGTCCCCAACTGATTCAGTGGCAAATGTGCCAGCCAGAAGGCATTACCGTCGTCAAGACATGACCAAGCCCCCCGAAGGTGTAGCTACGGTTCAGGCCGACTAGGTCCCCAACTGACTTTATAGGGATATCTACGTTCTCTTTGGTTCGAATACGACCTATGTTTGAACAGCTGTGTTCTCTTTGGTTCGAATACGACCTATGTTTGAACAGGAAGCCCCATGTTTGAATAGCTATGTTCTCTTTGGTTCGAATACGACCTATGTTTGAACAGGAAGCCTCATGTTTGAATAGCTATGTTCTCTTTGGTTCGAATACGACCTATGTTTGAACAGGAAGCCTCATGTTTGAACCAGGATATTGAACCGGATATTAAGAGCTGCACAGCCTTGCCGGAGACATCTTCCCTTGAACCGGCTTTTAAACCGGAATCTATATCTTAAACTTTGTGAAGCGACCTTCAGGGGCAAACACACATGATGTGAAGAACAGCAGAGACCCCGCTTTACTCGAGGCCCGGTTTATTGTATTAATCATAATATATACATGGTCATGATATGTACATAAGCAGAGCCCAGGGCTCATGTATAATAAGGCCGTAGATGAGCTATGTTCCACGGCCTGCTGGTCTCCTCCTCTGATGTGCGTGAATCCTTGCGCTCTCGAATATCAATCAGGTAATATGACCCATTATTCAGATTCTTGCTGACCACAAAAGGTCCTTCCGAAGGCGGGGATAATTTATGCTGATCAGACTGATCCTGGATGAGCTGAAGCACCAAATCCCCCTCCTGAAAGGTTCTGGTCCTAACCCGACGGCTGTGGTAACGGCGAAGATCTTGCTGGTAAATCGCTGAACGGGCAGCGGCCAAGTCACGCTCCTCATCCAACAAGTCAAGAGCGTTCTGCCGCGCCTGCTCATTGTCCGCTTCAACATATGCTGCCACGCGAGGCGAGTCATGTCGTATATCACTTGGGAGAACCGCCTCTGCCCCATAGACCATAAAAAACGACGTAAACCCCGTGGATCTGTTAGGCGTGGTGTTGATGCTCCATAACACAGACGGTAGTTCCTCCACCCAACAACCCGGCGTCCTTTGCAAAGGGACCAGAAGCCGGGGTTTGATACCTCTCAAGATCTCTTGGTTCGCTCGTTCTGCCTGACCATTAGACTGTGGGTGCGCCACCGAAGAAACATCAAGCCGTATATGCTCCCGTGCACAGAACTCTTTCATGGCCCCCTTGGATAGATTGGTACCATTATCTGTGATGATGCTGTGTGGAAATCCAAACCGGAAGATCACCTTCTTTATAAACTGAACCGCCGTGGCAGCATCACACTTACTGACAGGCTCTGCCTCCACCCACTTTGTAAATTTGTCAACCGCCACCAGTAGGTGGGTCTTCTTATCTTTGGAACGCTTAAAAGGCCCAACCATATCCAGCCCCCAAGTCGCGAACGGCCAAGTGATTGGAATCATCCTCAATTCTTGAGCCGGTATGTGCGCTCGTCTGGAAAATTTCTGACAACCATCACATAACCGGACCAGGTCTTCTGCATCAGCATGAGCAGTTAACCAGTAGAAACCGTGGCGGAACGCTTTAGCCACCAATGATTTTGAACCGGCGTGGTGGCCACAATCCCCTTCATGTATTTCTCGTAGCATCTCGCACCCCTCCTTAGGGGATATGCACCGTTGCAGCGCTCCTGACACGCTGCGATGATGTAACTCTCCTTGGAAAATAGTCATAGACTTGGACCGTCGTACTATCTGTCGAGCCAAGCTTTCATCCTCTGGCAACTCACCCCGGTTCATATATGCCAGGTAAGGGATCGTCCAATCCGGGGTGGTGTGAAGAGCCGCCACTAACTGAGCCTCCGGATCAGGAACAGCCAACTCCAGCTCACCGGGCAGCTGTACCGATGGGTGGTACAATACATCCAAGAAAACGTTGGGTGGGACCGGTTTACGCTGAGAGCCCAAGCGACTTAAAGCGTCCGCTGCCTCATTCTTTCTGCGGTCCACATGATCCACTTGATAGCCTTTGAAATGCCCAGCCACCATATCTACTTCACGTCGGTATGCAGCCATAAGCGGATCCTTAGAATCCCAGGTGCCAGATACCTGCTGAGCCACCAGATCCGAATCACCAAAGCACCTAACTCTGCTCAGATTCATCTCTTTAGCCACCCGAAGACCATGAAGCAAAGCCTCATACTCAGCCGCATTATTTGTACAGGGGAACATTAAACGGAGGACATAACAAAACTGATCTCCTCGTGGGGAAGTTAAAACGACTCCAGCCCCCGAGCCTTCCAATTGTCTTGACCCGTCGAAATGAACGGTCCAATAGGTGTTATCTGGCTTCTCCTCAGGCGCTTGTAACTCTGTCCAATCGTTTATGAAATCGACAAGTGCCTGTGACTTTATGGCCGTCCGGGGTACGTACCTTAAATCGTGCGGCCCGAGCTCGATAGCCCACTTGGCAATCCGACCGGTCGCCTCCCGGTTCTGTATAATGTCACCCAAAGGAGCAGAACTGACCACCGTGATTGGGTGTCCTTGAAAATATTGCCTCAGCTTCCGGCTTGCCATAAAAACGCCATATACTAGCTTCTGCCAATGCGGATACCTTTGTTTGGACTCGATAAGTACTTCACTGATATAGTAAACCGGCCGCTGAACCGGATGTTCCTTTCCAGCCTCCTTTCGTTCTACCACCATCGCCACACTGACTGCTCTGGCATTCGCTGCAACATATAACAGGAGTGGCTCTTTATCTACAGGGGCCGCTAATACTGGCGGATTGACTAGCTGCCGCTTCAAGTCCTCAAATGCTTTATCAGCCTCCGGACTCCAGACAAACTGATCCGTCTTCTTGAGCATTTGATACAAGGGGATCGCCTTTTCACCAAGGCGACTGATAAACCGGCTTAACGCCGCAATCCGCCCGGCCATGCGCTGAACATCATTGATGCATTTCGGTTTGGCCAGGGAGGTGATGGCTGTGATCTTTTCTGGATTAGCCTCAATTCCTCTATGGGACACTAAAAATCCCAACAGCTTGCCTGCAGGTACACCAAAAACACATTTAGCCGGGTTGAGCATCATTTGGTACACTCTCAAGTTATCGAAAGTCTCCTTCAAATCGTCAACCAGGGTCTCCTTTTTTCGTGACTTGACCACGATATCATCCACATAAGCATGCACATTACGCCCAATCTGCTCGTGGAGGCAATTTTGCACACATCGTTGATAAGTAGCCTGCGCACTCTTAAGCCCAAAGGGCATAGACACATAGCAGAAGGCTCCAAAGGGAGTAATGAACGCTGTCTTCTCCTGGTCCTTAACTGCCATCTTGATCTGATGATAACCCGAATAAGCATCCAAAAAACTTAAACGCTCGCAACCTGCCGTAGCATCAATGATCTGATCAATCCGGGGGAGAGCAAAAGGATCTGCTGGGCAAGCTTTATTCAAGTCTGTGTAGTCCACGCACATGCGCCAGGTGCCGTTTTTCTTAAGAACTAGCACCGGATTAGCGAGCCATTCGGGATGAAAAACCTCAATGATAAAACCAGCCGCCAGGAGCCTGGCCACTTCCTCACCAATCGCTTTACGTCTTTCTTCATTAAACCGTCGTAAGAACTGCTTTACCGGCTTGTACTTAGGATCCACATTAAGGGTGTGCTCAGCGAGTTGCCTCGGTACACCAGGCATGTCAGATGGCTTCCATGCAAAGATGTCCCGATTCTCACGGATGAACTCGATGAGCGCGCTTTCCTATTTTGGATCCAAGTTTGCACTGATGCTGAACTGCTTGGATGAATCGCCAGGCACGAAGTCAACAAGCTTAGCTTCGGCCGCTGACTTGAACTTCAAGGCTGGATCGTGGTCCGTAGTCGGCTTTTTTAGTGAAGTCATATCCTCCGGATCAACACTGTCTTTGTACTGCTTTAGCTCCTCCGTAGCACAAACCGATTCTGCATAAGTCGCATCTCCTTCCTCGCATTCCAAAGCGATTCGACGGCTCCCATGAACCGTTATAGTGCCCTTGTAACCTGGCATCTTAAGTTGCAGATACACGTAACAGGGCCGTGCCATAAATTTTGCATAAGCTGGCCGTCCGAACAAAGCGTGGTACGGACTTTGGATTTTAACCACTTCGAACGTCAGCGTCTCTGATCTGGAATCGTGACTATCGCCAAATACCACTTCAAGGGCTATCTTACCAACCGGATATGCAGACTTGCCAGGCACCACCCCGTGGAATACTGTATTGGTCGGTTTGAGATCCTTATCTACCAGTCCCATACGACGGAAGGTCTCGTAATACAAGATGTTAATGCTGCTTCCTCCGTCCATGAGCACCTTGGTGATCTTATAACCTCCCACCTGAGGCGCCACCACCAACGCTAACTGACCCGGATTATAAACCCTGGGAGGGTGATCCTCTCGGCTCCATATGATGGGCTGTTCTGACCAGCGCAGATAGTGAGGCGTGGCTGGTTCGATAGCACTAACTGCTCGCCTCTGAAGCTTTCTATCCCGTTTATCCAGGCTTGTAGTAAAGACATGATACTGTCCACCACTCAACTGTTTCGGGTTGTTCTGATAACCTGTTTGCTGCTGCCCGAAACCACTCTGATTACTTTGATTATTTTGATTATTTTGGCTGACCTGTCCGCCCGGATTGCCCTGGAACCCTGGACCGGAACCTGAACCGCCGCCGGAGCTGCGATCATTCTGGAAAGTGTTTGAGCTTTTGAACTCCTTCATGATATAACAATCCTTCCAAAGATGGGTTGCTGGTTCCTCCTTTGTTCCGTGCTTCGGACAGGGCTGGTTTAAGAAAAAGTTCAAACGCTCCGGATTAGGACCAGGATCTGTGCGGAAACTTGGCTGTTTTCCCTTGCGCCGCTGGCCCTTATTCTGTGCACTTGTGTTGGCTACAAAGTCCATGCTCCCGTCCGGTTTACGCTTACCTCCGCCTCCATTACCTGTCGGTCGATGCTGCTGGCCTTTGATGTCGCTATTCTTCCTTCCCTTCCCTGCTTTGTCATCACCAGAGTCAGGATCCTTGGTACTGTCAGAATCCGCATACTTCACTAAAGCGGTCATGAGCGTTCTTATATCTGTACAATCACGCTTCATCCGGCCCAGCTTTAGCTTCAAAGGGCCAAACCGACAGTTGCTTTCCAACAGCACTACAGCGGTGTCTGCGTTGATGCGATCTGATGAGTGCAAGACTTCTGATACTCGTCGTACCCAATGGGTAGTTGATTCCCCTTCTTGCTGAACGCAGGCTGCTAGGTCAACTATTGACATTGGCTGTTTGCACGTGTCCTTGAAGTTCTTGATAAACCGGGCGCGCAATTGGGCCCACGAGCTAATTGAATTAGGCGGCAAACTCTTTAGCCAAGTCCGGGCCGTCCCTTCTAACATCATAGTAAAATACTTGGCACACGCCGCGTCATCCACATCCAGCATCTCCATAGCCATTTCATAGCTCTCCACCCACGTCTCCGGAGGCTGATCTGCCGTGTAGTTTGATACTTTGCGGGGGCCTTTGTAGTCTTTGGGCAGGCGTACGTTGCGCAAGGCGGGGACAAGGCATGGTACTCCCAAAGAACTGGAAACCACCCCCGGTTCAGCCGCAGCTGCTGGGAAGATAGGTGTAATCTGATGAGCCTGACGCTGCGCTGCCAATCCGGCCTCCCGTGCACGGGTGCGATCCACATTCTCATGAGCATTGTCAACGCCCGCCGGATTGTTGCCGCGGAGCGCTTCACGCCTGGCATTACTTGAAACGGCCGGCTCCTCCACGCGTCTGCTATAGCTCCCGCTGAGGCGAGGGGTGGAGTGAATCCGATCACGGCTTTGCGAATATGCTTCTTGCTGGGCCAACGCGGTCCTAAGGAGCTCCTTGACCCGGCGTGTTTCTTCATCCTGCGGTGAATCTCCTTCGATCGGAATGGCTTCCAACCGAGCCGCCGCCGCGACGAGGTTATCCATCGGGTTGGAATAATGACCTGACGGCGTTGGCATGGCCGGTGGCGTATCAGTGTTATGACGAGGCAGGTTTATAATCCGGTGCTGGACCAGGGCGCCGGCCGTCACCTCCGCACGGTTTACCCCCGGGGTGTTGAAGAGGTTTCGAGCGTCGAGGGCGGCGGGCAACCGAGACCGGGATTTTCTCTTCAGGACCTCCTGAGACGCCTTCTGGTCCATCATGAGCCTGTAAGCCTGTGCATCTAAAGCGGCGCGCTCCGCCGTCATCCTGATCTCTTCTGCTGCCAGCTCCGCTTTAGCCCGAGTGATCTGCTCTCTCACTTTGGCAATCTCAGCATTGTGAACCTCCTGATCCACCGGGTTGGCTTCCGGCATGAGCGCTGCTAGTGCGTCAAACAGGTCTGACAAAACTTGGGCTGGTGAACCTGTAGGGCCTCCTGCCCGCGCAGCCGTTGCTGCTGCTGATCCGGAGATCGCTGCTGCTGCAGTTGATGATCGTGGCGCCGCCTGTGTTCCAGCCATGAAGATCCCAACCCGACAGGGCAGATCCGAGGGGTCCGGAATACTGTTGCCATCGGAGCCGCCCTCAATCCGATCATCTTGTAGCTGGTAAAGAGATTCGGTTTCCCCGGTCGACGTTTCATCGCCGGAGTAGATGGCGGTTTCGTCACCAGATTCGGATCCTTCCTCGTAGCTTCCTCCGCGGATGACCCCTACGAAGGCATGCTTTCTGATCGTTTTAGCCAGGGTCGATCTCGTGCACTGAGCTGTCTCGATGAGGTCGGCGCAGACGCCTAACTCGGGGCCCGGTTCTCCGATCTTGCCGATGAAAACATGGATGCCGCCAAAGGGGACCCGGTACCCGTACTCGATCGAGCCGGCGTCGGGGCCCCAGCCTGTGCTGTCGATGTAGAGTTTTCCGCGGCGGCTCTTGGTCATCCGTCCCACAGCGTAACCCTCGAGTCCTTCAAAGCGGCCCTCCAAGAACCTGAAACCATCTCGCGATGGCCCCACGGTGGGCGCCAAC

TGTCGTGGATTTGTCACGGCAGATGTCCTAGTAAGAGGACTTAGTCGTGGAGCCATCGCAACGGGTTAGCTTGAAGGGGTTAAAGTGGACACAAGGACACGGTTTTTATACTAGTTCGGCCCCTTCGATGAAGGTAAAAGCCTACGTCTAGTTGTGATGGGATTGATGGGTTTCGAGGGCTTAAGGAGCGATATGCTTCGCCTAAACCTCGAATTCTTCTTCTCTGTCCCCAAACCGCCGTCGGGTCGTCCCCTTATATACACGGGTGACGCCCGTCGGTCTGCAAAGTCTCGACCTCCGGCTTATAAACGTGCCCGGGTCGGTCTCTCTATTCCTATCTTACAATACAAGTTGGCCCCAGGCCGGTTTACACCATAAACCTTTAAACCGGTTACAGACCTTGGGCCTTTCTACTTCTCCTCGGGCTTTAACATCTCGGGTCTGTTGACGGCGGTAACCCGGGCCCTGCAGGCCGGTTTACCCCAGATAATAACATCCCCAACA

CGCCGGCTCAGAAGGCTTACTCCTCTTGGAAGGAGTTCGCCCACCGTGGACCGGGACTGTCGAGTCCGGCGCGGTGTCCGGCACAAAGCCGGACTCGGAGCCCTGGGGGGTTTCATCCCCTTTA

>Aegilops umbellulata 1-053;

TGCCACAAATAAGTTGCACTATCATTATCAACTCTGCATCTTTTGGCTTCAACATTATGAATATGTGTATCACTACTATCAAGATTCATCAAAAATAGACCACTCTTTAAGGGTGCATGACCAT

TGTCGTGGATTTGTCACGGCAGATGTCCTAGTGAGAGGACTTAGTCGTGGAGCCATCGCAACGGGTTAGCTTGAAGGGGTTAAAAGTGGACACGAGGACACGGTTTTTATACTAGTTCGGCCCCTTCGATGAAGGTAAAAGCCTACGTCTAGTTGTGATGGGATTGATGGGTTTCGAGGGCTTAGGGAGCGATAAGCTTCGCCTAAACCTCGAGTTCTTGTTCTCTGTCCCCAAACCGCCGCCGGGTCATCCCCTTATATACACGGGTGACGCCCGTCGGTCTGCAAAGTCCCAACCTCCGGCTTATAAACGTGCCCGGGTCGGTCTCTCTATTCCTAACTTACAATACAAGTTTACCCCAGGCCGGTTTACACCATAAACCTTTAAACCGATTACAGGCCTTAGGCCCTTCTATTTCTCCTTGGGTTTTAACATCTCGGGTCTGTTGACGGAGGTAACCCGGGCCCTGCAGGCCGGTTTACCCCAGATAGCAACATCCCCAACA

TTAGGCCCCAGATTGATTTGAACAGGTTCATGTCAATCCTTAATAAGAATTTCTGTCTTCAAATGTCTTCCAGTGACTTGTTAAGCCGTCGTTTCTCCGTCTCCTCTTGCTGTGGTAAACCGATATGACGTCAGCGCAAGTTGCCGTCTCATTTCGCTAACCCGCAAGAATCAGGGCGACGGCTGGGCTTCCGAAATCTCCGGCCCCTAGATTCGCGCGCCTGACATGCGCGCCGCGCCTTATAAATAGGACTTGAAGGCCATCTTCCTTTCCTCCCTTTCGTCCCCTTCTTCGTCTTCCTCGCGACCCAGACCTCAGAGCTCCGCCGCCGCCGTCAGCTGCTGCATCAACTCCGGCCGCTGCATCAACCTGATCGGATCAGAGCACCGCGGCGACTTTCCACATCTGTTTTGGATCCCGGTAAAGCCTCTGCTCTTCCGCTTTTAGATCTGTTCTAGGGTTCCAGGGTTTGTCGTCCTGTTACTGTTTCTTGTTCATCCCCGTAGCAGCTTTGCGAACAGATGATGATACTTGTACCGTGATCTGTAGTCCCTCTTAGCAGTCGCCATAAATCTGTACTCATCTCCTTAGATCTCACTCTTGTTCCTGCTTGTCGAACTTATGTTGTTCTGCTGTGAGCCCGACTTCATATTACCTGTTTTTATTTCTTTTTCGCCCTAGCTGTAGATCCATTGCTTTGAAAGAATCCGCACGGGATCTGTTTCTGCTTTCATACTCACAACTGTTTAGATCTGGTCGGTTTAACTCTTAGCAAAGCGAACAAACCGATCTACACATCCATTAGTCCCCTTGATGAACCGCCGGTTGATATTATTGTAGATAACCTGACTCTATATAAGAGCATCTTCGGTTTACAATCATACTTGCTCTCAAACCGGAATACCTTTGCCTTGTAGATTTCCTCATGGCAAAACAGGTGTATGAATGCAACTGGGTTCCCTCTTGCATCACTGAGACCCAACTAAACAACTTAGTTCTGACTGGTGCTTTGAGTAGCAAAAGTACCATCCATTGGAGGGTTCCAGGAGACGAATGTCCTCCTACCCCGCAGGAGGGAGAAGTGGTGGTGTTTGCGGATCACCTGGCCCGGGGGTTTAATCCACCCGGTTCAAAATTTTATCGGGACGTCTTGGCTAATTTTAGGCTGCGCCCACAAGATGTTGGTCCAAATTCAGTCACGAATATCTGCCATTTTCAAGTACTTTGCGAGGCGTACTTCCAAGAAGAACCTACTGTAGAGCTGTTCCGGGACTTTTTCCATTTGAACCGACGTACTGAATTTACCGACGGCCCTAATATGGAACTGGGTGGTATGGCGATTCAGAAGAGGAAAGAGGTGACTTATCCTCACGCCAAGTTGCATAGTCATCCGCAAGAATGGAATTGCACTTGGTTCTATTGCAAGGATACTTCCCCTGCTGAAGAAAATCCACTGCCCGGTTTCCGTCCAGAGAGACTTAGCAACACTCACCCCTTCCCCCCACGGCTAACTGCCAAAGAGAGGAGTAAGTATGCCCCTCAGCTGTCCAAGCTCAGAGCCTTCATGGCTAATGGTTTAACGGGGGTTGATCTTGCCCGTTGCTGGATTTCATGGAGCATCCTGCCCCTTAGCATCCGCTCCGGTTTGATGTGCCAATACACGGGTCGGGTTGATGATCCTTTGAGGCACTGCAACATTCAGCTGACGGAGGACGAAGTCGCTGAAGCCGTGAAAAAGATGCTGAACGAACCGGAACATGTTTGCGCTCGAACCGGCCTGCTTCCATTTTGCGCCACAAACAAACCACCTGCTGTAAGTAACTCCTCCCTGTTTATAGAATTTTTGATCTCCTTATATATATTGCTAACTTCCAGCCCTTTCCGAAACAGGGAGATGATCCGTTTTGGAGCAAGAAGCTTCAGGAGCCAACCGAAAAGATAATCAGACCCAAGACAAGGGTGAAGAAGCCTGCTACAAAGAAAAGGCACACTGCTTCCTGTACCCCCGATTTAGAGGAGGATGATGTGGGTAATCCGGACCTTGGGGTAGACCTTGATTCAGTTGGCTTACTTTTTGTACGTCTTATTGACGATGATATTTGTCAGGACGACGCTGAAGCGAGCAATGCTGAAGGCCCAGAGGTAACTATTCTTTCTTCTGATGCCGTTCCTTTGCCTTTGAAAAACCCTCGCCAAGCAAACCGGAAAATTAAATTTTCTCACCCTCTTGCTTACTTGGATCCCAAATTTCTTGTGAAGACCCAGCAACATGAAGCCCGACGCACCACCCGGCATAGCGGCCAGGTAGTTACCTCCGCCGGTTTACCGAACAGCCCGGTTCGGAAACGCCGCTCAGAGGTCTCCGATTTATGCGCTAGCTTTCCTCCTAAAGCAGGTTTAACTCGTCAGCCTCTAAATCCATCTGACTCCGATTATCAGGTCACTTCCCATTCCTCTTCCGGTGAATCTTCAGCCACTCAACTGCCACCGTTGAAGACAGTTATTGGGTAAGTTGGACTGATCACAACATATCTCTGTATGGGTCTCTTAATCCATCTTATACCGTTTTTCCAATCTTTGGCCTTTTTTTTTTCTCAGGGCTAAACCTAGACCGAGCAAGAAGGCTCGTCTGGATAACCCGGCTGAAGGGACTGCCGCTCCTGAACCGGAGGAAACTCCTGGTGCTGACACTCTGGGTCGTGAAGATACTCAGAATTATCTATCTCCGCAAGATGACACCTGTGCTGAAGAAAGGACTATCGAACCTGCCAGCCGTACTGATCCGCCTGCCAGTCCTGTTCGGGTTGAGGATTCCATCCCCCCTTCTGGAACTGTTGATAAACCGACAGGGCCACTGCAGAGCAGCGATTCGAAGGATGATGATGTTGTGATTACCGGCATTGGCCGCTCTGAACCGGGGAACACTGCCACTTTGGCCAAGCATACTGCCAAGGACGAAATCTCGGCCATAAAGGGCAAATGGGACCTTGACTCCTCTACGTACGCCGCTTTAAGCGCCCCGGATCTTTATTCTGGGTACCTGAACCGACTGTACACCAGCCGGGATTATGAAGCTGGCATGATCAAGATGATGAAGGAGAAACTTGAGGTAACTCCTTTGTGCTTCATTTCATTGCAACTTAGCTTATCAATATTTCCAAACCGAGATGGTAAACCGGATCAGGCTGCCTTGACAATTAAGGTCCGGTTTAGAAAACCCAAGGCTTTAACATAGGTCATAATCAGAAAACATCATCAGATAACCATTAGCCCCCAAGTGCCAAGTTTAAATACTTGTATTAATCTTGGGACTTTTTGTAATGGAAAACTTGCGTTAAAATCAAGTATGCATTAGCCCCCAAGTATCGAGCGCATAACTTGCTTGTGTGGTTGATACTTCCAAACCGTGCAATATTTTCTGAACACTGTTGACGACCTGCTGCAGGGGGATCTGAAGACCAAAGCGAAACAAGTCTCGGACCTTCAGGAAATCTTAAAAACCCAACAGGCTGAAACTGAAAAAACGAAGGACGAATTGGCCCATGCCTTAACCGTTATGGAACAGCTGAAAGAAAGCCACAAAAAAGAACAAGCTGATTGGGCTACTGAAAGAGCTCTTTTGACGAAGAGGGCTGAAAATGCCGAAGCTGCTCTTAAACCGGTGGTCGATGAATTATCCACCGTGAAGCGACAGATACACTCCATGACCTCTGCCGTCTTTGGTAAGCTTTTCACCGCCCTTCTTCCAATTTGTACCTTGTCTGCTACATCCTATGTCCGGTTTGTAATCCATCTTGGAACACTGCAGGCTCACGTATTGGACATTTGGGTTCTGATGTGCGGATGAAACTCAAAGCTGCTTACACTCTGATTGAACAGTTGTACACCGGAGCACAAAGAGCTATCACCACTGCATCGCACAACAACCCTGCGCCGTCTCTGATTCAGGACACGTTAAAGAGGCTGTCCATGCTTCCAGCCCGGATTGAAGAGTTGAAGAGATCAGCTGCTCGAACTGGAGCCATTACTGCCTTGATCCGGGCCAAAGCGTGGGTACCAGATTTCGATCCTGTCGAAGCGGCCCAAGGATACCCCAGCTTGAAGGAAGACGGTTCAGACTTTGGTGAGGACGATTTGAGGGCGATAAACCGGGCGGTGCGCCCCCTAGCTTGTCAATTGGCTGAGGAAGCAGATCTGTCACATTACCAAGCCCACTACGACAACCAGAACAAACGAGTAGCTGCCCCGGTCCCTGAAGCGGGAAACCTTATTCCTCCAAACCGTAAGCATACTTACGCCCCTGACATTGAACCGTCTTTGCTGATTCACGAAGAGGCCGTCTTTCAAGCGCTAATGGGAATCGACTGGACAACTGCTGATTTCCAGCCAATGGGTAACCAAGATAGAGCTGAAGCGGCACAAGACGATCCCCAGCCTTCGAACCGAGGCGGCGAACAGGCGTAATCAACGCTCCGGTTCACCTTAATTGGTGACGTTTTACTCCGTTAAAGAACAATATATTCAGGCACTATGTTGCCTTGTAATAAGATAGTTTAATACCTCTGCATTTGAATGTGCCTTCGCGCACGGGTACTGATGCTCTTTCTAATGACGTTTTCCAACTTTGTTTCCTGTCTTTACTGAGTACAAGATCTTCCGACCCTATGAACCATGCTGCGCCTTTGCCAAACCGGGCAACTTGCCTCTGGGTTAAAAAACGGACCAGGCTATGCCAGCTGAATCGGTTTAAAAAGACTTGACGTTCCGAAGAACAAAAAATACCACATTAAAGGCAGAAAACAACAACCAAATATGAAAAGGCAACGCGGTAGTAAAACGCATGCAAATAGTGGTTACTATGAACCGTTCAGAAGGCGTGACCATCGTCTAGATAAAACCGAGCCCCCAGGAGGTGTAGCTACGGTTTAGGTCGACTAGGTTCCTAACTGAATCGTGGCAAATATGCCGGTCAAATGGCATTGCATTGGTTCGTCAACCAAGCCCCCAAGTGACTCAATGGCCAAGGGCCGATCAAGTGGCATTGCATTGGTTCGGACACGACCAAGCCCCCAAATGATTCATTGGCTAAAAAGCCGACCAGAAGGCATTACCATCATCAAGACATGACCGAGTTCCCCTAAGGCGTAATCTACGGTTCAAGTCGATTAGGTCCCCAACTGATTCAGTGGCAAATGTGCCAGCCAGAAGGCATTACCGTCGTCAAGACATGACCAAGCCCCCCGGAGGTGTAGCTACGGTTCAGGCCGACTAGGTCCCCAACTGACTTTATAGGGTTTAGCTATGTTCTCTTTGGTTCGAACCCGACCTATGTTTGAACAGGAAGCCCCATGTTTGAATAGCTATGTTCTCTTTGGTTCGAATACGACCTATGTTTGAACAGGAAGCCTCATGCTGAATGGCTATGTTCTCTTTGGCTCGAATACGACCTATGTTTGAACAGTAAGCCTCATGCTTGAACCGGGATATTGAACCGGATACTAAGAGCTTCACAGCCCTGTAGGAGACATCTTCCCTTGAACCGGCCTTTAAACCGGAATCTATAATTTGAACTTCTGTGAAGCGACCTCCAGGGGTAAGCACACATGATGTGAAGAACAGAAGAGACCCCGCTTTACTCAAGGCTCCGGTTTATTGTATTAATCATAATATATACATGGTCATGATATGTACATAAACAGAGCCCAGGGCTCATGTATAATAAGGTCGAAGATGAGCTATGTTCCATGGCCTGCTGGTCTCCTCCTTTGATGTGCGTGAATCCTTGCGCTCTCGAATATCAATCAGGTAATATGACCCATTATTCAGATTTTTGCTAACCACAAAAGGTCCCTCCCAAGGCGGGGATAATTTATGCTGGTCAGACTGATCCTGGATGAGCCGGAGCACCAAATCCCCCTCCTGAAAGGTTCTGGTCCTAACCCGACGACTGTGATAACGGCGAAGATCTTGCTGGTAAATCGCTGAACGGGCAGCTGCCAAGTCACGCTCCTCATCCAACAAGTCAAGAGCGTTCTGCCGTGCCTGCTCATTGTCCGCTTCAACATATGCTGCCACGCGAGGTGAGTCATGTCGTATATCACTTGGGAGAACCGCCTCGGCTCCATAGACCATAAAAAATGGCATAAACCCCGTGGATCTGTTAGGCGTGGTGTTGATGCTCCATAATACAGACGGTAGCTCCTCCACCCAACAACCCGGTGTCCTTTGCAAAGGGACCAGAAGCCGGGGTTTGATACCTCTCAAGATCTCTTGGTTTGCTCGTTCTGCCTGACCATTAGACTGTGGGTGCGCTACCGAAGAAACATCAAGCCGTATATGCTCCCGTGCACAGAACTCTTTCATGGCCCCCTTGGATAGATTGGTACCATTATCTGTGATGATGCTGTGCGGAAATCCAAACCGGAAGATCACCTTCTTTATAAACCGAACCGCCGTGGCAGCATCACACTTACTGACAGGTTCTGCCTCCACCCACTTTGTAAATTTGTCAACCGCCACCAGCAGGTGGGTCTTCTTATCTTTGGAACGCTTAAAAGGCCCAACCATATCGAGCCCCCAAGTCGCAAACGGCCAAGTGATTGGAATCATTCTCAATTCTTGGGCCGGTATGTGTGCTCGTCTGGAAAATTTCTGGCATCCATCACATAACCTGACCAGGTCTTCTGCATCAGCATGAGCAGTTAACCAGTAGAAACCGTGGCGGAACGCTTTAGCCACCAATGACTTTGAACCGGCGTGGTGGCCGCAATCCCCTTCATGTATTTCTCGTAATATCTCACACCCCTCCTTAGGGGATACGCACCGTTGCAGCGCTCCTGACACACTGCGATGATGTAACTCTCCTTTGAAAATGGTCATAGACTTGGATCGTCGTACTATCTGTCGAGCCAAGCTTTCATCTTCTGGCAACTCACCCCGGTTCATATATGCCAGATAAGGGAGCGTCCAATCCAGGGTGATGTGAAGAGCCGCCACCAATTGAGCCTCCGGGTCAGGAACAGCCAACTCCAGCTCACCGGGCAGTTGTACCGATGGGTGATACAATACGTCCAAGAAAACATTGGGTGGGACCGGTTTACGCTGAGAGCCCAAGCGGCTTAAAGCGTCCGCTGCCTCATTCTTTCTGCGGTCCACATGGTCCACTTGATAGCCTTTGAAATGCCCGGCCACCATATCTACTTCACGTCGGTACGCCTCCATGAGCGGGTCCTTAGAATCCCAGGTGCCAGATACCTGCTGAGCCACCAGATCCGAATCACCAAAGCACCTAACTCTGCTCAGATTCATCTCTTTAGCCATCCGAAGACCATGAAGCAAAGCTTCATACTCAGCCGCATTATTTGTACAGGGGAACATTAAACGGAGGACATAACAAAACTTATCTCCTCGTGGGGAAGTTAAAACAACTCCAGCCCCCGAGCCTTCCAATTGCCTTGATCCGTCAAAATGAACGGTCCAATAGGTGTTATCTGGCTTCTCCTCAGGCGCTTGTAACTCTGTCCAATCGTTTATGAAATCGACAAGTGCCTGTGATTTTATGGCCGTCCGGGGTACGTACCTTAAATCGTGCGGCCCGAGCTCGATAGCCCACTTGGCAATCCGACCGGTCGCCTCCCGGTTCTGTATAATGTCACCCAAAGGAGCAGAGCTGACCACCGTGATGGGGTGTCCTTGAAAATATTGCCTCAGCTTCCGGCTTGCCATAAAAACACCATATACCAGCTTCTGCCAATGCAGATACCTTTGTTTGGACTCGATAAGTACCTCACTGATATAGTAAACCGGTCGCTGAACCGGATGTTCTTTTCCAGCCTCCTTTCGTTCTACCACCATCGCCACACTAACCGCTCTGGCATTCGCAGCAACATATAACAGGAGTGGTTCTTTATCTACAGGGGCTGCCAAAACTGGCGGATTGATTAGCTGCCGCTTCAAATCCTCAAACGCTTTATCCGCCTCCGCACTCCAGACAAATTGATCCGTTTTCTTGAGCATTTGATACAAGGGGATCGCCTTTTCACCGAGGCGACTGATGAACCGGCTTAAGCTGCAATCCGTCCGGCCATGCGCTGAACATCATTAATGCATTTCGGCTTGGCCAGGGAGGTGATGGCTGTGATCTTTTCTGGATTGGCCTCAATTCCTCTATGGGACACTAAAAATCCCAACAACTTGCCAGCAGGTACACCAAAAACACATTTAGCCGGGTTGAGCATCATTTGGTACACTCTCAAGTTATCGAAAGTCTCCTTCAAATCGTCAACCAGGGTCTCCTTCTTTCGTGACTTGACCACAATATCATCCACATAAGCATGCACATTACGACCAATCTGCTCGTGGAGGCAATTTTGCACACATCGTTGATAAGTAGCCTGCGCACTCTTAAGCCCAAAGGGCATAGACACATAGCAGAAGGCTCCAAAGGGAGTAATGAACGCTGTCTTCTCCTGGTCCTTAACTGCCATCTTGATCTGATGATAACCCGAATAGGCATCCAAAAAACTTAAACGCTCGCAACCTGCCGTAGCATCAATGATCTGATCAATCCGGGGGAGGGCAAAAGGATCCGCTGGGCAAGCTTTATTCAAGTCTGTGTAGTCCACACACATGCGCCAGGTGCCGTTTTTCTTAAGAACTAGCACCGGATTAGCAAGCCATTCGGGATGAAAAACCTCAATGATAAACCCAGCTGCCAGGAGCCTGGCCACTTCCTCACCAATCGCTTTACGTCTTTCTTCGTTAAACCGCCGTAAGAACTGCTTTACCGGCTTATACTTAGGATCCACATTAAGGGTGTGCTCAGCGAGTTGCCTCGGTACACCAGGCATGTCAGACGGCTTCCATGCAAAGATGTCCCGATTCTCACGGATGAACTCGATGAGCGCGCTTTCCTATTTTGGATCCAAGTTTGCACTGATGCTGAACTGCTTGGATGAATCGCCAGGCACGAAGTCAACAAGCTTAGTCTCGGCCGCTGACTTGAACTTCAAAGCCGGATCGTGGTCCGTAGTTGGCTTTTTTAATGAAGTCATATCCTCCGGATCAACACTGTCTTTGTACTGCTTTAGCTCCTCCGTAGCACAAACCGATTCTACATAAGTCGCATCTCCTTCCTCGCATTCTAAAGCGATTCGACGGCTGCCATGAACCGTTATAGTGCCTTTGTAACCCGGCATCTTAAGTTGCAGATACACGTAGCAGGGCCGTGCCATAAATTTCGCATAGGCTGGCCGTCCGAACAAAGCGTGGTACGGACTTTGGATTTTAACCACCTCGAACGTCAGCGTCTCTGATCTGGAATCGTGACTATCGCCAAATACCACTTCAAGGGCTATCTTACCAACCGGATATGCAGACTTGCCAGGCACCACCCCGTGGAACACTGTATTGGTCGGTTTGAGATCCTTATCTACCAGTCCCATACGATGGAAGGTCTCGTAATACAAGATGTTAATGCTGCTCCCTCCGTCCATGAGCACCTTGGTGAGCTTATAACCTCCCACCTGAGGCGCTACCACCAGTGCTAACTGACCCGGATTATACACCCTGGGAGGATGATCTTCTCGGCTCCATATGATAGGCTGTTCCGACCAACGCAGATAGTAAGGCGTGGCCGGTTCGACAGCACTGACTGCCCGCCTCTGAAGCTTTCTATCTCGTTTATCCAAGCTTGTAGTAAAGACATGATACTGTCCACCACTCAACTGTTTCGGGTTGTTCTGATAACCTGACTGCTGCTGTTCGTAACCACTTTGATTACCTTGATTATTTCGGCTGTCCTGTCCGCCCGGATTACCCTGGAACCCTGGACCGGAATTTCCGCCACCGTATCCTGATCCGGAACCTGAACCACCGCCGGAGCTGTGATCATATTGGAAAGTGTTTGAGCTTTTGAATTCCTTCATGATATAACAATCCTTCCAAAGATGGGCTGCTGGTTCCTCCTTTGTTCCGTGCTTCGGACAGGGCTGGTTTAAGAAAAAGTTCAAACGCTCCGGATTAGGACCGGGATGTGCGCGGAAACTTGGCTGTTTCCCCTTACGCCGCTGGCCTTTATCCTGTGCACTTGTGCTGGCCACCAAGTCCATGCTCCCGTCCGGTTTACGCTTTCCTCCACCTCCATTACCTGCCGGCCGATGCTGCTGGCCTTTGGTGTTGCTATTCTTCTTTCCCTTCCCTGCTTTGTCATCACCAGAGTCAGGATCCTTGGTACTGTCAGAATCCGCATACTTCACTAAAGCGGTCATGAGCGTTCCTATATCTGTACAATCACGCTTCATCCGGCCCAGATTTAGCTTCAAAGGGCCAAACCGACAGTTGCTCTCCAACAGCACCACGGCGGTGTCTGCGTTGATGCGATCTGACGAGTGCAAAACTTCTGATACCCGCCGTACCCAATGGGTAGTTGATTCCCCTTCTTGCTGAACGCAGGCTGCTAGGTCAACTATTGACATTGGCTGTTTGCACGTGTCCTTGAAATTCTTGATAAACCGGGCGCGCAATTGGGCCCATGAACTAATTGAATTAGGCGGTAAGCTCTTTAGCCAAGTCCGGGCCGTTCCTTCTAACATCATAGTGAAATACTTGGCACACGCCGTGTCATCCACATCCAGCATCTCCATAGCCATTTCATAGCTCTCCACCCATGTCTCCGGAGGCTGATCTGCCGTGTAGTTTGGTACCTTGCGGGGGCCTTTGAAGTCTTTGGGCAGGCGTACGTTGCGTAAAGCGGGAACAAGGCACGGTACTCCCAAAGAACTGGAAACCACCCCCGGTTCAACCGTAGCTGCTGGGAAGATAGGTGTAAGCTGACGAGCCTGATGCTGCGCTGCTAATCCGGCCTCCCGTGCACGGGTGCGATCCACATTCTCGTGAGTATTGTCGACGCCCGCCGGATTGTTGCCGCGGAGCGCTTCACGCCTGGCGTTACTTGAAATGGCCGGCTCCTCCACGCGCCTACTATAGCTCCTGCTGGGGCGAGGGGTGGAGTGAATCCGATCACGGCTTTGCGAATACGCTTCTTGCTGGGCCAACGCGGTCCTAAGGAGCTCCTTGACCCGGCGTGTCTCTTCATCCTGCGGTGAATCTCCTTCGATCGAAATGGCTTCCAACCGAGCCGCCGCCGCGACGAGGTTATCCATCGGGTTGGAATAATGACCTGACGGTGCTGGCATGGCCGGTGGCGTATCAGTGTTATGACGAGGCAGGTCTATAATCCGGTGCTGAACCGGGGCGCCGGCTGTCGCCTCCGCACGGTTTACCCCCGGGGTGTTGAAGAGATTTCGAGCGTCGAGGGCGGCGGGCAACCGAGACCGGGATTTTCTCTTCAGGACCTCCTGAGACGCCTTCTGGTCCATCATGAGCCTGTAAGCCTGTGCATCTAAAGCGGCGCGCTCCGCCGTCATCCTGATCTCTTCTGCTGCCAGCTCCGCTTTAGCCCGAGTGATCTGCTCTCTCACTTTGGCAATCTCAGCATTGTGAACCTCCTGATCCACCGGGTTGGCTTCCGCCATGAGCGCTGCTAGTGCGTCAAACGGGTCTGACAAAACTTGGGCTGGTGAACCCGTAGGGCCTCCTGCCCGCGCAGCCGTTGCTGCTGCCGATCCGGAGATCGCTGCTGCTGCAGTTGATGATCGTGGCGCCGCCTGTGTCCCAGCCATGAAGATCCCAACCCGATAGGGCAGATCAGAGGGGTCCGGAATACTGTTGCCATCGGAGCCGCCCCCAACCCGATCATCTTGTAGCTGGTAAAGAGATTCGGTCTCCCCGTCGACGTTTCATCGCCAGAGTAGATGGCGGTTTCGTCACCAGATTCGGATCCTTCCTCGTAGCTTCCCCCGTGGATGACTCCCACGAAGGCGTGCTTCCTGGTCGGTTTAGCCAGGGTCGATCTCGTGCACTGAGCTGTCTCGATGAGGTCGGCGCAGATGCCTAACTCGGGGCCCGGTTCTCCGATCTTGCCGATGAAAACATGGATGCCGCCAAAGGGGACCCGGTACCCGTACTCGGTCGAGCCGGCGTCGGGGCCCCAGCCTGTGCTGTCGATGTAGAGTTTTCCGCGGCGGCTCTTGGTCATCCGTCCCACAGCGTAACCCTCGAGTCCTTCAAAGCGGCCCTCCAAGAACCTGAAACCATCTCGCGATAGCCCCACGGTGGGCGCCAAC

TGTCGTGGATTTGTCACGGCAGATGTCCTAGTGAGAGGACTTAGTCGTGGAGCCATCGCAACGGGTTAGCTTGAAGGGGTTAAAAGTGGACACGAGGACACGGTTTTTATACTAGTTCGGCCCCTTCGATGAAGGTAAAAGCCTACGTCTAGTTGTGATGGGATTGATGGGTTTCGAGGGCTTAGGGAGCGATAAGCTTCGCCTAAACCTCGAGTTCTTGTTCTCTGTCCCCAAACCGCCGCCGGGTCATCCCCTTATATACACGGGTGACGCCCGTCGGTCTGCAAAGTCCCAACCTCCGGCTTATAAACGTGCCCGGGTCGGTCTCTCTATTCCTAACTTACAATACAAGTTTACCCCAGGCCGGTTTACACCATAAACCTTTAAACCGATTACAGGCCTTAGGCCCTTCTATTTCTCCTTGGGTTTTAACATCTCGGGTCTGTTGACGGAGGTAACCCGGGCCCTGCAGGCCGGTTTACCCCAGATAGCAACATCCCCAACA

ACCATAAAAGATATTACTCATATAAATAGAACAACCATTATTCTCTGATTTAAATGAATAACAGTCTCGCATCAAACAAGATCCATATATAATGTTCATGCTCAACGCTGGCACCAAATAACAA

>Aegilops umbellulata 1-062;

TGGCCTCGAACCGCCGCCGCCGCGCCAGTTTTCCCTCCGCGCGGCGTCCCCGCGCCGGGATCCGGCCGGATCCGGCGAGATCCCGCCCGGATCCGCCATCTCCGGCAACTCCCGCCTCCTTCCC

TGTTGGGGATGTTGCTATCTGGGGTAAACCGGCCTGCAGGGCCCGGGTTACCACATCAACAAGACCCGAGATGTTAAAGCCCAAGGAGGAGTAGAAAGGCCCAAGATCTATAAACGGTTTAAAGGTTTATGATGTAAACCGGCCTGGGGCCAACTTGTACTGTAAGATAGGAATAGAGAGACCGACCCGGGCACGTTTATAAGCCGGAGGTCGAGACTTTGCAGACCGACGGGCGTCACCCGTGTATATAAGGGGACGACCCGACGACGGTTTGGGGACAGAGAAGAAGAATTCGAGGTTTAGGCGAAGCATATCGCTCCTTAAGCCCTCGAAACCCATCAATCCCATCACAACTAGACGTAGGCTTTTACCTTCATCGAAGGGGCCGAACTAGTATAAAAACCGTGTCCTTGCGTCCGCTTTAACCCCTTCAAGCTAACCCGTTGCGATGGCTCCACGACTAAGTCCTCTTCCTAGGACATCTGCCGTGACAAATCCACGACA

GTTGGCGCCCACCGTGGGGCTATCGCGAGATGGTTTCAGGTTCTTGGAGGGCCGCTTCGAAGGACTCGAGGGTTACGCTGTGGGACGGATGACCAAGAGCCGCCACGGAAAACTCTACATTGACAGCACAGGCTGGGGCCCCGACGCCGGCTCAATCGAGTACGGGTACCGGGTCCCCTTTGGCGGCATCCATGTTTTCATCGGCAAGATCGGAAAACCGGGCCCCGAGTTAGGCGACTGCGCCGACCTCATTGAGACAGCTCAGTGCACGAGATCGACCCTGGCTAAACCGACCAGGAAGCACGCCTTCGTGGGGGTCATCCACGGGGGAAGCTACGAGGAAGGATCCGAATCCGGTGACGAAACCGCCATCTACTCTGGCGATGAAACGTCGACCGGGGAGACCGAATCTCTTTACCAGCTACAAGATGATCGGGTTGGGGGCGGCTCCGATGGCAACAGTATTCCGGACCCCTCTGATCTGCCCTATCGGGTTGGGATCTTCATGGCTGGGACACAGGCGACGCCACGATCATCAACTGCGGCAGCAGCGATCTCCGGATCGGCAGCAGCAACGGCTGCGCGGGCAGGAGGCCCTACCGGTTCACCAGCCCAAGTTTTGTCAGACCTGTTTGACGCACTAGCAGCGCTCATGGCGGAAGCCAACCCGGTGGATCAGGAGGTTCACAATGCTGAGATTGCCAAAGTGAGAGAGCAGATCACTCGGGCTAAAACGGAGCTGGCAGCAGAAGAGATCAGGATGACGGCGGAGCGCGCCGCTTTAGATGCACAGGCTTACAGGCTCATGATGGACCAGAAGGCATCCCAGGAGGTCCTGAAGAGAAAATCCCGGTCTCGGTTGCCCGCCGCCTTCGACGCTCGAAATCTTTTCAACACCCCGGGGGTAAACCGTGCGGAGGCGACAGCCGGCGCCCCGGTTCAGCACCGGATTATAGACCTGCCTCGTCATAACACTGATACGCCACCGGCCATGCCAACGCCGTCAGGTCATTATTCCAACCCGATGGATAACCTCATCGCGGCGGCGGCTCGGTTGGAAGCCATTCCGATCGAAGGGGATTCACCGCAGGATGAAGAGACACGCCGGGTCAAGGAGCTCCTTAGGACTGCGTTGGCCCAGCAAGAAGCGTATTCGCAAAGCCGTGATCGGATTCACTCCACCCCTCGCCCCAGCAGGAGCTATAGTAGGCGCGTGGAGGAGCCGGCCGTTTCAAGTAACGCCAGGCGTGAAGCGCTCCGCGCCAACAATCCGGCGGGCGTCGACAATACTCACGAAAATGTGGACCGCACCCGTGCACGGGAGGCCGGATTAGAAGGGCAGCATCAGGCTCGTCAGCTTACGCCTATCTTCCCCGCAGCTACGGTCGAACCGGGGGTGGTTTCCAGTTCTTTGGGAGTACCGTGCCTTGTTCCCGCTTTACGCAACGTACGCCTGCCCAAAGACTTCAAAGGCCCCCGCAAGGTACCAAACTACACGGCAGATCAGCCTCCGGAGACATGGGTGGAGAGCTATGAAATGGCTATGGAGATGCTGGATGTGGATGACACGGCGTGTGCCAAGTATTTTACTATGATGTTAGAGGGAACGGCCCGGACTTGGCTAAAGAGCTTGCCGCCTAATTCAATTAGCTCATGGGCCCAATTGCGTGCCCGGTTTATCAAGAACTTCAAGGACACGTGCAAACAGCCAATGTCAATAGTTGACCTAGCAGCCTGCGTTCAGCAAGAAGGGGAATCAACTACCCATTGGGTACGACGAGTATCAGAAGTCTTGCACTCATCAGATCGCATCAACGCAGACACCGCTGTAGTGCTGTTGGAAAGCAACTGTCGGTTTGGCCCTTTGAAGCTAAAGCTGGGCCGGATGAAGCGTGATTGCACAGATATAGGAACGCTCATGACCGCTTTAGTGAAGTATGCGGATTCTGACAGTACCAAGGATCCTGACTCTGGTGATGACAAAGCAGGGAAGGGAAGGAAGAATAGCAACGTCAAAGGCCAGCAGCATCGACCGACAGGTAACGGAGGCGGAGGTAAGCGTAAACCGGATGGGAGCATGGACTTTGTAGCCAACGCAAGTGCACAGAATAAGGGCCAGCGGCGCAAGGGAAAACAGCCAAGTTTCCGCACAGATCCTGGTCCTAATCCGGAGCGTTTGAACTTTTTCTTAAACCAGCCCTGTCCGAAGCACGGAACAAAGGAGGAACCAGCAGCCCATCTTTGGAAGGATTGTTATATCATGAAGGAGTTCAAAAGCTCAAACACGTTCCAGAATGATCGCAGCTCCGGCGGCGGTTCAGGTTCCGGTCCAGGGTTCCAGGGTAACCCGGGCGGACAGGTCAGCCAAAATCAGAATAATCAAAGTAATCAAAGTGGTTATCAGAGCAACCCGAAACAGTTGAGTGGTGGACAGTATCATGTCTTTACTACGAGTTTGGATAAACGAGATAGGAAGCTTCAGAGGCGGGCAGTCATTGCTGTCGAACCGGCCACGCCTCACTATCTGCGCTGGTCAGAACAGCCCATCATATGGAGCCGAGAGGATCACCCTCCCAGGGTTTATAATCCGGGTCAGTTAGCATTGGTGGTGGCGCCTCAGGTGGGAGGTTATAAGCTCACCAAGGTGCTCATGGACGGAGGGAGCAGCATTAACATCTTGTATTACGAGACCTTCCGTCGTATGGGACTGGTAGATAAGGATCTCAAACCGACCAATACAGTGTTCCACGGGGTGGTGCCTGGCAAGTCTGCATATCCGGTTGGTAAGATAGCCCTTGAAGTGGTATTTGGCGATAGTCACGATTCCAGATCAGAGACGCTGACGTTCGAAGTGGTTAAAATCCAAAGCCCGTACCACGCTTTGTTCGGACGGCCAGCTTATGCAAAATTTATGGCACGACCCTGCTACGTGTATCTGCAACTTAAGATGCCAGGTTACAAGGGCACTATAACGGTTCATGGGAGCCGTCGAATCTCTTTGGAATGCGAGGAAGGAGATGCGACTTATGCAGAATCGGTTTGTGCTACGGAGGAGCTAAAGCAGTACAAAGACAGTGTTGATCTGGAGGATATGACTTCATTAAAAAAGCCGACTACGGACCACGATCCGGCCTTGAAGTTCAAGTCAGCGGCCGAAACTAAACTTGTTGACTTCGTACCTGGCGATTCATCCAAGCAGTTCAGCATCAGTGCAAACTTGGATCCAAAATAGGAAAGCGCGCTCATCGAGTTCATCCGTGAGAATCGGGACATCTTCGCATGGAAGCCATCTGACATGCCTGGTGTACCGAGGCAACTCGCTGAGCACACCCTTAATGTGGATCCTAAGTACAAGCCGGTAAAGCAGTTCTTACGACGGTTTAATGAAGAAAGACGTAAAGCGATTGGTGAGGAAGTGGCCAGGCTCCTGGCAGCTGGGTTTATCATTGAGGTTTTTCATCCCGAATGGCTCGCTAATCCGGTGCTAGTTCTTAAGAAAAACGGCACCTGGCGCATGTGCGTGGACTACACAGACTTGAATAAAGCTTGCCCAGCAGATCCTTTTGCTCTCCCCCGGATTGATCAGATCATTGATGCTACGGCAGGTTGCGAGCGTTTAAGTTTTTTGGATGCTTATTCGGGTTATCATCAGATCAAGATGGCAGTTAAGGACCAGGAGAAGACAGCGTTCATTACTCCCTTTGGAGCCTTCTGCTATGTGTCTATGCCCTTTGGGCTTAAGAGTGCGCAGGCTACTTATCAACGATGTGTGCAAAATTGCCTCCACGAGCAGATTGGGCGTAATGTGCATGCTTATGTGGATGATATCGTGGTCAAGTCACGAAAGAAGGAGACCCTGGTTGACGATTTGAAGGAAACTTTCGATAACTTGAGAGTGTACCAAATGATGCTCAACCCGGCTAAATGTGTTTTTGGTGTACCTGCAGGCAAGCTGTTGGGATTTTTAGTGTCCCATAGAGGAATTGAGGCCAATCCGGAAAAGATCACAGCCATCACCTCCCTGGCCAAACCGAAATGCATCAATGATGTTCAGCGCATGGCCGGGCGGATTGCGGCGTTAAGCCGGTTTATCAGTCGCCTTGGTGAAAAGGCGATCCCCCTGTATCAAATGCTCAAGAAAACGGATCAGTTTGTCTGGAGTCCGGAGGCTGATAAAGCGTTTAAGGACTTGAAGCAACAACTAGTCAATCCGCCAGTGTTGGCAGCCCCTGTAGATAAAGAGCCACTCCTGCTATATGTTGCAGCGAATGCCAGAGCAGTCAGTGTGGCGATGGTGGTAGAGCGAAAGGAGGCTGGAAAGGAACATCCGGTTCAGCGGCCGGTTTACTATATCAGTGAAGTACTTATCGAGTCCAAACAAAGGTATCCGCATTGGCAGAAGCTGGTATATGGCGTTTTTATGGCAAGCCGGAAGCTGAGGCAATATTTTCAAGGACACCCAATCACGGTGGTCAGTTCTGCTCCTTTGGGTGATATTATACAGAACCGGGAGGCGACCGGTCGGATTGCCAAGTGGGCTATCGAGCTCGGGCCGCACGATTTAAGGTACGTACCCCGGACGGCCATAAAGTCACAGGCGCTTGTCGATTTCATAAACGATTGGACAGAGTTGCAAGCGCCTGAGGAGAAGCCAGATAACACCTATTGGACTGTTCATTTCGACGGGTCAAGACAATTGGAAGGCTCGGGGGCTGGAGTCGTTTTAACTTCCCCGCGAGGAGATAAGTTTTGTTACGTCCTCCGTTTGATGTTCCCCTGTACAAATAATGCGGCGGAGTATGAAGCTTTGCTTCATGGTCTTCGGGTGGCTAAAGAGATGAATTTGAGCAGAGTTAGGTGCTTTGGTGATTCGGATCTGGTGGCTCAGCAGGTATCTGGCACCTGGGATTCTAAGGATCCACTTATGGCTGCGTACCGACGTGAAGTGGACATGGTGGCTGGACATTTCAAAGGTTATCAAGTGGATCATGTGGACCGCAGAAAGAATGAGGCAGCGGACGCTTTAAGTTGCTTGGGCTCTCAGCGTAAACCGGTCCCACCTAATGTGTTCTTGGATGTATTGTACCACCCATCGGTACAGCTGCCCGGTGAGCTGGAGTTGGCTGTTCCTGATCCGGAGGCTCAGTTAGTGGCGGCCCTTCACGCCACCCCGGATTGGACGCTCCCTTACCTGGCATATATGAACCGGGGTGAATTGCCAGAAGATGAAAGCTTGGCTCGACAGATAGTACGACGGTCCAAGTCTATGACCATTTTCCAAGGAGAGTTACATCATCGCAGTGTATCAGGAGCGCTGCAACGGTGCATATCCCCTAAGGAGGGATGCGAGATATTACGAGAAATACATGAAGGGGATTGCGGCCACCACGCCGGTTCAAAATCATTGGTGGCTAAAGCGTTCCGCCACGGTTTCTACTGGTTAACTGCTCATGCTGATGCAGAAGACCTGGTCAGATTATGTGATGGTTGCCAGAAATTTTCCAGACGAGCACACATACCGGCTCAAGAATTGAGGATGATTCCAATCACTTGGCCGTTTGCGACTTGGGGGCTTGATATGGTTGGGCCTTTTAAGCGTTCCAAAGATAAGAAGACCCATCTGCTGGTGGCGGTGGACAAATTTACAAAGTGGGTAGAGGCAGAACCAGTTAGTAAGTGTGATGCAGCCACGGCGGTCCGGTTTATAAAGAAGGTGATCTTCCGGTTTGGTTTTCCACACAGCATCATTACAGATAATGGTACCAATCTATCCAAGGGGGCCATGAAGGAGTTCTGTGCACGGGAGCATATACGGCTTGATGTTTCTTCGGTAGCGCACCCACAGTCTAATGGTCAGGCAGAACGAGCGAACCAAGAGATCTTGAGAGGTATCAAACCCCGGCTTCTAGTCCCTTTGCAAAGGACGCCGGGTTGTTGGGTGGAGGAGCTACCGTCTGTATTATGGAGCATCAACACCACGCCTAACAGATCCACGGGGTTTACGCCGTTTTTTATGGTTTATGGAGCAGAGGCGGTTCTCCCAAGTGATATACGACATGACTCACCTCGCGTGGCAGCATATATTGAAGCGGATAATGAGCAGGCACGGCAGAACGCTCTTGACTTGTTGGATGAGGAGCGTGACTTGGCAGCTGCCCGTTCAGCGATTTACCAGCAAGATCTTCGCCGTTACCACAGCCGTCGGGTTAGGACCAGAACCTTTCAGGAGGGGGATTTGGTGCTTCGGCTCATCCAGGATCAGTCTGATCAGCATAAATTATCCCCGCCTTGGGAAGGACCTTTTGTGGTCAGCAAGAATCTGAATAATGGGTCATATTACCTGATTGATATTCGGGAGCGCAAGGATTCACGCACATCAGAGGAGGAGACCAGCAGGCCGTGGAACATAGCTCATCTACGGCCTTATTATACATGAGCCCTGGGCTCTGCTTATGTACATATCATGACCATGTATATATTATGATTAATACAATCAACCGGAGCCTCGAGTAAAGCAGGGTCTCTGCTGTTCTTCACATCATGTGTGTTTTCCCCTGGAGGTTGCTTCACAAAGTTCAAGATATAGATTCTGTTTTAAAAGCCAGTTCAAGGGAAGATGTCTCCGGCCAGGCTGTGCAGCTCTTAATATCCGGTTCAATATCCCGGTTCAAACATGAGGCTTCCTGTTCAAACATAGGTCGTATTCGAACCAAAGAGAACATAGCTATTCAAACATGGGGCTTCCTGTTCAAACATAGGTCGTATTCGAACCAAAGAGAACGTGGCTATCCCTATAAAGTCAGTTGGGGACCTAGTCGGCCTGAACCGTGGCTACACCTTCGGGGGGCTTGGTCATGTCTTGACGACGGTAATGCCTTCTGGCTGGCACATTTGCCACTGAATCAGTTGGGGACCTAATCGACTTAAACCGTAGATTACACCTTAGGGGAACTCGGTCATGTCTTGATAATGGTAATGCCTTCTGGTCGGCTTTTTAGCCATTGAATCATTTGGGGGCCTGGTCGTGTCCGAACCAACGCAATGCCACTTGATCGGCCCTTGGCCATTGAGTCACTTGGGGGCTTGGTTGACGAACCAATGCAATGCCATTTGGCCGGCATATTTGCCACGATTCAGTTAGGAACCTAGTCGGCTTAAACCGTAGCTACACCTCTTGGGGGCTCGGTTTTGTCTTGACGATGGTCACGCCTACTGAACGGTTCATAATAACCACTATTCGCGTGCGTTTATTATCGCTTTGCCTTTCATATTTGGTTTGGTTTTTTCTGCCTTGATTGTGGTGTCTTTTTTAGTTCTTCGGAACGTCAAGCATCCTTAAACCGATCCAGCTGGCGTAGCCTGGTCCGTTTTTTAACCCGGAGGCAAATTGCCCGGTTTGGCAAAGGCGCAGCACGGTTCAGGTAGTCGAAAAATAGCATTGTGTACTCAGTAAAGGCAGAAAACATAAGATGGAAACATCATTGGAAAGAGCATCAGTACCCGTGCGCGAAGGCACATTCAAATGCAGAAGTATTAAACTATCTTATTACAAGGCAACGTAGTGCCTGAATATATTGTTCACTGACGGATTATAGTTTCGCCAAAGGTGAGCCGGAGCGCTGATTACGCCTGTTCGCCGCCTCGGTTCGAAGGCTGGGGATCATCTTGCGCCGCTTCAGCTCCATCTTGATTACCCATTGGCTGGAAATCAGCAGTGGTCCAGTCGATTCCCATTAGCGCTTGAAAGACGGCTTCTTCATGAATCAGCGAAGACGGTTCAATGTCTGGGGCGTAAGTATGCTTACGGATTGGAGGAACAAGGTTTCCCGCTTCAGGGACCGGGGCAGATACTCGTTTATTCTGGTTGTCGTATTGGGCTTGGTAACGTGACAGATCTGCCTCCTCCGCCAATTGACAAGCTAGGGGGCGCACCGCCCGGTTTATCGCCCTCAAATCGTCTTCCCCAAAGTCTGAACCGTCCTCCTTCAAGCTGGGGTATCCTTGGGCCGCTTCGACAGGATCGAAATCTGGTACCCACGCTTTGGCCCGGATCAAGGCAGTAATGGCTCCAGTTCGAGCAGCTGATCTTTTCAATTCTTCAATCCGGGCTGGAAGCATGGACAACCTCTTTAATGTGTCTTGAATCAGAGACGGCGCAGGGTTGTTATGCGATGCAGTGGTGATAGCTCTTTGTGCTCCGGTGTATAACTGTTCAATCAGAGTATAAGCAGCTTTGAGTTTCATCCGCACGTCAGAACCCAAGTGTCCAATACGTGAGCCTGCAGTGGTTCAAGGTGGATTACAAACCGGACATAGGGTGTGGCAAACAAGGTACAAATTGGAAGAAGGATATCGAAAAAGTTTACCAAAGACGGCAGAGGTCATGGAGTGTATTTGTCGCTTCACGGTGGATAACTCATCAACCACCGGTTTAAGAGCAGCTTCGGCATTTTCAGCCCTCTTCGTCAAAAGAGCTCTTTCAGTAGCCCAATCGGCTTGCTCCTTTTTGCGGCTTGCTTTCAGCTGCTCCATAACAGTTAAGGCTTTGGTCAATTCCTCCTTTGCTTTTTCAGTTTCAATCTGCTGGGCTTTTAAAGCTTCCTGAAGGTCCGAGACTTGGTTCTCTTTGGTCTTCAAATCCCCCTGCGGCAGGTCATCAACAGTGTTCAGAAAATATTACACGGTTTGGAAGTATCAACCACACAAGCAAGTTATGTGCTCGATACTTGGGGGCTAATGCATATTTGATTTTAACATAAGTTTTCCATTACAAAAAGTCCCAAGATTAATACAAGTATTTAAGCTTGGCACTTGGGGGCTAATGGTTAGCTGTTGATGTTTCTCTGGTTATGACCGATGTTGAAACCTTGGGTTTTCTAAACCAGACCTTAATTGTCAAAAGCAGCCTGGTCCGGATTATCATCTCGGTTTGGAAGTATTTGATAACCTAAAGTTGCAATAGAATGAAGCACAAGGGAGTTACCTCAAGTTTCTCCTTCATCATCTTGATCATAGCAGCCTCATAATCCCGACTGGTGTACAGCCGGTTCAGATACCCAGAATAAAGATCCGGGGCGCTTAAAGAGGCGTACGTAGATAAGTCAAGGTCCCATTTGCCCTTTATGGCCGAGGCTTCGTCCTTGGCAGTATGCTTGGCCAAAGTAGCAGTGTTCCCCGGTTCAGAGCGGCCAATACCGGTAATCACGACATCATCATCCTTGGAGTCGCTGCTCTGCAGTGGCCCTGTCGGTTTATCAGCAGTTGCAGAAGGGGGGACGGATTCCTCAACCCGAACAGGACTGGCAGGCGGATCAGTACGACTAGCAGGTTCGGTAGTCCTTTCTTCAGCGCAGATGTCATCTTGCGGAGATAGATAGTTCGGAGTATCTTCACGATCCAGAGCGTCAGCACCAGGAGTTTTCTCCGGTTCAGGAGCGGCAGTTCCTTCAGCCGGGTTATCCAGACGAGCCTTCTTGCTCGGTCTAGGTTTAGCCCTGGAAAAACCCAAAGATTGGTAAAACAGTATAAGATGGATTAAGAGACCCATACAGAGATATGTTGTGATCGGTCCAACTTACCCAATAACTGTCTTCAACGGTGGCAGTTGAGTAGCTGAGGATTCACCAGAAGATGAATGGGAAGTGACCTGATAATCGGAGTCAGATGGATTTAGAGGCTGGCGAGTTAAACCGGCTTTAGGAGGAAAGCTAGCGCATAAATCGGAGACCTCTGAACGGCGTTTCCGAACCGGGCTGTTCGGTAAACCGGCGGAGGTGACTACCTGGCCGCTATGACGGGTGGTGCGTCGAGCTTCATGTTGCTGGGTCTTCACAAGAAATTTGGGATCCAAGTAAGCAAGAGGGTGAGAAAATTTAATTTTCCGATTTGCCTGGCGAGGTTTTTTCAAAGGCAAAGGAACGGCATCAGAAGAAAGAATAGTTACCTCTGGGCCTTCGGCATGGCTCACTTCAGCATCATCCTGACAAATATCATCATCAATAAGACGTACAAAAAGCAAACCAACTGAATCGAGGTCTACCCCGAGGTCCGGATTACCCACATCATCCTCCTCTAAATCCAGAGTATTGGAAGCGGTGTTCCTTTTCCTCTGAGCAGGCTTCTTCACCCTCGTCTTGGGTCGGGTTACCTTTTCGGTCGGCTCTTGTAACTTCTTGCTCCAAAATGGATCATCTCCCTGTCTCAGGAAGGACTGGAGTTAACAACATATATATAAGGAGATCAAAGATTCTATAAACAGAGGAATTACTTACAGCAGGTGGTTTGTTTGTGGCACAAAATGGAAGCAGGCCGGTTTGAGCGCAAACAGCTTCCGGTTCGTTCAGCATCTTCTTCACAGCTTCAGCGACTTCGTCCTCCGTCAGCTGAATGTTGCAGTGCCTCAGAGGGTCATCAACCCGACCCGTGTATTGGCACATCAAACCGGAGCGGATGCTAAGGGGCAGGATGCTCCAGGAAATCCAGCAACGAGCAAGATCAACCCCCGTTAAACCATTAGCCATGAAGGCTCTGAGCTTGGACAGCTGAGGGGCATACTTACTCCTCTCTTTGGCAGTTAGCCGTGGGGGGAAGGGGTGAGTGTTGCTAAGTCTCTCTGGACGGAAACCGGGCAGTGGATTTTCATCGGCAGGGGAAGTATCCTTGCAGTAGAACCAAGTACAATTCCATTCTTGCGGATGACTATGCAACTTGGCGTGAGGATAAGTCACCTCTTTCCTTTTCTGAATCGCCATACCACCCAGTTCCATGTTAGGGCCGTCGGTAAATTCAGTACGTCGGTTCAAATGGAAAAAGTCCCGGAACAGCTCTGCAGTAGGTTCTTCTTGGAAGTACGCCTCGCAAAGTACTTGAAAATGGCAGATATTCGTGACTGAATTTGGACCAACATCTTGTGGGCGCAGCCTAAAATTAGCCAAGACATCCCGATAAAATTTTGAACCGGGTGGATTAAACCCCCGGGCCAGGTGATCCGCAAACACCACCACTTCTCCCTCCTGCGGGGTAGGAGGACATTCGTCTCCTGGAACCCTCCAATGGATGATACTTTTGCTACTCAAAGCACCAGTCAGAACTAAGTTGTTTAGATGGGTCTCAGTGATGCAAGAGGGAACCCAGTTGCATTCATACACCTGTTTTGCCATGAGGAAATCTACAAGGCAAAGGTATTCCGGTTTGAGAGCAAGTATGATTGTAAACCGAAGATGTTCTTATATAGAGTCAGGTTATCTACAGTAATTTCACCGGCGGTTCATCAAGGGGACTAATGGATGTGTAGATCGGTTTTTTTGCTTTGCTAAGAGTTAAACCGACCAGATCTAAGCAGTTGTGAGTATGAAAGCAGAAACAGATCCCGTACGGATTCGTTCAAAGCAATGGATCTACAGCGAGGGTGAAAAACAAATAAAAACAGGTAATATGGAGTCGGGCTCACAGCAGAGCAACATAAGTTCGACAAGCAGGAACAAGAGTGAGATCTAAGGAAATGAATGCAGATTTATGGCGACTGCTAAGAGAAACTACAAATCCAGGTGCAGGTATCGTCATCTGTTCGTAAAGGCTGCTACGGGGATGAGCAAGAAACAGTAGCAGGACGATGAACCCTAGAACAGATCTAAAGCAACAAGGACAGGGGTCTTACCGGGGTCCAAACAGATGCGGAAGGTCGCCGCGGTGCTCTGAACCGATCAGGTTGATGCAGCGGCCGGAGTTGATGCAGCAGTTGACGGCGGCGGCGGAGCTCTGAGGTCTGGGTCGCGAGGAAGACGAAGAAGGAGAGGAAAGTAAGATGGCCTTCAAGTCCTATTTATAAGGCGCGGCGCGTATATCAGGCGCGCGAATCCAGGGGCCGGAGATTTCGGAAGCCCAGCCGTCGCCTTGATTCTTGCGGGTTAGCGAGAGGAGACGGTAGCCTGCGTTGACGTCACATCGGTTTACCGCAGCAGAGGAGACGGAGAAACGACGGTTTAACAAGTCACTGGAAGACATTTGAAGACAGAAATTCTTATTAAGGATTGACATGAACCTGTTCAAATCAATCTGGGGCCTAA

TGTTGGGGATGTTGCTATCTGGGGTAAACCGGCCTGCAGGGCCCGGGTTACCACATCAACAAGACCCGAGATGTTAAAGCCCAAGGAGGAGTAGAAAGGCCCAAGATCTATAAACGGTTTAAAGGTTTATGATGTAAACCGGCCTGGGGCCAACTTGTACTGTAAGATAGGAATAGAGAGACCGACCCGGGCACGTTTATAAGCCGGAGGTCGAGACTTTGCAGACCGACGGGCGTCACCCGTGTATATAAGGGGACGACCCGACGACGGTTTGGGGACAGAGAAGAAGAATTCGAGGTTTAGGCGAAGCATATCGCTCCTTAAGCCCTCGAAACCCATCAATCCCATCACAACTAGACGTAGGCTTTTACCTTCATCGAAGGGGCCGAACTAGTATAAAAACCGTGTCCTTGCGTCCGCTTTAACCCCTTCAAGCTAACCCGTTGCGATGGCTCCACGACTAAGTCCTCTTCCTAGGACATCTGCCGTGACAAATCCACGACA

TTCCCCGTCATCCCCGGCGAGCCCCGCTCCGGCGAGCCTCGGTTCCCGTTGACTTTTCTCGGAGGGTATATTTCTCCAAGTCCCGAAATTATTGTTATTATCATGACATGTTCATCATGCCATA

>Aegilops umbellulata 1-068;

GACTGCTGTCCAGTGATCTACTCCTAGATCACTATTGTACTCCCTTGCCAAACTCAGTGTAGGGTATACAATAGATCTGGTACACAGCATGGCATACTTTATAGAACCTATGGCCAAGGCATAG

TGTCGTGGATTTGTCACGGCAGATGTCCTAGTGAAAGGACTTAGTCGTGGAGCCATCGCAACGGGTTAGCTTGAAGGGGTTAAAAGTGGACACAAGGACACGGTTTTTATACTAGTTCGGCCCCTTCGATGAAGGTAAAAGCCTACGTCTAGTTGTGATGGGATTGATGGGTTTCGAGGGCTTAAGGAGCGATAAGCTTCGCCTAAACCTCGAATTCTTCTTCTCTCTGTCTCACACCGCCGTCGGGTCGTCCCCTTATATACACGGGTGACGCCCGTCGGTCTGCAAAATCCCAACCTCCGGCTTATAAACGTGCCCGGGTTGGTCTCTCTATTCCTATCTTATAATACAAGTTGGCCCCAGGCCGGTTTACACCATAAACCTTTAAACCGGTTATAGGCCTTGGGCCTTCCTATTTCTCCTTGGGCTTTAACATCTCGGGTCCGTTGACGGCGGTAACCCGGGCCCTGCAGGCCGGTTTACCCCAGATAGCAACATCCCCAACA

TTAGGCCCCAGATTGATTTGAACAGGTTCATGTCAATCCTTAATAAGAATTTCTGTCTTCAAATGTCTTCCAGTAACTTGTTAAACCGCTGTTTCTCCGTCCCCTCTTGCTATGGTAAACCGATATGACGTCAGCGCAAGTTACCGTCTCCTTTCGCTAACCCGCAAAAATCAGGGCGACGGCTGGGCTTCCGAAATCTCCGGCCCCTGGATTTGCGCGCCTGACACGCGCGCCGCGCCTTATAAATAGGACTTGAAGGCCATCTTCCTTTCCTCCCCTTCTTCGTCTTCCTCGCGACCCAGACCTCAGAGCTCCGCCGCCGCCGTCAGCTGCTGCATCAACTCCGGCCGCTGCATCAACCTGATCGGATCAGAGCACCGCGGCGACTTTCCACATCTGTTTGGACCCCGGTAAAGCCCCTGTTCTTGTTGCTTTAGATCTGTTCTAGGGTTCATCGTCCTGCTACTGTTTCTTGCTCATCCCCATAGCAGCCTTTGCGAACAGATGATGATACCTGTATCTGGATTTGTAGTTCCTCTTAGCAGTCGCCATAAATCTGCACTCATTTCCTTAGATCTCACTCTTGTCCCTGCTTGTCGAACTTATGTTGTTCTGCTGTGAGCCCGACTTCATACTACTTGTTTTTATTTCTTTTTCACCCTAGCTGTAGATCCATTGCTTTGAAAGAATCCGTATGGGATCTGTTTCTGCTTTCATACTTACAGCCGTTAGATCTGGTCGGTTTAACTCTTAGCAAAGCGAACAAGACGATCCGCACATCCATTAGTCCCCTTGATGAACCGCTGGTTGATATCATTGTGGATAACCTGACTCTATATAAGATCATCTTCGGTTTGTAATCACACTTGTTCTCAAACCGGATTACATTTATCTTGTAGATTTCCGCATGGCAAAACAGGTGTACGAATGCAACTGGGTTCCCTCTTGCATCACCGAGACCCAACTAAACAACTTAGTTCTGACTGGTGCTTTGAGTAGCAAAAATACCATCCATTGGAGGGTTCCAGGAGACGAATGTCCTCCTACCCCGCAGGAGGGAGAAGTGGTGGTGTTTGCGGATCACCTGGCCCGGGGGTTTAACCCACCCGGTTCAAAATTTTATCGGGACGTCTTGGCTAATTTTAGGTTGCGGCCACAAGACGTTGGTCCAAATTCAGTCACGAACATCTGTCATTTTCAAGTACTTTGCGAGGCATACTTTCAAGAGGAACCCACTGTAGAGCTGTTCCGGGACTTTTTCCATCTGAACCGACGTACTGAATTTACCGATGGCCCCAATATGGAACTGGGTGGTATGGCGATTCAGAAGAGGAAAGAGGTGACTTATCCTCACGCCAAATTGCATAGTCATCCGCAAGAATGGAATTGCACTTGGTTCTATTGCAAGGATACTTCCCCTGCTGAAGAAAATCCACTGCCCGGTTTCCGCCCAGAGAGACTTAGCAATACTCACCCCTTCCCCCCACGGCTAACTGCCAAAGAGAGGAGTAAGTATGCCCCTCAGCTGTCCAAGCTCAGAGCCTTCATGGCTAACGGTTTAACGGGGGTAGATCTTGCTCGTTGCTGGATTTCGTGGAGCATCCTGCCCCTTAGCATCCGCTCCGGTTTAATGTGCCAATACACGGGTCGGGTTGATGATCCTTTGAGGCACTGCAACATTCAGCTGACAGAGGACGAAGTCGCTGAAGCTGTGAAGAAGATGCTGAATGAACCGGAACATGTTTGCGCTCGAACCGGCCTGCTTCCATTTTGTGCCACAAACAAACCACCTGCTGTAAGTAACTCCTCTGTTTATAGAATCTTTGATCTCCTTATATATACGTTGTTAACTCCAGTCCTTCCTGAGACAGGGAGATGATCCATTTTGGAGCAAGAAGTTACAAGAGCCGACCGAAAAGGTAACCCGACCCAAGACGAGGGTGAAGAAGCCTGCTCAGAGGAAAAGGAACACCGCTTCCAATACTCCGGATTTAGAGGAGGATGATGTGGGTAATCCGGACCTCGGGGTAGACCTCGATTCAGTTGGTTTGCTTTTTATACGTCTTATTGATGATGATATTTGTCAGGACGACGCTGAAGTGAGCCATGCCGAAGGCCCAGAGGTAACTATTCTTTCTTCTGATGCCGTTCCTTTGCCTTTGAAAAAACCTCGCCAAGCAAACCGGAGAATTAAATTTTCTCACCCTCTTGCTTACTTGGATCCCAAATTTCTTGTGAAGACCCAGCAACATGAAGCTCGACGCACCACCCGACATAGCGGCCAGGTAGTCACCTCCGCCGGTTTACCGAACAGCCCGGTTCGGAAACGCCGCTCAGAGGTCTCCGATTTACGCGCTAGCTTTCCTCCTAAAGCAGGTTTAACTCGTCAGCCTCTAAATCCATCTGACTCCGATTATCAGGTCACTTCCCATTCCTCTTCTGGTGAATCTTCAGCCACTCAACTGCCACCGTTGAAGACAGTTATTGGGTGAGTTGGACCGATCACAACATATCTCTGTATGGGTCTCTTAATCCATCTTATACCGTTTTTTCAATCTTTGGGCTTTTTCCAGGGCCAAACCTAGACCGAGCAAGAAGGCTCGTCTGGATAACCCGGCTGGAGGAACTGCCGCTCCTGAACCGGAGAAAACTCCTGGTGCTGACGCTCTGGGTCGTGAAGATACTCAGAATTATCTATCTCCGCAAGATGACACCTGCGCTGAAGAAAGGATTATCGAACCTGCCAGCCATACTGATCCGCCTGTCAGTCCTGTTCGGGTTGAGGAATCCATCCCCCCTTCTGGAACTGCTGATAAACCGACAGGGCCACTGCAGAGCAGCGATTCCAAGGATGATGATGTTGTGATTACCGGCATCGGTCACTCTGAACCGGGGAACACTGCTACCTTAGCCAAGCATACTGCCAAGGACGAAATCTCGGCCGTGAAGGGCAAATGGGACCTTGACTCCTCTACGTACGCCGCTTTAAGCGCCCCGGATCTTTATTCTGGGTATCTGAACCGACTGTACACCAGTCGGGATTATGAAGCTGGCATGATCAAGATGATGAAGGAGAAACTTGAGGTGACTCCCTTGTGCTTCATTCCTATTGCAACTTTAGCTTATCAATATTTCCAAACCAAAATGGTAGACCGGATCAGGCTGCCTTGACAATTAAAGTCCGGTTTAGAAAACCCCAAGGCTTCAACATAGGTCATAACCAGAAAACATCATTAGATAACTATTAGCCCCCAAGTGCCAAGCTTAAATACTTGTATTAATCTTGGGACTTTTTGTAATGGATAACTTGTGTTAAAATCAAATATGCATTAGCCCCCAAGTATCGAGCACATAACTTGCTTGTGTGGTTGATACTTCCAAACCGTGCAATATTTTCTGAACACTGTTGATGACCTGCTGCAGGGGGATTTGAAGACCAAAGAGAACCAAGTCTCGGACCTTCAGGAAGCTTTAAAAGCCCAGCGGATTGAAACTGAAAAAGCAAAGGAGGAATTGACCAAAGCCTTAACTGTTATGGAGCAGCTGAAAGCAAGCCGCAAAAAGGAGCAAGCCGATTGGGCTACTGAAAGAGCTCTTTTGACGAAGAGGGCTGAAAATGCCGAAGCTGCTCTTAAACCGGTGGTTGATGAGTTATCCACCGTGAAGCGACAAATACACTCCATGACCTCTGCCGTCTTTGGTAAGCTTTTCGATGCCCTTCTTCCAATTTGTACCTTGTTTGCTACACCCTATGTCCGGTTTGTAATCCACCTTGAAACACTGCAGGCTCACGTATTGGACACTTGGGTTCTGACGTGCGGATGAAACTCAAAGCTGCTTATACTCTGATTGAACAGTTATACACCGGAGCACAAAGAGCTATCACCACTGCATCGCATAACAACCCTGCGCCGTCTCTGATTCAAGACACATTAAAGAGGCTGTCCATGCTTCCGGCCCGGATTGAAGAGTTGAAAAGATCAGCCGCTCGAACTGGAGCCATCACTGCCTTGATCCGGGCCAAAGCGTGGGTACCAGATTTCGATCCTGTCGAAGCAGCCCAAGGATACCCCAGCTTGAAGGAAGACGGTTCAGACTTTGGTGAAGATGATTTGAGGGCGATAAACCGGGCGGTGCGCCCCCTAGCTTGTCAATTGGCTGAGGAAGCAGATCTGTCACGTTACCAAGCCCAATACGACAATCAGAACAAACGAGTATCTGCCCCGGTCCCTGAAGCGGGAAACCTTGTTCCTCCAATCCGTAAGCATACTTACGCCCCAGACATTGAACCGTCTTCGCTGATTCATGAAGAAGCCGTCTTTCAAGCGCTAATGGGAATCGACTGGACCACTGCTGATTTCCAGCCAATGGGTAACCAAGATGGAGCTGAAGCGGCGCGAGACGATCCCCAGCCTTCGAACCAAGGCGGCGAACAGGCGTAATCAACGCTCCGGTTCACCTTACTTGGTGACGTCTTAATCCGTCAACGAACAATATATTCAGGCACTACGTTGCCTTGTAATAAGATAGTTTAATACCTCTACATTTGAATGTGCCTTCGCGCACGGGTGCTGATGCTCTTTTCAATGACGTTTCCAACTTATGTTTCCTGCCTTTACTGAGTACACAATGTGATTTTTCGACCACCTGAACCGTGCTGCGCCTTTGCCAAACCGGGCAATTTGCCTCCGGGTTAAAAAACGGATCAGGCTATGCCAGCTGAATCGGTTTTAAGAATACTTGGCGTTCCGAAGAACCAAAAAACACCACAATAAAGGCAGAAAACAAAACCAAATATGAAAAGGCAACGCGACAGTAAAACGCATGCGAATAGTGGTTACTATGAACCGTTCAGAAGGCGTGACCATCGTCAAGACAAAACCGAGCCCCCAAGAGGTGTAGCTACGGTTTAGGTCGACTAGGTTCCTAACTGAATCGTGGCAATTATGCCAGCCAAATGGCATTGCATTGGTTCGTCAACCAAGCCCCCAAGTGACTCAATGGCCAAGGGCCGATCAAGTGGCATTGCGTTGGTTCGGACACGACCAAGCCCCCAAATGATTCATTGGCTAAAAAGCCGACCAGAAGGCATTACCATCATCAAGACATGACCGAGTTCCCATAAGGCGTAATCTACGGTTCAAGTCGATTAGGTCCCCAACTGATTCAGTGGCAAATATGCCAGCCAGAAGGCATTACCATCGTCAGGACATGACCAAGCCCCCCGGTGGTGTAGCTACGGTTCAGGCCGACTAGGTCCCCAACTGACTTTATAGGGTTTAGCTATGTTCTCTTTGGTTCGAACCCGACCTATGTTTGAACAGGAAGCCCCATGTTTGAATAGCTATGTTCTCTTTGGTTCGAATACGACCTATGTTTGAACAGGAAGCCTCATGCTGAATGGCTATGTTCTCTTTGGCTCGAATACGGCCTATGTTTGAACAGTAAGCCTTATCCTTGAACCGGGATATTGAACCGGATATTAAGAGCTTCACAGCCTTGTAGGAGACATCCTCCCTTGAACCGGCTTTTAAACCGGAGTCTATAATTTGAACTTCTGTGAAACGACCTCCAGGGGTAAGCACACATGATGTGAAGAACAGTAGAGACCCCGTTTTACTCGAGGCTCCGGTTTATTGTATTAATCATAATATATACATGGTCATGATATGTACATAAACAGAGCCCAGGGCTCATGTATAATAAGGTCGAAGATGAGCTATGTTCCACGGCCTGCTGGTTTCCTCCTCTGATGTGCGTGAATCCTTGCGCTCTCGAATATCAATCAGGTAATATGACCCATTATTCAGATTCTTGCTGACCACAAAAGGTCCCTCCCAAGGCGGGGATAATTTATGCTGATCAGATTGATCCTGGATGAGCCGAAGCACCAAATCCCCCTCCTGAAAGGTTCTGGTCCTAACCCGACGGCTGTGGTAACGGCGAAGATCTTGCTGGTAAATCGCTGAACGGGCAGCTGCCAAGTCACGCTCCTCATCCAACAAGTCAAGAGCGTTCTGCCGTGCCTGCTCATTATCCGCTTCAACATATGCTGCCACGCGAGGTGAGTCATGTCGTATATCACTTGGGAGAACCGCCTCTGCTCCATAAACCATAAAAAACGGCGTAAACCCCGTGGATCTGTTAGGCGTGGTGTTGATGCTCCATAATACAGATGGTAGCTCCTCCACCCAACAACCCGGCGTCCTTTGCAAAGGGACCAGAAGCCGGGGTTTGATACCTCTCAAGATCTCTTGGTTCGCTCGTTCTGCCTGACCATTAGACTGTGGGTGCGCTACCGAAGAAACATCAAGCCGTATATGCTCCCGTGCACAGAACTCCTTCATGGCCCCCTTGGATAGATTGGTACCATTATCTGTGATGATGCTGTGTGGAAAGCCAAACCGGAAGATCACCTTCTTTATAAACCGAACCGCCGTGGCTGCATCACAATTACTGACAGGTTCTGCCTCTACCCACTTTGTAAATTTGTCAACCGCCACCAGTAGGTGGGTCTTCTTATCTTTGGAACGCTTAAAAGGCCCAACCATATCAAGCCCCCAAGTCGCGAACGGCCAAGTGATTGGAATCATCCTCAATTCTTGAGCCGGTATGTGCGCTCGTCTGGAAAATTTCTGGCAACCATCACATAACCGGACCAGGTCTTCCGCATCAGCATGAGCAGTTAACCAGTAGAAACCGTGGCGGAACGCTTTAGCCACCAATGATTTTGAACCGGCGTGGTGGCCGCAATCCCCTTCATGTATTTCTCGTAGTATCTCGCACCCCTCCTTAGGGGATATGCACCGTTGCAGCGCTCCTGACACGCTGCGATGATGTAACTCTCCTTGGAAAATGGTCATAGACTTGGACCGTCGCACTATCTGTCGAGCCAAGCTTTCATCCTCTGGCAACTCACCCCGGTTCATATATGCCAGGTAAGGGAGCGTCCAATCCGGGGTGGCGTGAAGAGCTGCCACTAACTGAGCCTCCGGATCAGGAACAGCCAACTCCAGCTCACCGGGCAGCTGTACCGATGGGTGGTACAATACATCCAAGAAAACATTGGGTGGGACCGGTTTACGCTGAGAGCCCAAGCGACTTAAAGCGTCCGCTGCCTCATTCTTTCTGCGGTCCACATGATCCACTTGATAACCTTTGAAATGCCCAGCCACCATATCCACTTCACGTCGGTACGCAGCCATAAGCGGATCCTTAGAATCCCAGGTGCCAGATACCTGCTGAGCCACCAGATCCGAATCACCAAAGCACCTAACTCTGCTCAAATTCATCTCTTTAGCCACCCGAAGTCCATGAAGCAAAGCTTCATACTCAGCCGCATTGTTTGTACAGGGGAACATTAAACGGAGGACATAACAAAACTTATCTCCTCGTGGGGAAGTTAAAACGACTCCAGCCCCCGAGCCTTCCAATTGTCTTGACCCGTCGAAATGAACGGTCCAATAGGTGTTATCTGGCTTCTCCTCAGGCGCTTGTAACTCTGTCCAATCGTTTATGAAATCGACAAGTGCCTGTGATTTTATGGCCGTCCGGGGTACGTACCTTAAATCGTGCGGCCTGAGCTCGATAGCCCACTTGGCAATCCGACCGGTCGCCTCCCGGTTCTGTATAATGTCACCCAAAGGAGCAGAACTGACCACCGTGATTGGGTGTCCTTGGAAATATTGCCTCAGCTTCCGGCTTGCCATAAAAACGCCATATACCAGCTTCTGCCAATGCGGATACCTTTGTTTGGACTCGATAAGTACTTCACTGATATAGTAAACCGGTCGCTGAACCGGATGTTCCTTTCCAGCCTCCTTTCGTTCTACCACCATCGCCACACTGACTGCTCTGGCATTCGCTGCAACATATAACAGGAGTGGCTCTTTATCTACAGGGGCTGCCAACACTGGCGGATTGACTAGTTGTCGCTTCAAGTCCTCAAACGCTTTATCGGCCTCCGGACTCCAGACAAACTGATCCGTCTTCTTGAGCATTTGATACAAGGGGATCGCCTTTTCACCAAGGCGACTGATAAACTGGCTTAACGCCGCAATCCGCCCGGCCATGCGCTGAACATCATTGATGCATTTCGGTTTGGCCAGGGAGGTGATGGCTGTGATCTTTTCTGGATTGGCCTCAATTCCTCTATGGGACACTAAAAATCCCAACAGCTTGCCTGCAGGTACACCAAAAACACATTTAGCCGGGTTGAGCATCATTTGGTACACTCTCAAGTTATCAAAAGTCTCCTTCAAATCGTCAACCAGGGTCTCCTTCTTTCGTGACTTGACCACGATATCATCCACATAAGCATGCACATTACGCCCAATCTGCTCGTGGAGGCAATTTTGCACACATCGTTGATAAGTAGCCTGCGCACTCTTAAGCCCAAAGGGCATAGACACATAGCAGAAGGCTCCAAAGGGAGTAATGAACGCTGTCTTCTCCTGGTCCTTAACTGCCATCTTGATCTGATGATAACCCGAATAAGCATCCAAAAAACTTAAACGCTCGCAACCTGCCGTAGCATCAATGATCTGATCAATCCGGGGGAGAGCAAAAGGATCTGCTGGGCAAGCTTTATTCAAGTCTGTGTAGTCCACGCACATGCGCCAGGTGCCGTTTTTCTTAAGAACTAGCACCGGATTAGCGAGCCATTCGGGATGAAAAACCTCAATGATAAACCCAGCTGCCAGGAGCCTGGCCACTTCCTCACCAATCGCTTTACGTCTTTCTTCGTTAAACCGCCGTAAGAACTGCTTTACCGGCTTGTACTTAGGATCCACATTAAGGGTGTGCTCAGCGAGTTGCCTCGGTACACCAGGCATGTCAGATGGCTTCCATGCAAAGATGTCCCGATTCTCACGGATGAACTCGATGAGCGCGCTTTCCTATTTTGGATCCAAGTTTGCACTGATGCTGAACTGCTTGGATGAATCGCCAGGTACGAAGTCAACAAGCTTAGTTTCGGCCGCTGACTTGAACTTCAAGGCCGGATCGTGGTCCGTAGTTGGCTTTTTTAATGAAGTCATATCCTCCGGATCAACACTGTCTTTGTACTGCTTTAGCTCCTCCGTAGCACAAACCGATTCTGCATAAGTCGCGTCTCCTTCCTCGCATTCCAAAGCGATTCGACGGCTCCCATGAACCGTTATAGTGCCTTTGTAACCTGGCATCTTAAGTTGCAGATACACGTAGCAGGGCCGTGCCATAAATTTTGCATAAGCTGGCCGTCCGAACAAAGCGTGGTACGGGCTTTGGATTTTAACCACTTCGAACGTCAGCGTCTCTGATCTGGAATCGTGACTATCGCCAAATACCACTTCAAGGGCTATCTTACCAACCGGATATGCAGACTTGCCAGGCACCACCCCGTGGAACACTGTATTGGTTGGTTTGAGATCCTTATCTACCAGTCCCATACGACGGAAGGTCTCGTAATACAAGATGTTAATGCTGCTCCCTCCGTCCATGAGCACCTTGGTGAGCTTATAACCTCCCACCTGAGGCGCCACCACCAATGCTAACTGACCCGGATTATAAACCCTGGGAGGGTGATCCTCTCGGCTCCATATGATAGGCTGTTCTGACCAGCGCAGATAGTGAGGCGTGGCTGGTTCGATAGCACTAACTGCTCGCCTCTGAAGCTTTCTATCCCGTTTATCCAGGCTTGTAGTAAAGACATGATACTGCCCACCACTCAACTGTTTCGGGTTGTTCAGATAACCTGTTTGCTGCTGCCCGAAACCACTCTGATTACTTTGATTATTTTGGCTGACCTGTCCGCCCGGATTGCCCTGGAACCCTGGACCGGAACCTGAACCGCCGCCGGAGCTGCGATCATTCTGGAAAGTGTTTGAGCTTTTGAACTCCTTCATGATATAACAATCCTTCCAAAGATGGGTTGCTGGTTCCTCCTTTGTTCCGTGCTTCGGACAGGGCTGGTTTAAGAAAAACTTCAAACGCTCCGGATTAGGACCAGGATCTGTGCGGAAACTTGGCTGTTTTCCCTTGCGCCGCTGGCCCTTATTTTGTGCACTTGTGTTGGCTACAAAGTCCATGCTCCCGTCCGGTTTACGCTTACCTCCGCCTCCATTACCTGTCGGTCGATGCTGCTGGCCTTTGACGTTGCTATTCTTCCTTCCCTTCCCTGCTTTGTCATCACCAGAGTCAGGATCCTTGGTACTGTCAGAATCCGCATACTTCACTAAAGCGGTCATGAGCGTTCCTATATCTGTGCAATCACGCTTCATCCGGCCCAGCTTTAGCTTCAAAGGGCCAAACCGACAGTTGCTTTCCAACAGCACTACAGCGGTGTCTGCGTTGATGCGATCTGATGAGTGCAAGACTTCTGATACTCGTCGTACCCAATGGGTAGTTGATTCCCCTTCTTGCTGAACGCAGGCTGCTAGGTCAACTATTGACATTGGCTGTTTGCACGTGTCCTTGAAGTTCTTGATAAACCGGGCGCGCAATTGGGCCCACGAGCTAATTGAATTAGGCGGCAAACTCTTTAGCCAAGTCCAGGCCGTTCCTTCTAACATCATAGTAAAATACTTGGCACACGCCGCGTCATCCACATCCAGCATCTCCATAGCCATTTCATAGCTCTCCACCCACGTCTCCGGAGGCTGATCTGCCGTGTAGTTTGGTACTTTGCGGGGGCCTTTGAAGTCTTTGGGCAGGCGTACGTTGCGCAAGGCGGGAACAAGGCACGGTACTCCCAAAGAACTGGAAACCACCCCCGGTTCAGCCGCAGCTGCTGGGAAGATAGGTGTAATCTGATGAGCCTGATGCTGCGCTGCCAATCCGGCCTCCCGTGCACGGGTGCGATCCACATTCTCGTGAGCATTGTCAACGCCCGCCGGATTGTTGCCGCGGAGTGCTTCACGCCTGGCATTACTTGAAACGGCCGGCTCCTCCACGCGCCTACTATAGCTCCCGCTGGGGCGAGGGGTGGAGTGAATCCGATCACGGCTTTGCGAATATGCTTCTTGCTGGGCCAACGCGGTCCTAAGGAGCTCCTTGACCCGGCGTGTTTCTTCATCCTGCGGTGAATCTCCTTCGATCGGAATGGCTTCCAACCGAGCCGCCGCCGCGACGAGGTTATCCATCGGGTTGGAATAATGACCTGACGGCGTTGGCATGGCCGGTGGCGTATCAGTGTTATGACGAGGCAGGTTTATAATCCGGTGCTGAACCGGGGCACCGGCCGTCACCTCCGCACGGTTTACCCCCGGGGTGTTGAAGAGGTTTCGAGCGTCGAGGGCGGCGGGCAACCGAGACCGGGATTTTCTCTTCAGGACCTCCTGAGACGCCTTCCGGTCCATCATGAGCCTGTAAGCCTGTGCATCTAAAGCGGCGCGCTCCGCCGTCATCCTGATCTCTTCTGCTGCCAGCTCCGCTTTAGCCCGAGTGATCTGCTCTCTCACTTTGGCAATCTCAGCATTGTGAGCCTCCTGATCCACCGGGTTGGCTTCCGCCATGAGCGCTGCTAGTGCGTCAAACAGGTCTGACAAAACTTGGGCTGGTGAACCTGTAGGGCCTCCTGCCCGCGCAGCCGTTGCTGCTGCCGATCCGGAGATCGCTGCTGCTGCAGTTGATGATCGTGGCGCCGCCTGTGTTCCGGCCATGAAGATCCCAACCCGACAGGGCAGATCCGAGGGGTCCGGAATACTGTTGCCATCGGAGCCGCCCTCAATCCGATCATCTTGTAGCTGGTAAAGAGATTCGGTTTCCCCGGTCGACGTTTCATCGCCGGAGTAGATGGCGGTTTCGTCACCAGATTCGGATCCTTCCTCGTAGCTTCCTCCGCGGATGACCCCCACGAAGGCGTGCTTTCTGATCGGTTTAGCCAGGGTCGATCTCGTGCACTGAGCCGTCTCGACGAGGTCGGCGCAGACGCCTAGCGCGGGGCCCGGTTCTCCGATCTTGCCGATGAAAACATGGATGCCGCCAAAGGGGACCCGGTACCCGTACTCGATCGAGCCGGCGTCGGGGCCCCAGCCTGTGCTGTCGATGTAGAGCTTTCCGCGGCGGCTCTTGGTCATCCGTCCCACAGCGTAACCCTCGAGTCCTTCAAAGCGGCCCTCCAAGAACCTGAAACCATCTCGCGATAGCCCCACGGTGGGCGCCAAC

TGTCGTGGATTTGTCACGGCAGATGTCCTAGTGAAAGGACTTAGTCGTGGAGCCATCGCAACGGGTTAGCTTGAAGGGGTTAAAAGTGGACACAAGGACACGGTTTTTATACTAGTTCGGCCCCTTCGATGAAGGTAAAAGCCTACGTCTAGTTGTGATGGGATTGATGGGTTTCGAGGGCTTAAGGAGCGATAAGCTTCGCCTAAACCTCGAATTCTTCTTCTCTCTGTCTCACACCGCCGTCGGGTCGTCCCCTTATATACACGGGTGACGCCCGTCGGTCTGCAAAATCCCAACCTCCGGCTTATAAACGTGCCCGGGTTGGTCTCTCTATTCCTATCTTATAATACAAGTTGGCCCCAGGCCGGTTTACACCATAAACCTTTAAACCGGTTATAGGCCTTGGGCCTTCCTATTTCTCCTTGGGCTTTAACATCTCGGGTCCGTTGACGGCGGTAACCCGGGCCCTGCAGGCCGGTTTACCCCAGATAGCAACATCCCCAACA

CATAGGGAATGACTTTCATTCTCCTTCTATCTTCTGCCGTGGTCGAGTTTTGAGTCTTTACTCAACTTCATACCTTGTAACACAGGAAAGAACTCTTTCTTTGACTGTTCCATTTTGAACTACT

>Aegilops umbellulata 1-071;

AGTAGAGTAAGTATTTCCCACAGTTTTTGAGAACCAAGGTATCAATCCAGTAGGAGGCTATGCGCGAGTCCCTCGTACCTACACAAAACAAATAGCTCAATGTAACCAACGCGATTAGGGGTTG

TGTCGTGGATTTGTCACGGCAGATGTCCTAGTGGGAGGACTTAGTCGTGGAGCCATCGCAACGGGTTAGCTTGAAGGGGTTAAAAGTGGACACAAGGACACGGTGTTTATACTAGTTCGGCCCCTTCGATGAAGGTAAAAGCCTACGTCTAGTTGTGATGGGATTGATGGGTTTCGAGGGCTTAGGGAGCGATCAGCTTCGCCTAAACCTCGAGTTCTTCTTCTCTGTCTCCAAACCGCCATCGGGTCGTCCCCTTATATACACGGGTGACGCCCGTCGGTCTGCAAAGTCCCGACCTCCGGCTTACAAACGTGCCCGGGTCGGTCTCTCTATCCCTAACTTACAATGCAAGTTTACCTCAGGCCGGTTTACACCGTAAACCTCTAAACCGATTATAGGCCTTGGGCCCTTCTATTTCTCCTTGGGCTTTAACATCTCGGGTCCATTGACGGAGGTAACCCGGGCCCTGCAGGCCGGTTAACCCCAATTAGCCATATCCCCAACA

TTAGGCCCCAGATTGATTTGAACAGGTTCATGTCAGTCCTTAAAAAATTTCTGTCTTCAAATGTCTTCCAGTAACTGTTGAACCGCTATTTGCTTCGTCTCCTTTTGCTGCGGTAAACCGATATGACGTCAGTACAAAGTTACCGTCTCCTTTTGCTAATCCGCAATAATCAGGGCGACGGCTGGGCTCCCGAAATCTCCGGCCCCTGGATTCGCGCGCCTGGCACGCACGCCGCGCCTTATAAATAGGACTTGAAGGCCATCTTCCTTTCCTCCGTTTCGCCCCCTTCTTCGTCTTCCTCGCGACCCAGACCTCGGAGCTCCGCCGCCGCCGTCAGCTACTGCCTCCACTCCGGCCGCTGCATCAACCTGATCGGACCAGAGCACCGCGGCGACCTTCCACCTCCGTTTGGATCCCGGTTAGACCCCTGCTCTTCTCGCTTCAGATCTGTTCTAGGGTTCGTCATCCTGCCACTGTTCCTTGTTCATCTCCATAGCAACTTTTGCGAACAGATGATGATACCTATATTGTGAACTTGTAGTCCCTCTCAGTGTTCGCCAGAAATCTGTACTCATCTCCTTAGATCCCACTCTTTTCCCTGTTTGTCGAACCTATGTTGTTCTGCTGTGAGCCAAACCTCGTATTTTTACTTCTTTTTCGCCCTAGCTGTAGATCCATTGCTTTGAAAGAATCCGTACGGGATCTGTTTCTGCTTTCATATTCACAACTGTTAGATCTGGTCGGTTTAACTTCTAGCAAAGCGAACAAACCAATCCACACATCCATTAGTCCCCTTGATGAACCGCTGGATGATATCATTGTAGATAACCTTGCTCTATATAAGAGCATCTTCGGTTTACATTCACACTTTGTTCTCAAACCGGTACACCTTTGTCTTGTAGATTCTCTCATGGCAAAACAGGTGTACGAATGCAACTGGGTTCCCTCTCGCATCACTGAGACCCAACTGAACAACTTAGTTCTGACTGGCGCTTTGAACAGCAAAAATGCCATCCATTGGAGGGTTCCAGGAAACGAATGTCCTCCTACCCCACAGAAGGGAGAAGTGGTGGTGTTTGCGGATCACCTGGCCCGGGGTTTTAATCCACCCGGTTCAAAATTTTATCGGGACGTCCTGGCTAACTTTAAGTTGCGCCCACAAGATGTTGGTCCAAACTCAGTCACGAATATCTGCCATTTTCAAGTGCTTTGCGAGGCGTACTTCCAGGAGGAACCTACTGTAGAGCTATTCCGGGACTGCTTTCATTTGAACCGACGTACTGAATTCACCGACGGCCCTAATATGGAACTGGGTGGTGTGACGATTCAAAAGAGGAAAGAAGTGACTTATCCTCATGCCAAGCTGCATAGTCATCCGCAAGAATGGAATCACACTTGGTTCTATTGCAAGGATACTTCCCCTGCTGAAGAAAATCCACTGCCCGGCTTCCGTCCGGAGAGACTTAGCAACACACACCCCTTCCCCCAAAGGCTAACCGCCAAAGAGAGGAGTAAGTACGCCCCTCAGCTGTCCAAGCTCAGAGCCTTCATGGCTAACGGTTTGACGGGGGTAGACCTTGCCCGCTGCTGGATTTCATGGAGCATCCTGCCTCTTAGCATCCGCTCCGGTTTAATGTGCCAATACACGGGCCGGGTTGATGATCCTTTGAGGCACTGCAACATTCAGCTAACGGAAGACGAAGTCACCGAAGCTGTGAAAAAGATGCTGAATGAACCGGAATATGTTTGCGCCCGAACCGGCCTGCTGTAAGTACTTCCCCTCTGTTTGTTATAGAATTTATGATCTCCTTATACACATATTGCTAACTCCAGTCCTTTCTGACACAGGGAGATGATCCGTTTTGGAGCAAGAAGTTTCAAGAGCCGACCGAAAGGGTAACCCGACCCAAAACGAGGGTGAAGAAACCCGCTCAGAAAAAACGGAACACCGCTTCCTCCACCCCGGATTTAGAGGAGGATGATGTGGGTAATCCGGACCTTGGGGTAGACCTCGATTCAGTTGGCTTACTTTTTATGCGTCTTATTGATGATGATATTTGTTAGGACGACGCTGAAGCGAGCAATGCTGAAGGCCCAGAGGTAACTATTCTTTCTTCTGACGCCGTTCCTTTGCCTTTGAAAAAACCTCGCCAAGCAAACCGGAAAATTAAATTTTCTCATCCTCTTGCTTACTTGGATCCCAAATTTCTTGTGAAGACCCAGCAACATGAAGCTCGACGCACCACCCGGCATAGCGGCCAGGTAGTTACCTCCGCCGGTTTACCGAACAGCCCGGTTCGGAAACGCCGCTCAGAGGTCTCCGATTTATGCGCTAGCTTTCCTCCTAAAGCAGGTTTAACTCGTCAGCCTCTAAATCCATCTGACTCCGATTATCAGGTCACTTCCCATTCATCTTCCGGTGAATCTTCAGCCACTCAACTGCCACCGTTGAAGACAGTTATTGTGTAAGTTGGACCGATCACAACATATCTCTGTATGGGTCTCTTAATCCATCTTATACCGTTTTTTCAATCTTTGGGTTTTTTCCAGGGCTAAACCTAGACCGAGCAAGAAGGCTCGTCTGGATAACCCGGCTGAAGGAACTGCCGCTCCTGAACCGGAGAAAACTCCTGGTGCTGACGCTCTGGGTCGTGAAGATACTCAGAATTATCTATCTCCGCAAGATGACTCCTGTGCTGAAGAAAGGCCTACCAAATCTGCCAGCCGTGCTAATCCGCCTGCCAGTCCTGTTCGGGTTGATGAATCCATCCCCCCTTCTGCAACTGTTGATAAACCGACAGGGCCACTGCAGAGCAGCGACTCCAAGGATGACGATGTTGTGATTACCGGCATTGGCCGCTCTGAACCGGGGAACACTGCTACTTTGGCCAAGCATACTGCCAAGGACGAAATCTCGGCCATAAAGGGCAAATGGGACCTTGACTCCTCTACGTACGCCTCTTTAAGCGCCCCGGATCTTTATTCTGGGTACCTGAACCGACTGTACACCAGTCGGGATTATGAAGCTGGCATGATCAGGATGATGAAGGAGAAACTTGAGGTAAATCTCTTGTGTTTCATTCCATTGCAACTTAGCTTATCAATATTTCCAAACCGAGATGGTAATCCGGATCAGGCTGCCTTGACAATTAAGGTCCGGTTTAGAAAACCCAAGGCTTCAACATAGGTCGTAATCAGAAAACATCATCAGATAACCATTAGCCCCTAAGTGCCAAGTTTAAATACTTGTATAAACCCTGGGACTTTTGTAATGTAAAACATGCGTTAAGATCAAGTATGCATTAGCCCCCAAGTATCGAGCACATAACTTGCTTGTGTGGTTGATACTTCCAAACCGTGCACTATTTTCTGAACACTGCTGATGACCTGCTGCAGGGGGATTTGAAGACCAAAGAGAAACAAGTCTCGGACCTTCAGGAAGCTTTAAAAACCCAGCAGGCTGAGACTGAAAAAGCGAAGGAGGAATTGACCAAAGCCTTAACTGTTATGGAGCAGCTGAAAGAAAGCCGCAAAAAGGAGCAAGCTGATTAGGCTACCGAAAAAACTCTTTTGACGAAGAGGGCTGAAAATGCTGAAGCTGCTCTCAAACCGGTGGTTGATGAATTATCCGTCGTGAAGCGACAAATACACTCTATGACCTCTGCCGTCTTCGGTAAACTTCTTTCAATGCCCCTCTTCCAATTTGTACCTTGTCTGCTATATCTTATATCCGGTTTGTAATCGATCCTGAAACACTGCAGGCACACGTATTGGACACTTGGGTTCTGATGTGCGGATGAAGCTCAAAGCTGCATATACTCTGATTGAACAGTTGTACACCGGAGCACAAAGAGCTATCACCACTGCATCGCATAACAACCCTGCGCCGTCCCTGATTCAAGACACGTTAAAAAGGCTATCTATGCTTCCAGCCCGGATTGAAGAGTTGAAAAGATCAGCTGCTCGAACTGGAGCCATCACTGCCTTGATCCGGGCTAAAGCGTGGGTACCAGATTTCGATCCTGTCGAAGCGGCCCAAGGATACCCCAGCTTGAAAGAAGACGGTTCAGACTTTGGTGAAGATGATTTGAGGGCGATAAACCGGGCGGTGCGCCCCCTAGCTTGTCAATTGGCTGAAGAAGCAGATCTGACCAATTACCAAGCCCAGTATGATAACCAGAATAAGCGAGTAGCTGCCCCGGTCCCTGAAGCAGAAAATCTTATTCCTCCAATCCGTAAGCATACCTATGCCCCTGACATTGAACCGTCTTTGCTGATTCACGAAGAAGCTGTCTTTCAAGCGCTAATGGGAATCGACTGGACCACTGCTGATTTCCAGCCAATGGGTAGCCAAGAGGATGCGGCGCAAGATGATCCACAGCCTTCGAACCGAGGCGGCGAACAGGCGTAATCAACGCTCCGGTTCACCTTAATTGGTGACGTTTAATCCGTCAACGAACAATATACTCAGGCACTATGTTGCCTTGTAATAAGATAGTTTAATACCTCTGCATTTGAATGTGCCTTCGCGCACGGGTACTGATGCTCTTTCCAATGACGTTTTCCAACTTTGTTTCCTGTCTTTACTGAGTACACAAGATCTTTCGACCCTATGAACCGTGTTGCGCCTTTGCCAAACCGGGCAACTTGCCTCCGGGTTAAAAAATGGACCAGGCTATGTCAGCTGAATCGGTTTAAAAATACTTGACGTTCCGAAGAACAAAAAACACCACAATAAAGGCAGAAAACAAAAACCAAATATGAAAAGGCAACGCGATAGCAAAACGCATGCAAATAGTGGTTGCTATGAACCGTTCAGAAGGCATGACCATCGTCAAGACAAAATAAACCAAGCCCCCAAGAGGTGTAGCTACGGTTTAGGTCGACTAGGTTCCTAACTGAATCGTGGCAAATATGCCGGTCAAATGGCATTGCATTGGTTCGGATTCGACCAAGCCCCCAAGTGACTCAGTGGCCAAGGGCCGATCAAGTGGCATTGCGTTGGTTCGGACACGACCAAGCCCCCAAATGATTCATTGGCTAAAAAGCCGACCAGAAGGCATTACCATTATCAAGACATGACCGAGCTCCCATAAGGCGTAATCTACGGTTCAAGTCGATTAGGTCCCCAACTGATTCAGTGGCAAATGTGCCAGCCAGAAGGCATTACCGTCGTCAAGACATGACCAAGCCCCCCGGAGGTGTAGCTACGGTTCAGGCCGACTAGGTCCCCAACTGACTTTATAGGGATAGCTATGTTCTCTTTGGTTCGAATACGACCTATGTTTGAACAGGAAGCCCCGTGTTTGAATAGCTATGTTCTCTTTGGTTCGAATACGACCTATGTTTGAACAGGAAGCCCCATGTTTGAATAGCTATGTTCTCTTTGGTTCGAATACGACCTATGTTTGAATAGGAAGCCCCATGTTTGAATAGCTATGTTCTCTTTGGTTCGAATACGACCTATGTTTGAACAGGAAGCCTCATGCTTGAACCGGGATATTGAACCGGATATTAAGAGCTTCACAGCCTTGTAGAAGACATCTTCCCTTGAACCGGCTTTTAAACCGGAATCTATAATTTGAACTTCTGTGAAGCGACCTCCAGGGGTAAACACACATGATGTGAAGAACAGCAGAGACCCCGCTTTACTCGAGGCTCCGGTTTATTGTATTAATCATAATATATACATGGTCATGATATGTACATAAACAGAGCCCAAGGCTCATGTATAATAAGGTCGAAGATGAGCTATGTTCCACGGCCTGCTGGTCTCCTCCTCTGATGTGCATGAATCCTTGCGCTCTCGAATATCAATCAGGTAATATGACCCATTATTCAGTTTCTTGCTGACCACAAAAGGTCCCTCCCAAGGCGGGGATAATTTATGCTGATCAGACTGATCCTGGATGAGCCGAAGCACCAAATCCCCCTCCTGAAAGGTTCTGGTCCTAACCCGACGGCTGTGGTAACGGCGAAGATCTTGCTGGTAAATCGCTGAACGGGCAGCTGCTAAGTCACGTTCCTCATCCAACAAGTCAAGAGCGTTCTGCCGTGCCTGCTCGTTGTCCGCTTCAACATACGCTGCCACACGCGGTGAATCATGTCGTATATCACTTGGGAGAACCGCCTCTGCTCCGTAGACCATAAAAAACGGCGTAAACCCCGTGGATCTGTTAGGCGTGGTGTTGATGCTCCATAATACAGACGGTAGCTCTTCCACCCAACAACCCGGCGTCCTTTGCAAAGGGACCAGAAGCCGGGGTTTGATACCTCTCAAGATCTCTTGATTCGCCCGTTCTGCCTGACCATTAGATTGTGGGTGCGCCACCGAAGAAACATCAAGCCGTATATGCTCCCGCGCACAGAACTCTCTCATGGTCCCTTTGGATAGATTGGTACCATTATCTGTGATGATGCTGTGTGGAAAACCAAACCGAAAGATCACCTTTTTTATAAACCGAACTGCCGTGGCAGCATCACATTTACTGACAGGTTCTGCCTCCACCCACTTTGTAAATTTGTCAACCGCCACCAGTAGGTGGGTCTTCTTATCTTTGGAACGCTTAAAAGGCCCAACCATATCGAGCCCCCAAGTTGCAAACGGCCAAGTGATTGGAATCATCCTCAATTCTTGAGCCGGTACGTGCGCTCGTCTGGAAAATTTCTGGCAACCATCACATAACCTGACCAGGTCTTCTGCATCAGCATGAGCAGTTAACCAATAGAAACCATGGCGGAACGCTTTAGCCACCAATGATTTTGAACCGGCGTGGTGACCGCAATCCCCTTCATGTATTTCACGTAATATCTCGCACCCCTCCGCAGGGGACACGCACCGTTGCAGCGCTCCTGACACGCTGCGATGATGTAGCTCTCCCTTGAGAATGGTCATAGATTTGGACCGCCGTACTATCTGTCGAGCCAGGCTTTCGTCTTCTGGCAACTCACCCCGGCTCATGTATGCCAGATAAGGGAGCGTCCAATCCGGGGTGACGTGAAGAGCCGCCACTAATTGAGCTTCCGGATCAGGAACAGCCAACTCCAGCTCACCGGGTAGCTGTACCGATGGGTGATACAATACATCCAAGAAAACATTGGGTGGGACCGGTTTACGCTGAGAGCCCAAGCGGCTTAAAGCGTCCGCTGCCTCATTCTTTCTGCGGTCCACATGGTCCACTTGATAGCCTTTGAAATGCCCAGCCACCATATCTACTTCACGTCGGTATGCAGCCATGAGCGGGTCCTTAGAATCCCAGGTGCCAGATACCTGCTGAGCCACCAGATCCGAATCACCAAAGCACCTAACTCTGCTCAGATTCATCTCTTTAGCCATCCGAAGACCATGAAGCAAAGCTTCATACTCAGCCGCATTATTTGTACAGGGGAACATTAAACGGAGGACATAACAAAACTTATCTCCTCGTGGGGAAGTTAAAACGACTCCAGCCCCCGAGCCTTCCAATTGCCTGGACTCGTCAAAATGAATAGTCCAATAGGTGTTATCTGGCTTCTCCTCAGGCGCTTGTAACTCTGTCCAATCGTTTATGAAGTCGACAAGTGCTTGTGACTTTATAGCCGTCCGGGGCACGTATCTTAAACCGTGCGGCCCAAGCTCGATAGCCCACTTAGCAATCCGACCGGTCGCCTCCCGGTTCTGTATGATGTCACCCAAAGGAGCAGAACTGACCACCGTGATGGGGTGTCCTTGAAAATATTGCCTCAGCTTCCGGCTTGCCATAAAAACACCATATACCAGCTTCTGCCAATGCGGATACCTTTGTTTGGACTCGATAAGTACTTCACTGATATAGTAAACCGGTCGCTGAACCGGATGTTCCTTTCCAGCCTCCTTTCGTTCTACCACCATCGCCACACTAACTGCTCTGGCATTCGCAGCCACATATAACAGGAGTGGCTCTTTATCTACAGGGGCTGCCAAAACTGGCGGATTGACTAGCTGCCGCTTCAAGTCCTCGAACGCTTTATCTGCCTCCGCACTCCAGACAAATTGATCCGTCTTCCTGAGCATTTGATACAAGGGGATCGCCTTTTCACCAAGGCGACTGATAAACCGGCTTAACGCTGCAATCCGTCCGGCCATGCGCTGAACATCATTGACGCATTTCGGTTTGGCCAGGGAGGTGATGGCTGTGATCTTTTCCGGATTAGCCTCAATTCCTCTATGGGATACTAAAAATCCCAACAACTTGCCTGCAGGCACACCAAAAACGCATTTAGCCGGGTTGAGCATCATTTGGTACACTCTCAAATTATCGAAAGTCTCCTTCAAATCGTCAACCAGGGTCTCCTTCTTTCGTGACTTGACCACAATATCATCCACATAAGCATGCACATTACGGCCAATCTGCTCGTGGAGGCAATTTTGCACACACCGTTGATAAGTAGCTTGCGCACTCTTAAGCCCGAAGGGCATAGACACATAGCAGAAGGCTCCAAAGGGAGTAATGAACGCCGTCTTCTCCTGGTCCTTAACTGCCATCTTGATCTGGTGATAACCTGAATAGGCATCCAAAAAACTCAAACGCTCGCAACCTGCCGTAGCATCAATGATCTGATCAATCCGGGGGAGAGCAAAAGGATCCGCTGGACAAGCTTTATTCAAGTCTGTGTAATCCACACACATGCGCCAGGTGCCGTTTTTCTTAAGAACCAGCACCGGATTAGCAAGCCATTCGGGATGAAAAACCTCGACGATAAAACCAGCTGCCAGGAGCCTGGCCACTTCCTCACCAATCGCTTTACGTCTTTCTTCGTTAAACCGCCGTAAGAACTGTTTTACCGGTTTATACTTAGGATCCACATTAAGGGTGTGCTCAGCGAGTTGCCTCGGTACACCAGGCATGTCAGACGGCTTCCATGCAAAGATGTCCCGATTCTCACGGATGAACTCGATGAGCGCGCTTTCCTATTTTGGATCCAAGTTTGCACTGATGCTGAACTGCTTGGATGAATCGCCAGGCACGAAGTCAACAAGCTTAGTTTCGGCCGCTGATTTGAACTTCAAGGCCGGATCGTGGTCCGTAGTTGGCTTTTTTAATGAAGTCATATCCTCCGGATCAACACTGTCTTTGTACTGCCTTAGCTCCTCCGTAGCACAAACCGATTCTGCATAAGTCGCATCTCCTTCCTCGCATTCCAAAGCGATTCGACGGCTGCCATGAACCGTTATAGTGCCCTTGTAACCCGGCATCTTAAGCTACAGATACACGTAGCAGGGCCGTGCCATAAATTTTGCATAGGCTGGCCGTCCGAACAAAACGTGGTACGGACTTTGGATTTTAACCACTTCGAACGTCAGCGTCTCTGACCTGGAATCGTGACTATCGCCAAAGACCACTTCAAGGGCTATCTTACCAACCGGATATGCAGACTTGCCAGGCACCACCCCGTGGAACACGGTATGGGTCGGTTTGAGATCCTTATCCACCAGTCCCATGCGACGGAAGGTCTCATAATACAAGATGTTAATGCTGCTCCCTCCGTCCATGAGCACCTTGGTGAGCTTATAACCTCCCACCTGAGGCGCTACCACCAATGCTAACTGACCCGGATTATAAACCCTGGGAGGATGATCTTCTCGGCTCCATATGATAGGCTGTTCCGACCAACGCAGATAGTGAGGCGTGGCCGGTTCGACAGCACTGACTGCCCGCCTCTGAAGCTTTCTATCCCGTTTATCCAAGCTTGTAGTAAAGACATGATACTGTCCACCACTCAGCTGTTTCGGGTTGTTCTGATAACCTGATTGCTGCTGTTCATAACCACTTTGATTACTTTGACTATTTCGGTTGTCCTGTCCGCCCGGATTACCCTGGAACCCTGGACCGGAATTTCCGCCACCGTATCCTGACCCGGAACCTGAACCATCGCCGGAGCTGTGATCATATTGGAAAGTGTTTGAGCTTTTGAATTCCTTCATGATATAACAATCCTTCCAAAGATGGGTTGCTGGTTCCTCTTTTGTTCCGTGCTTCGGACAGGGCTGGTTTAAGAAAAAGTTCAAACGCTCCGGATTAGGACCGGGATTTGCGCGGAAACTTGGTTGTTTCCCCTTACGCCGCTGGCCTTTATTCTGTGCACTTGTGCTGGCCACCAAGTCCATGCTCCCGTCCGGTTTACGCTTACCTCCGCCTCCATTACCTATCGGCCGATGTTGCTGGCCTTTGGTGTTGCTATTCTTCTTTCCCTTCCCTGCTTTGTCATCACCAGAGTCAGGATCCTTGGTACAGTCAGAATCCGCGTACTTCACTAAAGCGGTCATGAGCGTTCCTATATCTGTACAATCACGCTTCATCCGGCCCAGCTTTAGCTTCAAAGGGCCAAACCGACAGTTGCTTTCCAACAGCACCACGGCGGTGTCTGCGTTGATGCGATCTGACGAGTGCAAAACTTCTGATACCCGCCGTACCCAATGGGTAGTTGATTCCCCTTCTTGCTGAACGCAGGCTGCTAGGTCAACTATCGACATTGGCTGTTTGCACGTGTCCTTGAAATTCTTGATAAACCGGGCGCGCAATTGGGCCCACGAACTAATTGAATTAGGCGGTAAGCTCTTTAGCCAAGTCCGGGCCGTTCCTTCTAACATCATAGTAAAATACTTGGCACACGCCGTGTCATCCACATCCAGCATCTCCATAGCCATTTCATAGCTCTCCACCCATGTCTCCGGAGGCTGATCTGCCGTGTAGTTTGGTACCTTGCGGGGGCCTTTGAAGTCTTTGGGCAGGCGTACGTTGCGCAAAGCGGGAACAAGGCACGGTACTCCCAAAGAACTGGGGACCACCCCCGGTTCAACCGTAGCCGCTGGGAAGGTAGGTCTAAGCTGTCGAGCCTGATGCTGCGCTGCTAATCCGGCCTCCCGCGCACGGGCACGATCCATATTCTCCTGAGCATTGCCGACGCCCGCCGGGTTGTTGCCGCGGAGCGCTTCGCGCCAGGCATTGCTTGACACAGCCGGCTCCTCCATGCGCCTACTATGGCTCCCGCTGGGGCGAGGGGTGGAGTGAATCCGATCACGGCTTTGTGAATATGCTTCTTGTTGGGCCAACGCGGTCCTAAGGAGCTCCTTGACCCGGCGTGTCTCTACGTCCTGCGGTGAATCGCCTTCGATCGGAATGGCTTCCAGCCGAGCTGCCGCCGCGACGAGGTTATCCATCGGGTTGGAATAATGACCTGATGGTGTTGGCACGGCCTGTGGTGTACCAGTGTTGTGACGAGGCAGGTTTATTATCCGGTGCTGAACCGGGGCGCCAACTGTCGCCTCCGCACGGTTTACCCCCGGGGTGTTGAAGAGGTTTCGGGCGTCGAGGGCTGCGGGCAATCGAGATCGGGATTTCCTCTTTAGGACTTCCTGAGACGCATTCTGGTCCATCATGAGCCTGTAAGCCTGTGCATCCAAAGCGGCGCGCTCCGCCGTCATCCTGATCTCTTCTGCTGCCAGTTCCGCTTTAGCTTGAGTGATCTGCTCTCTCACTTTGGCAATCTCAGCATTGTGAACCTCCTGATCCACCGGGTTGGCTTCCGCCATGAGCGCTGCTAGTGCGTCAAACAGGTCTGACAAAACTTGGGCTGGTGAACCCGTAGGGCCTCCTGCCCGCGCAGCCGTTGCCGCTGCCGATCCGGAGATCGCTGCTGCTGCAGTTGATGATCGTGGCACCGCCTGCGTTCCAGCCATGAAGATCCCCACCCGAAAGGGCAGATCAGAGGGGTCCGGAATACTGTTGCCATCGGAGCCGCCCCCAATCCGATCATCTTGCAGCTGGTAAAGAGATTCGGTTTCCCCGGTCGACGTTTCATCGCCGGAGTAGATGGCGGTTTCGTCACCGGATTCGGATCCTTCCTCGTAGCTTCCTCCGTGGATGACCCCCACGAAGGCGTGCTTCCTGGTCGGTTTAGCCAGGGTCGATCTCGCGCACTGAGCTGTCTCGACGAGGTCGGCGCAGGCGCCTAACTCGGGGCCTGGCTCTCCGATCTTGCCGATGAAAACATGGATGCCACCAAAGGGGACCCGGTACCCGTACTCGATCGAGCCGGCGTCGGGGCCCCAGCCTGTGCTATCGATGTAGAGTTTTCCGCGGCGGCTCTTGGTCATCCGACCCACAGCGTAACCCTCGAGTCCTTCAAAGCGGCCCTCCAAAAACTTGAAACCATCTCGCGATAGCCCCACGGTGGGCGCCAAC

TGTCGTGGATTTGTCACGGCAGATGTCCTAGTGGGAGGACTTAGTCGTGGAGCCATCGCAACGGGTTAGCTTGAAGGGGTTAAAAGTGGACACAAGGACACGGTGTTTATACTAGTTCGGCCCCTTCGATGAAGGTAAAAGCCTACGTCTAGTTGTGATGGGATTGATGGGTTTCGAGGGCTTAGGGAGCGATCAGCTTCGCCTAAACCTCGAGTTCTTCTTCTCTGTCTCCAAACCGCCGTCGGGTCGTCCCCTTATATACACGGGTGACGCCCGTCGGTCTGCAAAGTCCCAACCTCCGGCTTACAAACGTGCCCGGGTCGGTCTCTCTATCCCTAACTTACAATGCAAGTTTACCTCAGGCCGGTTTACACCGTAAACCTCTAAACCGATTATAGGCCTTGGGCCCTTCTATTTCTCCTTGGGCTTTAACATCTCGGGTCCATTGACGGAGGTAACCCGGGCCCTGCAGGCCGGTTTACCCCAATTAGCCATATCCCCAACA

GGTTGTCAATCCCTTCACGGTCACTTACGAGAGTGAGATCTGATAGAGATGATAGATAATATTTTTGGTATTTTTGGTATAAAGATGCAAAGTAAAATAAAAGGGCAAAGTAAAGAAGCAAAGC

>Aegilops umbellulata 1-073;

TGTGTGTGTTGGCTTGCAAGGTGACTTCTTCGACGGGAAAGAACCAAACCGTGAAGCTACCGCGGATGAGTTGAAGATGCTGCAATACAACGCTCAAGCTTGTGATATTCTCTTCAACGGATTG

TGTCGTGGATTTGTCACGGCAGATGCCCTAGTAAGAGGACTTAGTCGTGGAGCCATCGCAACGGGTTAGCTTGAAGGGGTTAAAGTGGACACAAGGACACGGTTTTTATACTAGTTCGGCCCCTTCGATGAAGGTAAAAGCCTACGTCTAGTTGTGATGGGATTGATGGGTTTCGAGGGCTTAAGGAGCAATATGCTTCGCCTAAACCTCGAACTCTTCTTCTTCTCCCAAACCGCCGTCGGGTCGTCCCCTTATATACACGGGTGACGCCCGTCGGTCTGCAAAATCTCGACCTCCGGCTTATAAACGTGCCCGGGTCGGTCTCTCTATTCCTATCTTACAATACAAGTTGGCCCCAGGCCGGTTTACACCATAAACCTCTAAACCGGTTATAGACCTTGGGCCTTTCTACTTCTCCGTGGGCTTTAACATCTCGGATCTGTTGACGGCGGTAACCCGGGCCCTGCAGGCCGGTTTACCCCAGATAATAGCATCCCCAACA

TTAGGCCCCAGATTGATTTGAACAGGTTCATGTCAATCCTTAATAAGCCTCTGTCTTCAAATGTCTTCCAGTGACTTGTTTAAACCGTTGTTTCTCCGTCCCCTCTTGCTGTGGTAAACCGATATGACGTCAGCGCAAGCTACCGTCTCCTTTCGCTAACCCGCAAGAATTAGGGCGACGGCTGGGCTTCCGAAATCTCCGGCCCCTGGATTCACGCGCCTGACACGCGCGCCGCACCTTATAAATAAGACTTGAAGGCCATCTTCCTTTCCTCCCCTTCTTCGTCTTCCTTGCGACCCAGGCCTCAGAGCTCCGCCGCCGCCGTCAGCTGCTGCACCACCTCCAGCCGCTGCATCAACCTGATCGGATCAGAGCACCGCGGCGATTTTCCGCATCTGTTTGGACCCCGGTAAGGCCCCTGTTCTTATTGCTTTAGATCTGTTCTAGGGTTCATCGTCCTGCTACTGTTTCTTGCTCATCCCCATAGCAGCCCTTGCGAACAGATAATGCTACCTGTATCTGGATTGTAGTTCCTCTTAGCAGTCGCCATAAATCTGTACTCATTTCCTTAGACCTCACCCTTGTTCCTGCTTGTCGAACTTTATGTTATTCTGCTATGAGCCCGACTTCATATTACCTGCTTTTATTTCTTTTCTGCCCTAGCTGTAGATCCATTGTTTTGAAAGAATCCGTACGGGCTCTGTTTCTGCTTTCATACTCACAACTGTTTAGATCTGGTCGGTTTAACTCTTAGCAAAGCGAACAAACCGATCCACACATCCATTAGTCCCCTGGATGAACCGCCGGTTGATATTATTGTAGATAACCTGACTCCATATAAGAGCATCTTCGGTTTACTATCATACTTGCCCTCAAACCGGAATACCTTTGCCTCGTAGATTTCCTCATGGCAAAACAGGTGTATGAATGCAACTGGGTTCCCTCTTGCATCACTAAGACCCAACTAAACAACTTAGTTCTGACTGGTGCTTTGAGTAGCAAAAGTACCATCCATTGGAGGGTTCCAGGAGATGAATGCCCTCCTACCCCGCAAGAGGGAGAAGTGGTGGTGTTTGCGGATCACCTGGCCCGGGGGTTTAATCCACCCGGTTCAAAATTTTATCGGGATGTCTTGGCTAATTTTAGGCTGCGCCCACAAGATGTTGGTCCAAATTCAGTCACGAACATCTGCCATTTTCAAGTACTTTGCGAGGCATACTTTCAAGAGGAACCCACTGTAGAGCTGTTCCGGGATTTTTTCCATCTGAACCGACGTACTGAATTCACCGATGGCCCTAATATGGAACTTGGTGGTATGGCGATTCAGAAGAGGAAAGAGGTGACTTATCCTCACGCCAAGTTGCATAGTCATCCGCAAGAATGGAATTGCACTTGGTTCTATTGCAAAGATACTTCCCCTGCTGAAGAAAATCCACTGCCCGGTTTCCGTCCAGAGAGGCTTAGCAATACTCACCCCTTCCCCCCACGGCTAACTGCCAAAGAGAGGAGTAAGTACGCCCCTCAGCTGTCCAAGCTCAGAGCCTTCATGGCTAACGGTTTAACAGGGGTAGATCTTGCTCGTTGCTGGATTTCATGGAGCATCCTGCCCCTTAGCATCCGCTCCGGTTTAATGTGCCAATACACGGGTCGGGTTGATGATCCTTTGAGGCACTGCAACATTCAGCTGACGGAGGATGAAGTCGCTGAAGCTGTGAAGAAGATGCTGAACGAACCGGAACATGTCTGCGCTCGGACCGGCCTGCTTCCATTTTGTGCCACAAACAAACCACCTGCCGTAAGTAACTCCTCTGTTTATAGAATCTTTGATCTCCTTATATATATGTTGTTAACTCCAGTCCTTCCTGAAACAGGGAGATGATCCATTTTGGAGCAAGAAGTTACAAGAGCCGACCGAAAAGGTAACCCGACCCAAGACGAGGGTTAAGAAGCCTGCTCAGAGGAAAAGGAACACCGCTTCCAATACTCCGGATTTAGAGGAGGATGATGTGGGTAATCCGGACCTCGGGGTAGACCTCGATTCAGTTGGTTTGCTTTTTATACGTCTTATTGATGATGATATTTTTCAGGACGACGCTGAAGTGAGCCATGCCGAAGGCCCAGAGGTAACCATTCTTTCTTCTGATGCCGTTCCTTTGCCTTTGAAAAAACCTCGCCAAGCAAACCGGAGAATTAGATTTTCTCACCCTCTTGCTTACTTGGATCCCAAATTTCTTGTGAAGACCCAGCAACATGAAGCTCGACGCACCACCCGACATAGCGGCCAGGTAGTCACCTCCGCCGGTTTACCGAACAGCCCGGTTCGGAAACGCCGTTCAGAGGTCTCCGATTTATCAGGTCACTTCCCATTCCTCTTCCGGTGAATCTTCAGCCACTCAACTGCCACCGTTGAAGACAGTTATTGGGTGAGTTGGACCGATCACAACATATCTCTGTATGGGTCTCTTAATCCATCTTATACCGTTTTTTCAATCTTTGGGCTTTTTTCAGGGCTAAGCCTAGACCGAGCAAGAAGGCTCGTCTGGATAACCCGGCTGAAGGAACTGCCGCTCCTGAACCGGAGAAAACTCCTGGTGCTGACGCTCTGGGTTGTGAAGATACTCAGAACTATCTATCTCCGCAAGATGACATCTGCGCTGAAGAAAGGACTACCGAACCTGCTAGCCGTACTGATCCGCCTGCCAGTCCTGTTCGGGTTGAGGAATCCATCCCCCCTTCCGCAACTGCTGATAAACCGACAGGGCCACTGCAGAGCAGCGACTCCAAGGATGATGATGTCGTGATTACTGGTATTGGCCGCTCTGAACCGGGGAACACTGCTACTTTGGCCAAGCATACTGCCAAGGACGAAATCTCGGCCATAAAGGGCAAATGGGACCTTGACTTGTCTACGTACGCCTCTTTAAGCGCCCCGGATCTTTATTCTGGGTATCTGAACCGGCTGTACACCAGTCGGGATTATGAGGCTGCCATGATCAAGATGATGAAGGAGAAACTTGAGGTAACTCCCTTGTGCTTCATTCTATTGCAACTTAGCTTATCCATATTTCCAAACCAAGATGATAAACCGGATCAGGCTGCCTTGACAATTAAAGTCCGGTTTAGAAAACCCCAAGGCTTCCAACATAGGTCATAACCAGAAAACATCATTAGATAACTATTAGCCCCCAAGTGCCAAGCTTAAATACTTGTATTAATCTTGGGACTTATTGTAATGGATAATTTGTGTTTAAAATCAAATATGCATTAGCCCCCAAGTATCGAGCACATAACTTGCTTGTGTGGTTGATACTTCCAAACCGTGCAATATTTTCTGAACACTTGATAACCTGCTGCAGGGGGATTTGAAGACCAAAGAGAACCAAGTCTCGGACCTTCAGGAAGCTTTAAAAGCCCAGCAGATTGAAACTGAAAAAGCGAAGGAGGAATTGACCAAAACCTTAACTGTTATGGAGCAGCTGAAAGCAAGCCGCAAAAAGGAGCAAGCCGATTGGGCTACTGAAAGAGCTCTTTTGACGAAGAGGGCTGAAAATGCCGAAGCTGCTCTTAAACCGGTGGTTGATGAATTATCCACCGTGAAGCGACAGATACACTCCATGACCTCTGCCGTCTTTGGTAAACTTTTCGATGCCCTTCTTCCAATTTGTACCTTGTTTGCTACACCCTATGTCCGGTTTGTAATCCACCTTGAAACACTGCAGGCTCACGTATTGGTCACTTGGGTTCTGACGTGCGGATGAAACTCAAAGCTGCTTATACTCTGATTGAACAGTTATACACCGGAGCACAAAGAGCTATCACCACTGCATCGCATAACAACCCTGCGCCGTCTCTGATTCAAGACACATTAAAGAGGCTGTCCATGCTTCCGGCCCGGATTGAAGAGTTGAAGAGATCAGCTGCTCGAACTGGAGCCATTACTGCCTTGATCCGGGCCAAAGCGTGGGTACCAGATTTCGATCCTGTCGAAGCGGCCCAAGGATACCCCAGCTTGAAGGAAGACGGCTCAGACTTTGGTGAAGACGACTTGAGGGCGATAAACCGGGCGGTGCGCCCCTAGCTTGTCAATTGGCTGAGGAAGCAGATCTGTCACGTTACCAAGCCAATACGATAACCAGAACAAACGAGTATCTGCCCCGGTTCCTGAAGCGGGAAACCTTGTTCCTCCAATCCGTAAGCATACTTATGCCCCAGACATTGAACCGTCTTCGCTGATTCATGAAGAAGCCGTCTTTCAAGCGCTAATGGGAATCGACTGGACCACTGCTGATTTCCAGCCAATGGGTAACCAAGATGGAGCTGAAGCAGCGCGAGACGATCCCCAGCCTTCGAACCAAGGCGGCGAACAGGCGTAATCAACGCTCCGGTTCACCCTACTTGGTGACGTCTTAATCCGTCAACGAACAATATAGTCAGGCACTATGTTTGCCTTGTAATAAGATAGCTTGATACCTATGCATTTGAATGTGCCCTCGCGCACGAGTACTGATGCTCTTTTCTAATGTCGTTTTCCAACATTGTTTCCTTGCTTTATTGAGTACACAAGATCTTCCGGCCCTATGAACCATGCTGCGCCCTTGCCAAACCGGGTAACTTGCCTCTGTGTTAAAAAACGGACCAGGCTATGCCAGCTGAATCGGTTTAAGGATACTTGACGTTCCGAAGAACCGAAAAAACACCACAATAAAGGCAGAAAACAAAACCAAATATGAAAAGGCAACGCGATAGTAAAACGCATGCGAATAGTGGTTACTATGAACCGTTCAGAAGGCGTGTCCATCGTCAAGACAGAACCGAGCCCCCAAGAGGTGTAGCTACGGTTTAAGTCGACTAGGTTCCTAACTGAATCTTGGCAAATATGCCGGCCAAATGGCATTGCATTGGTTCGTCAACCAAGCCCCCAAGTGACTCAATGGCTAAAAAGCCGACCAGAAGGCATTACCATCATCAAGACATGACCGAGTTCCCATAAGGCGTAATCTACGGTTCAAGTCGATTAGGTCCCCAACTGATTCAGTGGAAAATATGCCAGCCAGAAGGCATTACCGTCGTCAAGACCTGACCAAGCCCCCCGGAGGTGTAGCTATAGTTCAGGCCGACTAGGTCCCCAACTGACTTTATAGGGATAGCTATGTTCTCTTTGGTTCGAACACGACCTATGTTTGAACCGCTGTGTTCTCTTTGGTTCGAATACGACCTATGTTTGAACAGAAATCCCCATGTTTGAATAGCTATGTTCTCTTTGGTTCGAATACGACCTATGTTTGAACAGGAAGCCCCATGTTAAATAGCTATGTTCTCTTTGGCTCGAATACGGCCTATGTTTGAACAGTAAGCCTTATCTTTGAACCGGGATATTGAACCGGATATTAAGAGCTTCGCAGCCTTGTAGGAGACATCTTCCCTTGAACCGGCTCTTAAACCGGAGTCTATAATTTGAACTTCTGTGAAGCGACCTTCAGGGGTAAACACGCATGATGTGAAGAACAGCAGAGACCCCGCTTTACTCGAGGCTCCGGTTTATTGTATTAATCATAATATATACATGGTTATGATATGTACATAAGCAGAGCCCAGGGCTCATGTATAATACGGCCGAAGATGAGCTATGTTCCACGGCCTGCTGGTCTCCTCCTCTGATGTGCGTGAATCCTTGCGCTCTCGAATATCAATCAGGTAATATGACCCATTATTCAGATTCTTGCTGACCACAAAAGGTCCCTCCCAAGGCGGGGATAATTTATGCTGATCAGATTGATCCTGGATGAGCCGAAGCACCAAATCCCCCTCCTGAAAGGTTCTGGTCTTAACCCGACGGCTGTGGTAACGGCGAAGATCTTGCTGGTAAATCGCTGAACGGGCAGCTGCCAAGTCACGCTCCTCATCCAACAAGTCCAGAGCGTTCTGCCGTGCCTGCTCATTATCCGCTTCAACATATGCTGCCACGCGAGGTGAGTCATGTCGTATATCACTTGGGAGAACCGCCTCCGCTCCATAAACCATAAAAAACGGCGTAAACCCCGTGGATCTGTTAGGCGTGGTGTTGATGCTCCATAATACAGATGGTAGTTCCTCCACCCAACAACCCGGCGTCCTTTGCAAAGGGACCAGAAGCCGGGGTTTGATACCTCTCAAGATCTCTTGGTTCGCTCGTTCTGCCTGACCATTAGACTGCGGGTGCGCTACCGAAGACACATCAAGCCGTATATGCTCCCGTGCACAGAACTCCTTCATGGCCCCCTTGGATAGATTGGTACCATTATCTGTGATGATGCTGTGTGGAAAACCAAACCGGAAGATCACCTTCTTTATAAACCGAACCGCCGTGGCGGCATCACACTTACTGACCGGTTCTGCCTCTACCCACTTTGTAAATTTGTCAACCGCCACCAGTAGGTGGGTCTTCTTATTTTTGGAACGCTTAAAAGGCCCAACCATGTCAAGCCCCCAAGTCGCAAACGGCCAAGTGATTGGAATCATCCTCAATTCTTGAGCCGGTATGTGTGCTCGTCTGGAAAATTTCTGGCAACCATCACATAATCTGACCAGGTCTTCCGCATCAGCATGAGCAGTTAACCAGTAGAAACCGTGGCGGAACGCTTTAGCCACCAATGATTTTGAACCGGCGTGGTGGCCGCAATCCCCTTCATGTATTTCTCGTAGTATCTCGCACCCCTCCTCCGGGGATATGCACCTTTGCAGCGCTCCTGACACGCTGCGATGATGCAACTCTCCTTGGAAAATGGTCATAGACTTGGACCGTCGTACTATCTGTCGAGCCAAGCTTTCATCTTCTGGCAACTCACCCCGGTTCATATATGCCAGGTAAGGGAGCGTCCAATCCGGGGTGGCGTGAAGAGCCGCCACTAACTGAGCCTCCGGATCAGGAACAGCCAACTCCAGCTCACCGGGCAGCTGTACCGATGGGTGGTACAATACATCCAAGAACACATTGGGCGGGACCGGTTTACGCTGAGAGCCCAAGCGACTTAAAGCGTCCGCTGCCTCATTCTTTCTGCGGTCCACATGATCCACTTGATAACCTTTGAAATGTCCAGCCACCATATCCACTTCACGTCGGTACGCCGCCATAAGCGGATCCTTAGAATCCCAGGTGCCAGATACCTGCTGGGCCACCAGATCTGAATCACCAAAGCACCTAACTCTGCTCAAATTCATCTCCTTAGCCACCCGAAGACCATGAAGCAAAGCTTCATACTCCGCCGCATTATTTGTACAGGGGAACATCAAACGGAGGACGTAACAAAACTTATCTCCTCGTGGGGAAGTTAAAACGACTCCAGCCCCCGAGCCTTCCAATTGTCTTGACCCGTCGAAATGAACGGTCCAATAGGTGTTATCTGGTTTCTCCTCAGGCGCTTGTAACTCTGTCCAATCGTTTATGAAATCGACAAGTGCCTGTGATTTTATGGCCGTCCGGGGTACGTACCTTAAATCGTGCGGCCCGAGCTCGATAGCCCACTTGGCAATCCGACCGGTCGCCTCCCGGTTCTGTATAATGTCACCCAAAGGAGCTGAACTGACCACCGTGATTGGGTGTCCTTGGAAATATTGCCTCAGCTTCCGGCTTGCCATAAAAACGCCATATACCAGCTTCTGCCAATGCGGATACCTTTGTTTGGACTCGATAAGCACTTCACTGATATAGTAAACCGGTCGCTGAACCGGATGTTCCTTTCCAGCCTCCTTTCGTTCTACCACCATCGCCACACTGACTGCCCTGGCGTTCGCTGCAACATATAACAGGAGTGGCTCTTTATCTACAGGGGCTGCCAACACCGGCGGATTGACTAGTTGTTGCTTCAAGTCCTCAAACGCCTTATCAGCCTCCGGACTCCAGACAAACTGATCCGTTTTCTTGAGCATTTGATACAAGGGAATCGCCTTTTCACCAAGGCGACTGATAAACCGGCTTAACGCCGCAATCCGCCCGGCCATGCGCTGAACATCATTGATGCATTTCGGTTTGGCCAGGGAGGTGATGGCTGTGATCTTTTCTGGATTGGCCTCAATTCCTCTATGGGACACTAAAAACCCCAACAGCTTGCCTGCAGGTACACCAAAAACACATTTAGCCGGGTTGAGCATCATTTGGTACACTCTCAAGTTATCGAAAGTCTCCTTCAAATCGTCAACCAGGGTCTCCTTCTTTCGTGACTTGACCACGATATCATCCACATAAGCATGCACATTACGCCCAATCTGCTCGTGGAGGCAATTTTGCACACATCGTTGATAAGTAGCCTGCGCACTCTTAAGCCCAAAGGGCATAGACACATAGCAGAAGGCTCCAAAGGGAGTAATGAACGCTGTCTTCTCCTGGTCCTTAACTGCCATCTTGATCTGATGATAACCCGAATAAGCATCCAAAAAACTTAAACGCTCGCAACCTGCCGTAGCATCAATGATCTGATCAATCCGGGGGAGAGCAAAAGGATCTGCTGGGCAAGCTTTATTCAAGTCTGTGTAGTCCACACACATGCGCCAGGTGCCGTTTTTCTTAAGAACTAGCACCGGATTAGCGAGCCATTCGGGATGAAAAACCTCAATGATAAAACCAGCTGCCAGGAGCCTGGCCACTTCCTCTCCAATCGCTTTACGTCGTTCTTCGTTAAACCGCCGTAAGAACTGCTTTACCGGCTTATATTTAGGATCCACATTAAGGGTGTGCTCAGCGAGTTGCCTCGGTACACCAGGCATGTCAGATGGCTTCCATGCAAAGATGTCCCGATTCTCACGGATGAACTCGATGAGCGCGCTTTCCTATTTTGGATCCAAGTTTGCACTGATGCTGAACTGCTTGGATGAATCGCCAGGTACGAAGTCAACAAGCTTAGTTTCGGCCGCTGACTTGAACTTCAAGGCCGGATCGTGGTCCGTAGTTGGCTTTTTTAATGAAGTCATATCCTCCGGATCAACACTGTCTTTGTACTGCTTTAGCTCCTCCGTAGCACAAACCGATTCTGCATAAGTCGCATCTCCTTCCTCGCATTCCAAAGCGATTCGACGGCTCCCATGAACCGTTATAGTGCCCTTGTAACCTGGCATCTTAAGTTGCAGATACACGTAACAGGGCCGTGCCATAAATTTTGCATAAGCCGGCCGTCCGAACAAAGCGTGGTACGGACTTTGGATTTTAACCACTTCGAACGTCAGCGTCTCTGATCTGGAATCGTGACTATCGCCAAATACCACTTCAAGGGCTATCTTACCAACCGGATATGCAGACTTGCCAGGCACCACTCCGTGGAATACTGTATTGGTCGGTTTAAGATCCTTATCTACCAGTCCCATACGACGGAAGGTCTCGTAATACAAGTTGTTGATGCTGCTTCCTCCGTCCATGAGCACCTTGGTAATCTTATAACCTCCCACCTGAGGCGCCACCACCAATGCTAACTGACCCGGATTATAAACCCTGGGAGGGTGATCCTCTCGGCTCCATATGATGGGCTGTTCTGACCAGCGCAGATAGTGAGGCGTGGCTGGTTCGATAGCACTAACTGCTCGCCTCTGAAGCTTTCTATCCCGTTTATCCAGGCTTGTAGTAAAGACATGATACTGTCCACCACTCAACTGTTTCGGGTTGCTCTGATAACCTGTTTGCTGTTGCCCGAAACCACTCTGATTACTTTGATTATTTTGGCTGACCTGCCCGCCCGGATTGCCCTGGAACCCTGGACCGGAACCTGAACCGCCGCCGGAGCTGTGATCATTCTGGAAAGTGTTTGAGCTTTTGAACTCCTTCATGATATAACAATCCTTCCAAAGATGGGTTGCTAGTTCCTCCTTTGTTCCGTGCTTCGGACAGGGCTGGTTTAAGAAAAAGTTCAAACGCTCCGGATTAGGACCAGGATCTGTGCGGAAACTTGGCTGTTTCCCCTTGCGCCGCTGGCCCTTGTTCTGTGCACTTGCGTTGGCTACAAAGTCCATGTTCCCGTCCGGTTTACGCTTACCTCCGCCTCCATTACCTGTCGGTCGAGGCTGCTGGCCTTTGGTGTTGCTATTCTTCCTTCCCTTCCCTGCTTTGTCATCACCAGAGTCAGGATCCTTGGTACTGTCAGAATCCGCATACTTCACTAAAGCGGTCATGAGCGTTCCTATATCTGTGCAATCACGCTTCATCCGGCCCAGCTTTAGCTTCAAAGGGCCAAACCGACAGTTGCTTTCCAACAGTACTACAGCGGTGTCTGCGTTGATGCGATCTGATGAGTGCAAGACTTCTGATACTCGTCGTACCCAATGGGTAGTTGATTCCCCTTCTTGCTGAACGCAGGCTGCTAGGTCAACTATTGACATTGGCAGTCTGCACGTGTCCTTGAAGTTCTTGATAAACCGGGCGCGCAATTGGGCCCACGAGCTAATTGAATTAGGCGGCAAACTCTTTAGCCAAGTCCGGGCCGTTCCTTCTAACATCATAGTAAAATACTTGGCACACGCCGCGTCATCCACATCCAGCATCTCCATAGCCATTTCATAGCTCTCCACCCACGTCTCCGGAGGCTGATCTGCCGTGTAGTTTGGTACCTTGCGGGGGCCTTTGAAGTCTTTGGGCAGGCGTACGTTGCGCAAGGCGGGGACAAGGCACGGTACTCCCAAAGAACTGGAAACCACCCCCGGTTCAGCCGCAGCTGCTGGGAAGACAGGTGTAACCTGATGAGCCTGATGCTGCGCTGCCAATCCGGCCTCCCGTGCACGGGTGCGATCCACATTCTCATGAGCATTGTCAACGCCCGCCGGATTGTTGCCGCGGAGCGCTTCACGCCTGGCATTACTTGAAACGGCCGGCTCCTCCACGCGCCTGCTATAGCTCCCGCTGGGGCGAGGGGTGGAGTGAATCCGATCACGGCTTTGCGAATATGCTTCTTGCTGGGCCAACGCGGTCCTAAGGAGCTCCTTGACCCGGCGTGTTTCTTCATCCTGCGGTGAATCTCCTTCGATCGGAATGGCTTCCAACCGAGCCGCCGCCGCGACGAGGTTATCCATCGGATTGGAATAATGACCTGACGGCGTTGGCATGGCCGGTGGCGTATCAGTGTTATGACGAGGCAGGTTTATAATCCGATGCTGAACCGGGGCGCCGGCCGTCACCTCCGCACGGTTTACCCCCGGGGTGTTGAAGAGATTTCGAGCATCAAGGGCGGCGGGCAACCGAGACCGGGATTTTCTCTTCAGGACCTCCTGAGACGCCTTCTGGTCCATCATGAGCCTGTAAGCCTGTGCATCTAAAGCGGCGCGCTCCGCCGTCATCCTGATCTCTTCTGCTGCCAGCTCCGCTTTAGCCCGAGTGATCTGCTCTCTCACTTTGGCAATCTCAGCATTGTGAACCTCCTGATCCACCGGGTTGGCTTCCGCCATGAGCGCTGCTAGTGCGTCAAACAGGTCTGACAAAACTTGGGCTGGTGAACCTGTAGGGCCTCCTGCCCGCGCAGCCGTTGCTGCTGCTGATCCGGAGATCGCTGCTGCTGCAGTTGATGATCGTGGCGCCGCCTGTGTTCCAGCCATGAAGATCCCAACCCGACAGGGCAGATCCGAGGGGTCCGGAATACTGTTGCCATCGGAGCCGCCCTCAATCCGATCATCTTGTAGCTGGTAAAGAGATTCGGTTTCCTCGGTCGACGTTTCATCGCCGGAGTAGACGGCGGTTTCGTCAGCAGATTCAGATCCTTCCTCGTAGCTTCCTCCGCGGATGACCCCCACGAAGGCGTGCTTTCTGATCGGTTTAGCCAGGGTCGATCTCGTGCACTGAGCTGTCTCGATGAGGTCGGCGCAGACGCCTAACTCGGGGCCCGGTTCTCCGATCTTGCCGATGAAAACATGGATGCCGCCAAAGGGGACCCGGTACCCGTACTCAATCGAGCCGGCGTCGGGGCCCCAGCCTGTGCTATCGATGTAGAGTTTTCCGCGGCGGCTCTTGGTCATCCGTCCCACAGCGTAACCCTCGAGTCCTTCAAAGCGGCCCTCCAAGAACTTGGAACCATCTCGCGATAGCCCCACGGTGGGCGCCAAC

TGTCGTGGATTTGTCACGGCAGATGCCCTAGTAAGAGGACTTAGTCGTGGAGCCATCGCAACGGGTTAGCTTGAAGGGGTTAAAGTGGACACAAGGACACGGTTTTTATACTAGTTCGGCCCCTTCGATGAAGGTAAAAGCCTACGTCTAGTTGTGATGGGATTGATGGGTTTCGAGGGCTTAAGGAGCAATATGCTTCGCCTAAACCTCGAACTCTTCTTCTTCTCCCAAACCGCCGTCGGGTCGTCCCCTTATATACACGGGTGACGCCCGTCGGTCTGCAAAATCTCGACCTCCGGCTTATAAACGTGCCCGGGTCGGTCTCTCTATTCCTATCTTACAATACAAGTTGGCCCCAGGCCGGTTTACACCATAAACCTCTAAACCGGTTATAGACCTTGGGCCTTTCTACTTCTCCGTGGGCTTTAACATCTCGGATCTGTTGACGGCGGTAACCCGGGCCCTGCAGGCCGGTTTACCCCAGATAATAGCATCCCCAACA

GATTGTGCCCCGAAGAATTCAACAAAATCAGCCGTCTTGAGAATGCAAAGGAAATTTGGGATACTTTGATTGATATGCACGAAGGTACTGACTCCGTCAAGGAATCCAAATTGGATGTGCTTCA

>Aegilops umbellulata 1-075;

GATCGGTGTCAGGCAGTTGGGTGTCAGACAGGTGCTTAGAAACTTCAGACTGATGGTCTGTAGGTGACTGTGCCATATCTTCAGATGCAAGCAACTGAAGCTGATCAGAAAGCAGAGCAGGTAG

TGTCGTGGATTTGTCACGGCAGATGTCCTAGTAAGAGGACTTAGTCGTGGAGCCATCGCAACGGGTTAGCTTGAAGGGGTTAAAGTGGACACAAGGACACGGTTTTTATACTAGTTCGGCCCCTTCGATGAAGGTAAAAGCCTACGTCTAGTTGTGATGGGATTGATGGGTTTCGAGGGCTTAGGGAGCGATAAGCTTCGCCTAAACCTCGAGTTCTTTTTCTCTGTCTCCAAACCGCCGCCGGGTCATCCCCTTATATACACGGGTGACGCCCGTCGGTCTGCAAAGTCCCAACCTCCGGCTTATAAACGTGCCCGGGTCGGTCTCTCTATTCCTAACTTACAACACAAGTTTACATCAGGCCGGTTTATACCATAAACCTTTAAACCGATTATGGGCCTTAGGCCCTTCTATCTCTCCTTGGGCTTTAACATCTCGGGTCTGTTGACGGAGGTAACCCGGGCCCTGCAGGCCGGTTTACCCCGGATAGCAACATCCCCAACA

TTAGGCCCCAGATTGATTTGAACAGGTTCATGTCAATCCTTAATAAGAATTTCTGTCTTCAAATGTCTTCCAGTAACTTGTTAAACCGCTGTTTCTCCGTCTCCTCTTGCTATGGTAAACCGATATGACGTCAGCGCAAGTTGCCGTTTCCTTTCGCTAACCCGCAAGAATCAGGGCGACGGCTGGGCTTCCGAAATCTCCGGCCCCTGGATTCGCGCGCCTGACACGCGCGCCGCGCCTTATAAATAAGACTTGAAGGCCATCTTCCTTTCCTCCCCTTCTTCGTCTTCCTCGCGACCCAGACCTCAGAGCTCCGCCGCCGCCGTCAGCTGCTGCATCAACTTCGGCCGCTGCATCAACCTGATCGGATCAGAGCACCGCGGCGACTTTCCACATCTGTTTGGACCCCGGTAAGGCCCCTGTTCTTGTTGCTTTGGATCTGTTCTAGGGTTCGTCGTCCTGCTACTGTTCCTTGCTCATCCCCATAGCAGCCTTTGCGAACAGATGATGATACCTGTATCTGGATTTGTAGTTCCTCTCGGTATTCGCCATAAATCTGTATTCGTCTTCTTAGATCTCGCTCATTTCCTGCTTGTCGAACTTACGTTGTCCTGCTGTGAGCCAAACTTCATATTTTTATTTCTTTTTCGCCCTAGCTGTAGATCCATTGCTTTGAAAGAATCCGTACGGGATCTGTTTCTGCTTTCATACTCACAGCTGTTAGATCTGGTCGGTTTAACTCTTAGCAAAGTGAACAAAACGATCCACACATCCATTAGTCCCCTTGATGAACCGCTGGTTGATATCATTGTGGATAACCTGACTCTATATAAGATCATCTTCGGTTTATAATCACACTTGTTCTCAAACCGGAATACATTTATCTTGTAGATTTCCGCATGGCAAAACAGGTGTACGAATGCAACTGGGTTCCCTCTTGCATCACTGAGACCCAACTAAACAACTTAGTTCTGACTGGTGCTTTGAGTAGCAAAAATACCATCCATTGGAGGGTTCCAGGAGACGAATGTCCTCCTACCCCGTAGGAGGGAGAAGTGGTGGTGTTTGCGGATCACCTGGCCCGGGGGTTTAACCCACCCGGTTCAAAATTTTATCGGGACGTCTTGGCTAATTTTAGGCTGCGCCCACAAGATGTTGGTCCAAATTCAGTCACGAATATCTGCCATTTTCAAGTACTTTGCGAGGCGTACTTTCAAGAAGAACCTACTGTAGAGCTGTTCCGGGACTTTTTCCATTTGAACCGACGTACTGAATTTACCGACGGCCCTAATATGGAACTGGGTGGTATGGCGATTCAGAAGAGGAAGGAGGTGACTTATCCTCACGCCAAGTTGCATAGTCATCCGCAAGAATGGAATTGCACTTGGTTCTATTGCAAGGATACTTCCCCTGCTGAAGAAAATCCACTGCCCGGTTTCCGTCCAGAGAGACTTAGCAACACTCACCCCTTCCCCCCACGGCTAACTGCCAAAGAGAGGAGTAAGTATGCCCCTCAGCTGTCCAAGCTCAGAGCCTTCATGGCTAATGGTTTAACGGGGGTTGATCTTGCTCGTTGCTGGATTTCATGGAGCATCCTGCCCCTTAGCATCCGCTCCGGTTTGATGTGCCAATACACGGGTCGGGTTGATGATCCTTTGAGGCACTGCAACATTCAGCTGACGGAGGACGAAGTCGCTGAAGCTGTGAAGAAGATGCTGAACGAAACGGAACATGTTTGCGCTCGAACCGGCCTGCTTCCATTTTGTGCCACAAACAAACCACCTGCTGTAAGTAACTCCTCTGTTTATAGAATCTTTGATCTCCTTATATATATGTTGTTAACTTCCAGTCCTTTTCGAAACAGGGAGATGATCCGTTTTGGAGCAAGAAGCTTCAGGAGCCAACCGAAAAGATAATCAGACCCAAGACAAGGGTGAAGAAGCCTGCTACAAAGAAAAGGCACACTGCTTCCTGTACCCCCGATTTAGAGGAGGATGATGTGGGTAATCCGGACCTTGGGGTAGACCTTGATTCAGTTGGCTTACTTTTTGTACGTCTTATTGACGATGATATTTGTCAGGACGACGCTGAAGCGAGCAATGCTGAAGGCCTAGAGGTAACTATTCTTTCTTCTGATGCCGTTCCTTTGCCTTTGAAAAAACCTCGCCAAGCAAACCGGAAAATTAAATTTTCTCACCCTCTTGCTTATTTGGATCCCAAATTTCTAGTGAAGACCCAGCAACATGAAGCTCGACGCACCACTCGGCATAGCGGCCAGGTAGTCACCTCCGCCGGTTTACCGAACAGCCCGGTTCGGAAACGCCGCTCAGAGGTCTCCAATTTATGCGCTAGCTTTCCTCCTAAAGCAGGTTTAACTCGTCAGCCTCTAAATCCATCTGACTCCGATTATCAGGTCACTTCCCATTCCTCTTCCGGTGAATCTTCAGCCACTCAACTGCCACCGTTGAAGACAGTTATTGGGTAAGTTGGACCGATCACAACATATCTCTGTATGGGTTTCTTAATCCATCTTATACCGTTTTTTCAATCTTTGGGCTTTTTCCAGGGCCAAACCTAGACCGAGCAAGAATGCTCGTCTGGATAACCCGGCTGGAGGAACTGCCGCTCCTGAACCGGAGAAAACTCCTGGTGCTGACGCTCTGGGTCGTGAAGATACTCAGAGTTATCTATCTCCGCAAGATGACACCTGCGCTGAAGAAAGGATTATCGAACCTGCCAGCCATACTGATCCGCCTGCCAGTCCTGTTCGGGTTGAGGAATCCATCCCCCCTTCTGGCACTGCTGATAAACCGACAGGGCCACTGCAGAGCAGCGATTCCAAGGATGATGATGTGGTGATTACCGGCATCGGTCACTCTGAACCGGGGAACACTGCTACCTTAGCCAAGCATACTGCCAAGGATGAAATCTCGGCCATGAAGGGCAAATGGGACCTTGACTCTTCTACGTACGCCGCTTTAAGCGCCCCGGATCTTTATTCTGGGTACCTGAACCGACTGTACACCAGTCGGGATTATGAAGCTGGCATGATTAAGATGATGAAGGAGAAACTTGAGGTAACTCCCTTGTGCTTCATTCCATTGCAACTTAGCTTATCAATATTTCCAAACCGAGATGGTAAACCGGATCAGGCTGTCTTGACAATTAAAGTCCGGTTTAGAAAACACCAGGCTTCAACATAGGTCATAACCAGAAAACATCATTAGATAACTATTAGCCCCCAAGTGCCAAGTTTAAATACTTGTATTAATCTTGGGACTTTTTGCAATGGGAAACTTGCGTTAAAATCAAATATGCATTAGCCCCCAAGTATCGAGCACATAACTTGCTTGTGTGGTTGATACTTCCAAACCGTGCCATATTTTCTGAACACTGTTTTGACGACCTGCTGCAGGGGGATCTAAAGACCAAAGCGAAACAAGTCTCGGACCTTCAGGAAATCTTAAAAACCCAACAGGCTGAAACTGAAAAAACGAAGGACGAATTGGCCCATGCCTTAACCGTTATGGAACAGCTGAAAGAAAGCCACCAGAAAGAACAAGCTGATTGGGTTACTGAGAGAGCTCTTTTGACGAAGAGGGCTGAAAATGCCGAAGCTGCTCTTAAACCGGTGGTCGATGAATTATCCACCGTGAAGCGACAGATACACTCCATGACCTCTGCCGTCTTTGGTAAGCTTTTCGACACCCTTCTTCCAATTTGTACCTTATCTGTTACATCCTATGTCCGGTTTGTAATCCATCTTAAAACACTGCAGGCTCATGTATTGGACACTTGGGTTCTGATGTGCGGATGAAACTCAAAGCTGCTTATACTCTGATTGAACAGTTGTACACCGGAGCACAAAGGGCTATTACCACTGCGTCGCATAACAACCCTGCGCCGTCTCTGATTCAAGACACATTAAAGAGGCTGTCCATGCTTCCAGCCCGGATTGAAGAATTGAAAAGATCAGCTGCTCGAACTGGAGCCATTACTGCCTTGATCCGGGCCAAAGCATGGGTACCAGATTTCGATCCTGTCGAAGCGGCCCAAGGATACCCCAGCTTGAAGGAAGACGGTTCAGACTTTGGTGAAGACGATTTGAGGGCGATAAACCGGGCGGTGCGCCCCCTAGCTTGTCAATTGGCTGAGGAAGCAGATCTGTCACGTTACCAAGCCCAATACGACAACCAGAACAAACGAGTGGCTGCTCCGGTCCCTGAAGCGGGAAACCTTGTTCCTCCAATCCGTAAGCATACTTACGCCCCTGACATTGAACCGTCTTTGCTGATTCACGAAGAAGCCGTCTTTCAAGCGCTAATGGGAATCGACTGGACCACTGCTGATTTCCAGCCAATGGGTAACCAAGATAGAGCTGAAGCGGCGCAAGACGATCCCCAGCCTTCGAACCGAGGCGGCGAACAGGCCTAATCAACGCTCCGGTTCACCTTACTTGGTGACGTTTTAATCCGTCAACGAACAATATATTCAGGCACTACGTTTGCCTTGTAATAAGATAGTTTAATACCTCTGCATTTGAGTGTGCCTTCGCGCACGGGTACTGATGCTCTTTCCAATGACGTTTTCCAACTTTGTTTCCTGTCTTTATTGAATACACAAGATCTTCCGGCCCTATGAACCGTGCCGCGCCCTTGCCAAACCGGGCAACTTGCCTCTGGGTTAAAAAACGGACCAGGCTATGTCAGCTGAATCGGTTTAAAAACACTTGACGTTCCGAAGAACAAAAAACACCACAATAAAGGCAGAAAACCAAATATGAAAAGGCAACGCGATAGTAAAACGCATGCAAATAGTGGCTACTATGAACCGTTCAGAAGGCGTGACCATCGTCAAGACAAAACCGAGCCCCCAGGAGGTGTAGCTACGGTTTAGGTCGACTAGGTTCCTAACTGAATCGTGGCAAATATGCCGGCCAAATGGCATTGCATTGGTTCGTCAACCAAGCCCCCAAGTGACTCAATGGCCAAGGTCCGATCAAGTGGCATTGCGTTGGTTCGGACACGACCAAGCCCCCAAATGATTCATTGGCTAAAAAGCCGACCAGAAGGCATTACCATCATCAAGACATGACCGAGTTCCCATAAGGCGTAATCTACGGTTCAAGTCGATTAGGTCCCCAACTGATTCAGTGGCAAATGTGCCAGCCAGAAGGCATTACCGTCGTCAAGACATGACCAAGCCCCCCGGAGGTGTAGCCACGGTTCAGGCCGACTAGGTCCCCAACTGACTTTATAGGGTTTAGCTATGTTCTCTTTGGTTCGAACCCGACCTATGTTTGAACAGGAAGCCCCATGTTTGAATAGCTATGTTCTCTTTGGTTCGAATACGACCTATGTTTGAACAGGAAGCCTCATGCTGAATGGCTATGTTCTCTTTGGCTCGAATACGGCCTATGTTTGAACAGTAAGCCTTATGCTGAATGGCTATGTTCTCTTTGGCTCGAATACGGCCTATGTTTGAACAGTAAGCCTTATGCTTGAACCGGGATATTGAACCGGATATTAAGAGCTTCACAACCCTGTAGGAGACATCTTCCCTTGAACCGGCCTTTAAACCGGAATCTATAATTTGAACTTCTGTGAAGCGACCTCCAGGGGTAAGCACACATGATGTGAAGAACAGAAGAGACCCCGTTTTACTCGAGGCTTCGGTTTATTGTATTAATCATAATATATACATGGTCATGATATGTACATAAACAGAGCCCAGGGCTCATGTATAATAAGGTCGAAGATGAGCTATGTTCCACGGCCTGCTGGTTTCTTCCACTGATGTGCGTGAATCCTTGCTCTCTCGAATATCAATCAGGTAATATGACCCATTATTCAGATTCTTGCTGACCACAAAAGGTCCCTCCCAAGGCGGGGATAATTTATGCTGATCAGATTGATCCTGGATGAGCCGAAGCACCAAATCCCCCTCCTGAAAGGTTCTGGTCCTAACCCGACGGCTGTGGTAACGGCGAAGATCTTGCTGGTAAATCGCTGAATGGGCAGCTGCCAAGTCACGCTCCTCATCCAACAAGTCAAGAGCGTTCTGCCGTGCCTGCTCATTATCCGCTTCAACATATGCTGCCACGCGAGGTGAGTCATGTCGTATATCACTTGGGAGAACCGCCTCGGCTTCATAGACCATAAAAAACGGCGTAAACCCCGTGGATCTGTTAGGCGTGGTGTTGATGCTCCATAATACAGACGGTAGCTCCTCCACCCAACAACCCGGTGTCCTTTGCAAAGGGACCAGAAGCCGGGGTTTGATACCTCTCAAGATCTCTTGGTTCGCTCGTTCTGCCTGACCATTAGACTGTGGGTGCGCTACCGAAGAAACATCAAGCCGTATATGCTCCCGTGCACAGAACTCCTTCATGGCCCCCTTGGACAGATTGGTACCATTATCTGTGATGATGCTGTGTGGAAAACCAAACCGGAAGATCACCTTCTTTATAAACCGAACCGCCGTGGCCGCATCACACTTACTGACAGGTTCGGCCTCCACCCATTTTGTAAATTTGTCAACCGCCACCAGTAGGTGGGTCTTCTTATCTTTGGAACGCTTAAAAGGCCCAACCATATCAAGCCCCCAAGTCGCGAACGGCCAAGTGATTGGAATCATCCTCAATTCTTGAGCCGGTATGTGCGCTCGTCTGGAAAATTTCTGGCAGCCATCACATAACCGGACCAGGTCTTCCGCATCAGCATGAGCAGTTAACCAGTAGAAACCGTGGCGGAACGCTTTAGCCACCAATGATTTTGAACCGGCGTGGTGGCCGCAATCCCCTTCATGTATTTCTCGTAGTATCTCGCACCCCTCCTTAGGGGATATGCACCGTTGCAGCGCTCCTGACACGCTGCGATGATGTAACTCTCCTTGGAAAATGGTCATAGACTTGGACCGTCGTACTATCTGTCGAGCCAAGCTTTCATCCTCTGGCAACTCACCCCGGTTCATATATGCCAGGTAAGGGAGCGTCCAATCCGGGGTGGCGTGGAGAGCCGCCACTAACTGAGCCTCCGGATCAGGAACAGCCAACTCCAGCTCACCGGGCAGCTGTACCGATGGGTGGTACAATACATCCAAGAAGACATTGGGTGGGACCGGTTTGCGCTGAGAGCCCAAGCGACTTAAAGCGTCCGCTGCCTCATTCTTTCTGCGGTCCACATGATCCACTTGATAGCCTTTGAAATGCCCAGCCACCATATCTACTTCACGTCGGTATGCAGCCATGAGCGGATCCTTAGAATCCCAGGTGCCAGATACCTGCTGAGCCACCAGATCCGAATCACCAAAGCACCTAACTCTGCTCAGATTCATCTCTTTAGCCACCTGAAGACCATGAAGCAAAGCTTCATACTCCGCCGCATTATTTGTACAGGGGAACATTAAACGGAGGACATAACAAAACTTATCTCCTCGTGGGGAAGTTAAAACGACTCCAGCCCCCGAGCCTTCCAATTGTCTTGACCCGTCAAAATGAACGGTCCAATAGGTGTTATCTGGCTTCTCCTCAGGCGCTTGTAACTCTGTCCAATCGTTTATGAAATCAACAAGTGCTTGTGATTTTATGGCCGTCCGGGGTACGTACCTTAAATCGTGCGGCCCGAGCTCGATAGCCCACTTGGCAATCCGACCGGTCGCCTCCCGGTTCTGTATAATGTCACCCAAAGGAGCAGAACTGACCACCGTGATGGGGTGTCCTTGAAAATATTGCCTCAGCTTCCGGCTTGCCATAAAGACACCATATACCAGCTTCTGCCAATGCGGATACCTTTGTTTGGACTCGATAAGTACCTCACTGATATAGTAAACCGGTCGCTGAACCGGATGTTCCTTTCCAGCCTCTTTTCGCTCTACCACCGTCGCCACACTAACCGCTCTGGCATTCGCAGCAACATACAACAGGAGTGGCTCTTTATCTACAGGGGCTGCCAAAACTGGCGGATTGACTAGCTGTTGCTTCAAGTCCTCAAACGCTTTATCAGCCTCCGGACTCCAGACAAATTGATCCGTCTTCTTGAGCATTTGATACAAGGGGATCGCCTTTTCACCAAGGCGACTGATAAACCGGCTTAACGCTGCAATCCGCCCGGCCATGCGCTGAACATCATTGATGCATTTTGGTTTGGCCAGGGAGGTGATGGCTGTGATCTTTTCTGGATTGGCCTCAATTCCTCTATGGGACACTAAAAATCCCAACAGCTTGCCTGCAGGTACACCAAAAACACATTTAGCCGGGTTGAGCATCATTTGGTACACTCTCAAGTTATCGAAAGTCTCCTTCAAATCGTCAACCAGGGTCTCCTTCTTTCGTGACTTGACCACAATATCATCCACATAAGCATGCACATTACGACCAATCTGCTCGTGGAGGCAATTTTGCACACATCGTTGATAAGTAGCCTGCGCACTCTTAAGCCCAAAGGGCATAGACACATAGCAGAAGGCTCCAAAGGGAGTAATGAACGCTGTCTTCTCCTGGTCCTTAACTGCCATCTTGATCTGATGATAACCCGAATAGGCATCCAAAAAACTTAAACGCTCGCAACCTGCCGTAGCATCAATAATCTGATCAATCCGGGGGAGAGCAAAAGGATCTGCTGGGCAAGCTTTATTCAAGTCTGTGTAGTCCACACACATGCGCCAGGTGCCGTTTTTCTTAAGAACTAGCACCGGATTAGCAAGCCATTCGGGATGAAAAACCTCAATGATAAACCCAGCTGCCAGGAGCCTGGCCACTTCCTCACCAATCGCTTTACGTCTTTCTTCGTTAAACCGCCGTAAGAACTGCTTTACCGGCTTGTACTTAGGATCCACATTAAGGGTGTGCTCAGCGAGTTGCCTCGGTACACCAGGCATGTCAGACGGCTTCCATGCAAAGATGTCCCGATTCTCACGGATGAACTCGATGAGCGCGCTTTCCTATTTTGGATCCAAGTTTGCACTGATGCTGAACTGCTTGGATGAGTCGCCAGGTACGAAGTCAACAAGCTTAGTTTCGGCCGCTGACTTGAACTTCAAGGCCGGATCGTGGTCCGTAGTCGGCTTTTTTAATGAAGTCATATCCTCCGGATCAACACTGTCTTTGTACTGCTTTAGCTCCTCCGTAGCACAAACCGATTCTGCATAAGTCGCATCTCCTTCCTCACATTCCAAAGCGATTCGACGGCTCCCATGAACCGTTATAGTGCCCTTGTAACCTGGCATCTTAAGTTGCAGATACACGTAGCAGGGCCGTGCCATAAATTTTGCATAAGCTGGCCGTCCGAACAAAGCGTGGTACGGACTTTGGATTTTAACCACTTCGAACGTCAGCGTCTCTGATCTGGAATCGTGACTATCGCCAAATACCACTTCAAGGGCTATCTTACCAACCGGATATGCAGACTTGCCAGGCACCACCCCGTGGAACACTGTATTGGTCGGTTTGAGATCCTTATCTACCAGTCCCATACGACGGAAGGTCTCGTAATACAAGATGTTAATGCTGCTCCCTCCGTCCATGAGCACCTTGGTGAGCTTATAACCTCCCACCTGAGGCGCCACCACCAATGCTAACTGACCCGGATTATAAACCCTGGGAGGGTGATCCTCTCGGCTCCATATGATAGGCTGTTCTGACCAGCGCAGATAATGAGGCGTTGCCGGTTCAATAGCACTGACTGCTCGCCTCTGAAGCTTTCTATCCCGTTTATCCAAGCTTGTAGTAAAAACATGATACTGTCCACCGCTCAACTGTTTCGGGTTGCTCTGATAACCACTCTGATTACTTTGATTATTTTGGCTGACCTGTCCGCCCGGATTACCCTGGAACCCTGGACCGGAATTTCCGCCACCGTACCCTGATCCGGAACCGGAACCGCCGCCGGAGCTGTGATCATATTGGAAAGTGTTTGAGCTTTTGAATTCCTTCATGATATAACAATCCTTCCAAAGATGGGCTGCTGGTTCCTCCTTTGTTCCGTGCTTCGGACAGGGCTGGTTTAAGAAAAAGTTCAAACGCTCCGGATTAGGACCGGGATGTGCGCGGAAACTTGGCTGTTTCCCCTTACGCCGCTGGCCTTTATCCTGTGCACTTGTGCTGGCCACCAAGTCCATGCTCCCGTCCGGTTTACGCTTTCCTCCGCCTCCATTACCTGTCGGCCGATGCTGCTGGCCTTTGGTGTTGCTATTCTTCTTTCCCTTCCCTGCTTTGTCATCACCAGAGTCAGGATCCTTGGTACTGTCAGAATCCGCATACTTCACTAAAGCGGTCATGAGCGTTCCTATATCTGTACAATCACGCTTCATCCGGCCCAGCTTTAGCTTCAAAGGGCCAAACCGACAGTTGCTCTCCAACAGCACCACGGCGGTGTCTGCGTTGATGCGATCTGACGAGTGCAAAACTTCTGATACCCGCCGTACCCAATGGGTAGTTGATTCCCCTTCTTGCTGAACGCAGGCTGCTAGGTCAACTATTGACATTGGCTGTTTGCACGTGTCCTTGAAATTCTTGATAAACCGGGCGCGTAATTGGGCCCATGAACTAATTGAATTAGGCGGTAAGCTCTTTAGCCAAGTCTGGGCCGTTCCTTCTAACATCATAGTGAAATACTTGGCACACGCCGTGTCATCCACATCCAGCATCTCCATAGCCATTTCATAGCTCTCCACCCATGTCTCCGGAGGCTGATCTGCCGTGTAGTTTGGTACCTTGCGGGGGCCTTTGAAGTCTTTGGGCAGGCGTACGTTGCGTAAAGCGGAAACAAGGCACGGTACTCCCAAAGAACTGGAAACCACCCCCGGTTCAACCGTAGCTGCGGGGAAGATAGGTGTAAGCTGACGAGCCTGATGCTGCGCTGCTAATCCGGCCTCCCGTGCACGGGTGCGATCCACATTCTCGTGAGTATTGTCGACGCCCGCCGGATTGTTGGCGCGGAGCGCTTCACGCCTGGTGTTACTTGAAACGGCCGGCTCCTCCACGCGCCTACTATAGCTCCTGCTGGGGCGAGGGGTGGAGTGAATCCGATCACGGCTTTGTGAATACGCTTCTTGCTGGGCCAACGCGGTCCTAAGGAGCTCCTTGACCCGGCGTGTCTCTTCATCCTGCGGTGAATCTCCTTCGATCGGAATGGCTTCCAACCGAGCCGCCGCCGCGACGAGGTTATCCATCGGGTTGGAATAATGACCTGACGGCGTTGGCATGGCCGGTGGCGTATCAGTGTTATGACGAGGCAGGTCTATAATCCGGTGCTGAACCGGGGCGCCGGCTGTCGCCTCCGCACGGTTTACCCCCGGGGTGTTGAAGAGATTTCGAGCGTCGAGGGCGGCGGGCAACCGAGACCGGGATTTTCTCTTCAGGACCTCCTGAGACGCCTTCTGGTCTATCATGAGCCTGTAAGCCTGTGCATCTAAAGCGGCGCGCTCCGCCGTCATCCTGATCTCTTCTGCTGCCAGCTCCGCTTTAGCCCGAGTGATCTGCTCTCTCACTTTGGCAATCTCAGCATTGTGAACCTCCTGATCCACCAGGTTGGCTTCTGCCATGAGTGCTGCTAGTGCGTCAAACAGGTCTGACAAAACTTGGGCTGGTGAACCCGTAGAGCCTCCTGCCCGCGCAGCCGTTGCTGCTGCCGATCCGGAGATCGCTGCTGCTGCAGTTGATGATCGTGGCGCCGCCTGTGTCCCAGCCATGAAGATCCCAACCCGATAGGGCAGATCAGAGGGGTCCGGAATACTGTTGCCATCGGAGCCGCCCCCAACCCGATCATCTTGTAGCTGGTAAAGAGATTCGGTCTCCCCGGTCGACGTTTCATCGCCAGAGTAGATGGCGGTTTCGTCACCAGATTCGGATCCTTCCTCGTAGCTTCCCCCGTGGATGACTCCCACGAAGGCGTGCTTCCTGGTCGGTTTAGCCAGGGTCGATCTCGTGCACTGAGCTGTCTCGATGAGGCCGGCGCAGACGCCTAACTCGGGGCCCGGTTCTCCGATCTTGCCGATGAAAACATGGATGCCGCCAAAGGGGACCCGGTACCCGTACTCGATCGAGCCGGCGTCGGGGCCCCAGCCTGTGCTGTCGATGTAGAGTTTTCCGCGGCGGCTCTTGGTCATCCGTCCCACAGCGTAACCCTCGAGTCCTTCAAAGCGGCCCTCCAAGAACCTGAAACCATCTCGCGATGGCCCCACGGTGGGCGCCAAC

TGTCGTGGATTTGTCACGGCAGATGTCCTAGTAAGAGGACTTAGTCGTGGAGCCATCGCAACGGGTTAGCTTGAAGGGGTTAAAGTGGACACAAGGACACGGTTTTTATACTAGTTCGGCCCCTTCGATGAAGGTAAAAGCCTACGTCTAGTTGTGATGGGATTGATGGGTTTCGAGGGCTTAGGGAGCGATAAGCTTCGCCTAAACCTCGAGTTCTTTTTCTCTGTCTCCAAACCGCCGCCGGGTCATCCCCTTATATACACGGGTGACGCCCGTCGGTCTGCAAAGTCCCAACCTCCGGCTTATAAACGTGCCCGGGTCGGTCTCTCTATTCCTAACTTACAACACAAGTTTACATCAGGCCGGTTTATACCATAAACCTTTAAACCGATTATGGGCCTTAGGCCCTTCTATCTCTCCTTGGGCTTTAACATCTCGGGTCTGTTGACGGAGGTAACCCGGGCCCTGCAGGCCGGTTTACCCCGGATAGCAACATCCCCAACA

GGTAGTGCATTTGAGGTAGTAGTAACTTCAGGTTGTAGTTCCTGAATGACCACAGATGATGGAGGCATCTGCTCAAAGTGAATTCCAGTGTGATCAGAAGAAACCAAAACTGGAACTTCAGCAC

>Aegilops umbellulata 1-077;

CAGGAAGACCAGGCATATCAAGCGTCGCTTCAACTCCATTCGTGAAAGTGTTCAAAATGGAGACATAGAGATTTGTAAAGTACATACGGACCTGAATGTGGCGGATCCGTTGACTAAACCTCTC

TGTCGTGGATTTGTCACGGCAGATGTCCTGGCAAAAGGACTTAGTCGTGGAGCCATCGCAACGGGTTAGTTTGAAGGGGTTAGAGCGGACGCAAGGACACAGAGTTTATACTAGTTCGGCCCCTTCAATGAAGGTAAAAGCCTACGTCTAGTTGTGATGGGATTGATGGGTTTCGAGGGTTAGGGAGCGATAAGCTTCGCCTAAACCTCGAGTTGTTATTCTCTGTCCCCAAACCGCCGTCGGGTCGTCCCCTTATATACACGGGTGACGCCCGTCGGTCTGTAAAGTTCCAACCCCCGGTTCATAAACGTGCCCGGGTCGGTCTCTCTATTCCTAACTTACAATGCAAGTTTACCTCAGGCCGGTTTACGCCCTGAGACCTCTAAACCGATTATAGGCTTTGGGCCCTTCTATTTCTCCGGGGGCTTTAACATCTCGGGTCCATTGACGGAACTAACCCGGGCCCTGTAGGCCGGTTTACCTCAAATAGCAATATCCCCAACA

TTAGGCCCCAGATTGATTTGAACAAGTTCATGTCAATCCTTAATAAGAATTTCTGTCTTCAAATGTCTTCCAGTAGCTGTTGAACTGCCGTTTCTTCGTCTCCTTTTGCTGCGATAAACCGATATGACGTCAGTGCAAAGTTACCGTCTCCTTTTGCTAATCCGCAATAATCAGGGCGACGGCTGGGCTCCCGAAATCTCCGGCCCCTGGATTCGCGCGCCTGGCACGCGCGCCGCGCCTTATAAATAGGACTTGAAGGCCATCTTCCTTTCCTCCCTTTCGCCCCCTTCTTCGTCTTCCTCGCGACCCAGACCTCGGAGCTCCGCCGCCGCCGTCAGCTACTGCATCAACTCCGGCCGCTGCATCAGCCTGATCGGACCAGAGCACCGCGGCGACCTTTCACATCCGTTTGGATCCCGGTAAGGCCCCTGTTCTTCTTGCTTTTAGATCTGTTCTAGGGTTCGTCATCCTGCCACTGTTCTTTGTTCATCCCCATAGTAGCTTTGACGCTAGGACGACATCGTCAGTTCGATACCCGCTGAGAGCTTTTGCGAACAGATGATGATGCTATGTACTCATCTCTATAAATCTGTACTCATCTCCTTAGATCCCACTCATTTCCCTGCTTGTCAAACTTATGTTGTTCTGCTGTGAGCCAAACCTCATATTTTTACTTCTTTTTCGCCCTAGCTGTAGATCCATTGCTTTGAAAGAATCTGTATGGGATCTGTTTCTGCTTTCATATTCACAACTGTTAGATCTGGTCGGTTTAACTTCTAGCAAAGCGAGCAAACCAATCCACACATCCATTAGTCCCCTTGAACAACCGCTGGATGATATCATCATAGATAACCTTACTCTATATAAGAGCATCTTCGGTTTACATTCACACTTGTTCTCAAACCGGAACACCTTTGTCTTGTAGATTTCCTCATGGCAAAACAGGTGTACGAATGCAACTGGGTTCCCTCTCGCATCACTGAGACCCAACTAAACAACTTAGTTCTGACTGGCGCTTTGAGTAGCAAAAATGCCATCCATTGGAGGGTTCCAGGAAACGAATGTCCTCCTACCCCACAGGAGGGAGAAGTGGTGGTGTTTGCGGATCACCTGGCCCGGGGTTTTAATCCACCCGGTTCAAAATTTTATCGGGACGTCCTGGCTAACTTTAGGTTGCGCCCACAAGACGTTGGTCCAAACTCAGTCACGAATATCTGCCATTTTCAAGTGCTTTGCGAGGTGTACTTCCAGGAGGAACCTATTGTAGAGCTGTTCCGGGACCTGTTTCATTTGAACCGACGTACTGAATTCACCGACGGCCCTAATATGGAACTGGGTGGTATGACGATTCAAAAGAGGAAAGAGGTGACTTATCCTCACGCCAAGTTGCATAGTCATCCGCAAGAATGGAATTACACTTGGTTCTATTGCAAGGATACTTCTCCTGCTGAAGAAAATCCTCTGCCTGGCTTTCGTCCGGAGAGACTTAGCAATACACACCCCTTCCCCCCAAGACTAACCGCCAAAGAGAGGAGTAAGTATGCCCCTCAGCTGTCCAAGCTCAGAGCCTTCATGGCTAACGGTTTAACGGGGGTAGATCTTGCCCGTTGCTGGATTTCATGGAGCATCCTGCCCCTTAGCATCCGCTCCGGTTTAATGTGCCAATACACGGGCCGGGTTGATGATCCTTTGAGGCACTGCAACATTCAGCTGACGGAAGATGAAGTCACTGAGGCTGTGAAAAAGATGCTGAATGAACCGGAATACGTGTGCGCCCGAACCGGCCTGCTTGCATTTTGTGCCACAAACAAACCACCTGCTGTAAGTACCTCCCCTCGGTTTGTTATAGAATTTATGATCTCCTTATACACATATTGCTAACACCGGTCCTTTCTGAGACAGGGAGATGATCCGTTTTGGAGCAAGAAGTTTCAAGAGCCGACCGAAAAGGTAACCCGACCCAAAACGAGGGTGAAGAAACCCGCTCAGAAGAAAAGGAACACCGCTTCCTCTACCCCAGATTTAGAGGAGGATGATGTGGGTAATCCGGACCTTGGGGTAGACCTCGATTCTGTTGGCTTACTTTTTATACGTCTTATTGATGATGATATTTGTCAGGACGACGCTGAAGCGAGCAATGCTGAAGGCCCAGAGGTAACTATTCTTTCTTCTGATGCAGTTCCTTTGCCTTTGAAAAAACCTCGCCAAGCAAACCGGAAAATTAAATTTTCTCATCCTCTTGCTTACTTGGATCCCAAATTTCTTGTGAAGACCCAGCAACATGAAGCTCGACGCACCACCCGGCATAGCGGCCAGGTAGTTACCTCCGCCGGTTTACCGAACAACCCGGTTCGGAAACGCCGCTCAGAGGTCTCCGATTTATGCGCTAGCTTTCCTCCTAAAGCAGGTTTAACTCGTCAGCCTCTTAATCCATCTGACTCCGATTATCAGGTCACTTCCCATTCATCTTCTGGTGAATCCTCAGCCACTCAACTGCCACCGCTAAAGACGGTTATTGGGTAAGTTGGACCGATCACAACATATTTCTGTATGAGTCTCTTAATCCATCTTACACCGTTTTTTCAATCTTTGGGTTTTTCCCAGGGCTAAACCTAGACCGAGCAAGAAGGCTCGTCTGGACAACCCGGCCGAAGGAACCGCCGCTCCTGAACCGGAGAAAACGCCTGGTTCTGACGCTCTGGGTCGTGAAGATGTTCAGAATTATCTATCTCCACAAGATGACACCTGTGTTGAAGAAAGGCCTACCAACCCTGCCAGCCGTACTGATCCGCCTGCTAGCCCTGTTCGGGTTGAGGGATCCATCCCCCCTTCTGGAACTGTTGATAAACCGACAGGGCCAATGCAGAGCAGTGACTCCAAGGATGATGATGTTGTGATTACCGGCATTGGTCGCTCTAAACCGGGGAACACTGCCACTTTAGCCAAGCATACTGCCAAGGACGAAATCTCGGCCATAAAAGGCAAATGGGATGTTGACTCCTCAACATATGCCGCTTTAAGCGCCCAAGATCTTTATTCTGGGTACCTGAACCGACTGTACACCAGTCGCGATTATGAAGCTGGTATGATCAGGATGATGAAAGAAAAACTTGAGGTAACTCTCTTGTGCTTCCTTCTGTTACAACCTAGCTTCTCAATCTGATCCGAATCCGAGATGGTAACCCGAATCATGCTGCCTTGACCACTAAGGTCCGGTTTAAAAACCCAGGGCTTCAACATAGGCCTTTAACCAGAAACATCATTAAATAACCATTAGCCCCCAAGTGCCAAGTTTTAATACTTGTATTAATCCTGGGACTTTTTGTAGTGGAAAACATGTGTTAAAATCAAGTATGCATTAGCCCCCAAGTATCGAGCACATAACTTGCTTGTGTGGTTGATACTTCCAAACCGTGCACTATTTTCTGAACACTGCTGATGACCTGCTGCAGGGGGATTTGAAGACCAAAGAGAAACAAGTCTCGGACCTTCAGGAAACTTTAAAAACCCAGCAGGCTGAAACTGAAAAAGCGAAGGAGGAATTGACCAATGCCTTAACCGTTATGGAACAGCTGAAAGAAAACCGCAAAAAGGAGCAAGCTGTTTGGGCTACTGAAAAAGCTCTTTTGACGAAGAGGGCTGAGAATGCTAAAGCTGCTCTCAAACCGGTGGTCGATGAATTATCCACCGTGAAGCGACAAATACACTCCATGACCTCTGCCGTCTTTGGTAAATTTCTTTCGATGCCCTTCTTCCAATTTGTACCTTGTCTGCTATATCCTATGTCCGGTTTGTAATCCATCTTGAAACACTGCAGGCTCACGTATTGGACACTTGGGTTCTGATGTGCGGATGAAACTCAAAGCTGCCTATACTCTGATTGAACAGTTGTACACCGGAGCACAGAGAGCTATCACCACTGCGTCGCACAACCACCCTGCACCGTCTCTGATTCAAGACACGTTAAAGAGTCTATCCATGCTTCCAGCCCGGATTGAAGAGTTGAAAAGATCAGCTGCTCGAACTGGAGCCATCACTGCCTTGATCCGGGCCAAAGCGTGGGTACCAGATTTCGATCCTGTCGAAGCGGCCCAAGGATACCCCAGCTTGAAAGAAGATGGTTCAGACTTTGGTGAAGATGATTTGAGGGCGATAAACCGGGCGGTGCGCCCCCTAGCTTGTCAATTGGCTGAAGAAGCAGACCTGAACCATTACCAAGCCCAATATGATAGCCAGAATAGGCGAGTAGCTGCCCCGGTCCCTGAAGCGGAAAACCTTATTCCTCCAATCCGTAAGCATACCTACGCCCCTGACATTGAACCGTCTTTGCTGATTCACGAAGAAGCTGTCTTTCAAGCGCTAATGGGAATCGACTGGACCACTGCTGATTTCCAGCCAATGGGTAGCCAAGAGGATGCGGCGCAAGATGATCCACAACCTTCGAACCGAGGCGGCGAACAGGCGTAATCAACGCTCCGGTTCACCTTAATTGGTGACGTTTAATCCGTCAAAGAACAATATATTCAGGCACTACGCTGCCTTGTAATAAGATAGTTTAATACCTCTGCATTTGAATGTGCCTTTGCGCACGGGTACTGATGCTCTTTCCAATGACGTTTTCCAACTTTATGTTTCCTGTCTTTACTGAGTACACAAGATCTTTCGACCCTATGAACCGTGCTGCGCCTTTGCCAAACCGGGCAACTTGCCTCCGGGTTAAAAAACGGACCAGGCTGTGTCAGCTGAATCGGTTTAAAAGCACTTGACGTTCCGAAGAACAAAAAATACCACAATAAAGGCAAAAAACAAAAACCAAATACGAGAGGCCAACGCGATAGTAAAACGCATGCAAATAGTGGTTGCTATGAACTGTTCAGAAGGCGTGACCATCGTCAAGACAAAATAAACCAAGCCCCCAAGAGGTGTAGCTACGGTTTAGGTCGACTAGGTTCCTAACTGAATCATGGCAAATATGCCGGTCAAATGGCATTGCATTGGTTCGGATTCGACCAAGCCCCCAAGTGACTCAGTGGCCAAGGGCCGATCAAGTGGCATTGCGTTGGTTCGGACACGACCAAGCCCCCAAATGATTCATTGGCTAAAAAGCCGACCAGAAGGCATTACCATCATCAAGACATGACCGAGCTCCCATAAGGCGTAATCTACGGTTCAAGTCGATTAGGTCCCCAACTGATTCAGTGGCAAATGTGCCAGCCAGAAGGCATTACCGTCGTCAAGACACGACCAAGCCCCCCGGAGGTGTAGCTACGGTTCAGGCCGACTAGGTCCCCAACTGACTTTATAGGGATAGCTATGTTCTCTTTGGTTCGAATACGACCTATGTTTGAACAAGAAGCCCCATGTTTGAATAGCTATGTTCTCTTTGGTTCGAATACGACCTATGTTTGAACAGGAAGCCCCATGTTTGAATAGCTATGTTCTCTTTGGTTCGAATACGACCTATGTTTGAACAGGAAGCCCCATGCTTGAACCGGGATATTGAACCGGATATTAAGAGCTTCACAGCTTTGCGGGAGACATCTTCCCTTGAACCGGCTTTTAAACCGGAATCTTTAATTTGATCTTCTGTGAAGCGACCTCAAGGGGAAGAACACGCATGATGTGAAGAACAGCAGAGACCCCGCTTTACTCGAGGCTCCGGTTTATTGTATTAATCATAATATATACATGGTCATGATATGTACATAAACAGAGCCCAAGGCTCATGTATAATAAGGTCGAAGATGAGCTATGTTCCACGGCCTGCTGGTCTCCTCCTCTGATGTGCGCGAATCCTTGCGCTCTCGAATATCAATCAGGTAGTATGACCCATTATTCAGATTCTTGCTGACCACAAAAGGTCCCTCCCAAGGCGGGGATAATTTATGCTGATCAGACTGATCCTGGATGAGCCGAAGCACCAAATCCCCCTCCTGAAAGGTTCTGGTCCTAACCCGACGGCTGTGGTAACGGCGAAGATCTTGCTGGTAAATCGCTGAACGGGCAGCTGCTAAGTCACGTTCCTCATCCAACAAGTCAAGAGCGTTCTGCCGTGCCTGCTCGTTGTCCGCTTCAACATACGCTGCCACACGAGGTGAGTCATGTCGTATATCACTTGGGAGAACCGCCTCTGCTCCATAAACCATAAAGAACGGCGTAAACCCCGTGGATCTGTTAGGCGTGGTGTTGATGCTCCATAATACAGACGGTAGCTCCTCCACCCAACAACCCGGCGTCCTTTGCAAAGGGACCAGAAGCCGGGGTTTGATACCTCTCAAGATCTCTTGATTCGCTCGTTCTGCCTGACCATTAGATTGTGGGTGCGCTACCGAAGAAACATCAAGCCGTATATGCTCCCGTGCACAGAACTCTTTCATGGCCCCCTTGGATAGATTGGTACCATTATCTGTGATGATGCTGTGTGGAAAGCCAAACCGGAAGATCACCTTCTTTATAAACCGAACCGCCGTGGCAGCATCACATTTACTGACAGGTTCTGCCTCCACCCACTTTGTAAATTTGTCAACCGCCACCAGTAGGTGGGTCTTCTTATCTTTGGAACGCTTAAAAGGCCCAACCATATCGAGCCCCCAAGTCGCAAACGGCCAAGTGATTGGAATCATCCTCAATTCTTGAGCCGGTATGTGTGCTCGTCTGGAAAATTTCTGGCAACCATCACATAACCTGACCAGGTCTTCTGCATCAGCATGAGCAGTTAACCAGTAGAAACCGTGGCGGAACGCTTTAGCCACCAATGATTTTGAACCGGCGTGGTGGCCGCAATCCCCTTCATGTATTTCACGTAATATCTCACACCCCTCCTCAGGGGATACGCACCGTTGCAGCGCTCCTGACACGCTGCGATGATGTAGCTCTCCTTTGAGAATGGTCATAGACTTGGACCGCCGTACTATCTGTCGAGCCAAGCTTTCGTCTTCTGGCAACTCACCCCGGTTCATATATGCCAGATAAGGGAGCGTCCAATCTGGGGTGACGTGAAGAGCCGCCACTAGTTGAGCCTCCGGATCAGGAACAGCCAACTCCAGCTCACCGGGTAGCTGTACCGATGGGTGATACAATACATCCAAGAAAACATTGGGTGGGACCGGTTTACGCTGAGAGCCCAAGCGGCTTAAAGCGTCTGCTGCCTCATTCTTTCTGCGGTCCACATGGTCCACTTGATAGCCTTTGAAATGCCCGGCTACTATATCCACTTCACGTCGGTATGCAGCCATGAGCGGGTCCTTTGAATCCCAGGTGCCAGATACCTGCTGAGCCACCAGATCCGAATCACCAAAGCACCTAACTCTGCTCAGATTCATCTCTTTAGCCATCCGAAGACCATGCAGCAAAGCTTCATACTCAGCCGCATTATTTGTACAGGGGAACATTAAACGGAGGACATAACAAAACTTATCACCTCGTGGGGAAGTTAAAACGACTCCAGCCCCCGAGCCTTCCAATTGTCTTGACCCGTCAAAATGAATGGTCCAATAGGTGTTATCTGGCTTCTCCTCAGGCGCTTGTAACTCTGTCCAATCGTTTATGAAATCAACAAGTGCTTGTGACTTTATGGCCGTCTGGGGTACGTATCTTAAATCGTGCGGCCCGAGCTCGATAGCCCACTTGGCAATCCGACCGGTCGCCTCCCGGTTCTGTATGATGTCACCCAAAGGAGCAGAACTGACCACCGTGATGGGGTGTCCTTGAAAATATTGCCTCAGCTTCCGGCTTGCCATAAAAACACCATATACCAGCTTCTGCCAATGCGGATACCTTTGTTTGGACTCGATAAGCACTTCACTGATGTAGTAAACCGGTCGCTGAACCGGATGTTCCTTTCCAGCCTCCTTTCGTTCTACCACCATCGCCACACTAACCGCTCTGGCATTCGCTGCAACATATAACAGGAGTGGCTCTTTATCTACAGGGGCTGCCAACACTGGCGGATTGACTAGCTGTCGCTTCAAGTCCTCAAACGCTTTATCAGCCTCCGGACTCCAGACAAATTGATCCGTCTTCTTGAGCATTTGATACAAGGGGATCGCCTTTTCACCGAGGCGACTGATAAACCGGCTTAACGCTGCAATCCGCCCGGCCATGCGTTGAACATCATTGACGCATTTCGGTTTGGCCAGGGAGGTGATGGCTGTGATCTTTTCCGGATTGGCCTCAATTCCTCTATGGGACACTAAAAATCCCAACAACTTGCCTGCAGGTACACCAAAAACACATTTAGCTGGGTTGAGCATCATTTGGTACACTCTTAAGTTATCGAAAGTCTCCTTCAAATCGTCAACCAGGGTCTCCTTCTTTCGTGACTTGACCACAATATCATCCACATAAGCATGCACATTACGACCAATCTGCTCGTGGAGGCAATTTTGCACACATCGTTGATAAGTAGCCTGCGCACTCTTAAGCCCGAAGGGCATAGACACATAGCAGAAGGCTCCAAAGGGAGTAATGAACGCCGTCTTCTCCTGGTCCTTAACTGCCATCTTGATCTGGTGATAACCCGAATAGGCATCCAAAAAACTCAAACGCTCGCAACCTGCCGTAGCATCAATGATCTGATCAATCCGGGGGAGAGCAAAAGGATCCGCTGGACAAGCTTTATTCAAGTCTGTGTAATCCACACACATGCGCCAGGTGCCGTTTTTCTTAAGAACCAGCACCGGATTAGCAAGCCATTCGGGATGAAAAACCTCAACGATAAACCCAGCTGCCAGGAGCCTGGCCACTTCCTCACCAATCGCTTTACGTCTTTCTTCGTTAAACCGGCGTAAGAACTGTTTTACCGGTTTATACTTAGGATCCACATTAAGGGTGTGCTCAGCGAGTCGCCTCGGTACACCAGGCATGTCAGACGGCTTCCATGCAAAGATGTCCCGATTCTCACGGATGAACTCGATGAGCGCGCTTTCCTATTTTGGATCCAGGTTTGCACTGATGCTGAACTGCTTGGACGAATCGCCAGGTACGAAGTCAACAAGCTTAGTTTCGGCAGCCGATTTGAACTTCAAGGCCGGATCGTGGTCCGTGGTTGGCTTCTTCAACGAAGTCATGTCCTCCGGGTCAACACTGTCTTTGTACTGCTTTAGCTCCTCCGTAGCACAAAACGATTCTGCATAAGTCGCATCTCCTTCCTCGCATTCCAAAGCGATTCGACGGCTTCCATGAACCGTTATAGTGCCCTTGTAACCCGGCATCTTAAGTTGCAGATATACATAGCAGGGCCGTGCCATAAATTTTGCATAGGCTGGCCGTCCGAACAAAGCGTGGTACGGACTTTGGATTTTAACCACTTCGAACGTCAGCGTCTCTGATCTGGAATCGTGACTATCGCCAAAGACCACTTCAAGGGCTATCTTACCAACCGGATATGCAGACTTGCCAGGCACCACCCCGTGGAACACGGTATTGGTCGGTTTGAGATCCTTATCTACCAGTCCCATGCGACGGAAGGTCTCATAATACAAGATGTTAATGCTGCTCCCTCCGTCCATGAGCACCTTGGTGAGCTTATAACCTCCCACCTGAGGTGCCACCACCAATGCTAACTGACCCGGATTATAAACCCTGGGAGGGTGATCTTCTCGGCTCCATATGATAGGCTGTTCCGACCAGCGCATATAGTGAGGCGTGGCCGGTTCGATAGCACTGACTGCCCGCCTCTGAAGCTTTCTATCCCGCTTATCCAAGCTTGTAGTAAAGACATGATACTGTCCACCACTCAACTGTTTCGGGTTGCTCTGATAACCTGATTGCTGCTGTCCGTAACCACTTTGATTATTTTGATTATTTCGGCTGTCCTGCCCGCCCAGATTACCCTGGACCCCTGGACCGGGATTTCCGCCACCGTATCCTGATCCGGAGCCTGAACCGCCGCCGGAGCTGTGATCATATTGGAAAGTGTTTGAGCTTTTGAATTCCTTCATGATATAACAATCCTTCCAAAGATGGGTTGCTGGTTCCTCTTTTGTTCCGTGCTTCGGACAGGGCTGGTTTAAGAAAAAGTTCAAACGCTCCGGATTAGGACCGGGATTTGCGCGGAAACTTGGCTGTTTCCCCTTACGCCGCTGGCCTTTATTCTGTGCACTTGTGTTGGCCACAAAGTCCATGCTCCCGTCCGGTTTACGCTTACCTCCGGCTCCATTACCTGTCGGTCGATATTGCTGGCCTTTGGAGTTGCTATTCTTCTTTCCCTTCCCTGCTTTGTCATCACCAGAGTCAGGATCCTTGGTACTGTCAGAATCCGCATACTTCACTAAAGCGGTCATGAGCGTTCCTATGTCCGTACAATCACGCTTCATCCGGCCCAGCTTTAGCTTCAAAGGGCCAAACCGACAGTTGCTTTCCAACAGCACCACGGCGGTGTCTGCATTGATGCGATCTGATGAGTGCAAAACTTCTGACACCCGCCGCACCCAATGGGTAGTTGATTCCCCTTCTTGCTGAACGCAGGCTGCTAGGTCAACAATCGACATTGGCTGTTTGCACGTGTCCTTGAAATTCTTGATAAACCGGGCGCGCAATTGGGCCCACGAACTGATTGAATTAGGCGGTAAGCTTTTTAGCCAAGTCCGGGCCGTTCCTTCTAACATCATAGTAAAATACTTGGCACACGCCGTGTCATCCACATCCAGCATCTCCATAGCCATTTCATAGCTCTCCACCCATGTCTCCGGAGGCTGATCTGCCGTGTAGTTTGGTACCTTGCGGGGGCCTTTGAAGTCTTTGGGCAGGCGTACGTTGCGCAAAGCGGGAACAAGGCACGGTACTCCCAAAGAACTGGAGACCACCCCCGGTTCAACCGTAGCCGCTGGGAAGATAGGTGTAAGCTGACGAGCCTGATGCTGCGTTGCTAATCCGGCCTCCCGCGCACAGGTGCGATCCACATTCTCCTGAGCATTGTCGACGCCCGTCGGGTTGTTGCCGCGGAGCGCTTCACGCCTGGCATTGCTTGACACAGCCGTCTCCTCCATGCGCCTACTATGGCTCCCGCTGGGGCGAGGGGTGGAGTGAATCCGATCACGGCTTTGTGAATATGCTTCTTGTTGGGCCAACGCGGTCCTAAGGAGCTCCTTGACCCGGCGTGTCTCTACGTCCTGCGGTGAATCGCCTTCGATCGGAATGGCTTCCAGCCGAGCTGCCGCCGCGACGAGGTTATCCATCGGGTTGGAATAATGACCTGACGGTGTTGGCACGGCCTGTGGTGTACCAGTGTTGTGACGAGGCAGGTTTATTATCCGGTGCTGAACCGGGGCGCCAACTGTCGCCTCCGCACGGTTTACCCCCGGGGTGTTGAAGAGATTTCGGGCGTCGAGGGCTGCGGGCAATCGAGATCGGGATTTCCTCTTCAGGACTTCCTGAGACGCATTCTGGTCCATCATGAGCCTGTAAGCCTGTGCATCCAAAGCGGCGCGCTCCGCCGTCATCCTGATCTCTTCTGCTGCCAGCTCCGCTTTAGCTTGAGTGATCTGCTCTCTCACTTTGGCAAACTCAGCATTATGAACCTCCTGATCCACCGGGTTGGCTTCCGCCATGAGCGCTGCCAGTGCGTCAAACAGGTCTGACAAAATTTGGGCTGGTGAACCCGTAGGGCCTCCTGCCCGCGCAGCCGTTGCCGCTGCCGATCCGGAGATCGCTGCTGCTGTAGTTGATGATCGTGGCACCGCCTGTGTTCCAGCCATGAAGATCCCAACCCGACAGGGCAGATCAGAGGGATCCGGAATACTGTTGCCATCGGAGCCGCCCCCAATCCGATCATCTTGCAACTGGTAAAGAGATTCGGTTTCCCCGGTCGACGTTTCATCGCCGGAGTAGATGGCGGTTTCGTCACCGGATTCGGATCCTTCCTCGTAGCCTCCTCCGTGGATGACTCCCACGAAGGCGTGCTTCCTGGTCGGTTTAGCCAGGGCCGATCTCGTGCACTGAGCTGTCTCAACGAGGTCGGCGCAGACGCCTAACTCGGGGCCTGGTTCTCCGATCTTGCCGATGAAAACATGGATGCCGCCAAAGGGGACCCGGTACCCGTACTCGATCGAGCCGGCGTCGGGGCCCCAGCCTGTGCTGTCGATGTAGAGTTTTCCGCGGCGGCTCTTGGTCATCCGACCCACAGCGTAACCCTCGAGTCCTTTGAAGCGGCCCTCCAAAAACTTGAAACCATCTCGCGATAGCCCCACGGTGGGCGCCAAC

TGTCGTGGATTTGTCACGGCAGATGTCCTGGCAAAAGGACTTAGTCGTGGAGCCATCGCAACGGGTTAGTTTGAAGGGGTTAGAGCGGACGCAAGGACACAGAGTTTATACTAGTTCGGCCCCTTCAATGAAGGTAAAAGCCTACGTCTAGTTGTGATGGGATTGATGGGTTTCGAGGGTTAGGGAGCGATAAGCTTCGCCTAAACCTCGAGTTGTTATTCTCTGTCCCCAAACCGCCGTCGGGTCGTCCCCTTATATACACGGGTGACGCCCGTCGGTCTGTAAAGTTCCAACCCCCGGTTCATAAACGTGCCCGGGTCGGTCTCTCTATTCCTAACTTACAATGCAAGTTTACCTCAGGCCGGTTTACGCCCTGAGACCTCTAAACCGATTATAGGCTTTGGGCCCTTCTATTTCTCCGGGGGCTTTAACATCTCGGGTCCATTGACGGAACTAACCCGGGCCCTGTAGGCCGGTTTACCTCAAATAGCAATATCCCCAACA

CTCTCCCTAGAGCAAAACATGATCAACACCAAAATTCCATGGGTGTGCGATTCATCACAATGTAACTAGACTATTGTCTCTAGTGCAAGTGGGAGACTGTTGGAAATATGCCCTAGAGGCAATA

>Aegilops umbellulata 1-131;

TTCAGGCCCCGGACATCCGGCGCGACCCCAGACATCCGGCGCGACCCCGGACATCTGGCGCCTCACCAACACCCGGACATCCGGCCTCTTCCCTGGAAATTCGGCATCGACGACAGAACGCACC

TGTCGTGGATTTGTCACGGCAGATGTCCTAGCAAAAGGACTTAGTCGTGGAGCCATCGCAACGGGTTAACTTGAAGGGGTTAAAACGGACGCAAGGACACAGAGTTATACTAGTTCGGCCCCTTCGATGAAGGTAAGAGCCTACGTCTAGTTGTGATGGGATTGATGTGTTTCGAGGGCTAGGGAGCGATAAGCTTCGCCTAAACCTCGAGTTGTTGTTCTCTGTCTTTAAACCGCCGTCGGGTCGTCCCCTTATATACACGGGTGACGCCCGTCGGTCTACAAAGTCCCAACCTCCGGTTCATAAACGTGCCCGGGTTGGTCTCTCTATTCCTAACTTACAATGCAAGTTTACCTCAGGCCGGTTTACGGCCATAAACCTTTGAACCGATTATAGGCCTTGGGCCCTTCTATTTCTCCTTGGGCTTTAACATCTTGGGTCCATTGACGGAGCTAACCCGGGCCCTGTAGGCCGGTTTACCCCAAATAGCAATATCCCCAACA

TTAGGCCCCAGATTGATTTGAACAGGTTCATGTCAATCCTTAATAAGAATTTCTGTCTTCAAATGTCTTCCAGTAACTGTTAAACCGCCGTTTCGTCGTCTCCTCTTGCTGCGGTAAACCGATATGACGTCAGTGCAAAGTTACCGTCTCTTTTTGCTAATCCGCAATAATCAGGGCGACGGCTGGGCTCCCGAAATCACCGGCCCCTGGATTCGCGCTCCTGGCACGCGCGCCGCGCCTTATAAATAGGGCTTGAAGGCCATCTTCCTTTCCTCCCTTCCGTCCCCTTCTTCGTCTTCCTCGCGACTCAGGCTTCGGAGCTCCGCCGCCGCCGTCAGCTACTGCATCAACTCAGGCCGCTGCATCAACTTGACCGGACCAGAGTACCGCGGCGACCCTCCACATCCGTTTGGATCCCGGTAAGGCTCCTGTTCTTCTTGCTTTAGATCTGTTCTAGGGTTCGTCATCCTGCCACTGTTCCTTGTTCATCTCCATAGCAAGCTTTTGTGAACAGATTATGATACTTATATTGTGATTTGTAGTCACTCTCAGTATTCGCCATAAATCTGTACTCATCTCCTTAGATCCCACTCATTTCCCTGCTTGTCGAACTTATGCTGTTCTGCTGCGAGCCAAACCTCATATTTTTACTTCTTTTTTGCCCTAGCTGTAGATCCATTGTTTTGAAAGAATCCGTACGGGATCTGTTTCTGCTTTCATATTCACAACTGTTAAATCTGGTCGGTTTAACTTTTAGCAGAGCGAACAACCCAAACTATATATCCATTAGTCCCCTTGATGAACCGCCAGATGATATCATTGTAGATAACTCCATTCTATATAAGAGCATCTTCGGTTTACAATCACACCTGTTCTCAAACCGGAATACCTTTGTCTTGTAGATTTCCTCATGGCAAAACAGGTATACGAATGCAACTGGGTTCCCTCTCGCATCACCGAGACCCAATTAAACAACTTAGTTCTGACTGGCGCCTTGAGTAGCAAAAATGCCATCCATTGGAGGGTTCCAGGGAATGAATGTCCTCCTACCCCACAGGAAGGAGAAGTGGTGGTGTTTGCGGATCACCTGGCCCGGGGCTTTAATCCACCCGGTTCAAAATTTTATCGGGACGTCCTGGCTAATTTTAAGTTGCGCCCACAAGATGTTGGTCCAAACTCAGTCATGAATATCTGCCATTTTCAAGTGCTTTGTGAGGTGTACTTCCAGGAGGAACCTACTGTAGAGCTGTTCCGAGACTGTTTTCATTTGAACCGACGTACTGAATTCACCGACGGCCCTAATATGGAACTGGGTGGTATGGCGATTCAAAAGAGGAAAGAAGTGACTTATCCTCACGCCAAGCTGCATAGCCATCCGCAAGAATGGAATCACACTTGGTTCTATTGCAAGGATACTTCCCCTGCTGAAGAAAATCCTCTGCCTGGCTTCCGTCCAGAGAGACTTAGCAATACACACCCCTTCCCCCCAAGGCTAACCGCCAAAGAGAGGAGTAAGTATGCCCCTCAGCTGTCCAAGCTCAGAGCCTTCATGGCTAACGGTTTAACGGGGGTAGATCTTGCCCGTTGCTGGATTTCATGGAGCATCCTGCCCCTTAGTATCCACTCCGGTTTAATGTGCCAATACACGGGCCGGGTTAATGATCCTTTGAGGCACTGCAACATTCAGCTGACGGAAGACGAAGTTACTGAAGCTGTGAAAAAGATGCTGAATGAACCGGAATATGTTTGCGCCCGAACCGGCCTGCTTCCCTTTTGTGCTACAAATAAACCACCTGCTGTAAGCATCTCACCTCTGTTTGTTATAGAATTTATGATCTCCTTATATGCACACTGTTAACTCCAGTCCTTTCTGAGACAGGGAGATGATCCGTTTTGGAGCAAGAAGCTTCAAGAGCCGACAGAAAAGGTAACCCGACCCAAAACAAGGGTGAAGAAGCCCGCTCAGAAGAAAAGGAACACCGCTTCCTCTACCCCGGATTTAGATGAGGATGATGTGGGTAATCCGGACCTTGAGGTAGACCTCGATTCAGTTGGCTTACTTTTTATACGTCTTATTGATGATGATATTTGTCAGGACGACGCTGAAGCGAGTAATGCTAAAGGCCTAGAGGTAACTATTCTTTCTTCTGATACAGTTCCTTTGCCTTTGAATAAACCTCGCCAAGCAAACCGGAAAGTTAAATTTTCTCATCCTCTTGCTTACTTGGATCCCAAATTTCTTGTGAAGACCCAGCAACATGAAGCTCGTCGCACCACCCGGCATAGTGGCCAGGTAGTTACCTCCGCCGGTTTACCGAATAGCCCGGTTCGGAAACGCCGCTCAGAGGTCTCCGATTTATGCGCTAGCTTTCCTCCTAAAGCAGGTTTAATTCGCCAGCCTCTTAATCCATCTGACTCCGATTATCAGGTCACTTCCCATTCATCTTCTGGTGAATCTTCAGCCACTCAACTGCCACCGCTAAAGACAGTTATTGGGTAAGTTGGACCGATCACAACATATTTCCATATGGTTTTCTTAATCCATCTTACACCGTTCTTTCAATCTTTGGGTCTTTCCCAGGGCTAAACCTAGACCGAGCAAGAAGGCTCGTCCGGATAATCCGGCCGAAGGAACTGCCGCTCCTGAACCGGAGAGAACTCCTGGTGCTGATGTTCTAGTTCGTGAAGACATTCAGAATTATCTACCTCCACAAGATGACGCCTATGCTGAAGAAAGGCCTACCAATCCTGCCAGCCATACCGATCAGCCTGCCAGTCCTGTTCGGGTTGAGGAATCCATCCCCCCTTCTGGAACTGTTGATAAACCGACAGTGCCAATGCAGAGCAGCGATTCCAAAGATGATGATGTTGTGATTACCGGCATTGGTCGCTCTGAACCGGGGAACTCTGCTACTTTAGCCAAGCATACTGCCAAGGAAGAAATCTCGGCCATAAAGGGCAAATGGGACGTTGATTCCTCAACGTACGCCGCTTTAAGCGCCCAAGATCTTTATTCTGGGTACCTGAACCGACTGTACACCAGTCGCGATTATGAAGCTGGTATGATCAGGATGATGAAAGAAAAACTTGAGGTAACTCTCTTGTGCTTCATTCTGTTGCAACTTAGCTTCTCAATTTTTTCCAATCCGAGATGGTAATCCGAATCATGCTGCCTTGACAACTAAGGCCCGTTTTAAAAACCCAGGGCTTCAACATAGGCCGTTAACCAGAAAACATCATCAAATAACCATTAGCCCCAAGTACCAAGTTTAAATACTTGTATTAATCCTGGGACTTTTGTAATGTAAAACATGCGTTTAAGATCAAGTACGCATTAGCCCCCAAGTATCGAGCACATAACTTGCTTGTGTGGTTGATACTTCCAAACCGTGCAATATTTTCTGAACACTGCTGACGACCTGCTGCAGGGGGACCTGAAGACCAAAGAGAAACAAGTCTCGGACCTTCAGGAAATCCTAAAAACCCAGCAGGCTGAAACTGCCAAAGCGAAGGAAGAATTGACCAATGCCTTAACCGTTATGGAACAGCTGAAAGAAACCCGCAAAAAGGAACAAGCTGATTGGGCTACTGAAAAAGCTCTTTTGATTAAAAGGGCTGAAAATGCCGAAGCTGCTCTCAAACCGGTGGTTGATGAATTATCCACCGTAAAGCGACAAATACACTCCATGACCTCTGCCGTCTTTGGTAAATTTCTTTTGATGCCCTTCCTCCAATTTGCACTTTGTCTGCTATATCCTATGTCCGGTTTGTAATCCATCTTGAAACACTGCAGGCACACGTATTGGACACTTGGGCTCTGATGAGCGGATGAAACTCAAAGCTGCTTATACTTTGATTGAACAGTTGTATACCGGAGCACAGAGAGCTATCACCACTGCATCGCATAACAACCCCGCACCGTCTCTGATTCAAGACACGTTAAAAAGGTTATCCATGCTTCCTGCCCGGATTGAAGAGTTAAAAAGATCAGCTGCTCAAACCGGAGCCATCACTGCCTTGATCCGGGCCAAAGCGTGGGTACCAGACTTTGATCCTGTCGAAGTGGCACAAGGATATCCCAGCTTGAAAGAAGACGGTTCAGACTTCGGTGAAAATGACTTGAGGGCGATAAACCGGGCGGTGCGCCCCCTAGCTTGTCAATTGGCTGAAGAAGCAGATCTGACACATTATCAGGCCCAATATGATAACCAGAACAAGCGAGTAGCTGCCCCGATCCCTGAAGCGGAAAACCTTATTCCTCCAATCCGTAAGCATACCTACGCCCCTGACACTGAACCGTCTTTGCTGATTCATGAAGAAGCTGTCTTTCAAGCGTTAATGGGAATCGACTGGACCACTGCTGATTTCTAGCCAATGGGTAGCCATGAGAAGGCTGAAGCGGCGCAAGATGATCCACAACCTTCGAACCGAGGCGGCGAACAGGCGTAATCAAAGCTCCGGTTCACCTCAATTGGTGATGTTAATCCGTCAAGAAACAATATATTCAGGCACTACGCTGCCTTGTAATAAGATAGTTAATACCTCTGTGTTTGAATGCGCCTTTGTGCACGGGTACTGATGCCCTTGCCATTGACTTTTTCCAACCCGTGTTTCCTGTCTTTACTAGGTACAAAGGACGGTCTTTCGACCATACGAACCGTGCTGCGCCTTTGCCAAACCGGGCAACTTGCCTCCGGGTTAAAAAACGGACCAGGCTGTGTCAGCTGAATCGGTTTAAAAGTATTTGTCATTCCGAAGAACAAAAACACCACAAGAAGGAAGATAACAAAAAACCAAATACGGGAGGAAAACGCAATAGTAAAACGCATGCAAATGGTGGTTGCTATGAACCGTTCAGAAGGCGTTACCATTGTCAAGACAAAAAAAACCAAGCCCCCAAGAGGTGTAGCTACGGTTTAAGTCGACTAGGTTCCTAACTGAATCGTGGCAAATATGCCGGTCAAATGGCATTGCATTGGTTCGGATTCGACCAAGCCCCCAAGTGACTCAGTGGCCTAGGGCCGATCAAGTGGCATTGCGTTGGTTCGGACACGACCAAGCCCCCAAATGACTTATTGGCTGTTTAAAAGCCGATCAGAAGGCATTACCATCATCAAGACATGACCGAGCTCCCATAAGGTGTAACTACGGTTCAAATCGATTAGGTCCCCAACTGACTTAGTGGCAAATGTGCCAGCCAGAAGGCATTACCGTCGTCAAGACATGACCAAGCTCCCCGGAGGTGTAGCTACGGTTCAGGTCGACTAGGTCCCCAACTGACTTTGTTGATTTCCTGTGAAGCGATCTCAAGGGGTAAACCGGAGGTTGCCCTAAAGCGAATCAACTCCGTGTAACCACGCATGATGTGAAGAAAAACAGAGACCCCGCTTTACTCGAGGCTCCGGTTCATTATATTGATCATAATATATACATGGTCATGATATGTACATAAACAGAGCCCAAGGCTCATGTATAGTAAGGCCGAAGATGAGCTATGTTCCACGGCCTGCTGGTCTCCTCCTCTGATGTGCGTGAATCTTTGCGCTCTCGAATATCAATCATGTAGTATGACCCATTATTCAAATTCTTGCTGACCACAAAAGGTCCTTCCCAAGGCGGGGATAATTTATGCTGATCAGACTGATCCTGGATGAGCCGAAGCACCAAATCCCCCTCCTGAAAGGTTCTGGTCTTAACCCGACGGCTGTGGTAACGGCGAAGATCTTGCTGGTAAATCGCTGAACGGGCAACTGCTAATTCACGTTCCTCATCCAACAAGTCAAGAGCGTCCTGCCGTGCCTGCTCGTTGCCCGCTTCAACATAAGCTGCCACACGAGGTGAATCATGTCGTATATCACTGGGGAGAACCGCCTCTACTCCATAGACCATAAAGAACGGCGTAAACCCCGTGGATCTGTTAGGCGTGGTGTTGATACTCCATAATACAGACGGTAGCTCCTCAACCCAACAACCCGGCGTCCTTTGCAAAGGGACCAGAAGCCGGGGTTTGATACCTCTCAAGATCTCTTGATTCGCTCGTTCTGCTTGACCATTAGATTGTGGGTGAGCTACTGAAGAAACATCAAGCCGTATATGCTCACGTGCGCAGAACTCTTTCATGGCACCTTTGGATAGATTGGTACCATTATCTGTCATGATGCTGTGTGGAAAGCCAAACCGGAAGATCACCTTCTTTATAAACCGAACCGCCGTGGCCGCATCACATTTACTGACAGGTTCTGCCTCCACCCACTTTGTAAATTTGTCAACCGCCACCAATAGGTGGGTCTTCTTATCTTTGGAACGTTTAAAAGGTCTAACCATATCGAGCCCCCAAGCCGCAAACGGCCAAGTAATTGGAATCATCCTCAATTCTTGAGCCGGTATGTGCGCTCGCCTGGAAAATTTCTGGCAATTATCACATAACCTGACCAGGTCTTCTGCATCAGCATGAGCAGTTAACCAGTAGAAACCGTGGCGGAACGCTTTAGCCACCAATGATTTTGAACCGGCGTGGTGGCCGCAATCCCCTTCATGTATTTCACGTAATATCTCGCACCCCTCCTCAGGGGATACACACCGTTGCAGCGCTCCTGACACGCTGCGATGATGTAGCTCCCCCTTGAGAATGGTCATAGACTTGGACCGCCGTACTATCTGCCGAGCCAAGGTTTCGTCTTCTGGCAACTCACCCCGGTTCATATATGCCAGATAAGGAAGCGTCCAATCCGGGGTGACGTGAAGAGCCGCCACTAATTGAGCCTCCGGATCAGGAATAGCCAACTCCAGCTCACCGGGTAGCTGTACCGATGGGTGATACAATACATCCAAGAAAACATTGGGTGGGACCGGTTTACGCTGAGAGCCCAAGCGGCTTAAAGCGTCCGCTGCCTCATTCTTTCTGCGGTCCACATGGTCCACTTGATAGCCTTTGAAATACCCAGCCACCATATCTACTTCACGACGGTATGCAGCCATGAGCGGGTCCTTAGAATCCCAGGTGCCAGATACCTGCTGAGCCACTAGATCCGAATCACCAAAGCACCTAACTCGGCTCAGATTCATCTCTTTAGCCATCCGAAGACCATGAAGCAAAGCTTCATACTCAGCCGCATTATTTGTACAGGGGAACATTAAACGGAGGACATAACAAAACTTATCTCCTCGTGGGGAAGTTAAAACGACTCCAGCCCCCGAGCCTTCCAATTGTCTGGATCCGTCAAAATGAATAGTCCAATATGTGTTATCTGGCTTCTCCTCAGGTGCTTGTAACTCTGTCCAATCGTTTATGAAGTAGACAAGTGCTTGTGACTTTATGGCCGTCCGGGGTACGTATCTTAAACCGTGCGGCCCAAGCTCGATAGCCCACTTGGCAATCCGACCGGTCGCCTCCCGGTTCTGTATGATGTCACCCAAAGGAGCAGAACTGACCACCATGATGGGGTGTCCTTGAAAATATTGCCTCAGCTTCCGGCTTGCCATAAAAACACCATATACCAGCTTCTGCCAATGCGGATACCTTTGTTTGGACTCGATAAGCACTTCGCTGATATAGTAAACCGGTCGCTGAACCGGATGCTCCTTCCCAGCCTCCTTGCGTTCTACCACCATCGCCACACTGACCGCTCTGGCGTTCGCAGCAACATATAACAGGAGTGGCTCTTTATCTACAGGGGCTGCCAACACTGGCGGATTGACCAACTGCCGCTTCAAGTCCTCAAACGCTTTATCAGCCTCCGGACTCCAGACAAATTGATCTGTCTTCTTGAGCATTTGATACAAGGGGATCGCCTTTTCACCGAGGCGGCTGATAAACCGGCTTAATGCTGCAATCCGTCCGGCCATGCGCTGAACATCATTGACGCATTTCGGTTTGGCCAGGGAGGTGATGGCTGTGATCTTTTCCGGATTGGCCTCAATTCCTCTATGGGACACTAAGAATCCCAACAACTTGCCTGCCGGTACACCAAAAACACATTTAGCTGGGTTGAGCATCATTTGGTACACTCTTAAGTTATCGAAGGTTTCTTTCAAATCATCAACCAGGGTCTCCTTCTTTCGTGACTTGACCACAATATCATCCACATAAGCATGTACATTACGACCAATCTGTTCGTGGAGGCAATTTTGCACACACCGTTGATAAGTAGCCTGCGCACTCTTAAGCCCGAAGGGCATAGAAACATAGCAGAAGGCTCCAAAGGGAGTAATGAATGCCGTCTTCTCCTGGTCCTTAACTGCCATCTTGATCTGATGATAACCCGAATAGGCATCCAAAAAACTCAAACGCTCGCAACCTGCCGTAGCATCAATGATTTGATCAATACGGGGGAGAGCAAAAGGATCCGCTGGACAGGCTTTATTCAAGTCTGTGTAATCCACACACATGCGCCAGGTGCCATTTTTCTTAAGAACCAGCACCGGATTAGCAAGCCACTCGGGATGAAAAACCTCAATAATGAAACCAGCTGCCAGGAGCCTGGCTACTTCCTCACCAATCGCTTTACGTCTTTCTTCGTTAAACCGGCGTAGGAACTGTTTTACCGGTTTATACTTAGGATCCACATTAAGGGTGTGCTCAGTGAGTCGCCTCGGTACACCAGGCATGTCAGACGGCTTCCATGCAAAGATGTCCCGATTCTCACGGATGAACTCGATGAGCGCGCTTTCCTATTTTGGATCCAAGTTTGTACTGATGCTGAACTCCTTGGACGAATCGCCAGGTACGAAGTCAACAAGCTTAGTTTCGGCAGCTGATTTGAACTTCAAGGCCGGATCGTGGTCCGTAGTTGGCTTCTTTAATGAAGTCATGTCCTCCGGATCGACATTGTCTTTGTACTGCTTTAACTCCTCCGTAGCACAAACCGATTCTGCATAAGTCGCATCTCCTTCCTCGCATTCCAAAGCGATTCGACGGCTCCCATGAACCGTTATAGTGCCCTTGTAACCCGGCATCTTGAGTTGCAGATATACATAGCAGGGCCGTGCCATAAATTTTGCATAGGCCGGTCGTCCGAACAAAGCGTGGTATGGACTTTGGATTTTAACCACTTCAAACGTCAGCGTCTCTGATCTGGAATCATGACTGTCGCCGAAGACCACTTCAAGGGCTATCTTACCAACAGGGTACGCAGACTTGCCAGGCACCACCCCGTGGAACACTGTATTGGTCGGTTTGAGATCCTTATCCACCAGTCCCATGCGACGGAAGGTCTCATAATACAAGATGTTAATGCTGTTCCCTCCGTCCATGAGTACCTTGGTGAGCTTATAACCTCCCACCTGAGGCGCCACCACCAATGCTAACTGACCCGGATTATAAACCCTGGGAGGGTGATCTTCTCGGCTCCATATGATTGGTTGTTCCGACCAGCGCAGATAGTAAGGCGTGGCCGGTTCAACAGCACTAACTGCCTGCCTCTGAAGCTTTCTATCCCGTCTATCCAAGCTTGTAGTAAAAACATGATACTGTCCACCACTCAACTGTTTCGGGTTGCTCTGATAACCTGATTGCTGCTGTTGCTGCTGGCTGTAACCACTTTGATTACCTTGATTATTTTGGCTGTTCTGTCCGCCCGGATTACCATGGAACCCTGGACCGGAATTTCCGCCGCCGTATCCTGATCCGGAACCTGAACCGCCGCCAGAGCTGTGATCATATTGGAAAGTGTTTGAGCTTTTGAATTCCTTCTTGATGTAACAATCCTTCCAAAGATGGGTTGCTGGCTCCTCTTTTGTTCCGTGCTTCGGACAGGGCTGGTTTAAGAAAAAGTTCAAACGCTCCGGATTAGGACCGGGATTTCCGCGAAAACCTGGCTGTTTCCCCTTACGTCGCTGGCCCTTATTCTGTGCACTTGTGTTGGCCACAAAATCCACGCTCCCATCCGGTTTACGCTTACCTCCGCCTCCATTACCTGTTGGTCGATGCTGCTGGCCTTTGGTGTTGCTATTCTTCTTTCCCTTCCCTGCTTTGTCGTCACCAGAGTCAGGATCCTTGGTACTGTCAGAATCCACATACTTCACTAAAGCGGTCATGAGCGTTCCCATATCCGTGCAATCACGCTTCATCCGGCCTAACTTTAGCTTCAAAGGGCCGAACCGACAGTTGCCTTCCAACAGCACCACGGCGGTGTCTGCGTTGATGCGATCTGATGAGTGCAAAACTTCTGATACCCGGCGTACCCAATGGGTAGTTGACTCCCCTTCTTGCTGAACGCAAGCTGCTAGGTCAACTATCGACATTGGCTGTTTGCACGTGTCCTTGAAATTTTTGATGAACCGGGCTCGCAACTGGGCCCACGAATTAATGGAATTAGGCGGTAGGCTCTTTAGCCAAGTCCGGGCCGTTCCTTCTAACATCATGGTAAAATACTTCGCACACGCTGTGTCATCCACATCCAGCATCTCCATAGCCATCTCATAGCTCTCCACCCATGTCTCCGGAGGCTGATCTGCCGTGTAGTTTGGTACCTTGCGGGGGCCTTTGAAGTCTTTAGGCAGGCGTACGTTGCGCAAAGCGGGAACAAGGCACGGTACTCCCAAAGAACTAGAAACCACCCCCGGTTCAATCGTAGCAGTTGGGAAGATAGGTGTAAGCTGACGAGCCTGATGCTGCGCTGCTAATCCGGCCTCCTGCGCACGGGCGCGATCCACATTCTCCTGAGCATTGTCGACGCCCGCCGGATTGTTGCCGCGGAGCGCTTCACGCCTGGCATTACTTGAAACAGCTGTCTCCTCCATGCGCCTACTATAGCTCCTGCTGGGGCGAGGGGTGGAGTGAATCCGATCATGGCTTTGCGAATATGCTTCTTGTTGGGCCAAAGCGGTCCTAAGGAGCTCCTTGACCCGGCGTGTCTCTACATCCTGCGGTGAATCGCCTTCGAACGGAATGGCTTCCAACCGAGCTGCCGCCGCGACGAGGTTATCCATCGGGTTGGAATAATGGCCCGATGGTGTTGGTACGGCCTGTGGTGTACCAGTGTTGTGACGAGGCAGGTTTATTATACGGTGCTGAACCGGGGCGCCAACTGTCGCTTCCGCACGGTTTACCCCCGGGGTGTTGAAGAGATTTCTGGCATCGAGGGCTGCGGGCAATCGAGATCGGAATTTCCTCTTCAGGACTTCCTGAGACGCATTCTGGTCCATCATGAACCTGTAAGCCTGTGCATCTAAAGCGGCGCGCTCTGCCGTCATCCTGGCCTCTTCTGCTGCCAGCTCCGCTTTAGCTTGAGTGATCTGTTCTCTCACTTTGGCAATCTTGGCATTGTGAATCTCCTGATCCACCGGGTTGGCTTCCGCCATGAGCGCTGCCAGGGCGTCAAATAGGTCTGATAATACTTGGGCTGGTGAGTCCGCAGGGCCCCCTGCCCGCGCAGCTGTTGCCGCTGCCGATCCGGAGATCACTGCTGCCGCAGTTGATGAATGTGGCGCCGCCTGTGTCCCAGCCATGAAGATCCCAACCCGATTGGGCAGACCAGAGGGGTCCGGAATACTGTCGCCATCGGAGCTGCCCCCAATCCGATCATCTTGCAGCTGGTATAGAGACTCGGTTTCCCCGGTTGACGTTTCATCGCCGGAGTAGATGGCGGTTTCGTCACCGGATTCGGATCCTTCCTCGTAGCTTCCTCCGTGGATGACTCCCACGAAGGCGCGCTTCCTGGTCGGTTTAGCCAGGGTCGATCTCGTGCACTGAGCTGTCTCGACGAGGTCGGTGCAGACGCCCAGCTCGGGGCCCGGTTCGCCGATCTTGCCAATGAAAACATGGATGCCGCCAAAGGGGACCCGGTACCCGTACTCGATCGAGCCGGCGTCGGGGCCCCAGCCTGTGCTGTCGATGTAGAGTTTTCCGCGACGACTTTTGGTCATCCGGCCCACAGCGTAGCCCTCGAGTCCTTCAAAGCGGCCCTCCAAGAACTTGAAACCATCTCGCGATAGCCCCATGGTGGGCGCCAAC

TGTCGTGGATTTGTCACGGCAGATGTCCTAGCAAAAGGACTTAGTCGTGGAGCCATCGCAACGGGTTAACTTGAAGGGGTTAAAACGGACGCAAGGACACAGAGTTATACTAGTTCGGCCCCTTCGATGAAGGTAAGAGCCTACGTCTAGTTGTGATGGGATTGATGTGTTTCGAGGGCTAGGGAGCGATAAGCTTCGCCTAAACCTCGAGTTGTTGTTCTCTGTCTTTAAACCGCCGTCGGGTCGTCCCCTTATATACACGGGTGACGCCCGTCGGTCTACAAAGTCCCAACCTCCGGTTCATAAACGTGCCCGGGTTGGTCTCTCTATTCCTAACTTACAATGCAAGTTTACCTCAGGCCGGTTTACGGCCATAAACCTTTGAACCGATTATAGGCCTTGGGCCCTTCTATTTCTCCTTGGGCTTTAACATCTTGGGTCCATTGACGGAGCTAACCCAGGCCCTGTAGGCCGGTTTACCCCAAATAGCAATATCCCCAACA

GCACCCGAAGTTGGCCACTTCAGCCCGGACATCCGGCCCGAGCCCCGAACATCTGGCGCCTCCTGAAGCCCGGACATCCGGCCCCCTGCCCGGACATCCGGCACCTGCGCGTGCACAGCCGGGC

>Aegilops umbellulata 1-141;

TCCCATGCTTTATGCAAATCAAATCAAAATAATTGCAAACAAAACTCCCCCGGGACTCTTGTTAGTTGGGGGCACTCGTTGTTTCGAGCAAGCCATGGATTGATGCTTGTTGGTGGAGGGGGAG

TGTTGGGGATGTTGCTATCCGGGGTAAACCGGCCTGCAGGGCCCGGGTTACCTCCGTCAGCAGACCCGAGATGTTAAAACCCAAGGAGAGATAGAAGGGCCTAAGGCCCATAATCGGTTTAAAGGTTTATGGTGTAAACCGGCCTGATGTAAACTTGTATTGTAAGTTAGGAATAGAGAGACCGACCCGGGCACGATTATTAGCCGGAGGTTGGGACTTTGCAGACCGACGGGCGTCACCCGTGTATATAAGGGGATGACCTGGCGGCGGTTTGAGGACAGAGAAAGAGAACTCGAGGTTTAGGCGAAGCTTATCGCTCCCTGAGCCCTCGAAACCCATCAATCCCATCACAACTAGACGTAGGCTTTTACCTTCATCGAAGGGGCCGAACTAGTATAAAACCGTGTCCTTGTGTCCACTTTAACCCCTTCAAGCTAACCCGTTGCGATGGCTCCACGACTAAGTCCTCTTACTAGGACATCTGCCGTGACAAACCGACGACA

GTTGGCGCCCACCGTGGGGCTATCGCGAGATGGTTTCAGGTTCTTGGAGGGCCGCTTTGAAGGACTCGAGGGTTACGCTGTGGGACGGATGACCAAGAGCCGCCGCGGAAAACTCTACATCGACAGCGCAGGCTGGGGCCCCGACGCCGGTTCGATCGAGTACGGGTACCGGGTCCCCTTTGGCAGCATCCATGTTTTCATCGGCAAGATCGGAGAACCGGGCCCCGAGTTAGGCGACTGCGCCGACCTCATTGAGACAGCTCAGTGCACGAGATCGACCCTGGCTAAACCGACCAGGAAGCACGCCTTCGTGGGAGTCATCCACGGGGGAAGCTACGAGGAAGGATCCGAATCTGGTGACGAAACCGCCATCTACTCTGGCGATGAAACGTCGACCGGGGAGACCGAATCTCTTTACCAGCTACAAGATGATCGGGTTGGGGGCGGCTCCGATGGCAACAGTATTCCGGACCCCTCTGATCTGCCCTATCGGGTTGGGATCTTCATGGCTGGGACACAGGCGACGCCACGATCATCAACTGCAGCAGCAGCGATCTCCGGATCGGCAGCAGCAACGGCTGCGCGGGCACGAGGCCCTACCGGTTCACCAGCCCAAGTTTTGTCAGACCTGTTTGACGCACTAGCAGCGCTCATGGCGGAGGCCAACCCGGTGGATCAGGAGGTTCACAATGCTGAGATTGCCAAAGTGAGAGAGCAGATCACTCGGGCTAAAGCGGAGCTGGCAGCAGAAGAGATCAGGATGACGGCGGAGCGCGCCGCTTTAGAGGCACAGGCTTACAGGCTCATGATGGACCAGAAGGCATCTCAGGAGGTCCTGAAGAGAAAATCCCGGTCTCGGTAGCCCGCCGCCCTCGACGCTCGAAATCTCTTCAACACCCCGGGGGTAAACCGTGCGGAGGCGACAGCCGGCGCCCCGGTTCAGCACCGGATTATAGACCTGCCTCGTCATAACACTGATACGCCACCGGCCATGCCAACGCCGTCAGGTCATTATTCCAACCCGATGGATAACCTCGTCGCGGCGGCGGCTCGGTTGGAAGCCATTCCGATCGAAGGAGATTCACCGCAGGATGAAGAGACACGCCGGGTCAAGGAGCTCCTTAGGACCGCGTTGGCCCAGCAAGAAGCGTATTCGCAAAGCCGTGATCGGATTCACTCCACCCCTCGCCCCAGCAGGAGTTATAGTAGGCGCGTGGAGGAGCCGGCCGTTTCAAGTAACGCCAGGCGTGAAGCGCTCCGTGCCAACAATCCGGCGAGCGTCGACAATACTCACGAGAATGTGGATCGCACCCGTGCACGGGAGGCCGGATTAGCAGCGCAGCATCAGGCTCGTCAGCTTACACCTATCTTCCCCGCAGCTACGGTTGAACCGGGGGTGGTTTCCAGTTCTTTGGGAGTACCGTGCCTTGTTCCCGCTTTACGCAACGTACGCCTGCCCAAAGACTTCAAAGGCCCCCGCAAGGTACCAAACTACACGGCAGATCAGCCTCCGGAGACATGGGTGGAGAGCTATGAAATGGCTATGGAGATGCTGGATGTGGATGACACGGCGTGTGCTAAGTATTTCACTATGATGTTAGAAGGAACGGCCCGGACTTGGCTAAAAAGCTTACCGCCTAATTCAATTAGTTCATGGGCCCAATTGCGCGCCCGGTTTATCAAGAATTTCAAGGACACGTGCAAACAGCCAATGTCAATAGTTGACCTAGCAGCCTGCGTTCAGCAAGAAGGGGAATCAACTACCCATTGGGTACGGCGGGTATCAGAAGTTTTGCACTCGTCAGATCGCATCAACGCAGACACCGCCGTGGTGCTGTTGGAGAGCAACTGTCGGTTTGGCCCTTTGAAGCTAAAGCTGGGCCGGATGAAGCGTGATTGTACAGATATAGGAACGCTCATGACCTCTTTAGTGAAGTATGCGGATTCTGACAGTACCAAGGATCCTGACTCTGGTGATGACAAAGCAGGGAAGGGAAGGAAGAATAGCAACACCAAAGGCCAGCAGCATCGGCCGACAGGTAATGGAGGCGGAGGAAAGCGTAAACCGGACGGGAGCATGGACTTGGTGGCCAGCACAAGTGCACAGGATAAAGGCCAGCGGCGTAAGGGGAAACAGCCATGTTTCCGCGCACATCCCGGTCCTAATCCGGAGCGTTTGAACTTTTTCTTAAACCAGCCATGTCCGAAGCACGGAACAAAGGAGGAACCAGCAGCCCATCTTTGGAAGGATTGTTATATCATGAAGGAGTTCAAAAGCTCAAACACTTTCCAATATGATCACAGCTCCGGTGGCGGTTCCGGTTCCGGATCAGGGTACGGTGGCGGGAATTCCGGTCCAGGGTTCCAGGGTAACCCGGGCGGACAGGTCAGCCAAAATAATCAGAATAATCAAAGTAATCAAAGTGGTTATCAGAGCAACCCGAAACAGTTGAGTGGTGGACAGTATCATGTCTTTACTACGAGCTTGGATAAACGAGATAGGAAGCTTCAGAGGCGGGCAGTCAGTGCTGTCGAACCGGCCACGCCTCACTATCTGCGTTGGTCGGAACAGCCTATCATATGGAGCCGAGAAGATCATCCTCCCAGGGTGTATAATCCGGGTCAGTTAGCACTGGTGGTAGCGCCTCAGGTGGGAGGTTATAAGCTCACCAAGGTGCTCATGGACGGAGGGAGCAGCATTAACATCTTGTATTATGAGACCTTCCGTCGTATGGGACTGGTAGATAAGGATCTCAAACCGACCAATACAGTGTTCCACGGGGTGGTGCCTGGCAAGTCTGCGTATCCGGTTGGTAAGATAGCCCTTGAAGTGGTATTTGGCGATAGTCACGATTCCAGACCAGAGACGCTGACGTTCGAGGTGGTTAAAATCCAAAGTCCGTACCACGCTTTGTTCGGGCGGCCAGCTTATGCAAAATTTATGGCACGGCCCTGCTACGTATATCTGCAGCTTAAGATGCCAGGTTACAAGGGCACTATAACGGTTCATGGGAGCCGCCGAATCGCTTTGGAATGCGAGGAAGGAGATGCGACTTATGCTGAATCGGTTTGTGCTACGGAGGAGCTAAAGCAGTACAAAGACAGTGTTGATCCGGAGGATATGACTTCATTAAAAAAGCCAACTACGGACCACAATCCGGCCTTGAAGTTCAAGTCAGTGGCCGAAACTAAGCTTGTTGACTTCGTACCTGGCGATTCATCTAAGCAGTTCAGCATCAGTGCAAACTTGGATCCAAAATAGGAAAGCGCGCTCATCGAGTTCATCCGTGAGAATCGGGACATCTTTGCATGGAAGCCATCTGACATGCCTGGTGTACCGAGGCAACTCGCTGAGCACACCCTTAATGTAGATCCTAAGTACAAGCCGGTAAAGCAGTTCTTACGGCGGTTTAACGAAGAAAGACGTAAAGCGATTGGTGAGGAAGTGGCCAGGCTCCTGGCAGCTGGGTTTATCATTGAGGTTTTTCATCCCGAATGGCTCGCTAATCCGGTGCTAGTTCTTAAGAAAAACGGCACCTGGCGCATGTGTGTGGACTACACAGACTTGAATAAAGCTTGCCCAGCAGATCCTTTTGCTCTCCCCCGGATTGATCAGATCATTGATGCTACGGCAGGTTGCGAGCGTTTAAGTTTTTTGGATGCTTATTCGGGTTATCATCAGATCAAGATGGCAGTTAAGGACCAGGAGAAGACAGCGTTTATTACTCCCTTTGGAGCCTTCTGCTATGTGTCTATGCCCTTTGGGCTTAAGAGTGCGCAGGCTACTTATCAACGATGTGTGCAAAATTGCCTCCACGAGCAGATTGGTCGAAATGTGCATGCTTATGTGGATGATATTGTGGTCAAGTCACGAAAGAAGGAGACCCTGGTTGACGATTTGAAGGAGACTTTCGATAACTTGAGAGTGTACCAAATGATGCTCAACCCGGCTAAATGTGTTTTTGGTGTACCTGCAGGTAAGCTGTTGGGATTTTTAGTGTCCCATAGAGGGATTGAGGCCAATCCAAAAAAGATCACAGCCATCACCTCCCTGGCCAAACCGAAGTGCATCAATGATGTTCAGCGCATGGCCGGGCGGATTGCAGCGTTAAGCCGGTTTATCAGTCGCCTTGGTGAAAAGGCGATCCCTTTGTATCAAATGCTCAAGAAGACGGATCAGTTTGTCTGGAGTCCGGAGGCTGATAAAGCGTTTGAGGACTTGAAGCGACAACTAGTCAATCCGCCAGTGTTGGCAGCCCCTGTAGATAAAGAGCCACTCCTGTTATATGTTGCAGCGAATGCCAGAGCAGTCAGTGTGGCGATGGTGGTAGAACGAAAGGAGGCTGGAAAGGAACATCCGGTTCAGCGACCGGTTTACTATATCAGTGAAGTACTTATCGAGTCCAAACAAAGGTATCCGCATTGGCAGAAGCTGGTATATGGCGTTTTTATGGCAAGCCGGAAGCTGAGGCAATATTTCCAAGGACACCCAATCACGGTGGTCAGTTCTGCTCCTTTGGGTGACATTATACAGAACCGGGAGGCGACCGGTCGGATTGCCAAGTGGTCTATCGAGCTCGGGCCGCACGATTTAAGGTACGTACCCCGAACGGCCATAAAGTCACAGGCACTTGTCGATTTCATAAACGATTGGACAGAGTTACAAGCGCCTAAGGAGAAGCCAGATAAGACCTATTGGACCGTTCATTTTGACGGATCAAGACAATTGGAAGGCTCGGGGGCTGGAGTCGTTTTAACTTCCCCACGAGGAGATAAGTTTTGTTATGTCCTCCGTTTAATGTTCCCCTGTACAAATAATGCGGCGGAGTATGAAGCTTTGATTCATGGTCTTCGGGTGGCTAAAGAGATGAATCTGAGCAGAGTTAGGTGCTTTGGTGATTAGGATCTGGTGGCTCAGCAGGTATCTGGCACCTGGGATTCTAAGGATCCGCTTATGGCTGCATACCGACGTGAAGTAGATATGGTGGCTGGGCATTTCAAAGGCTATCAAGTGGATCATGTGGACCGCAGAAAGAATGAGGCAGCTGACGCTTTAAGTCGCTTGGGCTCTCAGCGTAAACCGGTCCCACCCAATGTTTTCTTGGATGTATTGTACCACCCATCGGTTCAGCTGCCCGGTGAGCTGGAGTTGGCTGTTCCTGATCCGGAGGCTCAGTTAGTGGCGGCTCTTCACGCCACCCCGGATTGGACGCTCCCTTATCTGGCATATATGAACCGGGGTGAGTTGCCAGAAGATGAAAGTTTGGCTCGACAGATAGTACGACGGTCCAAGTCTATGACCATTTTCCAAGGAGAGTTGCATCATCGCAGCGTGTCAGGAGCACTGCAACGGTGCATATCCCCTAAGGAGGGGTGCGAGATATTACGAGAAATACATGAAGGGGATTGCGGCCACCACGCCGGTTCAAAATCATTGGTGGCTAAAGCGTTCTGCCACGGTTTCTACTGGTTAACTGCTCATGCTGATGCAGAAGACCTGGTCAGGTTATGTGATGGGTGCCAGAAATTTTCCAGACGAGCACACATACCGGCTCAAGAATTGAGGATGATTCCAATCACTTGGCCGTTTGCGACTTGGGGGCTTGATATGGTTGGACCTTTTAAGCGTTCCAAAGATAAGAAGACCCACCTACTGGTGGCGGTTGACAAATTTACCAAGTGGGTGGAGGCAGAACCTGTCAGTAAGTGTGATGCTGCCACGGCGGTTCAATTTATAAAGAAGGTGATCTTCCGGTTTGGTTTTCCACACAGCATCATCACAGATAATGGTACCAATCTATCCAAAGGGGCCATGAAAGAGTTCTGTGCACGGGAGCATATACGGCTTGATGTTTCTTCGGTAGAGCACCCACAGTCTAATGGTCAGGCGGAACGAGCGAACCAAGAGATCTTGAGAGGTATCAAACCCCGGCTTCTGGTCCCTTTGCAAAGGACGCCGGGTTGTTGGGTGGAGGAACTACCGTCGGTGTTATGGAGCATCAACACCACGCCTAACAGATCCACGGGGTTTACGCCGTTTTTTATGGTCTATGGAGCAGAGGCGGTTCTCCCAAGTGATATACGACATGACTCGCCTCGTGTGGCAGCATATGTTGAAGCGGACAATGAGCAGGCACGGCAGGACGCTCTTGACTTGTTGGATGAGGAGCGCGACTTGGCAGCTGCCCGTTCAGCGATTTACCAGCAAGATCTTCGCCGCTACCACAGCCGTCGGGTTAGGACCAGAACCTTTCAGGAGGGGGATTTGGTGCTTCGGCTCATCCAGGATCAGTCTGATCAGCATAAGTTATCCCCGCCTTGGGAAGGACCCTTTGTGGTCAGCAAGAATCTGAATAATGGGTCATATTACCTGATTGATATTCGAGAGCGCAAGGATTCACGCACAGCGGAGGAGGAGACCAGCAGGCCGTGGAACATAGCTCATCTTCGACCTTATTATACATGAGCCCTGGGCTCTGTTTATGTACATATCATGACCATGTATATATTATGATTGAATACAATAAACCGGAGCCTCGAGTAAAGCGGGGTCTCTTCTGTTCTTCACATCATGTGTACTTACCCCTGGAGGTCGCTTCACAGAAGTTCAAATTCCGGTTTAAAAGCTGGTTCAAGGGAAGATGTCTCCTACAGGGTTGTGAAGCTCTTAATATCCGGTTCAATATCCCGGTTCAAGCATAAGGTTTACTGTTCAAACATAGGCCGTATTCGAGCCAAAGAGAACATAGCCATTCAGCATAAGGCTTACTGTTCAAACATAGGCCGTATTCGAGCCAAAGAGAACATAGCCATTCAGCATGAGGCTTCCTGTTCAAACATAGGTCGTATTCGAACCAAAGAGAACATAGCTATTCAAACATGGGGCTTCCTGTTCAAACATAGGTCGTATTCGAACCAAAGAGAACATAGCTATTCAAACATGGGGATTTCTGTTCAAACATAGGTCGTATTCGGACCAAAGAGAACACAGCTGTTCAAACATAGGTCGTATTCGAACCAAAGAGAACATAGCTATCCCTATAAAGTCAGTTGGGGACCTAGTCGGCCTGAACCGTAGCTACACCTCCGGGGGGCTTGGTCAGGTCTTGACGACGGTAATGCCTTCTGGCTGGCATATTTGCCACTGAATCAGTTGGGGACCTAATCGGCTTGAACCGTAGATTACACCTTATGGGAACTCGGTCATGTCTTGATGATGGTAATGCCTTCTGGTCGGCTTTTTAGCCATTGAGTCACTTGGGGGCTTGGTTGATGAACCAATGCAATGCCATTTGGCCGGCATATTTGCCACGATTCAGTTAGGAACCTAGTCGACTTAAACCGTAGCTACACCTCTTGGGGGCTCGGTTTTGTCTTGACGATGGTCACGCCTTCTGAACGGTTCATAGTAACCACTATTCGCATGCGTTTTACTATCGCGTTGCCTTTTCATATTTGGTTTTGTTTTCTGCCTTTATTGTGGTGTTTTTTGGTTCTTCGGAACGTCAAGTATCCTTAAACCGATTCAGCTGGCATAGCCTGGTCCGTTTTAACCCGGAGGCAAATTGCCCGGTTTGGCAGAGGCGCAGCACGGTTCAGGTGGTCGAAAAAATCACATTGTGTACTCAGTAAAGACAGGAAACATAAGTTGGAAACGTCATTGGAAAGAGCATCAGTACCCGTGCGCGAAGGCACATTCAAAGGCAGAGGTATTAAACTATCTTATTACAAGGCAACGTAGTGCCTGAATATATTGTTCGTTGACGGATTAAGACGTCACCAAGTAGGGTGAACCGGAGCGTTGATTACGCCTGTTCGCCGCCTTGGTTCGAAGGCTGGGGATCGTCTCGCGCCGCTTCAGCTCCATCTTGGTTACCCATTGGCTGGAAATCAGCAGTGGTCCAGTCGATTCCCATTAGCGCTTGAAAGACGGCTTCTTCGTGAATCAGCGAAGACGGTTCAATGTCCGGGGCGTAAGTATGCTTACGGATTGGAGGAACAAGGTTTCCCGCTTCAGGGACCGGGGCAGATACTCGTTTGTTCTGGTTGTCGTATTGGGCTTGGTAACGTGACAGATCTGCCTCCTCAGCCAATTGACAAGCTAGGGGGCGCACCGCCCGGTTTATCGCCCTCAAATCATCTTCACCAAAGTCTGAACCGTCTTCCTTCAAGCTGGGGTATCCTTGGGCCGCTTCGACAGGATCGAAATCTGGTACCCACGCTTTGGCCCGGATCAAGGCAGTGATAGCTCCAGTTCGAGCAGCTGATCTTTTCAACTCTTCAATCCGGGCCGGCAGCATGGACAGCCTCTTTAATGTGTCTTGAATCAGAGCCGGCGCAGGGTTGTTGTGCGATGCAGTGGTGATAGCTCTTTGTGCTCCAGTGTATAACTGTTCAATCAGAGTATAGGCAGCCTTGAGTTTCACCCGCACATCAGAACCCAAGTGTCCAATACGTGAGCCTGCAGTGTTTCAAGATGGATTACAAACCGGACATAGGAGGTAACAGACAAGGTACGAATTGGAAGAAGGGCGTCGAAAAGCTTACCAAAGACAGCAGAGGTCATGGAGTGTATCTGTCGCTTCACGGTGGATAATTCATCAACCACCGGTTTAAGAGCAGCTTCGGCATTTTCAGCCCTCTTCGTCAGAAGAGCTCTCTCAGTAGCCCAGTCAGCTTGTTCCTTTTTGTGGCTCTCTTTCAGCTGTTCCATAACGGTTAAGGCGTTGGCCAATTCATCCTTCGCTTTTTCAGTTTCAGCCTGTTGGGTTCTTAAGATTTCCTGAAGGTCCGAGACTTGGTTCGCTTTGATCCTCAGGTCCCCCTGCAGCAGGTCGTCAACAGTGTTCAGAAAATATTGCACGGTTTGGAAGTATCAACCACACAAGCAGGTTATGTGCTCGATACTTGGGGGCTAATGCATATTTGATTTTAACACAAGTTTCCCATTGCGAAAAGTCCCAAGATTAATACAAGTATTTAAACTTGGCACTTGGGGGCTAATAGTTATCTAATGATGTTTTCGGGTTATGACCTATGTTGAAGCCTTGGGTTTTCTAAACCGGGCTTTAATTGTCAAGACAGCCTGATCCGGTTTACCATTTCGGTTTGGAAACATTGATAATCTAAGTTGCAAGGGAATGAAGCACTAGGGAGTTACCTCAAGTTTCTCCTTCATCATCTTGATCATGCCAGCTTCATAATCCCGACTGGTGTACAGTCGGTTCAGGTACCCGGAATAAAGATCCGGGGCGCTTAAAGCGGCGTACGAAGAAGAGTCAAGGTCCCATTTGCCCTTCATGGCCGAGATTTCGTCCTTGGCAGTATGCTTGGCTAAGGTAGCAGTGTTCCCCGGTTCAGAGTGACCGATGCCGGTAATCACAACATCATCGTCCTTGGAATCGCTGCTCTGCAGTGGCCCTGTCGGTTTATCAGCAGTTCCAGAAGGGGGGATGGATTCCTCAACCCGAACAGGACTGGCAGGCGGATCAGTATGGCTGGCAGGTTCGATAATCCTTTCTTCAGCGCAGGTGTCATCTTGCGGAGATAGATAACTCTGAGTATCTTCACGACCCAGAGCGTCAGCACCAGGAGTTTTCTCCGGTTCAGGGGCGGCAGTTCCTCCAGCCGGGTTATCCAGACGAGCCTTCTTGCTCGGTCTAGGTTTGGCCCTGGAAAAAGCCCAAAGATTGAAAAAACGGTATAAGATGGATTAAGAAACCCATACAGAGATATGCTGTGATCGGTCCAACTTACCCAATAACTGTCTTCAACGGTGGCAGTTGAGTGGCTGAAGATTCACCGGAAGAGGAATGGGAAGTGACCTGATAATCGGAGTCAGATGGATTTAGAGGCTGACGAGTTAATCCTGCTTTAGGAGGAAAGCTAGTGCGTGAATCGGAGACCTCTGAGGGGCGTTTCCGAACCGGGCTGTTCGGTAAACCGGCGGAGGTGACTACCTGGCCGCTATGTCGGGTGGTGCGTCGAGCTTCATGTTGCTGGGTCTTCACAAGAAATTTGGGATCCAAGTAAGCAAGAGGGTGAGAAAATTTAATTCTCCGGTTTGCTTGGCGAGGTTTTTTCAAAGGCAAAGGAACGGCATCAGAAGAAAGGATAGTTACCTCTGGGCCTTCGGCATGGCTCACTTCAGCGTCGTCCTGACAAATATCATCATCAATAAGACGTATAAAAAGCAAACCAACTGAATCGAGGTCTACCCCGAGGTCCGGATTACCCACATCATCCTCCTCTAAATCCGGAGTAGAGGAAGCGGTGTTCCTTTTCCTCTGAGCAGGCTTCTTCACCCTCGTCTTGGGTCGGGTTACCTTTTCGGTTGGCTCTTGTAACTTCTTGCTCCAAAATGGATCATCTCCCTGTCTCAGGAAGGACTGGAGTTAGCAATATGTGTATAAGGGGATCATAAGTTCTATAACAAACAGAGGGGAGATACTTACAGCAGGAGGCTTGTTGGTGGTACAGAATGGAAGCAGGCCGGTTTGGGCACAAACATGTTCCGGTTCATTCAGCATCTTTTTCACAGCTTCAGCGACTTCGTCCTCCGTCAGCTGAATGTTGCAGTGCCTCAAGGGATCATCAACCCGACCCGTGTATTGGCACATTAAACCGGAGCGGATGCTAAGGGGCAGGATGCTCCACGAAATCCAGCAACGAGCAAGATCTACCCCCGTTAAACCGTTAGCCATGAAGGCTCTGAGCTTGGACAGCTGAGGGGCGTACTTACTCCTCTCTTTGGCAGTTAGCCGTGGGGGGAAGGGGTGAGTATTGCTAAGTCTCTCTGGACGGAAACCGGGCAGTGGATTTTCTTCAGCAGGGGAAGTATCCTTGCAATAGAACCAAGTGCAATTCCATTCTTGCGGATGACTATGCAACTTGGCGTGAGGATAAGTCACCTCTTTCCTCTTCTGAATCGCCATACCACCCAGTTCCATATTAGGGCCATCGGTGAATTCAGTACGTCGGTTCAGATGGAAAAAGTCCCGGAACAGCTCTACAGTGGGTTCCTCTTGAAAGTACGCCTCGCAAAGTACTTGGAAATGGCAGATATTCGTGACTGAATTTGGACCAACATCTTGTGGGCGCAGTCTAAAATTAGCCAAGACGTCCCGATAAAATTTTGAACCGGGTGGATTAAACCCCCGGGCCAGGTGATCCGCAAACACCACCACTTCTCCTTCCTGCGGGGTAGGAGGACATTCGTCTCCTGGAACCCTCCAATGGATGGTACTTTTGCTACTCAAAGCACCAGTCAGAACTAAGTTGTTTAGTTGGGTCTCAGTGATGCAAGAGGGAACCCAGTTGCATTCATACACCTGTTTTGCCATGAGGAAATCTACAAGGCAAAGGTATTCCGGTTTGAGAGCAAGTATGATTGTAAACCGAAGATGCTCTTATATAGAGTCAGGTTATCTACAATAATATCAACCGGCGGTTCATCAAGGGGACTAATGGATGTGTAGATCGGTTTGTTCGCTTTGCTAAGAGTTAAACCGACCAGATCTAAACAGTTGTGAGTATGAAAGCAGAAACAGATCCCGTACAGATTCTTTCAAAGCAATGGATCTACAGCTAAGGCGAAAAAGAAATAAAAAACAGGTAATATGAAGTCAGGCTCACAGCAGAACAACATAAGTTCGACAAGCAGGAACAAGAGCTAGATCTAAGGAAATGAGTGCAGATTTATGGCGACTGCTAAGAGGAACTACAAATCCAGATACAGGTAGCATCATCTGTTCGCAAAGGCTGCTATGGGGATGAGCAAGAAACAGTAGCAGAACGATGAACCCTAGAACAGATCTAAAGCAACAAGAACAGGGGCCTTACCGGGGTCCAAACAGATGCGGAAAGTCGCCGCGGTGCTCTGAACCGATCAGGTTGATGCAGCGGCCGGAGTTGGTGCAGCAGCTGACGGCGGCGGCGGAGCTTTGAGGTCTGGGTCGCGAGGAAGACGAAGAAGGGGAGGAAAGGAAGATGGCCTTCAAGTCTTATTTATAAGGCGCGGCGCGCGTGTCAGGCGCGCGAATCCAGGAGCCGGAGATTTCGGAAGCCCAGCCGTCGCCCTGATTCTTGCGGGTTAGCGAAAGGAGACGGTAGCTTGCGCTGACGTCATATCGGTTTACCACAGCAAGAGGAGACGGAGAAACGACGGTTTAACAAGTCACTGGAAGACATTTGAAGAAAGAAATTCTTATTAAGGATTGACATGAACCTGTTCAAATCAATCTGGGGCCTAA

TGTTGGGGATGTTGCTATCCGGGGTAAACCGGCCTGCAGGGCCCGGGTTACCTCCGTCAGCAGACCCGAGATGTTAAAACCCAAGGAGAGATAGAAGGGCCTAAGGCCCATAATCGGTTTAAAGGTTTATGGTGTAAACCGGCCTGATGTAAACTTGTATTGTAAGTTAGGAATAGAGAGACCGACCCGGGCACGATTATTAGCCGGAGGTTGGGACTTTGCAGACCGACGGGCGTCACCCGTGTATATAAGGGGATGACCTGGCGGCGGTTTGAGGACAGAGAAAGAGAACTCGAGGTTTAGGCGAAGCTTATCGCTCCCTGAGCCCTCGAAACCCATCAATCCCATCACAACTAGACGTAGGCTTTTACCTTCATCGAAGGGGCCGAACTAGTATAAAACCGTGTCCTTGTGTCCACTTTAACCCCTTCAAGCTAACCCGTTGCGATGGCTCCACGACTAAGTCCTCTTACTAGGACATCTGCCGTGACAAACCGACGACA

GGGAGTATAAACTTTACCATTCTGTTTGGGAACCGCCTATAATGTGTGTAGCATGGAAGATATCGCCATCTCTCAGTTGTTATGTTGACAATGAAAGTATGCCGCTCAAAATATTATGTATCTC

>Aegilops umbellulata 1-145;

TCCTTGGCACGTCTTGTCAAAGCAGAGATCGTGTCCCCCTTTTACGGGATTCTCATCAATACAGACGTGGGTAACCCAATCGTGCCCGTTAGCACGTTTCCTCGATTAAAGACGAGTCCCAAAC

TGTCGTGGATTTGTCACGGCAGATGTCCTAGCGAAAGGACTTAGTCGTGGAGCCATCGCAACGGGTTAACTTGAAGGGGTTAAAACGGACGCAAGGACACAGAGTTATACTAGTTCGGCCCCTTCGATGAAGGTAAAAGCCTACGTCTAGTTGTGATGGGATTGATGTGTTTCGAGGGCTAGGGAGCGATAAGCTTCGCCTAAACCTCGAGTTGTTATTCTCTGTCCTTAAACCGCCGTCGGGTCGTCCCCTTATATACACGGGTGACGCCCGTCGGTCTACAAAGTCCCAACCTCCGGTTCATAAACGTGCCCGGGTTGGTCTCTCTATTCCTAACTTACAATGCAAGTTTACCTCAGGCCGGTTTACGGCCATAAACCTTTAAACCGATTATAGGCCTTGGGTCCTTCTATTTCTCCTTGGGCTTTAACATCTTGGGTCCATTGACGGAGCTAACCCGGGCCCTGTAGGCCGGTTTACCCCAAATAGCAATATCCCCAACA

TTAGGCCCCAGATTGATTTGAACAGGTTCATGTCAATCCTTAATAAGAATTTCTGTCTTCAAATGTCTTCCAGTAACTGTTAAACCGCCATTTCGTCGTCTCCTCTTGCTGCTGTAAACCGATATGACGTCAGCACAAAGCTACCGTTTCCTTTTGCTCAAACCGCAATAATCAAGGCGACGGCTGGGTTCCCGAAATCACCGGCTCCTGGATTCGCGCGCCTGGCACGCGCGCCGCGCCTTATAAATAGGACTAGAAGGCCATCTTCCTTTCCTCCCTTTCGTCCCCTTCTTCGTCTTCCTCGCGACTCAGACTTCGGAGCTCCGCCGCCGCCGTTAGCCACTGCATCAACTCGGGCCGCTGCATCAACCTGTCCGGACCAGAGTACCGCGGCGACCCTCCGCATCCGTTTGGATCCCGGTAAGGCTCCTGTTCTTCTTGCCTCAGATCTGCTCTAGGGTTCATCATCCTGCCACTGTTCATTGTTCATCTCCGTAGCAAGCTTTTGTGAACAGATTATGATACTTATATTGTGATTTGTAGTCACTCTCAGTATTCGTCATAAATCTGTACTCATCTCCTTAGATCCCACTCATTTCCCTGCTTGTCGAACTTGTGCTGTTCTGCTGCGAGCCAAACCTCATATTTTTACTTCTTTTTTGCCCTAGCTGTAGATCCATTGTTTTGAAAGAATCCGTACGGGATCTGTTTCTGCTTTCACATTCACAACTGTTAGATCTGGTCGGTTTAACTTTTAGCAGAGCGAACAACCCAAACTATATATCCATTAGTCCCCTTGATGAACCGCCAGATGATGTCATTGTAGATAACTCCGTTCTATATAAGAGCATCTTTGGTTTACAATCACACCTGTTCTCAAACCGGAATACCTTTGTCTTGTAGATTTCCTCATGGCAAAACAGGTATATGAATGCAACTGGGTTCCCTCTCGCATCACCGAGACCCAATTAAACAACTTAGTTCTGACTGGCGCCTTGAGTAGCAAAAATGCCATCCATTGGAGGGTTCCAGGGAATGAATGTCCTCCTACCCCACAGGAAGGAGAAGTTGTTGTGTTTGCGGATCACCTGGCCCAGGGCTTTAATCCACCCGGTTCAAAATTTTATCGGGACGTCCTGGCTAACTTTAAGTTGCGCCCACAAGATGTTGGTCCAAACTCAGTCACGAATATCTGCCATTTTCAAGTGCTTTGTGAGGTGTACTTCCAGGAGGAACCTACTGTAGAGTTGTTCCGAGACTGTTTTCATTTGAACCGATGTACTGAATTCACCGACGGCCCTAATATGGAACTGGGTGGTATGGCGATTCAAAAGAGGAAAGAAGTGACTTATCCTCACGCCAAGCTACATAGTCATCCGCAAGAATGGAATCACACTTGGTTCTATTGCAAGGATACTTCCCCTGCTGAAGAAAATCCTCTGCCTGGCTTCCGTCCAGAGAGACTTAGCAATACACACCCCTTCCCCCCAAGGCTAACCGCCAAAGAGAGGAGTAAGTATGCCCCTCAACTATCCAAGCTCAGAGCCTTCATGGCTAACGGTTTAACGGGGGTAGATCTTGCCCGTTGCTGGATTTCATGGAGCATCCTGCCCCTTAGTATCCGCTCCGGTTTAATGTGCCAATACACGGGCCGGGTTAATGATCCTTTGAGGCACTGCAACATTCAGCTGACGGAAGACGAAGTTACTGAAGCTGTGAAAAAGATGCTGAACGAACCGGAATATGTTTGCGCCCGAACCGGCCTGCTTCCCTTTTGTGCTACAAATAAACCACCTGCTGTAAGCATCTCACCTCTGTTTGTTATAGAATTTATGATCTCCTTATATGCACCCTGTTAACTCCAGTCCTTTCTGAGACAGGGAGATGATCCGTTTTGGAGCAAGAAGCTTCAAGAGCCGACAGAAAAGGTAACCCGACCCAAAACAAGGGTGAAGAAACCCGCTCAGAAGAAAAGGAACACCGCTTCCTCTACCCCGGATTTAGATGAGGATGATGTGGGTAATCCGGACCTTGAGGTAGACCTCGATTCAGTTGGCTTACTTTTTATACGTCTTATTGATGATGATATTTGTCAGGACGACGTTGAAGCGAGTAATGCTGAAGGCCCAGAGGTAACTATTCTTTCTTCTGATACAGTTCCTTTGCCTTTGAAAAAACCTCGCCAAGCAAACCGGAAAGTTAAATTTTCTCATCCTCTTGCTTACTTGGATCCCAAATTTCTTGTGAAGACCCAGCAACATGAAGCTCGTCGCACCACCCGGCATAGTGGCCAGGTAGTTACCTCCGCCGGTTTACCGAACAGCCCGGTTCGGAAACGCCGCTCAGAGGTCTCCGATTTATGCGCTAGCTTTCCTCCTAAAGCAGGTTTAACTCGTCAGCCTCTTAATCCATCTGACTCCGATTATCAGGTCACTTCCCATTCATCTTCTGGTGAATCTTCAGCCACTCAACTGCCACCGCTAAAGACAGTTATTGGGTAAGTTGGACCGATCACAACATATTTCCATGTGGGTTTCTTAATCCATCTTACACCGTTCTTTTCAATCTTTGGGTCTTTCCCAGGGCTAAACCTAGACCGAGCAAGAAGGCTCGTCTGGATAATCCGGCCGAAGGAACTGCCGCTCCTGAACCGGAGAGAACTCCTGGTGCTGACGCTCTAGTTCGTGAAGACATTCAGAATTATCTACCTCCACAAGATGACGCCTATGCTGAAGAGAGGCCTACCAATCCTGCCAGCCGTACCGATCAGCCTGCCAGTCCTGTTCGGATTGAGGAATCCATCCACCCTTCTGGAACTGTTGATAAACCGACAGTGCCAATGCAGAGCAGCGATTCCAAAGATGATGATGTTGTGATTACCGGCATTGGTCGCTCTGAACCGGGGAACTCTGCTACTTTAGCCAAGCATACTGCCAAGGAAGAAATCTCGGCCATAAAGGGCAAATGGGACGTTGATTCCTCAACGTACGCCGCTTTAAGCGCCCAAGATCTTTATTCTGGGTACCTGAACCGACTGTACACCAGTCGCGATTATGAAGCTGGCATGATCAGGATGATGAAAGAAAAACTTGAGGTAACTCTCTTGTGCTTCATTCTGTTGCAACTTAGCTTCTCAATTTTTTCCAATCCGAGATGGTAATCCGAATCATGCTGCCTTGACAACTAAGGTCCGGTTTAAAAACCCAGGGCTTCAACATAGGTCGTAACCAGAAAACATCATCAAATAACCATTAGCCCCCAAGTGCCAAGTTTAAATACTTGTATTAATCCTGGGACTTTTGTAATGTAAAACATGCGTTAAGATCAAGTACGCATTAGCCCCCAAGTATCGAGCACATAACTTGCTTGTGTGGTTGATACTTCCAAACCGTGCAATATTTTCTGAACACTGCTGATGACCTGCTGCAGGGGGATCTGAAGACCAAAGAGAAACAAGTCTCGGACCTTCAGGAAATCCTAAAAACCCAGCAGGCTGAAACTGCCAAAGCGAAGGAAGAATTGACCAATGCCTTAACTGTTATGGAACAGCTGAAAGAAGCCCGCAAAAAGGAACAAGCTGATTGGGCTACTGAAAAAGCTCTTTTGATTAAAAGGGCTGAAAATGCCGAAGCTGCTCTTAAACCGGTGGTTGATGAATTATCCACCGTAAAGCGACAAATACACTCCATGACCTCTGCCGTCTTTGGTAAATTTCTTTTGATGCCCTTCCTCCAATTTGCACTTTGTCTGCTATATCCTATGTCCGGTTTGTAATCCATCTTGAAACACTGCAAGCACACGTATTGGACACTTGGGTTCTGATGTGCGGATGAAACTCAAAGTTGCTTATACTTTGATTGAACAGTTGTATACCGGAGCACAGAGAGCTATCACCACTGCATCGCATAACAACCCCGCACCGTCTCTGATTCAAGACACGTTAAAAAGGATATCCATGCTTCCTGCCCGGATTGAAGAGTTAAAAAGATCAGCTGCTCGAACCGGAGCCATCACTGCCTTGATCCGGGCCAAAGCGTGGGTGCCAGACTTTGATCCTGTCGAAGCGGCACAAGGATATCCCAGCTTGAAAGAAGACGGTTCAGACTTCGGTGAAAATGACTTGAGGGCGATAAACCGGGCGGTGCGCCCCCTAGCTTGTCAATTGGCTGAAGAAGCAGATCTGACACATTATCAGGCCCAATATGATAACCAGAACAAGCGAGTAGCTGCCCCGATCCCTGAAGCGGAAAACCTTATTCCTCCAATCCGTAAGCATACCTACGCCCCTGACATTGAACCGTCTTTGCTGATTCATGAAGAAGCTGTCTTTCAAGCGTTAATGGGAATCGACTGGACCACTGCTGATTTCCAGCCAATGGGTAGCCATGATAAGGCTGAAGCGGCGCAAGATGATCCACAACCTTCGAACCGAGGCGGCGAACAGGCGTAATCAAAGCTCCGGTTCACCTCCATTGGTGATGTGAATCCGTAAAAAAAAACAATATATTCAGGCACTACGCTGCCTTGTAATAAGATAGTTAATACGTCTGTGTTTCAATGCGCCTTTGTGCACGGGTACTGATGCCCTTGCCATTGACTTTTTCCAACTTGTGTTTCCTGTCTTTACTGGGTACAAAGGACGGTCTTTCGACCATACGAACCGTGCTGCGCCTTTGCCAAACCGGGCAACTTGCCTCCGGGTTAAAAAATGGACCAGGCTGTGTCAGCTGAATCGGTTTAAAAGTATTTGTCGTTCCGAAGAACAAAAACACCACAAGAAGGAAGATAACAAAAAACCAAATACGAGAGGAAAACGCAATAGTAAAACGCATGCAAATGGTGGTTGCTATGAACCGTTCAGAAGGCGTTACCATCGTCAAGACAAAGAAAACCAAGCCCCCAAGAGGTGTAGCTACGGTTTAAGTCGACTAGGCTCCTAACTGAATCGTGGCAAATATGCCGGTCAAATGGCATTGCATTGGTTCGGATTCGACCAAGCCCCCAAGTGACTCAGTGGCCTAGGGCCGATCAAGTGGCATTGCGTTGGTTCGGACACGACCAAGCCCCCAAATGACTTATTGGCTGTTTAAAAGCCGATCAGAAGGCATTACCATCATCAAGACATGACCGAGCTCTCATAAGGTGTAACTACGGTTCAAGTCGATTAGGTCCCCAACTGACTTAGTGGCAAATGTGCCAGCCAGAAGGCATTACCGTCGTCAAGACATGACCAAGCTCCCCGGAGGTGTGGCTACGGTTCAGGTCGACTAGGTCCCCAACTGACTTTGTTGATTTCCTGTGAAGCGATCTCAAGGGGTAAACCGGAGGTTGCCCTAAAGCGAACCAACTCCGTGTAACCACGCATGATGTGAAGAAAAAAACAGAGACCCCGCTTAACTCGAGGCTCCGGTTCATTATATTGATCATAATATATACATGGTCATGATATGTACATAAACAGAGCCCAAGGCTCATGTATAGTAAGGCCGAAGATGAGCTATGTTCCACGGCCTGCTGGTCTCTTCTTCTGATGTGCGTGAATCTTTGCGCTCTCGAATATCAATCAGGTAGTATGACCCATTATTCAGATTCTTGCTGACCACAAAAGGTCCTTCCCAAGGTGGGGATAATTTATGCTGATCAGACTGATCCTGGATGAGCCGAAGCACCAAATCCCCCTCCTGAAAGGTTCTGGTCTTAACCCGACGGCTGTGGTAACGGCGAAGATCTTGCTGGTAAATCGCTGAACGGGCAGCTGCTAATTCACGTTCCTCATCCAACAAGTCAAGAGCGTCCTGCCGTGCCTGCTCGTTGCCCGCTTCAACATAAGCTGCCACACGAGGTGAATCATGTCGTATATCACTGGGGAGAACCGCCTCTGCTCCATAGACCATAAAGAACGGCGTAAACCCCGTGGATCTGTTAGGCGTGGTGTTGATACTCCATAATACAGACGGTAGCTCCTCAACCCAACAACCCGGCGTCCTTTGCAAAGGGACCAGAAGCCGGGGTTTGATACCTCTCAAGATCTCTTGATTCGCTCGTTCTGCTTGACCATTAGATTGTGGGTGAGCTACTGAAGAAACATCAAGCCGTATATGCTCACGTGCGCAGAACTCTTTCATGGCACCTTTGGATAGATTGGTACCATTATCTGTGATGATGCTGTGTGGGAAGCCAAGCCGGAAGATCACCTTCTTTATAAACCGAACCGCCGTGGCCGCATCACATTTACTGACAGGTTCTGCCTCCACCCACTTTGTAAATTTGTCAACCGCCACCAATAGGTGGGTCTTCTTATCTTTGGAACGTTTAAAAGGCCCAACCATATCGAGCCCCCAAGTCGCAAACGGCCAAGTAATTGGAATCATCCTCAATTCTTGAGCCGGTATGTGCGCTTGTCTGGAAAATTTCTGGCAACCATCACATAACCTGACCAGGTCTTCTGCATCAGCATGAGCAGTTAACCAGTAGAAACTGTGGCGGAACACTTTAGCCACCAATGATTTTGACCCGGCGTGGTGACCTCAATCCCCTTCATGTATTTCACGTAATATCTCACACCCCTCCTCAGGGGATACGCACCGTTGCAGCGCTCGTGACACGCTGCGATGATGTAGCTCCCCTTTGAGAATGGTCATAGACTTGGACCGCCGTACTATCTGCCGAGCCAAGGTTTCGTCTTCCGGCAACTCACCCCGGTTCATATATGCCAGATAAGGAAGCGTCCAATCCGGGATGACGTGAAGAGCCGCCACTAATTGAGCCTCCGGATCAGGAATAGCCAACTCCAGCTCACCGGGTAGCTGTACCGATGGGTGATACAATACATCCAAGAAAACATTGGGTGGGACCGGTTTACGCTGAGAGCCCAAGCGGCTTAAAGCGTCCGCTGCCTCATTCTTTCTGCGGTCCACATGGTCCACTTGATAGCCTTTGAAATGCCCAGCCACCATATCTACTTCACGACGGTATGCAGCCATGAGCGGGTCCTTAGAATCCCAGGTGCCAGATACCTGCTGAGCCACTAGATCCGAATCACCAAAGCACCTAACCCGGCTCAAACTCATCTCTTTAGCCATCCGAAGACCATGAAGCAAAGCTTCATACTCAGCCGCATTATTTGTACAGGGGAACATTAAACGGAGGACATAACAAAACTTATCTCCTCGTGGGGAAGTTAAAACGACTCCAGCCCCCGAGCCTTCCAATTGTCTGGATCCGTCAAAATGAATAGTCCAATATGTGTTATCTGGCTTCTCCTCAGGTGCTTGTAACTCTGTCCAATCGTTTATGAAGTCGACAAGTGCTTGTGACTTTATGGCCGTCCGGGGTACGTATCTTAAACCGTGCGGCCCAAGCTCGATAGCCCACTTGGCAATCCGACCGGTCGCCTCCCAGTTCTGTATGATGTCACCCAAAGGAGCAGAACTGACCACCGTGATGGGGTGTCCTTGAAAATATTGCCTCAGCTTCCGGCTTGCCATAAAAACACCATATACCAGCTTCTGCCAATGCGGATACCTTTGTTTGGACTCGATAAGCACTTCGCTGATATAGTAAACCGGTCGCTGAACCGGATGCTCCTTCCCAGCCTCCTTGCGTTCTACCACCATCGCCACACTGACCGCTCTGGCGTTTGCAGCAACATATAACAGGAGTGGCTCTTTATCTACAGGGGCTGCCAACACTGGCGGATTGACTAACTGTCGCTTCAAGTCCTCAAACGCTTTATCAGCCTCCGGACTCCAGACAAATTGATCTGTCTTCTTGAGCATTTGATACAAGGGGATCGCCTTTTCACCGAGGCGGCTGATAAACCGGCTTAACGCTGCAATCCGCCCGGCCATGCGCTGAACATCATTGATGCATTTCGGTTTGGCCAGGGAGGTGATGGCTGTGATCTTTTCCGGATTGGCCTCAATTCCTCTATGGGACACTAAGAATCCCAACAACTTGCCTGCAGGTACACCAAAAACACATTTAGCTGGGTTGAGCATCATTTGGTACACTCTCAAGTTATCGAAGGTTTCCTTCAAATCATCAACCAGGGTCTCCTTCTTTCGTGACTTGACCACAATATCATCCACATAAGCATGTACATTACGACCAATCTGTTCGTGGAGGCAATTTTGCACACACCGTTGATAAGTAGCCTGCGCACTCTTAAGCCCGAAGGGCATAGAAACATAGCAGAAGGCTCCAAAGGGAGTAATGAACGCCGTCTTCTCCTGGTCCTTAACTGCCATCTTGATCTGATGATAACCCGAATAGGCATCCAAAAAACTCAAACGCTCGCAACCTGCCGTAGCATCAATGATTTGATCAATACGGGGGAGAGCAAAAGGATCCGCTGGACAGGCTTTATTCAAGTCTGTGTAATCCACACACATGCGCCAGGTGCCATTTTTCTTAAGAACTAGCACCGGATTAGCAAGCCACTCGGGATGAAAAACCTCGATAATAAAACCAGCTGCCAGGAGCCTGGCTACTTCCTCACCAATCGCTTTACGTCTTTCTTCGTTAAACCGGCGTAGGAACTGCTTTAACGGTTTATACTTAGGATCCACATTAAGGGTGTGCTCAGCGAGTCGCCTCGGTACACCAGGCATGTCAGACGGCTTCCATGCAAAGATGTCCCGATTCTCACGGATGAACTCGATGAGCGCGCTTTCCTATTTTGGATCCAAGTTTGCACTGATGCTGAACTGCTTGGACGAATCGCCAGGTACGAAGTCAACAAGCTTAGTTTCCGCAGCTGATTTGAACTTCAAGGCCGGATCGTGGTCCGTAGTTGGCTTCTTTAATGAAGTCATGTCCTCCGGATCAACATTGTCTTTGTACTGCTTTAACTCCTCCGTAGCACAAACCGATTCTGCATAAGTCGCATCTCCTTCCTCGCATTCCAAAGCGATTCGACGGCTCCCATGAACCGTTATAGTGCCCTTGTAACCCGGAATCTTGAGTTGCAGATATACATAGCAGGGCCGTGCCATAAATTTTTCATAGGCCGGTCGTCCGAACAAAGCGTGGTATGGACTTTGGATTTTAACCACTTCAAACGTCAGCGTCTCTGATCTGGAATCATGACTATCGCCGAAGACCACTTCAAGGGCTATCTTACCAACCGGGTACGCAAACTTGCCAGGCACCACCCCGTGGAACACTGTATTGGTCGGTTTGAGATCCTTATCCACCAGTCCCATGCGACGGAAGGTCTCATAATACAAGATGTTAATGCTGCTCCCTCCGTCCATGAGTACCTTGGTGAGCTTATAACCTCCCACCTGAGGCGCCACCACCAATGCTAACTGACCCGGATTATAAACCCTGGGAGGGTGATCTTCTCGGCTCCATATGATTGGTTGTTCCGACCAGCGCAGATAGTAAGGCGTGGCCGGTTCAACAGCACTAACTGCCCGCCTCTGAAGCTTTCTATCCCGTCTATCCAAGCTTGTAGTAAAAACATGATACTGTCCGCCACTCAACTGTTTCGGGTTGCTCTGATAACCTGATTGCTGCTGTTGCTGCTGGCTGTAACCACTCTGATTACCTTGATTATTTTGGCTGCTCTGTCCGCCCGGATTACCATGGAACCCTGGACCGGAATTTCCGCCGTCGTACCCTGATCCGGAACCTGAACCGCCGCCAGAGCTGTGATCATATTGGAAAATGTTTGAGCTTTTGAATTCCTTCATGATGTAACAATCCTTCCAAAGATGGGTTGCTGGCTCCTCTTTTGTTCCGTGCTTCGGACAGGGCTGGTTTAAGAAAAAGTTCAAACGCTCCGGATTAGGACCGGGATTTCCGCGAAAACCTGGCTGTTTCCCCTTACGACGCTGGCCCTTATTCTGTGCACTTGTGTTGGCCACAAAATCCACGCTCCCATCCGGTTTACGCTTACCTCCGCCTCCATTACCTGTTGGTCGATGCTGCTGGCCTTTGGTGTTGCTATTCTTCTTTCCCTTCCCTGCTTTGTCGTCACCAGAGTCAGGATCCTTGGTACTGTCAGAATCCGCATACTTCACTAAAGCGGTCATGAGCGTTCCCATATCCATGCAATCACGCTTCATCCGGCCTAACTTTAGCTTCAAAGGGCCGAACCGACAGTTGCCTTCCAACAGCACCACGGCGGTGTCTGCGTTGATGCGATCTGATGAGTGCAAAACTTCTGATACCCGGCGTACCCAATGGGTAGTTGACTCCCCTTCTTGCTGGACGCAAGCTGCTAGGTCAACTATCGACATTGGCTGTTTGCACGTGTCCTTGAAATTTTTGATGAACCAGGCTCACAACTGGGCCCACGAATTAATGGAATTAGGCGGTAGGCTCTTTAGCCAAGTCCGGGCCGTTCCTTCTAACATCATGGTAAAATACTTCGCACACGCTGTGTCATCCACATCCAGCATCTCCATAGCCATCTCATAGCTCTCCACCCATGTCTCCGGAGGCTGATCTGCCGTGTAGTTTGGTACCTTGCGGGGGCCCTTGAAGTCTTTCGGCAGGCGTACGTTGCGCAAAGCGGGAACAAGGCACGGTACTCCCAAAGAACTAGAAACCACCCCCGGTTCAATCGTAGCAGCTGGGAAGATAGGTGTAAGCTGACGAGCCTGATGCTGCGCTGCTAATCCGGCCTCCTGCGCACGGGCACGATCCACATTCTCCTGAGCATTGTCTACGCCCGCCGGATTGTTGCCGCGGAGCGCTTCACGCCTGGCATTACTTGAAACAGCCGGCTCCTCCATGCGCCTACTATAGCTCCTGCTGGGGCGAGGGGTGGAGTGAATCCGATCATGGCTTTGCGAATATGCTTCTTGTTGGTCCAAAGCGGTCCTAAGGAGCTCCTTGACCCGGCGTGTCTCTTCATCCTGCGGTGAATTGCCTTCGATCGGAATGGCTTCCAACCGAGCTGCCGCCGCGACGAGGTTATCCATCGGGTTGGAATAATGGTCCGATGGTGTTGGCACGGCCTGTGGTGTACCAGTGTTGTGACGAGGCAGGGTTATTATACGGTGTTGAACCGGGGCGCCAACTGTCGCTTCCGCGCGGTTTACCCCCGGGGTGTTGAAGAGATTTCTGGCATCGAGGGCTGCGGGCACTCGAGATCGGAATTTCCTCTTCAGGACTTCCTGAGACGCATTCTGGTCCATCATGAACCTGTAAGCCTGTGCATCTAAAGTGGCGCGCTCTGCTGTCATCCTGGCCTCTTCTGCTGCCAGCTCCGCTTTAGCTTGAGTGATCTGTTCTCTCACTTTGGCAATCTCGGCATTGTGAACCTCCTGATCCACCGGGTTGGCTTCCGCCATGAGCGCTGCCAGCGCGTCAAACAGATCTGACAATACTTGGGCTGGTGAGTCCGCAGGGCCTCCTGCCCGCGCAGCTGTTGCCGCTGCCGATCCGGAGATCACTGCTGCCGCAGTTGATGAATGTGGCGCCGCCTGTGTTCCGGCCATGAAGATCCCAACTCGATAGGGCGGATCAGAGGGGTCCGGAATACTATCGCCATCGGAGCTGCCCCCAATCCGATCATCTTGCAGCTGGTATAGAGACTCGGTTTCCCCGGTTGACGTTTCATCGCCGGAGTAGATGGCGGTTTCGTCACCGGATTCAGATCCTTCCTCGTAGCTTCCTCCGTGGATGACTCCCATGAAGGCGCGCTTCCTGGTCGGTTTAGCCAGGGTCGATCTCGTGCACTGAGCTGTCTCGACGAGGTCGGTGCAGACGCCCAGCTCGGGGCCCGGTTCACCGATCTTGCCGATGAAAACATGGATGCCGCCAAAGGGGACCCGGTACCCGTACTCGATCGAGCTGGCGTCGGGGCCCCAGCCTGTGCTGTCGATGTAGAGTTTTCCGCGACGACTTTTGGTCATCCGGCCCACAGCGTAGCCCTCGAGTCCTTCAAAGCGGCCCTCCAAGAACTTGAAACCATCTCGCGATAGCCCCACGGTGGGCGCCAAC

TGTCGTGGATTTGTCACGGCAGATGTCCTAGCGAAAGGACTTAGTCGTGGAGCCATCGCAACGGGTTAACTTGAAGGGGTTAAAACGGACGCAAGGACACAGAGTTATACTAGTTCGGCCCCTTCGATGAAGGTAAAAGCCTACGTCTAGTTGTGATGGGATTGATGTGTTTCGAGGGCTAGGGAGCGATAAGCTTCGCCTAAACCTCGAGTTGTTATTCTCTGTCCTTAAACCGCCGTCGGGTCGTCCCCTTATATACACGGGTGACGCCCGTCGGTCTACAAAGTCCCAACCTCCGGTTCATAAACGTGCCCGGGTTGGTCTCTCTATTCCTAACTTACAATGCAAGTTTACCTCAGGCCGGTTTACGGCCATAAACCTTTAAACCGATTATAGGCCTTGGGTCCTTCTATTTCTCCTTGGGCTTTAACATCTTGGGTCCATTGACGGAGCTAACCCGGGCCCTGTAGGCCGGTTTACCCCAAATAGCAATATCCCCAACA

CAAACGGTTACGGGGAGGGCTCTTGGTATTCAACCTCTTATAAAGAGACCAAGGCCTTACTCCTTTCAATCTCAAGCGAGTTCGCCCGTCGCCTCGAGTTCCAACACCCTAGGCTCCAGATTCC

>Aegilops umbellulata 1-173;

ACCTTCAAGTTGAGGAGTGCCACGACATCGAGCTATGGTACAACACCACCCATGACAAATTTGCACCCTATGCATGGATTGACACATATTATGATTGTCTTGTTGCACTTATTCCCATGTCATC

TGTTGGGGATGTTGCTATTCGGGGTAAACCGGCCTGCAGGGCCCGGGTTACCTCCGTCCACAGACCCGAGATGTTAAAGCCCAAGGAGAGATAGAAGGGCCTAAGGCCCATAATCGGTTTAAAGGTTTACGGTATAAACCGGCCTGATGTAAACTTGTATTGTAAGTTAGGAATAGAGAGACCGACCCGGGCACGTTTATAAGCCGGAGGTTGGGACTTTGCAGACCGACGGGCGTCACCCGTGTATATAAGGGGATGACCCGGCGGCGGTTTGGGGACAGAGAAGAAGAATTCGAGGTTTAGGCGAAGCATATCGCTCCTTAAGCCCTCGAAACCCATCAATCCCATCACAACTAGACGTAGGCTTTTACCTTCATCGAAGGGGCCGAACTAGTATAAAAACCGTGTCCTCGCGTCCACTTTAACCCCTTCAAGCTAACCCGTTGCGATGGCTCCACGACTAAGTCCTCTTCCTAGGACATCTGCCGTGACAAATCCACGACA

GTTGGCGCCCACCGTGGGGCTATCGCGAGATGGTTTCAGGTTCTTGGAGGGCCGCTTTGAAGGACTCGAGGGCTACGCTGTGGGACGGATGACCAAGAGCCGCCGCGGAAAACTCTACATCGACAGCACAGGCTGGGGCCCCGACGCCGGCTCGATCGAGTACGGGTACCGGGTCCCCTTTGGCGGCATCCATGTTTTCATCGGCAAGATCGGAGAACCGGGCCCCGAGTTAGGCGTCTGCGCCGACCTCATCGAGACAGCTCAGTGCACGAGATCGACCCTGGCTAAACCGATCAGAAAGCACGCCTTCGTGGGGGTCATCCGCGGAGGAAGCTACGAGGAAGGATCCGGATCTGGTGACGAAACTGCCATCTACTCCGGCGATGAAACGTCGACCGGGGAAACCGAATCTCTTTACCAGCTACAAGATGATCGGATTGAGGGCGGTTCCGATGGCAACAGTATTCCGGACCCCTCGGATCTGCCCTGTCGGGTCGGGATCTTCATGACTGGAACACAGGCAGCGCCACGATCATCAACTGTAGCAGCAGCGATCTCCGGATCGGCAGCAGCAACGGCTGCGCGGGCAGGAGGCCCTACAGGTTCACCAGCCCAAGTTTTGTCAGACCTGTTTGACGCACTAGCAGCGCTCATAGCGGAAGCCAACCCGGTGGATCTGGAGGCTCACAATGCTGAGATTGCCAAAGTGAGAGAGCAGATCGCTCGGGCTAAAGCGGAGCTGGTAGCAGAAGAGATCAGGATGACGGCGGAGCGCGCCGCTCTAGATGCACAGGCTTACAGGCTCATGATGGACCAGAAGGCGTCTCAGGAGGTCCTGAAGAGAAAATCCCGGTCTCGGTTGCCCGCCGCCCTCGACGCTCGGAACCTCTTTAACACCCCGGGGGTAAACCGTGCGGAGGTGACGACCGGCGCCCCGGTTCAGCACCGGATTATAAACCTGCCTCGTCATAACACTGATACGCCACCGGCCATGCCAACGCCGTCAGGTCATTATTCTAACCCGATGGATAACCTCGTCGCGGCGGCGGCTCGGTTGGAAGCCATTCCGATCGAAGGAGATTCACCGCAGGATGAAGAAACACGCCGGGTCAAGGAGCTCCTTAGGACCGCGTTGGCCCAGCAAGAAGCATATTCGCAAAGCCGTGATCGGATTCACTCCACCCCTCGCCCCAGCGGGAGCTATAGCAGGCGCGTGGAGGAGCCGGCCGTTTCAAGTAATGCCAGGCGTGAAGCGCTCCGCGGCAACAATCCGGCGGGCGTTGACAATGCTCATGAGAATATGGATCGCACCCGTGCACGGGAGGCCGGATTGGCAACGCAGCATCAGGCTCATCAGATTACACCAACCTTCCCAGCAGCTGCGGCTGAACCGGGGATGGTTTCCAGTTCTTTGGGAGTACCGTGCCTTGTTCCCGCCTTGCGCAATGTACGCCTGCCCAAAGACTTCAAAGGTCCCCGCAAGGTACCAAACTACACGGCAGATCAGCCTCCGGAGACGTGGGTGGAGAGCTATGAAATGGCTATGGAGATGTTGGATGTGGATGACATGGCGTGTGCCAAGTATTTTACTATGATGTTAGAAGGAACGGCCCGGACTTGGCTAAAGAGTCTGCCACCTAATTCAATTAGCTCGTGGGCCCAATTGCGTGCCCGGTTTATCAAGAACTTCAAGGACACGTGCAAACAGCCAATGTCAATAGTTGACCTAGCAGCCTGCGTTCAGCAAGAAGGGGAATCAACTACCCATTGGGTACGACGAGTATCAGAAGTCTTGCACTCATCAGATCGCATCAACGCAGACACCGCTGTAGTGCTGTTGGAAAGCAACTGTCGGTTTGGCCCTTTGAAGCTAAAGCTGGGCCGGATGAAGCGTGATTGCACAGATATAGGAACGCTCATGACCGCTTTAGTGAAGTATGCGGATTCTGACAGTACCAAGGATCCTGACTCTGGTGATGACAAAGCAGGGAAGGGAAGGAAGAGTAGCAACGTCAAAGGCCAGCAGCATCGACCGACAGGTAACGGAGGCGGAGGTAAGCGTAAACCGGATGGGAGCATGGACTTTGTAGCCAACGCAAGTGCACAGAATAAGGGCCAGCGGCGCAAGGGAAAACAGCCAAGTTTCCGCACAGATCCTGGTCCTAATCCGGAGCGTTTGAATTTTTTCTTAAACCAGCCCTGTCCGAAGCACGGGACAAAGGAGGAACCAGCAGCCCATCTTTGGAAGGATTGTTATATCATGAAGGAGTTCAAAAGCACAAACACGTTCCAGAATGATCGCAGCTCCGGCGGCGGTTCAGGTTCAGGTCCAGGGTTCCAGGGTAACCCGGGCGGACAGGTCAGCCAAAATCAGAATAATCAAAGTAATCAGAGTGGTTATCAGAGCAACCCGAAACAGTTGAGTGGTGGACAGTATCATGTCTTTACTACGAGTTTGGATAAACGAGATAGGAAGCTTCAGAGGCGGGCAGTCAGTGCTGTCGAACCGGCCACGCCTCACTATCTGCGCTGGTCAGAACAGCCCATCATATGGAGCCGAGAGGATCACCCTCCCAGGGTTTATAATCCGGGCCAGTTAGCGTTGGTGGTAGCGCCTCAGGTGGGAGGTTATAAGCTCACCAAGGTGCTCATGGACGGAGGGAGCAGCATTAACATCTTGTATTACGAGACCTTCCGTCGTATGGGACTGGTAGATAAGGATCTCAAACCGACCAATACAGTGTTCCACGGGGTGGTGCCTGGTAAGTCTGCATATCCGGTTGGTAAGATAGCCCTTGAAGTGGTATTTGGCGATAGTCACGATTCCAGATCAGAGACGCTGACGTTTGAGGTGGTTAAAATCCAAAGTCCGTACCACGCTTTGTTCGGGCGGCCAGCTTATGCAAAATTTATGGCACGGCCCTGCTACGTATATCTGCAACTTAAGATGCCAGGTTACAAGGGCACCATAACGGTTCATGGGAGCCGCCGAATCGCTTTGGAATGCGAGGAAGGAGATGCGACTTATGCTGAATCGGTTTGTGCGACGGAGGAGCTAAAGCAGTACAAAGACAGTGTTGATCCGGAGGATATGACTTCATTAAAAAAGCCGACTACGGACCACGATCCGGCCTTGAAGTTCAAGTCAGCGGCCGAAACTAAACTTGTTGACTTCGTACCTGGCGATTCATCCAAGCAGTTCAGCATCAGTGCAAACTTGGATCCAAAATAGGAAAGCGCGCTCATCGAGTTCATCCGTGAGAATCGGGACATCTTTGCATGGAAGCCATCTGACATGCCTGGTGTACCGAGGCAACTCGCTGAGCACACCCTTAATGTGGATCCTAAGTACAAGCCGGTAAAGCAGTTCTTACGACGGTTTAATGAAGAAAGACGTAAAGCGATTGGTGAGGAGGTGGCCAGGCTCCTGGCAGCTGGGTTTATCATTGAGGTTTTTCATCCCGAATGGCTCGCTAATCCGGTGCTAGTTCTGAAGAAAAACGGCACCTGGCGCATGTGCGTGGACTACACAGACTTGAATAAAGCTTGCCCAGCAGATCCTTTTGCTCTCCCCCGGATTGATCAGATCATTGATGCTACGGCAGGTTGCGAGCGTTTAAGTTTTTTGGATGCTTATTCGGGTTATCATCAGATCAAGATGGCAGTTAAGGACCAGGAGAAGACAGCGTTCATTACTCCCTTTGGAGCCTTCTGCTATGTATCTATGCCCTTTGGGCTTAAGAGTGCGCAGGCTACTTATCAACGATGTGTGCAAAATTGCCTCCACGAGCAGATTGGGCGTAACGTGCATGCTTACGTGGATGATATCGTGGTCAAGTCACGAAAGAAGGAGACCCTGGTTGACGATTTGAAGGAGACTTTCGATAACTTGAGAGTGTACCAAATGATGCTCAACCCGGCTAAATGTGTTTTTGGTGTACCTGCAGGCAAGCTGTTGGGATTTTTAGTGTCCCATAGAGGAATTGAGGCCAATCCGGAAAAGATCACAGCCATCACCTCCCTGGCCAAACCGAAATGCATCAATGATGTGCAGCGCATGGCCGGGCGGATTGCGGCGTTAAGCCGGTTCATCAGTCGCCTTGGTGAAAAGGCGATCCCCTTGTATCAAATGCTCAAGAAAACGGATCAGTTTGTCTGGAGTCCGGAGGCTGATAAAGCGTTTGAGGACTTGAAGCGACAACTAGTCAATCCGCCAGTGTTGGCAGCCCCTGTAGATAAAGAGCCACTCCTGTTATATGTTGCAGCGAATGCCAGAGCAGTCAGTGTGGCGATGGTGGTGGAACGAAAGGAGGCTGGAAAGGAACATCCGGTCCAGCGGCCGGTTTACTATATCAGTGAGGTGCTTATCGAGTCCAAACAAAGGTATCCGCATTGGCAGAAGCTGGTATATGGCGTTTTTATGGCGAGCCGGAAGCTGAGGCAATATTTTCAAGGACACCCAATCACGGTGGTCAGTTCTGCTCCTTTGGGTGACATTATACAGAACCGGGAGGCGACCGGTCGGATTGCCAAGTGGGCTATCGAGCTCGGGCCGCACGATTTAAGGTACGTACCCCGGACGGCCATAAAGTCACAGGCACTTGTCGATTTCATAAACGATTGGACAGAGTTACAAGCGCCTGAGGAGAAGCCAGATAACACCTATTGGACCATTCATTTCGACGGGTCAAGACAATTGGAAGGCTCGGGGGCTGGAGTCGTTTTAACTTCCCCACGAGGAGATCAGTTTTGTTATGTCCTCCGCTTAATGTTCCCCTGTACAAATAATGCGGCTGAGTATGAGGCTTTGCTTCATGGTCTTCGGGTGGCTAAAGAGATGAATCTGAGCAGAGTTAGGTGCTTTGGTGATTCAGATCTGGTGGCTCAGCAGGTATCGGGCACCTGGGATTCTAAGGATCCGCTTATGGCTGCATACCGACGTGAAGTAGATATGGTGGCTGGGCATTTCAAAGGTTATCAAGTGGATCATGTGGACCGCAGAAAGAATGAGGCAGCGGACGCTTTAAGTCGCTTGGGCTCTCAGCGTAAACCGGTCCCCCCCAACGTTTTCTTGGATGTATTGTACCACCCATCGGTACAGCTGCCCGGTGAGCTGGAGTTGGCTGTTCCTGATCCGGAGGCTCAGTTAGTGGCGGCTCTTCACGCCACCCCGGATTGGACGATCCCTTACCTGGCATATATGAACCGGGGTGAGTTGCCAGAGGATGAAAGCTTGGCTCGACAGATAGTACGACGGTCCAAGTCTATGACCATTTTCCAAGGAGAGTTACATCATCGCAGTGTATCAGGAGCGCTGCAACGGTGCATATCCCCTAAGGAGGGATGCGAGATATTACGAGAAATACATGAAGGGGATTGCGGTCACCACGCCGGTTCAAAATCATTGGTGGCTAAAGCGTTCCGCCACGGTTTCTATTGGTTAACTGCTCATGCTGATGCAGAAGACCTGGTCAGATTATGTGATGGTTGCCAGAAATTTTCCAGACGAGCACACATACCGGCTCAAGAATTGAGGATGATTCCAATCACTTGGCCGTTTGCGACTTGGGGGCTTGATATGGTTGGGCCTTTTAAGCGTTCCAAAGATAAGAAGACCCATCTGCTGGTGGCGGTGGACAAATTTACAAAGTGGGTAGAGGCAGAACCAGTTAGTAAGTGTGATGCAGCCACGGCGGTCCGGTTTATAAAGAAGGTGATCTTCCGGTTTGGTTTTCCACACAGCATCATCACAGATAATGGTACCAATCTATCCAAGGGGGCCATGAAGGAGTTTTGTGCACGGGAGCATATACGGCTTGATGTTTCTTCGGTAGCGCACCCACAGTCTAATGGTCAGGCAGAACGAGCGAACCAAGAGATCTTGAGAGGTATCAAACCCCGGCTTCTAGTCCCTTTGCAAAGGACGCCGGGTTGTTGGGTGGAGGAGCTACCGTCTGTATTATGGAGCATCAACACCACGCCTAACAGATCCACGGGGTTTACACCGTTTTTTATGGTTTATGGAGCAGAGGCGGTTCTCCCAAGTGATATACGACATGACTCACCTCGCGTGGCAGCATATGTTGAAGCGGATAATGAGCAGGCACGGCAGAACGCTCTTGACTTGTTGGATGAGGAGCGTGACTTGGCAGCTGCCCGTTCAGCGATTTATCAGCAAGATCTTCGCCGTTACCACAGCCGTCGGGTTAGGACCAGAACCTTTCAGGAGGGGGATTTGGTGCTTCGGCTCATCCAGGATCAGTCTGATCAGCATAAATTATCCCCGCCTTGGGAAGGACCTTTTGTGGTCAGCAAGAATCTGAATAATGGGTCATATTACCTGATTGATATTCGGGAGCGCAAGGAGTCACGCACATCAGAGGAGGAGACCAGCAGGCCGTGGAACATAGCTCATCTACGGCCTTATTATACATGAGCCCTGGGCTCTGCTTATGTACATATCATGACCATGTATATATTATGATTAATACAATAAACCGGAGCCTCGAGTAAAGCGGGGTTTCTGCTGTTCTTCACATCATGTGTGTCTTTTCCCCTGGAGGTTGCTTCACAAAGCTCAAGATATAGATTTCGTTTTAAAAGCCGGTTCAAGGGAAGATGTCTCCGACCAGGCGGTGCAGCTCTTAATATCCGGTTCAACATTCCGGTTCAAACCTGAGGCTCCCTGTTCAAACATAGGTCGTATTCGAACCAAAGAGAACATAGCTATTCAAATATGAGGCTTCCTGTTCAAACATAGGTCGTATTCGAACCAAAGAGGACATAGCTATTCAAACATGGGGCTTCCTGTTCAAACATAGGTCGTATTCGAACCAAAGAGAACACAGCTGTTCAAACATAGGTCGTATTCGAACCAAAGAGAACATGGCTATCCCTATAAAGTCAGTTGGGGACCTAGTCGGCCTGAACCGTGGCTACACCTTCGGGGGGCTTGGTCATGTCTTGACGACGGTAATGCCTTCTGGCTGGCACATTTGCCACTGGATCAGTTGGGGACCTAATCGACTTAAACCGTAGATTACACCTTAGGGGAACTCGGTCATGTCTTGATAATGGTAATGCCTTCTGGTCGGCTTTTTAGCCAATGAATCATTTGGGGGCTTGGTCGTGTCCGAACCAACGCAATGCCACTTGATCGGCCCTTGGCCATTGAGTCACTTGGGGGCTTGGTTGACGAACCAATGCAATGCCATTTGGCCGGCATATTTGCCACGATTCAGTTAGGAACCTAGTCGGCTTAAACCGTAGCTACAGCTCTTGGGGGCTCGGTTTTGTCTTGACGATGGTCACGCCGACTGAACGGTTCATAATAACCACTATTCGCATGCGTTTATTATCGCTTTGCCTTTCGTATTTGGTTTGGTTTTTTCTGCCTTGATTGTGGTGTTTTTTTTTAGTTCTTCGGAACATCAAGCATCCATAAACCGATCCAGCTGGCGTAGCCTGGTCCGTTTTTTAACCCGGAGGCAAATTGCCCGGTTTGGCAAAGGCGCAGCACGGTTCAGGTAGTCGAAAAATAGCAATGTGTACTCAGTAAAGGCAGAAAACATAAGATGGAAACATCATTGGAAAGAGCATCAGTACCCGTGCGCGAAGGCACATTCAAATGCAGAAGTATTAAACTATCTTATTACAAGGCAACGTAGTGCCTGAATATATTGTTCGCTGACGGATTATAGTTTCGCCAAAGGTGGGCCGGAGCACTGATTACGCCTGTTCGCCGCCTCGGTTCGAAGGCTGGGGATCATCTTGCGCCGCTTCAGCTCCATCTTGGTTACCCATGGGCTGGAAATCAGCAGTGGTCCAGTCGATTCCCATTAGCGCTTGAAAGATGGCTTCTTCATGAATCAGCGAAGACGGTTCAATGTCTGGGGCGTAAGTGTGCTTACGGATTGGAGGAACAAGGTTTCCCGCTTCAGGGACCGGGGCAGATACTCGTTTGTTCTGGTTGTCGTATTGGGCTTGGTAACGTGACAGATCTGCTTCCTCAGCCAATTGACAAGCTAGGGGGCGCACCGCCCGGTTTATCGCCCTCAAATCGTCTTCACCAAAGTCTGAACCGTCCTCCTTCAAGCTGGGGTATCCTTGGGCTGCTTCGACAGGATCGAAATCTGGTACCCACGCTTTGGCCCGGATCAAGGCAGTAATGGCTCCAGTTCGAGCAGCTGATCTTTTCAATTCTTCAATCCGGGCTGGAAGCATGGACAACCTCTTTAATGTGTCTTGAATCAGAGACGGCGCAGGGTTGTTATGCGATGCAGTGGTGATAGCTCTTTGCGCTCCGGTGTATAACTGTTCAATCAGAGTATAAGCAGCTTTGAGTTTCATCCGCACGTCAGAACCCAAGTGTCCAATACGTGAGCCTGCAGTGTTTCAAGGTGGATTACAAACCGGACATAGGGTGTGGCAAACAAGGTACAAATTGGAAGAAGGGCATCGAAAAAGTTTACCAAAAACGGCAGAGGTCATGGAGTGTATTTGTCGCTTCACGGTGGATAACTCATCAACCACCGGTTTAAGAGCAGCTTCGGCATTTTCAGCCCTCTTCGTCAAAAGAGCTTTTTCAGTAGCCCAATCGGCTTGCTCCTTTTTGCGGCTTGCTTTCAGCTGCTCCATAACAGTTAAGGCTTGGGTCAATTCCTCCTTTGCTTTTTCAGTTTCAATCTGCTGGGCTTTTAAAGCTTCCTGAAGGTCCGAGACTTGGTTCTCTTTGGTCTTCAAATCCCCCTGCAGCAGGTCATCAACAGTGTTCATAAAATATTGCACGGTTTGGAAGTATCAACCACACAAGCAAGTTATGTGCTCGATACTTGGGGGCTAATGCATATTTGATTTTAACATAAGTTTTCCATTACAAAAAAGTCCCAAGATTAATACAAGTATTTAAGCTTGGCACTTGGGGGCTAATGGTTATCTGTTGATGTTTTTTCTGGTTATGACCGAGGTTGAAACCTTGGGTTTTTTAAACCAGACCTTAATTGTCAAAAGCAGCCAGGTCCGGGCTATCATCTCGGTTTGGAAGTATTTGATAAGCTAAAGTTGCAATAGAATGAAGTACAAGGGAGTTACCTCAAGTTTCTCCCTCATCATCTTGATCATAGCAGCCTCATAATCCCGACTGGTGTACAGCCGGTTCAGATACCCAGAATAAAGATCCGGGGCGCTTAAAGAGGCGTACGCAGATAAGTCAAGGTCCCATTTGCCCTTTATGGCCGAGACTTCGTCCTTGGCAGTATGCTTGGCCAAAGTAGCAGTGTTCCCCGGTTCAGAGCGGCCAATACCGGTGATCACGACATCATCATCCTTGGAGTCGCTGCTCTGCAGTGGCCCTGTCGGTTTATCAGCAGTTGCAGAAGGGGGGATGGATTCCTCAACCCGAACAGGACTGGCAGACGGATCAGTACGGCTAGCAGGTTCGGTAGTCCTTTCTTCAGCGCAGATGTCATCTTGCGGAGATGGATAGTTCTGAGTATCTTCACGATCCAGAGCGTCAGCACCAGGAGTTTTCTCCGGTTCAGGAGCGGCAGTTCCTTCAGCCGGGTTATCCAGACGAGCCTTCTTGCTCGGTCTGGGTTTAGCCCTGAAAAAACCCAAAGATTGGTAAAACAGTGTAAGATGGATTAAGAGACCCATACAGAGATATGTTGTGATCGATCCAACTTACCCAATAACTGTCTTCAACGGTGGCAGTTGGGTAGCTGAGGATTCACCAGAAGATGAATGGGAAGTGACCTGATAATCGGAGTCAGATGGATTTAGAGGCTGGCGAGTTAAACCGGCTTTAGGAGGAAAGCTAGCGCATAAATCGGAGACCTCTGAACGGCGTTTCCGAACCGGGCTGTTCGGTAAACCGGCGGAGGTGACTACCTGGCCGCTATGACGGGTGGTGCGTCGAGCTTCATGTTGCTGGGTCTTCACAAGAAATTTGGGATCCAAGTAAGCAAGAGGGTGAGAAAATTTAATTTTCCGATTTGCCTGGCGAGGTTTTTTCAAAGGCAAAGGAACGGCATCAGAAGAAAGAATAGTTACCTCTGGGCCTTCGGCATGGCTCACTTCAGCATCATCCTGACAAATATCATCATCAATAAGACGTACAAAAAGCAAACCAACTGAATCGAGGTCTACCCCGAGGTCCGGATTACCCACATCATCCTCCTCTAAATCCAGAGTATTGGAAGCGGTGTTCCTTTTCCTCTGAGCAGGCTTCTTCACCCTCGTCTTGGGTCGGGTTACCTTTTCGGTCGGCTCTTGTAACTTCTTGCTCCAAAATGGATCATCTCCCTGTCTCAGGAAGGACTGGAGTTAACAACATATATATAAGGAGATCAAAGATTCTATAAACAGAGGAATTACTTACAGCAGGTGGTTTGTTTGTGGCACAAAATGGAAGCAGGCCGGTTTGAGCGCAAACAGGTTCCGGTTCGTTCAGCATCTTCTTCACAGCTTCAGCGACTTCGTCCTCCGTCAGCTGAATGTTGCAGTGCCTCAGAGGGTCATCAACCCGACCCGTGTATTGGCACATCAAACCGGAGCGGATGCTAAGGGGCAGGATGCTCCAGGAAATCCAGCAACGAGCAAGATCAACCCCCGTTAAACCATTAGCCATGAAGGCTCTGAGCTTGGACAGCTGAGGGGCATACTTACTCCTCTCTTTGGCAGTTAGCCGTGGGGGGAAGGGGTGAGTGTTGCTAAGTCTCTCTGGACGGAAACCGGGCAGTGGATTTTCATCGGCAGGGGAAGTATCCTTGCAGTAGAACCAAGTACAATTCCATTCTTGCGGATGACTATGCAACTTGGCGTGAGGATAAGTCACCTCTTTCCTTTTCTGAATCGCCATACCACCCAGTTCCATGTTAGGGCCGTCGGTAAATTCAGTACGTCGGTTCAAATGGAAAAAGTCCCGGAACAGCTCTGCAGTAGGTTCTTCTTGGAAGTACGCCTCGCAAAGTACTTGAAAATGGCAGATATTCGTGACTGAATTTGGACCAACATCTTGTGGGCGCAGCCTAAAATTAGCCAAGACATCCCGATAAAATTTTGAACCGGGTGGATTAAACCCCCGGGCCAGGTGATCCGCAAACACCACCACTTCTCCTTCTTGCGGGGTAGGAGGACATTCGTCTCCTGGAACCCTCCAATGGATGATACTTTTACTACTCAAAGCACCAGTCAGAACTAAGTTGTTTAGATGGGTCTCAGTGATGCAAGAGGGAACCCAGTTGCATTCGTACACCTGTTTTGCCATGAGGAAATCTACAAGGCAAAAGTATTCCGGTTTGAGAGCAAGTATGATTGTAAACCGAAGATGTTCTTATATAGAGTCAGGTTATCTACAATAATGTCACCGGCGGTTCATCAAGGGGACTAATGGATGTGTAGATCGGTTTATTCGCTTTGCTAAGAATTAAACCGACCAGATCTAAGCAGTTGTGAGTATGAAAGCAGAAACAGATCCCGTACGAACTCGTTCAAAGCAATGGATCTACAGCGAGGGTGAAAAACAAATAAAAACAGGTAATATGGAGTCGGGCTTACAGCAGAGCAACATAAGTTCGACAAGCAGGAACAAGAGTGAGATCTAAGGAAATGAATGCAGATTTATGGCGACTGCTAAAAGAAACTACAAATCCAGGTGCAGGTATCGTCATCTGTTCGTAAAGGCTGCTACAGGGATGAGCGAGAAACAGTAGCAGGACGATGAACCCTAGAACAGATCTAAAGCAACAAGAACAGGGATCTTACCGGGGTCCAAACAGATGCGGAAGGTCGCCGCGGTGCTCTGAACCGATCAGGTTGATGCAGCGGCCGGAGTTGATGCAGCGGTTGACGGCGGCGGCGGAGCTCTGAGGTCTGGGTCGCGAGGAAGACGAAGAAGGGGAGGAAAGGAAGATGGCCTTCAAGTCCTATTTATAAGGCGCGGCGCGTGTATCAGGCGCGCGAATCCAGGAGCCGGAGATTTCGGAAGCCCAGCCGTCGCCTTGATTCTTGCGGGTCAGCGAAAGGAGACGGTAGCCTGCGTTGACGTCACATCGGTTTACCGCAGCAGAGGAGACGGAGAAACGACGGTTTAACAAGTCACTGGAGGACATTTGAAGACAGAAATTTTTATTAAGGATTGACATGAACCTGTTCAAATCAATCTGGGGCCTAA

TGTTGGGGATGTTGCTATTCGGGGTAAACCGGCCTGCAGGGCCCGGGTTACCTCCGTCCACAGACCCGAGATGTTAAAGCCCAAGGAGAGATAGAAGGGCCTAAGGCCCATAATCGGTTTAAAGGTTTACGGTATAAACCGGCCTGATGTAAACTTGTATTGTAAGTTAGGAATAGAGAGACCGACCCGGGCACGTTTATAAGCCGGAGGTTGGGACTTTGCAGACCGACGGGCGTCACCCGTGTATATAAGGGGATGACCCGGCGGCGGTTTGGGGACAGAGAAGAAGAATTCGAGGTTTAGGCGAAGCATATCGCTCCTTAAGCCCTCGAAACCCATCAATCCCATCACAACTAGACGTAGGCTTTTACCTTCATCGAAGGGGCCGAACTAGTATAAAAACCGTGTCCTCGCGTCCACTTTAACCCCTTCAAGCTAACCCGTTGCGATGGCTCCACGACTAAGTCCTCTTCCTAGGACATCTGCCGTGACAAATCCACGACA

TCATCCATGATATATGAGCTTGTGCATTTTCTTAGCAAATTTGTTGTGATCTTTCTTGATGACATATTCATAACTCGTGATCATATTGCCTACCATGAATTGCATGATAACATAATCATATCGA

>Aegilops umbellulata 1-189;

CACTGATACTGAACTGCATGGACGAATCGCCAGGTACGAAGTCAACAAGCTTAGTCCCAGCGGCTGACATAAACTTCAGAGCCGGATCATGCTCCGTAGTAGGCTTCTTTAAAGGAGTCATATC

TGTCGTGGATTTGTCACGGCAGATGTCCAAGTAAGAGGACTTAGTCGTGGAGCCATCGCAACGGGTTAGCTTGAAGGGGTTAAAGTGGACACAAGGACACGGTTTTTATACTAGTTCGGCCCCTTCGATGAAGGTAAAAGCCTACGTCTAGTTGTGATGGGATTGATGGGTTTCGAGGGCTTAGGGAGCGATAAGCTTCGCCTAAACCTCGAGTTCTTCTTCTCTGTCCTAAACCGCCGTCGGGTCGTCCCCTTATATACACGGGTGACGCCCGTCGGTCTGCAAAATCCCAACCTCCGGCTTATAAACGTGCCCGGGTCGGTCTCTCTATTCCTAACTTACAGTACAAGTTGGCCCCAGGCCGGTTTACACCATAAACCTTTAAACCGGTTATAGGCCTTGGGCCTTTCTATTTCTCCTTGGGCTTTAACATCTCGGGTCTGTTTGACGGCGGTAACCCGGGCCCTGCAGGCCGGTTTACCCCAGATAGCGACATCCCCAACA

TTAGGCCCCAGATTGATTTGAACAGGTTCATGTCAATCCTTAATAAGAATTTCTGTCCTCAAATGTCTTCCAGTAACTTGTTAAACCGCTGTCTCTCCGTCTCCTCTTGCTATGGTAAACCGATATGACGTCAGCGCAAGCTACCGTCTCCTTTCGCTAACCCGCAAGAATCAGGGCGACGGCTGGGCTTCCGAAATCTCCGGCCCCTGGATTCGCGCGCCTGACACGCGCGCCGCGCCTTATAAATAGGACTTGAAGGCCATCTTCTTTTCCTCCCCTTCTTCGTCTTCCTCGCGACCCAGACCTCAGAGCTCCGCCGCCGCCGTCAGCTGCTGCATCAACTCCGGCCGCTGCATCAACCTGATCGGATCAGAGCACCGCGGCGACTTTCCACATCTGTTTGGACCCCGGTAAAGCCCCTGTTCTTGTTGCTTTGGATCTGTTCTAGGGTTCGTCGTCCTGCTACTGTTTCTTGCTCATCCCCATAGCAGCCTTTGCGAATAGATGATGATACCTGTACCTGGATTTGTAGTTCCTCTCGGTATTCGCCATAAATCTGCATTCGTCTTCTTAGACCTCGCTCATTTCCTGCTTGTCGAACTTATGTTGTCCTGCTGTGAGCCAAACTTCGTATTTTTATTTCTTTTTCGCCCTAGCTGTAGATCCATTGCTTGAAAGAATCCGTATGGGATCTGTTTCTGCTTTCATACTCACAGCTGTTAGATCTGGTCGGTTTAACTCTTAGCAAAGTGAACAAAACAATCCACACATCCATTAGTCCCCTTGATGAACCGCTGGTTGATATCATCGTGGATAACCTGACTCTATATAAGATCACCTTCGGTTTATAATCACACTTGTTCTCAAACCGGAATACCTTTATCTTGTAGATTTCCTCATGGCAAAACAGGTGTACGAATGTAACTGGGTTCCCTCTTGCATCACTGAGACCCAACTAAACAACTTAGTTCTGACTGGTGCTTTGAGTAGCAAAAATACCATCCATTGGAGGGTTCCAGGAGACGAATGTCCTCCTACCCCGCAGGAGGGAGAAGTGGTGGTGTTTGCGGATCACCTGGCCCGGGGGTTTAACCCACCCGGTTCAAAATTTTATCGGGACGTCTTGGCTAATTTTAGGTTGCGCCCACAAGATGTTGGTCCAAATTCAGTCACGAACATCTGCCATTTTCAAGTACTTTGCGAGGCGTACTTTCAAGAGGAACCCACTGTAGAGCTGTTCCGGGACTTTTTCCATCTGAACCGACGTACTGAATTCACCGATGGCCCTAATATGGAACTGGGTGGTATGGCGATTCAGAAGAGGAAAGAGGTGACTTATCCTCACGCCAAGTTGCATAGTCATCCGCAAGAATGGAATTGCACTTGGTTCTATTGCAAGGATACTTCCCCTGCTGAAGAAAATCCACTGCCCGGTTTCCGTCCAGAGAGACTTAGCAACACTCACCCCTTCCCCCCACGGCTAACTGCCAAAGAGAGGAGTAAGTATGCCCCTCAGCTATCCAAGCTCAGAGCCTTCATGGCTAATGGTTTAACGGGGGTTGATCTTGCTCGTTGCTGGATTTCATGGAGCATCCTGCCCCTTAGCATCCGCTCCGGTTTGATGTGCCAATACACGGGTCGGGTTGATGATCCTTTGAGGCACTGCAACATTCAGCTGACGGAGGACGAAGTCGCTGAAGCTGTGAAGAAGATGCTGAACGAACCGGAACCTGTCTGCGCTCGAACCGGCTTGCTTCCATTTTGTGCCACAAACAAACCACCTGCTGTAAGTAACTCCTCTGTTTATAGAATCTTTGATCTCCTTATATATACGTTGTTAACTTCCAGTCCTTTTCGAAACAGGGAGATGATCCGTTTTGGAGCAAGAAGCTTCAGGAACCAACCGAAAAGATAATCAGACCCAAGACAAGGGTGAAGAAGCCTGCTACGAAGAAAAGGAACACTGCTTCCTGTACCCCCGATTTAGAGGAGGATGATGTGGGTAATCCGGACCTTGGGGTAGACCTCGATTCAGTTGGCTTACTTTTTGTACGTCTTATTGATGCTGATATTTGTCAGGACGATGCTGAAGCGAGCAATGCTGAAGGCCCAGAGGTAACTATTCTTTCTTCTGATGCTGTTCCTTTGCCTTTGAAAAAACCTCGCCAAGCAAACCGGAAAATTAAATTTTCTCACCCTCTTGCTTATTTGGATCCCAAATTTCTTGTGAAGACCCAGCAACATGAGGCTCGACGCACCACTCGTCATAGCGGTCAGGTAGTCACCTCCGCCGGTTTACCGAACAGCCCGGTTCGGAAACGCCGCTCAGAGGTCCCCAATTTATGCGCTAGCTTTCCTCCTAAAGCAGGTTTAACTCGTCAGCCTCTAAATCCATCTGACTCCGATTATCAGGTCACTTCCCATTCATCTTCCGGCGAATCTTCAGCCACTCAACTGCCACCGTTGAAGACAGTCATTGGGTAAGTTGGACCGATCACAACATATCTCTGTATGGGTCTCTTAATCCATCTTATACTGTTTTTTCAATCTTTGGGCTTTTTTCAGGGCTAAACCTAGACCGAGCAAGAAGGCTCGTTTGGATAACCCGGCTGAAGGGACTGCCGCTCCTGAACCGGAGAAAACTCCTGGTGCTGACACTCTGGGTCGTGAGGATGCTCAGAATTATCTATCTCCGCAAGATGACACCTGCGCTGAAGAAAGGACTATCGAACCTGCCAGCCCTGCCAGCCCTGTTCGGGTTGAGGAATCCATCCCCCCTTCTCGAACTGTTGATAAACCGACAGGGCCCCTGCAGAGCAGCGATTCCAAGGATGATGATGTTGTGATTACCGGCATCGGTCACTCTGAACCGGGGAACACTGCTACCTTAGCCAAGCATACTGCCAAGGACGAAATCTCGGCCATGAAGGGCAAATGGGACCTTGACTCTTCTACGTACGCCGCTTTAAGCGCCCCGGATCTTTATTCCGGGTACCTGAACCGACTGTACACCAGTCGGGATTATGAAGCTGGCATGATCAAGATGATGAAGGAGAAACTTGAGGTAACTCCCTTGTGCTTCATTCCATTGCAACTTAGCTTATCAATATTTCCAAACCGAGATGGTAAACCGGATCAGGCTGTCTTGACAACTAAAGTCCGGCTTAGAAAACCCAAGGCTTCAACATAGGCCATAACCAGAAAACATCATTAGATAACTATTAGCCCCCAAGTGCCAAGTTTAAATACTTGTATTAATATTGGGACTTTTTGCAATGGGAAACTTGCGTTAAAATCAAATATGCATTAGCCCCCAAGTATCGAGCACATAACTTGCTTGTGTGGTTGATACTTCCAAACCGTGCAATATTTTCTGAACACTGTTTTGACGACCTGCTGCAGGGGGATCTAAAGACCAAAGCGAAACAAGTCTCGGACCTTCAGGAAATCTTAAAAACCCAACAGGCTGAAACTGAAAAAACGAAGGACGAATTGGCCCATGCTTTAACCATCATGGAACAGCTGAAAGAAAGCCACCAGAAAGAACAAGCTGATTGGGTTACTGAGAGAGCTCTTTTGACGAAGAGGGCTGAAAATGCCGAAGCTGCTCTTAAACCGGTGGTCGATGAATTATCCACCGTGAAGCGACAGATACACTCCATGACCTCTGCTGTCTTTGGTAAGCTTTTCGACGCCCTTCTTCCAATTTGTACCTTGTCTGTTACATCCTATGTCCGGTTTGTAATCCATCTTGAAACACTGCAGGCTCACGTATTGGACACTTGGGTTCTGATGTGCGGATGAAACTCAAAGCTGCTTATACTCTGATTGAACAGTTGTACACCGGAGCACAAAGGGCTATCACCACTGCGTCGCATAACAACCCTGCGCCGTCCCTGATTCAAGACACATTAAAGAGGCTGTCCATGCTTCCAGCCCGGATTGAAGAATTGAAAAGATCAGCTGCTCGAACTGGAGCCATTACTGCCTTGATCCGGGCCAAAGCATGGGTACCAGATTTCGATCCTGTCGAAGCGGCCCAAGGATACCCCAGCTTGAAGGAAGACGGTTCAGATTTTGGTGAAGACGATTTGAGGGCGATAAACCGGGCGGTGCGCCCCCTAGCTTGTCAATTGGCTGAGGAAGCAGATCTGTCACGTTACCAAGCCCAATACGACAACCAGAACAAACGAGTGGCTGCTCCGGTCCCTGAAGCGGGAAACCTTATTCCTCCAATCCGTAAGCATACTTACGCCCCTGACATTGAACCGTCTTTGCTGATTCACGAAGAGGCCGTCTTTCAAGCGCTAATGGGAATCGACTGGACCACTGCTGATTTCCAGCCAATGGGTAACCAAGATAGAGCTGAAGCGGCGCAAGACGATCCCCAGCCTTCGAACCGAGGCGGCGAACAGGCCTAATCGACGCTCCGGTTCACCTTACTTGGTGGCGTTTTAATCCGTCAACGAACAATATATTCAGGCACTACGTTTGCCTTGTAATAAGATAGTTTAATACCTCTGCATTTGAATGTGCCTTCGCGCACGGGTACTGATGCTCTTTCCAATGACGTTTTCCAACTTTGTTTCCTGTCTTTACTGAGTACACAATGTGATTTTTCGACCACCTGAACCGTGCTGCGCCTTTGCCAAACCGGGCAATTTGCCTCCGGGTTAAAAAACGGACCAGGCTATGCCAGCTGAACCGGTTTTAAGAATACTTGACGTTCCGAAGAACCAAAAAACACCACAATAAAGGCAGAAAACAAAACCAAATATGAAAAGGCAACGCGATAGCAAAACGCATGCGAATAGTGGTTACTATGAACCGTTCAGAAGGCGTGACCATCGTCAAGACAAAACCAAGCCCCCAAGAGGTGTAGCCACGGTTTAGGTCGACTAGGTTCCTAACTGAATCGTGGCAAATATGCCGGCCAAATGGCATTGCATTGGTTCGTCAACCAAGCCCCCAAGTGACTCAATGGCCAAGGGCCGATCAAGTGGCATTGCGTTGGTTCGGACACGACCAAGCCCCCAAATGATTCATTGGCTAAAAAGCCGACCAGAAGGCATTACCATCATCAAGACATGACCGAGTTCCCATAAGGCGTAATCTACGGTTCAAGTCGATTAGGTCCCCAACTGATTCAGTGGCAAATGTGCCAGCCAGAAGGCATTACCGTCGTCAAGACATGACCAAGCCCCCCGGAGGTGTAGCTACGGTTCAGGCCGACCAGGTCCCCAACTGACTTTATAGGGATAGCTATGTTCTCTTTGGTTCGAATACGACCTATGTTTGAACAGGAAGCCCCGTGTTTGAATAGCTATGTTCTCTTTGGTTCGAATACGACCTATGTTTGAACAGGAAGCCTCGTGCTTGAATAGCTATGTTCTCTTTGGTTCGAATACGACCTATGTTTGAACAGGAAGCCTCGTGCTTGAATAGCTATGTTCTCTTTGGTTCGAATACGACCCATGTTTGAACAGGAAGCCTCATGCTTGAACCGGGATATTGAACCGGATATTAATAGCTTCACAGCCTTGTAGGAGACATCTTCCCTTGAAACGGCTTTTAAACCGGAATCTATAATTTGAACTTCTGTGAAGCGACCTCCAGGGGTAAGCACACATGATGTGAAGAACAATAGAGACCCCGCTTTACTCGAGGCTCCGGTTTATTGTATTTAATCATAATATATACATGGTCATGATATGTACATAAACAGAGCCCAGGGCTCATGTATAATAAGGTCGAAGATGAGCTATGTTCCACGGCCTGCTGGTCTCCTCCTCTGACGTGCGTGAATCCTTGCGCTCTCGAATATCAATCAGGTAATATGACCCATTATTCAGATTCTTGCTGACCACAAAGGGTCCCTCCCAAGGCGGGGATAATTTATGCTGATCAGATTGATCCTGGATGAGCCGAAGCACCAAATCCCCCTCCTGAAAGGTTCTGGTCCTAACCCGACGGCTGTGGTAGCGGCGAAGATCTTGCTGGTAAATCGCTGAACGGGCAGCTGCCAAGTCACGCTCCTCATCCAACAAGTCAAGGGCGTTCTGCCGTGTCTGCTCATTATCCGCTTCAACATATGCTGCCACGCGAGGTGAGTCATGTCGTATATCACTTGGGAGAACCGCCTCTGCTCCATAAACCATAAAAAACGGCGTAAACCCCGTGGATCTGTTAGGCGTGGTGTTGATGCTCCATAATACAGACGGTAGCTCCTCCACCCAACAACCCAGCGTCCTTTGCAAAGGGACCAGAAGCCGGGGTTTGATACCTCTCAAGATCTCTTGGTTCGCTCGTTCTGCCTGACCATTAGACTGTGGGTGCGCTACCGAAGAAACATCAAGCCGTATATGCTCCCGTGCACAGAACTCTTTCATGGCCCCTTTAGATAAATTGGTACCATTATCTGTGATGATACTGTGTGGAAAACCAAACCGGAAGATCACCTTTTTTATAAACCGAACTGCCGTGGCCGCATCACACTTACTGACAGGTTCTGCCTCTACCCACTTTGTAAATTTGTCAACCGCCACCAGTAGGTGGGTCTTCTTATCTTTGGAACGCTTAAAAGGCCCAACCATATCAAGCCCCCAAGTCGCAAACGGCCAAGTGATTGGAATCATCCTCAATTCTTGAGCCGGTATGTGTGCTCGTCTGGAAAATTTCTGGCAACCATCACATAATCTGACCAGGTCTTCCGCATCAGCATGAGCAGTTAACCAGTAGAAACCGTGGCGGAACGCTTTAGCCACCAATGATTTTGAACCGGCGTGGTGGCCGCAATCCCCTTCATGTATTTCTCGTAATATCTCACACCCCTCCTCAGGGGACACGCACCGTTGCAGCGCTCCTGATACGCTGCGATGATGTAGCTCTCCTTTGAAGATGGTCATAGACTTGGACCGGCGTACTATCTGTCGAGCCAAGCTTTCGTCCTCTGGCAACTCACCCCGGTTCATATATGCCAGATACGGGAGCGTCCAATCCGGGGTGGCGTGGAGAGCCGCCACTAACTGAGCCTCCGGATCAGGAACAGCCAACTCCAGCTCACCGGGCAGCTGTACCGATGGGTGGTACAATACATCCAAGGAAACATTGGGTGGGACCGGTTTACGCTGAGAGCCCAAGCGACTTAAAGCGTCCGCTGCCTCATTCTTTCTGCGGTCCACATGATCCACTTGATAGCCTTTGAAATGCCCAGCCACCATATCTACTTCACGTCGGTATGCAGCCATAAGCGGATCCTTAGAATCCCAGGTGCCAGATACCTGCTGAGCCACCAGATCCGAATCACCAAAGCACCTAACTCTGCTCAGATTCATCTCTTTAGCCACCCGAAGACCATGAAGCAAAGCTTCATACTCCGCCGCATTATTTGTACAGGGGAACATTAAACGGAGGACATAACAAAACTTATCTCCTCGTGGGGAAGTTAAAACGACTCCAGCCCCCGAGCCTTCCAATTGTCTTGACCCGTCAAAATGAACGGTCCAATAGGTGTTATCTGGCTTCTCCTCAGGCGCTTGTAACTCTGTCCAATCGTTTATGAAATCGACAAGTGCCTGTGATTTTATGGCCGTTCGGGGTACGTACCTTAAATCGTGCGGCCCGAGCTCGATAGCCCACTTGGCAATCCGACCGGTCGCCTCCCGGTTCTGTATAATGTCACCCAAAGGAGCAGAACTGACCACCGTGATTGGGTGTCCTTGGAAATATTGCCTCAGCTTCCGGCTTGCCATAAAAACGCCATACACCAGCTTCTGCCAATGCGGATACCTTTGTTTGGACTCGATAAGTACTTCACTGATATAGTAAACCGGTCGCTGAACCAGATGTTCCTTTCCAGCCTCCATTCGTTCTACCACCATCGCCACACTGACCGCTCTGGCATTCGCTGCAACATATAACAGGAGTGGCTCTTTATCTACAGGGGCTGCTAACACTGGCGGATTGACTAGCTGTCGCTTCAAGTCCTCAAACGCTTTATCAGCCTCCGGACTCCAGACAAATTGATCCGTCTTCTTGAGCATTTGATACAAGGGGATCGCCTTTTCACCGAGGCGACTGATGAACCGGCTTAACGCTGCAATCCGCCCGGCCATGCGCTGAACATCGTTGATGCATTTCGGTTTGGCCAGGGAGGTGATGGCTGTGATCTTTTCCGGATTAGCCTCAATTCCTCTATGGGACACTAAAAATCCCAACAACTTGCCTGCAGGAACACCAAAAACACATTTAGCTGGGTTGAGCATCATTTGGTACACTCTCAAGTTATCGAAAGTCTCCTTCAAATCGTCAACCAGGGTCTCCTTCTTTCGTGACTTGACCACAATATCATCCACATAAGCATGCACATTACGACCAATCTGCTCGTGGAGGCAATTTTGCACACATCGTTGATAAGTAGCCTGTGCACTCTTAAGCCCAAAGGGCATAGACACATAGCAGAAGGCTCCAAAGGGGGTAATGAACGCTGTCTTCTCCTGGTCCTTAACTGCCATCTTGATCTGATGATAACCCGAATAGGCATCCAAAAAACTTAAACGCTCGCAACCCGCCGTAGCATCAATGATCTGATCAATCCGGGGGAGAGCAAAAGGATCTGCTGGGCAAGCTTTATTCAAGTCTGTGTAGTCCACACACATGCGCCAGGTGCCGTTTTTCTTAAGAACTAGCACCGGATTAGCGAGCCATTCGGGATGAAAAACCTCAATGATAAACCCAGCTGCCAGGAGCCTGGCCACTTCCTCACCAATCGCTTTACGTCTTTCTTCGTTAAACCGCCGTAAAAACTGCTTTACCGGCTTGTACTTAGGATCCACATTAAGGGTGTGCTCAGCGAGTCGCCTCGGTACACCAGGCATGTCAGACGGCTTCCATGCAAAGATGTCCCGATTCTCACGGATGAACTCGATGAGCGCGCTTTCCTATTTTGGATCCAAGTTTGCACTGATGCTGAACTGCTTGGATGAATCGCCAGGTACGAAGTCAACAAGCTTAGTTTCGTCCGCTGACTTGAACTTCAAGGCCGGATCGTGGTCCGTAGTTGGCTTTTTTAATGAAGTCATGTCCTCCGGATCAACACTGTCTTTGTACTGCTTTAGCTCCTCCGTAGCACAAACCGATTCTGCATAAGTCGCATCTCCTTCCTCGCATTCCAAAGCGATTCGACGGCTCCCATGAACCGTTATAGTGCCCTTGTAACCTGGCATCTTAAGTTGCAGATACACATAGCAGGGCCGTGCCATAAATTTTGCATAAGCTGGCCGTCCGAACAAAGCGTGGTACGGACTTTGGATTTTAACCACCTCAAACGTCAGCGTCTCTGATCTGGAATCGTGACTATCGCCAAATACCACTTCAAGGGCTATCTTACCAACCGGATATGCAGATTTGCCAGGCACCACCCCGTGGAACACTGTATTGGTCGGTTTGAGATCCTTATCTACCAGTCCCATACGACGGAAGGTTTCGTAATACAAGATGTTAATGCTGCTCCCTCCGTCCATGAGCACCTTAGTGAGCTTATAACCTCCCACCTGAGGCGCCACCACCAACGCTAACTGACCCGGATTATAAACCCTGGGAGGGTGATCCTCTCGGCTCCATATGATAGGCTGTTCTGACCAGCGCAGATAGTGAGGCGTGGCCGGTTCAATAGCACTGACTGCTCGCCTCTGAAGCTTTCTATCCCGTTTATCCAAGCTTGTAGTAAAAACATGATACTGTCCACCGCTCAACTGTTTCGGGTTGCTCTGATAACCACCTTGATTACTTTGATTATTTTGATTATTTTGGCTGACCTGTCCGCCCGGATTACCCTGGAACCCTGGACCGGAATTTCCGCCACCGTACCCTGATCCGGAACCAGAACCGCCGCCGGAGCTGTGATCATATTGGAAAGTGTTTGAGCTTTTGAATTCCTTCATGATATAACAATCCTTCCAAAGATGGGCTGCTGGTTCCTCCTTTGTTCCGTGCTTCGGACAGGGCTGGTTTAAGAAAAAGTTCAAACGCTCCGGGTTAGGACCGGGATGTGCGCGAAAACTTGGCTGTTTCCCCTTACGCCGCTGGCCTTTATCCTGCGCACTTGTGCTGGCCACCAAGTCCATGCTCCCGTCCGGTTTACGCTTACCTCCGCCTCCATTACCTGCCGGCCGATGTTGCTGGCCTTTGGTGTTGCTATTCTTCCTTCCCTTCCCTGCTTTGTCATCACCAGAGTCAGGATCCTTGGTACTGTCAGAATCCGCATACTTCACTAAAGCGGTCATGAGCGTTCCTATATCTGTACAATCACGCTTCATCCGGCCCAGCTTTAGCTTCAAAGGGCCAAACCGACAGTTGCTTTCCAATAGCACTACGGCGGTGTCTGCGTTGATGCGATCTGATGAGTGCAAAACTTCTGATACTCGTTGTACCCAATGGGTAGTTGATTCCCCTTCCTGCTGAACGCAAGCAGCTAAGTCCACAATTGACATTGGCTGTTTGCACGTGTCCTTGAAGTTCTTGATAAACCGGGCGCGCAATTGGGCCCACGAGCTAATTGAATTAGGCAGCAAACTCTTTAGCCAAGTCCGGGCCGTTCCTTCTAACATCATAGTAAAATACTTGGCACACGCCGCGTCATCCACATCCAGCATCTCCATAGCCATTTCATAACTCTCCACCCACGTCTCCGGAGGCTGATCTGCCGTGTAGTTTGGTACTTTGCGGGGGCCTTTGAAGTCTTTGGGCAGGCGTACGTTGCGCAAGGTGGGAACAAGGCACGGTACTCCCAAAGAACTGGAAACCACCCCCGGCTCAGCCGCAGCTGCTGGGAAGATAGGCGTAAGCTGACGAGCCTGATGCTGCGCTGCCAATCCGGCCTCCCGTGCACGGGTGCGATCCGCATTCTCGTGAGCATTGTCGTCGCCCGCCGGATTGTTGCCGCGGAGCGCTTCACGCCTGGCGTTACTTGAAACGGCCGGCTCCTCCACGCGCCTACTATAGCTCCTGCTGGGGCGAGGGGTGGAGTGAATCCGATCACGGCTTTGCGAATATGCTTCTTGCTGGGCCAACGCGGTCCTAAGGAGCTCCTTGACCCGGCGTGTCTCTTCATCCTGCGGTGAATCTCCTTCGATCGGAATGGCTTCCAACCGAGCCGCCGCCGCGACGAGGTTATCCATCGGATTGGAATAATAACCCGACGGCGTTGGCATGGCCGGTGGCGTATCAGTGTTATGACGAGGCAGGTCTATAATCCGGTGCTGAACCGGGGCGCCGGCTGTCGCCTCCGCACGGTTTACCCCCGGGGTGTTGAAGAGATTTCGAGCGTCGAGGGCGGCGGGCAACCGAGACCGGGATTTTCTCTTCAGGACCTCCTGAGACGCCTTCTGGTCCATCATGAGCCTCTAAGCCTGTGCATCTAAAGCGGCGCGCTCCGCCGTCATCCTGATCTCTTCTGCAGCCAGCTCCGCTTTAGCCCGAGTGATCTGCTCTCTCACTTTGGCAATCTCAGCATTGTGAACCTCCTGGTCCACCGGGTTGGCCTCCGCCATGAGCGCTGCTAGTGCGTCAAACAGGTCTGACAAAACTTGGGCTGGTGAACCCGTAGGGCCTCCTGCCCGCGCAGCCGTCGCTGCTGCCGATCCGGAGATCGCTGCTGCTGCAGTTGATGATCGTGGCGCCGCCTGTGTTCCAGCCATGAAGATCCCAACCCGATAGGGTAGAACAGAGGGGTCCGGAATACTGTTGCCATCGGAGCCGCCCCCAACCCGATCACCTTGTAGCTGGTAAAGAGATTCGGTCTCCCCGGTCGACGTTTCATCGCCAGAGTAGATGGAGGTTTCGTCACCAGATTCGGATCCTTCCTCGTAGCTTCCCCCGTGGATGACTCCCACGAAGGCGTGCTTCCTGGTCGGTTTAGCCAGGGTCGATCTCGTGCACTGAGCTGTCTCGATGAGGTCGGCGCAGACGCCTAACTCGGGGCCCGGTTCTCCGATCTTGCCGATGAAAACATGGATGCCGCCAAAGGGGACCCGGTACCCGTACTCGATCGAGCCGGCGTCGGGGCCCCAGCCTGTGCTGTCGATGTAGAGTTTTCCGCGGCGGCTCTTGGTCATCCGTCCCACAGCGTAACCCTCGAGTCCTTCAAAGCGGCCCTCCAAGAACCTGAAACCATCTCGCGATAGCCCCACGGTGGGCGCCAAC

TGTCGTGGATTTGTCACGGCAGATGTCCAAGTAAGAGGACTTAGTCGTGGAGCCATCGCAACGGGTTAGCTTGAAGGGGTTAAAGTGGACACAAGGACACGGTTTTTATACTAGTTCGGCCCCTTCGATGAAGGTAAAAGCCTACGTCTAGTTGTGATGGGATTGATGGGTTTCGAGGGCTTAGGGAGCGATAAGCTTCGCCTAAACCTCGAGTTCTTCTTCTCTGTCCTAAACCGCCGTCGGGTCGTCCCCTTATATACACGGGTGACGCCCGTCGGTCTGCAAAATCCCAACCTCCGGCTTATAAACGTGCCCGGGTCGGTCTCTCTATTCCTAACTTACAGTACAAGTTGGCCCCAGGCCGGTTTACACCATAAACCTTTAAACCGGTTATAGGCCTTGGGCCTTTCTATTTCTCCTTGGGCTTTAACATCTCGGGTCTGTTTGACGGCGGTAACCCGGGCCCTGCAGGCCGGTTTACCCCAGATAGCGACATCCCCAACA

ATATCCGTCGGATCAACATTGTCTTTGTAATACTTGAGTTCCTCGGTACTACAAACTGACTCTGCGTAGGCCGCATCTCCTTCTTCCCATTCCAAAGCGACCTTGCGGCTTCCGTGAACCGTGA

>Aegilops umbellulata 1-197;

GTATGCTAGACAACTATGCTTGGAATATGATTATACTTGTTGCTCTAGGATGAAGGCTCCACACCTTCCCTTTTCATGCAAATTTAATGATAATAAAACCTTAGCTTCTTATGCTAATGGTATA

TGTCGTGGATTTGTCACGGCAGATGTCCTAGTGAGAGGACTTAGTCGTGGAGCCATCGCAACGGGTTAGCTTGAAGGGGTTAAAGGTGGACACAAGGACACGGTTTTTATACTAGTTCGGCCCCTTCGATGAAGGTAAAAGCCTACGTCTAGTTGTGATGGGATTGATGGGTTTCGAGGGCTTAAGGAGCGATAAGCTTCGCCTAAACCTCGAATTCTTCTTCTCTGTCCCCAAACCGCCGTCGGGTCGTCCCCTTATATACACGGGTGACACCCGTCGGTCTGCAAAGTCCCAACCTCCGGCTTATAAACGTGCCCGGGTCGGTCTCTCTATTCCTATCTTACAATACAAGTTGGCCCCAGGCCGGTTTACACCATAAACCTTTAAACCGGTTATAGGCCTTGGGCCTTCCTATTTCTCCTTGGGCTTTAACATCTCGGGTCTGTTGACGGCGGTAATCCGGGCCCTGCAGGCCGGTTTACCCCAGATAGCAACATCCCCAACA

TTAGGCCCCAGATTGATTTGAACAGGTTCATGTCAATCCTTAATAAGAATTTCTATCTTCAAATGTCTTCCAGTAACTTGTTAAACCGCTGTTTCTCCGTCTCCTCTTGCTATGGTAAACCGATATGACGTCAGCGCAAGTTACCGTCTCCTTTCGCTAACCCGCAAGAATCAGGGCGACGGCTGGGCTTCCGAAATCTCCGGCCCCTGGATTCGCGCGCCTGACACGCGCGCCGCGCCTTATAAATAGGACTTGAAGGCCATCTTCCTTTCCTCCCCTTCTTCGTCTTCCTCGCGACCCAGACCTCAGCGCTCCGCCGCCGCCGTCAGCTGCTGCATCAACTCCGGCCGCTGCATCAACCTGATCGGATCAGAGCACCGCGGCGACTTTCCACATCTGTTTGGACCCCGGTAAGGCCCCTGTTCTTGTTGCTTTGGATCTGTTCTAGGGTTCGTCGTCCTGCTACTGTTTCTTGCTCATCCCCATAGCAGCCTTTGCGAACAGATGATGATACCTGTATCTGGATTTGTAGTTCCTCTCGGTATTCGCCATAAATCTGTATTCGTCTTCTTAGATCTCGCTCATTTCCTGCTTGTCGAACTTACGTTGTCCTGCTGTGAGCCAAACTTCATATTTTTATTTCTTTTTCGCCCTAGCTGTAGATCCATTGCTTTGAAAGAATCCGTATGGGATCTGTTTCTGCTTTCATACTCACAGCTGTTAGATCTGGTCGGTTTAACTCTTAGCAAAGTGAACAAAACGATCCACACATCCATTAGTCCCCTTGATGAACCGCTGGTTGATATCATTGTGGATAACCTGACTCTATATAAGATCATCTTCGGTTTATAATCACACTTGTTCTCAAACCGGAATACATTTATCTTGTAGATTTCCGCATGGCAAAACAGGTGTACGAATGCAACTGGGTTCCCTCTTGCATCACTGAGACCCAACTAAACAACTTAGTTCTGACTGGTGCTTTGAGTAGCAAAAATACCATCCATTGGAGGGTTCCAGGAGACGAATGTCCTCCTACCCCGCAGGAGGGAGAAGTGGTGGTGTTTGCGGATCACCTGGCCCGGGGGTTTAACCCACCCGGTTCAAAATTTTATCGGGACGTCTTGGCTAATTTTAGGTTGCGCCCACAAGATGTTGGTCCAAATTCAGTCACGAACATCTGTCATTTTCAAGTACTTTGCGAGGCGTACTTTCAAGAGGAACCCACTGTAGAGCTGTTCCGGGACTTTTTCCATCTGAACCGACGTACTGAATTTACCGATGGCCCTAATATGGAACTGGGTGGTATGGCGATTCAGAAGAGGAAAGAGGTGACTTATCCTCACGCCAAGTTGCATAGTCATCCGCAAGAATGGAATTGCACTTGGTTCTATTGCAAGGATACTTCCCCTGCTGAAGAAAATCCACTGCCCGGTTTCCGTCCAGAGAGACTTAGCAATACTCACCCCTTCCCCCCACGGCTAACTGCCAAAGAGAGGAGTAAGTATGCCCCTCAGCTGTCCAAGCTCAGAGCCTTCATGGCTAACGGTTTAACGGGGGTAGATCTTGCTCGTTGCTGGATTTCATGGAGCATCCTGCCCCTTAGCATCCGCTCCGGTTTAATGTGCCAATACACGGGTCGGGTTGATGATCCTTTGAGGCACTGCAACATTCAGCTGACGGAGGACGAAGTTGCTGAAGCTGTGAAGAAGATGCTAAACGAACCGGAACATGTTTGCGCTCGAACCGGCCTGCTTCCATTTTGTGCCACAAACAAACCACCTGCTGTAAGTAACTCCTCTGTTTATAGAATCTTTGATCTCCTTATATATATGTTGTTAACTTCCAGTCTTTTTCGAAACAGGGAGATGATCCGTTTTGGAGCAAGAAGCTTCAGGAGCCAACCGAAAAGATAATCAGACCCAAGACAAGGGTGAAGAAGCCTGCCACGAAGAAAAGGAACACTGCTTCCTGTACCCCCGATTTAGAGGAAGATGATGTGGGTAATCCGGACCTTGGGGTAGACCTCGATTCAGTTGGCTTACTTTTTGTACGTCTTATTGATGATGATATTTGTCAGGACGACGCTGAAGCGAGCAATGCTGAAGGCCCAGAGGTAACTATTCTTTCTTCTGATGCCGTTCCTTTGCCTTTGAAAAAACCTCGCCAAGCAAACCGGAAAATTAAATTTTCTCACCCTCTTGCTTATTTGGATCCCAAATTTCTTGTGAAGACCCAGCAACATGAAGCTCGACGCACCACTCGGCATAGCGGCCAGGTAGTCACCTCCGCCGGTTTACCGAACAGCCCGGTTCGGAAACGCCGCTCAGAGGTCTCCAATTTATGCGCTAGCTTTCCTCCTAAAGCAGGTTTAACTCGTCAGCCTCTAAACCCATCTGACTCCGATTATCAGGTCACTTCCCATTCATCTTTCGGTGAATCTTCAGCCACTCAACTGCCACCGTTGAAGACAGTTATTGGGTAAGTTGGACCGATCACAACATATCTCTGTATGGGTCTCTTAATCCATCTTATACCGTTTTTTCAATCTTTGGGCTTTTTCCAGGGCCAAACCTAGACCGAGCAAGAAGGCTCGTCTGGATAACCCGGCTGGAGGAACTGCCGCTCCTGAACCGGAGAAAACTCCTGGTGCTGACGCTCTGGGTCGTGAAGATACTCAGAGTTATCTATCTCCGCAAGATGACACCTGCGCTGAAGAAAGGATTATCGAACCTGCCAGCCATACTGATCTGCCTGCCAGTCCGGTTCGGGTTGAGGAATCCATCCCCCCTTCTGGAACTGCTGATAAACCGACAGGGCCACTGCAGAGCAGCGATTCCAAGGATGATGATGTTGTGATTACCGGCATCGGTCACTCTGAACCGGGGAACACTGCTACCTTAGCCAAGCATACTGCCAAGGACGAAATCTCGGCCATGAAGGGCAAATGGGACCTTGACTCTTCTACATACGCCGCTTTAAGCGCCCCGGATCTTTATTCCGGGTACCTGAACCGACTGTACACCAGTCGGGATTATGAAGCTGGCATGATCAAGATGATGAAGGAGAAACTTGAGGTAACTCCTTTGTGCTTCATTCCATTGCAACTTAGCTTATCAATATTTCCAAACCGAGATGGTAAACCGGATCAGGCTGTCTTGACAATTAAAGTCCGGTTTAGAAAACCCAAGGCTTCAACATAGGTCATAACCAGAAAACATCATTAGATAACTATTAGCCCCCAAGTGCCAAGTTTAAATACTTGTATTAATCTTGGGACCTTTTGCAATGGGAAACTTGCGTTAAAATCAAATATGCATTAGCCCCCAAGTATCGAGCACATAACTTGCTTGTGTGGTTGATACTTCCAAACCGTGCAATATTTTCTGAACACTGTTTTGACGACCTGCTGCAGGGGGATCTAAAGACCAAAGCGAAACAAGTCTCAGACCTTCAGGAAATCTTAAAAACCCAACAGGCTGAAACTGAAAAAACGAAGGACGAATTGGCCCATGCTTTAACCGTTATGGAACAGCTGAAAGAAAGCCACCAGAAAGAACAAGCTGATTGGGTTACTGAGAGAGCTCTTTTGACGAAGAGGGCTGAAAATGCCGAAGCTGCTCTTAAACCGGTGGTCGATGAATTATCCACCGTGAAGCGACAGATACACTCCATGACCTCTGCCGTCTTTGGTAAGCTTTTCGACACCCTTCTTCCAATTTGTACCTTATCTGTTACATCCTATGTCCGGTTTGTAATCCCTCTTGAAACACTGCAGGCTCACGTATTGGACACTTGGGTTCTGATGTGCGGATGAAACTCAAAGCTGCTTATACTCTGATTGAACAGTTGTACACCGGAGCACAAAGGGCTATTACCACTGCGTCGCATAACAACCCTGCGCCGTCTCTGATTCAAGACACATTAAAGAGGCTGTCCATGCTTCCAGCCCGGATTGAAGAATTGAAAAGATCAGCTGCTCGAACTGGAGCCATTACTGCCTTGATCCGGGCCAAAGCATGGGTACCAGATTTCGATCCTGTCGAAGCGGCCCAAGGATACCCCAGCTTGAAGGAAGATGGTTCAGACTTTGGTGAAGACGATTTGAGGGCGATAAACCGGGCGGTGCGCCCCCTAGCTTGTCAATTGGCTGAGGAAGCAGATCTGTCACGTTACCAAGCCCAATACGACAACCAGAACAAACGAGTGGCTGCTCCGGTCCCTGACACGGGAAACCTTATTCCTCCGATCCGTAAGCATACTTACGCCCCTGACATTGAACCGTCTTTGCTGATTCACGAAGAAGCCGTCTTTCAAGCGCTAATGGGAATCGACTGGACCACTGCTGATTTCCAGCCAATGGGTAACCAAGATAGAGCTGAAGCGGCGCAAGACGATCCCCAGCCTTCGAACCGAGGCGGCGAACAGGCCTAATCAACGCTCCGGTTCACCTTACTTGGTGACGTTTTAATCCGTCAACGAACAATATATTCAGGCACTACGTTTGCCTTGTAATAAGATAGTTTAATACCTCTGCATTTGAATGTGCCTTCGCGCACGGGTACTGATGCTCTTTCCAATGACGTTTTCCAACTCTGTTTCCTGTCTTTACTGAGTACACAATGTGATTTTTCGACCACCTGAACCGTGCTGCGCCTTTGCCAAACCGGGCAATTTGCCTCCGGGTTAAGAATACTTGACGTTCCGAGGAACCAAAAAACACCACAATAAAGGCAGAAAACAAAACCAAATATGAAAAGGCAACGCGATAGTAAAACGCATGCGAATAGTGGTTACTATGAACCGTTCAGAAGGCGTGACCATCGTCAAGACAAAACCGAGCCCCCAAGAGGTGTAGCTACGGTTTAGGTCGACTAGGTTCCTAACTGAATCGTGGCAATTATGCCGGCCAAATGGCATTGCATTGGTTCGTCAACCAAGCCCCCAAGTGACTCAATGGCCAAGGGCCGATCAAGTGGCATTGCGTTGGTTCGGACACGACCAAGCCCCCAAATGATTCATTGGCTAAAAAGCCGACCAGAAGGCATTACCATCATCAAGACATGACCGAGTTCTCATAAGGCGTAATCTACGGTTCAAGTCGATTAGGTCCCCAACTGATTCAATGGCAAATGTGCCAGCCAGAAGGCATTACCATCGTCAAGACATGACCAAGCCCCCCGGAGGTGTAGCTACGGTTCAGGCCGACTAGGTCCCCAACTGACTTTATAGGGATAGCTATGTTCTCTTTGGTTCGAATACGACCTATGTTTGAACAAGAAGCCCCGTGTTTGAATAGCTATGTTCTCTTTGGTTCGAATACGACCTATGTTTGAATAGGAAACCCCGTGTTTGAATAGCTATGTTCTCTTTGGTTCGAATACGACCTATGTTTGAACAGGAAGCCTCATGCTTAAACCGGGATATTGAACCGGATATTAAGAGCTTCACAGCCTTGTAGGAGACATCTTCCCTTGAACCGGCTTTTAAACCGGAGTCTATAATTTGAATTTCTGTGAAACGACCTCCAGGGGTAAGCACACATGATGTGAAGAACAGTAGAGACCCCGTTTTACTCGAGGCTCCGGTTTATTGTATTAATCATAATATATACATGGTCATGATAGGTACATAAACAGAGCCCAGGGCTCATGTATAATAAGGTCGAAGATGAGCTATGTTCCACGGCCTGCTGGTTTCCTCCTCTGATGTGCGTGAATCCTTGCGCTCTCGAATATCAATCAGGTAATATGACCCATTATTCAGATTCTTGCTGACCACAAAAGGTCCCTCCCAAGGCGGGGATAATTTATGCTGATCAGATTGATCCTGGATGAGCCGAAGCACCAAATCCCCCTCCTGAAAGGTTCTGGTCCTAACCCGACGGCTGTGGTAACGGCGAAGATCTTGCTGGTAAATCGCTGAACGGGCAGCTGCCAAGTCACGCTCCTCATCCAACAAGTCAAGAGCGTTCTGCCGTGCCTGCTCATTATCCACTTCAACATATGCTGCCACGCGAGGTGAGTCATGTCGTATATCACTTGGGAGAACCGCCTCTGCTCCATAAACCATAAAAAACGGCGTAAACCCCATGGATCTGTTAGGCGTGGTGTTGATGCTCCATAATACAGACGGTAGCTCCTCCACCCAACAACCCGGCGTCCTTTGCAAAGGGACCAGAAGCCGGGGTTTGATACCTCTCAAGATCTCTTGGTTCGCTCGTTCTGCCTGACCATTAGACTGTGGGTGCGCTACCGAAGAAACATCAAGCCGTATATGCTCCCGTGCACAGAACTCCTTCATGGCCCCCTTGGACAGATTGGTACCATTATCTGTGATGATGCTGTGTGGAAAACCAAACCGGAAGATCACCTTCTTTATAAACTGAACCGCCGTGGCAGCATCACACTTAATGACAGGTTCGGCCTCCACCCATTTTGTAAATTTGTCAGCCGCCACCAGTAGGTGGGTCTTCTTATCTTTGGAACGCTTAAAAGGCCCAACCATATCAAGCCCCCAAGTCGCGAACGGCCAAGTGATTGGAATCATCCTCAATTCTTGAGCCGGTATGTGCGCTCGTCTGGAAAATTTCTGGCAACCATCACATAACCGGACCAGGTCTTCCGCATCAGCATGAGCAGTTAACCAGTAGAAACCGTGGCGGAACGCTTTAGCTACCAATGATTTTGAACCGGCGTGGTGGCCGCAATCCCCTTCATGTATTTCTCGTAATATCTCGCACCCCTCCTTAGGGGATATGCACCGTTGCAGCGCTCCTGACACGCTGCGATGATGTAACTCTCCTTGGAAAATGGTCATAGACTTGGACCGTCGTACTATCTGTCGAGCCAAGCTTTCATCCTCTGGCAACTCACCCCGGTTCATATATACCAGGTAAGGGAGCGTCCAATCCGGGGTGGCGTGAAGAGCTGCCACTAACTGAGCCTCCGGATCAGGAACAGCCAACTCCAGCTCACCGGGCAGCTGTACCGATGGGTGGTACAATACATCCAAGAAAACATTGGGTGGGACCGGTTTACGCTGAGAGCCCAAGCGACTTAAAGCGTCCGCTGCCTCATTCTTTCTGCGGTCCACATGATCCACTTGATAGCCTTTGAAATGCCCAGCCACCATATCTACTTCACGTCGGTATGCAGCCATAAGCGGATCCTTAGAGTCCCAGGTGCCAGATACCTGCTGAGCCACCAGATCCGAATCACCAAAGCACCTAACTCTGCTCAGATTCATCTCTTTAGCCACCCGAAGACCATGAAGCAAAGCTTCATACTCCGCCGCATTATTTGTACAGGGGAACATTAAACGGAGGACATAACAAAACTTATCTCCTCGTGGGGAAGTTAAAACGACTCCAGCCCCCGAGCCTTCCAATTGTCTTGACCCGTCAAAATGAACGGTCCAATAGGTGTTATCTGGCTTCTCCTCGGGCGCTTGTAACTCTGTCCAATCGTTTATGAAATCGACAAGTGCCTGTGACTTTATGGCCGTTCGGGGTACGTACCTTAAATCGTGCGGCCCGAGCTCGATAGCCCACTTGGCAATCCGACCGGTCGCCTCCCGGTTCTGTATAATGTCACCCAAAGGAGCAGAACTGACCACCGTGATTGGGTGTCCTTGGAAATATTGCCTCAGCTTCCGGCTTGCCATAAAAACGCCATATACCAGCTTCTGCCAATGCGGATACCTTTGTTTGGACTCGATAAGTACTTCACTGATATAGTAAACCGGTCGCTGAACCGGATGTTCCTTTCCAGCCTCCTTTCGTTCTACCACCATCGCCACACTGACCGCTCTGGCATTCGCTGCAACATATAACAGGAGTGGCTCTTTATCTACAGGGGCTGCTAATACTGGCGGATTGACTAGCTGTCGCTTCAAGTCCTCAAACGCTTTATCCGCCTCCGGACTCCAGACAAATTGATCCGTCTTCTTGAGCATTTGATACAAGGGGATCGCCTTTTCACCAAGGCGACTGATAAACCGGCTTAACGCTGCAATCCGCCCGGCCATGCGCTGAACATCATTGATGCATTTTGGTTTGGCCAAGGAGGTGATGGCTGTGATCTTTTCTGGATTGGCCTCAATTCCTCTATGGGACACTAAAAATCCCAACAGCTTGCCTGCAGGTACACCAAAAACACATTTAGCCGGGTTGAGCATCATTTGGTACACTCTCAAGTTATCGAAAGTCTCCTTCAAATCGTCAACCAGGGTCTCCTTCTTTCGTGACTTGACCACAATATCATCCACATAAGCATGCACATTACGACCAATCTGCTCGTGGAGGCAATTTTGCACACATCGTTGATAAGTAGCCTGCGCACTCTTAAGCCCAAAGGGCATAGACACATAGCAGAAGGCTCCAAAGGGAGTAATGAACGCTGTCTTCTCCTGGTCCTTAACTGCCATCTTGATCTGATGATAACCCGAATAAGCATCCAAAAAACTTAAACGCTCGCAACCTGCCGTAGCATCAATGATCTGATCAATCCGGGGGAGAGCAAAAGGATCTGCTGGGCAAGCTTTATTCAAGTCTGTGTAGTCCACACACATGCGCCAGGTGCCGTTTTTCTTAAGAACTAGCACCGGATTAGCGAGCCATTCGGGATGAAAAACCTCAATGATAAACCCAGCTGCCAGGAGCCTGGCCACTTCCTCACCAATCGCTTTACGTCTTTCTTCGTTAAACCGCCGTAAGAACTGCTTTACCGGCTTGTACTTAGGATCCAGATTAAGGGTGTGCTCAGCGAGTTGCCTCGGTACACCAGGCATGTCAGACGGCTTCCATGCAAAGATGTCCCGATTCTCACGGATGAACTCGATGAGCGCGCTTTCCTATTTTGGATCCAAGTTTGCACTGATGCTGAACTGCTTGGATGAATCGCCAGGTACGAAGTCAACAAGCTTAGTTTCGGCCGCTGACTTGAACTTCAAGGCCGGATCGTGGTCCGTAGTTGGCTTTTTTAATGAAGTCATATCCTCCGGATCAACACTGTCTTTGTACTGCTTTAGCTCCTCCGTAGCACAAACCGATTCTGCATAAGTCGCATCTCCTTCCTCGCATTCCAAAGTGATTCGACGGCTCCCATGAACCGTTATAGTGCCCTTGTAACCTGGCATCTTAAGTTGCAGATACACGTAGCAGGGCCGTGCCATAAATTTTGCATAAGCTGGCCGTCCGAACAAAGCGTGGTATGGACTTTGGATTTTAACCACTTCGAACGTCAGCGTCTCTGATCTGGAATCGTGACTATCGCCAAATACTACTTCAAGGGCTATCTTACCAACCGGATATGCAGACTTGCCAGGCACCACCCCGTGGAACACTGTATTGGTCGGTTTGAGATCCTTATCTACCAGTCCCATACGACGGAAGGTCTCGTAATACAAGATGTTAATGCTGCTCCCTCCGTCCATGAGCACCTTGGTGAGCTTATAACCTCCCACCTGAGGCGCCACCACCAATGCTAACTGACCCGGATTATAAACCCTGGGAGGGTGATCCTCTCGGCTCCATATGATAGGCTGTTCTGACCAGCGCAGATAGTGAGGCGTGGCTGGTTCGATAGCACTAACTGCTCGCCTCTGAAGCTTTCTATCCCGTTTATCCAGGCTTGTAGTAAAGACATGATACTGTCCACCACTCAATTGTTTCGGGTTGCTCTGATAACCTGTTTGCTGCTGCCCGAAACCACTCTGATTACTTTGATTATTTTGGCTGACCTGTCCGCCCGGATTGCCCTGGAACCCTGGACCGGAACCTGAACCGCCGCCGGAGCTGCGATCATTTTGGAAAGTGTTTGAGCTTTTGAACTCCTTCATGATATAACAATCCTTCCAAAGATGGGTTGCTGGTTCCTCCTTTGTTCCGTGCTTCGGACAGGGCTGGTTTAAGAAAAAGTTCAAACGCTCCGGATTAGGACCAGGATCTGTGCGGAAACTTGGCTGTTTCCCCTTGCGCCGCTGGCCCTTATTCTGTGCACTTGTGTTGGCTACAAAGTCCATGCTCCCGTCCGGTTTACGCTTACCTCCGCCTCCATTACCTGTCGGCCGATGCTGCTGGCCTTTGGTGTTGCTATTCTTCCTTCCCTTCCCTGCTTTGTCATCACCAGAGTCAGGATCCTTGGTACTGTCAGAATCCGCATACTTCACTAAAGCGGTCATGAGCGTTCCTATATCTGTACAATCACGCTTCATCCGGCCTAGCTTTAGCTTCAAAGGGCCAAACCGACAGTTGCTTTCCAACAGCACTACAGCGGTGTCTGCGTTGATGCGATCTGATGAGTGCAAAACTTCTGATACTCGTCGTACCCAATGGGTAGTTGATTCCCCTTCTTGCTGAACGCAGGCTGCTAGGTCAACTATTGACATTGGCTGTTTGCACGTGTCCTTGAAGTTCTTGATAAACCGGGCGCGCAATTGGGCCCACGAGCTAATTGAATTAGGCGGCAAACTCTTTAGCCAAGTCCGGGCCGTTCCTTCTAACATCATAGTAAAATACTTGGCACACGCCGCGTCATCCACATCCAGCATCTCCATAGCCATTTCATAGCTCTCCACCCACGTCTCCGGAGGCTGATCTGCCGTGTAGTTTGGTACTTTGCGGGGGCCTTTGAAGTCTTTGGGCAGGCGCACGTTGCGCAAGGCGGGAATAAGGCACGGTACTCCCAAAGAACTGGAAACCACCCCCGGTTCAGCCGCAGCTGCTGGGAAGATAGGTGTAAGCTGATGAGCCTAATGCGGCGCTGCCAATCCGGCCTCCCGTGCACGGGTGCGATCCACATTCTCGTGAGTATTGTCGACGCCCGCCGGATTGTTGCCGCGGAGCGCTTCACGCCTGGCGTTACTTGAAACGGCCGGCTCCTCCACGCGCCTACTATAGCTCCTGCTGGGGCGAGGGGTGGAGTGAATCCGATCACGGCTTTGCGAATACGCTTCTTGCTGGGCCAACGCGGTCCTAAGGAGCTCCTTGACCCGGCGTGTCTCTTCATCCTGCGGTGAATCTCCTTCGATCGGAATGGCTTCCAACCGAGCCGCCGCCGCGACGAGGTTATCCATCGGGTTGGAATAATGACCTGACAGCGTTGGCATAGCCGGTGGTGTATCAGTGTTATGACGAGGCAGGTCTATAATCCGGTGCTGAACCGGGGCGCCGGCTGTCGCCTCCGCACGGTTTACCCCCGGGGTGTTGAAGAGATTTCGAGCGTCGAGGGCGGCGGGCAACCGAGACCGGGATTTTCTCTTCAGGACCTCCTGAGACGCCTTCTGGTCCATCATGAGCCTGTAAGCCTGTGCATCTAAAGCGGCGCGCTCCGCCGTCATCCTGATCTCTTCTGCTGCCAGCTCCGCTTTAGCCCGAGTGATCTGCTCTCTCACTTTGGCAATCTCAGCATTGTGAACCTCCTGATCCACCGGGTTGGCTTCCGCCATGAGCGCTGCTAGTGCGTCAAACAGGTCTGAAAAAACTTGGGCTGGTGATCCCATAGGGTCTCCTGCCCGCGCAGCCGTTGCTGCTGCCGACCCGGAGATCGCTGCTGCTGCAGTTGATGATCGTGGCGCCGCCTGTGTCCCAGCCATGAAGATCCCAACCCGATAGGGCAGATCAGAGGGGTCCGGAATACTGTTGCCATCGGAGCCGCCCCCAACCCGATCATCTTGTAACTGGTAAAGAGATTCGGTCTCCCCGGTCGACGTTTCATCGCCAGAGTAGATGGCGGTTTCGTCACCAGATTCGGATCCTTCCTCGTAGCTCCCCCCGTGGATGACTCCCACAAAGGCGTGCTTCCTGGTCGGTTTAGCCAGGGTCGATCCCGTGCACTGAGCTGTCTCGATGAGGTCGGCGCAGACGCCTAACTCGGGGCCCGGTTCTCCGATCTTGCCGATGAAAACATGGATACCGCCAAAGGGGACCCGGTACCCGTACTCGATCGAGCCGGCGTCGGGGCCCCAGCCTGTGCTGTCGATGTAGAGTTTTCCGCGGCGGCTCTTGGTCATCCGTCCCACAGCGTAACCCTCGAGTCCTTCAAAGCGGCCCTCCAAGAACCTGAAACCATCTCGCGATAGCCCCACGGTGGGCGCCAAC

TGTCGTGGATTTGTCACGGCAGATGTCCTAGTGAGAGGACTTAGTCGTGGAGCCATCGCAACGGGTTAGCTTGAAGGGGTTAAAGGTGGACACAAGGACACGGTTTTTATACTAGTTCGGCCCCTTCGATGAAGGTAAAAGCCTACGTCTAGTTGTGATGGGATTGATGGGTTTCGAGGGCTTAAGGAGCGATAAGCTTCGCCTAAACCTCGAATTCTTCTTCTCTGTCCCCAAACCGCCGTCGGGTCGTCCCCTTATATACACGGGTGACACCCGTCGGTCTGCAAAGTCCCAACCTCCGGCTTATAAACGTGCCCGGGTCGGTCTCTCTATTCCTATCTTACAATACAAGTTGGCCCCAGGCCGGTTTACACCATAAACCTTTAAACCGGTTATAGGCCTTGGGCCTTCCTATTTCTCCTTGGGCTTTAACATCTCGGGTCTGTTGACGGCGGTAATCCGGGCCCTGCAGGCCGGTTTACCCCAGATAGCAACATCCCCAACA

GTATATATGATTACTATGATGTGGAACAAATAGAAGAATCTGTTGCTTTTATGGGTGCTTATGAAATTGAATCTATGTATGAAGATTTTGAAGATTTTGATGATGATGCTTATAGACCTGAAAA

>Aegilops umbellulata 1-204;

CTTGTTGCGAGTCTTGCGCTCAGTTGTCTCAAGAATAGCAACTGGATGCTCATGATAGGACAAATCTTCTTGAAGATCAATCTCTTCGAAGTTGATTGTGCGCTCAGGCGTCTTGAAGCACTTG

TGTTGGGGATATTGCTATTTGGGGTAAACCGGCCTACAGGGCCCGGGTTAGCTCCGTCAATGGACCCGAGATGTTAAAGCCCAAGGAGAAATAGAAGGGCCCAAGGCCTATAATCGGTTTAGAGGTTTACGGCCGTAAACCGGCCTGAGGTAAACTTGCATTGTAAGTTAGGAATAGAGAGACCAACCCGGGCACGTTTATGAACCGGAGGTTGGGACTTTGCAGACCGACGGGCGTCACCCGTGTATATAAGGGGACGACCCGACGGCGGTTTGAGGACAGAGAACAACAACTCGAGGTTTAGGCGAAGCTTATCGCTCCCTAGCCCTCGAAACACATCAATCCCATCACAACTAGACGTAGGCTTTTACCTTCATCGAAGGGGCCGAACTAGTATAAACTCCGTGTCCTTGCGTCCGCTTTAACCCCTTCAAGTTAACCCGTTGCGATGGCTCCACGACTAAGTCCTTTCACTAGGACATCTGCCGTGACAAATCCACGACA

GTTGGCGCCCACCGTGGGGCTATCGCGAGATGGTTTCAAGTTTTTGGAGGGCCGCTTTGAAGGACTCGAGGGTTACGCTGTGGGTCGGATGACCAAAAGTCGTCGCAGAAAACTCTACATCGACAGCACAGGCTGGGGCCCCGACGCCGGCTCGATCGAGTACGGGTACCGGGTCCCCTTTGGTGGCATCCATGTTTTCATCGGCAAGATCGGTGAACCGGGCCCCGAGCTGGGCGTCTGCACCGACCTCGTCGAGACAGCTCAGTGCACGAGATCGACCCTGGCTAAACCAACCAGGAAGCGCGCCTTCGTGGGAGTCATCCACGGAGGAAGCTACGAGGAAGGATCCGAATCCGGTGACGAAACCGCCATCTACTCCGGCGATGAAACGTCAACCGGGGAAACTGAATCTCTATACCAGCTGCAAGATGATCGGATTGGGGGCAGCTCCGATGGCGACAGTATTCCGGACCCCTCTGATCTGCCCTATCGGGTTGGGATCTTCATGGCTGGAACACAGGCGGCGCCACATTCATCAACTGCGGCAGCAGTGATCTCTGGATCGGCAGCGGCAACAGCTGCACGGGCAGGAGGCCCTGCGGATTCACCAGCCCAAGTATTGTCAGACCTGTTTGACGCACTGGCAGCGCTCATGGCGGAAGCCAACCCGGTGGATCAGGAGGTTCACAATGCTGAGATTGCCAAAGTGAGAGAACAGATCACTCAAGCTAAAGCGGCGCTGGCAGCAGAAGAGGCCAGGATGACGGCGGAGCGCGCCGCTTTGGATGCACAGGCTTACAGGCTCATGATGGACCAGAATGCGTCTCAGGAAGTCCTGAAGAGGAAATTCTAATCTTGATTGCCCGCAGCCCTCGATGCCCGAAATCTCTTCAACACCCCGGGGGTAAACCGTGCGGAGGCGACAGTTGGCGCCCCGGTTCAGCACCGGATAATAAACCTGCCTCGTCACAACACTGGTACACCACAGGCCGTGCCAACACCGTCGGGCCATTATTCCAACCCGATGGATAACCTCGTCGCGGCGGCAGCTCGGTTGGAAGCCATTCCGATCGAAGGCGATTCACCGCAGGATGTAGAGACACGCCGGGTCAAGGAGCTCCTTAGGACCGCGTTGGCCCAACAAGAAGCATATTCGCAAAGCCGTGATCGGATTCATTCCACCCCTCGCCCCAGCGGGAGCTATAGTAGGCGCATGGAGGAGCCGGCTGTTTCAAGTAATGCCAGGCGTGAAGCGCTCCGCGGCAACAATCCGGCGGGCGTCGACAATGCTCAGGAGAATGTGGATCGCGCCCGTGCGCGGGAGGCCGGATTAGCAGCGCAGCATCAGGCTCGTCAGCTTACACCTATCTTCCCAGCTGCTACGGTTGAACCGGGGGTGGTTTCTAGTTCTTTGGGAGTACCGTGCCTTGTTCCCACTTTGCGCAACGTACGCCTGCCCAAAGACTTCAAAGGCCCCCGCAAGGTACCAAACTACACGGCAGATCAGCCTCCGGAGACATGGGTGGAGAGCTATGAGATGGCTATGGAGATGCTGGATGTGGATGACACGGCGTGTGCCAAGTATTTTACTATGATGTTAGAAGGAACGGCCCGGACTTGGCTAAAGAGCTTACCGCCTAATTCCATTAATTCGTGGGCCCAGTTGCGCGCCCGGTTTATCAAGAATTTCAAGGACACGTGCAAACAACCAATGTCGATAGTTGACCTAGCAGCTTGCGTTCAGCAAGAAGGGGAATCAACTACCCATTGGGTACGCTGGGTATCAGAAGTTTTGCACTCGTCAGATCGCATCAACGCAGACACCGCCGTGGTGCTGTTGGAAAGCAACTGTCGGTTTGGCCCTTTGAAGCTAAAGTTAGGCCGGATGAAGCGTGATTGCACGGATATGGGAACGCTCATGACCGCTTTAGTGAAGTATGCGGATTCTGACAGTACCAAGGATCCTAACTCTGGTGACGACAAAGCAGGGAAGGGAAAGAAGAATAGCAACACCAAAGGCCAGCAGCATCGACCGACAGGTAATGGAGGCGGAGGTAAGCGTAAACCGGATGGGAGCATGGATTTTGTGGCCAACACAAGTGCACAGAATAAGGGCCAGCGGCGCAAGGGGAAACAGCCAAGTTTTCGCGGAAATCCCGGTCCTAATCCGGAGAGTTTGAACTTTTTCTTAAACCAGCCCTGTCCGAAGCACGGAACAAGAGGAGCCAGCAACCCATCTTTGGAAGGATTGTTACATCATGAAGGAATTCAAAAGCTCAAACACTTTCCAATATGATCACAACTCTGGCGGCGGTTCAGGTTCCGGATCAGGATACGGCGGCGGAAATTCCGGTCCAGGGTTCCACGGTAATCCGGGCGGACAGGACAACCAAAATAATCAAGGTAATCAAAGTGGTTACAGACAGTAGCAATCAGGTTATCAGAGCAACCCGAAACAGTTGAGCGGTGGACAGTATCATGTCTTTACTACAGGCTTGGATAAACGGGATAGAAAGCTTCAGAGGCGGGCAGTTAGTGCTGTTGAACCGGCCACGCCTCACTATCTGCGCTGGTCGGAACAACCTATCATATGGAGCCGAGAAGATCACCCTCCCAGGGTTTATAATCCGGGTCAGTTAGCATTGGTGGTGGAGCCTCAGGTGGGAGGTTATAAGCTCACCAAGGTGCTCATGGACGGAGGGAGCAGCATTAACATCTTGTATTATGAGACCTTCCGTCGCATGGGACTGGTAGATAAGGATCTCAAACCGACCAATACAGTGTTCCACGGGGTGGTGCCTGGCAAGTCTGCGTACCCGGTTGGTAAGATAGCCCTTGAAGTGGTCTTCGGCGATAGTCATGATTCCAGATCAGAGACGCTGACGTTCGATGTGGTTAAAATCCAAAGTCCATACCACGCTTTGTTCGGACGGCCAGCCTATGCAAAATTTATGGCACGGCCCTGCTATGTATATCTGCAACTCAAGATGCCGGGTTACAAGGGCACTATAACGGTTCATGGGAGCCGTCGAATCGCTTTAGAATGCGAGGAAGGAGATGCGACTTATGCAGAATCGGTTTGTGCTACGGAGGAGCTAAAGCAGTACAAAGACAGTGTTGACCCGGAGGACATGACTTCGTTGAAGAAGCCAACTACGGACCACGATCCGGCCTTGAAGTTCAAATCGGCTGCCGAAACTAAGCTTGTTGACTTCGTACCTGGCGATTCGTCCAAGCAGTTCAGCATCAGTGCAAACCTGGATCCAAAATAGGAAAGCGCGCTCATCGAGTTCATCCGTGAGAATCGGGACATCTTTGCATGGAAGCCGTCTGACATGCCTGGTGTACCGAGGCGACTCGCTGAGCACACCCTTAATGTGGATCCTAAGTATAAACCGGTAAAACAGTTCTTACGCCGGTTTAACGAAGAAAGACGTAAAGCGATTGGTGAGGAAGTGGCCAGGCTCCTGGCAGCTGGTTTTATTATTGAGGTTTTTCATCCCGAGTGGCTTGCTAATCCAGTGCTGGTTCTTAAGAAAAATGGCACCTGGCGCATGTGTGTGGATTACACAGACTTGAATAAAGCCTGTCCAGCGGATCCTTTTGCTCTCCCCCGTATTGATCAAATCATTGATGCTACGGCAGGTTGCGAGCGTTTGAGTTTTTTGGATGCCTATTCGGGTTATCATCAGATTAAGATGGCAGTTAAGGACCAGGAGAAGACGGCGTTCATTACTCCCTTTGGAGCCTTCTGCTATGTTTCTATGCCCTTCGGGCTTAAGAGTGCGCAGGCTACTTATCAACGGTGTGTGCAAAATTGCCTCCACGAGCAGATTGGTCGTAATGTGCATGCTTATGTGGATGATATTGTGGTCAAGTCACGAAAGAAGGAGACCCTGGTTGACGATTTGAAGGAAACTTTCGATAACTTGAGAGTGTACCAAATGATGCTCAACCCAGCTAAATGTGTTTTTGGTGTACCTGCAGGCAAGTTGTTGGGATTTTTAGTGTCCCATAGAGGAATTGAGGCCAATCCGGAAAAGATGACACCCATCACCTCCCTGGCCAAACCGAAATGCGTCAATGATGTTCAGCGCATGGCCGGACGGATTGCAGCGTTAAGCCGGTTTATCAGCCACCTCGGTGAAAAGGCGATCCCCTTGTATCAAATGCTCAAGAAGACGGATCAATTTATCTGGAGTCCGGAGGCTGATAAAGCGTTTGAGGACTTGAAGCGACAGCTAGTCAATCCGCCAGTGTTGGCAGCCCCTGTAGATAAAGAGCCACTCCTGTTATATGTTGCTGCGAATGCCAGAGCGGTTAGTGTGGCGATGGTGGTAGAACGAAAGGAGGCTGGGAAGGAACATCCGGTTCAGCGACCGGTTTACTATATCAGTGAAGTGCTTATCGAGTCCAAACAAAGGTATCCGCATTGGCAGAAGCTGGTATATGGTGTTTTTATGGCAAGCCGAAAGCTGAGGCAATATTTTCAAGGACACCCTATCACGGTGGTCAGTTCTGCTCCTTTGGGTGACATCATACAGAACCGGGAGGCGACCGGTCGGATTGCCAAGTGGGCTATCGAGCTTGGACCGCACGATTTAAGATACGTACCCCGGACGGCCATAAAGTCACAAGCACTTGTCGACTTCATAAACGATTGGACAGAGTTACAAGCACCTGAGGAGAAGCGAGATAACACATATTGGACTATTCATTTTGACGGGTCCAGACAATTGGAAGGCTCGGGGGCTGGAGTCGTTTTAACTTCCCCACGAGGAGATAAGTTTTGTTATGTCCTCCGTTTAATGTTCCCCTGTACAAATAATGCGGCTGAGTATGAAGTTTTGCTTCATGGTCTTCAGATGGCTAAAGAGATGAATCTGAGCCGAGTTAGGTGCTTTGGTGATTCGGATCTAGTGGCTCAGCAAGTATCTGGCACCTGGGATTCTAAGGACCCGCTCATGGCTGCATACCGACGTGAAGTAGATATGGTGGCCGGGCATTTCAAAGGCTATCAAGTGGACCATGTGGACCGCAGAAAGAATGAGGCAGCGGATGCTTTAAGCCGCTTGGGCTCTCAACGTAAACCGGTCCCACCCAATGTTTTCTTGGATGTACTGTATCACCCATCGGTACAGCTACCCGGTGAGCTGGAGTTGGCTATTCCCGATCCGGAGGCTCAATTAGTGGCGGCTCTTCACGTCACCCCGGATTGGACGCTTCCTTATCTGGCATATATGAACCGGGGTGAGTTGCCAGAAGACGAAAGCTTGGCTCGGTAGATAGTACGGCGGTCCAAGTCTATGACCATCCTCAAAGGGGAGCTACATCATCGCAGCGTGTCAGGAGCACTGCAACGGTGCGTATCCCCTGAGGAGGGGTGCGAGATATTACGTGAAATACATGAAGGGGATTGTGGCCACCACGCCGGTTCAAAATCATTGGTGGCTAAAGCGTTCCGCCACGGTTTCTACTGGTTAACTGCTCATGCTGATGCAGAAGACCTGGTCAGGTTATGTGATGGTTGCCAGAAATTTTCCAGACGAGCGCACATACCGGCTCAAGAATTGAGGATGATTCCAATTACTTGGCCGTTTGCGACTTGGGGGCTCGATATGGTTGGGCCTTTTAAGCGTTCCAAAGATAAGAAGACCCACCTACTGGTGGCGGTTGACAAATTTACAAAGTGGGTGGAGGCAGAACCTGTCAGTAAATGTGATGCGGCCACGGCGGTTCGGTTTATAAAGAAGGTGATCTTCCGGTTTGGCTTTCCACACAGCATCATCACAGATAATGGTACCAATCTATCCAAAGGGGCCATGGAAGAGTTCTGCGCACGGGAGCATATACGGCTTGATGTTTCTTCGGTAGCTCACCCACAATCTAATGGTCAGGCAGAACAAGCGAATCAAGAGATATTGAGAGGTATCAAACCCCGGCTTCTGGTCCCTTTGCAAAGGACGCCGGGTTGTTGGGTTGAGGAGCTACCGTCTGTATTATGGAGCATCAACACCACGCCTAACAGATCCACGGGGTTTACGCCGTTTTTTATGGTCTATGGAGCAGAGGCGGTTCTCCCAAGTGATATACGACATGACTCACCTCGTGTGGCAGCGTATGTTGAAGCGGACAACGAGCAGGCACGACAGAACGCTCTTGACTTGTTGGATGAGGAACGTGACTTAGCAGCTGCCCGTTCAGCGATTTACCAGCAAGATCTTCGCCGTTACCACAGCCGTCGGGTTAAGACCAGAACCTTTCAGGAGGGGGATTTGGTGCTTCGGCTCATCCAGGATCAGTCTGATCAGCATAAATTATCCCCGCCTTGGGAGGGACCTTTTGTGGTCAGCAAGAATCTGAATAGTGGGTCATACTACCTGATTGATATTCGAGAGCGCAAAGATTCACGCACATCAGAGGAGGAGACCAGCAGGCCGTGGAACATAGCTCATCTTCGACCTTATTATACATGAGACTTGGGCTCTGTTTATGTACATATCATGACCATGTATATATTATGATTAATACAATAAACCGGAGCCTCGAGTAAAGCGGGGTCTCTGCTGTTCTTCACATCATGCGTGGTTACATGGAGTGGATTCGCTTTAATGCAATTTCTGGTTTACCCCTTGAGGTCGCTTCACAGAAGATCAAAGTGAAGATTCCGGTTTAAAATCCAGTTCAAGGGAAGATGTCTCCCACAAAGCTTTGAAGCTCTTAATATCCGGTTCAAGATCCCGGTTCAAGAAAAATTTATCTCTTGCAAAAAGGTTAAAAATTACTGAAGAGGACCTGCTGACATGATGCGCGGTTCAAACATGGGGCTTCCTGTTCAAACATAGGTCGTATTCGAACCAAAGAGAACATAGCTATCCTTACAAAGTCAGTTGGGGACCTAGTCGACCTGAACCGTAGCTACACCTCCGGGGAGCTTGGTCATGTCTTGACGACGGTAATGCCTTCTGGCTGGCACATTTTCCACTAAGTCAGTTGGGGACCTAATCGACTTGAACCGTGGTTACACCTTATGGGAGCTCGGTCATGTCTTGATGATGGTAATGCCTTCTGATCGGCTTTTTAGCCAATAAGTCATTTGGGGGCTTGGTCGTGTCCGAACCAACGCAATGCCACTTGATCGGCCCTAGGCCACTGAGTCACTTGGGGGCTTGGTCGAATCCGAACCAATGCAATGCCATTTGACCGGCATATTTGCCACGATTCAGTTAGGAACCTAGTCGACTTGAACCGTAGCTACACCTCTTGGGGGCTTGGTTTCTTTTGTCTTGACGATGGTCACGCCTTCTGAACGGTTCATAGCAACCGCTATTTGCATGCGTTTTACTATCGCGTTGTCCTCTCGTATTTGGTTTTTGTTTTCTGCCTTTATTGTGGTATTTTTGTTCTTCGGAACGTCAAATACTTTTAAACCGATTCAGCTGACACAGCCTGGTCCGTTTTTTAACCCGGAGGCAATTTGCCCGGTTTGGCAAAGGCGCGGCACGGTTCGTATGGTCAAAAGACCATCCTTTGTACTCAGTAAAGACAGGAAACATAAGTTGGAGAAAGTCAACGGAAAGGGCATCAGTACCCGTGCACAAAGGCGCATTCAAACACAGAGGTATTAACTATCTTATTACAAGGCAGCGTAGTGCCTGAATATATTGTTTTTTGACGGATTAAACGTCACCAATTAAGGTGAACCGGAGCGTTGATTACGCCTGTTCGCCGCCTCGGCTCGAAGGCTGTGGATCATCTTGCGCCGCTTCAGCTTTCTCTTGGCTACCCATTGGCTGGAAATCAGCAGTGGTCCAGTCGATTCCCATTAGCGCTTGAAAGACAGCTTCTTCATGAATCAGCAAAGACGGTTCAATGTCAGGGGCGTAGGTATGCTTACGGATTGGAGGAATAAGGTTTTCCGCTTCAGGGACCGGGGCAGCTACTCGCTTGTTCTGGTTATCATATTGGGCCTGATAATGTGTCAGATCTGCTTCTTCAGCCAATTAACAAGCTAGGGGGCGCACCGCCCGGTTTATCGCCCTCAAGTCATTTTCGCCAAAATTTGAACTGTCTTCTTTCAAGCTGGGGTATCCTTGGGCCGCTTCGACAGGATCAAAATCTGGTACCCACGCTTTGGCCCGGATCAAGGCAGTGATGGCTCCGGTTCGAGCAGCTGATCTTTTTAACTCTTCAATCCGGGCTGGAAGCATGGATAACCTTTTTAACGTGTCTTGAATCAGAGACGGTGCAGGGTTGTTATGCGATGCAGTGGTGATAGCTCTCTGTGCTCCGGTGTACAACTGTTCAATCAAAGTATAGGCAGCTTTGAGTTTCATCCGCACATCAGAACCCAAGTGTCCAATACGTGTGCCTGCAGTGTTTCAAGATGGATTACAAACCGGACATGGGATATAGCAAACAAGGTGCAAATTGGAAGAAGGGCATCGAAAGGAATTTACCAAAGACGGCAGAGGTCATGGAGTGTATTTGTCGCTTTACGGTGGATAATTCATCAACCACCGGTTTGAGAGCAGCTTCGGCATTTTCAGCCCTTTTCATCAAAAGAGCCTTTTCAGTAGCCCAATCAGCTTGTTCCTTTTTGCGGATTTCTTTCAGCTGTTCCATAACGGTTAAGGCATTGGTCAATTCCTCCTTCGCTTTTGCAGTTTCAGCCTGCTGGGTTTTTAGGATTTCCTGAAGGTCTGAGACTTGTTTCTCTTTGGTCTTCAGATCCCCCTGCAGCAGGTCGTCAGCAGTGTTCAGAAAATATTGCACGGTTTGGAAGTATCAACCACACAAGCAAGTTATGTGCTCGATACTTGGGGGCTAATGCATACTTGATCTTAACGCATGTTTTCCATTACAAAAGTCCCAGGGTTAATACAAGTATTTAAACTTGGTACTTGGGGGCTAATGGTTATTGGATGATGTTTCTGGTTAACGGCCTATGTTGAAGCCCTGGATTTTTAAACCGGACCTTAGTGGTCAAGGCAGCATGATTCGGATTACCATCTCGGATTGGAATAAATTGATAAGCTAGGTTGCAACAGAAGGAAGCACAAGAGAGTTACCTCAAGTTTTTCTTTCATCATCCTGATCATACCAGCTTCATAATCGCGACTGGTGTACAGTCGGTTCAGGTACTCAGAATAAAGATCTTGGGCGCTTAAAGCGGTGTACGTTGAGGAGTCAACGTCCCATTTGCCCTTTATGGCCGAGATTTCTTCCTTGGCAGTATGCTTGGCTAAAGTAGCAGAGTTCCCCGGTTCAGAGCGACCAATGCCGGTAATCACAACATCATCATCTTTGGAGTCGCTGCTCTGCATTGGCGCTGTCGGTTTATCAACAGTTCCAGAAGGGGGGATGGATTCCTCAACCCGAACAGGACTGACAGGCTGATCAGTACGGCTGGCAGGATTGGTAGGCCTTTCTTCAGCATAGGCGTCATCTTGTGGAGATAGATAATTCTGAATGTCTTCACGACCCAGAGCATCAGCACCAGGAGTTCTCTCCGGTTCAGGAGCGGCAGTTCCTTCGGCCGGGTTATCCAGACGAGCCTTCTTGCTCGGTCTAGGTTTAGCCCTGGGAAAGACCCAAAGATTGAAAGAACGGTGTAAGATGGATTAAGAAACCCATATAGAAATATGTTGTGATCGGTCCAACTTACCCAATAACTGTCTTTAGCGGTGGCAGTTGAGTGGCTGAAGATTCACCAGAAGATGAATGGGAAGTGACCTGATAATCGGAGTCAGATGGATTAAGAGGCTGACGAGTTAAACCTGCTTTAGGAGGAAAGCTAGCGCATAAATCGGAGACCTCTGAGCGGCGTTTCCGAACCGGGCTGTTCGGTAAACCGGCGGAGGTAACTACCTGGCCGCTATGCCGGGTGGTGCGTCGAGCTTCATGTTGCTGGGTCTTCACAAGAAATTTGGGATCCAAGTAAGCAAGAGGATGAGAAAATTTAATTTTCCGGTTTGCTTGGCGAGGTTTTTTCAAAGGCAAAGGAACTGCATCAGAAGAAAGAATAGTTACCTCTGGGCCTTCAGCATTACTCGCTTCAGCGTCGTCCTGACAAATATCATCATCAATAAGACGTATAAAAAGTAAGCCAACTGAATCGAGGTCTACCCCAAGGTCCGGATTACCCACATCATCCTCCTCTAAATCCGGGGTAGAGGAAGCGGTGTTCCTTTTCTTCTGAGCGGGCTTCTTCACCCTTGTTTTGGGTCGGGTTACCTTTTCGGTCGGCTCTTGAAACTTCTTGCTCCAAAACGGATCATCTCCCTATCTCAGAAAGGACTGGAGTTAACAATATGTATATAAGGAGATCATAAATTCTATAACAAACAGAGGTGAGATGCTTACAGCAGGTGGGTTGTTTGTAGCACAAAAGGGAAGCAGGCCGGTTCGGGCGCAAACATATTCCGGTTCATTCAGCATCTTTTTCACAGCTTCAGTAACTTCGTCTTCCGTCAGCTGAATGTTGCAGTGCCTCAAAGGATCATCAACCCGGCCTGTGTATTGGCACATTAAACCGGAGCGGATGCTAAGGGGCAGGATGCTCCATGAAATCCAGCAACGGGCAAGATCTACCCCCATTAAACCGTTGGCCATGAAGGCTCTGAGCTTGGACAGCTGAGGGGCATACTTACTCCTCTCTTTGGCGGTTAGTCTTGGGGGGAAGGGGTGTGTATTGCTAAGTCTCTCTGGACGGAAGCCAGGCAGAGGATTTTCTTCAGCAGGGGAAGTATCCTTGCAATAGAACCAAGTGTGATTCCATTCTTGCGGATGACTATGCAACTTGGCATGAGGATAAGTCACTTCTTTCCTCTTTTGAATCGCCATACCACCCAGTTCCATATTAGGGCCGTCGGTGAATTCAGTACATCGGTTCAAATGAAAACAGTCTCGGAACAGCTCTACAGTAGGTTCCTCCTGGAAGTACACCTCACAAAGCACTTGAAAATGGCAGATATTTGTGACTGAGTTTGGGCCAACATCTTGTGGGCGCAATTTAAAATTAGCCAGGACGTCCCGATAAAATTTTGAACCGGGTGGATTAAAACCCCGGGCCAGGTGATCCGCAAACACCACCACTTCTCCTTCCTGTGGGGTAGGAGGACATTCATCCCCTGGAACCCTCCAATGGATGGCATTTTTGCTACTCAAGGCGCCAGTCAGAACTAAGTTGTTTAGTTGGGTCTCGGTGATGCGAGAGGGAACCCAGTTGCATTCGTACACCTGTTTTGCCATGAGGAAATCTACAAGACAAAGGTATTCCGGTTTGAGAACAGGTGTGATTGTAAACCGAAGATGCTCTTATATAGAATGAAGTTATCTACAATGATATCATCTGGCGGTTCATTGAGGAGACTAATGGATATATGGTTTGGGTTGTTCGCTCTGCTAAAAGTTAAACCGACCAGATCTAACAGTTGTGAATATGAAAGCAGAAACAGATCCCGTACGGATTCTTTCAAAACAATGGATCTACAGCTAGGGCAAAAAAGAAGTAAAAATACGAGGTTTGGCTCACAGCAGAACAGCATAAGTTCGACAAGCAGGGAAATGAGTGGGATCTAAGGAGATGAGTGCAGATTTATGGCGAATATTGAGGTTGACTACAAATCACAATATAAGTATCATAATCTGTTCGCAAAAGCTGCTATGGAGATCAACAAGGAACAGTGGCAGGATGACGAACCCTAGAACAGATCTAAAGCAAGAAGAACAGGAGCCTTACCGGGATCCAAACGGATGTGGAGGGTCGCCGCGGTACTCTGGTCCGGTCAGGTCGATGCAGCGGCCGGAGTTGATGCAGTAGCTGACGGTGGCGGCGGAGCTCCGAGGTCTGGGTCGCGAGGAAGACGAAGAAGGGGACGAAAGGGAGGAAAGGAAGATGGCCTTCAAGTCCTATTTATAAGGCGCGGCGCGCGTGCCAGGCGCGCGAATCCAGGGGCCGGTGATTTCGGGAGCCCAGCCGTCGCCCTGATTATTGCGGATTAGCAAAAGGAGACGGTAACTTGCGCTGACGTCATATCGGTTTACCGCAGCAAGAGGAGACGAAGAAACGGCGGTTTAACAGTCACTGGAAGACATTTGAAGACAGAAATTCTTATTAAGGATTGACATGAACCTGTTCAAATCAATCTGGGGCCTAA

TGTTGGGGATATTGCTATTTGGGGTAAACCGGCCTACAGGGCCCGGGTTAGCTCCGTCAATGGACCCGAGATGTTAAAGCCCAAGGAGAAATAGAAGGGCCCAAGGCCTATAATCGGTTTAGAGGTTTACGGCCGTAAACCGGCCTGAGGTAAACTTGCATTGTAAGTTAGGAATAGAGAGACCAACCCGGGCACGTTTATGAACCGGAGGTTGGGACTTTGCAGACCGACGGGCGTCACCCGTGTATATAAGGGGACGACCCGACGGCGGTTTGAGGACAGAGAACAACAACTCGAGGTTTAGGCGAAGCTTATCGCTCCCTAGCCCTCGAAACACATCAATCCCATCACAACTAGACGTAGGCTTTTACCTTCATCGAAGGGGCCGAACTAGTATAAACTCCGTGTCCTTGCGTCCGCTTTAACCCCTTCAAGTTAACCCGTTGCGATGGCTCCACGACTAAGTCCTTTCACTAGGACATCTGCCGTGACAAATCCACGACA

ACTTGCGAAGCTGTGACACGTGGAAGACATCGTGCACATTTGCAAAGTTGGAGGGAAGCTCAAGTTGATAGGCGAGATCGCCTCGTTTCCCGATGATCTTGAAGGGACCGACGTATCGCGGGGC

>Aegilops umbellulata 1-208;

TGAAGCACATCCAATTTGGATTCCTTGACGGAGTCGGTACCTTCGTGCATATCAATCAAAGTATCCCAAATTTCCTTTGCATTCTCAAGACGGCTGATTTTGTAGAATTCTTCGGGGCACAATC

TGTTGGGGATATTGCTATCTGGGGTAAACCGGCCTGCAGGGCCCGGGTTACCTCCGTCCATAGACCCGAGATGTTAAAGCCCAAGGAGAAATAGAAGGGCCCAAGGCCTATAATCGGTTTAAAGGTTTATGGTGTAAACCGGCCTGGGGCAAACTTGTATTGTAAGTTAGGAATAGAGAGACCGACCCGGGCACGTTTATAAGCCGGAGGTCGGGACTTTGCAGCCCGAGGGGCGTCACCCGTGTATATAAGGGGACGACCCGACGGCGGTTTGAGAGAAGAAGAACTCGAGGTTTAGGCGAAGCTCATCGCTCCTTAAGCCCTCGAAACCCATCAATCCCATCACAACTAGACGTAGGCTTTTACCTTCATCGAAGGGGCCGAACTAGTATAAAAACCGTGTCCTTGTGTCCACTTTTAACCCCTTCAAGCTAACCCGTTGCGATGGCTCCACGACTAAGTCCTCTCACTAGGACATCTGCCGTGACAAATCTACGACA

GTTGGCGCCCACCGTGGGGCTATCGCGAGATGGTTACAAGTTTTTGGAGGGCCGCTTTGGAGGACTCGAGGGTTACGCGGTGGGTCGTATGACCAAGAGCCGCCGCGGAAAGCTCTACATCGATAGCACAGGCTGGGGCCCCGACGCCGGCTCGATCGAGTACGGGTACCGGGTCCCCTTTGGCGGCATCCATGTTTTCATCGGCAAGATCGGAGAGCCAGGCCCCGAGTTAGGCGTCTGCGCCGACCTCGTCGAGACAGCTCAGTGCACGAGATCGACCCTGGCTAAACCGACCAGGAAGCACGCCTTCGTGGGAGTCATCCACGGAGGAAGCTACGAGGAAGGATCTGAATCCAGTGACGAAACCGCCATCTACTCCGGCGATGAAACGTCGACCGGGGAAACCGAATCTCTTTACCAGCTGCAAGATCATCGGATTGGGGGCGGCTCCGATGGCAACAGTATTTCGGACCCCTCTGATCTGCCCTATCGAGTTGGGATCTTCATGGCTGGAACGCAGGCGGTGCCACGATCATCAACTGCAGCAGCAGTGATCTCCGGATCGGCAGCGGCAACGGCTGCGCGGGCAGGAGGCCCTACGGGTTCACCAGCCCAAGTTTTGTCAGACCTGTTTGACACACTAGCAGCGCTCATGGCGGAAGCCAACCCGGTGGATCAGGAGGTTCACAATGCGGAGATTGCCAAAGTGAGAGAGCATATCACTCGGGCTAAAGCGGAGCTGGCAGCAGAAGAGATCAGGATGACGGCGGAGCGCGCCGCTTTAGATGCACAGGCTTACAGGCTCATGATGGATCAGAAGGCGTCTCAGGAAGTCCTGAAGAGAAAATCCCGATCTCGGTTGCCCGCCGCCCTCGACGCTCGAAATCTCTTCAACACCCCGGGGGTAAATCGTGCGGAGGCGACAGCCGGCGCCCCGGTTCAGCACCGGATAATAAACCTGCCTCGTCATGACACTGGTACACCACCGGCCATGCCAACGCCGTCAGGTCATTATTCCAACCCGATGGATAACCTCGTCGCGGCGGCGGCTCGGTTGGAAGCCATTCCGATCGAAGGAGATTCACCACAGGATGAAGAGACACGCCGGGTCAAGGAGCTCCTTAGGACCGCGTTGGCCCAGCAAGAAGCATATTCGCAAAGCCGTGATCGGATTCACTCCACCCCTCGCCCCAGCGGGAGCTGTAGTAGGCGCGTGGAGGAGCCGGCCGTTTCAAGTAACGCCAGGCGTGAAGCGCTCCGCGGCAACAATCCGGCGGGCATCGACAATGCTCACGAGAATGTGGATCGCACCCGTGCACGGGAGGACGGATTAGCAGCGCAGCTTCAGGATCGTCAGCTTACACCTACGGTTGAACCGGGGGTGGTTTCCAGTTCTTTGGGAGTACCGTGCCTTGTTCCCGCTTTGCGCAACGTACGCCTGCCCAAAGACTTCAAAGGCCCCCGCAAGGTACCAAACTACACGGCAGATCAGCCTCCGGAGACATGGGTGGAGAGCTATGAGATGGCTATGGAGATGCTGGATGTGGATGACACGGCGTGTGCCAAGTATTTTACTATGATGTTAGAAGGAACGGCCCGGACTTGGCTAAAGAGCTTACCGCCTAATTCGATTAGTTCATGGGCCCAATTGCGCGCCCGGTTTATCAAGAATTTCAAGGACACGTGCAAACAGCCAATGTCGATAGTTGACCTAGCAGCCTGCGTTCAGCAAGAAGGGGAATCAACTACCCATTGGGTACGGCGGGTATCAGAAGTTTTGCACTCGTCAGATCGCATCAACGCAGACACCGCCGTAGTGCTATTGGAAAGCAACTGCCGGTTTGGCCCTTTGAAGCTAAAGCTGGGCCGGATGAAGCGTGATTGTACAGATATAGGAACGCTCATGACCGCTTTAGTAAAGTATGCGGATTCTGACAGTACCAAGGATCCTGATTCTGGTGATGACAAAGCAGGGAAGGGAAAGAAGAATAGCAACACCAAGGGCCAGCAACATCGGCCGGCGGGTAATGGAGGCGGAGGTAAGCGTAAACCGGACGGGAGCATGGACTTGGTGGCCAGCACAAGTGCACAGGATAAAGGCCAGCGGCGTAAGGGGAAACAGCCAAGTTTCCGCGCACATCCCGGTCCTAATCCGGAGCGTTTGAACTTTTTCTTAAACCAGCCCTGTCCGAAGCACGGAACAAAGGAGGAACCAGCAGCCCATCTTTGGAAGGATTGTTATATCATGAAGGAATTCAAAACCTCAAACACTTTCCAATATGATCACAGCCCCGGCGGCGGTTCAGGTTCCGGATCAGGATACGGTGGCGGAAATTCCGGTCCTGGGTTCCAGGGTAATCCGGGCGGACATGTCAGCCAAAATAATCAAAGTAATCAGAGTGGTTTCGGACAGCAGCAAACAGGTTATCAGAGCAACCCGAAACAATTGAGTGGTGGACAGTATCACGTCTTTACTACAAGCTTAGATAAACGGGATAGAAAGCTTCAGAGGCGAGCAGTCAGTGCTATCGAACCGGCCACGCCTCACTATCTGCGCTGGTCAGAACAGCCTATCATATGGAGCCGAGAGGATCACCCTCCCAGGGTTTATAATCCGGGTCAGTTAGCGTTGGTGGTGGCGCCTCAGGTGGGAGGTTATAAGCTCACCAAGGTGCTCATGGACGGAGGTAGCAGCATTAACATCTTGTATTACGAGACCTTCCGTCGTATGGGACTGGTAGATAAGGATCTCAAACCGACCAATACAGTGTTTCACGGGGTGGTGCCTGGCAAGTCTGCATATCCGGTTGGTAAGATAGCCCTTGAAGTGGTATTTGGCGATAGTCACGATTCCAGATCAGAGACGCTGACGTTCGAAGTGGTTAAAATCCAAAGTCCGTACCATGCTTTGTTCGGACGGCCAGCTTATGCAAAATTTATGGCACGGCCCTGCTACGTGTATCTGCAACTTAAGATGCCAGGTTACAAGGGCACTATAACGGTTCATGGGAGCCGTCGAATCGCTTTGGAATGCGAGGAAGGAGATGCGACTTATGCAGAATCGGTTTGTGCTACGGAGGAGCTAAAGCAGTACAAAGACAGTGTTGACCCGGAGGACATGACTTCGTTAAAGAAGCCAACCACGGACCACGATCCGGCCTTGAAGTTCAAATCGGCTGCCGAAACTAAGCTTGTTGACTTTGTACCTGGCGATCCATCCAAGCAGTTCAGTATCAGCGCAAACCTGGATCCAAAATAGGAAAGCGCGCTCATCGAGTTCATCCGTGAGAATCGGGACATCTTTGCATGGAAGCCGTCTGACATGCCTGGTGTACCGAGGCGACTCGCTGAGCACACCCTTAATCTGGATCCTAGGTATAAACTGGTAAAGCAGTTTTTACGCCGGTTTAACGAAGAAAGGCGTAAAGCGATTGGTGAGGAAGTGGCCAGGCTCCTAGCAGCTGGGTTTATCATTGAGGTTTTTCATCCCGAATGGCTTGCTAATCCGGTGCTAGTTCTTAAGAAAAACGGCACCTGGCGCATGTGTGTGGACTACACAGACTTGAATAAAGCTTGCCCAGCGGATCCTTTTGCTCTCCCCCGGATTGATCAGATCATTGATGCTACGGCAGGTTGCGAGCGTTTAAGTTTTTTGGATGCCTATTCGGGTTATCATCAGATCAAGATGGCAGTTAAGGACCAGGAGAAGACAGCGTTCATTACTCCCTTTGGAGCCTTCTGCTATGTGTCTATGCCCTTTGGGCTTAAGAGTGCGCAGGCTACTTATCAACGATGTGTGCAAAATTGCCTCCACGAGCAGATTGGTCGTAATGTGCATGCTTATGTGGATGATATTGTGGTCAAGTCACGAAAGAAGGAGACCCTGGTTGACGATTTGAAGGAGACTTTCGATAACTTGAGAGTGTACCAAATGATGCTCAACCCGGCTAAATGTGTTTTTGGTGTACCTGCAGGCAAGTTGTTGGGATTTTTAGTGTCCCATAGAGGAATTGAGGCCAATCCAGAAAAGATCACAGCCATCACCTCCCTGGCCAAACCGAAATGCATCAATGATGTTCAGCGGATGGCCGGGCGGATTGCAGCGTTAAGCCGGTTTATCAGTCGCCTTGGTGAAAAGGCGATCCCCTTGTATCAAATGCTCAAGAAGACGGATCAATTTGTCTGGAGTCCGGAGGCTGATAAAGCGTTTGAGGACTTGAAGCGACAGCTAGTCAATCCGCCAGTTTTGGCAGCCCCTGTAGATAAAGAGCCACTCCTGTTATATGTTGCTGCGAATGCCAGAGCGGTTAGTGTGGCGATGGTGGTAGAACGAAAGGAGGCTGGAAAGGAACATCCGGTTCAGCGACCGGTTTACTATATCAGTGAAGTACTTATCGAGTCCAAACAAAGGTATCCGCATTGGCAGAAGCTGGTATATGGTGTTTTTATGGCAAGCCGGAAGCTGAGGCAATATTTTCAAGGACACCCCATCACGGTGGTCAGTTCTGCTCCTTTGGGTGACATTATACAGAACCGGGAGGCGACCGGTCGGATTGCCAAGTGGGCTATCGAGCTCGGGCCGCACGATTTAAGGTACGTACCCCGGACGGCCATAAAATCACAGGCACTTGTCGATTTCATAAACGATTGGACAGAGTTACAAGCGCCTGAGGAGAAGCCAGATAACACCTATTGGACCGTTCATTTTGACGGGTCAAGGCAATTGGAAGGCTCGGGGGCTGGAGTCGTTTTAACTTCCCCACGAGGAGATAAGTTTTGTTATGTCCTCCGTTTAATGTTCCCCTGTACAAATAATGCGGCTGAGTATGAAGCTTTGCTTCATGGTCTTCGGGTGGCTAAAGAAATGAATCTGAGCAGAGTTAGGTGCTTTGGTGATTCGGATCTGGTGGTTCAGCAGGTATCTGGCACCTGGGATTCTAAGGATCCGCTTATGGCTGCATACCGACGTGAAGTAGATATGGTGGCTGGGCATTTCAAAGGCTATCAAGTGGATCATGTGGACCGCAGAAAGAATGAGGCAGCGGACGCTTTAAGTCGCTTGGGCTCTCAGCGTAAACCGGTCCCACCCAATGTTTTCTTGGATGTATTGTATCACCCATCGGTACAGCTACCCGGTGAGCTGGAGTTGGCTGTTCCTGATCCGGAGGCTCAATTAGTGGCGGCTCTTCACACCACCCCGGATTGGACGCTCCCTTATCTGGCATATATGAACCGGGGTGAGTTGCCAGAAGACGAAAGCTTGGCTCGACAGATAGTACGGCGGTCCAAGTCTATGACCATCTTCAAAGGAGAGCTACATCATCGCAGCGTATCAGGAGCGCTGCAACGGTGCGTATCCCCTGAGGAGGGGTGCGAGATATTACGAGAGATACATGAAGGGGATTGCGGCCACCACGCCGGTTCAAAGTCATTAGTGGCTAAAGCGTTTCGCCACGGTTTCTACTGGTTAACTGCTCATGCTGATGCAGAAGACCTGGTCAGGTTATGTGATGGGTGCCAGAAATTTTCCAGACGAGCACACATACCGGCTCAAGAATTGAGGATGATTCCAATCACTTGGCCGTTTGCGACTTGGGGGCTTGATATGGTTGGGCCTTTTAAGCGTTCCAAAGATAAGAAGACCCACCTACTGGTGGCGGTTGACAAGTTTACAAAGTGGGTGGAGGCAGAACCTGTCAGTAAGTGTGATGCTGCCACGGCGGTTCAGTTTATAAAGAAGGTGATCTTCCGGTTTGGCTTTCCACACAGCATCATCACAGATAATGGTACCAATCTATCCAAGGGGGCCATGAAAGAGTTCTGTGCACGGGAGCATATACGGCTTGATGTTTCTTCGGTAGCGCACCCACAGTCTAATGGTCAGGCAGAACGAGCGAACCAAGAGATCTTGAGAGGTATCAAACCCCGGCTTCTGGTCCCTTTGCAAAGGACGCCGGGTTGTTGGGTGGAGGAGCTACCGTCTGTATTATGGAGCATCAACACCACGCCTAACAGATCCACGGGGTTTACGCCGTTTTTTATGGTCTATGGAGCAGAGGCGGTTCTCCCAAGTGATATACGACATGACTCACCTCGCGTAGCAGCATATGTTGAAGCGGACAATGAGCAGGCACGGCAGAACGCTCTTGACTTGTTGGATGAGGAGCGTGACTTGGCAGCTGCCCGTTCAGCGATTTACCAGCAAGATCTTCGCCGCTACCACAGCCGTCGGGTTAGGACCAGAACCTTTCAGGAGGGGGATTTGGTGCTTCGGCTCATCCAGGATCAGTCTGATCAGCATAAATTATCCCCGCCTTGGGAAGGACCTTTTGTGGTCAGCAAGAATCTGAATAATGGGTCATATTACCTGATTGATATTCGAGAGCGCAAGGATTCACGCACATCAGAGGAGGAGACCAGCAGGCCGTGGAACATAGCTCATCTTCGACCTTATTATACATGAGCCTTGGGCTCTGTTTATGTACATATCATGACCATGTATATATTATGATTAATACAATAAACCGGAGCCTCGAGTAAAGCGGGGTCTCTGCTGTTCTTCACATCATGTGTGTTTACCCCTGGAGGTCGCTCCACAGAAGTTCAAATTATAAATTCCGGTTTAAAAGCCGGTTCAAGGGAAGATGTCTCCTACAAGGCTGTGAAGCTCTTAATATCCGGTTCAATATCCCGGTTCAAGCATGAGGCTTCCTGTTCAAACATAGGTCGTATTCGAACCAAAGAGAACATAGCTATTCCAACACGGGGCTTCCTGTTCAAACATAGGTCGTATTCGAACCAAAGAGAACATAGCTATCCCTATAAAGTCAGTTGGGGACCTAGTCGGCCTGAACCGTAGCTACACCTCCGGGGGGGGGGGGCTTGGTCATGTCTTGACGACGGTAATGCCTTCTGGCTGGCACATTTGCCACTGAATCAGTTGGGGACCTAATCGACTTGAACCGTAGATTACGCCTTATGGGAACTCGGTCATGTCTTGATGATGGTAATGCCTTCTGGTCGGCTTTTTAGCCAATGAATCATTTGGGGGCTTGGTCGTGTCCGAACCAACGCAATGCCACTTGATCGGCCCTTGGCCATTGAGTCACTTGGGGGCTTGGTTGACGAACCAATGCAATGCCATTTGACCGGCATATTTGCCACGATTCAGTTAGGAACCTAGTCGACCTGAACCGTAGCTACACCTCTTGGGGGCTCGGTTTTGTCTTGACGATGGTCACGCCTTCTGAACGGTTCATAGTAACCACTATTTGCATGCGTTTTACTATCGCGTTGCCTTTTCATATTTGGTTTTTGTTTTCTGCCTTTATTGTGGTATTTTTTGTTCTTCGGAACGTCAAGTATTTTTAAACCGATTCAGCTGACATAGCCTGGTCCGTTTTTTAACCCAGAGGCAAGTTGCCCGGTTTGGCAAAGGCGCAGCACGGTTCATAGGGTCGGAAGATCTTGTGTACTCAGTAAAGACAGGAAACAAAGTTGGAAAACGTCATTGGAAAGAGCATCAGTACCCGTGCGCGAAGGCACATTCAGATGCAGAGGTATTAAACTATCTTATTACAAGGCAACGTAGTGCCTGAATATATTGTTCGTTGACGGATTAAAACGTCACCAAGTAAGGTGAACCGGAGCGTTGATTACGCCTGTTCGCCGCCTCGGTTCGAAGGCTGGGGATCGTCTTGCGCCGCTTCAGCTCTATCTTGGTTACCCATTGGCTGGAAATCAGCAGTGGTCCAATCGATTCCCATTAGCGCTTGAAAGACGGCTTCTTCGTGAATCAGCAAAGACGGTTCAATGTCAGGGGCGTAAGTATGCTTACGGATTGGAGGAATAAGGTTTCCCGCTTCAGGGACCGGGGCAGCTACTCGTTTGTTCTGGTTGTCGTATTGGGCTTGGTAACGTGACAGATCTGCTTCCTCAGCCAATTGACAAGCTAGGGGGCGCACCGCGCGGTTTATCGCCCTCAAATCGTCTTCACCAAAGTCTGAACCGTCTTCCTTCAAGCTGGGGTATCCTTGGGCCGCTTCGACAGGATCGAAATCTGGTACCCACGCTTTGGCCCGGATCAAGGCAGTGATGGCTCCAGTTCGAGCAGCTGATCTTTTCAACTCTTCAATCCGGGCTGGAAGCATGGACAGCCTCTTTAACGTGTCTTGAATCAGAGACGGCGCAGGGTTGTTATGCGATGCAGTGGTGATAGCTCTTTGTGCTCCGGTGTACAACTGTTCAATCAGAGTATAAGCAGCTTTGAGTTTCATCCGCACATCAGAACCCAAGTGTCCAATACGTGAGCCTGCAGTGGTTCAAGATGGATTACAAACCGGACATAAGGTGTAGCAGACAAGGTACAAATTGGAAGAAGGGCATCGAAAAGTTTACCAAAGACGGCAGAGGTCATGGAGTGTATTTGTCGCTTCACGGTGGATAATTCATCGACCACCGGTTTAAGAGCAGCTTCGGCATTTTCAGCCCTCTTCGTCAAAAGAGCTCTTTCAGTAGCCCAATCAGCTTGCTCCTTTTTGCGGCTTTCTTTCAGCTGCTCCATAACAGTTAAGGCTTTGGTCAATTCCTCCTTCGCTTTTTCAGTTTCAATCTGCTGGGTTTTTAAAGCTTCCTGAAGGTCCGAGACTTGTTTCTCTTTGGTCTTCAAATCCCCCTGCAGCAGGTCGTCAACAGTGTTCAGAAAATATTGCACGGTTTGGAAGTATCAACCACACAAGCAAGTTATGTGCTCGATACTTGGGGGCTAATGCATACTTGATTTTAACGCAAGTTTTCCATTACAAAAAGTCCCAAGATTAATACAAGTATTTAAACTTGGCACTTGGGGGCTAATGGTTATCTGATGATGTTTTCTGGTTATGACCTATGTTGAAGCCTTGGGTTTTCTAAACCGGACCTTAATTGTCAAGGCAGCCTGATCCGGCTTACCATCTCGGTTTGGAAATATTGATAAGCTAAGTTGCAATGGAATGAAGCACAAGGGAGTTACCTCAAGTTTCTCCTTCATCATCTTGATCATGCCAGCTTCATAATCCCGACTGGTGTACAGTCGGTTTAGGTACCCAGAATAAAGATCCGGGGCGCTTAAAGCGGCGTACGTAGAGGAGTCAAGGTCCCATTTGCCCTTCATGGCCGAGATTTCGTCCTTGGCAGTATGCTTGGCTAAGGTAGCAGTGTTCCCTGGTTCAGAGTGACCGATGCCGGTAATCACAACATCATCATCCTTGGAATCGCTGCTCTGCAGTGGCCCTGTCGGCTTATCAACAGTTCCAGAAGGGGGGATGGATTCATCAACCCGAACAGAACTGGCAGGCGGATCAGTACGGCTGGCAGGTTCGATAGTCCTTTCTTCAGCGCAGGTGTCATCTTGCGGAGATAGATAATTCTGAGTATCTTCACGACCCAGAGCGTCAGCACCAGGAGTTTTCTCCGGTTCAGGAGCGGCAGTTCCTCCAGCCGGGTCATCCAGACGAGCCTTCTTGCTCGGTCTAGGTTTAGCCCTGGAAAAAGCCCAAAGATTGATAAAACGGTATAAGATGGATTAAGAGACCCATACAGAGATATGTTGTGATCGGTCCAACTTACCCAATAACTGTCTTCAACGGTGGCAGTTGAGTGGCTGAAGATTCACCGGAAGATGAATGGGAAGTGACCTGATAATCGGAGTCAGATGGATTTAGAGGCTGACGAGTTAAACCTGCTTTAGGAGGAAAGCTAGCGCGTAAATCGGAGACCTCTGAGCGGCGTTTCCGAACCGGGCTGTTCGGTAAACCGGCGGAGGTGACTACCTGGCCGCTATGTCGGGTGGTGCGTCGAGCTTCATGTTGCTGGGTCTTCACAAGAAATTGGGGATCCAAGTAAGCAAGAGGGTGAGAAAATTTAATTTCCCGGTTTGCTTGGCGAGGTTTTTTCAAAGGCAAAGGAACGGCATCAGAAGAAAGAATAGTTACCTCTGGGCCTTCGGCATGGCTCGCTTCAGCGTCGTCCTGACAAATATCATCATCAATAAGACGTATAAAAAGCAAACCAACTGAATCGAGGTCTACCCCGAGGTCCGGATTACCCACATCATCCTCCTCTAAATCCGGAGTAGAGGAAGCGGTGTTCCTTTTCCTCTGAGCGGGCTTCTTCACCCTCGTCTTGGGTCGGGTTACCTTTTCGGTCGGCTCTTGTAACTTCTTGCTCCAAAATGGATCATCTCCCTGTCTCAGGAAGGACTGGAGTTAGCAATATGTGTACAAGGAGATCATAAGTTCTATAACAAACAGAGGGGAGATACTTACAGCAGGTGGCTTGTTTGTGGCACAAAATGGAAGCAGGCCGGTTCGGGCGCAAACATGTTCCGGTTCATTCAGCATCTTTTTCACAGCTTCAGCGACTTCGTCCTCCGTCAGCTGAATGTTGCAGTGCCTCAAAGGATCATCAACCCGACCCGTGTATTGGCACATTAAACCGGAGCGGATGCTAAGGGGCAGGATGCTCCATGAAATCCAGCAACGAGCAAGATCTACCCCCGTTAAACCGTTAGCCATGAAGGCTCTGAGCTTGGACAGCTGGGGGGCATACTTACTCCTCTCTTTGGCAGTTAGCCGTGGGGGGGAGGGGTGAGTATTGCTAAGTCTCTCTAGACGGAAACCGGGCAGTGGATTTTCTTCAGCAGGGGAAGTATCCTTGCAATAGAACCAAGTGCAATTCCATTCTTGCGGATGACTATGCAACTTGGCGTGAGGATAAGTCACCTCTTTCCTCTTCTGAATCGCCATACCACCCAGTTCCATATTAGGGCCGTCGGTAAATTCAGTACGTCGGTTCAAATGGAAAAAGTCCCGGAACAGCTCTACAGTAGGTTCCTCTTGGAAGTACGCCTCGCAAAGTACTTGAAAATGGCAGATATTCATGACTGAATTTGGACCAACATCTTGTGGGCGCAACCTGAAATTAGCCAGGACGTCCCGATAAAATTTTGAACCGGGTGGATTAAACCCCCGGGCCAGGTGATCCGCAAACACCACCACTTCTCCCTCCTGCGGGGTAGGAGGACATTCGTCTCCTGGAACCCTCCAATGGATGGTATTTTTGCTACTCAAAGCACCAGTCAGAACTAAGTTGTTTAGTTGGGTCTCAGTGATGCAAGAGGGAACCCAGTTGCATTCGTACACCTGCTTTGCCATGAGGAAATCTACAAGACAAGGATATTCCGGTTTGAGAACAAGTATGATTGTAAACCGAAGATGCTCTTATATAGAGTCAGGTTATCTACAATAATATCAACCGGCGGTTCATCAAGGGGACTAATGGATGTGTGGATCGTTTTGTTCACTTTGCTAAGAGTTAAACCGACCAGATCTAAACAGTTGTGAGTATGAAAGCAGAAACAGATCCCGTACGGATTCTTTCAAAGCAATGGATCTACAACTAGGGCGAAAAAGAAATAAAAATATGAAGTCTGGCTCACAGCAGGACAACATAAAGTTCGACAAGCAGGAAAAATAGTGAGATCTAAGGAGATGAGTACGGATTTATGACGACTGCTGAGAGGGACTACAAATCACAATACAGGTATCATCATCTGTTCGCAAAGCTGCTATGGGGATGAACAAGAAACAGTAACAGGATGACAAACCCTAGAACAGATCTAAAAGCGGAAGAACCGAGGCTTTACCGGGATCCAAACAGATGTGGGAAGTCGCCGCGGTGCTCTGGTACGATCAGGTTGAGGCAGCGGCCGGAGTTGATGCAGCAGCTGACGGCGGCGGCGGAGCTCTGAGGTCTGAGTCGCGAGGAAGACGAAGAAGGGGACGAAAGGGAGGAAAGGAAGATGGCCTTCAAGTCCTATTTATAAGGCGCGGCGCGCGTGTCAGGCGCGCGAATCCAGGGGCCGGAGATTTCGGGAGCCCAGCCGTCGCCCTAATTCTTGCGGGTTAGCAAAAGGAGACGATAACTTCGCACTGACGTCATATCGGTTCACCGCAGTAAAAGGAGACGAAGAGAACGGCGGTTCAACAGTTACTGGAAGACATTTGAAAGACAGAATTTTTTTATTAAGGATTGACATGAACCTGTTCAAATCAATCTGGGGCCTAA

TGTTGGGGATATTGCTATCTGGGGTAAACCGGCCTGCAGGGCCCGGGTTACCTCCGTCCATAGACCCGAGATGTTAAAGCCCAAGGAGAAATAGAAGGGCCCAAGGCCTATAATCGGTTTAAAGGTTTATGGTGTAAACCGGCCTGGGGCAAACTTGTATTGTAAGTTAGGAATAGAGAGACCGACCCGGGCACGTTTATAAGCCGGAGGTCGGGACTTTGCAGCCCGAGGGGCGTCACCCGTGTATATAAGGGGACGACCCGACGGCGGTTTGAGAGAAGAAGAACTCGAGGTTTAGGCGAAGCTCATCGCTCCTTAAGCCCTCGAAACCCATCAATCCCATCACAACTAGACGTAGGCTTTTACCTTCATCGAAGGGGCCGAACTAGTATAAAAACCGTGTCCTTGTGTCCACTTTTAACCCCTTCAAGCTAACCCGTTGCGATGGCTCCACGACTAAGTCCTCTCACTAGGACATCTGCCGTGACAAATCTACGACA

CAATCCGTTGAAGAGAATATCACAAGCTTGAGCGTTGTATTGTAGCATCTTCAACTCATCCGCAGTAGCTTCTCGGTTTGGTTCTTTCCCATCAAAGAAGTCACCTTGCAAACCAACACACACA

>Aegilops umbellulata 1-211;

GCGTACCATCTCGCCTCAAAGTTGGCTAGAGAGGTGGAGTAGTACAAGAGTGCCCTGTACTCACTTAGCGATTTGTAATTAAGGTGCTTCTGCATGTTTTTTTCCACGTGCCATGAGCAGAGAC

TGTCGTGGAATTGTCACGTCAGATGTCCTAGTAAGAGGACTTAGTCGTAGAGCCATCGCAACGGGTTAGCTTGAAGGGGTTAAAGTGGACACAAGGACACGGTTTTTATACTAGTTCGGCCCCTTCGATGAAGGTAAAAGCCTACGTCTAGTTGTGATGGGATTAATGGGTTTCGAGGGCTTAGGGAGCGATATGATTCGCCTAAGCCTCGAGTTCTTCTTCTCTGTCCTCAAACCGCCGTCGGGTCGTCCCCTTATATACACGGGTGACGCCCGTCCGTCTGCAAAGTCTCGACCTCCGGCTTATAAACGTGCCCGGGTCGGTCTCTCTATTCCTATCTTACAGTACAAGTTGGCCCCAGGCCGGTTTACACCATAAACCTTTAAACCGTTTATAGATCTTGGGCCTTTCTACTCCTCCTTGGGCTTTAACATCTCGGGTCTTGTTGACGGTGGTAACCCGGGCCCTGCAGGCCGGTTTACCCCAGATAGCAACATCCCCAACA

TTAGGCCCCAGATTGATTTGAACAGGTTCATGTCAATCCTTAATAAGAATTTCTGTCTTCAAATGTCTTCCAGTGACTTGTTAAACCGTCGTTTCTCCGTCTCCTCTGCTGTGGTAAACCGATGTGACGTCAACGCAGGCTACCGTCTCCTTTCGCTAACCCGCAAGAATCAAGGCGACGGCTGGGCTTCCGAAATCTCCGGCCCCTGGATTCGCGCGCCTGATACACGCGCCGCGCCTTATAAATAGGACTTGAAGGCCATCTTCCTTTCCTCCCCTTCTTCGTCTTCCTCGCGACCCAGACCTCAGAGCTCCGCCGCCGCCGTCAACTGCTGCATCAACTCCGGCCGCTGCATCAACCTGATCGGTTCAGAGCACCGCGGCGACCTTCCGCATCTATTTGGACCCCGGTAAGGCCCCTGTTCTTGTTGCTTTAGATCTGTTCTAGGGTTCATCGTCCTGCTACTGTTTCTTGCTCATCCCCATAGCAGCCTTTACGAACAGACGACGATACCTGTACCTGGATTTGTAGTTTCTCTTAGCAGTCGCCATAAATCTGCATTCATTTCCTTAGATCTCACTCTTGTTCCTGCTTGTCGAACTTATGTTGTTCTGCTGTGAGCCCGACTTCATATTACCTGTTTTTATTTCTTTTTCACCCTAGCTGTAGATCCATTGCTTTGAACGAATCCGTACGGGATCTGTTTCTGCTTTCATACTCACAACTGCTTAGATCTGATCGGTTTAACTCTTAGCAAAGTGAACAAAACAACCCACACATCCATTAGTCCCCTTGATGAACCGCTGGTTGATATCATTGTGGATAACCTGACTCTATAAGATCATCTTTGGTTTATAATCACACTTGTTCTCAAACCGGAAAACATTTATCTTGTAGATCTCCTCATGGCCAAACAGGTGTACGAATGCAACTGGGTTCCCTCTTGCATCACTGAGACCCATTTAAACAACTTAGTCCTGACTGGTGCTTTGAGTAGCAAAAATACCATCCATTGGAGGGTTCCAGGAGACGAATGTCCTCCTACCCCGCAGGAGGGAGAAGTGGTGGTGTTTGCGGATCACCTGGCCCGGGGGTTTAACCCACCCGGTTCGAAATTTTATCGGGACGTTTTGGCTAATTTTAGGTTGCGCCCACAAGATGTTGGTCCAAATTCAGTCACGAACATCTGCCATTTTCAAGTACTTTGCGAGGCGTACTTTCAAGAGGAACCCACTGTAGAGCTGTTCCGGGACTTTTTCCATCTGAACCGACGTACTGAATTCACCGATGGTCCCAATATGGAACTGGGCGGTATGGCGATCCAGAAGAGGAAAGAGGTGACTTATCCTCACGCCAAGTTGCATAGTCATCCGCAAGAATGGAACTGCACTTGGTTCTATTGCAAGGATACTTCCCCTGCTGAAGAAAATCCACTGCCCGGTTTCCGTCCAGAGAGACTTAGCAACACTCACCCATTCCCCCCACGGCTAACTGCCAAAGAGAGGAGTAAGTATGCCCCTCAGTTGTCCAAGCTCAGAGCCTTTATGGCTAATGGTTTAACGGGGGTTGATCTTGCTCGTTGCTGGATTTCATGGAGCATCCTGCCCCTTAGCATCCGCTCCGGTTTGATGTGCCAATACACGGGTCGGGTTGATGATCCTTTGAGGCACTGTAACATTCAGCTGACGGAGGACGAAGTCGCTGAAGCTGTGAAGAAGATGCTGAACGAACCGGAACATGTCTGCGCTCGAGCCGGCCTGCTTCCATTTTGTGCCACAAACAAACCACCTGCTGTAAGCAACTCCTCTGTTCATAGAATCTTTGATCTCCTTATATACATGCCGTTAACTTCCAGTCCTTTTCGAAACAGGGAGATGATCCATTTTGGAGCAAGAAGTTACAAGAGCCGACCGAAAAGGTAACCCGACCCAAGACGAGGGTGAAGAAGCCTGCTCAGAGGAAAAGGAACACCGCTTCCTCCACTCCGGATTTAGAGGAGGACGATGTGGGTAATCCGGACCTCGGGGTAGACCTCGATTCAGTTGGTTTGCTTTTTATACGTCTTATTGATGATGATATTTGTCAGGACGACGCTGAAGTGAGCCATGCCGAAGGCCCAGAGGTAACTATTCTTTCTTCTGATGCCGTTCCTTTGCCTTTGAAAAAACCTCGCCAAGCAAACCGGAGAATTAAATTTTCTCACCCTCTTGCTTACTTGGATCCCGAATTTCTTGTGAAGACCCAGCAACATGAAGCTCGACGCACCACCCGACATAGCGGCCAGGTAGTCACCTCCGCCGGTTTACCGAACAGCCCGGTTCAGAAACGCCGCTCAGAGGTCTCCGATTTACGCGCTAGCTTTCCTCCTAAAGCAGGTTTAACCCGTCAGCCTCTAAATCCATCTGACTCCGATTATCAGGTCACTTCCCATTCCTCTTCCGGTGAATCTTCAGCCACTCAACTGCCCCCGTTGAAGACAGTTATTGGGTAAGTTGGACCGATCACAACATATCTCTGTATGGGTCTCTTAATCCATTTTATACCGTTTTTTTCAATCTTTGTGCTTTTTTCAGGGCTAAGCCTAGACCGAGCAAGAAGGCTCGTCTGGATAATCCGGCTGAAGGAACTGCCGCTCCTGAACCGGAGAAAACTCCTGGTGCTGACGCTCTGGGTTGTGAAGATACTCAGAACTATCCATCTCCGCAAGATGACATCTGCGCTGAAGAAAGGACTACCGAACCTGCTAACCGTACTGATCCGCCTGCCAGTCCTGTTCGGGTTGAAGGATCCATCCCCCCTTCTGCAACTGCTGATAAACCGACAGGGCCACTGCAGAGCAGCGACTCCAAGGATGATGATGTCGTTATTACCGGTATTGGCCGCTCTGAACCGGGGAACACTGCTACTTTGACCAAGCATACTGCCAAAGACGAAATCTCGGCCACAAAGGGCAAATGGGACCTTGACTTGTCTACGTTCGCCTCTTTAAGCGCCCCGGATCTTTATTCTGGGTATCTGAACCGGCTGTACACCAGTCGGGATTATGAGGCTGCCATGATCAAGATGATGAAGGAGAAACTTGAGGTAACTCCCTTGTGCTTCATTCTATTGCAACTTAGTTTATCAATATTTCCAAACTGAGGTAGTACACCGGACCAGGCTGCTTTTGACAATTAAGGTCTGGTTTAGAAAACCCCAGGTTTCAACATCGGTCATAATCAGAAAACATCATCAGATAACCATTAGCCCCCAAGTGCCAAGTTTAAATACTTGTATTAATCTTGGGACTCTTTGTAATGGAAAACTTGCGTTAAAATCAAGTATGCATTAGCCCCCAAGTATCGAGCACATAACTTGCTTGTGTGGTTGATACTTCCAAACCGTGCAATATTTTCTGAACACTGTTGATGACTTGCTGCAGGGGGATTTGAAGACCAAAGAGAACCAAGTCTCGGACCTTCAGGAAGCTTTAAAAGCCCAGCAGATTGAAACTGAAAAAGCAAAGGAGGAATTGACCAAAGCCTTAACTGTTATGGAGCAGCTGAAAGCAAGCCGCAAAAAGGAGCAAGCCGATTGGGCTACTGAAAGAGCTCTTTTGACGAAGAGGGCTGAAAATGCCGAAGCTGCTCTTAAACCGGTGGTTGATGAGTTATCCACCGTGAAGCGACAAATACACTCCATGACATCTGCCGTCTTTGGTAAACCTTTCAATGCCCTTCTTCCAATTTGTACCTTGTTTGCTACACCCTATGTCCGGTTTGTAATCCACCTTGAAACACTGCAGGCTCACGTATTGGACACTTGGGTTCTGATGTGCGGATGAAACTCAAAGCTGCTTATACTCTGATTGAACAGTTATACACCGGAGCCCAAAGAGCTATCACCACTGCATCGCACAACAACCCTGCGCCGGCTCTGATTCAAGACACATTAAAGAGGCTGTCCATGCTTCCGGCCCGGATTGAAGAGTTGAAAAGATCAGCTGCTCGAACTGGAGCTATCACTGCCTTGATCCGGGCCAAAGCGTGGGTACCAGATTTCGATCCTGTCGAAGCGGCCCAAGGATACCCCAGCTTGAAGGAAGACGGTTCAGACTTTGGTGAAGATGATTTGAGGGCGATAAACCGGGCGGTGCGCCCCCTAGCTTGTCAATTGGCTGAGGAGGCAGATCTGTCACGTTACCAAGCCCAATATGACAACCAGAACAAACGAGTATCTGCCCCGGTCCCTGAAGCGGGAAACCTTGTTCCTCCAATCCGTAAGCATACTTACGCCCCAGACATTGAACCGTCTTCGCTGATTCACGAAGAAGCCGTCTTCCAAGCGCTAATGGGAATCGACTGGACCACTGCTGATTTCCAGCCAATGGGTAATCAAGATGGAGCTGAAGCGGCGCGAGATGACCCCCAGCCTTCGAACCGAGGCATCGAACAGGCGTAATCAACGCTCCGGTTTACTTGGTGACGTCTTAATCCGTCAACGAACAATATATTCAGGCACTACGTTGCCTTGTAATAAGATAGTTTAATACTTCTGCCTTTGAACGTGCCTTCGCGCACGGGTACTGATGCTCTTTCCAGCGACATTTCCAACTTATGTTTCCCGTCTTTACTGAGTGCACAATATGATTTTTCGACCACCTGAACCGTGCTGCGCCTCTGCCAAACCGGGCAATTTGCCTCCGGGTTTAAAACGGACCGGGCTATGCCAGCTGAATCGGTTTAAGGATACTTGACGTTCCGAAGAACCAAAAAACACCACAATAAAGGCAGAAAACAAAACCAAATATGAAAAGGCAACGCGATAGTAAAACGCATGCGAATAGTGGTTACTATGAACCGTTCAGAAGGCGTGACCATCGTCAAGACAAAACCGAGCCCCCAAGAGGTGTAGCTACGGTTTAAGTCGACTAGGTTCCTTACTGAATCGTGGCAAATATGCCGGCCAAATGGCATTGCATTGGTTCGTCAACCAAGCCCCCAAGTAACTCAATGGCTAAAAAGCCGACCAGAAGGCATTACCATCATCAAGACATGACCGAGTTCCCATAAGGCGTAGTCTACGGTTCAAGTCGATTAGGTCCCCAACTGATTCTGTGGCAAATATGCCAGCCAGAAAGCATTACCGTCATCAAGGCCTGACCAAGCCCCCCGGAGGTGTAGCTACGGTTCAGGCCGACTAGGTCTCCAACTGACTTTATAGGGATAGCTATGTTCTCTTTGGTTCGAATACGACCTATGTTTGAACAGCTGTGTTCTCTTTGGTTCGAATACGACCTATGTTTGAACAGAAATCCCCATGTTTGAATAGCTATGTTCTCTTTGGTTCGAATACGACCTATGTTTGAACAGGAAGCCTCATGTTTGAATAGCTGTGTTCTCTTTGGTTCGAATACGACCTATGTTTGAACAGGAAGCCTCATGCTTGAACCGGGATATTGAACCGGATATTAAGAGTTTCACAACCCTGTAGGAGACATCTTCCCTTGAACCAGCTTTTAAACCGGAATTTGAACTTCTGTGAAGCGACCTCCAGGGGTAGGTACACATGATGTGAAGAACAGTAGAGACCCCGCTTTACTTGAGGCTCTGGTTTATTGTATTCAATCATAATATACACATGGTCATGATATGTACATAAACAGAGCCCAGGGCTCATGTATAATAAGGTCGAAGATGAGCTATGTTCCACGGCCTGCTGGTCTCCTCCTCTGACGTGCGTGAATCCTTGCGCTCTCTAATATCAATCAAGTAATATGACCCATTATTCAGATTCTTGCTGACCACAAAAGGTCCCTCCCAAGGCGGGGATAATTTATGCTGATCAGATTGATCCTGGATGAGCCGAAGCACCAAATCCCCCTCCTGAAAGGTTCTGGTCCTAACCCGACGGCTGTGGTAACGGCGGAGATCTTGCTGGTAAATCGCTGAACGGGCAGCTGCCAAGTCACGCTCCTCATCCAACAAGTCCAGAGCGTCCTGCCGTGCCTGCTCATTGTCCGCTTCAACATATGCTGCCACTCGAGGCGAGTCATGTCGTATATCACTTGGGAGAACCGCCTCTGCCCCATAAACCATAAAGAACGGCGTAAACCCCGTGGATCTGTTAGGCGTGGTGTTGATGCTCCATAATACAGACGGTAGCTCCTCCACCCAACAACCCGGCGTCCTTTGCAAAGGGACCAGAAGTCGGGGTTTGATACCTCTCAAGATCTCTTGGTTCGCTCGTTCTGCCTGACCATTAGACTGTGGGTGCGCTACCGAAGAAACATCAAGCCGTATATGCTCCCGTGCACAAAACTCTTTCATGGCCCCCTTGGATAGATTGGTACCATTATCCGTGATGATGCTGTGTGGGAAACCAAACCGGAAGATCACCTTCTTTATAAACCGAACCGCCGTGGCAGCATCACATTTACTGACAGGTTCGGCCTCCACCCACTTTGTAAATTTGTCAACCGCCACCAGTAGGTGGGTCTTCTTATCTTTGGAACGCTTAAAAGGCCCAACCATATCAAGCCCCCAAGTCGCGAACGGCCAAGTGATTGGAATCATCCTCAATTCTTGAGCCGGTATGTGTGCTCGTCTAGAAAATTTCTGGCACCCATCACATAACCTGACTAGGTCTTCCGCATCAGCATGAGCAGTTAACCAGTAGAAACCGTGGCGGAACGCTTTAGCCACCAATGATTTTGAACCGGCGTGGTGGCCGCAATCCCCTTCATGTATTTCTCGTAATATCTCGCACCCCTCCTTAGGGGATATGCACCGTTGCAGCGCTCCTGATACGCTGCGATGATGTAACTCTCCTTGGAAAATGGTCATAGACTTGGACCGTCGTACTATCTGTCGAGCCAAGCTTTCATCTTCTGGCAACTCACCCCGGTTCATATATGCCAGGTAAGGGAGCGTCCAATCCGGGGTGGCATGCAGAGCCGCCACTAACTGAGCCTCCGGATCAGGAATAGCCAACTCCAGCTCACCGGGCAGCTGTACCGATGGGTGGTATAATACATCCAAGAAAACATTGGGTGGGACCGGTTTACGCTGAGAGCCCAAGCGACTTAAAGCGTCTGCTGCCTCATTTTTTCTGCGGTCCACATGATCCACTTGATAGCCTTTGAAATGCCCAGCCACCATATCTACTTCACGTCGGTATGCAGCCATAAGCGGATCCTTAGAATCCCAGGTGCCAGATACTTGCTGAGCCACCAGATCCGAATCACCAAAGCACCTAACTCTGCTCAGATTCATCTCTTTAGCCACCCGAAGACCATGAAGCAAAGCTTCATACTCCGCCGCATTATTTGTACAGGGGAACATTAAACGGAGGACATAACAAAACTTATCTCCTCGTGGGGAAGTTAAAACGACTCCAGCCCCCGAGCCTTCCAATTGTCTTGATCCGTCAAAATGAATGGTCCAATAGGTGTTATCCGGCTTCTCCTCAGGCGCTTGTAACTCCGTCCAATCGTTTATGAAATCGACAAGTGCCTGTGACTTTATGGCTGTTCGGGGTACGTACCTTAAATCGTGCGGCCCGAGCTCGATAGCCCACTTGGCAATCCGACCAGTCGCCTCCCGGTTCTGTATAATGTCACCCAAAGGAGCAGAACTGACCACCGTGATTGGGTGTCCTTGGAAATATTGTCTCAGCTTCCGGCTTGCCATAAAAACTCCATATACCAGCTTCTGCCAATGCGGATACCTTTGTTTGGACTCGATAAGTACTTCACTGATATAGTAAACCGGTCGCTGAACCGGATGTTCCTTTCCAGCCTCTTTTCGTTCTACCACCATCGCCACACTGACTGCTCTGGCATTCGCTGCAACATATAACAGAAGTGGCTCTTTATCCACAGGGGCTGCCAACACTGGCGGATTGACTAGTTGTCGCTTCAAGTCCTCAAACGCTTTATCAGCCTCCGGACTCCAGACAAACTGATCCGTCTTCTTGAGCATTTGATACAAAGGGATCGCCTTCTCACCAAGGCGACTGATAAACCGGCTTAACGCTGCAATCCGCCCGGCCATGCGCTGAACATCATTGATGCATTTCGGTTTGGCCAGGGAGGTGACGGCTGTGATCTTTTCTGGATTGGCCTCAATTCCTCTATGGGACACTAAAAATCCCAACAGCTTGCCTGCAGGTACACCAAAAACACATTTAGCCGGGTTGAGCATCATTTGGTACACTCTCAAGTTATCGAAAGTCTCCTTCAAATCGTCAACCAGGGTCTCCTTCTTTCGTGACTTGACCACAATATCATCCACATAAGCGTGCACATTACGACCAATCTGCTCGTGGAGGCAATTTTGCACACATCGTTGATAAGTAGCCTGCGCACTCTTAAGCCCAAAGGGCATAGACACATAGCAGAAGGCTCCAAAGGGAGTAATGAACGCTGTCTTCTCCTGGTCCTTAACTGCCATCTTGATCTGATGATAGCCCGAATAGGCATCCAAAAAACTTAAACGCTCGCAACCTGCCGTAGCATCAATGATCTGATCAATCCGGGGGAGAGCAAAAGGATCTGCAGGGCAAGCTTTATTCAAGTCTGTGTAGTCCACACACATGCGCCAGGTACCGTTTTTCTTAAGAACTAGCACCGGATTAGCGAGCCATTCGGGATGAAAAACCTCAATGATAAACCCAGCTGCTAGGAGCCTGGCCACTTCCTCACCAATCGCTTTACGTCTTTCTTCGTTAAACCGCCGTAAGAACTGCTTTACCGGCTTGTACTTAGGATCCACATTAAGGGTGTGCTCAGCGAGTTGCCTCGGTACACCAGGCATGTCAGATGGCTTCCATGCAAAGATGTCCCGATTCTCACGGATGAACTCGATGAGCGCGCTTTCCTATTTTGGATCCAAGTTTGCACTGATGCTGAACTGCTTGGATGAATCGCCAGGTACAAAGTCAACAAGCTTAGTTTCGGCCGCTGACTTGAACTTCAAGGTCAGATCGTGGTCCGTAGTTGGCTTTTTTAATGAAGTCATATCCTCCGGATCAACACTGTCTTTGTACTGTTTTAGCTCCTCCGTAGCACAAACCGATTCAGCATAAGTCGCATCTCCTTCCTCGCATTCCAAAGCGATTTGGCGGCTCCCGTGAACCGTTATAGTACCCTTGTACCCTGGCATCTTAAGTTGCAAATATACGTAGTAGGGCCGTGCCATAAATTTTGCATAGGCTGGCCGCCCGAACAAAGCGTGGTACGGACTTTGGATTTTAACCACCTCGAACGTCAGCGTCTCTGATCTGGAATCGTGGCTATCGCCAAATACCACTTCAAGGGCTATCTTACCAACCGGATATGCAGACTTGCCAGGCACCACCCCATGGAACACTGTGTTGGTCGGTCTGAGATCCTTATCTACCAGTCCCATACGACGGAAGGTCTCATAATACAAGATGTTAATGCTGCTCCCTCCGTCCATGAGCACCTTGGTGAGCTTATAACCTCCCACCTGCGGCGCTACCACCAGTGCTAACTGACCCGGATTATACACCCTGGGAGGATGATCTTCTCGGCTCCATATGATAGGCTGCTCCGACCAACGCAGATAGTGAGGCGTGGCCGGTTCGACAGTACTGACTGCCCGCCTCTGAAGCTTCCTATCTCGTTTATCCAAGCTCGTAGTAAAGACATGATACTGTCCACCACTCAATTGCTTCGGGTTGCTCTGATAACCGCTTTGATTCCCTTGATTATTCTGATTGCTTTGGCTGACCTGTCCGCCCGGGTTACCCTGGAACCCTGGACCGGAATTCCCGCCACCGTACCCTGATCCGGAACCGGAACCGCCACCGGAGCTGTGATCATATTGGAAAGTGTTTGAGCTTTTTAACTCCTTCATGATATAACAATCCTTCCAAAGATGGGCTGCTGGTTCCTCTTTTGTTCCGTGCTTCGGACAGGGCTGGTTTAAGAAAAAGTTCAAACGCTCCGGGTTGGGACCGGGATGCTCGCGAAAACTCGGCTGTTTCCCCTTACGCCGCTGGCCTTTAACCTGTGCATTTGCGCTGGCCACCAAGTCCATGCTCCCGTCCGGTTTACGCTTTCCTCCGCCTCCATTACCTGTCGGCCGATGCTGCTGGCCTTTGGTGTTGCCATTCTTCTTTCCCTTCCCTGCTTTGTCATCACCAGAGTCAGGATCCTTGGTACTGTCAGAATCCGCATACTTCACTAAAGCGGTCATGAGCGTTCCTATATCTGTACAGTCACGCTTCATCCGGCCCAGCTTTAGCTTCAAAGGGCCAAACCGACAGTTGCTCTCCAACAGCACCACGGCGGTGTCTGCGTTGATGCGATCTGACGAGTGCAAAACTTCTGATACCCGCCGTACCCAATGGGTAGTTGATTCCCCTTCTTGCTGAACGCAGGCTGCTAGGTCCACTATTGACATTGGCTGCTTGCACGTGTCCTTGAAGTTCTTGATAAACCGGGCGCGTAATTGGGCCCATGAACTAATTGAATTAGGCGGTAAGCTCTTTAGCCAAGTCCGGGCCGTTCCTTCTAACATCATAGTGAAATATTTGGCACACGCTGTGTCATCCACATCCAGCATCTCCATAGCCATTTCATAGCTCTCCACCCATGTCTCCGGAGGCTGATCTGCCGTGTAGTTTGGTACCTTGCGGGGGCCTTTGAAGTCTTTGGGCAGGCGTACATTGCGTAAAGCGGGAACAAGACACGGTACTCCCAAAGAACTGGAAACCACCCCCGGTTCAACCATAGCTGCGGGGAAGATAGGTGTAAGCTGATGAGCCTGATGCTGCGCTGCTAACCCGGCCTCCCGTGCACGGGTGCGATCCACATTCTCGTGAGCATTGTCGACGCCCGCCGGATTGTTGCCGCGGAGCGCTTCACGCCTGTCGTTAGAAGAGACGGCTGGCTCCTCCACGCGCCTACTATAGCTCCTGCTGGGGCGAGGGGTGGAGTGAATCCGATCACGGCTTTGCGAATACGCTTCTTGCTGGGCCAACGCGGTCCTAAGGAGCTCCTTGACCCGGCGTGTCTCTTCATCCTGCGGTGAATCTCCTTCGATCGGAATGGCTTCCAACCGAGCCGCCGCCGCGACGAGGTTATCCATCGGGTTGGAATAATGACCTGACGGCGTTGGCATGGCCGGTGGCGTATCAGTGTTATGACGAGGCAAGTTTATAATCCGATGCTGAACCGGGGCGCCGGCCGTCGCTTCCGCACGGTTTACCCCCGGGGTATTGAAGAGATTTCGAGCGTCGAGGGCGGCGGGCAACCGAGACCGGGATTTTCTCTTCAGGACCTCCTGAGATGCCTTCTGGTCCATCATGAGCCTGTAAGCCTGTGCTTCTAAAGCGGCGCACTCCGCCGTCATCCTGATCTCTTCTGCTGCCAGCTCCGCTTTAGCCCGAGTGATCTGCTCTCTCACTTTGGCAATCTCAGCGTTGTGAACCTCCTGATCCACCGGGTTAGCCTCCGCCATGAGCGCTGCTAGTGCGTCAAACAGGTCTGACAAAACTCGGGCTGGTGAACCGGCAGGGCCTTCTGCCCGCGCAGCCGTTGCTGCTGCCGATCCGGAGATCGCTGCTGCTGCCGTTGATGATCGTGGCGTCGCCTGTGTCCCAGCCATGAAGATCCCAACCCGATAGGGCAGATCAGAGGGGTCCGGAATGCTGTTGCCATCGGAGCCGCCCCCAACCCGATCATCTTGTAGCTGGTAAAGAGATTCAGTCTCCCCGGTTGACGTTTCATCGCCAGAGTAGATGGCGGTTTCGTCACCAGATTCGGATCCTTCCTCGTAGCTTCCCCCGTGGATGACTCCCACGAAGGCGTGCTTCCTGGTCGGTTTAGCCAGGGTCGATCTCGTGCACTGAGCTGTCTCAATGAGGTCTGCGCAGTCGCCTAACTCGGGGCCCGGTTCTCCGATCTTGCCGATGAAAACATGGATGCCGCCAAAGGGGACCCGGTACCCGTACTCGATCGAACCGGCGTCGGGGCCCCAGCCTGTGCTGTCGATGTAGAGTTTTCCGCGGCGGCTCTTGGTCATCCGTCCCACAGCGTAACCCTCGAGTCCTTCAAAGCGGCCCTCCAAGAACCTGAAACCATCTCGCGATAGCCCCACGGTGGGCGCCAAC

TGTCGTGGAATTGTCACGTCAGATGTCCTAGTAAGAGGACTTAGTCGTAGAGCCATCGCAACGGGTTAGCTTGAAGGGGTTAAAGTGGACACAAGGACACGGTTTTTATACTAGTTCGGCCCCTTCGATGAAGGTAAAAGCCTACGTCTAGTTGTGATGGGATTAATGGGTTTCGAGGGCTTAGGGAGCGATATGATTCGCCTAAGCCTCGAGTTCTTCTTCTCTGTCCTCAAACCGCCGTCGGGTCGTCCCCTTATATACACGGGTGACGCCCGTCCGTCTGCAAAGTCTCGACCTCCGGCTTATAAACGTGCCCGGGTCGGTCTCTCTATTCCTATCTTACAGTACAAGTTGGCCCCAGGCCGGTTTACACCATAAACCTTTAAACCGTTTATAGATCTTGGGCCTTTCTACTCCTCCTTGGGCTTTAACATCTCGGGTCTTGTTGACGGTGGTAACCCGGGCCCTGCAGGCCGGTTTACCCCAGATAGCAACATCCCCAACA

GAGACGGTGCAACACATCAACCAAAACCAACCGAATAGCTCGTATCATTGCAGCGTCTCCATCTGTGATGATTGACCTTGGCTTTTTATGACAATTGGCCTTGAGGAATGTCTGGAGCAGCCAC

>Aegilops umbellulata 1-216;

GGCGGCGACGGCGGGTCTCCGGCGAAGACGAAGCGACGAATGGGTGCGGCGATGAGTGGCGGAGGCGTAGGTGAGGTCGGGCGGCGACGGGGCGTCCTCTGCGGGCAGCAACGGTGATGGGAAC

TGTTGGGGATGTTATTATCTGGGGTAAACCGGCCTGCAGGGCCCGGGTGACCGCCGTCAACAGACCCGAGATGTTAAAGCCCAAGGAGAAGTAGAAAGGCCCAAGGTTCATAACCGGTTTAAAGGCTTATGGTGTAAACCGGCCTGGGGCCAACTTGTATTGTAAGATAGGAATAGAGAGACCGACCCGGGCACGTTTATAAGCCGGAGGTCGAGACTTTGCAGACCGACGGGCGTCACCCGGGTATATAAGGGGACGACCCGACGGCGGTTTGGGGGACAGAGAAGAAGAATTCGAGGTTTAGGCGAAGCATATCGCTCCTTAAGCCCTCGAAACCCATCAATCCCATCACAACTAGACGTAGGCTTTTACCTTCATCGAAGGGGCCGAACTAGTATAAAAACCGTGTCCTTGTGTCCACTTTAACCCCTTCAAGCTAACCCGTTGCGATGGCTCCACGATTAAGTCCTCTTCCTAGGACATCTGCCGTAACAAATCCACGACA

GTTGGCGCCCACCGTGGGGCTATCGCGAGATGGTTTCAGGTTCTTGGAGGGCCGCTTTGAAGGACTCGAGGGTTACGCTGTGGGACGGATGACCAAGAGCCGCCACGGAAAACTCTACATCGACAGCACAGGCTGGGGCCCCGACGCCGGCTTGATCGAGTACGGGTACCGGGTCCCCTTTGGCGGCATCCATGTTTTCATCGGCAAGATCGGAGAACCGGGCCCCGCGCTAGGCGTCTGCGCCGACCTCGTCGAGACGGCTCAGTGCACGAGATCGGCCCTGGCTAAACCGGTCAGAAAGCACGCCTTCGTGGGGGTCATCCGCGGGGGAAGCTACGAGGAAGGATCCGAATCTGGTGACAAAACCGCCATCTACTCCGGCGATGAAACGTCGACCGGGGAAACCGAATCTCTTTACCAGTTACAAGATGATCGGATTGAGGGCGGTTCCGATGGCAACAGTATTCCGGACCCCTCGGATCTGCCCTGTCGGGTTGGGATCTTCATGACTGGAACACAGGCAGCGCCACGATCATCAACTGTAGCAGCAGCGATCTCCGGATCGGCAGCAGCAACGGCTGCGCGGGCAGGAGGCCCTACAGGTTCACCAGCCCAAGTTTTGTCAGACCTGTTTGACGCACTAGCAGCGCTCATAGCGGAAGCCAACCCGGTGGATCAGGAGGCTCACAATGCTGAGATTGCCAAAGTGAGAGAGCAGATCGCTCGGGCTAAAGCGGAGCTGGCAGCAGAAGAGATCAGGATGACGGCGGAGCGCGCCGCTTTAGATGCACAGGCTTACAGGCTCATGATGGACCAGAAGGCGTCTCAGGAGGTCCTGAAGAGAAAATCCCGGTCTCGGTTGCCCGCCGCCCTCGACGCTCGAAACCTCTTTAACACCCCGGGGGTAAACCGTGCGGAGGTGACGGCCGGCGCCCCGGTTCAGCACCGGATTATAAACCTGCCTCGTCATAACACTGATACGCCACCGGCCATGCCAACGCCGTCAGGTCATTATTCCAACCCGATGGATAACCTCGTCGCGGCGGCGGCTCGGTTGGAAGCCATTCCGATCGAAGGAGATTCACCGCAGGATGAAGAAACACGCCGGGTCAAGGAGCTCCTTAGGACCGCGTTGGCCCAGCAAGAAGCATATTCGCAAAGCCGTGATCGGATTCACTCCACCCCTCGCCCCAGTGGGAGCTATAGCAGGCGCGTGGAGGAGCCGGCCGTTTCAAGTAATGCCAGGCGTGAAGCGCTCCGCGGCAACAATCCGGCGGGCGTTGACAATGCTCATGAGAATGTGGATCGCACCCGTGCACGGGAGGCCGGATTGGCAGCGCAGCATCAGGCTCATCAGATTACACCTATCTTCCCAGCAGCTGCGGCTGAACCGGGGGTGGTTTCCAGTTCTTTGGGAGTACCGTGCCTTGTCCCCGCCTTGCGCAACGTACGCCTGCCCAAAGACTTCAAAGGCCCCCGCAAGGTACCAAACTACACGGCAGATCAGCCTCCGGAGACGTGGGTGGAGAGCTACGAAATGGCTATGGAGATGCTGGATGTGGATGACGCGGCGTGTGCCAAGTATTTTACTATGATGTTAGAAGGAACGGCCCGGACTTGGCTAAAGAGTCTGCCACCTAATTCAATTAGCTCGTGGGCCCAATTGCGCGCCCGGTTTATCAAGAACTTCAAGGACACGTGCAAGCAGCCAATGTCAATAGTTGACCTAGCAGCCTGCGTTCAGGAAGAAGGGGAATCAACTACCCATTGGGTACGACGAGTATCAGAAGTCTTGCACTCATCAGATCGCATCAACGCAGACACCGCTGTAGTACTGTTGGAAAGCAACTGTCGGTTTGGCCCTTTGAAGCTAAAGCTGGGCCGGATGAAGCGTGATTGTACAGACATAGGAACGCTCATGACCGCTTTAGTGAAGTATGCGGATTCTGACAGTACCAAGGATCCTGACTCTGGTGATGACAAAGCAGGGAAGGGAAGGAAGAATGGCAACACCAAAGGCCAGCAGCATCGACCGACAGGTAATGGAGGCGGAGGTAAGCGTAAACCGGACGGGAGCACGGACTTTGTAGCCAATGCAAGTGCACAGAATAAGGGCCAGCGGCGCAAGGGGAAACAGCCAAGTTTCCGCACAGATCCTGGTCCTAATCCGGAGCGTTTGAACTTTTTCTTAAACCAGCCCTGTCCGAAGCACGGAACGAAGGAGGAACCAGCAACCCATCTTTGGAAGGATTGTTATATCATGAAGGAGTTCAAAAGCTCAAACACTTTCCAGAATGATCGCAGCTCCGGCGGCGGTTCAGGTCCCGGTCCAGGGTTCCAGGGTAATCCGGGCGGACAGGTCAGCCAAAATAATCAGAGTAATCAGAGTGGTTATCAGAGCAACCCGAAACAGTTGAGCGGTGGACAGTATCATGTCTTTACTACAAGCCTGGATAAACGGGATAGAAAGCTTCAGAGGCGAGCAGTTAGTGCTATCGAACCAGCCACGCCTCACTATCTGCGCTGGTCAGAACAGCCCATCATATGGAGCCGAGAGGATCACCCTCCCAGGGTTTATAATCCGGGTCAGTTAGCATTGGTGGTGGCGCCTCAGGTGGGAGGTTATAAGCTCACCAAGGTGCTCATGGACGGAGGGAGCAGCATTAACATCTTGTATTACGAGACCTTCCGTCGTATGGGACTGGTAGATAAGGATCTCAAACCGACCAATACAGTGTTCCACGGGGTGGTGCCTGGCAAGTCTGCATATCCGGTTGGTAAGATAGCCCTTGAAGTGGTATTTGGCGATAGTCACGATTCCAGATCAGAGACGCTGACGTTCAAAGTGGTTAAAATCCAAAGCCCGTACCACGCTTTGTTCGGACGGCCAGCTTATGCAAAATTTATGGCACGACCCTGCTACGTGTATATGCAACTTAAGATGCCAGGTTACAAGGGCACTATAACGGTTCATGGGAGCCGTCGAATCGCTTTGGAATGCGAGGAAGGAGATGCGACTTATGCAGAATCGGTTTGTGCTACGGAGGAGCTAAAGCAGTACAAAGACAGTGTTGATCCGGAGGATATGACTTCATTAAAAAAGCCGACTACGGACCATGATCCGGCCTTGAAGTTCAAGTCAGCGGCCGAAACTAAACTTGTTGACTTCGTACCTGGCGATTCATCCAAGCAGTTCAGCATCAGTGCAAACTTGGATCCAAAATAGGAAAGCGCGCTCATCGAGTTCATCCGTGAGAATCGGGACATCTTTGCATGGAAGCCATCTGACATGCCTGGTGTACCGAGGCAACTCGCTGAGCACACCCTTAATGTGGATCCTAAGTACAAGCCGGTAAAGCAGTTCTTACGACGGTTTAATGAAGAAAGACGTAAAGCGATTGGTGAGGAAGTGGCCAGGCTCCTGGCAGCTGGGTTTATCATTGAGGTTTTTCATCCCGAATGGCTCGCTAATCCGGTGCTAGTTCTTAAGAAAAACGGCACCTGGCGCATGTGCGTGGACTACACAGACTTGAATAAAGCTTGCCCAGCAGATCCTTTTGCTCTCCCCCGGATTGATCAGATCATTGATGCTACGGCAGGTTGCGAGCGTTTAAGTTTTTTGGATGCTTATTCGGGTTATCATCAGATCAAGATGGCAGTTAAGGACCAGGAGAAAACAGCGTTCATTACTCCCTTTGGAGCCTTCTGCTATGTATCTATGCCCTTTGGGCTTAAGAGTGCGCAGGCTACTTATCAACGATGTGTGCAAAATTGCCTCCACGAGCAGATTGGGCGTAACGTGCATGCTTATGTGGATGATATCGTGGTCAAGTCACGAAAGAAGGAGACCCTGGTGGACGATTTGAAGGAGACTTTCGATAACTTGAGAGTGTACCAAATGATGCTCAACCCGGCTAAATGTGTTTTTGGTGTACCTGCAGGCAAGCTGTTGGGATTTTTAGTGTCCCATAGAGGAATTGAGGCCAATCCGGAAAAGATCACAGCCATCACCTCCCTGGCCAAACCGAAATGCATCAATGATGTTCAGCGCATGGCCGGGCGGATTGCGGCGTTAAGCCGGTTTATCAGTCGCCTTGGTGAAAAGGCGATCCCCTTGTATCAAATGCTCAAGAAGACGGATCAGTTTGTCTGGAGTCCGGAGGCTGATAAAGCGTTTGAGGACTTAAAGCGGCAGCTAGTCAATCCGCCAGTATTAGCGGCCCCTGTAGATAAAGAGCCACTCCTGTTATATGTTGCAGCGAATGCCAGAGCAGTCAGTGTGGCGATGGTGGTAGAACGAAAGGAGGCTGGAAAGGAACATCCGGTTCAGCGGCCGGTTTACTATATCAGTGAAGTACTTATCGAGTCCAAACAAAGGTATCCGCATTGGCAGAAGCTGGTATATGGCGTTTTTATGGCAAGCCGGAAGCTGAGGCAATATTTTCAAGGACACCCAATCACGGTGGTCAGTTCTGCTCCTTTGGGTGACATTATACAGAACCGGGAGGCGACCGGTCGGATTGCCAAGTGGGCTATCGAGCTCGGGCCGCACGATTTAAGGTACGTACCCCGGACGGCCATAAAGTCACAGGCACTTGTCGACTTCATAAACGATTGGACAGAGTTACAAGCGCCTGAGGAGAAGCCAGATAACACCTATTGGACCGTTCATTTCGACGGGTCAAGACAATTGGAAGGCTCGGGGGCTGGAGTCGTTTTAACTTCCCCACGAGGAGATCAGTTTTGTTATGTCCTCCGTTTAATGTTCCCCTGTACAAATAATGCGGCTGAGTATGAGGCTTTGCTTCATGGTCTTCGGGTGGCTAAAGAGATGAATCTGAGCAGAGTTAGGTGCTTTGGTGATTCAGATCTGGTGGCTCAGCAGGTATCTGGCACCTGGGATTCTAAGGATCCGCTTATGGCTGCATACCGACGTGAAGTAGATATGGTGGCTGGGCATTTCAAAGGTTATCAAGTGGATCATGTGGACCGCAGAAAGAATGAGGCAGCGGACGCTTTAAGTCGCTTGGGCTCTCAGCGTAAACCGGTCCCCCCCAACGTTTTCTTGGATGTATTATACCACCCATCGGTACAGCTGCCCGGTGAGCTGGAGTTGGCTGTTCCTGATCCGGAGGCTCAGTTAGTGGCGGCTCTTCACGCCACCCCGGATTGGACGATCCCTTACCTGGCATATATGAACCGGGGTGAGTTGCCAGAGGACGAAAGCTTGGCTCGACAGATAGTACGACGGTCCAAGTCTATGACTATTTTCCAAGGAGAGTTACATCATCGCAGCGTGTCAGGAGCGCTGCAACGGTGCATATCCCCTAAGGAGGGGTGCGAGATACTACGAGAAATACATGAAGGGGATTGCGGCCACCACGCCGGTTCAAAATCATTGGTGGCTAAAGCGTTCCGCCACGGTTTCTACTGGTTAACTGCTCATGCTGATGCAGAAGACCTGGTCCGGTTATGTGATGGTTGCCAGAAATTTTCCAGACGAGCGCACATACCGGCTCAAGAATTGAGGATGATTCCAATCACTTGGCCGTTCGCGACCTGGGGGCTGGATATGGTTGGGCCTTTTAAGCGTTCCAAGGATAAGAAGACCCACCTACTGGTGGCGGTTGACAAATTTACAAAGTGGGTAGAGGCAGAACCTGTCAGTAAGTGTGATGCTGCCACGGCGGTTCAGTTTATAAAGAAGGTGATCTTCCGGTTTGGATTTCCACACAGCATCATCACAGATAATGGTACCAACCTATCCAAGGGGGCCATGAAAGAGTTCTGTGCACGGGAGCATATACGGCTTGATGTTTCTTCGGTGGCGCACCCACAGTCTAATGGTCAGGCAGAACGAGCGAACCAAGAGATCTTGAGAGGTATCAAACCCCGGCTTCTGGTCCCTTTGCAAAGGACGCCGGGTTGTTGGGTGGAGGAACTACCGTCTGTGTTATGGAGCATCAACACCACGCCTAACAGATCCACGGGGTTTACGCCGTTTTTTATGGTTTATGGAGCAGAGGCGGTTCTCCCAAGTGATATACGACATGACTCACCTCGCGTGGCAGCATATGTTGAAGCGGATAATGAGCAGGCACGGCAGAACGCTCTTGACTTGTTGGATGAGGAGCGTGACTTGGCAGCTGCCCGTTCAGCGATTTACCAGCAAGATCTTCGCCGTTACCACAGCCGTCGGGTTAGGACCAGAACCTTTCAGGAGGGGGATTTGGTGCTTCGGCTCATCCAGGATCAGTCTGATCAGCATAAATTATCCCCGCCTTGGGAAGGACCTTTTGTGGTCAGCAAGAATCTGAATAATGGGTCATATTACCTGATTGATATTCGAGAGCGCAAGGATTCACGCACATCAGAGGAGGAGACCAGCAGGCCGTGGAACATAGCTCATCTACGGCCTTATTATACATGAGCCCTGGACTCTGCTTATGTACATATCATGACCATGTATATATTATGATTAATACAATAAACCGGAGCCTCGAGTAAAGCGGGGTCTCTGCTGTTCTTCACATCATGTGTGTTTGCCCCTGGAGGTTGCTTCACAAAGTTCAAGATATAGATTCCGGTTTAAAAGCCGGTTCAAGGGAAGATGTCTCCGGCCAGGCTGTGCAGCTCTTAATATCCGGTTCAATATCCCGGTTCAAACATGAGGCTTCCTGTTCAAACATAGGTCGTATTCGAACCAAAGAGAACATAGCTATTCAAACATGAGGCTTCCTGTTCAAACATAGGTCGTATTCGAACCAAAGAGAACATAGCTATTCAAACATGGGGCTTCCTGTTCAAACATAGGTCGTATTCGAACCAAAGAGAACACAGCTGTTCAAACATAGGTCGTATTCGAACCAAAGAGAACGTAGCTATCCCTATAAAGTCAGTTGGGGACCTAGTCGGCCTGAACCGTGGCTACACCTTCGGGGGGCTTGGTCATGTCTTGACGACGGTAATGCCTTCTGGCTGGCACATTTGCCATTGAATCAGTTGGGGACCTAATCGACTTGAACCGTAGATTACGCCTTAGGGGAACTCGGTCATGTCTTGATAATGGTAATGCCTTCTGGTCGGCTTTTTAGCCAATGAATCATTTGGGGGCTTGGTCGTGTCCGAACCAACGCAATGCCACTTGATCGGCCCTTGGCCATTGAGTCACTTGGGGGCTTGGTTGACGAACCAATGCAATGCCATTTGGCCGGCATATTTGCCACGATTCAGTTAGGAACCTAGTCGGCCTAAACCGTAGCTACACCTCTTGGGGGCTCGGTTTTGTCTTGACGATGGTCACGCCTACTGAACGGTTCATAGTAACCACTATTCGCATGCGTTTATTATCGCTTTGCCTTTTCATATTTGGTTTGGTTTTTCTGCCTTGATTGTGGTGTCTTTTTTAGTTCTTCGGAACGTCAAGCATCCTTAAACCGATTCAGCTGGCGTAGCCTGGTCCGTTTTTTTTTTAACCCGGAGGCAAATTGCCCGGTTTGGCAAAGGCGCAGCACGGTAGTCGAAAAATCGCATTGTGTACTCAGTAAAGGCAGAAAACATAAGATGGAAACATCATTGGAAAGAGCATCAGTACCCGTGCGCGAAGGCACATTCAAATGCAGAAGTATTAAACTATCTTATTACAAGGCAACGTAGTGCCTGAATATATTGTTCGCTGACGGATTATAGCTTCGCCAAAGGTGAACCGGAGCACTGATTACGCCTGTTCGCCGCCTCGGTTCGAAGGCTGGGGATCGTCTTGCGCCGCTTCAGCTCCATCTTGGTTACCCATTGGCTGGAAATCAGCAGTGGTCCAGTCGATTCCCATTAGCGCTTGAAAGACAGCTTCTTCATGAATCAGCGAAGACGGTTCAATGTCTGGGGCGTAAGTATGCTTACGGATTGGAGGAACAAGGTTTCCCGCTTCAGGGACCGGGGCAGATACTCGTTTGTTCTGGTTGTCGTATTGGGCTTGGTAACGTGACAGATCTGCTTCCTCAGCCAATTGACAAGCTAGGGGGCGCACCGCCCGGTTTATCGCCCTCAAATCGTCTTCACCAAAGTCTGAACCGTCCTCCTTCAAGCTGGGGTATCCTTGGGCCGCTTCGACAGGATCGAAATCTGGTACCCACGCTTTGGCCCGGATCAAGGCAGTAATGGCTCCAGTTCGAGCAGCTGATCTTTTCAATTCTTCAATCCGGGCTGGAAGCATGGACAACCTCTTTAATGTGTCTTGAATCAGAGACGGCGCAGGGTTGTTATGCGATGCAGTGGTGATAGCTCTTTGTGCTCCGGTGTATAACTGTTCAATCAGAGTATAAGCAGCTTTGAGTTTCATCCGCACGTCAGAACCCAAGTGTCCAATACATGAGCCTGCAGTGTTTCAAGGTGGATTACAAACCGGACATAGGGTGTGGCAAACAAGGTACAAATTGGAAGAAGGGCATCGAAAAAGTTTACCAAAGACGGCAGAGGTCATGGAGTGTATTTGTCGCTTCACGGTGGATAACTCATCAACCACCGGTTTAAGAGCAGCTTCGGCATTTTCAGCCCTCTTCGTCAAAAGAGCTTTTTCAGTAGCCCAATCGGCTTGCTCCTTTTTGCGGCTTGCTTTCAGCTGCTCCATAACAGTTAAGGCTTTGGTCAATTCCTCCTTTGCTTTTTCAGTTTCAATCTGCTGGGCTTTTAAAGCTTCCTGAAGGTCCGAGACTTGGTTCTCTTTGGTCTTCAAATCCCCCTGCAGCAGGTCATCAACAGTGTTCAGAAAATATTGCACGGTTTGGAAGTATCAACCACACAAGCAAGTTATGTGCTCGATACTTGGGGGCTAATGCATATTTGATTTTAACATAAGTTTTCCATTACAAAAAGTCCCAAGATTAATACAAGTATTTAAGCTTGGCACTTGGGGGCTAATGGTTATCTGTTGATGTTTTTCTGGTTATGACCGATGTTGAAACCTTGGGTTTTCTAAACCAGACCTTAATTGTCAAAAGCAGCCTGGTCCGGACTATCATCTCGGTTTGGAAGTATTTGATAAGCTAAAGTTGCAATAGAATGAAGCACAAGGGAGTTACCTCAAGTTTTTCCTTCATCATCTTGATCATAGCAGCCTCATAATCCCGACTGGTGTACAGCCGGTTCAGATACCCAGAATAAAGATCCGGGGCGCTTAAAGAGGCGTACGTAGATAAGTCAAGGTCCCATTTGCCCTTTATGGCCGAGACTTCGTCCTTGGCAGTATGCTTGGCCAAAGTAGCAGTGTTCCCCGGTTCAGAGCGGCCAATACCGGTAATCACGACATCATCATCCTTGGAGTCGCTGCTCTGCAGTGGCCCTGTCGGTTTATCAGCAGTTGCAGAAGGGGGGATGGATTCCTCAACCCGAACAGGACTGGCAGGCGGATCAGTACGGCTAGCAGGTTCGGTAGTCCTTTCTTCACCAAAGATGTCATCTTGCGGAGATAGAAAGTTCTGAGTATCTTCACGATCCAGAGCGTCAGCGCCAGGAGTTTTCTCCGGTTCAGGAGCGGCAGTTCCTTCAGCCGGGTTATCCAGACGAGCCTTCTTGCTCGGTCTAGGTTTAGCCCTGGAAAAACCCAAAGATTGGTAAAACAGTATAAGATGGATTAAGAGACCCATACAGAGATATGTTGTGATCGGTCCAACTTACCCAATAACTGTCTTCAACGGTGGCAGTTGAGTAGCTGAAGATTCACCGGAAGATGAATGGGAAGTGACCTGATAATCGGAGTCAGATGGATTTAGAGGCTGGCGAGTTAAACCGGCTTTAGGAGGAAAGCTAGCGCATAAATCGGAGACCTCTGAACGGCGTTTCCGAACCGGGCTGTTCGGTAAACCGGCGGAGGTGACTACCTGGCCGCTATGACGGGTGGTGCGTCGAGCTTCATGTTGCTGGGTCTTCACAAGAAATTTGGGATCCAAGTAAGCAAGAGGGTGAGAAAATTTAATTTTCCGATTTGCCTGGCGAGGTTTTTTCAAAGGCAAAGGAACGGCATCAGAAGAAAGAATAGTTACCTCTGGGCCTTCGGCATGGCTCACTTCAGCGTCATCCTGACAAATATCATCATCAATAAGACGTACAAAAAGCAAACCAACTGAATCGAGGTCTACCCCGAGGTCCGGATTACCCACATCATCCTCCTCTAAATCCAGAGTATTGGAAGCGGTGTTCCTTTTCCTCTGAGCAGGCTTCGTCACCCTCGTCTTGGGTCGGGTTACCTTTTCGGTCGGCTCTTGTAACTTCTTGCTCCAAAATGGATCATCTCCCTGTCTCAGGAAGGACTGGAGTTAACAACATATATATAAGGAGATCAAAGATTCTATAAACAGAGGAATTACTTACAGCAGGTGGTTTGTTTGTGGCACAAAATGGAAGCAGGCCGGTTTGAGCGCAAACAGGTTCCGGTTCGTTCAGCATCTTCTTCACAGCTTCAGCGACTTCGTCCTCCGTCAGCTGAATGTTGCAGTGCCTCAGAGGGTCATCAACCCGACCCGTGTATTGGCACATCAAACCGGAGCGAATGCTAAGGGGCAGGATGCTCCATGAAATCCAGCAACGAGCAAGATCAACCCCCGTTAAACCATTAGCCATGAAGGCTCTGAGCTTGGACAGCTGAGGGGCATACTTACTCCTCTCTTTGGCAGTTAGCCGTGGGGGGAAGGGGTGAGTGTTGCTAAGTCTCTCTGGACGGAAACCGGGCAGTGGATTTTCATCGGCAGGGGAAGTATCCTTGCAATAGAACCAAGTACAATTCCATTCTTGCGGATGACTATGCAACTTGGCGTGAGGATAAGTCACCTCTTTCCTTTTCTGAATCGCCATACCACCCAGTTCCATATTAGGGCCGTCGGTAAATTCAGTACGTCGGTTCAAATGGAAAAAGTCCCGGAACAGCTCTGCAGTAGGTTCTTCTTGGAAGTACGCCTCGCAAAGTACTTGGAAATGGCAGATATTCGTGACTGAATTTGGACCAACATCTTGTGGGCGCAGCCTAAAATTAGCCAAGACATCCCGATAAAATTTTGAACCGGGTGGATTAAACCCCCGGGCCAGGTGATCCGCAAACACCACCACTTCTCCCTCCTGCGGGGTAGGAGGACATTCGTCTCCTGGAACCCTCCAATGGATGATACTTTTGCTACTCAAAGCACCAGTCAGAACTAAGTTGTTTAGATGGGTCTCAGTGATGCAAGAGGGAACCCAGTTGCATTCATACACCTGTTTTGCCATGAGGAAATCTACAAGGCAAAGGTATTCCGGTTTGAGAGCAAGTATGATAGTAAACCGAAGATGCTCTTATATAGAGTCAGGTTATCTACAGTAATATCAACCGGCGGTTCATCAAGGGGACTAATGGATGTGTAGATCGGTTTGTTCGCTTTGCTAAGAGTTAAACCGGCCAGATCTAAACGGTTGTGAGTATGAAAGCAGAAACAGAGCCCGTACGGATTCTTTCAAAACAATGGATCTACAGCTAGGGCGGAAAAAGAAATAAAAACAGGTAATATGAAGTCGGGCTCACAGCAGAACAACATAAGTCCGACAAGCAGGAACAAGAGTGAGATCTAAGGAAATGAGTGCAGATTTATGGCGACTGCTAAGAGGAACTACAAATCCAGATACAGGTAGCATTATCTGTTCGCAAGGGCTGCTATGGAGATGAGCAAGAGACAGTAGCAGAACGATGAACCCTAGAACAGATCTAAAGCAACAAGAACAGGGGCCTTACCGGGGTCCAAACAGATGCGGAAAATCGCCGCGGTGCTCTGATCCGATCAGGTTGATGCAGCGGCCGGAGTTGGTGCAGCAGCTGACGGCGGCGGCGGAGCTCTGAAGCTTGGGTCGCGAGGAAGACGAAGAAGGGGAGGAAAGGAAGATGGCCTTCAAGTCTTATTTATAAGGTGCGGCGCGTGTGTCAGGCGCGCGAATCCAGGGGTCGGAGATTTCGGAAGCCCAGCCGTCGCCCTAATTCGTGCGGGTTAGCGAAAGGAGATGGTAGCCGGCGCTGACGTCATATCGGATTACCACAACAAGAGGAGACGGAGAAACAACGGTTTAAACAAGTCACTGGAAGACATTTGAAGACAGAGTCTTATTAAGGATTGACATGAACCTGTTCAAATCAATCTGGGGCCTAA

TGTTGGGGATGTTATTATCTGGGGTAAACCGGCCTGCAGGGCCCGGGTGACCGCCGTCAACAGACCCGAGATGTTAAAGCCCAAGGAGAAGTAGAAAGGCCCAAGGTTCATAACCGGTTTAAAGGCTTATGGTGTAAACCGGCCTGGGGCCAACTTGTATTGTAAGATAGGAATAGAGAGACCGACCCGGGCACGTTTATAAGCCGGAGGTCGAGACTTTGCAGACCGACGGGCGTCACCCGGGTATATAAGGGGACGACCCGACGGCGGTTTGGGGGACAGAGAAGAAGAATTCGAGGTTTAGGCGAAGCATATCGCTCCTTAAGCCCTCGAAACCCATCAATCCCATCACAACTAGACGTAGGCTTTTACCTTCATCGAAGGGGCCGAACTAGTATAAAAACCGTGTCCTTGTGTCCACTTTAACCCCTTCAAGCTAACCCGTTGCGATGGCTCCACGATTAAGTCCTCTTCCTAGGACATCTGCCGTAACAAATCCACGACA

GGAACGGCGGTGTGCTCCGGCGGCGGCGGTGTTCGGCTACGTCTCCGGCGACAAAAAGGTGTTAGAAGAGTGCAGCGCTGAAGGGGGAATTGAACGGAGCAGGCGGTGAGGCTCGGCGCGTTCT

>Aegilops umbellulata 1-229;

TTGCTGTCATCTCTATTCGTATCGTGCTACTGTCATGAGAAACTCCTTTATCTTTGCACGTTAAAGTTTTGCAACCTATGATAAATGGCAATTCTTTGAGTGTTGAGTTGAGCTAATTGGCACT

TGTTGGGGATGTTGCTATCCGGGGTAAACCGGCCTGCAGGGCCCGGGTTACCTCCGTCAGCAGACCCGAGATGTTAAAACCCAAGGAGAGATAGAAGGGCCTAAGGCCCGTAATCGGTTTAAAGGTTTATGGTGTAAACCGGCCTGATGTAAACTTGTATTGTAAGTTAGGAATAGAGAGACCGACCCGGGCACGTTTATTAGCCGGAGGTTGGAACTTTGCAGACCGACGGGCGTCACCCGTGTATATAAGGGGATGACCCGGCGGCGGTTTGAGGAGAGAGAAAAAAGAAGAACTCGAGGTTTAGGCGAAGCTTATCGCTCCCTAAGCCCTCGAAACCCATCAATCCCATCACAACTAGACGTAGGCTTTTACCTTCATCGAAGGGGCCGAACTAGTATAAAAACCGTGTCCTTGTGTCCACTTTAACCCCTTCAAGCTAACCCGTTGCGATGGCTCCACGACTAAGTCCTCTTACTAGGACATCTGCCGTGACAAATCCACGACA

GTTGGCGCCCACCGTGGGGCTATCGCGAGATGGTTTCAGGTTCTTGGAGGGCCGCTTTGAAGGACTCGAGGGTTACGCTGTGGGACGGATGACCAAGAGCCGCCGCGGAAAACTCTACATCGACAGCATAGGCTGGGGCCCCGACGCCGGCTCGATCGAGTACGGGTACCGGGTCCCCTTTGGCGGCATCCATGTTTTCATCGGCAAGATCGGAGAACCGGGCCCCGAGTTAGGCGACTGCGCCGACCTCATTGAGACAGCTCAGTGCACGAGATCGACCCTGGCTAAACCGACCAGGAAGCACGCCTTCGTGGGAGTCATCCACGGGGGAAGCTACGAGGAAGGATCCGAATCTGGTGACGAAACCGCCATCTACTCTGGCGATGAAACGTCGACCGGGGAGACCGAATCTCTTTACCAGCTACAAGATGATCGGGTTGGGGGCGGCTCCGATGGCAACAGTATTCCGGACCCCTCTGATCTGCCCTATCGGGTTGGGATCTTCATGGCCGGGACACAGGCGACGCCACGATCATCAACTGCAGCAGCAGCGATCTCCGGATCGGCAGCAGCAACGGCTGCGCGGGCGGGAGGCCCTACCGGTTCACCAGCCCAAGTTTTGTCAGACCTGTTTGACGCGCTAGCAGCGCTCATGGCGGAGGCCAACCCGGTGGATCAGGAGGTTCACAATGCTGAGATTGCCAAAGTGAGAGAGCAGATCACTCGGGCTAAAGCGGAGCTGGCAGCAAAAGAGATCAGGATGACGGCGGAGCGCGCCGCTTTAGATGCACAGGCTTACAGGCTCATGATGGACCAGAAGGCATCTCAGGAGGTCCTGAAGAGAAAATCCCGGTCTCGATTGCCCGCCGCCCTCGACGCTCGAAATCTCTTCAACACCCCGGGGGTAAACCGTGCGGAGGCGACAGCCGGCGCCCCGGTTCAGCACCGGATTATAGACCTGCCTCGTCATAACACTGATACGCCACCGGCCATGCCAACGCCGTCAGGTCATTATTCCAACCCGATGGATAACCTCGTCGCGGCGGCGGCTCGGTTGGAAGCCATTCCGATCGAAGGAGATTCACCGCAGGATGAAGAGACACGCCGGGTCAAGGAGCTCCTTAGGACCGCGTTGGCCCAGCAAGAAGCGTATTCGCAAAGCCGTGATCGGATTCACTCCACCCCTCGCCCCAGCAGGAGCTATAGTAGGCGCGTGGAGGAGCCGGCCGTTTCAAGTAACGCCAGGCGTGAAGCGCTCCGCGCCAACAATCCGGCGGGCGTCGACAATACTCACGAAAATGTGGATCGCACCCGTGCACGGGAGGCCGGATTAGAAGTGCAGCATCAGGCTCGTCAGCTTACACCTATCTTCCCCGCAGCTACGGTCGAACCGGGGGTGGTTTCCAGTTCTTTGGGAGTACCGTGCCTTGTTCCCGCTTTACGCAACGTACGCCTGCCCAAAGACTTCAAAGGCCCCCGCAAGGTACCAAACTACACGGCAGATCAGCCTCCGGAGACATGGGTGGAGAGCTATGAAATGGCTATGGAGATGCTGGATGTGGATGACACGGCGTGTGCCAAGTATTTTACTATGATGTTAGAAGGAACGGCCCGGACTTGGCTAAAGAGTTTGCCGCCTAATTCAATTAGCTCATGGGCCCAATTGCGTGCCCGGTTTATCAAGAACTTCAAGGACACGTGCAAACAGCCAATGTCAATAGTTGACCTAGCAGCCTGCGTTCAGCAAGAAGGGGAATCAACTACCCATTGGGTACGACGAGTATCAGAAGTCTTGCACTCATCAGATCGCATCAACGCAGACACCGCTGTAGTGCTGTTGGAAAGCAACTGTCGGTTTGGCCCTTTGAAGCTAAAGCTGGGCCGGATGAAGCGTGATTGCACAGATATAGGAACGCTCATGACCGCTTTAGTGAAGTATGCGGATTCTGACAGTACCAAGGATCCTGACTCTGGTGATGACAAAGCAGGGAAGGGAAAAAAGAATAGCAACACCAAAGGCCAGCAGCATCGGCCGACAGGTAATGGAGGCGGAGGAAAGCGTAAACCGGACGGGAGCATGGACTTGGTGGCCAGCACAAATGCACAGGATAAAGGCCAGCGGCGTAAGGGGAAGCAGCCAAGTTTCCGCGCACATCCGGTCCTAATCCGGAGCGTTTGAACTTTTTCTTAAACCAGCCCTGTCCGAAGCACGGAACAAAGGAGGAACCAGCAGCCCATCTTTGGAAGGATTGTTATATCATGAAGGAATTCAAAAGCTCAAACACTTTCCAATATGATCACAGCTCCGGTGGCGGTTCCGGTTCTGGATCAGGGTACGGTGGCGGGAATTCCGGTCCAGGGTTCCAGGGTAACCCGGGCGGACAGGTCAGCCAAAATAATCAGAATAATCAAAGTAATCAAGGTGGTTATCAGAGCAACCCGAAACAGTTGAGTGGTGGACAGTATCATGTCTTTACTACGAGCTTGGATAAACGAGATAGGAAGCTTCAGAGGCGGGCAGTCAGTGCTGTCGAACCGGCCACGCCTCACTATCTGCGTTGGTCGGAACAGCCTATCATATGGAGCCGAGAAGATCATCCTCCCAGGGTGTATAATCCGGGTCAGTTAGCACTGGTGGTAGCGCCTCAGGTGGGAGGTTATAAGCTCACCAAGGTGCTCATGGACGGAGGGAGCAGCATTAACATCTTGTATTATGAGACCTTCCGTCGTATGGGACTGGTAGATAAGGATCTCAAACCGACCAATACAGTGTTCCACGGGGTGGTGCCTGGCAAGTCTGCGTATCCGGTTGGCAAGATAGCCCTTGAAGTGGTATTTGGCGATAGTCACGATTCCAGATCAGAGACGCTGACGTTCGAGGTGGTTAAAATCCAAAGTCCGTACCACGCTTTGTTCGGGCGGCCAGCTTATGCAAAATTTATGGCACGGCCCTGCTACGTATATCTGCAACTTAAGATGCCAGGTTACAAGGGCACTATAACGGTTCATGGGAGCCGCCGAATCGCTTTGGAATGCGAGGAAGGAGATGCGACTTATGCTGAATCGGTTTGTGCTACGGAGGAGCTAAGGCAGTACAAAGACAGTGTTGATCCGGAGGATATGACTTCATTAAAAAAGCCAACTACGGACCACGATCCGGCCTTGAAGTTCAAGTCAGCGGCCGAAACTAAGCTTGTTGATTTTGTACCTGGCAATTCATCCAAGCAGTTCAGCATCAGTGCAAACTTGGATCCAAAATAGGAAAGCGCGCTCATCGAGTTCATCCGTGAGAATCGGGACATCTTTGCATGGAAGCCATCTGACATGCCTGGTGTACCGAGGCAACTCGCTGAGCACACCCTTAATGTGGATCCTAAGTACAAGCCGGTAAAGCAGTTCTTACGGCGGTTTAACGAAGAAAGACGTAAAGCGATTGGTGAGGAAGTGGCCAGGCTCCTGGCAGCTGGGTTTATCATTGAGGTTTTTCATCCCGAATGGCTCGCTAATCCGGTGCTAGTTCTTAAGAAAAACGGCACCTGGCGCATGTGTGTGGACTACACAGACTTGAATAAAGCTTGCCCAGCAGATCCTTTTGCTCTCCCCCGGATTGATCAGATCATTGATGCTACGGCAGGTTGCGAGCGTTTAAGTTTTTTGGATGCCTATTCGGGCTATCATCAGATCAAGATGGCAGTTAAGGACCAGGAGAAGACAGCGTTCATTACTCCCTTTGGAGCCTTCTGCTATGTGTCTATGCCCTTTGGGCTTAAGAGTGCGCAGGCTACTTATCAACGATGTGTGCAAAATTGCCTCCACGAGCAGATTGGTCGTAATGTGCATGCTTATGTGGATGATATTGTGGTCAAGTCACGAAAGAAGGAGACCCTGGTTGACGATTTGAAGGAGACTTTCGATAACTTGAGAGTGTACCAAATGATGCTCAACCCGGCTAAATGTGTTTTTGGTGTACCTGCAGGCAAGTTGTTGGGATTTTTAGTGTCCCATAGAGGAATTGAGGCCAATCCAGAAAAGATCACAGCCATCACCTCCCTGGCCAAACCGAAATGCATCAATGATGTTCAGCGCATGGCCGGGCGGATTGCAGCGTTAAGCCGGTTTATCAGTCGCCTTGGTGAAAAGGCGATCCCTTTGTATCAAATGCTCAAGAAGACGGATCAGTTTGTCTGGAGTCCGGAGGCTGATAAGGCGTTTGAGGACTTGAAGCGACAACTAGTCAATCCGCCAGTGTTGGCAGCCCCTGTAGATAAGGAGCCACTCCTGTTATATGTTGCAGCGAATGCCAGAGCAGTCAGTGTGGCGATGGTGGTAGAACGAAAGGAGGCTGGAAAGGAACATCCGGTTCAGCGACCGGTTTACTATATCAGTGAAGTACTTATCGAGTCCAAACAAAGGTATCCGCATTGGCAGAAGCTGGTATATGGCGTTTTTATGGCAAGCCGGAAGCTGAGGCAATATTTCCAAGGACACCCAATCACGGTGGTCAGTTCTGCTCCTTTGGGTGACATTATACAGAACCGGGAGGCGACCGGTCGGATTGCCAAGTGGGCTATCGAGCTCGGGCCGCACGATTTAAAGTACGTACCCCGAACGGCCATAAAGTCACAGGCACTTGTCGATTTCATAAACGATTGGACAGAGTTACAAGCGCCTGAGGAGAAGCCAGATAACACCTATTGGACCGTTCATTTTGACGGATCAAGACAATTGGAAGGCTCGGGGGCTGGGGTCGTTTTAACTTCCCCACGAGGAGATAAGTTTTGTTATGTCCTCCGTTTAATGTTCCCCTGTACAAATAATGCGGCGGAGTATGAAGCTTTGCTTCATGGTCTTCGGGTGGCTAAAGAGATGAATCTGAGCAGAGTTAGGTGCTTTGGTGATTCGGATCTGGTGGCTCAGCAGGTATCTGGCACCTGGGATTCTAAGGATCCGCTTATGGCTGCATACCGACGTGAAGTAGATATGGTGGCTGGGCATTTCAAAGGCTATCAAGTGGATCATGTGGACCGCAGAAAGAATGAGGCAGCGGATGCTTTAAGTCGCTTGGGCTCTCAGCGTAAACCGGTCCCACCCAATGTTTTCTTGGATGTATTGTACCACCCATCGGTACAGCTGCCCGGTGAGCTGGAGTTGGCTGTTCCTGATCCGGAGGCTCAGTTAGTGGCGGCTCTTCACGCCACCCCGGATTGGACGCTTCCTTACCTGGCATATATGAACCGGGGTGAGTTGCCAGAAGACGAAAGCTTGGCTCGACAGATAGTACGACGGTCCAAGTCTATGACGATTTTCCAAGGAGAGTTACATCATCGCAGCGTGTCAGGAGCGCTGCAACGGTGCATATCCCCTGAGGAGGGGTGTGAGATACTACGAGAAATACATGAAGGGGATTGCGGCCACCACGCCGGTTCAAAATCATTGGTGGCTAAAGCGTTCCGCCACGGTTTCTACTGGTTAACTGCTCATGCTGATGCGGAAGACCTGGTCAGATTATGTGATGGTTGCCAGAAATTTTCCAGACGAGCACACATACCGGCTCAAGAATTGAGGATGATTCCAATCACTTGGCCGTTTGCGACTTGGGGGCTTGATATGGTTGGGCCTTTTAAGCGTTCCAAAGATAAGAAGACCCACCTACTGGTGGCGGTTGACAAATTTACGAAGTGGGTAGAGGCAGAACCTGTCAGTAAGTGTGATGCAGCCACGGCGGTTCGGTTTATAAAGAAGGTGATCTTCCGGTTTGGTTTTCCACACAGCATCATCACAGATAATGGTACCAATCTATCCAAGGGGGCCATGAAGGAGTTCTGTGCACGGGAGCATATACGGCTTGATGTTTCTTCGGTAGCGCACCCACAGTCTAATGGTCAGGCAGAACGAGCGAACCAAGAGATCTTGAGAGGTATCAAACCCCGGCTTCTGGTCCCTTTGCAAAGGACACCGGGTTGTTGGGTGGAGGAGCTACCGTCTGTATTATGGAGCATCAACACCACGCCTAACAGATCCACGGGGTTTACGCCGTTTTTTATGGTCTATGGAGCCGAGGCGGTTCTCCCAAGTGATATACGACATGACTCACCTCGCGTGGCAGCATATGTTGAAGCGGATAATGAGCAGGCACGGCAGAACGCTCTTGACTTGTTGGATGAGGAGCGTGACTTGGCAGCTGCCCGTTCAGCGATTTACCAGCAAGATCTTCGCCGCTACCACAGCCGTCGGGTTAGGACCAGAACCTTTCAGGAGGGGGATTTGGTGCTTCGGCTCATCCAGGATCAATCTGATCAGCATAAATTATCCCCGCCTTGGGAGGGACCCTTTGTGGTCAGCAAGAATCTGAATAATGGGTCATATTACTTGATTGATATTAGAGAGCGCAAGGATTCACGCACGTCAGAGGAGGAGACCAGCAGGCCGTGGAACATAGCTCATCTTCGACCTTATTATACATGAGCCCTGGGCTCTGTTTATGTACATATCATGACCATGTATATATTATGATTAATACAATAAACCGGAGCCTCGAGTAAAGCGGGGTCTCTACTGTTCTTCACATCATGTGTACTTACCCCTGGAGGTCGCTTCACAGAAGTTCAAATTCCGGTTTAAAAGCTGGTTCAAGGGAAGATGTCTCCTACAGGGTTGTGAAGCTCTTAATATCCGGTTCAGTATCCCGGTTCAAGCATAAGGCTTACTGTTCAAACATAGGCCGTATTCGAGCCAAAGAGAACATAGCCATTCAGCATGAGGCTTCCTGTTCAAACATAGGTCGTATTCGAACCAAAGAGAACATAGCTATTCAAACACGGGGATTTCTGTTCAAACATAGGTCGTATTCGAACCAAAGAGAACACAGCTGTTCAAACATAGGTCGTATTCGAACCAAAGAGAACATAGCTATCCCTATAAAGTCAGTTGGGGACCTAGTCGGCCTGAACCGTAGCTACACCTCCGGGGGGCTTGGTCAGGTCTTGACGACGGTAATGCCTTCTGGCTGGCATGTTTGCCACTGAATCAGTTGGGGACCTAATCGACTTGAACCGTAGATTACGCCTTATGGGAACTCGGTCATGTCTTGATGATGGTAATGCCTTCTGGTCGGCTTTTTAGCCATTGAGTCACTTGGGGGCTTGGTTGACGAACCAATGCAATGCCATTTGGCCGGCATATTTGCCACGATTCAGTTAGGAACCTAGTCGACTTAAACCGTAGCTACACCTCTTGGGGGCTCGGTTTTGTCTTGACGATGGTCACGCCTTCTGAACGGTTCATAGTAACCACTATTCGCATGCGTTTTACTATCGCGTTGCCTTTTCATATTTGGTTTTGTTTTCTGCCTTTATTGTGGTGTTTTTTGGTTCTTCGGAACGTCAAGTATCTTTAAACCGATTCAGCTGGCATAGCCTGGACCGTTTTAACCCGGAGGCAAATTGCCCGGTTTGGCAGAGGCGCAGCACGGTTCAGGTGGTCGAAATCTCACATTGTGTACTCAGTAAAGACAGGAAACATAAGTTGGAAACGTCATTGGAAAGAGCATCAGTACCCGTGCGCGAAGGCACATTCAAAGGCAGAGGTATTAAACTATCTTATTACAAGGCAACGTAGTGCCTGAATATATTGTTCGTTGACGGATTAAGACGTCACCAAGTAGGGTGAACCGGAGCGTTGATTACGCCTGTTCGCCGCCTTGGTTCGAAGGCTGGGGATCGTCTCGCGCCGCTTCAGCTCCATCTTGGTTACCCATTGGCTGGAAATCAGCAGTGGTCCAGTCGATTCCCATTAGCGCTTGAAAGACGGCTTCTTCGTGAATCAGCGAAGACGGTTCAATGTCTGGGGCGTAAGTATGCTTACGGATTGGAGGAACAAGGTTTCCCGCTTCAGGGACCGGGGCAGATACTCGTTTGTTCTGGTTGTCGTATTGGGCTTGGTAACGTGACAGATCTGCCTCCTCAGCCAATTGACAAGCTAGGGGGCGCACCGCCCGGTTTATCGCCCTCAAATCATCTTCACCAAAGTCTGAACCGTCTTCCTTCAAGCTGGGGTATCCTTGGGCCGCTTCGACAGGATCGAAATCTGGTACCCACGCTTTGGCCCGGATCAAGGCAGTGATAGCTCCGGTTCGAGCAGCTGATCTTTTCAACTCTTCAATCCGGGCCGGCAGCATGGACAGCCTCTTTAATGTGTCTTGAATCAGAGCCGGCGCAGGGTTGTTGTGCGATGCAGTGGTGATAGCTCTTTGTGCTCCGGTGTATAACTGCTCAATCAGAGTATAAGCAGCTTTGAGTTTCATCCGCATGTCAGAACCCAAGTGTCCAATACGTGAGCCTGCAGTGTTTCAAGGTGGATTACAAACCGGACATAGGGTATAGCAAACAAGGTACAAATTGGAAGAAGGGCATCGAAAAGTTTACCAAAGACGGCAGAGGTCATGGAATGTATTTGTCGCTTCACGGTGGATAATTCATCAACCACCGGTTTAAGAGCAGCTTTGGCATTTTCAGCCCTCTTCGTTAAAAGAGCTCTTTCAGTAGCCCAATCGGCTTGCTCCTTTTTGCGGCTTGCTTTCAGCTGCTCCATAACAGTTAAGGCTTTGGTCAATTCCTCCTTTGCTTTTTCAGTTTCAATCTGCTGGGCTTTTAAAGCTTCCTGAAGGTCCGAGACTTGGTTCTCTTTGGTCTTCAAATCCCCCTGCAGCAGGTCATCAACAGTGTTCAGAAAATATTGCACGGTTTGGAAGTATCAACCACACAAGCAAGTTATGTGCTCGACACTTGGGGGCTAATGCATATTTGATTTTAACGCAAGTTTTCCATTGCAAAAGGTCCCAAGATTAATACAAGTATTTAAACTTGGCACTTGGGGGCTAATAGTTATCTAATGATGTTTTCTGGTTATGACCTAAGTTGAAGCCTTGGGTTTTCTAAACCGGACTTTAATTGTCAAGACAACCTGATCCGGGTTTACCGTCTCGGTTTGGAAATATTGATAAGCTAAGTTGCAATGGAATAAAGCACAAGGGAGTTACCTCAAGTTTCTCCTTCATCATCTTGATCATGCCAGCTTCATAATCCCGACTGGTGTACAATCGGTTCAGGTACCCGGAATAAAGATCCGGGGCGCTTAAAGCGGCGTACGTAGAAGAGTCAAGATCCCATTTGCCCTTCATGGCCGAGATTTCGTCCTTGGCAGTATGCTTGGCTAAGGTAGCAGTGTTCCCCGGTTCAGAGTGACCGATGCCGGTAATCACAACATCATCGTCCTTGGAATCGCTGCTCTGCAGTGGCCCTGTCGGTTTATCAGCAGTTCCAGAAGGGGGGATGGATTCCTCAACCCGAACAGGACTGGCAGGCGGATCAGTATGGCTGGCAGGTTCGATAATCCTTTCTTCAGCGCAGGTGTCATCTTGCGGAGATAGATAACTCTGAGTGTCCTCACGACCCGGAGCGTTAGCACCAGGAGTTTTCTCCGGTTCAGGAGCGGCAGTTCCTCCAGCCGGGTTATCCAGACGAGCCTTCTTGCTCGGTCTAGGTTTGGCCCTGGAAAAAGCCCAAAGATTGAAAAAACGGTATAAGATGGATTAAGAAACCCATACAGAGATATGTTGTGATCGGTCCAACTTACCCAATAACTGTCTTCAACGGTGGCAGTTGAGTGGCTGAAGATTCACCGGAAGAGGAATGGGAAGTGACCTGATAATCGGAGTCAGATGGATTTAGAGGCTGACGAGTTAAACCTGCTTTAGGAGGAAAGCTAGCGCGTGAATCGGAGACCTCTGAGCGGCGTTTCCGAACCGGGCTGTTCGGTAAACCGGCGGAGGTGACTACCTGGCCGCTATGTCGGGTGGTGCGTCGAGCTTCATGTTGCTGGGTCTTCACAAGAAATTTGGGATCCAAGTAAGCAAGAGGGTGAGAAAATTTAATTCTCCGGTTTGCTTGGCGAGGTTTTTTCAAAGGCAAAGGAACGGCATCAGAAGAAAGAGTAGTTACCTCTGGGCCTTCGGCATGGCTCACTTCAGCGTCGTCCTGACAAATATCATCATCAATAAGACGTATAAAAAGCAAACCAACTGAATCGAGGTCTACCCCGAGGTCCGGATTACCCACATCATCCTCCTCTAAATCCGGAGTAGAGGAAGCGGTGTTCCTTTTCCTCTGAGCAGGCTTCTTCACCCTCGTCTTGGGTCGGGTTACCTTTTCGGTTGGCTCTTGTAACTTCTTGCTCCAAAATGGATCATCTCCCTGTCTCAGGAAGGACTGGAGTTAGCAATATGTGTATAAGGGGATCATAAGTTCTATAACAAACAGAGGGGAGATACTTACAGCAGGAGGCTTGTTGGCGGTACAGAATGGAAGCAGGCCGGTTTGAGCACAAACATGTTCCGGTTCATTCAGCATCTTTTTCACAGCTTCAGCGACTTCGTCCTCCGTCAGCTGAATGTTGCAGTGCCTCAAGGGATCATCAACCCGACCCGTGTATTGGCACATTAAACCGGAGCGGATGCTAAGGGGCAGGATGCTCCACGAAATCCAGCAACGAGCAAGATCTACCCCCGTTAAACCGTTAGCCATGAAGGCTCTGAGCTTGGACAGCTGAGGGGCGTACTTACTCCTCTCTTTGGCAGTTAGCCGTGGGGGGAAGGGGTGAGTATTGCTAAGTCTCTCTGGACGGAAACCGGGCAGTGGATTTTCTTCAGCAGGGGAAGTATCCTTGCAATAGAACCAAGTGCAATTCCATTCCTGCGGATGACTATGCAACTTGGCGTGAGGATAAGTCACCTCTTTCCTCTTCTGAATCGCCATACCACCCAGTTCCATATTAGGGCCATCGGTGAATTCAGTACGTCGGTTCAGATGGAAAAAGTCCCGGAACAGCTCTACAGTGGGTTCCTCTTGAAAGTACGCCTCGCAAAGTACTTGGAAATGGCAGATATTCGTGACTGAATTTGGACCAACATCTTGTGGGCGCAGTCTAAAATTAGCCAAGACGTCCCGATAAAATTTTGAACCGGGTGGATTAAACCCCCGGGCCAGGTGATCCGCAAACACCACCACTTCTCCTTCCTGCGGGGTAGGAGGACATTCGTCTCCTGGAACCCTCCAATGGATGGTACTTTTGCTACTCAAAGCACCAGTCAGAACTAAGTTGTTTAGTTGGGTCTCAGTGATGCAAGAGGGAACCCAGTTGCATTCATACACCTGTTTTGCCATGAGGAAATCTACAAGGCAAAGATATTCCGGTTTGAGAGCAAGTATGATTGTAAACCGAAGATGCTCTTATATAGAGTCAGGGTATCTACAATAATATCAACCGGCGGTTCATCAAGGGGACTAATGGATGTGTAGACCGGTTTGTTCGCTTTGCTAAGAGTTAAACCGATCAGATCTAAACAGTTGTGAGTATGAAAAGCAGAAACAGATCCCGTACAGATTCTTTCAAAGCAATGGATCTACAGCTAAGGCGAAAAAGAAATAAAAAAACAGGTAATATGAAGCCGGGCTCAGAGCAGAACAACATAAGTTCGACAAGCAGGAACAAGAGCGAGATCTAAGGAAATGAGTGCAGATTTATGGCGACTGCTAAGAGGAACTACAAATCCAGATACAGGTAGCATCATCTGTTCGCAAAGGCTGCCATGGGGATGAGCAAGAAACAGTAGCAGAACGATGAACCCTAGAACAGATCTAAAGCAACAAGAACAGGGGCCTTACCGGGGTCCAAACAGATGCGGAAAGTCGCCGCGGTGCTCTGAACCGATCAAGTTGATGCAGCGGCCGGAGTTGGTGCAGCAGCTGACGGTGGCGGCGGAGCTTTGAGGTCTGGGTCGCGAGGAAGACGAAGAAGGGGAGGAAAGGAAGATGGCCTTCAAGTCTTATTTATAAGGCGCGGCGCGCGTGTCAGGCGCGCGAATTCAGGAGCCGGAGATTTCGGAAGCCCAGCCGTCGCCCTGATTCTTGCGGGTTAGCGAAAGGAGACGGTAGCTTGCGCTGACGTCATATCGGTTTACCACAGCAAGAGGAGACGGAGAAACGACGGTTTAACAAGTCACTGGAGGACATTTGAAGACAGAAATTCTTATTAAGGATTGACATGAACCTGTTCAAATCAATCTGGGGCCTAA

TGTTGGGGATGTTGCTATCCGGGGTAAACCGGCCTGCAGGGCCCGGGTTACCTCCGTCAGCAGACCCGAGATGTTAAAACCCAAGGAGAGATAGAAGGGCCTAAGGCCCGTAATCGGTTTAAAGGTTTATGGTGTAAACCGGCCTGATGTAAACTTGTATTGTAAGTTAGGAATAGAGAGACCGACCCGGGCACGTTTATTAGCCGGAGGTTGGAACTTTGCAGACCGACGGGCGTCACCCGTGTATATAAGGGGATGACCCGGCGGCGGTTTGAGGAGAGAGAAAAAAGAAGAACTCGAGGTTTAGGCGAAGCTTATCGCTCCCTAAGCCCTCGAAACCCATCAATCCCATCACAACTAGACGTAGGCTTTTACCTTCATCGAAGGGGCCGAACTAGTATAAAAACCGTGTCCTTGTGTCCACTTTAACCCCTTCAAGCTAACCCGTTGCGATGGCTCCACGACTAAGTCCTCTTACTAGGACATCTGCCGTGACAAATCCACGACA

GCACTGATTAAATAAACTAATATCTCTCTTTAAGCAAGACTACTGTGTTGCTAACTTTACCCATTAAGATAATGAGGGTGCTTCTATTTGTTAGTGTATGTTTCATAAACTAGTCCCACTATTA

>Aegilops umbellulata 1-232;

CTATAGAAAACTTAAATGTCACACCAGCAACTTTGCTTGATTTTGGTGCAACTGCACAAAACATGATTGGATTTCCTATTCGTGTTGGTAGATTCGTATTTTATCTTGGTCGTCTCATGAAAGG

TGTCGTCGGTTTGTCACGGCAGATGTCCTAGTAAGAGGACTTAGTCGTGGAGCCATCGCAACGGGTTAGCTTGAAGGGGTTAAAGTGGACACAAGGACACGGTTTTATACTAGTTCGGCCCCTTCGATGAAGGTAAAAGCCTACGTCTAGTTGTGATGGGATTGATGGGTTTCGAGGGCTCAGGGAGCGATAAGCTTCGCCTAAACCTCGAGTCCTCTTTCTCTGTCCTCAAACCGCCGCCAGGTCATCCCCTTATATACACGGGTGACGCCCGTCGGTCTGCAAAGTCCCAACCTCCGGCTAATAATCGTGCCCGGGTCGGTCTCTCTATTCCTAACTTACAATACAAGTTTACATCAGGCCGGTTTACACCATAAACCTTTAAACCGATTATGGGCCTTAGGCCCTTCTATCTCTCCTTGGGTTTTAACATCTCGGGTCTGCTGACGGAGGTAACCCGGGCCCTGCAGGCCGGTTTACCCCGGATAGCAACATCCCCAACA

TTAGGCCCCAGATTGATTTGAACAGGTTCATGTCAATCCTTAATAAGAATTTCTGTCTTCAAATGTCTTCCAGTGACTTGTTAAACCGTCGTTTCTCCGTCTCCTCTTGCTGTGGTAAACCGATATGACGTCAGCGCAAGCTACCGTCTCCTTTCGCTAACCCGCAAGAATCAGGGCGACGGCTGGGCTTCCGAAATCTCCGGCTCCTGGATTCGCGCGCCTGACACGCGCGCCGCGCCTTATAAATAAGACTTGAAGGCCATCTTCCTTTCCTCCCCTTCTTCGTCTTCCTCGCGACCCAGACCTCAAAGCTCCGCCGCCGCCGTCAGCTGCTGCACCAACTCCGGCCGCTGCATCAACCTGATCGGTTCAGAGCACCGCGGCGACTTTCCGCATCTGTTTGGACCCCGGTAAGGCCCCTGTTCTTGTTGCTTTAGATCTGTTCTAGGGTTCATCGTTCTGCTACTGTTTCTTGCTCATCCCCATAGCAGCCTTTGCGAACAGATGATGCTACCTGTATCTGGATTTGTAGTTCCTCTTAGCAGTCACCATAAATCTGCACTCATTTCCTTAGATCTAGCTCTTGTTCCTGCTTGTCGAACTTATGTTGTTCTGCTGTGAGCCTGACTTCATATTACCTGTTTTTTATTTCTTTTTCGCCTTAGCTGTAGATCCATTGCTTTGAAAGAATCTGTACGGGATCTGTTTCTGCTTTCATACTCACAACTGTTTAGATCTGGTCGGTTTAACTCTTAGCAAAGCGAACAAACCGATCTACACATCCATTAGTCCCCTTGATGAACCGCCGGTTGATATTATTGTAGATAACCTGACTCTATATAAGAGCATCTTCGGTTTACAATCATACTTGCTCTCAAACCGGAATACCTTTGCCTTGTAGATTTCCTCATGGCAAAACAGGTGTATGAATGCAACTGGGTTCCCTCTTGCATCACTGAGACCCAACTAAACAACTTAGTTCTGACTGGTGCTCTGAGTAGCAAAAGTACCATCCATTGGAGGGTTCCAGGAGACGAATGTCCTCCTACCCCGCAGGAAGGAGAAGTGGTGGTGTTTGCGGATCACCTGGCCCGGGGGTTTAATCCACCCGGTTCAAAATTTTATCGGGACGTCTTGGCTAATTTTAGACTGCGCCCACAAGATGTTGGTCCAAATTCAGTCACGAATATCTGCCATTTCCAAGTACTTTGCGAGGCGTACTTTCAAGAGGAACCCACTGTAGAGCTGTTCCGGGACTTTTTCCATCTGAACCGACGTACTGAATTCACCGATGGCCCTAATATGGGACTGGGTGGTATGGCGATTCAGAAGAGGAAAGAGGTGACTTATCCTCACGCCAAGTTGCATAGTCATCCGCAAGAATGGAATTGCACTTGGTTCTATTGCAAGGATACTTCCCCTGCTGAAGAAAATCCACTGCCCGGTTTCCGTCCAGAGAGACTTAGCAATACTCACCCCTTCCCCCCACGGCTAACTGCCAAAGAGAGGAGTAAGTACGCCCCTCAGCTGTCCAAGCTCAGAGCCTTCATGGCTAACGGTTTAACGGGGGTAGATCTTGCTCGTTGCTGGATTTCGTGGAGCATCCTGCCCCTTAGCATCCGCTCCGGTTTAATGTGCCAATACACGGGTCGGGTTGATGATCCCTTGAGGCACTGCAACATTCAGCTGACGGAGGACGAAGTCGCTGAAGCTGTGAAAAAGATGCTGAATGAACCGGAACATGTTTGTGCCCAAACCGGCCTGCTTCCATTCTGTACCACCAACAAGCCTCCTGCTGTAAGTATCTCCCCTCTGTTTGTTATAGAACTTATGATCCCCTTATACACATATTGCTAACTCCAGTCCTTCCTGAGACAGGGAGATGATCCATTTTGGAGCAAGAAGTTACAAGAGCCAACCGAAAAGGTAACCCGACCCAAGACGAGGGTGAAGAAGCCTGCTCAGAGGAAAAGGAACACCGCTTCCTCTACTCCGGATTTAGAGGAGGATGATGTGGGTAATCCGGACCTCGGGGTAGACCTCGATTCAGTTGGTTTGCTTTTTATACGTCTTATTGATGATGATATTTGTCAGGACGACGCTGAAGTGAGCCATGCCGAAGGCCCAGAGGTAACTATCCTTTCTTCTGATGCCGTTCCTTTGCCTTTGAAAAAACCTCGCCAAGCAAACCGGAGAATTAAATTTTCTCACCCTCTTGCTTACTTGGATCCCAAATTTCTTGTGAAGACCCAGCAACATGAAGCTCGACGCACCACCCGACATAGCGGCCAGGTAGTCACCTCCGCCGGTTTACCGAACAGCCCGGTTCGGAAACGCCGCTCAGAGGTCTCCGATTCACGCGCTAGCTTTCCTCCTAAAGCATGTTTAAATCGTCAGCCTCTAAATCCATCTGACTCCGATTATCAGGTCACTTCCCATTCCTCTTCCGGTGAATCTTCAGCCACTCAACTGCCACCGTTGAAGACAGTTATTGGGTAAGTTGGACCGATCACAGCATATCTCTGTATGGGTTTCTTAATCCATCTTATACCGTTTTTTCAATCTTTGGGCTTTTTCCAGGGCCAAACCTAGACCGAGCAAGAAGGCTCGTCTGGATAACCCGGCTGGAGGAACTGCCGCCCCTGAACCGGAGAAAACTCCTGGTGCTGACGCTCTGGGTCGTAAAGATACTCAGAGTTATCTATCTCCGCAAGATGACACCTGCGCTGAAGAAAGGATTATCGAACCTGCCAGCCATACTGATCCGCCTGCCAGTCCTGTTCGGGTTGAGGAATCCATCCCCCCTTCTGGAACTGCTGATAAACCGACAGGGCCACTGCAGAGCAGCGATTCCAAGGACGATGATGTTGTGATTACCGGCATCGGTCACTCTGAACCGAGGAACACCGCTACCTTAGCCAAGCATACTGCCAAGGACGAAATCTCGGCCATGAAGGGCAAATGGGACCTTGACTCTTCTTCGTACGCCGCTTTAAGCGCCCCGGATCTTTATTCCGGGTACCTGAACCGACTGTACACCAGTCGGGATTATGAAGCTGGCATGATCAAGATGATGAAGGAGAAACTTGAGGTAACTCCCTTGTGCTTCATTCCCTTGCAACTTATATTATCAATGTTTCCAAACCGAAATGGTAAACCGGATCAGGCTGTCTTGAAAATTAAAGCCCGGTTTAGAAAACCCAAGGCTTCAACATAGGTCATAACCAGAAAACATCATTAGATAACTATTAGCCCCCAAGTGCCAAGTTTAAATACTTGTATTAATCTTGGGACTTTTCGCAATGGGAAACTTGTGTTAAAATCAAATATGCATTAGCCCCCAAGTATCGAGCACATAACCTGCTTGTGTGGTTGATACTTCCAAACCGTGCAATATTTTCTGAACACTGTTGACGACCTGCTGCAGGGGGACCTGAGGATCAAAGCGAACCAAGTCTCGGACCTTCAGGAAATCTTAAGAACCCAACAGGCTAAAACTGAAAAAGCGAAGGATGAATTGGCCAACGCCTTAACCGTTATGGAACAGCTGAAAGAGAGCCACAAAAAGGAACAAGCTGACTGGGCTACTGAGAGAGCTCTTCTGACGAAGAGGGCTGAAAATGCCGAAGCTGCTCTTAAACCGGTGGTTGATGAATTATCCACCGTGAAGCGACAGATACACTCCATGACCTCTGCTGTCTTTGGTAAGCTTTTCGATGCCCTTCTTCCAATTCGTACCTTGTCTGTTACCTCCTATGTCCGGTTTGTAATCCATCTTGAAACACTGCAGGCTCACGTATTGGACACTTGGGTTCTGATGTGCGGATGAAACTCAAGGCTGCCTATACTCTGATTGAACAGTTATACACTGGAGCACAAAGAGCTATCACCACTGCATCGCACAACAACCCTGCGCCGGCTCTGATTCAAGACACATTAAAGAGGCTGTCCATGCTGCCGGCCCGGATTGAAGAGTTGAAAAGATCAGCTGCTCGAACTGGAGCTATCACTGCCTTGATCCGGGCCAAAGTGTGGGTACCAGATTTCGATCCTGTCGAAGCGGCCCAAGGATACCCCAGCTTGAAGGAAGACGGTTCAGACTTTGGTGAAGATGATTTGAGGGCGATAAACCGGGCGGTGCGCCCCCTAGCTTGTCAATTGGCTGAGGAGGCAGATCTGTCACGTTACCAAGCCCAATACGACAACCAGAACAAACGAGTATCTGCCCCGGTCCCTGAAGCGGGAAACCTTGTTCCTCCAATCCGTAAGCATACTTACGCCCCAGACATTGAACCGTCTTCGCTGATTCACAAAGAAGCCGTCTTTCAAGCGCTAATGGGAATCGACTGGACCACTGCTGATTTCCAGCCAATGGGTAACCAAGATGGAGCTGAAGCGGCGCGAGACGATCCCCAGCCTTCGAACCAAGGCGGCGAACAGGCGTAATCAACGCTCCGGTTCACCCTACTTGGTGACGTCTTAATCCGTCAACGAACAATATATTCAGGCACTACGTTGCCTTGTAATAAGATAGTTTAATACCTCTGCCTTTGAATGTGCCTTCGCGCACGGGTACTGATGCTCTTTCCAATGACGTTTCCAACTTATGTTTCCTGTCTTTACTGAGTACACAATGTGATTTTTTCGACCACCTGAACCGTGCTGCGCCTCTGCCAAACCGGGCAATTTGCCTCCGGGTTAAAACGGACCAGGCTATGCCAGCTGAATCGGTTTAAGGATACTTGACGTTCCAAAGAACCAAAAAACACCACAATAAAGGCAGAAAACAAAACCAAATATGAAAAGGCAACGCGATAGTAAAATGCATGCGAATAGTGGTTACTATGAACCGTTCAGAAGGCGTGACCAACGTCAAGACAAAACCGAGCCCCCAAGAGGTGTAGCTACGGTTTAAGTCGACTAGGTTCCTAACTGAATCGTGGCAAATATGCCGGCCAAATGGCATTGCATTGGTTCGTCAACCAAGCCCCCAAGTGACTCAATGGCTAAAAAGCCGACCAGAAGGCATTACCATCATCAAGACATGACCGAGTTCCCATAAGGTGTAATCTACGGTTCAAGCCGATTAGGTCCCCAACTGATTCAGTGGCAAATATGCCAGCCAGAAGGCATTACCGTCGTCAAGACCTGACCAAGCCCCCCGGAGGTGTAGCTACGGTTCAGGCCGACTAGGTCCCCAACTGACTTTATAGGGATAGCTATGTTCTCTTTGGTTCGAATACGACCTATGTTTGAACAGCTGTGTTCTCTTTGGTCCGAATACGACCTATGTTTGAACAGAAATCCCCATGTTTGAATAGCTATGTTCTCTTTGGTTCGAATACGACCTATGTTTGAACAGGAAGCCTCATGCTGAATGGCTATGTTCTCTTTGGCTCGAATACGGCCTATGTTTGAACAGTAAGCCTTATGCTGAATGGCTATGTTCTCTTTGGCTCGAATACGGCCTATGTTTGAACAGTAAGCCTTATGCTTGAACCGGGATATTGAACCAGATATTAAGAGCTTCACAACCCTGTAGGAGACATCTTCCCTTGAACCAGCTTTTAAACCGGAATTTGAACTTCTGTGAAGCGACCTCCAGGGGTAAGTACACATGATGTGAAGAACAGAAGAGACCCCGCTTTACTCGAGGCTCCGGTTTATTGTATTCAATCATAATATATACATGGTCATGATATGTACATAAACAGAGCCCAGGGCTCATGTATAATAAGGTCGAAGATGAGCTATGTTCCACGGCCTGCTGGTCTCCTCCTCCGCTGTGCGTGAATCCTTGCGCTCTCGAATATCAATCAGGTAATATGACCCATTATTCAGATTCTTGCTGACCACAAAGGGTCCTTCCCAAGGCGGGGATAACTTATGCTGATCAGACTGATCCTGGATGAGCCGAAGCACCAAATCCCCCTCCTGAAAGGTTCTGGTCCTAACCCGACGGCTGTGGTAGCGGCGAAGATCTTGCTGGTAAATCGCTGAACGGGCAGCTGCCAAGTCGCGCTCCTCATCCAACAAGTCAAGAGCGTCCTGCCGTGCCTGCTCATTGTCCGCTTCAACATATGCCGCCACACGAGGCGAGTCATGTCGTATATCACTTGGGAGAACCGCCTCTGCTCCATAGACCATAAAAAACGGCGTAAACCCCGTGGATCTGTTAGGCGTGGTGTTGATGCTCCATAACACAGACGGTAGTTCCTCCACCCAACAACCCGGCGTCCTTTGCAAAGGGACCAGAAGCCGGGGTTTGATACCTCTCAAGATCTCTTGGTTCGCTCGTTCCGCCTGACCATTAGACTGTGGGTGCGCTACCGAAGAAACATCAAGCCGTATATGCTCCCGTGCACAGAACTCTTTCATGGCCCCTTTGGATAGATTGGTACCATTATCTGTGATGATGCTGTGTGGAAAACCAAACCGGAAGATCACCTTCTTTATAAATTGAACCGCCGTGGCAGCATCACACTTATTGACAGGTTCTGCCTCCACCCACTTGGTAAATTTGTCAACCGCCACCAGTAGGTGGGTCTTCTTATCTTTGGAACGCTTAAAAGGTCCAACCATATCAAGCCCCCAAGTCGCAAACGGCCAAGTGATTGGAATCATCCTCAATTCTTGAGCCGGTATGTGTGCTCGTCTGGAAAATTTCTGGCACCCATCACATAACCTGCTCAGGTCTTCTGCATCAGCATGAGCAGTTAACCAGTAGAAACCGTGGCGGAACGCTTTAGCCACCAATGATTTTGAACCGGCGTGGTGGCCGCAATCCCCTTCATGTATTTCTCGTAATATCTCGCACCCCTCCTTAGGGGATATGCACCGTTGCAGCGCTCCTGACACGCTGCGATGATGCAACTCTCCTTGGAAAATGGTCATAGACTTGGACCGTCGTACTATCTCTCGAGCCAAACTTTCATCTTCTGGCAACTCACCCCGGTTCATATATGCCAGATAAGGGAGCGTCCAATCCGGGGTGGCGTGAAGAGCCGCCACTAACTGAGCCTCCGGATCAGGAACAGCCAACTCCAGCTCACCGGGCAGCTGAACCGATGAGTGGTACAATACATCCAAGAAAACATTGGGTGGGACCGGTCTACGCTGAGAGCCCAAGCGACTTAAAGCGTCAGCTGCCTCATTCTTTCTGCGGTCCACATGATCCACTTGATAGCCTTTGAAATGCCCAGCCACCATATCTACTTCACGTCGGTATGCAGCCATAAGCGGATCCTTAGAATCCCAGGTGCCAGATACCTGCTGAGCCACCAGATCCGAATCACCAAAGCACCTAACTCTGCTCAGATTCATCTCTTTAGCCACCCGAAGACCATGAAGCAAAGCTTCATACTCCGCCGCATTATTTGTACAGGGGAACATTAAACGGAGGACATAACAAAACTTATCTCCTCGTGGGGAAGTTAAAACGACTCCAGCCCCCGAGCCTTCCAATTGTCTTGATCCGTCAAAATGAACGGTCCAATAGGTGTTATCTGGCTTCTCCTCAGGCGCTTGTAACTCTGTCCAATCGTTTATGAAATCGACAAGTGCCTGTGACTTTATGGCCGTTCGGGGTACGTACCTTAAATCGTGCGGCCCGAGCTCGATAGCCCACTTGGCAATCCGACCGGTCGCCTCCCGGTTCTGTATAATGTCACCCAAAGGAGCAGAACTGACCACCGTGATTGGGTGTCCTTGGAAATATTGCCTCAGCTTCCGGCTTGCCATAAAAACGCCATATACCAGCTTCTGCCAATGCGGATACCTTTGTTTGGACTCGATAAGTACTTCACTGATATAGTAAACCGGTCGCTGAACCGGATGTTCCTTTCCAGCCTCCTTTCGTTCTACCACCATCGCCACACTGACTGCTCTGGCATTCGCTGCAACATATAACAGGAGTGGCTCTTTATCTACAGGGGCTGCCAACACTGGCGGATTGACTAGTTGTCGCTTCGAGTCCTCAAACGCTTTATCAGCCTCCGGACTCCAGACAAACTGATCCGTCTTCTTGAGCATTTGATACAAAGGGATCGCCTTTTCACCAAGGCGACTGATAAACTGGCTTAACGCTGCAATCCGCCCGGCCATGCGCTGAACATCATTGATGCACTTCGGTTTGGCCAGGGAGGTGATGGCTGTGATCTTTTCTGGATTGGCCTCAATTCCTCTATGGGACACTAAAAATCCCAACAGCTTACCTGCAGGTACACCAAAAACACATTTAGCCGGGTTGAGCATCATTTGGTACACTCTCAAGTTATCGAAAGTCTCCTTCAAATCGTCAACCAGGGTCTCCTTCTTTCGTGACTTGACCACAATATCATCCACATAAGCATGCACATTTCGACCAATCTGCTCGTGGAGGCAATTTTGCACACATCGTTGATAAGTAGCCTGCGCACTCTTAAGCCCAAAGGGCATAGACACATAGCAGAAGGCTCCAAAGGGAGTAATAAACGCTGTCTTCTCCTGGTCCTTAACTGCCATCTTGATCTGATGATAACCCGAATAAGCATCCAAAAAACTTAAACGCTCGCAACCTGCCGTAGCATCAATGATCTGATCAATCCGGGGGAGAGCAAAAGGATCTGCTGGGCAAGCTTTATTCAAGTCTGTGTAGTCCACACACATGCGCCAGGTGCCGTTTTTCTTAAGAACTAGCACCGGATTAGCGAGCCATTCGGGATGAAAAACCTCAATGATAAACCCAGCTGCCAGGAGCCTGGCCACTTCCTCACCAATCGCTTTACGTCTTTCTTCGTTAAACCGCCATAAGAACTGCTTTACCGGCTTGTACTTAGGATCTACATTAAGGGTGTGCTCAGCGAGTTGCCTCGGTACACCAGGCATGTCAGATGGCTTCGATGCAAAGATGTCCCGATTCTCACGGATGAACTCGATGAGCGCGCTTTCCTATTTTGGATCCAAGTTTGCACTGATGCTGAACTGCTTAGATGAATCGCCAGGTACGAAGTCAACAAGCTTAGTTTCGGCCGCTGACTTGAACTTCAAGGCCGGATCGTGGTCCGTAGTTGGCTTTTTTAATGAAGTCATATCCTCCGGATCAACACTGTCTTTGTACTGCTTTAGCTCCTCCGTAGCACAAACCGATTCAGCATAAGTCGCATCTCCTTCCTCGCATTCCAAAGCGATTCGGCGGCTCCCATGAACCGTGATAGTGCCCTTGTAACCTGGCATCTTAAGCTGCAGATATACGTAGCAGGGCCGTGCCATAAATTTTGCATAAGCTGGCCGCCCGAACAAAGCGTGGTACGGACTTTGGATTTTAACCACCTCGAACGTCAGCGTCTCTGATCTGGAATCGTGACTATCGCCAAATACCACTTCAAGGGCTATCTTACCAACCGGATACGCAGACTTGCCAGGCACCACCCCGTGGAACACTGTATTGGTCGGTTTGAGATCCTTATCTACCAGTCCCATACGACGGAAGGTCTCATAATACAAGATGTTAATGCTACTCCCTCCGTCCATGAGCACCTTGGTGAGCTTATAACCTCCCACCTGAGGCGCTACCACCAGTGCTAACTGACCCGGATTATACACCCTGGGAGGATGATCTTCTCAGCTCCATATGATAGGCTGTTCCGACCAACGCAGATAGTGAGGCGTGGCCGGTTCGACAGCACTGACTGCCCGCCTCTGAAGCTTCCTATCTCGTTTATCCAAGCTCGTAGTAAAGACATGATACTGTCCACCACTCAACTGTTTCGGGTTGCTCTGATAACCACTTTGATTACTTTGATTATTCTGATTATTTTGGCTGACCTGTCCGCCCGGGTTACCCTGGAACCCTGGACCGGAATTCCCGCCACCGTACCCTGATCCGGAACCGGAACCGCCACCGGAGCTGTGATCATATTGGAAAGTGTTTGAGCTTTTGAACTCCTTCATGATATAACAATCCTTCCAAAGATGGGCTGCTGGTTCCTCCTTTGTTCCGTGCTTCGGACAGGGCTGGTTTAAGAAAAAGTTCAAACGCTCCGGATTAGGACCGGGATGTGCGCGGAAACTTGGCTGTTTCCCCTTACGCCGCTGGCCTTTATCCTGCGCACTTGTGCTGGCCACCAAGTCCATGCTCCCGTCCGGTTTACGCTTTCCTCCGCCTCCATTACCTGTCGGCCGATGCTGCTGGCCTTTGGTGTTGCTATTCTTCCTTCCCTTCCCTGCTTTGTCATCACCAGAGTCAGGATCCTTGGTACTGTCAGAATCCGCATACTTCACTAAAGCGGTCATGAGCGTTCCTATATCTGTACAATCACGCTTCATCCGGCCCAGCTTTAGCTTCAAAGGGCCAAACCGACAGTTGCTCTCCAACAGCACCACGGCGGTGTCTGCGTTGATGCGATCTGACGAGTGCAAAACTTCTGATACCCGCCGTACCCAATGGGTAGTTGATTCCCCTTCTTGCTGAACGCAGGCTGCTAGGTCAACTATTGACATTGGCTGTTTGCACGTGTCCTTGAAATTCTTGATAAACCGGGCGCGCAATTGGGCCCATGAACTAATTGAATTAGGCGGTAAGCTTTTTAGCCAAGTCCGGGCCGTTCCTTCTAACATCATAGTGAAATACTTAGCACACGCCGTGTCATCCACATCCAGCATCTCCATAGCCATTTCATAGCTCTCCACCCATGTCTCCGGAGGCTGATCTGCCGTGTAGTTTGGTACCTTGCGGGGGCCTTTGAAGTCTTTGGGCAGGCGTACGTTGCGTAAAGCGGAAACAAGGCACGGTACTCCCAAAGAACTGGAAACCACCCCCGGTTCAACCGTAGCTGCGGGGAAGATAGGTGTAAGCTGACGAGCCTGATGCTGCGCTGCTAATCCGGCCTCCCGTGCACGGGTGCGATCCACATTCTCGTGAGTATTGTCGACGCTCGCCGGATTGTTGGCACGGAGCGCTTCACGCCTGGCGTTACTTGAAACGGCCGGCTCCTCCACGCGCCTACTATAACTCCTGCTGGGGCGAGGGGTGGAGTGAATCCGATCACGGCTTTGCGAATACGCTTCTTGCTGGGCCAACGCGGTCCTAAGGAGCTCCTTGACCCGGCGTGTCTCTTCATCCTGCGGTGAATCTCCTTCGATCGGAATGGCTTCCAACCGAGCCGCCGCCGCGACGAGGTTATCCATCGGGTTGGAATAATGACCTGACGGCGTTGGCATGGCCGGTGGCGTATCAGTGTTATGACGAGGCAGGTCTATAATCCGGTGCTGAACCGGGGCGCCGGCTGTCGCCTCCGCACGGTTTACCCCCGGGGTGTTGAAGATATTTCGAGCGTCGAGGGCGGCGGGCAACCGAGACCGGGATTTTCTCTTCAGGACCTCCTGAGATGCCTTCTGGTCCATCATGAGCCTGTAAGCCTGTGCCTCTAAAGCGGCGCGCTCCGCCGTCATCCTGATCTCTTCTGCTGCCAGCACCGCTTTAGCCCGAGTGATCTGCTCTCTCACTTTGGCAATCTCAGCATTGTGAACCTCCTGATCCACCGGGTTGGCCTCCGCCATGAGCGCTGCTAGTGCGTCAAACAGGTCTGACAAAACTTGGGCTGGTGAACCGGTAGGGCCTCGTGCCCGCGCAGCCGTTGCTGCTGCCGATCCGGAGATCGCTGCTGCTGCAGTTGATGATCGTGGCGTCGCCTGTGTCCCAGCCATGAAGATCCCAACCCGATAGGGCAGATCAGAGGGGTCCGGAATACTGTTGCCATCGGAGCCGCCCCCAACCCGATCATCTTGTAGCTGGTAAAGAGATTCGGTCTCCCCGGTCGACGTTTCATCGCCAGAGTAGATGGCGGTTTCGTCACCAGATTTGGATCCTTCCTCGTAGCTTCCCCCGTGGATGACTCCCACGAAGGCGTGCTTCCTGGTCGGTTTAGCCAGGGTCGATCTCGTGCACTGAGCTGTCTCAATGAGGTCGGCGCAGTCGCCTAACTCGGGGCCCGGTTCTTCGATCTTGCCGATGAAAACATGGATGCTGCCAAAGGGGACCCGGTACTCGTACTCGATCGAACCGGCGTCGGGGCCCCAGACTGCGCTGTCGATGTAGAGTTTTCCGCGGCGGCTCTTGGTCATCCGTCCCACAGCGTAACCCTCGAGTCCTTCAAAGCGGCCCTCCAAGAACCTGAAACCATCTCGCGATAGCCCCACGGTGGGCGCCAAC

TGTCGTCGGTTTGTCACGGCAGATGTCCTAGTAAGAGGACTTAGTCGTGGAGCCATCGCAACGGGTTAGCTTGAAGGGGTTAAAGTGGACACAAGGACACGGTTTTATACTAGTTCGGCCCCTTCGATGAAGGTAAAAGCCTACGTCTAGTTGTGATGGGATTGATGGGTTTCGAGGGCTCAGGGAGCGATAAGCTTCGCCTAAACCTCGAGTCCTCTTTCTCTGTCCTCAAACCGCCGCCAGGTCATCCCCTTATATACACGGGTGACGCCCGTCGGTCTGCAAAGTCCCAACCTCCGGCTAATAATCGTGCCCGGGTCGGTCTCTCTATTCCTAACTTACAATACAAGTTTACATCAGGCCGGTTTACACCATAAACCTTTAAACCGATTATGGGCCTTAGGCCCTTCTATCTCTCCTTGGGTTTTAACATCTCGGGTCTGCTGACGGAGGTAACCCGGGCCCTGCAGGCCGGTTTACCCCGGATAGCAACATCCCCAACA

AAAGGTCAGTACAGGCCTACATCCACATGATTGTGCAGTGTGCTTTGAGATGCTGTGCTACTTGTATTGGTACGGTTTTAAATAAATGATGAATTGAAGCTATTGTATTGCTGTCTTTTCCCAT

>Aegilops umbellulata 1-249;

AGGTAATCACACGACCTCCTCTGGAATCTTTGTATTGCTGGAACCACTCAGCAGCTTGATCTTTGAGTTAAAAAGAGGCAAACTTGACAAAGTCCTCAGGTCTGACATTGCTGCACTCGAAATG

TGTCGTGGATTTGTCACGGCAGATGTCCTAGGAAGAGGACTTAGTCGTGGAGCCATCGCAACGGGTTAGCTTGAAGGGGTTAAAGTGGACGCGAGGACACGGTTTTTATACTAGTTCGGCCCCTTCGATGAAGGTAAAAGCCTACGTCTAGTTGTGATGGGATTGATGGGTTTCGAGGGCTTAAGGAGCGATATGCTTCGCCTAAACCTCGAATTCTTCTTCTCTGTCCCCAGACCGCCGCCGGGTCATCCCCTTATATATACGGGTGACGCCCGTCGGTCTGCAAAGTCCCAACCTCCGGCTTATAAACGTGCCCGGGTCGGTCTCTCTATTCCTAACTTACAATACAAGTTTACATCAGGCCGGTTTATACCATAAACCTTTAAACCGATTATGGGCCTTAGGCCCTTCTATCTCTCCTTGGGCTTTAACATCTCGGGTCTGTGGACGGAGGTAACCCGGGCCCTGCAGGCCGGTTTACCCCGGATAGCAACATCCCCAACA

TTAGGCCCCAGATTGATTTGAACAGGTTCATGTCAATCCTTAATAAAAATTTCCGTCTTCAAATGTCCTCCAGTGACTTGTTAAACCGTCGTTTCTCCGTCTCCTCTGCTGCGGTAAACCGATGTGACGTCAACGCAGGCTACCGTCTCCTTTCGCTGACCCGCAAGAATCAAGGCGACGGCTGGGCTTCCGAAATCTCCGGCCCCTGGATTCGCGCGCCTGATACACGCGCCGCGCCTTATAAATAGGACTTGAAGGCCATCTTCCTTTCCTCCCCTTCTTCGTCTTCCTCGCGACCCAGACCTCAGAGCTCCGCCGCCGCCGTCAACTGCTGCATCAACTCCAACCGCTGCATCAACCTGATCGGTTCAGAGCACCGCGGCGACCTTCCGCATCTGTTTGGACCCCGGTAAGATCCCTGTTCTTGTTGCTTTAGATCTGTTCTAGGGTTCATCGTCCTGCTACTGTTTCTCGCTCATCCCCGTAGCAGCCTTTACGAACAGATGACGATACCTGCACCTGGATTTGTAGTTTCTTTTAGCAGTCGCCATAAATCTGCATTCATTTCCTTAGATCTCACTCTTGTTCCTGCTTGTCGAACTTATGTTGCTCTGCTGTAAGCCCGACTCCATATTACCTGTTTTTATTTGTTTTTCACCCTCGCTGTAGATCCATTGCTTTGAACGAGTTCGTACGGGATCTGTTTCTGCTTTCATACTCACAACTGCTTAGATCTGGTCGGTTTAATTCTTAGCAAAGCGAATAAACCGATCTACACATCCATTAGTCCCCTTGATGAACCGCCGGTGATATTATTGTAGATAACCTGACTCTATATAAGAACATCTTCGGTTTACAATCATACTTGCTCTCAAACCGGAATACTTTTGCCTTGTAGATTTCCTCATGGCAAAACAGGTGTACGAATGCAACTGGGTTCCCTCTTGCATCACTGAGACCCATCTAAACAACTTAGTTCTGACTGGTGCTTTGAGTAGTAAAAGTATCATCCATTGGAGGGTTCCAGGAGACGAATGTCCTCCTACCCCGCAAGAAGGAGAAGTGGTGGTGTTTGCGGATCACCTGGCCCGGGGGTTTAATCCACCCGGTTCAAAATTTTATCGGGATGTCTTGGCTAATTTTAGGCTGCGCCCACAAGATGTTGGTCCAAATTCAGTCACGAATATCTGCCATTTTCAAGTACTTTGCGAGGCGTACTTCCAAGAAGAACCTACTGCAGAGCTGTTCCGGGACTTTTTCCATTTGAACCGACGTACTGAATTTACCGACGGCCCTAACATGGAGCTGGGTGGTATGGCGATTCAGAAAAGGAAAGAGGTGACTTATCCTCACGCCAAGTTGCATAGTCATCCGCAAGAATGGAATTGTACTTGGTTCTACTGCAAGGACACTTCCCCTGCCGATGAAAATCCACTGCCCGGTTTCCGTCCAGAGAGACTTAGCAACACTCACCCCTTCCCCCCACGGCTAACTGCCAAAGAGAGGAGTAAGTATGCCCCTCAGCTGTCCAAGCTCAGAGCCTTCATGGCTAATGGTTTAACGGGGGTTGATCTTGCTCGTTGCTGGATTTCCTGGAGCATCCTGCCCCTTAGCATCCGGTCCGGTTTGATGTGCCAATACACGGGTCGGGTTGATGACCCTCTGAGGCACTGCAACATTCAGCTGACGGAGGACGAAGTCGCTGAAGCTGTGAAGAAGATGCTGAACGAACCGGAACCTGTTTGCGCTCAAACCGGCCTGCTTCCATTTTGTGCCGCAAACAAACCACCTGCTGTAAGTAATTCCTCTGTTTATAGAATCTTTGATCTCCTTATATATATGTTGTTAACTCCAGTCCTTCCTGAGACAGGGAGATGATCCATTTTGGAGCAAGAAGTTACAAGAGTCGACCGAAAAGGTAACCCGACCCAAGACGAGGGTGAAGAAGCCTGCTCAGAGGAAAAGGAACACCGCTTCCAATACTCTGGATTTAGAGGAGGATGATGTGGGTAATCCGGACCTCGGGGTAGACCTCGATTCAGTTGGTTTGCTTTTTGTACGTCTTATTGATGATGATATTTGTCAGGATGATGCTGAAGTGAGCCATGCCGAAGGCCCAGAGGTAACTATTCTTTCTTCTGATGCCGTTCCTTTGCCTTTGAAAAAACCTCGCCAGGCAAATCGGAAAATTAAATTTTCTCACCCTCTTGCTTACTTGGATCCCAAATTTCTTGTGAAGACCCAGCAACATGAAGCTCGACGCACCACCCGTCATAGCGGCCAGGTAGTCACCTCCGCCGGTTTACCGAACAACCCGGTTCGGAAACGCCGTTCAGAGGTCTCCGATTTATGCGCTAGCTTTCCTCCTAAAGCCGGTTTAACTCGCCAGCCTCTAAATCCATCTGACTCCGATTATCAGGTCACTTCCCATTCATCTTCTGGTGAATCCTCAGCTACCCAACTGCCACCGTTGAAGACAGTTATTGGGTAAGTTGGACCGATCACAACATATCTCTGTATGGGTCTCTTAATCCATCTTACACTGTTTTACCAATCTTTGGGTTTTTTCAGGGCTAAACCTAGACCGAGCAAGAAGGCTCGTCTGGATAACCCGGCTGAAGGAACTGCCGCTCCTGAACTGGAGAAAACTCCTGGTGCTGACGCTCTGGATCGTGAAGATACTCAGAACTATCCATCTCCGCAAGATGACATCTGCGCTGAAGAAAGGACTACCGAACCTGCTAGCCGTACTGATCCGTCTGCCAGTCCTGTCCGGGTTGAGGAATCCATCCCCCCTTCTGCAACTGCTGATAAACCGACAGGGCCACTGCAGAGCAGCGACTCCAAGGATGATGATGTCGTGATTACCGGTATTGGCCGCTCTGAACCGGGGAACACTGCTACTTTGGCCAAGCATACTGCCAAGGACGAAGTCTCGGCCATAAAGGGCAAATGGGACCTTGACTTATCTGCGTACGCCTCTTTAGGCGCCCCGGATCTTTATTCTGGGTATCTGAACCGGCTGTACACCAGTCGGGATTATGAGGCTGCTATGATCAAGATGATGAGGGAGAAACTTGAGGTAACTCCCTTGTGCTTCATTCTATTGCAACTTTAGCTTATCAAATACTTCCAAACCGAGATGATAGTCCGGACCCGGCTGCTTTTGACAATTAAGGTGTTAAAATCAAATATGCATTAGCCCCCAAGTATCGAGCACATAACTTGCTTGTGTGGTTGATACTTCCAAACCGTGCAATATTTTATGAACACTGTTGATGACCTGCTGCAGGGGGATTTGAAGACCAAAGAGAACCAAGTCTCGGACCTTCAGGAAGCTTTAAAAGCCCAGCAGATTGAAACTGAAAAAGCAAAGGAGGAATTGACCCAAGCCTTAACTGTTATGGAGCAACTGAAAGCAAGCCGCAAAAAGGAGCAAGCCGATTGGGCTACTGAAAAAGCTCTTTTGACGAAGAGGGCTGAAAATGCCGAAGCTGCTCTTAAACCGGTGGTTGATGAGTTATCCACCGTGAAGCGACAAATACACTCCATGACCTCTGCCGTTTTTGGTAAACTTTTTCGATGCCCTTCTTCCAATTTGTACCTTGTTTGCCACACCCTATGTCCGGTTTGTAATCCACCTTGAAACACTGCAGGCTCACGTATTGGACACTTGGGTTCTGACGTGCGGATGAAACTCAAAGCTGCTTATACTCTGATTGAACAGTTATACACCGGAGCGCAAAGAGCTATCACCACTGCATCGCATAACAACCCTGCGCCGTCTCTGATTCAAGACACATTAAAGAGGTTGTCCATGCTTCCAGCCCGGATTGAAGAATTGAAAAGATCAGCTGCTCGAACTGGAGCCATTACTGCCTTGATCCGGGCCAAGGCATGGGTACCAGATTTCGATCCTGTCGAAGCAGCCCAAGGATACCCCAGCTTGAAGGAGGACGGTTCAGACTTTGGTGAAGACGATTTGAGGGCGATAAACCGGGCGGTGCGCCCCCTAGCTTGTCAATTGGCTGAGGAAGCAGATCTGTCACGTTACCAAGCCCAATACGACAACCAGAACAAACGAGTATCTGCCCCGGTCCCTGAAGCGGGAAACCTTGTTCCTCCAATCCGTAAGCACACTTACGCCCCAGACATTGAACCGTCTTCGCTGATTCATGAAGAAGCCGTCTTTCAAGCGCTAATGGGAATCGACTGGACCACCGCTGATTTCCAGCCAATGGGTAACCAAGATGGAGCTGAAGCGGCGCAAGATGATCCCCAGCCTTCGAACCGTGGCGGCGAACAGGCGTAATCAGCGCTCCGGCCCACCTTTGGCGAAACTATAATCCGTCAGCGAACAATATATTCAGGCACTACGTTGCCTTGTAATAAGATAGTTTAATACTTCTGCATTTGAATGTGCCTTCGCGCACGGGTACTGATACTCTTTCCAACGATGTTTCCATCTTATGTTTTCTGCCTTTACTGAGTACACATTGCTATTTTTCGACTACCTGAACCGTGCTGCGCCTTTGCCAAACCGGGCAATTTGCCTCCGGGTTAAAAAACGGACCAGGCTACGCCAGCTGGATCGGTTTATGGATGCTTGATGTTCCAAAGAACTAAAAAAGACACCACAATCAAGGCAGAAAAAACCAAACCAAATATGAAAGGCAAAGCGATAATAAACGCATGCGAATAGTGGTTATTATGAACCGTTCAGTAGGCGTGACCATCGTCAAGACAAAACCGAGCCCCCAAGAGGTGTAGCTACGGTTTAAGCCGACTAGGTTCCTAACTGAATCGTGGCAAATATGCCGGCCAAATGGCATTGCATTGGTTCGTCAACCAAGCCCCCAAGTGACTCAATGGCCAAGGGCCGATCAAGTGGCATTGCGCTGGTTCGGACACGACCAAGCCCCCAAATGATTCATTGGCTAAAAAGCCGACCAGAAGGCATTACCATTATCAAGACATGACCGAGTTCCCCTAAGGTGTAATCTACGGTTTAAGTCGATTAGGTCCCCAACTGATCCAGTGGCAAATGTGCCAGCCAGAAGGCATTACCGTCGTCAAGACATGACCAAGCCCCCCGAAGGTGTAGCCACGGTTCAGGCCGACTAGGTCCCCAACTGACTTTATAGGGATAGCCACGTTCTCTTTGGTTCGAATACGACCTATGTTTGAACAGCTGTGTTCTCTTTGGTTCGAATACGACCTATGTTTGAACAGGAAGCCCCATGTTTGAATAGCTATGTTCTCTTTGGTTCGAATACGACCTATGTTTGAACAGGAAGCCTCATATTTGAATAGCTATGTTCTCTTTGGTTCGAATACGACCTATGTTTGAACAGGAAGCCTCATATTTGAATAGCTATGTTCTCTTTGGTTCGAATACGACCTATGTTTGAACAGGGAGCCTCATGTTTGAACCGGGATGTTGAACCGGATATTAAGAGCTGCACAGCCTGGTCGGAGACATCTTCCCTTGAACCGGCTTTTAAAACGGAATCTATATCTTGAGCTTTGTGAAGCAACCTCCAGGGGAAAAGACACACATGATGTGAAGAACAGCAGAAACCCCGCTTTACTCGAGGCTCCGGTTTATTGTATTAATCATAATATATACATGGTCATGATATGTACATAAGCAGAGCCCAGGGCTCATGTATAATAAGGCCGTAGATGAGCTATGTTCCACGGCCTGCTGGTCTCCTCCTCTGATGTGCGTGAATCCTTGCGCTCCCGAATATCAATCAGGTAATATGACCCATTATTCAGATTCTTGCTGACCACAAAAGGTCCTTCCCAAGGCGGGGATAATTTATGCTGATCAGACTGATCCTGGATGAGCCGAAGCACCAAATCCCCCTCCTGAAAGGTTCTGGTCCTAACCCGACGGCTGTGGTAACGGCGAAGATCTTGCTGGTAAATCGCTGAACGGGCAGCTGCCAAGTCACGCTCCTCATCCAACAAGTCAAGAGCGTTCTGCCGTGCCTGCTCATTATCCGCTCCAACATATGCTGCCACGCGAGGTGAGTCATGTCGTATATCACTTGGGAGAACCGCCTCTGCTCCATAAACCATAAAAAACGGCGTAAACCCCGTGGATCTGTTAGGCGTGGTGTTGATGCTCCATAATACAGACGGTAGCTCCTCCACCCAACAACCCGGCGTCCTTTGCAAAGGGACTAGAAGCCGGGGTTTGATACCTCTCAAGATCTCTTGGTTCGCTCGTTCTGCCTGACCATTAGACTGTGGGTGCGCTACCGAAGAAACATCAAGCCGTATATGCTCCCGTGCACAGAACTCCTTCATGGCCCCCTTGGATAGATTGGTACCATTATCTGTGATGATGCTGTGTGGAAAACCAAACCGGAAGATCACCTTCTTTATAAATCGGACCGCCGTGGCTGCATCACACTTACTAACTGGTTCTGCCTCTACCCACTTTGTAAACTTGTCCACCGCCACCAGCAGATGGGTCTTCTTATCTTTGGAACGCTTAAAAGGCCCAACCATATCAAGCCCCCAAGTCGCAAACGGCCAAGTGATTGGAATCATCCTCAATTCTTGAGCCGGTATGTGTGCTCGTCTGGAAAATTTCTGGCAACCATCACATAATCTGACCAGGTCTTCTGCATCAGCATGAGCAGTTAACCAATAGAAACCGTGGCGGAACGCTTTAGCCACCAATGATTTTGAACCGGCGTGGTGACCGCAATCCCCTTCATGTATTTCTCGTAATATCTCGCATCCCTCCTTAGGGGATATGCACCGTTGCAGCGCTCCTGATACACTGCGATGATGCAACTCTCCTTGGAAAATGGTCATAGACTTGGACCGTCGTACTATCTGTCGAGCCAAGCTTTCATCCTCTGGCAACTCACCCCGGTTCATATATGCCAGGTAAGGGATCGTCCAATCTGGGGTGGCGTGAAGAGCCGCCACTAACTGAGCCTCCGGATCAGGAACAGCCAACTCCAGCTCACCGGGCAGCTGTACCGATGGGTGGTACAATACATCCAAGAAAACGTTGGGGGGGACCGGTTTACGCTGAGAGCCCAAGCGACTTAAAGCGTCCGCTGCCTCATTCTTTCTGCGGTCCACATGATCCACTTGATAACCTTTGAAATGCCCAGCCACCATATCTACTTCACGTCGGTATGCAGCCATAAGCGGATCCTTAGAATCCCAGGTGCCCGATACCTGCTGAGCCACCAGATCTGAATCACCAAAGCACCTAACTCTGCTCAGATTCATCTCTTTAGCCACCCGAAGACCATGAAGCAAAGCCTCATACTCCGCCGCATTATTTGTACAGGGGAACATTAAGCGGAGGACATAACAAAACTGATCTCCTCGTGGGGAAGTTAAAACGACTCCAGCCCCCGAGCCTTCCAATTGTCTTGACCCGTCGAAATGAATGGTCCAATAGGTGTTATCTGGCTTCTCCTCAGGCGCTTGTAACTCTGTCCAATCGTTTATGAAATCGACAAGTGCCTGTGACTTTATGGCCGTCCGGGGTACGTACCTTAAATCGTGCGGCCCGAGCTCGATAGCCCACTTGGCAATCCGACCGGTCGCCTCCCGGTTCTGTATAATGTCACCCAAAGGAGCAGAACTGACCACCGTGATTGGGTGTCCTTGAAAATATTGCCTCAGCTTCCGGCTCGCCATAAAAACGCCATATACCAGCTTCTGCCAATGCGGATACCTTTGTTTGGACTCGATAAGTACTTCACTGATATAGTAAACCGGCCGCTGGACCGGATGTTCCTTTCCAGCCTCCTTTCGTTCCACCACCATCGCCACACTGACTGCTCTGGCATTCGCTGCAACATATAACAGGAGTGGCTCTTTATCTACAGGGGCTGCCAACACTGGCGGATTGACTAGTTGTCGCTTCAAGTCCTCAAACGCTTTATCAGCCTCCGGACTCCAGACAAACTGATCCGTTTTCTTGAGCATTTGATACAAGGGGATCGCCTTTTCACCAAGGCGACTGATAAACCGGCTTAACGCCGCAATCCGCCCGGCCATGCGCTGCACATCATTGATGCATTTCGGTTTGGCCAGGGAGGTGATGGCTGTGATCTTTTCCGGATTGGCCTCAATTCCTCTATGGGACACTAAAAATCCCAACAGCTTGCCTGCAGGTACACCAAAAACACATTTAGCCGGGTTGAGCATCATTTGGTACACTCTCAAGTTATCGAAAGTCTCCTTCAAATCGTCAACCAGGGTCTCCTTCTTTCGTGACTTGACCACGATATCATCCACGTAAGCATGCACGTTACGCCCAATCTGCTCGTGGAGGCAATTTTGCACACATCGTTGATAAGTAGCCTGCGCACTCTTAAGCCCAAAGGGCATAGATACATAGCAGAAGGCTCCAAAGGGAGTAATGAACGCTGTCTTCTCCTGGTCCTTAACTGCCATCTTGATCTGATGATAACCCGAATAAGCATCCAAAAAACTTAAACGCTCGCAACCTGCCGTAGCATCAATGATCTGATCAATCCGGGGGAGAGCAAAAGGATCTGCTGGGCAAGCTTTATTCAAGTCTGTGTAGTCCACGCACATGCGCCAGGTGCCGTTTTTCTTAAGAACTAGCACCGGATTAGCGAGCCATTCGGGATGAAAAACCTCAATGATAAACCCAGCTGCCAGGAGCCTGGCCACCTCCTCACCAATCGCTTTACGTCTTTCTTCATTAAACCGTCGTAAGAACTGCTTTACCGGCTTGTACTTAGGATCCACATTAAGGGTGTGCTCAGCGAGTTGCCTCGGTACACCAGGCATGTCAGATGGCTTCCATGCAAAGATGTCCCGATTCTCACGGATGAACTCGATGAGCGCGCTTTCCTATTTTGGATCCAAGTTTGCACTGATGCTGAACTGCTTGGATGAATCGCCAGGTACGAAGTCAACAAGTTTAGTTTCGGCCGCTGACTTGAACTTCAAGGCCGGATCATGGTCCGTAGTCGGCTTTTTTAACGAAGTCATATCCTCCGGATCAACACTGTCTTTGTACCGTTTTAGCTCCTCCGCAGCACAAACCGATTCCGCATAGGTCGCATCTCCTTCCTCGCATTCCAAAGCGATTCGACGGCTCCCATGAACCGTTATAGTGCCCTTGTAACCTGGCATCTTAAGTTGCAGATACACGTAGCAGGGTCGTGCCATAAATTTTGCATAGGCTGGCCGTCCGAACAAGGCGTGGTACGGGCTTTGGATTTTAACCACTTCGAACGTCAGCGTCTCTGATCTGGAATCGTGACTATCGCCAAATACCACTTCAAGGGCTATCTTACCAACCGGATATGCAGACTTGCCAGGCACCACCCCGTGGAACACTGTATTGGTCGGTTTGAGATCCTTATCTACCAGTCCCATACGACGGAAGGTCTCGTAATACAAGATGTTAATGCTGCTCCCTCCGTCCATGAGCACCTTGGTGAGCTTATAACCTCCCACCTGCGGCGCCACCACCAACGCTAACTGGCCCGGATTATAAACCTTGGGAGGGTGATCCTCTCGGCTCCATATGATGGGCTGTTCTGACCAGCGCAGATAGTGAGGCGTGGCCGGTTCGACAGCACTGACTGCCCGCCTCTGAAGCTTCCTATCTCGTTTATCCAAACTCGTAGTAAAGACATGATACTGTCCACCACTCAACTGTTTCGGGTTGCTCTGATAACCACTCTGATTACTTTGACTATTCTGATTTTGGCTGACCTGTCCGCCCGGGTTACCCTGGAACCCTGGACCCGAACCTGAACCGCCGCCGGAGCTGCGATCATTCTGGAACGTGTTTGTACTTTTGAACTCCTTCATGATATAACAATCCTTCCAAAGATGGGCTGCTGGTTCCTCCTTTGTCCCGTGCTTCGGACAGGGCTGGTTTAAGAAAAAATTCAAACGCTCCGGATTAGGACCAGGATCTGTGCGGAAACTTGGCTGTTTTCCCTTGCGCCGCTGGCCCTTATTCTGTGCACTTGCGTTGGCTACAAAGTCCATGCTCCCATCCGGTTTACGCTTACCTCCGCCTCCGTTACCTGTCGGCCGATGCTGCTGGCCTTTGACGTTGCTACTCTTCCTTCCCTTCCCTGCTTTGTCATCACCAGAGTCAGGATCCTTGGTACTGTCAGAATCCGCATACTTCACTAAAGCGGTCATGAGCGTTCCTATATCTGTGCAATCACGCTTCATCCGGCCCAGCTTTAGCTTCAAAGGGCCAAACCGACAGTTGCTTTCCAACAGCACTACAGCGGTGTCTGCGTTGATGCGATCTGATGAATGCAAGACTTCTGATACTCGTCGTACCCAATGGGTAGTTGATTCCCCTTCTTGCTGAACGCAGGCTGCTAGGTCAACTATTGACATTGGCTGTTTGCACGTGTCCTTGAAGTTCTTGATAAACCGGGCACGCAATTGGGCCCACGAGCTAATTGAATTAGGTGGCAGACTCTTTAGCCAAGTCCGGGCCGTTCCTTCTAACATCATAGTAAAATACTTGGCACACGCCGTGTCATCCACATCCAGCATCTCCATAGCCATTTCATAGCTCTCCACCCACGTCTCCGGAGGCTGATCTGCCGTGTAGTTTGGTACTTTGCGGGGACCTTTGAAGTCTTTGGGCAGGCGTACATTGCGCAAGGCGGGAACAAGGCACGGTACTCCCAAAGAACTGGAAACCATCCCCGGTTCAGCCGCAGCTGCTGGGAAGGTTGGTGTAATCTGATGAGCCTGATGCTGCATTGCCAATCCGGCCTCCCGTGCACGGGTGCGATCCATATTCTCATGAGCATTGTCAACGCCCGCCGGATTATTGCCGCGGAGCGCTTCACGCCTGGCATTACTTGAAACGGCCGGCTCCTCCACGCGCCTGCTATAGCTCCCGCTGGGGCGAGGGGTGGAGTGAATCCGATCACGGCTTTGCGAATATGCTTCTTGCTGGGCCAATGCGGTCCTAAGGAGCTCCTTGACCCGGCGTGTTTCTTCATCCTGCGGTGAATCTCCTTCGATCGGAATGGCTTCCAACCGAGCCGCCGCCGCGACGAGGTTATCCATCGGGTTAGAATAATGACCTGACGGCGTTGGCATGGCCGGTGGCGTATCAGTGTTATGACGAGGCAGGTTTATAATCCGGTGCTGAACCGGGGCGCCGGTCGTCACCTCCGCACGGTTTACCCCCGGGGTGTTAAAGAGGTTCCGAGCGTCAAGGGCGGCGGGCAACCGAGACCGGGATTTTCTCTTCAGGACCTCCTGAGACGCCTTCTGGTCCATCATGAGCCTGTAAGCCTGTGCATCTAGAGCGGCGCGCTCCGCCGTCATCCTGATCTCTTCTGCTACCAGCTCCGCTTTAGCCCGAGCGATCTGCTCTCTCACTTTGGCAATCTCAGCATTGTGAGCCTCCAGATCCACCGGGTTGGCTTCCGCTATGAGCGCTGCTAGTGCGTCAAACAGGTCTGACAAAACTTGGGCTGGTGAACCTGTAGGGCCTCCCGCCCGCGCAGCCGTTGCTGCTGCCGATCCGGAGATCGCTGCTACTACAGTTGATGATCGTGGCGCTGCCTGTGTTCCAGTCATGAAGATCCCGACCCGACAGGGCAGATCCGAGGGGTCCGGAATACTGTTGCCATCGGAACCGCCCTCAATCCGATCATCTTGTAGCTGGTAAAGAGATTCGGTTTCCCCGGTCGACGTTTCATCGCCGGAGTAGATGGCGGTTTCGTCACCAGATTCGGATCCTTCCTCGTAGCTTCCTCCGCGGATGACCCCCATGAAGGCGTGCTTTCTGATCGGTTTAGCCAGGGTCGATCTCGTGCACTGAGCTGTCTCGATGAGGTCGGCGCAGACGCCTAACTCGGGGCCCGGTTCTCCGATCTTGCCGATGAAAACATGGATGCCGCCAAAGGGGACCCGGTACCCGTACTCGATCGAGCCGGCGTCGGGGCCCCAGCCTGTGCTGTCGATGTAGAGTTTTCCGCGGCGGCTCTTGGTCATCCGTCCCACAGCGTAGCCCTCGAGTCCTTCAAAGCGGCCCTCCAAGAACCTGAAACCATCTCGCGATAGCCCCACGGTGGGCGCCAAC

TGTCGTGGATTTGTCACGGCAGATGTCCTAGGAAGAGGACTTAGTCGTGGAGCCATCGCAACGGGTTAGCTTGAAGGGGTTAAAGTGGACGCGAGGACACGGTTTTTATACTAGTTCGGCCCCTTCGATGAAGGTAAAAGCCTACGTCTAGTTGTGATGGGATTGATGGGTTTCGAGGGCTTAAGGAGCGATATGCTTCGCCTAAACCTCGAATTCTTCTTCTCTGTCCCCAGACCGCCGCCGGGTCATCCCCTTATATATACGGGTGACGCCCGTCGGTCTGCAAAGTCCCAACCTCCGGCTTATAAACGTGCCCGGGTCGGTCTCTCTATTCCTAACTTACAATACAAGTTTACATCAGGCCGGTTTATACCATAAACCTTTAAACCGATTATGGGCCTTAGGCCCTTCTATCTCTCCTTGGGCTTTAACATCTCGGGTCTGTGGACGGAGGTAACCCGGGCCCTGCAGGCCGGTTTACCCCGGATAGCAACATCCCCAACA

AAATGCTTGCTGATGTCCACGAGCCAATCATCTACGTCTATGGCCTCGACACAGTAGCTGAAGGTCTTTGGCTGATTTGCAAGGAACTGGTTGAGTGAAGCGAAATGCGGCTGATTGTTGCCCT

>Aegilops umbellulata 1-260;

CGGGTGCTTCGGGACTCGGGCAAGGTGCTGCGTGGAGAGCTCCTCCAATAGAGAAGACTCCTCCTCGGGCGGGAGTGACGAGGAGGACAGCGACAGCGACGACGAGGGTAGCGGGCGGCAGGAT

TGTTGGGGATATTGCTATTTGGGTAAACCGGCCTACAGGGCCCGGGTTAGCTCCGTCAATGGACCCGAGATGTTAAAGCCCAAGGAGAAATAGAAGGGCCCAAGGCCTATAATCGGTTTAGAGGTTTACGGCCGTAAACCGGCCTGAGGTAAACTTGCATTGTAAGTTAGGAATAGAGAGACCAACCCGGGCACGTTTATGAACCGGAGGTTGGGACTTTGCAGACCGACGGGTGTCACCCGTGTATATAAGGGGACGACCCGACGGCGGTTTGAGGGGAGAGAACAACAACTCGAGGTTTAGGCGAAGCTTATCGCTCCCTAGCCCTCGAAACACATCAATCCCATCACAAACTAGACGTAGGCTTTTACCTTCATCGAAGGGGCCGAACTAGTATAAACACCGTGTCCTTGCGTCCGCTTTTAACCCCTTCAAGTTAACCCGTTGCGATGGCTCCACGACTAAGTCCTTTCGCTAGGACATCTGCCGTGACAAATCCACGACA

GTTGGCGCCCACCGTGGGGCTATCGCGAGATGGTTTCAAGTTTTTGGAGGGCCGCTTTGAAGGACTCGAGGGTTACGCTGTGGGTCGGATGACCAAAAGTCGTCGCGGGAAACTCTACATCGACAGCACAGGCTGGGGCCCCGACGCCGGCTCGATCGAGTACGGGTACCGGGTCCCCTTTGGCGGCATCCATGTTTTCATCGGCAAGATCGGTGAACCGGGCCCAGAGCTGGGCGTCTGCACCGACCTCGTCGAGAAAGCTCAGTGCACGAGGTCGACCCTGGCTAAACCGACCAGGAAGCGCGCCTTCGTGGGAGTCATCCACGGAGGAAGCTACGAGGAAGGATCCGAATCCGGTGACGAAACCGCCATCTACTCCGGCGATGAAACGTCAACCGGGGAAATCGAATCTCTATACCAGCTGCAAGATGATCGGATTGGGGGCAGCTCCGATGGCGACAGTATTCCGGACCCCTCTGATCTGCCTTGTCGGGTTGGGATCTTCATGGCTGGAACACAGGCGGCGCCACATTCATCAACTGCGGCAGCAGTGATCTCCGGATCGGCAGCGGCAACAGCTGCGCGGGCAGGAGGCCCTGCGGATTCACCAGCCCAAGTATTGTCAGACCTGTTTGACGCACTGGCAGCGCTCATGGCGGAAGCCAACCCGGTGGATCAGGAGGTTCACAATGCTGAGATTGCCAAAGTGAGAGAACAGATCACTCAAGCTAAAGCGGAGCTGGCAGCAGAAGAGGCCAGGATGACGGCGGAACGCGCCGCTTTAGATGCACAGGCTTACAGGCTCATGATGGACCAGAATGCGTCTCAGGAAGTCCTGAAGAGGAAATTCCGATCTCGATTGCCCGCAGCCCTCGATGCCCGAAATCTCTTCAACACCCCGGGGGTAAACCGTGCGGAGGCGACAGTTGGCGCCCCGGTTCAGCACCGGATAATAAACCTGCCTCGTCACAACACTGGTACACCACAGGCCGTGCCAACACCGTCGGGCCATTATTCCAACCCGATGGATAACCTCGTCGCGGCGGCAGCTCGGTTGGAAGCCATTCCGATCGAAGGCGATTCACCGCAAGATGTAGAGACACGCCGGGTCAAGGAGCTCCTTAGGACCGCGTTGGCCCAACAAGAAGCATATTCGCAAAGCCGTGATCGGATTCATTCCACCCCTCGCCCCAGCGGGAGCTACAGTAGGCGCATGGAGGAGCCGGCTGTTTCAAGTAATGCCAGGCGTGAAGCGATCCGCGGCAACAATCCGGCGGGCGTCGACAATGCTCAGGAGAATGTGGATCGCGCCCGTGCGCGGGAGGCCGGATTAGCAGCGCAGCATCAGGCTCGTCAGCTTACACCTATCTTCCCAGCTGCTACGGTTGAACCGGGGGTGGTTTCTAGTTCTTTGGGAGTACCGTGCCTTGTTCCCGCTTTGCGCAACGTACGCCTGCCCAAAGACTTCAAAGGTCCCCGCAAGGTACCAAACTACACGGCAGATCAGCCTCCGGAGACATGGGTGGAGAGCTATGAGATGGCTATGGAGATGCTGGATGTGGATGACACGGCGTGTGCCAAGTATTTTACTATGATGTTAGAAGGAACGGCCCGGACTTGGCTAAAGAGCTTACCGCCTAATTCCATTAATTCATGGGCCCAGTTGCGCGCCCGGTTTATCAAGAATTTCAAGGACACGTGCAAACAGCCAATGTCGATAGTTGACCTAGCAGCTTGCGTTCAGCAAGAAGGGGAATCAACTACCCATTGGGTACGTCGGGTATCAGAAGTTTTGCACTCGTCAGATCGCATCAACGCAGACACCGCCGTGGTGCTATTGGAAAGCAACTGTCGGTTTGGCCCTTTGAAGCTAAAGTTAGGCCGGATGAAGCGTGATTGCACGGATATGGGAACGCTCATGACCGCTTTAGTGAAGTATGCGGATTCTGACAGTACCAAGGATCCTAACTCTGGTGACGACAAAGCAGGGAAGGGAAAGAAGAATAGCAACACCAAAGGCCAGCAGCATCGACCGACAGGTAATGGAGGCGGAGGTAAGCGTAAACCGGATGGGAGCATGGATTTTGTGGCCAACACAAGTGCACAGAATAAGGGCCAGCGGCGTAAGGGGAAACAGCCAAGTTTTCGCGGAAATCCCGGTCCTAATCCGGAGCGTTTGAACTTTTTCTTAAACCAGCCCTGTCCGAAACACGGAACAAAAGAGGAGCCAGCAACCCATCTTTGGAAGGATTGTTACATCATGAAGGAATTCAAAAGCTCAAACACTTTCCAATATGATCACAGCTCTGGCGGCGGTTCAGGTTCCGGATCAGGATGCGGCGGCGGAAATTCCGGTCCAGGGTTCCATGGTAATCCGGGCGGACAGGACAACCAAAATAATCAAGGTAATCAAAGTGGTTACGGACAGCAGCAATCAGGTTATCAGAGCAACCTGAAACAGTTGAGTGGTGGACAGTATCATGTCTTTACTACAGGCTTGGATAAACGGGATAGAAAGCTTCAGAGGCGGGCAGTTAGTGCTGTGGAACCGGCCACGCCTCACTATCTGCGCTGGTCGGAACAACCTATCATATGGAGCCGAGAAGATCACCCTCCCAGGGTTTATAATCCGGGTCAGTTAGCATTGGTGGTGGCGCCTCAGGTGGGAGGTTATAAGCTCACCAAGGTGCTCATGGACGGAGGGAGCAGCATTAACATCTTGTATTATGAGACCTTCTGTCGCATGGGACTGGTAGATAAGGATCTCAAACCGACCAATACAGTGTTTCACGGGGTGGTGCCTGGCAAGTCGGCGTACCCAGTTGGTAAGATAGCCCTTGAAGTGGTCTTCGGCGATTGTCATGATTCCAGATCAGAGACGCTGACGTTCGAAGTGGTTAAAATCCAAAGTCCATACCACGCTTTGTTCGGACGGCCAGCCTATGCAAAATTTAAGGCACGACCCTGCTATGTATATCTGCAACTCAAGATGCCGGGTTACAAGGGCACTATAACGGTTCATGGGAGCCGTCGAATCGCTTTGGAATGCGAGGAAGGAGATGCGACTTATGCAGAATCGGTTTGTGCTACGGAGGAGCTAAAGCAGTACAAAGACAGTGTTGACCCGGAGGACATGACTTCGTTGAAGAAGCCAACTACGGACCACGATCCGGCCTTGAAGTTCAAATCGGCTGCCGAAACTAAGCTTGTTGACTTCGTACCTGGCGATTCGTCCAAGCAGTTCAGCATCAGTGCAAACCTGGATCCAAAATAGGAAAGCGCGCTCATCGAGTTCATCCGTGAGAATCGGGACATCTTTGCATGGAAGTCGTCTGACATGCCTGGTGTACCGAGGCGACTCGCTGAGCACACCCTTAATGTGGATCCTAAGTATAAACCGGTAAAACAGTTCTTACGCCGGTTTAACGAAGAAAGACGTAAAGCGATTGGTGAGGAAGTGGCCAGGCTCCTGGCAGCTGGGTTTATTATTGAGGTTTTTCATCCCGAGTGGCTTGCTAATCCGGTGCTGGTTCTTAAGAAAAACGGCACCTGGCGCATGTGTGTGGATTACACAGACTTGAATAAAGCTTGTCCAGCGGATCCTTTTGCTCTCCCCCGGATTGATCAAATCATTGATGCTACGGCAGGTTGCGAGCGTTTGAGTTTTTTGGATGCCTATTCGGGTTATCATCAGATCAAGATGGCAGTTAAGGACCAGGAGAAGACGGCGTTCATTACTCCCTTTGGAGCCTTCTGCTATGTTTCTATGCCCTTCGGGCTTAAGAGTGCGCAGGCTACTTATCAACGGTGTGTGCAAAATTGCCTCCACGAACAGATTGGTCGGAATGTACATGCTTATGTGGATGATATTGTGGTCAAGTCACGAAAGAAGGAGACCCTGGTTGATGATTTGAAGGAAACCTTCGATAACTTGAGAGTGTACCAAATGATGCTCAACCCAGCTAAATGTGTTTTTGGTGTACCTGCAGGCAAGTTGTTGGGATTCTTAGTGTCCCATAGAGGAATTGAGGCCAATCCGGAAAAAATCACAGCCATCACCTCACTGGCCAAACCGAAATGTGTCAATGATGTTCAGCGCATGGCCGGACGGATTGCAGCGTTAAGCCGGTTTATCAGCCGCCTCGGTGAAAAGGCGATCCCCTTGTATCAAATGCTCAAGAAGACAGATCAATTTGTCTGGAGTCCGGAGGCTGATAAAGCGTTTGAGGACTTGAAGCGACAGTTAGTCAATCCGCCAGTGTTGGCAGCCCCTGTAGATAAAGAGCCACTCCTGTTATATGTTGCTGCGAACGCCAGAGCGGTCAGTGTGGCGATGGTGGTAGAACGCAAGGAGGCTGGGAAGGAACATCCGGTTCAGCGACCGGTTTACTATATCAGTGAAGTGCTTATCGAGTCCAAACAAAGGTATCCGCATTGGCAGAAGCTGGTATATGGTGTTTTTATGGCAAGCCGGAAGCTGAGGCAATATTTTCAAGGACACCCCATCACGGTGGTCAGTTCTGCTCCTTTGGGTGACATCATACAGAACCGGGAGGCGACCGGTCGGATTGCCAAGTGGGCTATCGAGCTTGGGCCACACGGTTTAAGATACGTACCCCGGACGGCCATAAAGTCACAAGCACTTGTCGACTTCATAAACGATTGGACAGAGTTACAAGCACCTGAGGAGAAGCCAGATAACACATATTGGACGATTCATTTTGACGGGTCCAGACAATTGGAAGGCTCGGGGGCTGGAGTCGTTTTAACTTCCCCACGAGGAGATAAGTTTTGTTATGTCCTCCGTTTAATGTTCCCCTGTACAAATAATGCGGCTGAGTATGAAGCTTTGCTTCATGGTCTTCGGATGGCTAAAGAGATGAATCTGAGCCGAGTTAGGTGCTTTGGTGATTCGGATCTGGTGGCTCAGCAGGTATCTGGCACCTGGGATTCTAAGAACCCGCTCATGGCTGCATACCGTCGTGAAGTAGATATGGTGGCTGGGTATTTCAAAGGCTATCAAGTGGACCATGTGGACCGCAGAAAGAATGAGGCAGCGGACGCTTTAAGCCGCTTGGGCTCTCAGCGTAAACCGGTCCCACCCAATGTTTTCTTGGATGTATTGTATCACCCATCGGTACAGCTACCCGGTGAGCTGGAGTTGGCTATTCCTGATCCGGAGGCTCAATTAGTGGCGGCTCTTCACGTCACCCCGGATTGGACGCTTCCTTATCTGGCATATATGAACCGGGGTGAGTTGCCAGAAGACGAAACCTTGGCTCGGCAGATAGTACGGCGGTCCAAGTCTATGACCATTCTCAAAGGAGAGCTACATCATCGCAGCGTGTCAGGAGCGCTGCAACGATGCGTATCCCCTGAGGAGGGGTGCGAGATATTACGTGAAATACATGAAGGGGATTGCGGCCACCACGCCGGTTCAAAATCATTGGTGGCTAAAGCGTTCCGCCACGGTTTCTACTGGTTAACTGCTCATGCTGATGCAGAAGACCTGGTCAGGTTATGTGATGGTTGCCAGAAATTTTCCAGACGAGCGCACATACCGGCTCAAGAATTGAGGATGATTCCAATTACTTGGCCGTTTGCGACTTGGGGGCTCGATATGGTTGGACCTTTTAAACGTTCCAAAGATAAGAAGACCCACCTATTGGTGGCGGTTGACAAATTTACAAAGTGGGTGGAGGCAGAACCTGTCAGTAAATGTGATGCGGCCACGGCGGTGCGGTTTATAAAGAAGGTGATCTTCCGGTTTGGTTTTCCACATAGCATCATCACAGATAATGGTACCAATCTATCCAAAGGTGCCATGAAAGAGTTCTGCGCACGCGAGCATATACGGCTTGATGTTTCTTCAGTAGCTCACCCACAGTCTAATGGTCAAGCAGAACGAGCGAATCAAGAGATCTTGAGAGGTATCAAACCCCGGCTTCTGGTCCCTTTGCAAAGGACGCCGGGTTGTTGGGTCGAGGAGCTACCGTCTGTATTATGGAGTATCAACACCACGCCTAACAGATCCACAGGGTTTACGCCGTTCTTTATGGTCTATGGAGCAGAGGCGGTTCTCCCCAGTGATATACAACATGATTCACCTCGTGTGGCAGCTTATGTTGAAGCGGGCAACGAGCAGGCACGGCAGGACGCTCTTGACTTGTTGGATGAGGAACGTGAATTAGCAGCTGCCCGTTCAGCGATTTACCAGCAGGATCTTCGCCGTTACCACAGCCGTCGGGTTAAGACCAGAACCTTTCAGGAGGGGGATTTGGTACTTCGGCTCATCCAGGATCAGTCTGATCAGCATAAATTATCCCCGCCTTGGGAAGGACCTTTTGTGGTCAGCAAGAATCTGAATAATGGGTCATACTACCTGATTGATATTCGAGAGCGCAAAGATTCACGCACATCAGAGAAGGAGACCAGCAGGCCGTGGAACATAGCTCATCTTCGACCTTATTATACATGAGCCTTGGGCTCTGTTTATGTACATATCATGACCATGTATATATTATGATCAATACAATAAACCGGAGCCTCGAGTAAAGCGGGGTCTCTGCTGTTCTTCACATCATGCGTGGTTACATGGAGTTGATTCGCTTTAATGCAACTTCTGGTTTACCCCTTGAGGTCGCTTCATAGAAAATCAAAATGAAGATTCTGGTTTAAAATCCGGTTCAAGGGAGATATGTCTCCCACAAAGCTTTGAAACTCTTAAATATCCGGTTCAAGCTCCCGGTTCAGGAAAAAATTATCTCTTGCAAAAAGGTTAAAAATTGCTGAAGAGGACCTGCTGATATGATGCGCGGTTCAAACATGGGGCTTCCTGTTCAAACATAGGTCGTATTCGAACCAAAGAGAACATAGCTATCCTTACAAAGTCAGTTGGGGACCTAGTCGACCTGAACCGTAGCTACACCTCCGGGGAGCTTGGTCATGTCTTGACGACGGTAATGCCTTCTGGCTGGCATATTTGCCACTAAGTCAGTTGGGGACCTAATCGACTTGAACCGTAGGTACACCTTATGGGAGCTCGGTCATGTCTTGATGATGGTAATGCCTTCTGATCGGCTTTTAAACAGCCAATAAGTCATTTGGGGGCTTGGTCGTGTCCGAACCAATGCAATGCCACTTGATCGGCCCTAGGCCACTGAGTCACTTGGGGGCTTGGTCGAATCCGAACCAATGCAATGCCATTTGAACGGCATATTTGCCACGATTCAGTTAGGAACCTAGTCGGCTTAAACCGTAGCTACACCCCTTGGGGGCTTGGTTTTTTGTCTTGACGGCGGTAACGCCTTCTGAACGGTTCATAGCAACCACCCTTTGCATGCGTTTTATTATTGCGTTCTTCTCTCGTATTTGGTTGTTTGTTACCTTCCTTCTTGCGGTGTTTTTTGTTCTTTGGAACGACAAGTACTTTTAAACCGATTCAGCTGACACAGCCTGGTCCATTTTTTAACCCGGAGGCAAGTTGCCCGGTTTGGCAAAGGCGCAGCACGGTTCGTATGGTCGAAAGACCGTCCTTTGTACCCAGTAAAGACAAGAAACACAAGTTGGAAAAAGTCATGGCAAGGGCATCAGTACCCGTGCACAAAGGCGCGTTCAAAGGCAGAGGTATTAACTATCTTATTACAAGGCAGCGTAGTGCCTGAATATATTGTTTTTTGACGGATTAAACGTCACCAATTAAGGTGAACCGGAGCGTTGATTACGCCTGTTCGCCGCCTCGGTTCGAAGGCTGTGGATCATCTTGCGCCGCTTTAGCTTTCTCTTGGCTACCCATTGGCTGGAAATCAGCAGTGGTCCAGTCGATTCCCATTAGCGCTTGAAAGACAGCTTCTTCATGAATCAGCAAGGACGGTTCAATGTCAGGGGCGTAGGTATGCTTACGGATTGGAGGAATAAGGTTTTCCGCTTCAGGGACCGGGGCAGCTACTCGCTTGTTCTGGTTATCATATTGGGCCTGATAATGTGTCAGATCTGCTTCTTCAGCCAATTGACAAGCTAGGGGGCGCACCGCCCGGTTTATCGCCCTCAAGTCATTTTCGCCGAAATCTGAACCGTCTTCTTTCAAGCTGGGGTATCCTTGGGCCGCTTCGACAGGATTAAAATCTGGTACCCACGCTTTGGCCCGGATCAAGGCAGTGATGGCTCCGGTTCGAGCAGCTGATCTTTTTAACTCTTCAATCCGGGCTGGAAGCATGGATAACCTTTTTAACGTGTCTTGAATCAGATACGGTGCAGGGTTGTTATGCGATGTAGTGGTGATAGCTCTCTGTGCTCCGGTGTACAACTGTTCAATCAAAGTATAGGCAGCTTTGAGTTTCATCCGCACATCAGAACCCAAGTGTCCGATACGTGTGCCTGCAGTGTTTCAAGATGGATTACAAACCGGACATAGGATATAGCAGACAAGGTACAAATTGGAAGAAGGGCATCGAAAGAAATTTACCAAAGACGGCAGAGGTCATGGAGTGTATTTGTCGCTTTACGGTGGATAATTCATCAACCACCGGTTTGAGAGCAGCTTCGGCATTTTCAGCCCTTTTCATCAAAAGAGCCTTTTCAGTAGCCCAAGCAGCTTGTTCCTTTTTGCGGCTTTCTTTCAGCTGTTCCATAACGGTTAAGGCATTGGTCAATTCCTCCTTCGCTTTTGCAGTTTCAGCCTGCTGGGTTTTTAGGGTTTCCTGAAGGTCCGAGACTTGTTTCTCTTTGGTCTTCAGATCCCCCTGCAGCAGGTCGTCAGCAGTGTTCAGAAAATATTGCACGGTTTGGAAGTATCAACCACACAAGCAAGTTATGTGCTCGATACTTGGGGGCTAATGCATACTTGATCTTAACGCATGTTTTCCATTACAAAAAGTCCCAGGATTAATACAAGTATTTAAACTTGGTACGTGGGGGCTAATGGTTATTTGATGATGTTTCTGGTTAACGGCCTATGTTGAAGCCCTGGGTTTTTAAACCGGGCCTTAGTTGTCAAGGCAGCATGATTCGGATTACCATCTCGGATTGGAAAAAATTGAGAAGCTAGGTTGCAACAGAATGAAGCACAAGAGAGTTACCTCAAGTTTTTCTTTCATCATCCTGATCATACCAGCTTCATAATCGCGACTGGTGTACAGTCGGTTCAGATACCCAGAATAAAGATCTTGGGCGGTTAAAGCGGCGTACGTTGAGGAATCAACGTCCCATTTGCCCTTTATGGCCGAGATTTCTTCCTTGGCAGTATGCTTGGCTAAAGTAGCAGAGTTCCCCGGTTCAGAGCGACCAATGCCGGTAATCACAACATCATCATCTTTGGAGTCGCTGCTCTGCATTGGCACTGTCGGTTTATCAACAGTTCCAGAAGGGGGGATGGATTCCTCAACCCGAACAGGACTGGCAGGCTGATCAGTACGGCTGGCAGGATTGGTAGGCCTTTCTTCAACATAGGCGTCATCTTGTGGAGGTAGATAATTCTGAATGTCTTCACGAACTAGAGCATCAGCACCAGGAGTTCTCTCCGGTTCAGGAGCGGCAGTTCCTTCGGCCGGATTATCCAGACGAGCCTTCTTGCTCAGTCTAGGTTTAGCCCTGGGAAAGACCCAAAGATTGAAAGAACGATGTAAGATGGATTAAGAACCCCATATAGAAATATGTTGTGATCGGTCCAACTTACCCAATAACTGTCTTTAGCGGTGGCAGTTGAGTGGCTGAAGATTCACCAGAAGATGAATGGGAAGTGACCTGATAATCGGAGTCAGATGGATTAAGAGGCTGACGAGTTAAACCTGCTTTAGGAGGAAAGCTAGCGCATAAATCGGAGACCTCTGAGCGGCGTTTCCGAACCGGGCTGTTCGGTAAACCGGCGGAGGTAACTACCTGGCCGCTATGCCGGGTGGTGCGTCGAGCTTCATGTTGCTGGGTCTTCACAAGAAATTTGGGATCCAAGTAAGCAAGAGGATGAGAAAATTTAATTTTCTGGTTTGCTTGGCGAGGTTTTTTCAAAGGCAAAGGAACTGCATCAGAAGAAAGAATAGTTACCTCTGGGCCTTCAGCATTACTCGCTTCAGCGTCGTCCTGATAAATATCATCATCAATAAGACGTGTAAAAAGTAAGCCAACTGAATCGAGGTCTACCTCAAGGTCCGGATTACCCACATCATCCTCCTCTAAATCCGGGGTAGAGGAAGCGGTGTTCCTTTTCTTCTGAGCGGGCTTCTTCACCCTTGTTTTGGGTCGGGTTACCTTTTCGGTCGGCTCTTGAAACTTCTTGCTCCAAAACGGATCATCTCCCTGTCTCAGAAAGGACTGGAGTTAACAATATGTATATAAGGAGATCATAAATTCTATAACAAACAGAGGTGAGATGCTTACAGCAGGTGGTTTGTTTGTAGCACAAAAGGGAAGCAGGCCGGTTCGGGCGCAAACATATTCCGGTTCATTCAACATCTTTTTCACAGCTTCAGTAACTTCGTCTTCCGTCAGCTGAATGTTGCAGTGCCTCAAAGGATCATCAACCCAGCCCGTGTATTGGCACATTAAACCGGAGCGGATGCTAAGGGGCAGGATGCTCCATGATATCTAGCAACGGGCAAGATCTACCCCCGTTAAACCGTTAGCCATGAAGGCTCTGAGCTTGGACAGCTGAGGGGCATACTTACTCCTCTCTTTGGCGGTTAGCCTTGGGGGGAAGGGGTGTGTATTTCTAAGTCTCTCTGGACGGAAGCCAGGCAGAGGATTTTCTTCGGCAGGGGAAGTATCCTTGCAATAGAACCAAGTGTGATTCCATTCTTGCGGATGACTATGCAACTTGGCGTGAGGATAAGTCACTTCTTTCCTCTTTTGAATCGCCATACCACCCAGTTCCATATTAGGGCCGTCGGTGAATTCAGTACGTCGGTTCAAATGAAAACAGTCTCGGAACAGCTCTACAGTAGGTTCCTCCTGGAAGTACACCTCACAAAGCACTTGAAAATGGCAGATATTCGTGACTGAGTTTGGACCAACATCTTGTGGGCGCAACTTAAAATTAGCCAGGACGTCCCGATAAAATTTTGAACCGGGTGGATTAAAACCCCGGGCCAGGTGATCCGCAAACACCACCACTTCTCCTTCCTGTGGGGTAGGAGGACATTCATCTCCTGGAACCCTCCAATGGATGGCATTTTTGCTACTCAAAGCGCCAGTCAGAACTAAGTTGTTTAATTGGGTCTCGGTGATGCGAGAGGGAACCCAGTTGCATTCGTACACCTGTTTTGCCATAAGGAAATCTACAAGGCAAAGGTATTCCGGTTTGAGAACAGGTGTGATTGTAAACCGAAGATGCTCTTATATAGAATGAAGTTATCTACAATGATATCATCTGACGGTTCATTGAGGGGACTAATGGATATATGGTTTGGGTTGTTCGCTCTGCTAAAAGTTAAACCGACCGGATCTAACAGTTGTGAATATGAAAGCAGAAACAGATCCCGTACGGATTCTTTCAAAACAATGGATCTACAGCTAGGGGAAAAAAGAAGTAAAAATACGAGGTTTGGCTCACAGCAGAACAGTATAAGTTCGACAAGCACGGAAATGAATGGGATCTAAGGAGATGAGTGCAGATTTATGGCGAACATTGAGATTGACTACAAATCACAATATAAGTATCGTAATCTGTTCGCAAAAGCTGCTATGGAGATGAACAAGGAACAGTGGCAGGATGACGAACCCTAGAACAGATCTAAAGCAAGAAGAACAGGGGCCTTACCGGGATCCAAACGGATGTGGAGGGTCGCCGCGGTACTCTGGTCCGGTCAGGTCGATGCAGCGGCCGGAGTTGATGCAGTAGCTGACGGCGGCGGCGGAGCTCCGAGGTCTGGGTCGCGAGGAAGACGAAGAAGGGGACGAAAGGGAGGAAAGGAAGATGGCCTTCAAGTCCTATTTATAAGGCGCGGCGCGCGTACCAGGCGCGCGAATCCAGGGGCCGGTGATTTCGGGAGCCCAGCCGTCGCCCTGATTATTGCGGATTAGCAAAAGGAGACGGTAGCTTTGCGCTGACGTCATATCGGTTTACCGCAGCAAGAGGAGACGAAGAAACGGCGGTTTAACAGTTACTGGAAGACATTTGAAGACAGAAATTCTTATTAAGGATTGACATGAACCTGTTCAAATCAATCTGGGGCCTAA

TGTTGGGGATATTGCTATTTGGGTAAACCGGCCTACAGGGCCCGGGTTAGCTCCGTCAATGGACCCGAGATGTTAAAGCCCAAGGAGAAATAGAAGGGCCCAAGGCCTATAATCGGTTTAGAGGTTTACGGCCGTAAACCGGCCTGAGGTAAACTTGCATTGTAAGTTAGGAATAGAGAGACCAACCCGGGCACGTTTATGAACCGGAGGTTGGGACTTTGCAGACCGACGGGTGTCACCCGTGTATATAAGGGGACGACCCGACGGCGGTTTGAGGGGAGAGAACAACAACTCGAGGTTTAGGCGAAGCTTATCGCTCCCTAGCCCTCGAAACACATCAATCCCATCACAAACTAGACGTAGGCTTTTACCTTCATCGAAGGGGCCGAACTAGTATAAACACCGTGTCCTTGCGTCCGCTTTTAACCCCTTCAAGTTAACCCGTTGCGATGGCTCCACGACTAAGTCCTTTCGCTAGGACATCTGCCGTGACAAATCCACGACA

AGGATGACGGGTCCGACTAGGTGTCGGGGGCCGCTCCATGCGGCACCGGCGACCCCATCATCCGTGTCGCCGATTCCTTGAACTTCTCGGGGCCGTCTCCTTGGGCGGCTGGCGTAGGGAGGCT

>Aegilops umbellulata 1-299;

ATGAGGCCTCATCGCCCCCTACTACTCCCACCGCTAGCCAGGCCCTGCGTCGACGGCAGCCTCACACCGCAGCCGAACCAGTGAACCCTCGTACTCCTCTCCGCGTGGGCATCCACTCGCGCGT

TGTTGGGGATGTTGCTATCCGGGGTAAACCGGCCTGCAGGGCCCGGGTTACCTCCGTCAACAGACCCGAGATGTTAAAACCCAAGGAGAGATAGAAGGGCCTAAGGCCCATAATCGGTTTAAAGGTTTATGGAGTAAACCGGCCTGATGTAAACTTGTATTGTAAGTTAGGAATAGAGAGACCGACCCGGGCACGTTTATTAGCCGGAGGTTGGGACTTTGCAGACCGACGGGCGTCACCCGTGTATATAAGGGGATGACCCGGCGGCGGTTCAGGGACAGAGAAGAAGAACTCGAGGTTTAGGCGAAGCATATCGCTCCCTAAGCCCTCGAAACCCATCAATCCCATCACAACTAGACGTAGGCTTTTACCTTCATCGAAGGGGCCGAACTAGTATAAAAACCGTGTCCTTGTGTCCACTTTAACCCCTTCAAGCTAACCCGTTGCGATGGCTCCACGACTAAGTCCTCTTACTAGGACATCTGCCGTGACAAATCCACGACA

GTTGGCGCCCACCGTGGGGCTATCGCGAGATGGTTTCAGGTTCTTGGAGGGCCGCTTTGAAGGACTCGAGGGTTACGCTGTGGGACGGATGACCAAGAGCCGCCGCGGAAAACTCTACATCGACAGCACAGGCTGGGGCCCCGACGCCGGCTCGATCAAGTACGGGTACCGAGTCCCCTTTGGCGGCATCCATGTTTTCATTGGCAAGATCGGAGAACCGGGCCCCGAGTTAGGCGTCTGCGCCGACCTCATCGAGACAGCTCAGTGCACGAGATCGACCCTGGCTAAACCGACCAGGAAGCGCGCCTTCGTGGGAGTCATCCACGGGGGAAGCTACGAGGAAGGATCCGAATCTGGTGACGAAACCGCCATCTACTCTGGCGATGAAACGTCGACCGGGGAGACCGAATCTCTTTACCAGCTACAAGATGATCGGGTTGGGGGCGGCTCCGATGGCAACAGTATTCCGGACCCCTCTGATCTGCCCTATCGGGTTGGGATCTTCATGGCTGGGACACAGGCGGCGCCACGATCATCAACTGCAGCAGCAGCGATCTCCGGATCGGCAGCAGCAACGGCTGCGCGGGCAGGAGGCCCTACGGGTTCACCAGCCCAGGTTTTGTCAGACCTGTTTGACGCACTAGCAGCGCTCATGGCGGAAGCCAACCCGGTGGATCAGGAGGTTCACAATGCTGAGATTGCCAAAGTGAGAGAGCAGATCACTCGGGCTAAAGCGGAGCTGGCAGCAGAAGAGATCAGGATGACGGCGGAGCGCGCCGCTTTAGATGCACAGGCTTACAGGCTCATGATGGACCAGAAGGCGTCTCAGGAGGTCCTGAAGAGAAAATCCCGGTCTCGGTTGCCCGCCGCCCTCGACGCTCGAAATCTCTTCAACACCCCGGGGGTAAACCGTGCGGAGGCGACAGCCGGCGCCCCGGTTCAGCACCGGATTATAGACCTGCCTCGTCATAACACTGATACGCCACCGGCCATGCCAACGCCGTCAGGTCATTATTCCAACCCGATGGATAACCTCGTCGCAGCGGCGGCTCGGTTGGAAGCCATTCCGATCGAAGGAGATTCACCGCAGGATGAAGAGACACGCCGGGTCAAGGAGCTCCTTAGGACCGCGTTGGCCCAGCAAGAAGCGTATTCGCAAAGCCGTGATCGGATTCACTCCACCCCGCGCCCCAGCAGGAGCTATAGTAGGCGCGCGGAGGAGCCGGCCGTTTCAAGTAACGCCAGGCGTGAAGCGCTCCGCGGCAACAATCCGGCGGGCGTCGACAATAATCACGAGGATGTGGATCGCACCCGTGCACGGGAGGCCGGATTAGCAGCGCAGCATCAGGCTCGTCAGCTTACACCTATCTTCCCAGCAGCTACGGTTGAACCGGGGGTGGTTACCAGTTCTTTGGGAGTACCGTGCCTTGTTCCCGCTTTACGCAACGTACGCCTGCCCAAAGACTTCAAAGGCCCCCGCAAGGTACCAAACTACACGGCAGATCAGCCTCCGGAGACATGGGTGGAGAGCTATGAAATGGCTATGGAGATGCTGGATGTGGATGACACGGCGTGTGCCAAGTATTTCACTATGATGTTAGAAGGAACGGCCCGGACTTGGCTAAAGAGTTTACCGCCTAATTCAATTAGTTCATGGGCCCAATTGCGCGCCCGGTTTATCAAGAATTTCAAGGACACGTGCAAACAGCCAATGTCAATAGTTGACCTAGCAGCCTGCGTTCAGCAAGAAGGGGAATCAACTACCCATTGGGTACGGCGGGTATCAGAAGTTTTGCACTCGTCAGATCGCATCAACGCAGACACCGCCGTGGTGCTGTTGGAGAGCAACTGTCGGTTTGGCCCTTTGAAGCTAAAGCTGGGCCGGATGAAGCGTGATTGTACAGATATAGGAACGCTCATGACCGCTTTAGTGAAGTATGCGGATTCTGACAGTACCAAGGATCCTGACTCTGGTGATGACAAAGCAGGGAAGGGAAAGAAGAATAGCAACACCAAAGGTCAGCAGCATCGGCCGGCAGGTAATGGAGGCGGTGGAAAACGTAAACCGGACGGGAGCATGGACTTGGTGGCCAGCACAAGTGCACAGGATAAAGGCCAGCGGCGTAAGGGGAAACAGCCAAGTTTTCGCGCACATCCCGGTCCTAACCCGGAGCGTTTGAACTTTTTCTTAAACCAGCCCTGTCCGAAGCACGGAACAAAGGAGGAACCAGCAGCCCATCTTTGGAAGGATTGTTATATCATGAAGGAATTCAAAAGCTCAAACACTTTCCAATATGATCACAGCTCCGGCGGCAGTTCCGGTTCCGGATCAGGGTACGGTGGCGGGAGTTCCGGTCCAGGGTTCCAGGGTAACCCGAGCGGACAGGTCAGCCAAAATAATCAGAATAATCAAAGTAATCAAAGTGGTTATCAGAGCAACCCGAAACAGTTGAGTGGTGGACAGTATCATGTCTTTACTACGAGCTTGGATAAACGAGATAGGAAGCTTCAGAGGCGGGCAGTCAGTGCTGTCGAACCGGCCACGCCTCACTATCTGCGTTGGTCGGAACAGCCTATCATATGGAGCCGAGAAGATCATCCTCCCAGGGTGTATAATCCGGGTCAGTTAGCACTGGTGGTAGCGCCTCAGGTGGGAGGTTATAAGCTCACCAAGGTGCTCATGGACGGAGGGAGCAGCATTAACATCTTGTATTATGAGACCTTCCGTCGTATGGGACTGGTAGATAAGGATCTCAGACCGACCAATACAGTGTTCCACGGGGTGGTGCCTGGCAAGTCTGCATATCCGGTTGGTAAGATAGCCCTTGAAGTGGTATTTGGCGATAGTCACGATTCCAGATCAGAGACGCTGACGTTTGAGGTGGTTAAAATCCAAAGTCCGTACCACGCTTTGTTCGGGCGGCCAGCTTATGCAAAATTTATGGCACGGCCCTGCTACGTATATCTGCAACTTAAGATGCTAGGTTACAAGGGCACTATAACGGTTCATGGGAGCCGCCGAATCGCTTTGGAATGCGAGGAAGGAGATGCGACTTATGCTGAATCGGTTTGTGCTACGGAGGAGCTAAAGCAGTACAAAGACAGTGTTGATCCGGAGGATATGACTTCATTAAAAAAGCCAACTACGGACCACGATCCGGCCTTGAAGTTCAAGTCAGCGGCCGAAACTAAGCTTGTTGACTTCGTACCTGGCGATTCATCCAAGCAGTTCAGCATCAGTGCAAACTTGGATCCAAAATAGGAAAGCGCGCTCATCGAGTTCATCCGTGAGAATCGGGACATCTTTGCATGGAAGCCATCTGACATGCCTGGTGTACCGAGGCAACTCGCTGAGCACACCCTTAATGTGGATCCTAAGTACAAGCCGGTAAAGCAGTTCTTACGGCGGTTTAACGAAGAAAGACGTAAAGCGATTGGTGAGGAAGTGGCCAGGCTCCTGGCAGCTGGGTTTATCATTGAGGTTTTTCATCCCGAATGGCTCGCTAATCCGGTGCTAGTTCTTAAGAAAAACGGCACCTGGCGCATGTGTGTGGATTACACAGACTTGAATAAAGCTTGCCCAGCAGATCCTTTTGCTCTCCCCCGGATTGATCAGATCATTGATGCTACGGCAGGTTGCGAGCGTTTAAGTTTTTTGGATGCTTATTCTGGTTATCATCAGATCAAGATGGCAGTTAAGGACCAGGAGAAGACAGCGTTTATTACTCCCTTTGGAGCCTTCTGCTATGTGTCTATGCCCTTTGGGCTTAAGAGTGCGCAGGCTACTTATCAACGATGTGTGCAAAATTGCCTCCACGAGCAGATTGGTCGTAATGTGCATGCTTATGTGGATGATATTGTGGTCAAGTCACGAAAGAAGGAGACCCTGGTTGACGATTTGAAGGAGACTTTCGATAACTTGAGAGTGTACCAAATGATGCTCAACCCGGCTAAATGTGTTTTTGGTGTACCTGCAGGCAAGCTGTTGGGATTTTTAGTGTCCCATAGAGGAATTGAGGCCAATCCAGAAAAGATCACAGCCATCACCTCCCTGGCCAAACCGAAATGCATCAATGATGTTCAGCGTATGGCCGGGCGGATTGCAGCGTTAAGCCGGTTTATTAGTCGCCTTGGTGAAAAGGCGATCCCCTTGTATCAAATGCTCAAGAAGACGGATCAGTTTGTCTGGAGTCCGGAGGCTGATAAAGCGTTTGAGGACTTGAAGCGACAACTAGTCAATCCGCCAGTGTTGGCAGCCCCTGTAGATAAAGAGCCACTCCTGTTATATGTTGCAGCGAATGCCAGAGCAGTCAGTGTGGCGATGGTGGTAGAACGAAAGGAGGCTGGAAAGGAACATCCGGTTCAGCGACCGGTTTACTATATCAGTGAAGTACTTATCGAGTCCAAACAAAGGTATCCGCATTGGCAGAAGCTGGTATATGGCGTTTTTATGGCAAGCCGGAAGCTGAGGCAATATTTCCAAGGACACCCAATCACGGTGGTCAGTTCTGCTCCTTTGGGTGACATTATACAGAACCGGGAGGCGACCGGTCGGATTGCCAAGTGGGCTATCGAGCTCGGGCCGCACGATTTAAGGTACGTACCCCGGACAGCCATAAAATCACAGGCACTTGTCGATTTCATAAACGATTGGACAGAGTTACAAGCGCCTGAGGAGAAGCCAGATAACACCTATTGGACCGTTCATTTCGACGAGTCAAGACAATTGGAAGGCTCGGGGGCTGGAGTCGTTTTAACTTCCCCACGAGGAGATAAGTTTTGTTATGTCCTCCGTTTAATGTTCCCCTGTACAAACAATGCGGCTGAGTATGAAGCTTTGCTGCATGGACTTCGGGTGGCTAAAGAGATGAATTTGAGCAGAGTTAGGTGCTTTGGTGATTCGGATCTGGTGGCTCAGCAGGTATCTGGCACCTGGGATTCTAAGGATCCGCTTATGGCTGCATACCGACGTGAAGTAGATATGGTGGCTGGGCATTTCAAAGGTTATCAAGTGGATCATGTGGACCGCAGAAAGAATGAAGCAGCGGACGCTTTAAGTCGCTTGGGCTCTCAGCGTAAACCGGTCCCGCCCAATGTTTTCTTGGATGTATTGTACCACCCATCGGTACAGCTGCCCGGTGAGCTGGAGTTGGTTGTTCCTGATCCGGAGGCTCAGTTAGTGGCGGCTCTTCACGCCACCCCGAATTGGACGCTCCCTTACCTGGCATATATGAACCGGGGTGAGTTGCCGGAAGATGAAAGCTTGGCTCGACAGATAGTACGACGGTCCAAGTCTATGACCATTTTCCAAGGAGAGTTACATCACCGCAGCGTATCAGGAGCGCTGCAACGGTGCATATCCCCTAAGGAGGGGTGCGAGATATTACGAGAAATACATGAAGGGGATTGCGGCCATCACGCCGGTTCAAAATCATTGGTGGCTAAAGCGTTCCGCCATGGTTTCTACTGGTTAACTGCTCATGCTGATGCGGAAGACCTGGTCAGATTATGTGATGGTTGCCAGAAATTTTCCAGACGAGCACACATACCGGCTCAAGAATTGAGGATGATTCCAATCACTTGGCCGTTTGCGACTTGGGGGCTTGATATGGTTGGGCCTTTTAAGCGTTCCAAAGATAAGAAGACCCACCTACTGGTGGCGGTTGACAAATTTACAAAGTGGGTAGAGGCAGAACCTGTCAGTAAGTGTGATGCAGCCACGGCGGTTCGGTTTATAAAGAAGGTGATCTTCCGGTTTGGTTTTCCACACAGCATCATCACAGATAATGGTACCAATCTATCCAAGGGGGCCATGAAGGAGTTCTGTGCACGGGAGCATATACGGCTTGATGTTTCTTCGGTAGCGCACCCACAGTCTAATGGTCAGGCAGAACGAGCTAACCAAGAGATCTTGAGAGGTATCAAACCCCGGCTTCTGGTCCCTTTGCAAAGGACGCCGGGTTGTTGGGTGGAGGAGCTACCGTCTGTATTATGGAGCATCAACACCACGCCTAACAGATCCACGGGGTTTACGCCGTTTTTTATGGTTTATGGGGCAGAGGCGGTTCTCCCAAGTGATATACGACATGACTCGCCTCGCGTGGCAGCATATGTTGAAGCGGATAATGAGCAGGCACGGCAGAACGCTCTTGACTTGTTGGATGAGGAGCGTGACTTGGCAGCTGCCCGTTCAGCGATTTACCAGCAAGATCTTCGCCGTTACCACAGCCGTCGGGTTAGGACCAGAACCTTTCAGGAGGGGGATTTGGTGCTTCGGCTCATCCAGGATCAATCTGATCAGCATAAATTATCCCCGCCTTGGGAGGGACCTTTTGTGGTCAGCAAGAATCTGAATAATGGGTCATATTACCTGATTGATATTCGAGAGCGCAAGGATTCACGCACATCAGAGGAGGAAACCAGCAGGCCGTGGAACATAGCTCATCTTCGACCTTATTATACATGAGCCCTGGGCTCTGTTTATGTACATATCATGACCATGTATATATTATGATTAATACAATAAGCCGAAGCCTCGAGTAAAGCGGGGTCTCTTCTGTTCTTCACATCATGTGTGCTTACCCCTGGAGGTCGCTTCACAGAAGTTCAAATTATAGATTCCGGTTTAAAGGCCGATTCAAGGGAAGATGTCTCCTACAGGGCCGTGAAGCTCTTAATATCCGGTTCAATATCCCGGTTCACGCATGAGGCTTCCTGTTCAAACATAGGTCGTATTCGAACCAAAGAGAACATAGCTATTCAAACATGGGGCTTCCTGTTCAAACATAGGTCGGGTTCGAACCAAAGAGAACATAGCTAAACCTTATAAAGTCAGTTGGGGACCTAGTCGGCCTGAACCGTAGCTACGCCTCCGGGGGGCTTGGTCATGTCTTGACGACGGTAATGCCTTCTGGCTGGCACACTTGCCATTGAATCAGTTGGGGACCTAATCGACTTGAACCGTAGATTACGCCTTAGGGGAACTCGGTCATGTCTTGATGATGGTAATGCCTTCTGGTCGGCTTTTTAGCCAATGAATCATTTGGGGGCTTGGTCGTGTCCGAACCAACGCAATGCCACTTGATCGGCCCTTGGCCATTGAGTCACTTGGGGGCTTGGTTGACGAACCAATGCAATGCCATTTGGCCGGCATATTTGCCACGATTCAGTTAGGAACCTAGTCGACCTAAACCGTAGCTACACCTCTTGGGGGCTCGGTTTTGTCTTGACGATGGTCACGCCTTCTGAACGGTTCATAGTAACCATTATTCGCATGCGTTTTACTATCGCGTTGCCTTTTCATATTTGGTTTTTGTTTTCTGCCTTTATTGTGGTATTTTTTGTTCTTCGGAACGTCAAGTATCTTTAAACCGATTCAGCTGACATAGCCTGGTCCGTTTTTTAACCCAGAGGCAAGTTGCCCGGTTTGGCAAAGGCGCAGCACGGTTCATAGGGTTGGAAGATCTTGTGTACTCAATAAGGACAGGAAACAGAGTTGGAAAACGTCATTGGAACGTAGTGCCTGAATATATTGTTCGTAAAGGCGCAGGACGGTTCATAAAGAGCATCAGTACCAGTGCGCGAAGGCACATTCAAATGCAGAGGTATTAAACTATCTTATTACAAGACAACGTAGTGCCTGGATATATTGTTCGTTGACGGATTAAAACGTCACCAATTAAGGTGAACCGGAGCGTTGATTACGCCTGTTCGCCGCCTCGGTTCGAAGGCTGGGGATCGTCTTGTGCCGCTTCAGCTCTATCTTGGTTATCCATTGGCTGGAAATCAGCAGTTGTCCAGTTGATTCCCATTAGCGCTTGAAAGACGGCCTCTTCGTGAATCAGCAAAGACGGTTCAATGTCAGGGGCGTAAGTATGCTTACGGTTTGGAGGAATAAGGTTTCCCGCTTCAGGGACCGGGGCAGCCACTCGTTTGTTCTGGTTGTCGTAGTGGGCCTGGTAATGTGACAGATCTGCTTCCTCAGCCAATTGACAAGCTAGGGGGCGCACCGCCCGGTTTATCGCCCTCAAATCGTCCTCACCAAAGTCTGAACCGTCTTCCTTCAAGCTGGGGTATCCTTGGGCTGCTTCGACAGGATCGAAATCTGGTACCCACGCTTTGGCCCGGATCAAGGCAGTAATGGCTCCAGTTTGAGCAGCTGATCTCTTCAACTCTTCAATCCGGGCTGGAAGCATGGACAGCCTCTTTAACGTGTCCTGAATCAGAGACGGCGCAGGGTTGTGCGATGCAGTGGTGATAGCTCTTTGTGCTCCGGTGTACAACTGTTCAATCAGAGTGTAAGCAGCTTTGAGTTTCATCCGCACATCAGAACCCAAATGTCCAATACGTGAGCCTGCAGTGTTCCAAGATGGATTACAAACCGGACATAGGATGTAGCAGACAAGGTACAAATTGGAAGAAGGGCGGCGAAAAGCTTACCAAAGACGGCAGAGGTCATGGAGTGTATCTGTCGCTTCACGGTGGATAATTCATCAACCACCGGTTTAAGAGCAGCTTCGGCATTTTCAGCCCTCTTCGTCAAAAGAGCTCTCTCAGTAGCCCAATCAGCTTGTTCTTTTTTGTGGCTTTCTTTCAGCTGTTCCATAACGGTTAAGGCATGGGCCAATTCGTCCTTCGTTTTTTCAGTTTCAGCCTATTGGGTTTTTAAGATTTCCTGAAGGTCCGAGACTTGTTTCGCTTTGGTCTTCAGATCCCCCTGCAGCAGGTCGTCAACAGTGTTCAGAAAATATTGCACGGTTTGGAAGTATCAACCACACAAGCAAGTTATGCGCTCGATACTTGGGGGCTAATGCATACTTGATTTTAACGCAAGTTTTCCATTATAAAAAGTCCCAAGATTAATACAAGTATTTAAACTTGGCACTTGGGGGCTAATGGTTATCTGATGATGTTTTCTGATTATGACCTATGTTAAAGCCTTGAGTTTTCTAAACCGGACCTTAATTGTCAAGGCAGCCCGATCCGGTTTACCATCTCGTTTTGGAAATATTGATAAGTTAAGTTGCAATGAAATGAAGCATAAAGGAGTTACCTCAAGTTTCTCCTTCATCATCTTGATCATGCCAGCTTCATAATCCCGGCTGGTGTACAGTCGGTTCAGGTACCCAGAATAAAGATCCGGGGCGCTTAAAGCGGCATACGTAGAGGAGTCAAGGTCCCATTTGCCCTTTATGGCCGAGATTTCGTCCTTGGCAGTATGCTTGGCCAAAGTAGCAGTGTTCCCCGGTTCAGAGCGGCCAATGCCGGTAATCACAACATCATCATCCTTGGAATCGCTGCTCTGCAGTGGCCCTGTCGGTTTATCAACAGTTCCAGAAGGGGGGATGGAATCCTCAACCCGAACAGGACTGGCGGGCGGATCAGTACGGCTGGCATTTTCGATAGTCCTTTCTTCAGCGCAGGTGTCATCTTGCGGAGATAGATAATTCTGAGTATCTTCACGACCCAGAGTGTCAGCACCAGGAGTTTCTTCCGGTTCAGGAGCGGCAGTCCCTTCAGCCGGGTTATCCAGACGAGCCTTCTTGCTCGGTCTAGGTTTAGCCCTGAGAAAAAAAAGCCCAAAGATTGAAAAAACGGTATAAGATGGATTAAGAAACCCATACAGAGATATGTTGTGATCAGTCCAACTTACCCAATAACTGTCTTCAACGGTGGCAGTTGAGTGGCTGAGGATTCACCGGAAGAGGAATGGGAAGTGACCTGATAATCGGAGTCAGATGGATTTAGAGGCTGACGAGTTAAACCTGCTTTAGGAGGAAAGCTAGCGCATAAATCGGAGACCTCTGAGCGGCGTTTCCGAACCGGGCTGTTCGGTAAACCGGCGGAGGTAACTACCTGGCCGCTATGCCGGGTGGTGCGTCGGGCTTCATGTTGCTGGGTCTTCACAAGAAATTTGGGATCCAAGTAAGCAAGAGGGTGAGAAAATTTAATTTTCCGGTTTGCTTGGCGAGGGTTTTTCAAAGGCAAAGGAACGGCATCAGAAGAAAGAATAGTTACCTCTGGGCCTTCAGCATTGCTCGCTTCAGCGTCGTCCTGACAAATATCATCGTCAATAAGACGTACAAAAAGTAAGCCAACTGAATCAAGGTCTACCCCAAGGTCCGGATTACCCACATCATCCTCCTCTAAATCGGGGGTACAGGAAGCAGTGTGCCTTTTCTTTGTAGCAGGCTTCTTCACCCTTGTCTTGGGTCTGATTATCTTTTCGGTTGGCTCCTGAAGCTTCTTGCTCCAAAACGGATCATCTCCCTGTTTCGGAAAGGGCTGGAAGTTAGCAATGTATATAAGGAGATCAAAAATTCTATAAACAGGGGGAGTTACTTACAGCAGGTGGTTTGTTTGTGGCGCAAAATGGAAGCAGGCCGGTTCGAGCGCAAACATGTTCCGGTTCGTTCAGCATCTTTTTCACGGCTTCAGCGACTTCGTCCTCCGTCAGCTGAATGTTGCAGTGCCTCAAAGGATCATCAACCCGACCCGTGTATTGGCACATCAAACCGGAGCGGATGCTAAGAGGCAGGATGCTCCATGAAATCCAGCAACGAGCAAGATCAACCCCCGTTAAACCATTAGCCATGAAAGCTCTGAGCTTGGACAGCTGAGGGGCATACTTACTCCTCTCTTTGGCAGTTAGCCGTGGGGGGAAGGGGTGAGTGTTGCTAAGTCTCTCTGGACGGAAACCGGGCAGTGGATTTTCCTCAGCAGGGGAAGTATCCTTGCAATAGAACCAAGTGCAATTCCATTCTTGCGGATGACTATGCAACTTGGCGTGAGGATAAGTCACCTCTTTCCTTTTCTGAATCACCATACCACCCAGTTCCATATTAGGGCCGTCGATAAATTCAGTACGTCGGTTCAAATGGAAAAAGTCCCGGAACAGCTCTACAGTAGGTTCTTCTTGGAAGTACGCCTCGCAAAGTACTTGAAAATGGCAGATATTCGTGACTGAATTGGGACCAACATCTTGTGGGCGCAGCCTAAAATTAGCCAAGACGTCCCGATAAAATTTTGAACCGGGTGGATTAAACCCCCGGGCCAGGTGATCCGCAAACACCACCACTTCTCCCTCCTGCGGGGTAGGAGGACATTCGTCTCCTGGAACCCTCCAATGGATGGTACTTTTGCTACTCAAAGCACCAGTCAGAACTAAGTTGTTTAGTTGGGTCTCAGTGATGCAAGAGGGAACCCAGTTGCATTCATACACCTGTTTTGCCATGAGGAAATCTACAAGGCAAAGGTATTCCGGTTTGAGAGCAAGTATGATTGTAAACCGAAGGTGCTCTTATATAGAGTCAGGTTATCTACAATAATATCAACCGGCGGTTCATCAAGGGGACTAATGGATGTGGAGATCGGTTTGTTCGCTTTGCTAAGAGTTAAACCGACCAGATCTAAACAGTTGTGAGTATGAAAGCAGAAACAGATCCCGTACGGATTCTTTCAAAGCAATGGATCTACAGCTAGGGCGAAAAAGAAATAAAAACAGGTAATATGAAGTCGGGCTCACAGCAGAACAACATAAGTTCGACAAGCATGAACAAGAGTGAGATCTAAGGAAATGAGTACAGATTTATGTCGACTGCTAAGAGGAACTACAAATCCAGATACAGGTAGCATCATCTGTTCGCAAAGGCTGCTATGGGGATGAGCAAGAAACAGTAGCAGGACGATGAACCCTAGAACAGATCTAAAGCAACAAGAACAAGGGCATTACCGGGGTCCAAACAGATGCGGAAAGTCGCCGCGGTGCTCTGATCCGATCAGGTTGATGCAGCGGCCGGAGTTGGTGCAGCAGCTGACGGCGGCGGCGGAGCTCTGAGGTCTGGGTCGCGAGGAAGACGAAGAAGGGGAGGAAAGGAAGATGGCCTTCAAGTCCTATTTATAAGGCGCGGCGCGCGTGTCAGGCGCGCGAATCCAGGGGCCGGAGATTTCGGAAGCCCAGCCGTCGCCCTGATTCTTGCGGGTTAGCGAAAGGAGACGGTAGCTTGCGCTGACGTCATATCGGTTTACCACAGCAAGAGGAGACGGAGAAACAACGGTTTAACAAGTCACTGGAAGACATTTGAAGACAGAAATTCTTATTAAGGATTGACATGAACCTGTTCAAATCAATCTGGGGCCTAA

TGTTGGGGATGTTGCTATCCGGGGTAAACCGGCCTGCAGGGCCCGGGTTACCTCCGTCAACAGACCCGAGATGTTAAAACCCAAGGAGAGATAGAAGGGCCTAAGGCCCATAATCGGTTTAAAGGTTTATGGAGTAAACCGGCCTGATGTAAACTTGTATTGTAAGTTAGGAATAGAGAGACCGACCCGGGCACGTTTATTAGCCGGAGGTTGGGACTTTGCAGACCGACGGGCGTCACCCGTGTATATAAGGGGATGACCCGGCGGCGGTTCAGGGACAGAGAAGAAGAACTCGAGGTTTAGGCGAAGCATATCGCTCCCTAAGCCCTCGAAACCCATCAATCCCATCACAACTAGACGTAGGCTTTTACCTTCATCGAAGGGGCCGAACTAGTATAAAAACCGTGTCCTTGTGTCCACTTTAACCCCTTCAAGCTAACCCGTTGCGATGGCTCCACGACTAAGTCCTCTTACTAGGACATCTGCCGTGACAAATCCACGACA

CGCGTCTTCCCCGGCTCCGCGTCGTCCCCTTCCTAGGCCTCGCCGTCGTCCACCGCCTTGGTGCTCTCGGCGCGGCATGGTCAACGTGGTCAACGACATTCCATCGTAATACTAATGTACGTGG

>Aegilops umbellulata 1-315;

TTTTCATTAAAAACATTCCACTTAGGTACAGTCCATAGGGAATGAAAAACATGAATGATGGGATTACAAGTTCTAAATTCAGCTAAGGCCCGAAGGCTTACAAACTAAAAAGAGATAAGGTGCC

TGTCGTGGATTTGTCACGGCAGATGTCCTAGTAGAAGGACTTAGTCGTGGAGCCATCGCAACGGGTTAGCTTGAAGGGGTTACAGTGGACACAAGGACACGGTTTTTATACTAGTTCGGCCCCTTCGATGAAGGTAAAAGCCTACGTCTAGTTGTGATGGGATTGATGGGTTTCGAGGGCTTAGGGAGCGATAAGCTTCGCCTAAACCTCGAGTTCTTCTTTTTTGTCCTTAAACCGCCGTCGGGTCGTCCCCTTATATACACGGGTGACGCCCGTCGGTCTGTAAAGTTCCAACCTCCGGCTAAAACGTGCCCGGGTCGGTTTCTCTATTCCTAACTTACAATACAAGTTTACATCAGGCCGGTTTACACCATAAACCTTTAAATCGATTATGGGCCTTAGGCCCTTCTATCTCTCTTTGGGCTTTAACATCTCGGGTCTGTTGACGGAGGTAACCCGGGCCCTGCAGGCCGGTTTACCCCGGATAGTAACATCCCCAACA

TTAGGCCCCAGATTGATTTGAACAGGTTCATGTCAATCCTTAATAAGCACTTCTGTCTTCAAAATGTCTTCCAGTGACTTGTTAAACCGTCGTTTCTCCGTCTCCTTTTGCTGTGGTAAACCGATATAACGTCAGCGCAAGCTACCGTCTCCTTTCGCTAACCCGCAAGAATCAGGGCGACGGCTGGACTTCCGAAATCTCCGGCCCCTGGATTCGCGCGCCTGACACGCGCGCCGCGCCTTATAAATAGGACTTGAAGGCCATCTTCCTTTCCTCCCCTTCTTCGTCTTCCTCGCGACCTAGACCTCAAAGCTCCGCCGCCGCCGTCAGCTGCTGCATCAACTCCGGCCGCTGCATCAACCTGATCGGATCAGAGCACCGCGGCGACTTTCCACCTCTGTTTGGACCCCGGTAAGGCCCCTGTTCTTGTTGCTTTAGATCTTTTCTAGGGTTCATCGTTCTGCTACTGTTTCTTGCTCATCCCCATAGCAGCCCTTGCGAACAGATGTTGACACCTGTATTTGGATTTGTAGTTCCTTTTAGCAGTCGCCATAAATCCGCACTCATTTCCTTAAATCTCACTCTTGTTCCTGCTCTGTCGAACTTATGTTGTTCTGCTGTGAGCCCGACTTTATATTACCTGTTTTTATTTCTTTTTCGCCCTAGCTGTAGATCCATTACTTTGAAAGAATCCGTACGGGATCTGTTTCTGCTTCCATACTCACAACTGTTCAGATCTGGTCGGTTTAACTCTTAGCAAAGTGAACAAACCGATCTACACATCCATTAGTCCCCTTGATGAACCGCCAGTTGATATTATCGTAGATAACCTGACTCTATATAGGAGCATCTTCGGTTTATAATCATACTTGCTGTCAAACCGGAATACCTTTGCCTTGTAGATTTCCTCATGGCAAAGCAGGTGTATGAATGCAACTGGGTTCCCTCTTGCATCACTGAGACCCAACTAAACAACTTAGTTCTGACTGGTGCTTTGAGTAGCAAAAGTACCATCCATTGGAGGGTTCCAGGAGACGAATGTCCTCCTACCCCGCAGGAGGGAGAAGTAGTGGTGTTTGCGGATCACCTGGCCCGGGGGTTTAATCCACCCGGTTCCAAATTTTATCGGGACGTCTTGGCTAATTTTAGGCTGCGCCCACAAGATGTTGGTCCAAATTCAGTCACGAATATCTGCCATTTTCAAGTACTTTGCGAAGCGTACTTCCAAGAAGAACCTACTGCAGAGCTGTTCCGGGACTTTTTCCATTTGAACCGACGTACTGAATTTACCGACGGCCCTAATATGGAACTGGGTGGTATGGCGATTCAGAAGAGGAAAGAGGTGACTTATCCTTACGCCAAGTTGCATAGTCATCCGCAAGAATGGAACTGCACTTGGTTCTATTGCAAGGATACTTCCCCTGCTGAAGAAAATCCCCTGCCCGGTTTCCGTCCAGAGAGACTTAGCAACACTCACCCCTTCCCCCCACGGCTAACTGCCAAAGAGAGGAGTAAGTATGCCCCTCAGCTGTCCAAGCTCAGAGCCTTCATGGCTAATGGTTTAACGGGGGTTGATCTTGCTCGTTGCTGGATTTCATGGAGCATCCTGCCCCTTAGCATCCGCTCCGGTTTAATGTGCCAATACACGGGTCGGGTTGATGATCCTCTGAGGCACTGCAACATTCAGCTGACGGAGGATGAAGTCGCTGAAGCTGTGAAAAAGATGCTGAATGAACCGAAAAATGTTTGTGCCCAAACCGGCCTGCTTCCATTTTGTGCCACAAACAAGCCACCTGCTGTAAGTATCTCCCCTCTGTTTGTTATAGAACTTATGATCTCCTTATACACATATTGCTAATTCCAGTCCTTCCTGAGACAGGGAGATGACCCATTTTGGAGCAAGAAGTTACAAGAGCCGACCGAAAAGGTAACCCGACCCAAGACGAGGGTGAAGAAGCCCGCTCAGAGGAAAAGGAACACCGCTTCCTCTACTCCGGAATTAGAGGAGGAAGATGTGGGTAATCCGGACCTCGGGGTAGACCTCGATTCAGTTGGTTTGCTTTTTATACGTCTTATTGATGATGATATTTGTCAGGACGACGCTGAAGCGAGCCATGCCGAAGGCCCAGAGGTAACTATTCTTTCTTCTGATGCCGTTCCTTTGCTTTTGAAAAAACCTCGCCAGGCAAACCGGAAAATTAAATTTTCTCACCCTCTTGCTTACTTGGATCCCAAATTTCTTGTGAAGACCCAGCAACATGAAGCTCGACGCACCACCCGTCATAGCGGCCAGGTAGTCACCTCCGCCGGTTTACCGAACAGCCCGGTTCGGAAACGCCGTTCAGAGGTCTCCGATTTACGCGCTAGCTTTCCTCCTAAAGCAGGTTTAACTCGTCAGCCTCTAAATCCATCTGACTCCGATTATCAGGTCACTTCCCATTCATCTTCCGGTGAATCTTCAGCCACTCAACTGCCACCGTTGAAGACAGTTATTGGGTAAGTTGGACCGATCACAACATATCTCTGTATGGGTCTCTTAGTCCATCTTATATTGTTTTACCAATCTTTGGGTTTTTCCAGGGCTAAACCTAGACCGAGCAAGAAGGCTCGTCTGGATAACCCGGCTGAAGGAACTGCCGCTCCTGAACCGGAGAAAACTCCTGGTGCTGACGCTCTGGGTCGTGAAGATACTCAGAACTATCTATCTCCGCAAGATGACATCTGCGCTGAAGAAAGGACTACCGAACCTGCTAGCCGTACTGATCCGCTTGCCAGTCCTGTTCGGGTTGAGGAATCCATCCCCCCTTCTGCAACTGCTGATAAACCGACAGGGCCACTGCAGAGCAGCGACTCCAAGGATGATGATGTCGTGATTACCGGTATCGGCCGCTCTGAACCAGGGAACACTGCTACTTTGGCCAAGCATACTGCCAAGGACGAAGTCTCGGCCATAAAGGGCAAATGGGACCTTGACTTATCTACGTACGCCTCTTTAAGCGCCCCGGATCTTTATTCTGGGTATCTGAACCGGCTGTACACCAGTCGGGATTATGAGGCTGCCATGATCAAGATGATGAAGGAGAAACTTGAGGTAACTCCCTTGTGCTTCATTCTATTGCAACTTAGCTTATCAATATTTCCAAACCGAGATGGTAAACCGGACCAGGCTGCTTTTGACAATTAAGGTCTGGTTTAGAAAACCCAAGGCTTCAACATCGGTCATAACCAGAAAACATCATCAGATAACCATTAGCCCCCAAGTGCCAAGCTTAAATACTTGTATTAATCTTGGGACTTTTTGTAACGGAAAATTTGTGTTAAAATCAAACATGCATTAGCCCCCAAGTATCGAGCACATAACTTGCTTGTGTGGTTGATACTTCCAAACCGTGCGATATTTTCTGAACACTGTTGATGACCTGCCGCAGGGGGATTTGAAGACCAAAGAGAGCCAAGTCTCGGACCTTCAGGAAGCTTTAAAAGCCCAGCAGATTGAAACTGAAAAAGCAAAGGAGGAATTGACCCAAGCCTTAACTGTTATGGAGCAGCTGAAAGCAAGCCGCAAAAAGGAGCAAGCCGATTGGGCTACTGAAAGAGCTCTTTTGACAAAGAGGGCTGAAAATGCCGAAGCTGCTCTTAAACCGGTGGTTGATGAGCTATCCACCGTGAAGCGACAGATACACTCCATGACATCTGCCGTCTTTGGTAAACCTCTCAATGCCCTTTTTCCAATTTGTACCTTGTTTGCTACACCCTACGTCCGGTTTGTAATCCACCTTGAAACACTGCAGGCTCACGTATTGGACACTTGGGTTCTGATGTGCGGATGAAACTCAAAGCTGCTTATACCCTGATTGAACAGTTATACACCGGAGCACAAAGAGCTATCACCACCGCATCGCACAACAACCCTGCGCCGTCTCTGATTCAAGACACATTAAAGAGGCTGTCCATGCTTCCGGCCCGGATTGAAGAATTGAAAAGATCAGCTGCTCGAACTGGAGCCATCACTGCCTTGATCCGGGCCAAAGCGTGGGTACCAGATTTCGATCCTGTCGAAGCGGCCCAAGGATACCCCAGCTTGAAGGAAGACGGTTCAGACTTTGGTGAAGACGATTTGAGGGCGATAAACCGGGCGGTGCGCCCCCTAGCTTGTCAATTGGCTGAGGAAGCAGATCTGTCACGTTACCAAGCCCAATACGACAACCAGAACAAACGAGTATCTGCCCCGGTCCCTGAAGCGGGAAACCTTGTTCCTCCAATCCGTAAGCATACTTACGCCTCAGACATTGAACCGTCTTCGCTGATTCACGAAGAAGCCGTCTTTCAAGCGCTAATGGGAATCGACTGGACCACTGCTGATTTCCAGCCGATGGGTAACCAAGATGGAGCTGAAGCGGCGCGAGACGATCCCCAGCCTTCGAACCGAGGCGGCGAACAGGCGTAATCAACGCTCCGGTTCACCTTACTTGGTGACGTCTTAATCCGTCAATGAACAATATATTCAGGCACCACGTTGCCTTGTAATAAGATAGTTTAATACCTCTACATTTGAATGTGCCTTCGCGCACGGGTACTGATGCTCTTTCCAATGACGTTTCCAACTTATGTTTCCTGTCTTTACTGAGCACACAATGTGATCTAAACCGGGCAATTTGCCTCCGGGTTCAAAGACGGACCAGACTACGCCAGCTGAATCGGTTTAAGGATACTTGACGTTCCGAAGAACCAATAAACACCACAATAAAGGCAGAAAACAAAACCAAATATGAAAAGGCAACGCGATAGTAAAATGCATGCGAATAGTGGTTACGATGAACCGTTCAGAAGGTGTGACCATCGTCAAGACAAAACCGAGCCCCCAAGAGGTGTAGCTACGGTTTAGATCGACTAGGTTCCTAACTGAATCGTGGCAAATTTGCCGGCCAAATGGCATTGCATTGGTTCGTCAACCAAGCCCCCAAGTGACTCAATGGCCAAGGGCCGATCAAGTGGCATTGCGTTGGTTCGGACACGACCAAGCCCCCAAATGATTCATTGGCTAAAAAGCCGACCAGAAGGCATTACCATCATCAAGACATGACCGAGTACCCATAAGGCGTAATCCACGGTTCAAGTCGATTAGGTCCCCAACTGATTCAGTGGCAAATGTGCCAGCCAGAAGGCATTACCATCGTCAAGACATGACCAAGCCCCCTGAAGGTGTAGCTACGGTTCAGGCCGACTAGGTCCCCAACTGACTTCATAGGGATAGCTACGTTCTCTTTGGTTCGAATACGACCTATGTTTGAACAGCTGTGTTCTCTTTGGTTCGAATACGACCTATGTTTGAACAGGAACCCCCATGTTTGAATAGCTATGTTCTCTTTGGTTCGAATACGACCTATGTTTGAACAGGAACCCCCATGTTTGAATAGCTATGTTCTCTTTGGTTCGAATACGACCTATGTTTGAACAGGAAGCCTCATGTTTGAATAGCTATGTTCTCTTTGGTTCGAATACGACCTATGTTTGAACAGGAAGCCTCATGCTTGAACCGGGATATTGAACCGGATATTAAGAGCTACACAGCCTTGCCGGAGACATCTTCCCTTGAACCGGCTTTTAAACCGGAATCTATATCTTGAATTTTGTGAAGCGACCTCCAGGGGCAAACACACATGATGTGAAGAACAGCAGAGACCCCGCTTTACTCGAGGCTCCGGTTTATTGTATTAATCATAATGTATACATGGTCATGACATGTACATAAGCAGAGCCCAGGGATCATGTATAATAAGGTCGTAAATGAGCTATGTTCCACGGCCTGCTGGTCTCCTCCTCTGATGTGCGTGAATCCTTGCGCTCTCGAATATCAATCAGGTAATATGACCCATTATTCAGATTCTTGCTGACCACAAAAGGTCCTTCCCAAGGCGGGGATAATTTATGCTGATCAGACTGATCCTGTATGAGCCGAAGCACCAAATCCCCCTCCTGAAAGGTTCTGGTCCTAACCCGACGGCTGTGGTAACGGCGAAGATCTTGCTGGTAAATCGCTGAACGGGCAGCTGCCAAGTCACGCTCCTCATCCAACAAGTCAAGAGCATTCTGCCGTGCCTGCTCATTATCCGCTTCAACATAGGCTGCCATGCGAGGTGAGTCATGTCGCATATCACTTGGGAGAACCGCCTCTGCTCCATAAACCATAAAAAACGGCGTAAACCCCGTGGATCTGTTAGGCGTGGTGTTGATGCTCCATAATACAGACGGTAGCTCCTCCACCCAACAACCCGGCGTCCTTTGCAAAGGGACCAGAAGCCGGGGTTTGATACCTCTCAAGATCTCTTGGTTCGCTCGTTCTGCCTGACCATTAGACTGTGGGTGCGCTACCGAAGAAACATCAAGCCGTATATGCTCCCGTGCACAGAACTCCTTCATGGCCCCCTTGGATAGATTGGTACCATTATCTGTGATGATGCTGTGTGGAAAACCAAACCGGAAGATCACCTTCTTTATAAACCGAACCGCCGTGGCTGCATCACATTTACTGACAGGTTCTGCCTCTACCCACTTTGTAAATTTATCAACCGCCACCAGTAGATGGGTCTTCTTATCTTTGGAACGCTTAAAAGGCCCCACCATATCAAGCCCCCAAGTCGCAAACGGCCAAGTGATTGGAATCATCCTCAATTCTTGAGCCGGTATGTGCGCTCGTCTGGAAAATTTCTGGCAACCATCACATAACCGGACCAGGTCTTCCGCATCAGCATGAGCAGTTAACCAGTAGAAACCGTGGCGGAACGCTTTGGCCACCAATGATTTTGAACCGGCGTGGTGGCCGCAATCCCCTTCATGTATTTCTCGTAGTATCTCGCACCCCTCCTTAGGGGATATGCACCGTTGCAGTGCTCCTGACACGCTGCGATGATGTAACTCTCCTTGGAAAATGGTCATAGACTTGGACCGTCGCACTATCTGTCGAGCCAAGCTTTCATCCTCTGGCAACTCACCCCGGTTCATATATGCCAGGTAAGGGAGCGTCCAATCCGGGGTGGCGTGAAGAGCTGCCACTAACTGAGCCTCCGGATCAGGAACAGCCAACTCCAGCTCACCGGGCAGCTGTACCGATGGGTGGTACAATACATCCAAGAAAACATTGGGTGGGACCGGTTTACGCTGAGAGCCCAAGCGACTTAAAGCGTCCGCTGCCTCATTCTTTCTGCGGTCCACATGATCCACTTGATAACCTTTGAAGTGTCCAGCCACCATATCCACTTCACGTCGGTATGCAGCCATAAGCGGATCTTTAGAATCCCAGGTGCCAGATACCTGCTGAGCCACCAGATCCGAATCTCCAAAGCACCTAACTCTGCTCAGATTCATCTCTTTAGCCACCCGAAGACCATGAAGTAAAGCCTCATACTCAGCCGCATTATTTGTACAGGGGAACATTAAACGGAGGACATAACAAAACTTATCTCCTCGTGGGGAAGTTAAAACAACTCCAGCCCCCGAGCCTTCCAACTGTCTTGACCCGTCGAAATGAACGGTCCAATAGGTGTTATCTGGCTTCTCCTCAGGCGCTTGTAACTCTGTCCAATCGTTTATGAAATCGACAAGTGCCTGTGACTTTATGGCCGTCCGGGGTACGTACCTTAAATCGTGCGGCCCGAGCTCGATAGCCCACTTGGCAATCCGACCGGTCGCCTCCCGGTTCTGTATAATGTCACCCAAAGGAGCAGAACTGACCACCGTGATTGGGTGTCCTTGGAAATATTGCCTCATCTTCCGGCTTGCCATAAAAACGCCATATACCAGTTTCTGCCAATGCGGATACCTTTGTTTGGACTCGATAAGTACTTCACTGATATAGTAAACCGGTCGCTGAACCGGATGTTCCTTTCCAGCCTCTTTTCGTTCTACCACCATCGCCACACTGACTGCTCTGGCATTCGCTGCAACATATAACAGGAGTGGCTCTTTATCTACAGGGGCTGCCAACACTGGCGGATTGACTAGTTGTCGCTTCAAGTCCTCAAACGCCTTATCAGCCTCCGGACTCCAGACAAACTGATCCGTCTTTCTGAGCATTTGATACAAGGGGATCGCGTTTTCACCAAGGCGACTGATAAACCGGCTTAACGCCGCAATCCGCCCGGCCATGCGCTGAACATCATTGATGCATTTCGGTTTGTCCAGGGAGGTGATGACTGTGATCTTTTCTGGATTGGCCTCAATTCCTCTATGGGACACTAAAAATCCCAACAGCTTGCCTGCAGGTACACCAAAAACACACTTAGCCGGGTTGAGCATCATTTGGTACACTCTCAAGTTATCGAAAGTCTCCTTCAAATCGTCAACCAGGGTCTCCTTCTTTCGTGATTTGACCACGATATCATCCACATAAGCATGCACATTACGCCCAATCTGCTCGTGGAGGCAATTTTGCACACATCGTTGATAAGTAGCCTGCGCACTCTTAAGCCCAAAGGGCATAGACACATAGCAGAAGGCTCCAAAGGGAGTAATGAACGCTGTCTTCTCCTGGTCCTTAACTGCCATCTTGATCTGATGATAACCCGAATAAGCATCCAAAAAACTTAAACGCTCGCAACCTGCCGTAGCATCAATGATCTGATCAATCCGGGGGAGAGCAAAAGGATCTGCTGGGCAAGCTTTATTCAAGTCTGTGTAGTCCACGCACATGCGCCAGGTGCCGTTTTTCTTAAGAACTAACACCGGATTAGCGAGCCATTCGGGATGAAAAACCTCAATGATAAACCCAGCTGCCAGGAGCCTGGCCACTTCCTCACCAATCGCTTTACGTCTTTCTTCGTTAAACCGCCGTAAGAACTGCTTTACCGGCTTGTACTTAGGATCCACATTAAGGGTGTGCTCAGCGAGTTGCCTCGGTACACCAGGCATGTCAGATGGCTTCCATGCAAAGATGTCCCGATTCTCACGGATGAACTCGATGAGCGCGCTTTCCTATTTTGGATCCAAGTTTGCACTGATGCTGAATTGCTTGGATGAATCGCCAGGTACGAAGTCAACAAGCTTAGTTTCAGCTGCTGACTTGAACTTCAAGGCCGGATCGTGGTCCGTAGTTGGCTTTTTTAATGAAGTCATATCCTCCGGATCAACACTGTCTTTGTACTGCTTCAGCTCCTCCGTAGCACAAACCGATTCTGCATAAGTCGCATCTCCTTCCTCGCATTCCAAAGCGATTCGACGGCTCCCATGAACCATTATAGTGCCCTTGTAACCTGGCATCTTAAGTTGCAGATACACGTAGCAGGGCCGTGCCATAAATTTTGCATAAGCTGGCCGTCCAAACAAAGCGTGGTACGGGCTTTGGATTTTAACCACTTCGAACGTCAGCGTCTCTGATCTGGAATCGTGACTATCGCCAAACACCACTTCAAGGGCTATCTTACCAACCGGATATGCAGACTTGCCAGGCACCACCCCGTGGAACACTGTATTGGTCGGTTTGAGCTCCTTATCTACCAGTCCCATACGACAGAAGGTCTCGTAATATAAGATGTTAATGCTGCTCCCTCCGTCCATGAGCACCTTGGTGAGCTTATAACCTCCCACTTGAGGCGCCACCACCAACGCTAGCTGACCCGGATTATAAACCCTGGGAGGGTGATCCTCTCGGCTCCATATGATAGGCTGTTCTGACCAGCGCAGATAGTGAGGTGTGGCCGGTTCTACAGCACTGACTGCTCGTCTCTGAAGCTTTCTATCCCGTTTATCCAAGCTTGTAGTAAAGACATGATATTGTCCACCACTCAACTGTTTCGGGTTGCTCTGATAACCTGTCTGCTGCTGCCCGAAACCACTCTGATTACTTTGATTATTTTGGCTGACCTGTCCGCCCGGATTGCCCTGGAACCCTGGACCGGAACCTGAACCGCCGCCGGAGCTGCGATCATTCTGGAAAGTGTTTGAGCTTTTGAACTCCTTCATGATATAACAATCCTTCCAAAGATGGGTTGCTGGTTCCTCCTTTGTTCCGTGCTTCGGACAGGGCTGGTTTAGGAAAAAGTTCAAACGCTCCGGATTAGGACCAGGATCTGTGCGGAAACTTGGCTGTTTTGCCTTGCGCCGCTGGCCCTTATTCTGCGCACTTGTGTTGGCTACAAAGTCCATGCTCCCGTCCGGTTTACGCTTACCTCCACCTCCATTACCTGTCGGTCGATGCTGCTGGCCTTTGATGTTGCTATTCTTTCTTCCCTTCCCTGCTTTGTCATCACCAGAGTCAGGATCCTTGGTACTGTCAGAATCCGCATACTTCACTAAAGCGGTCATGAGCGTTCCTATATCTGTACAATCACGCTTCATCTGGCCCAGCTTTAGCTTTAAAGGGCCAAACCGACAGTTGCTTTCCAACAGCACTACAGCGGTGTCTGCGTTGATGCGATCTGATGAGTGCAAGACTTCTGATACCCGTCGTACCCAATGGGTAGTTGATTCCCCTTCTTGCTGAACGCAGGCTGCTAGGTCAACTATTGACATTGGCTGTTTGCATGTGTCCTTGAAGTTCTTGATAAACCGGTCGCGCAATTGGGCCCATGAGCTAATTGAATTAGGCGGCAAACTCTTTAGCCAAGTCCGGGCCGTTCCTTCTAACATCATAGTAAAATACTTGGCACATGCCGCGTCATCCACATCCAGCATCTCCATAGCCATTTCATAGCTCTCCACCCACGTCTCCGGAGGCTGATCTGCCGTGTAGTTTGGTACTTTGCGGGGGCCTTTGAAGTCTTTGGGCAGGCGTACGTTGCGCAAAGCGGGAACAAGGCACGGTACTCCCAAAGAACTGGAGACCACCCCCGGTTCAGCCGCAGCTGATGGGAAGACAGGTGTAATCTGATGAGCCTAATGCTGTGCTGCCAATCCGGCCTCCCGTGCACGGGTGCGATCCACATACTCGTGAGCATTGTCAACGCCCGCCGGATTGTTGCCGCGGAGCGCTTCACGCCTGGCATTACTTGAAACGGCCGGCTCCTCCACGCGCCTACTATAGCTCCCGCTGGGGCGAGGGGTGGAGTGAATCCGATCACGGCTTTGCGAATATGCTTCTTGCTGGGCCAGCGCGGTCCTGAGGAGCTCCTTGACCCGGCGTGTCTCTTCATCCTGCGGTGAATCTCCTTCGATCGGAATGGCTTCCAACCGAGACGCAGCCGCGACGAGGTTATCCATCGGGTTGGAATAATGACCTGACGGCGTGGGTATGGCCGGTGGTGTACCAGTGTTGTAACGAGGCAGGTCTATCACCCGGTGCTGAACCGGGGCGCCGGTTGTCGCCTCCGCACGGTTTACCCCCGGGGTGTTGAAGAGATTTCGAGCGTCGAGGGCGGCGGGCAACCGAGACCGGGATTTTCTCTTCAGGACCTCCTGAGACGCCTTCTGGTCCATCATGAGCCTGTACGCCTGTGCATCTAAAGCGGCGCGCTCCGCCGTCATCCTGATCTCTTCTGCTGCCAGCTCCGCTTTAGCCCGAGTGATCTGCTCTCTCACCTTGGTAATCTCAGCATTGTGAACTTCCTGATCCACCGGGTTGGCTTCCGCCATGAGCGCTGCTAGCGCGTCAAACAGGTCTGACAAAACTTGGGCTGGTGAACCCGTAGAGCCTCCTACCCGCGCAGCCGTTGCCGCTGCTAATCCGGAGATCGCTGCTGCTGCAGTTGATGATCGTGGCATCGCCTGCGTTCCAGCCATGAAGATCCCAACCCGATAGGGCAGATCAGAGGGGTCCGGAATACTGTTGCCATCGGAGCCGCCTCCAATCCGATCATCTTGTAGCTGGTAAAGAGATTCGGTCTCCCCGGTCGACGTTTCATCGCCAGAGTAGATGGCGGTTTCGTCACCAGATTCGGATCCTTCCTCGTAGCTTCCCCCGTGGATGACTCCCACGAAGGCGTGCTTCCTGGTCGGTTTAGCCAGGGTCGATCTCGTGCACTGAGCTGTCTCGATGAGGTCGGCGCAGACGCCTAACTCGGGGCCCGGTTCTCCGATCTTGCCGATGAAAACATGGATGCCGCCAAAGGGGACCCGGTACCCGTACTCGATCGAGCCGGCGTCGGGGCCCCAGCCTGTGCTGTCGATGTAGAGTTTTCTGCGGCGGCTCTTGGTCATCCGTCCCACAGCGTAACCCTCGAGTCCTTCAAAGCGGCCCTCCAAGAACCTGAAACCATCTCGCGATAGCCCCACGGTGGGCGCCAAC

TGTCGTGGATTTGTCACGGCAGATGTCCTAGTAGAAGGACTTAGTCGTGGAGCCATCGCAACGGGTTAGCTTGAAGGGGTTACAGTGGACACAAGGACACGGTTTTTATACTAGTTCGGCCCCTTCGATGAAGGTAAAAGCCTACGTCTAGTTGTGATGGGATTGATGGGTTTCGAGGGCTTAGGGAGCGATAAGCTTCGCCTAAACCTCGAGTTCTTCTTTTTTGTCCTTAAACCGCCGTCGGGTCGTCCCCTTATATACACGGGTGACGCCCGTCGGTCTGTAAAGTTCCAACCTCCGGCTAAAACGTGCCCGGGTCGGTTTCTCTATTCCTAACTTACAATACAAGTTTACATCAGGCCGGTTTACACCATAAACCTTTAAATCGATTATGGGCCTTAGGCCCTTCTATCTCTCTTTGGGCTTTAACATCTCGGGTCTGTTGACGGAGGTAACCCGGGCCCTGCAGGCCGGTTTACCCCGGATAGTAACATCCCCAACA

GTGCCCGTAATCCCTTTCTTGTCTCTCTCCTCAGGAGGCAGGTCAAGGAGGCGAAAAGGGCAAAAGGGGCGCCTAGGCGAGCTACGGGTGGCAGAAGACACCAAGAGAACATCTCGGTGGCCGT

>Aegilops umbellulata 1-321;

GGTTGCACAACCGCTTGACTCAGCTGGAAGTTAATCTCCCGGATGAGGCGGTCATTGACAGAATCCTTCAGTCGCTCCCACCTAGCTACAAGAGCTTCGTGAGAACTACAATATGCAGGGGATG

TGTCGTGGATTTGTCACGGCAGATGTCCTAGTAAGAGGACTTAGTCGTGGAGCCATCGCAACGGGTTAGCTTGAAGGGGTTAAAGTGGACACAAGGACACGGTTTTATACTAGTTCGGCCCCTTCAATGAAGGTAAAAGCCTACGTCTAGTTGTGATGGGATTGATGGGTTTCGAGGGCTTAGGGAGCGATAAGCTTCGCCTAAACCTCGAGTTCTTTTTTCTCTGTCCCCAAACCGCCGTCGGGTCGTCCCCTTATATACACGGGTGACGCCCGTCGGTCTGCAAAGTTCCAACCTCCGGCTAATAAACGTGCCCGGGTCGGTCTCTCTATTCCTAACTTACAATACAAGTTTACATCAGGCCGGTTTACACCATAAACCTTTAAACCGATTACGGGCCTTAGGCCCTTCTATCTCTCCTTGGGTTTTAACATCTCGGGTCTGCTGACGGAGGTAACCCGGGCCCTGCAGGCCGGTTTACCCCGGATAGCAACATCCCCAACA

TTAGGCCCCAGATTGATTTGAACAGGTTCATGTCAATCCTTAATAAGAATTTCTGTCTTCAAATGTCTTCCAGTGACTTGTTAAACCGTCGTTTCTCCGTCTCCTCTTGCTGTGGTAAACCGATATGACGTCAGCGCAAGCTACCGTCTCCTTTCGCTAACCCGCAAGAATCAGGGCGACGGCTGGGCTTCCGAAGTCTCCGGCTCCTGGATTCGCGCGCCTGACACGCGCGCCGCGCCTTATAAATAAGACTTGAAGGCCATCTTCCTTTCCTCCCCTTCTTCGTCTTCCTCGCGACCCAGACCTCAAAGCTCCGCCGCCGCCGTCAGCTGCTGCACCAACTCCGGCCGCCGCATCAACCTGATCGGTTCAGAGCACCGCGGCGACTTTCCGCATCTGTTTGGACCCCGGTAAGGCCCCTGTTCTTGTTGCTTTAGATCTGTTCTAGGGTTCATCGTTCTGCTACTGTTTCTTGCTCATCCCCATAGCAGCCTTTGCGAACAGATGATGCTACCTGTATCTGGATTTGTAGTTCCTCTTAGCAGTCGCCATAAATCTACACTCATTTCCTTAGATCTCGCTCTTGTTCCTGCTTGTCGAACTTATGTTGTTCTGCCGTGAGCCCGACTTCATATTACCTGTTTTTTATTTCTTTTTCGCCTTAGCTGTAGATCCATTGCTATGAAAGAATCTGTACGGGATCTGTTTCTGCTTTCATACTCACAACTGTTTAGATCTGGTCGGTTTAACTCTTAGCAAAGCGAACAAACCGATCTACACATCCATTAGTCCCCTTGATGAACCGCCGGTTGATATTATTGTAGATAACCTGACTCTATATAAGAGCATCTTCGGTTTACAATCATACTTGCTCTCAAACCGGAATACCTTTGCCTTGTAGATTTCCTCATGGCAAAACAGGTGTATGAATGCAACTGGGTTCCCTCTTGCATCACTGAGACCCAACTAAACAACTTAGTTCTGACTGGTGCTTTGAGTAGCAAAAGTACCATCCATTGGAGGGTTCCAGGAGACGAATGTCCTCCTACCCCGCAGGAAGGAGAAGTGGTGGTGTTTGCGGATCACCTGGCCCGGGGGTTTAATCCACCCGGTTCAAAATTTTATCGGGACGTCTTGGCTAATTTTAGACTGCGCCCACAAGATGTTGGTCCAAATTCAGTCACGAATATCTGCCATTTCCAAGTACTTTGCGAGGCGTACTTTCAAGAGGAACCCACTGTAGAGCTGTTCCGGGACTTTTTCCATCTGAACCGACGTACTGAATTCACCGATGGCCCTAATATGGAACTGGGTGGTATGGCGATTCAGAAGAGGAAAGAGGTGACTTATCCTCACGCCAAGTTGCATAGTCATCCGCAAGAATGGAATTGCACTTGGTTCTATTGCAAGGATACTTCCCCTGCTGAAGAAAATCCACTGCCCGGTTTCCGTCCAGAGAGACTTAGCAATACTCACCCCTTCCCCCCACGGCTAACTGCCAAAGAGAGGAGTAAGTACGCCCCTCAGATGTCCAAGCTCAGAGCCTTCATGGCTAACGGTTTAACGGGGGTAGATCTTGCTCGTTGCTGGATTTCGTGGAGCATCCTGCCCCTTAGCATCCGTTCCGGTTTAATGTGCCAATACACGGGTCGGGTTGATGATCCCTTGAGGCACTGCAACATTCAGCTGACGGAGGACGAAGTCGCTGAAGCTGTGAAAAAGATGCTGAATGAACCGGAACATGTCTGTGCCCAAACCGGCCTGCTTCCATTCTGTACCACCAGCAAGCCTCCTGCTGTAAGTATCTCCCCTCTGTTTGTTATAGAACTTATGATCCCCTTATACACATATTGCTAACTCCAGTCCTTCCTGAGACAGGGAGATGATCCGTTTTGGAGCAAGAAGTTACAAGAGCCAACCGAAAAGGTAACCCGACCCAAGACGAGGGTGAAGAAGCCTGCTCAGAGGAAAAGGAACACCGCTTCCTCTACTCCGGATTTAGAGGAGGATGATGTGGGTAATCCGGACCTCGGGGTAGACCTCGATTCAGTTGGTTTGCTTTTTATACGTCTTATTGATGATGATATTTGTCAGGACGACGCTGAAGTGAGCCATGCCGAAGGCCCAGAGGTAACTATTATTTCTTCTGATGCCGTTCCTTTGCCTTTGAAAAAACCTCGCCAAGCAAACCGGAGAATTAAATTTTCTCACCCTCTTGCTTACTTGGATCCCAAATTTCTTGTGAAGACCCAGCAACATGAAGCTCGACGCACCACCCGACATAGCGGCCAGGTAGTCACCTCCGCCGGTTTACCGAACAGCCCGGTTCGGAAACGCCGCTCAGAGGTCTCCGATTCACGCGCTAGCTTTCCTCCTAAAGCAGGTTTAACTCGTCAGCCTCTAAATCCATCTGACCCCGATTATCAGGTCACTTCCCATTCCTCTTCCGGTGAATCTTCAGCCACTCAACTGCCACCGTTGAAGACAGTTATTGGGTAAGTTGGACCGATCACAACATATCTCTGTATGGGTTTCTTAATCCATCTTATACCGTTTTTTCAATCTTTGGGCTTTTTCCAGGGCCAAACCTAGACCGAGCAAGAAGGATCGTCTGGATAACCCGGCTGGAGGAACTGCCGCTCCTGAACCGGAGAAAACTCCTGGTGCTAACGCTCCGGGCCGTGAGGACACTCAGAGCTATCTATCTCCGCAAGATGACACCTGCGCTGAAGAAAGGATTATCGAACCTGCCAGCCATACTGATCCGCCTGCCAGTCCTGTTCGGGTTGAGGAATCCATCCCCCCTTCTGGAACTGCTGATAAACCGACAGGGCCACTGCAGAGCAGCGATTCCAAGGACGATGATGTTGTGATTACCGGCATCGGTCACTCTGAACCGGGGAATACTGCTACCTTAGCCAAGCATACTGCCAAGGAAGAAATCTCGGCCATGAAGGGCAAATGGGACCTTGACTCTTCTACATACGCTGCTTTAAGCGCCCCGGATCTTTATTCCGGGTACCTGAACCGATTGTACACCAGTCGGGATTATGAAGCTGGCATGATCAAGATGATGAAGGAGAAACTTGAGGTAACTCCTTGTGCTTTATTCCATTGCAACTTAGCTTATCAATACTTCCAAACCGAGACGGTAAACCCGGATCAGGCTGTCTTGACAATTAAAGTCCGGTTTAGAAAACCCAAAGCTTCAACTTAGGTCATAACCAGAAAACATCATTAAATAACTATTAGCCCCCAAGTGCCAAGTTTAAATACTTGTATTAATCTTGGGACTTTTTGCAATGGAAAACTTGCGTTAAAATCAAATATGCATTAGCCCCCAAGTATCGAGCACATAACTTGCTTGTGTGGTTGATACTTCCAAACCGTGCAATATTTTCTGAACACTGTTGATGACCTGCTGCAGGGGGATTTGAAGACCAAAGAGAACCAAGTCTCGGACCTTCAGGAAGCTTTAAAAGCCCAGCAGATTGAAACTGAAAAAGCAAAGGAGGAATTGACCAAAGCCTTAACTGTTATGGAGCAGCTGAAAGCAAGCCGCAAAAAGGAGCAAGCCGATTGGGCTACTGAAAGAGCTCTTTTAACGAAGAGGGCTGAAAATGCCGAAGCTGCTCTTAAACCGGTGGTTGATGAATTATCCACCGTGAAGCGACAAATACACTCCATGACCTCTGCCGTCTTTGGTAAAGTTTTCGATGCCCTTCTTCCAATTTGTACCTTGTTTGCTACACCCTATGTCCGGTTTGTAATCCACCTTGAAACACTGCAGGCTCACGTATTGGACACTTGGGTTCCGACGTGCGGATGAAACTCAAAGCTGCTTATACTCTGATTGAACAGTTATACACCGGAGCACAAAGACCTATCACCACTGCATCGCACAACAACCCTGCGCCGGCCCTGATTCAAGACACATTAAAGAGGCTGTCCATGCTGCCGGCTCGGATTGAAGAGTTGAAAAGATCAGCTGCTCGAACTGGAGCTATCACTGCCTTGATCCGGGCCAAAGCATGGGTACCAGATTTCGATCCTGTCGAAGCGGCCCAAGGATACCCCAGCTTGAAGGAAGACGGTTCAGACTTTGGTGAAGATGATTTGAGGGCGATAAACCGGGCGGTGCGCCCCCTAGCTTGTCAATTGGCTGAGGAGGCAGATCTGTCACGTTACCAAGCCCAATACGACAACCAGAACAAACGAGTATCTGCCCCGGTCCCTGAAGCGGGAAACCTTGTTCCTCCAATCCGTAAGCATACTTATGCCCCAGACATTGAACCGTCTTCGCTGATTCACGAAGAAGCCGTCTTTCAAGCGCTAATGGGAATCGACTGGACCACTGCTGATTTCCAGCCAATGGGTAACCAAGATGGAGCTGAAGCGGCGCGAGACGATCCCCAGCCTTCGAACCGAGGCGTCGAACAGGCGTAATCAACGCTCCGGTTTACTTGGTGACGTCTTAATCCGTCAACGAACAATATATTCAGGCACTACGTTGCCTTGTAATAAGATAGTTTAATACTTCTGCCTTTGAATGTGCCTTCGCGCACGGGTACTGATGCTCTTTCCAATGACATTTCCAACTTATGTTTCCTGTCTTTACTGAGTACACAATATGATTTTTCGACCACCTGAACCGTGCTGCGCCTCTGCCAAACCGGGCAATTTGCCTCCGGGCTAAAACGGACCAGGCTATGCCAGCTGAATCGGTTTAAGGATACTTGACGTTCCGAAGAACTAAAAACACCACAATAAAGGCAGAAAACAAAACCAAATATGAAAAGGCAACGCGATAGTAAACGCATGCGAATAGTGGTTACTATGAACCGTTCAGAAGGCGTGACCATTGTCAAGACAAAACCGAGCCCCCAAGAGGTGTAGCTACGGTTTAAGTCGACTAGGTTCCTAACTGAATCGTGGCAAATATGCCGGCCAAATGGCATTGCATTGGTTCGTCAACCAAGCCCCCAAGTGACTCAATGGCTAAAAAGCCGACCAGAAGGCATTACCATCATCAAGACATGACCGAGTTCCCATAAGGCGTAATCTACGGTTCAAGTCGATTAGGTCCCCAACTGATTCAGTGGCAAATATGCCAGCCAGAAGGCATTACCGTCGTCAAGACCTGACCAAGCCCCCCGGAGGTGTAGCTACGGTTCAGGCCGACTAGGTCCCCAACTGACTTTATAGGGATAGCTATGTTCTCTTTGGTTCGAATACGACCTATGTTTGAACAGCTGTGCTCTCTTTGGTTCGAATACGACCTATGTTTGAACAGAAATCCCCATGTTTGAATAGCTATGTTCTCTTTGGTTCGAATACGACCTATGTTTGAACAGGAAGCCTCATGCTGAATGGCTATGTTCTCTTTGGCTCGAATACGGCCTATGTTTGAACAGTAAGCCTTATGCTTGAACCGGGATATTGAACCGGATATTAAGAGTTTCACAACCCTGTAGGAGACATCTTCCCTTGAACCAGCTTTTAAACCGGAATTTGAACTTCTGTGAAGCGACCTCCAGGGGTAAGTACACATGATGTGAAGAACAGTAGAGACCCCGCTTTACTCGAGGCTCCGGTTTATTGTATTAATCATAATATATACATGGTCATGATATGTACATAAACAGAGCCCAGGGCTCATGTATAATAAGGTCGAAGATGAGCTATGTTCCACGGCCTGCTGGTCTCCTCCTCTGACGTGCGTGAATCCTTGCGCTCTCTAATATCAATCAAGTAATATGACCCATTATTCAGATTCTTGCTGACCACAAAGGGTCCCTCCCAAGGCGGGGATAATTTATGCTGATCAGATTGATCCTGGATGAGCCGAAGCACCAAATCCCCCTCCTGAAAGGTTCTGGTCCTAACCCGACGGCTGTGGTAGCGGCGAAGATCTTGCTGGTAAATCGCTGAACGGGCAGCTGCCAAGTCACGCTCCTCATCCAACAAGTCAAGAGCGTTCTGCCATGCCTGCTCATTATCCGCTTCAACATATGCTGCCACGCGAGGTGAGTCATGTCGTATATCACTTGGGAGAACCGCCTCGGCTCCATAGACCATAAAAAACGGCGTAAACCCCGTGGATCTGTTAGGCGTGGTGTTGATGCTCCATAATACAGACGGTAGCTCCTCCACCCAACAACCCGGTGTCCTTTGCAAAGGGACCAGAAGCCGGGGTTTGATACCTCTCAAGATCTCTTGGTTCGCTCGTTCTGCCTGACCATTAGACTGTGGGTGCGCTACCGAAGAAACATCAAGCCGTATATGCTCCCGTGCACAGAACTCCTTCATGGCCCCCTTGGATAGATTGGTACCATTATCTGTGATGATGCTGTGTGGAAAACCAAACCAGAAGATCACCTTCTTTATAAACCGAACCGCCGTGGCTGCATCACACTTACTGACAGGTTCTGCCTCTACCCACTTCGTAAATTTGTCAACTGCCACCAGTAGGTGGGTCTTCTTATCTTTGGAACGCTTAAAAGGCCCAACCATATCAAGCCCCCAAGTCGCAAACGGCCAAGTGATTGGAATCATCCTCAATTCTTGAGCCGGTATGTGTGCTCGTCTGGAAAATTTCTGGCAACCATCACATAATCTGACCAGGTCTTCCGCATCAGCATGAGCAGTTAACCAGTAGAAACCGTGGCGGAACGCTTTAGCCACCAATGATTTTGAACCGGCGTGGTGGCCGCAATCCCCTTCATGTATTTCTCGTAGTATCTCACACCCCTCCTCATGGGATATGCACCGTTGCAGCGCTCCTGACACGCTGCGATGATGTAACTCTCCTTGGAAAATCGTCATAGACTTGGACCGTCGTACTATCTGTCGAGCCAAGCTTTCGTCTTCTGGCAACTCACCCTGGTTCATATATGCCAGGTAAGGAAGCGTCCAATCCGGGGTGGCGTGAAGAGCCGCCACTAACTGAGCCTCCGGATCAGGAACAGCCAACTCCAGCTCACCGGGCAGCTGTACCGATGGGTGGTACAATACATCCAAGAAAACATTGGGTGGGACCGGTTTACGCTGAGAGCCCAAGCGACTTAAAGCATCCGCTGCCTCATTCTTTCTGCGGTCCACATGATCCACTTGATAGCCTTTGAAATGCCCAGCCACCATATCTACTTCACGTCGGTATGCAGCCATAAGCGGATCCTTAGAATCCCAGGTGCCAGATACCTGCTGAGCCACCAGATCCGAATCACCAAAGCACCTAACTCTGCTCAGATTCATCTCTTTAGCCACCCGAAGACCATGAAGCAAAGCTTCATACTCCGCCGCATTATTTGTACAGGGGAACATTAAACGTAGGACATAACAAAACTTATCTCCTCGTGGGGAAGTTAAAACGACTCCAGCCCCCGAGCCTTCCAATTGTCTTGATCCGTCAAAATGAACGGTCCAATAGGTGTTATCTGGCTTCTCCTCAGGCGCTTGTAACTCTGTCCAATCGTTTATGAAATCGACAAGTGCCTGTGACTTTATGGCCGTTCGGGGTACGTACCTTAAATCGTGCGGCCCGAGCTCGATAGCCCACTTGGCAATCCGACCGGTCGCCTCCCGGTTCTGTATAATGTCACCCAAAGGAGCAGAACTGACCACCGTGATTGGGTGTCCTTGGAAATATTGCCTCAGTTTCCGGCTTACCATAAAAACGCCATACCAGCTTCTGCCAATGCGGATACCTTTGTTTAGACTCGATAAGTACTTCACTGATATAGTAAACCGGTCGCTGAACCGGATGTTCCTTTCCAGCCTCCTTTCGTTCTACCACCATCGCCACACTAACTGCTCTGGCATTCGCTGCAACATATAACAGGAGTGGCTCTTTATCTACAGGGGCTGCCAACACTGGCGGATTGACTAGTTGTCGCTTCAAGTCCTCAAACGCTTTATCAGCCTCCGGACTCCAGACAAACTGATCCGTCTTCTTGAGCATTTGATACAAAGGGATCGCCTTTTCACCAAGGCGACTGATAAACCGGCTTAACGCTGCAATCCGCCCGGCCATGCGCTGAACATCATTGATGCATTTCGGTTTGGCCAGGGAGGTGATGGCTGTGATCTTTTCTGGATTGGCCTCAATTCCTCTATGGGACACTAAAAATCCCAACAGCTTGCCTGCAGGTACACCAAAAACACATTTAGCCGGGTTGAGCATCATTTGGTACACTCTCAAGTTATCGAAAGTCTCCTTCAAATCGTCAACCAGGGTCTCCTTCTTTCGTGACTTGACCACAATATCATCCACATAAGCATGCACATTACGACCAATCTGCTCGTGGAGGCAATTTTGCACACATCGTTGATAAGTAGCCTGCGCACTCTTAAGCCCAAAGGGCATAGACACATAGCAGAAGGCTCCAAAGGGAGTAATAAACGCTGTCTTCTCCTGGTCCTTAACTGCCATCTTGATCTGATGATAGCCCGAATAGGCATCCAAAAAACTTAAACGCTCGCAACCTGCCGTAGCATCAATGATCTGATCAATCCGGGGGAGAGCAAAAGGATCTGCTGGGCAAGCTTTATTCAAGTCTGTGTAGTCCACACACATGCGCCAGGTGCCGTTTTTCTTAAGAACTAGCACCGGATTAGCGAGCCATTCGGGATGAAAAACCTCAATGATAAACCCAGCTGCCAGGAGCCTGGCCACTTCCTCACCAATCGCTTTACGTCTTTCTTCGTTAAACCGCCGTAAGAACTGCTTTACCGGCTTGTACTTAGGATCCACATTAAGGGTGTGCTCAGCGAGTTGCCTCGGTACACCAGGCATGTCAGATGGCTTCCATGCAAAGATGTCCCGATTCTCACGGATGAACTCGATGAGCGCGCTTTCCTATTTTGGATCTAAGTTTGCACTGATGCTGAACTGCTTGGATGAATCGCCAGGTACAAAATCAACAAGCTTAGTTTCGGCCGCTGACTTGAACTTCAAGGCCGGATCGTGGTCCGTAGTTGGCTTTTTTAATGAAGTCATATCCTCCGGATCAACACTGTCTTTGTACTGCTTTAGCTCCTCCGTAGCACAAACCGATTCAGCATAAGTTGCATCTCCTTCCTCGCACTCCAAAGCGATTCGGCGGCTCCCATGAACCGTTATAGTGCCCTTGTAACCTGGCATCTTAAGCTGCAGATATACGTAGCAGGGCCGTGCCATAAATTTTGCATAAGCTGGCCGCCCAAACAAAGCGTGGTACGGACTTTGGATTTTAACCACCTCGAACGTCAGCGTCTCTGATCTGGAATCGTGACTGTCGCCAAATACCACTTCAAGGGCTATCTTACCAACCGGATACGCAGACTTGCCAGGCACCACCCCGTGGAACACAGTATTGGTCGGTCTGAGATCCTTATCTACCAGTCCCATACGACGGAAGGTCTCATAATACAAGATGTTAATGCTGCTCCCTCCGTCCATGAGCACCTTGGTGAGCTTATAACCTCCCACCTGGGGCGCTACCACCAGTGCTAACTGACCCGGATTATACACCCTGGGAGGATGATCTTCTCGGCTCCATATGATAGGCTGTTCCGACCAACGCAGATAGTGAGGCGTGGCCGGTTCGACAGCACTGACTGCCCGCCTCTGAAGCTTCCTATCTCGTTTATCCAAGCTCGTAGTAAAGACATGATACTGTCCACCACTCAACTGTTTCGGGTTGCTCTGATAACCACTTTGATTACTTTGATTATTCTGATTATTTTGGCTGACCTGTCCGCCCGGGTTACCCTGGAACCCTGGACCGGAACCTGAACCGCCGCCGGAGCTGCGATCATTCTGGAACGTGTTTGAACTTTTGAACTCCTTCATGATATAACAATCCTTCCAAAGATGGGCTGCTGGTTCCTCCTTTGTTTCGTGCTTCGGACAGGGCTGGTTTAAGAAAAAGTTCAAACGCTCCGGATTAGGACCAGGATCTGTGCGGAAACTTGGCTGTTTTCCCTTGCGCCGCTGGCCCTTATTCTGTGCACTTGCGTTGGCTACAAAGTCCATGCTCCCATCCGGTTTACGCTTACCTCCGCCTCCGTTACCTGTCGGTCGATGCTGCTGGCCTTTGACGTTGCTATTCTTCCTTCCCTTCCCTGCTTTGTCATCACCAGAGTCAGGATCCTTGGTACTGTTAGAATCCGCATACTTCACTAAAGCGGTCATGAGCGTTCCTATATCTGTGCAATCACGCTTCATCCGGCCCAGCTTTAGCTTCAAAGGGCCAAACCGACAGTTGCTTTCCAACAGCACTACAGCGGTGTCTGCGTTGATGCGATCTGATGAGTGCAAGACTTCTGATACTCGTCGTACCCAATGGGTAGTTGATTCCCCTTCTTGCTGAACGCAGGCTGCTAGGTCAACTATTGACATTGGCTGTTTGCACGTGTCCTTGAAGTTCTTGATAAACCGGGCACGCAATTGGGCCCATGAGCTAATTGAATTAGGCGGCAAACTCTTTAGCCAAGTCCGGGCCGTTCCTTCTAACATCATAGTAAAATACTTGGCACACGCCGTGTCATCCACATCCAGCATCTCCATAGCCATTTCATAGCTCTCCACCCATGTCTCCGGAGGCTGATCTGCCGTGTAGTTTGGTACCTTGCGGGGGCCTTTGAAGTCTTTGGGCAGGCGTACGTTGCGTAAAGCGGGAACAAGGCACGGTACTCCCAAAGAACTGGAAACCACCCCCGGTTCGACCGTAGCTGCGGGGAAGATAGGTGTAAGCTGACGAGCCTGATGCTGCACTTCTAATCCGGCCTCCCGTGCACGGGTGCGATCCACATTTTCGTGAGTATTGTCGACGCCCGCCGGATTGTTGGCGCGGAGCGCTTCACGCCTGGCGTTACTTGAAACGGCCGGCTCCTCCACGCGCCTACTATAGCTCCTTCTGGGGCGAGGGGTGGAGTGAATCCGATCACGGCTTTGCGAATACGCTTCTTGCTGGGCCAACGCAGTCCTAAGGAGCTCCTTGACCCGGCGTGTCTCTTCATCCTGCGGTGAATCTCCTTCGATCGGAATGGCTTCCAACCGAGCCGCCGCCGCGACGAGGTTATCCATCGGGTTGGAATAATGACCTGACGGCGTTGGCATGGCCGGTGGCGTATCAGTGTTATGACGAGGCAGGTCTATAATCCGGTGCTGAACCGGGGCGCCGGCTGTCGCCTCCGCACGGTTTACCCCCGGGGTGTTGAAGAGATTTCGAGCGTCGAGGGCGGCGGGCAATCGAGACCGGGATTTTCTCTTCAGGACCTCCTGAGATGCCTTCTGGTCCATCATGAGCCTGTAAGCCTGTGCTTCTAAAGCGGCGCGCTCCGCCGTCATCCTGATCTCTTCTGCTGCCAGCTCCGTTTTAGCCCGAGTGATCTGCTCTCTCACTTTGGCAATCTCAGCATTGTGAACCTCCTGATCCACCGGGTTGGCCTCCGCCATGAGCGCTGCTAGTGCGTCAAACAGGTCTGACAAAACTTGGGCTGGTGAACCGGTAGGGCCTCCTGCCCGCGCAGCCGTTGCTGCTGCCGATCCGGAGATCGCTGCTGCTGCAGTTGATGATCATGGCGTCGCCTGTGTCCCAGCCATGAAGATCCCAACCCGATAGGGCAGATCAGAGGGGTCCGGAATACTGTTGCCATCGGAGCCGCCCCCAACCCGATCATCTTGTAGCTGGTAAAGAGATTCGGTCTCCCCGGTCGACGTTTCATCGCCAGAGTAGATGGCGGTTTCGTCACCGGATTCGGATCCTTCCTCGTAGCTTCCCCCGTGGATGACTCCCACGAAGGCGTGCTTCCTGGTCGGTTTAGCCAGGGTCGATCTCGTGCACTGAGCTGTCTCAATGAGGTCGGCGCAGTCGCCTAACTCGGGGCCCGGTTCTCCGATCTTGCCGATGAAAACATGGATGCCGCCAAAGGGGACCCGGTACCCGTACTCGATCGAACCGGCGTCGGGGCCCCAGCCTGTGCTGTCGATGTAGAGTTTTCCGCGGCGGCTCTTGGTCATCCGTCCCACAGCGTAACCCTCGAGTCCTTCAAAGCGGCCCTCCAAGAACCTGAAACCATCTCGCGATAGCCCCACGGTGGGCGCCAAC

TGTCGTGGATTTGTCACGGCAGATGTCCTAGTAAGAGGACTTAGTCGTGGAGCCATCGCAACGGGTTAGCTTGAAGGGGTTAAAGTGGACACAAGGACACGGTTTTATACTAGTTCGGCCCCTTCAATGAAGGTAAAAGCCTACGTCTAGTTGTGATGGGATTGATGGGTTTCGAGGGCTTAGGGAGCGATAAGCTTCGCCTAAACCTCGAGTTCTTTTTTCTCTGTCCCCAAACCGCCGTCGGGTCGTCCCCTTATATACACGGGTGACGCCCGTCGGTCTGCAAAGTTCCAACCTCCGGCTAATAAACGTGCCCGGGTCGGTCTCTCTATTCCTAACTTACAATACAAGTTTACATCAGGCCGGTTTACACCATAAACCTTTAAACCGATTACGGGCCTTAGGCCCTTCTATCTCTCCTTGGGTTTTAACATCTCGGGTCTGCTGACGGAGGTAACCCGGGCCCTGCAGGCCGGTTTACCCCGGATAGCAACATCCCCAACA

GGATGGAAAAGACCATTCTCGAGTTGTATTCAATGCTGAAATCTGCGGAGGTGGAGATCAAAAAGGCACATCAAGTGTTGATGGTGAATAAAACCACTAAGTTCAAGAAAGGCAAGGGTAAGAA

>Aegilops umbellulata 1-324;

ATCAATGAAAGTTTCCATAGCACACCATACGAAAGGACGAACATGCAAACCCTATCAGACAAACAAAAACAAAGAAATCTACATAATAAGCAACAGGAAACAAAACATGGCCGGGCTTACCTTG

TGTTGGGGATGTTGCTATCCGGGGTAAACCGGCCTGCAGGGCCCGGGTTACCTCCGTCAACAGACCCGAGATGTTAAAACCCAAGGAGAGATAGAAGGGCCTAAGGCCCACAATCGGTTTAAAGGTTTATGGTGTAAACCGGCCTGATGTAAGCTTGTATTGTAAGTTAGGAATAGAGAGACCGACCCGGGCACGTTTATTAGCCGGAGGTTGGGACTTTGCAGACCGACGGGCGTCACCCATGTATATAAGGGGATGACCCGGCGGCGGTTTGAGGACAGAGAAGAAGAACTCGAGGTTTAGGCGAAGCATATCGCTCCCTAAGCCCTCGAAACCCATCAATCCCATCACAACTAGACGTAGGCTTTTACCTTCATCGAAGGGGCCGAACTAGTATAAAAACCGTGTCCTTGTGTCCACTTTAACCCCTTCAAGCTAACCCGTTGCGATGGCTCCACGACTAAGTCCTCTTACTAGGACATCTGCCGTGACAAATCCACGACA

GTTGGCGCCCACCGTGGGGCTATCGCGAGATGGTTTCAGGTTCTTGGAGGGCCGCTTTGAAGGACTCGAGGGTTACGCTGTGGGACGGATGACCAAGAGCCGCCGCGGAAAACTCTACATCGACAGCACAGGCTGGGGCCCCGACGCCGGCTCGATCGAGTACGGGTACCGGGTCCCCTTTGGCGGCATCCATGTGTTCATCGGCAAGATCGGAGAACCGGGCCCCGAGTTAGGCGTCTGCGCCGACCTCATCGAGACAGCTCAGTGCACAAGGTCGACCCTAGCTAAACCGACCAGGAAGCACACCTTCGTGGGAGTCATCCACGGGGGAAGCTACGAGGAAGGATCCGAATCTGGTGACGAAACCGCCATCTACTCTGGCGATGAAACGTCGACCGGGGAGACCGAATCTCTTTACCAGCTACAAGATGATCGGGTTGGGGGCGGCTCCGATGGCAACAGTATTCCGGACCCCTCTGATCTGCCCTATCGGGTTGGGATCTTCATGGCTGGGACGCAGGCGGCGCCACGATCATCAACTGCAGCAGCAGCGATCTCCGGATCGGCAGCAGCAACGGCTGCGCGGGCAGGAGGCCCTACGGGTTCACCAGCCCAAGTTTTGTCAGACCTGTTTGACGCACTAGCAGCGCTCATGGCGGAAGCCAACCCGGTGGATCAGGAGGTTCACAATGCTGAGATCGCCAAAGTGAGAGAGCAGATCACTCGGGCTAAAGCGGAGCTGGCAGCAGAAGAGATCAGGATGACGGCGGAGCGCGCCGCTTTAGATGCACAGGCTTACAGGCTCATGATGGACCAGAAGGCGTCTCAGGAGGTCCTGAAGAGAAAATCCCGATCTCGGTTGCCCGCCGCCCTCGACGCTCGAAATCTCTTCAACACCCCGGGGGTAAACCGTGCGGAGGTGACAGCCGGCGCCCCGGTTCAGCACCGGATTATAGACCTGCCTCGTCATAACACTGATACGCCACCGGCCATGCCAACACCGTCAGGTCATTATTCCAACCCGATGGATAACCTCGTCGCGGCGGCGGCTCGGTTGGAAGCCATTCCGATCGAAGGAGATTCACCGCAGGATGAAGAGACGCGCCGGGTCAAGGAGCTCCTTAGGACCGCGTTGGCCCAGCAAGAAGCGTATTCGCAAAGCCGTGATCGGATTCACTCCACCCCTCGCCCCAGCAGGAGCTATAGTAGGCGCGTGGAGGAGCCGGCCGTTTCAAGTAACGCCAGGCGTGAAGCGCTCCGCGGCAACAATCCGGGGGGCGTCGACAATACTCACGAGAATGTGGATCGCGCCCGTGCACGGGAGGCCGGATTGGCAGCGCAGCATCAGGCTCATCAGCTTACACCTATCTTCCCAGCAGCTGCGGCTGAACCGGGGGTGGTTTCCAGTTCTTTGGGAGTACCGTGCCTTGTTCCCGCTTTGCGCAACGTACGCCTGCCCAAAGACTTCAAAGGCCCCCGCAAGGTACCAAACTACACGGCAGATCAGCCTCCGGAGACATGGGTGGAGAGCTATGAGATGGCTATGGAGATGCTGGACGTGGATGACACGGCGTGTGCCAAGTATTTTACTATGATGCTAGAAGGAACGGCCCGGACTTGGCTAAAGAGTTTGCCGCCTAATTCAATTAGCTCGTGGGCCCAATTGCGCGCCCGGTTTATCAAGAACTTCAAGGACACGTGCAAACAGCCAATGTCAATAGTTGACCTAGCAGCCTGCGTTCAGCAAGAAGGGGAATCAACTACCCATTGGGTACGACGAGTATCAGAAGTTTTGCACTCATCAGATCGCATCAACGCAGACACCGCCGTAGTGCTGTTGGAAAGCAACTGTCGGTTTGGCCCTTTGAAGCTAAAGCTGGGCCGGATGAAGCGTGATTGTACAGATATAGGAACGCTCATGACCGCTTTAGTGAAGTATGCGGATTCTGACAGTACCAAGGATCCTGACTCTGGTGATGACAAAGCAGGGAAGGGAAGGAAGAATAGCAACACCAAAGGCCAGCAGCATCGACCGACAGGTAATGGAGGCGGAGGTAAGCGTAAACCGGACGGGAGCATGGACTTTGTAGCCAACACAAGTGCACAGAATAAGGGCCAGCGGCGCAAGGGGAAACAGCCAAGTTTCCGCACAGATCCCGGTCCTAATCCGGAGCATTTGAACTTTTTCTTAAACCAGCCCTGTCCGAAGCACGGAACAAAGGAGGAGCCAGCAGCCCATCTTTGGAAGGATTGTTATATCATGAAGGAGTTCAAAAGCTCAAACACTTTCCAAAATGATCGCAGCTCCGGCGGCGGTTCGGGTTCCGGATCAGGGTACGGTGGCGGAAATTCCGGTCCAGGGTTCCAGGGCAATCCGGGCGGACAGACCAGCCAAAATAATCAAAGTAATCAGAGTGGTTTCGGGCAGCAGCAAACAGGTTATCAGAGCAACCCGAAACAGTTGAGTGGTGGGCAGTATCATGTCTTTACTACAAGCCTGGATAAACGGGATAGAAAGCTTCAGAGGCGAGCAGTTAGTGCTATCGAACCAGCCACGCCTCACTATCTGCGCTGGTCAGAACAGCCTATCATATGGAGCCGAGAGGATCACCCTCCCAGGGTCTATAATCCGGGTCAGTTAGCATTGGTGGTGGCGCCTCAGGTGGGAGGTTATAAGCTCACCAAGGTGCTCATGGACGGAGGGAGCAGCATTAACATCTTGTATTACGAGACCTTCCGTCGTATGGGACTGGTAGATAAGGATCTCAAACCGACCAATACAGTGTTCCACGGGGTGGTGCCTGGCAAGTCTGCATATCCGGTTGGTAAGATAGCCCTTGAAGTGGTATTTGGCGATAGTCACGATTCCAGATCAGAGACGCTGACGTTCGAAGTGGTTAAAATCCAAAGTCCGTACCACGCTTTGTTCGGACGGCCAGCTTATGCAAAATTTATGGCACGGCCTTGCTACGTATATCTGCAACTTAAGATGCCAGGTTACAAGGGCACTATAACGGTTCATGGGAGCCGTCGAATCGCTTTGGAATGCGAGGAAGGAGATGCGACTTATGCAGAATCGGTTTGTGCTACGGAGGAGCTAAAGCAGTACAAAGACAGTGTTGATCCGGAGGATATGACTTCATTAAAAAAGCCAACTACGGACCACGATCCGGCCTTGAAGTTCAAGTCAGCGGCCGAAACTAAGCTTGTTGACTTCGTACCTGGCGATACATCCAAGCAGTTCAGCATCAGTGCAAACTTGGATCCAAAATAGGAAAGCGCGCTCATTGAGTTCATCCGTGAGAATCGGGACATCTTTGCATGGAAGCCTTCTGACATGCCTGGTGTACCGAGGCAACTCGCTGAGCACACCCTTAATGTGGATCCTAAGTACAAACCGGTAAAGCAGTTCTTACGGCGGTTTAACGAAGAAAGACGTAAAGCGATTGGTGAGGAAGTGGCCAGGCTCCTGGCAGCTGGGTTTATCATTGAGGTTTTTCATCCCGAATGGCTCGCTAATCCGGTGCTAGTTCTTAAGAAAAACGGCACCTGGCGCATGTGTGTGGACTACACAGACTTGAATAAAGCTTGCCCAGCAGATCCTTTTGCTCTCCCCCGGATTGATCAGATCATTGATGCTACGGCAGGTTGCGAGCGTTTAAGTTTTTTGGATGCTTATTCGGGTTATCATCAGATCAAGATGGCAGTTAAGGACCAGGAGAAGACAGCGTTCATTACTCCCTTTGGAGCCTTCTGCTATGTGTCTATGCCCTTTGGGCTTAAGAGTGCGCAGGCTACTTATCAACGATGTGTGCAAAATTGCCTCCACGAGCAGATCGGTCGTAATGTGCATGCTTATGTGGATGATATTGTGGTCAAGTCACGAAAGAAGGAGACCCTGGTTGACGATTTGAAGGAGACTTTCGATAACTTGAGAGTGTACCAAATGATGCTCAACCCGGCTAAATGTGTTTTTGGTGTACCTGCAGGCAAGCTGTTGGGATTTTTAGTGTCCCATAGAGGAATTGAGGCCAATCCAGAAAAGATCACAGCCATTACCTCCCTGGCCAAACCAAAATGCATCAATGATGTTCAGCGCATGGCCGGGCGGATTGCAGCGTTAAGCCGGTTTATCAGTCGCCTTGGTGAAAAGGCGATCCCCTTGTATCAAATGCTCAAGAAGACGGATCAGTTTGTCTGGAGTCCGGAGGCTGATAAAGCGTTTGAGGACTTGAAGCGACAACTAGTCAATCCGCCAGTGTTGGCAGCCCCTGTAGATAAAGAGCCACTCCTGTTATATGTTGCAGCGAATGCCAGAGCAGTCAGTGTGGCGATGGTGGTAGAACGAAAGGAGGCTGGAAAGGAACATCCGGTTCAGCGACCGGTTTACTATATCAGTGAAGTACTTATCGAGTCCAAACAAAGGTATCCGCATTGGCAGAAGCTGGTATATGGCGTTTTTATGGCAAGCCGGAAGCTGAGGCAATATTTCCAAGGACACCCAATCACGGTGGTCAGTTCTGCTCCTTTGGGTGACATTATACAGAACCGGGAGGCGACCGGTCGGATTGCCAAGTGGGCTATCGAGCTCGGGCCACACGATTTAAGGTACGTACCCCGGACGGCCATAAAGTCACAGGCACTTGTCGATTTCATAAACGATTGGACAGAGTTACAAGCGCCTGAGGAGAAGCCAGATAACACCTATTGGACCGTTCATTTCGACGGGTCAAGACAATTGGAAGGCTCGGGGGCTGGAGTCGTTTTAACTTCCCCACGAGGAGATAAGTTTTGTTATGTCCTCCGTTTAATGTTCCCCTGTACAAACAATGCGGCTGAGTATGAAGCTTTGCTTCATGGACTTCGGGTGGCTAAAGAGATGAATTTGAGCAGAGTTAGGTGCTTTGGTGATTCGGATCTGGTGGCTCAGCAGGTATCTGGCACCTGGGATTCTAAGGATCCGCTTATGGCTGCGTACCGACGTGAAGTAGATATGGTGGCTGGGCATTTCAAAGGTTATCAAGTGGATCATGTGGACCGCAGAAAGAATGAGGCAGCGGACGCTTTAAGTCGCTTGGGCTCTCAGCGTAAACCGGTCCCACCCAATGTTTTCTTGGATGTATTGTACCACCCATCGGTACAGCTGCCCGGTGAGCTGGAGTTGGCTGTTCCTGATCCGGAGGCTCAGTTAGTGGCGGCTCTTCACGCCACCCCGGATTGGACGCTCCCTTACCTGGCATATATGAACCGGGGTGAGTTGCCAGAGGATGAAAGCTTGGCTCGACAGATAGTGCGACGGTCCAAGTCCATGACCATTTTCCAAGGAGAGTTACATCATCGCAGCGTGTCAGGGGCGCTGCAACGGTGCATATCCCCTAAGGAGGGGTGCGAGATACTACGAGAAATACATGAAGGGGATTGCGGCCACCACGCCGGTTCAAAATCATTGGTGGCTAAAGCGTTCCGCCACGGTTTCTACTGGTTAACTGCTCATGCTGATGCGGAAGACCTGGTCAGATTATGTGATGGTTGCCAGAAGTTTTCCAGACGAGCACACATACCGGCTCAAGAATTGAGGATGATTCCAATCACTTGGCCGTTTGCGACTTGGGGGCTTGATATGGTTGGGCCTTTTAAGCGTTCCAAAGATAAGAAGACCCACCTACTGGTGGCGGTTGACAAATTTACAAAATGGGTGGAGGCCGAACCTGTCAGTAAGTGTGATGCTGCCACGGCGGTTCGGTTTATAAAGAAGGTGATCTTCCGGTTTGGTTTTCCACACAGCATCATCACAGATAATGGTACCAATCTGTCCAAGGGGGCCATGAAGGAGTTCTGTGCACGGGAGCATATACGGCTTGATGTTTCTTCGGTAGCGCACCCACAGTCTAATGGTCAGGCAGAACGAGCGAACCAAGAGATCTTGAGAGGTATCAAACCCCGGCTTCTGGTCCCTTTGCAAAGGACGCCGGGTTGTTGGGTGGAGGAGCTACCGTCTGTATTATGGAGCATCAACACCACGCCTAACAGATCCACGGGGTTTACGCCGTTTTTTATGGTTTATGGAGCAGAGGCGGTTCTCCCAAGTGATATACGACATGACTCACCTCGCGTGGCAGCATATGTTGAAGCGGATAATGAGCAGGCACGGCAGAACGCTCTTGACTTGTTGGATGAGGAGCGTGACTTGGCAGCTGCCCGTTCAGCGATTTACCAGCAAGATCTTCGCCGTTACCACAGCCGTCGGGTTAGGACCAGAACCTTTCAGGAGGGGGATTTGGTGCTTCGGCTCATCCAGGATCAATCTGATCAGCATAAATTATCTCCGCCTTGGGAGGGACCTTTTGTGGTCAGCAAGAATCTGAATAATGGGTCATATTACCTGATTGATATTCGAGAGCGCAAGGATTCACGCACATCAGAGGAGGAAACCAGCAGGCCGTGGAACATAGCTCATCTTCGACCTTATTATACATGAGCCCTGGGCTCTGTTTATGTACATATCATGACCATGTATATATTATGATTAATACAATAAACCGGAGCCTCGAGTAAAACGGGGTCTCTACTGTTCTTCACATCATGTGTGCTTACCCCTGGAGGTCGTTTCACAGAAATTCAAATTATAGACTCCGATTTAAAAGCCGGTTCAAGGGAAGATGTCTCCTATAAGGCTGTGAAGCTCCTAATATCCGGTTCAATATCCCGGTTCAAGCATGAGGCTTCCTGTTCAAACATAGGTCGTATTCGAACCAAAGAGAACATAGCTATTCAAACACGGGGCTTCCTGTTCAAACATAGGTCGTATTCGAACCAAAGAGAACATAGCTATCCCTATAAAGTCAGTTGGGGACCTAGTCGGCCTGAACCATAGCTACACCTCCGGGGGGCTTGGTCATGTCTTGACGACGGTAATGCCTTCTGGCTGGCACATTTGCCATTGAATCAGTTGGGGACCTAATCGACTTGAACCGTAGATTACGCCTTATGGGAACTCGGTCATGTCTTGATGATGGTAATGCCTTCTGGTCGGCTTTTTAGCCAATGAATCATTTGGGGGCTTGGTCGTGTCCGAACCAACGCAATGCCACTTGATCGGCCCTTGGCCATTGAGTCACTTGGGGGCTTGGTTGACGAACCAATGCAATGCCATTTGGCCGGCATATTTGCCACGATTCAGTTAGGAACCTAGTCGACCTAAACCGTAGCTACACTTCTTGGGGGCTCGGTTTTGTCTTGACGATGGTCACGCCTTCTGAACGGTTCATAGTAGCCACTATTTGCATGCGTTTTACTATCGCGTTGCCTTTTCATATTTGGTTTTTGTTTTCTGCCTTTATTGTGGTATTTTTGTTCTTCGGAACGTCAAGTGTTTTTAAACCGATTAGGCTGACATAGCCTGGTCCGTTTTTTAGCCCAGAGGCAAGTTGCCCGGTTTGGCAAGGGCGCAGCACGGTTCATAGGGCCGGAAGATCTTGTGTACTCAATAAAGACAGGAAACAAAGTTGGAAAACGTCATTTGAAAGAGCATCAGTACCCGTGCGCGAAGGCACATTCAAATGCAGAGGTATTAAACTATCTTATTACAAGGCAAACGTAGTGCCTGAATATATTGTTCGTTGACGGATTAAGATGTCACCAAGTAAGGTAAACCGGAGCGTTGATTACGCCTGTTCGCCGCCTCGGTTCGAAGGCTGGGGATCGTCTCGCGCCGCTTCAGCTCCATCTTGGTTACCCATTGGCTGGAAATCAGTAGTGGTCCAGTCGATTCCCATTAGCGCTTGAAAGACGACTTCTTCGTGAATCAGCGAAGACGGTTCAATGTCTGGGGCGTAAGTATGCTTACGGATTGGAGGAATAAGGTTTCCCGCTTCAGGGACCGGAGCAGCCACTCGTTTGTTCTGGTTGTCGTATTGAGCTTGGTAACGTGACAGATCTGCTTCCTCAGCCAATTGACAAGCTAGGGGGCGCACCGCCCGGTTTATCGCCCTCAAATCGTCTTCACCAAAGTCTGAACCGTCTTCCTTCAAGCTGGGGTATCCTTGGGCCGCTTCGACAGGATCGAAATCTGGTACCCATGCTTTGGCCCGGATCAAGGCAGTAATGGCTCCAGTTCGAGCAGCTGATCTTTTCAATTCTTCAATCCGGGCTGGAAGCATGGACAGCCTCTTTAATGTGTCTTGAATCAGAGGCGACGCAGGGTTGTTATGCGACGCAGTGGTAATAGCCCTTTGTGCTCCGGTGTACAACTGTTCAATCAGAGTATAAGCAGCTTTGAGTTTCATCCGCACATCAGAACCCAAGTGTCCAATACGTGAGCCTGCAGTGTTTCAAGATGGATTACAAACCGGACATAGGATGTAACAGATAAGGTACAAATTGGAAGAAGGGTGTCGAAAAGCTTACCAAAGACAGCAGAGGTCATGGAGTGTATCTGTCGCTTCACGGTGGATAATTCATCGACCACCGGTTTAAGAGCAGCTTCGGCATTTTCAGCCCTTTTTGTCAAAAGAGCTCTCTCAGTAACCCAATCAGCTTGTTCTTTCTGATGGCTTTCTTTCAGCTGTTCCATAACGGTTAAGGCATGGGCCAATTCGTCCTTCGTTTTTTCAGTTTCAGCCTGTTGGGTTTTTAAGATTTCCTGAAGGTCCGAGACTTGTTTCGCTTTGGTCTTTAGATCCCCCTGCAGCAGGTCGTCAAAACAGTGTTCAGAAAATATTGCACGGTTTGGAAGTATCAACCACACAAGCAAGTTATGTGCTCGATACTTGGGGGATAATGCATATTTGATTTTAACGCAAGTTTCCCATTGCAAAAAGTCCCAAGATTAATACAAGTATTTAAACTTGGCACTTGGGGGCTAATAGTTATCTAATGATGTTTTCTGGTTATGACCTAAGTTGAAGCCTTGGGTTTTCTAAACCGGACTTTAATTGTCAAGACAGCCTGATCCGGGTTTACCATCTCGGTTTGGAAATATTGATAAGCTAAGTTGCAATGGAATGAAGCACAAGGGAGTTACCTCAAGTTTCTCCTTCATCATCTTGATCATGCCAGCTTCATAATCCCGACTGGTGTACAGTCGGTTCAGGTACCCGGAATAAAGATCCGGGGCGCTTAAAGTGGCGTACGTAGAAGAGTCAAGGTCCCATTTGCCCTTCATGGCCGAAATTTCGTCCTTGGCAGTATGCTTGGCTAAGGTAGCAGTGTTCCCCGGTTCAGAGTGACCGATGCCGGTAATCACAACATCATCATCCTTGGAATCGCTGCTCTGCAGTGGCCCTGTCGGTTTATCAGCAGTTCCAGAAGGGGGGATGGATTCCTCAACCCGAACAGGACTGGCAGGCGGATCAGTATGGCTGGCAGGTTCGATAATCCTTTCTTCAGCGCAGGTGTCATCTTGCGGAGATAGATGACTCTGAGTATCTTCACGACCCAGAGCGTCAGCACCAGGAGTTTTCTCCGGTTCAGGAGCGGCAGTTCCTCCAGCCGGGTTATCCAGACGAGCCTTCTTGCTCGGTCTAGGTTTGGCCCTGGAAAAAGCCCAAAGATTGAAAAAACGGTATAAGATGGATTAAGAGACCCATACAGAGATATGTTGTGATCGGTCCAACTTACCCAATAACTGTCTTCAACGGTGGCAGTTGAGTGGCTGAAGATTCACCGGAAGATGAATGGGAAGTGACCTGATAATCGGAGTCAGATGGATTTAGAGGCTGACGAGTTAAACCTGCTTTAGGAGGAAAGCTAGCGCATAAATTGGAGACCTCTGAGCGGCGTTTCCGAACCGGGCTGTTCGGTAAACCGGCGGAGGTGACTACCTGGCCGCTATGCCGAGTGGTGCGTCGAGCTTCATGTTGCTGGGTCTTCACAAGAAATTTGGGATCCAAATAAGCAAGAGGGTGAGAAAATTTAATTTTCCGGTTTGCTTGGCGAGGTTTTTTCAAAGGCAAAGGAACGGCATCAGAAGAAAGAATAGTTACCTCTGGGCCTTCAGCATTGCTCGCTTCAGCGTCGTCCTGACAAATATCATCATCAATAAGACGTACAAAAAGTAAGCCAACTGAATCAAGGTCTACCCCAAGGTCCGGATTACCCACATCATCCTCCTCTAAATCGGGGGTACAGGAAGCGGTGTGCCTTTTCTTTGTAGCAGGCTTCTTCACCCTTGTCTTGGGTCTGATTATCTTTTCGGTTGGCTCCTGAAGCTTCTTGCTCCAAAACGGATCATCTCCCTGTTTCGGAAAGGACTGGAAGTTAGCAATATATATAAGGAGATCAAAAATTCTATAAACAGGGAGGAGTTACTTACAGCAGGTGGTTTGTTTGTGGCGCAAAATGGAAGCAGGCCGGTTCGAGCGCAAACATGTTCCGGTTCGTTCAGCATCTTTTTCACGGCTTCAGCGACTTCGTCCTCCGTCAGCTGAATGTTGCAGTGCCTCAAAGGATCATCAACCCGACCCGTGTATTGGCACATCAAACCGGAGCGGATGCTAAGGGGCAGGATGCTCCATGAAATCCAGCAACGAGCAAGATCAACCCCCGTTAAACCATTAGCCATGAAGGCTCTGAGCTTGGACAGCTGAGGGGCATACTTACTCCTCTCTTTGGCAGTTAGCCGTGGGGGGAAGGGGTGAGTGTTGCTAAGTCTCTCTGGACGGAAACCGGGCAGTGGATTTTCTTCAGCAGGGGAAGTATCCTTGCAATAGAACCAAGTGCAATTCCATTCTTGCGGATGACTATGCAACTTGGCGTGAGGATAAGTCACCTCTTTCCTCTTCTGAATCGCCATACCACCCAGTTCCATATTAGGGCCGTCGGTGAATTCAGTACGTCGGTTCAAATGGAAAAAGTCCCGGAACAGCTCTACAGTAGGTTCTTCTTGGAAGTACGCCTCGCAAAGTACTTGAAAATGGCAGATATTCGTGACTGAATTTGGACCAACATCTTGTGGGCGCAGCCTAAAATTAGCCAAGACGTCCCGATAAAATTTTGAACCGGGTGGATTAAACCCCCGGGCCAGGTGATCCGCAAACACCACCACTTCTCCCTCCTGCGGGGTAGGAGGACATTCGTCTCCTGGAACCCTCCAATGGATGGTACTTTTGCTACTCAAAGCACCAGTCAGAACTAAGTTGTTTAGTTGGGTCTCAGTGATGCAAGAGGGAACCCAGTTGCATTCATACACCTGTTTTGCCATGAGGAAATCTACAAGGCAAAGGTATTCCGGTTTGAGAGCAAGTATGATTGTAAACCGAAGATGCTCTTATATAGAGTCAGGTTATCTACAATAATATCAACCGGCGGTTCATCAAGGGGACTAATGGATGTGTAGATCGGTTTGTTCGCTTTGCTAAGAGTTAAACCGACCAGATCTAAACAGTTGTGATTATGAAAGCAGAAACAGATCCCGTACGGATTCTTTCAAAGCAATGGATCTACAGCTAGGGCGAAAAAGAAATAAAAACAGGTAATATGAAGTCGGGCTCACAGCAGAACAACATAAGTTCGACAAGCAGGAACAAGAGTGAGATCTAAGGAAATGAGTGCAGATTTATGGCGACTGCTAAGAGGAACTACAAATCCAGATACAGGTATCATCATCTGTTCGCAAAGGCTGCTATGGGGATGAGCAAGAAACAGTAGCAGGACGATGAACCCTAGAACAGATCTAAAGCAACAAGAACAGGGGCCTTACCGGGGTCCAAACAGATGTGGAAAGTCGCCGCGGTGCTCTGATCCGATCAGGTTGATGCAGCGGCCGGAGTTGATGCAGCAGCTGACGGCGGCGGCGGAGCTTTGAGGTCTGGGTCGCGAGGAAGACGAAGAAGGGGAGGAAAGGAAGATGGCCTTCAAGTCCTATTTATAAGGCGCGGCGCGCGTGTCAGGCGCGCGAATCCAGGGGCCGGAGATTTCGGAAGCCCAGCCGTCGCCCTGATTCTTGCGGGTTAGCGAAAGGAGACGGTAGCTTGCGCTGACGTCATATCGGTTTACCACAGCAAGAGGAGACGGAGAAACGACGGTTTAACAAGTCACTGGAAGACATTTGAAGACAGAAATTCTTATTAAGGATTGACATGAACCTGTTCAAATCAATCTGGGGCCTAA

TGTTGGGGATGTTGCTATCCGGGGTAAACCGGCCTGCAGGGCCCGGGTTACCTCCGTCAACAGACCCGAGATGTTAAAACCCAAGGAGAGATAGAAGGGCCTAAGGCCCACAATCGGTTTAAAGGTTTATGGTGTAAACCGGCCTGATGTAAGCTTGTATTGTAAGTTAGGAATAGAGAGACCGACCCGGGCACGTTTATTAGCCGGAGGTTGGGACTTTGCAGACCGACGGGCGTCACCCATGTATATAAGGGGATGACCCGGCGGCGGTTTGAGGACAGAGAAGAAGAACTCGAGGTTTAGGCGAAGCATATCGCTCCCTAAGCCCTCGAAACCCATCAATCCCATCACAACTAGACGTAGGCTTTTACCTTCATCGAAGGGGCCGAACTAGTATAAAAACCGTGTCCTTGTGTCCACTTTAACCCCTTCAAGCTAACCCGTTGCGATGGCTCCACGACTAAGTCCTCTTACTAGGACATCTGCCGTGACAAATCCACGACA

CCTTGTGAACTGAACAACAGCAACAATAGTCAATAGTGTCAAATAGCACAGCGGGCTCCGATATCTCACTTCCAAACTGAAACAAGCTATCAAACAGAAAGTAAAAATCAAGAAACAAAGCAAG

>Aegilops umbellulata 1-325;

ATAAGTATGGGCACTTGACGTCGTACTATTCTCTCGAAAGCTCTTCAGATGTCACAGTTCAGAGATGCGGGCTGATTGTTGGGGTGGACGGTAACCGCATGCGCATGTATAAGGAGGTGGGTCT

TGTCGTGGACTTGTCACGGCAGATGTCCTAGTAAGAGGACTTAGTCGTGGAGCCATCGCAACGGGTTAGCTTGAAGGGGTTAAAGTGGACACAAGGACACGGTTTTTATACTAGTTCGGCCCCTTCGATGAAGGTAAAAGCCTACGTCTAGTTGTGATGGGATTGATGGGTTTCGAGGGCTTAGGGAGCGATATGCTTCGCCTAAACCTCGAGTTCTTCTTCTCTGTCCCAAACCGCCGCCGGGTCATCCCCTTATATACACGGGTGACGCCCGTCGGTCTGCAAAGTCCCAACCTCCGGCTAATAAACGTGCCCGGGTCGGTCTCTCTGTTCCTAACTTACAATACAAGTTTACATCAGGTCGGTTTACACCATAAACCTTTAAACCGATTATGGGCCTTAGGCCCTTCTATCTCTCCTTGGGTTTTAACATCTCGGGTCTGTTTGACGGAGATAACCCGGGCCCTGCAGGCCGGTTTACCCCGGATAGCAACATCCCCAACA
[truncated: 31,876,445 more chars]
